# Rigidified Bis(sulfonyl)ethylenes as Effective Michael Acceptors for Asymmetric Catalysis: Application to the Enantioselective Synthesis of Quaternary Hydantoins

**DOI:** 10.1021/acs.joc.2c02403

**Published:** 2023-01-11

**Authors:** Leire Villaescusa, Iker Hernández, Laura Azcune, Ainhoa Rudi, José M. Mercero, Aitor Landa, Mikel Oiarbide, Claudio Palomo

**Affiliations:** † Departamento de Química Orgánica I, Facultad de Química, Universidad del País Vasco UPV/EHU, Paseo Manuel Lardizabal 3, Donostia-San Sebastián 20018, Spain; ‡ Kimika Fakultatea, Euskal Herriko Unibertsitatea (UPV/EHU) & Donostia International Physics Center (DIPC), Donostia 20018, Spain

## Abstract

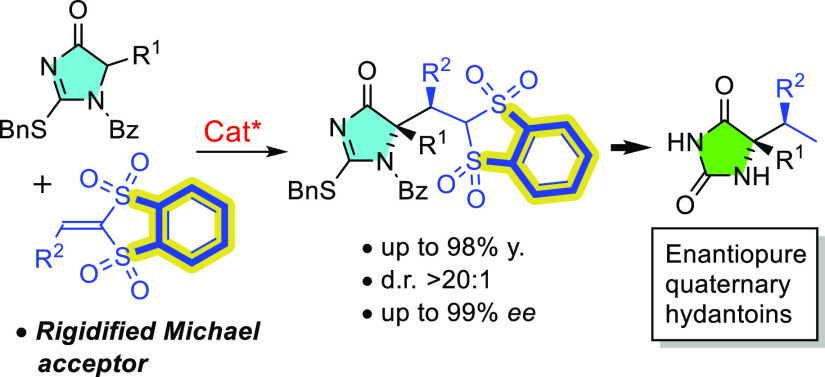

The catalytic, enantio- and diastereoselective addition of hydantoin surrogates **II** to “rigidified” vinylidene bis(sulfone) reagents is developed, thus overcoming the inability of commonly employed β-substituted vinylic sulfones to react. Adducts are transformed in enantioenriched 5,5-disubstituted hydantoins through hydrolysis and reductive desulfonylation processes providing new structures for eventual bioassays. Density functional theory studies that rationalize the observed reactivity and stereoselectivity trends are also provided.

## Introduction

**Figure 1:**
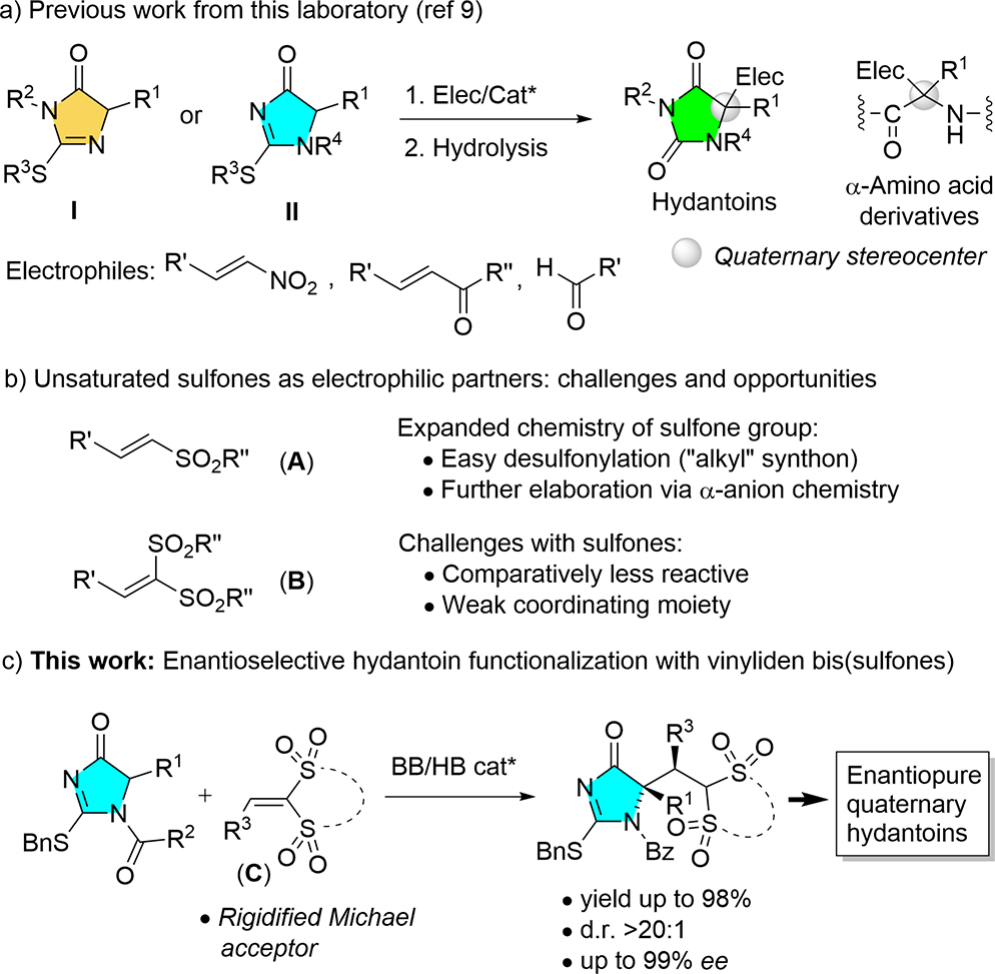
Enantioselective synthesis of quaternary hydantoins from templates **I**/**II** and the new extension using sulfonyl electrophiles.

Hydantoins are widespread heterocyclic scaffolds within biologically active compounds,^1^ and consequently, their chemical synthesis has raised considerable current interest.^2^ In particular, 5,5-disubstituted (quaternary) hydantoin structural subunits are found in marketed drugs^3^ and promising clinical candidates for the treatment of psoriasis^4^ as well as selective androgen receptor modulators.^5^ Compounds possessing α-quaternary hydantoin units also include new potent inhibitors of aggrecanase ADAMTS-5 (involved in cartilage degradation during osteoarthritis^6^) and inhibitors of the decaprenylphospho-β-d-ribofuranose 2-oxidase (DprE1), useful as antimycobacterial inhibitors.^7^ However, the number of stereoselective synthetic approaches to quaternary hydantoins, and more specifically methods involving direct and selective C-functionalization of preformed hydantoins, is still scarce.^8^ Recently, our laboratory has introduced sulfur-substituted dihydroimidazol-4-ones of general structures **I** and **II** as useful hydantoin surrogates amenable for base-promoted C–H functionalization (Figure 1a). More specifically, in the presence of a chiral Brønsted base/H-bonding (BB/HB) bifunctional catalyst, they can react smoothly with active electrophiles, for example, nitroolefins, enones, and aldehydes, affording the α-addition adducts in high yields and very high enantioselectivity for most cases. The resulting adducts may deliver the corresponding 5,5-disubstituted hydantoins or related α-modified α-amino acid derivatives with preserved configuration via hydrolytic protocols.^9^

**Figure 2:**
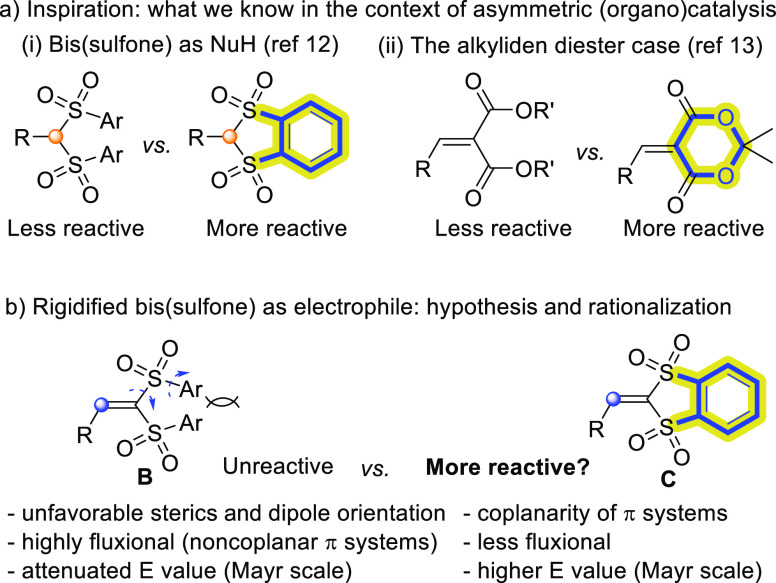
Tunning Nuc/Elec reactivity by substrate rigidification.

In order to expand this technology onto a broader range of α,α-disubstituted hydantoins and α-amino acid derivatives, we envisioned vinyl sulfones as an attractive category of electrophilic reaction partners. Sulfones are recognized as versatile intermediates in synthesis.^10,11^ For instance, they may be transformed into the parent alkanes through reductive desulfonylation or be further elaborated via well-established α-carbanion chemistry. However, preliminary experiments using simple sulfonyl (**A**) and β-substituted bis(sulfonyl)ethylene (**B**) reagents (Figure 1b) in conjunction with surrogates **I**/**II** and suitable BB/HB catalysts led to the recovery of unreacted materials mainly. This observation is ascribable to the relatively low reactivity of α,β-unsaturated sulfonyl systems, particularly the β-substituted ones (vide infra). Here, we present bis(sulfonyl)ethylenes **C** (Figure 2) as competent Michael acceptors in catalytic enantioselective reactions for which common acyclic congeners **B** are not. More specifically, the addition reaction of *N*-acyl surrogates **II** to **C** in the presence of suitable BB/HB catalysts proceeded smoothly at room temperature, affording the Michael reaction adducts as essentially single diastereomers in generally good yields and very high enantioselectivity (Figure 1c). This finding allows us to significantly broaden the range of 5,5-disubstituted hydantoin structures available in optically pure form for eventual biological activity screening programs.

Our selection of **C** as a potentially more reactive Michael acceptor sulfonyl system was routed on previous inspirational observations from the literature. On the one hand, lower reactivity of acyclic versus cyclic bis-sulfonyl alkanes as nucleophiles in iminium-mediated catalytic addition reactions has been reported by our group and others (Figure 2a).^12^ Similarly, the lower Michael acceptor reactivity of (acyclic) alkyliden malonates versus (cyclic) alkyliden Meldrum’s acids, which correlates with the lower carbon acidity of malonic esters versus Meldrum’s acid, is well recognized in the literature.^13^ In addition, Mayr has reported^14^ that, based on kinetic data, aryl-substituted cyclic bis(sulfones) are approximately 1 order of magnitude more electrophilic than their acyclic counterparts. Several attempts to rationalize theoretically these acidity and reactivity trends when comparing acrylic versus cyclic (rigidified) systems are known.^15^ With these precedents in mind, we hypothesized that given the fluxional nature of the four C–S bonds in the acyclic bis-sulfonyl system **B**, its low reactivity may be ascribed to the unfavorable relative orientation of the S=O dipoles of one SO_2_Ph group relative to the other and the two aryl rings relative to one another as a result of steric repulsions. In sharp contrast, the rigid structure of C would keep the S=O groups well aligned for catalyst coordination while the π aryl and olefin systems would stand perfectly coplanar, ultimately leading to highly ordered and compact transition structures.

## Results and Discussion

### Assessment of Pronucleophile Reactivity Trends Using β-Unsubstituted Ethylene Bis(sulfone) **1a**

Since the first organocatalytic conjugate addition to vinyl bis(sulfone) **1a** reported by Mossé and Alexakis in 2005,^16^ the implementation of enantioselective catalytic C–C bond-forming methods involving vinylic sulfones, and vinylidene bis(sulfones) in particular, has progressed unevenly. Reagent **1a** exhibits high reactivity (*E* = −7.50 on the Mayr scale)^14^ and has been often employed as an electrophilic reaction partner under various catalytic activation approaches. However, the sterically more congested β-substituted congeners, for example, **1b**, have been used less often^17^ because of their relatively lower electrophilicity (≈1 unit lower *E* values were reported)^14^ and the appearance of retro-Knoevenagel side reaction.^16c^ In this study, both bis(sulfonyl)olefins **1a** and **1b** along with related reagent **2** displaying a rigidified skeleton were tested in catalytic additions of hydantoin surrogates **I**/**II**.

The study was initiated by evaluating the addition reaction of various dihydroimidazol-4-ones **3** and **4** to bis(sulfonyl)ethylene **1a** using representative bifunctional BB/HB catalysts such as squaramide **C1**, Scheme 2. To our delight, the reaction of *N*-benzoyl dihydroimidazol-4-one **3a** in the presence of 10 mol % **C1** in dichloromethane as the solvent at 0 °C proceeded to almost completion within 24 h to afford product **10a** in 88% ee. Surprisingly, the *N*-acetyl analogue **4a** resulted completely unreactive under the same conditions. Differences in carbon acidity may be invoked to rationalize this huge difference in the reactivity of *N*-phenyl versus *N*-acetyl analogue. In a first estimate, the p*K*_a_ values according to Grzybowski’s prediction tool^18^ for **3a** and **4a** in DMSO are 15 and 16, respectively. In its turn, the “tautomeric” **8** reacted to a significant 80% conversion but produced essentially a racemic material. These results indicated that the present catalytic reaction system is quite sensitive in terms of both reactivity and selectivity to tinny structural variations on the substrate heterocycle. For comparative purposes, the reaction using azlactone 9 was also carried out, which led to full conversion with the formation of adduct 13 in 58% ee. Thus, the relatively higher reactivity of azlactones in this type of catalytic additions^19^ was corroborated.

**Scheme 1:**
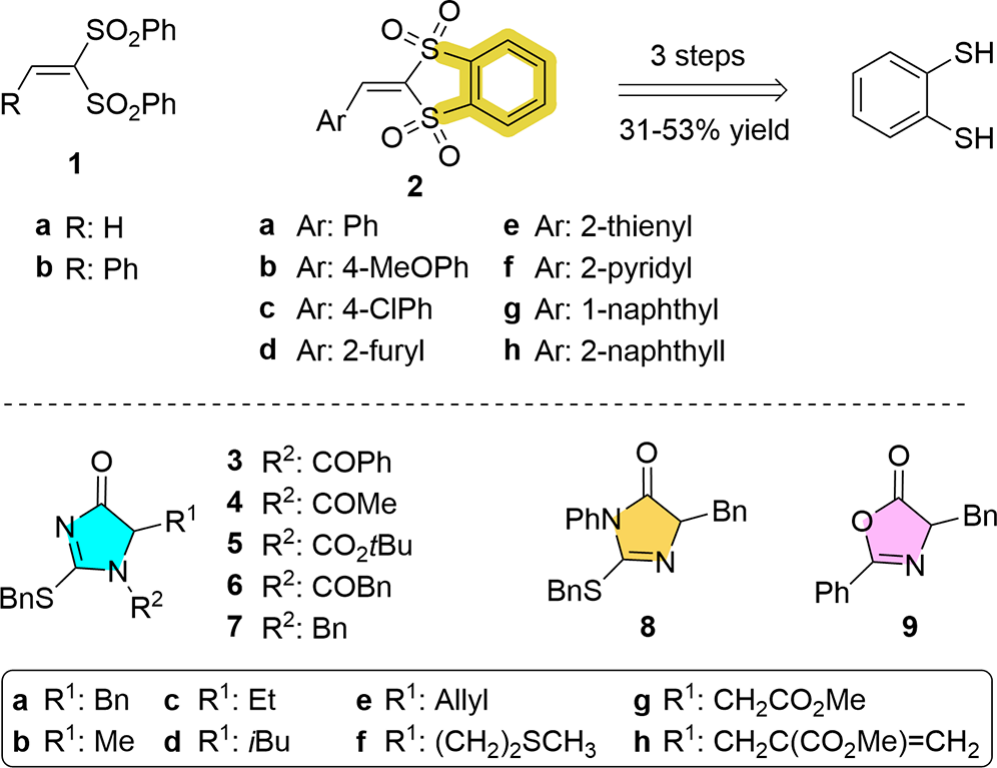
Vinylidene Bis(sulfones) and Pronucleophilic Heterocycles Employed in This Study

**Scheme 2:**
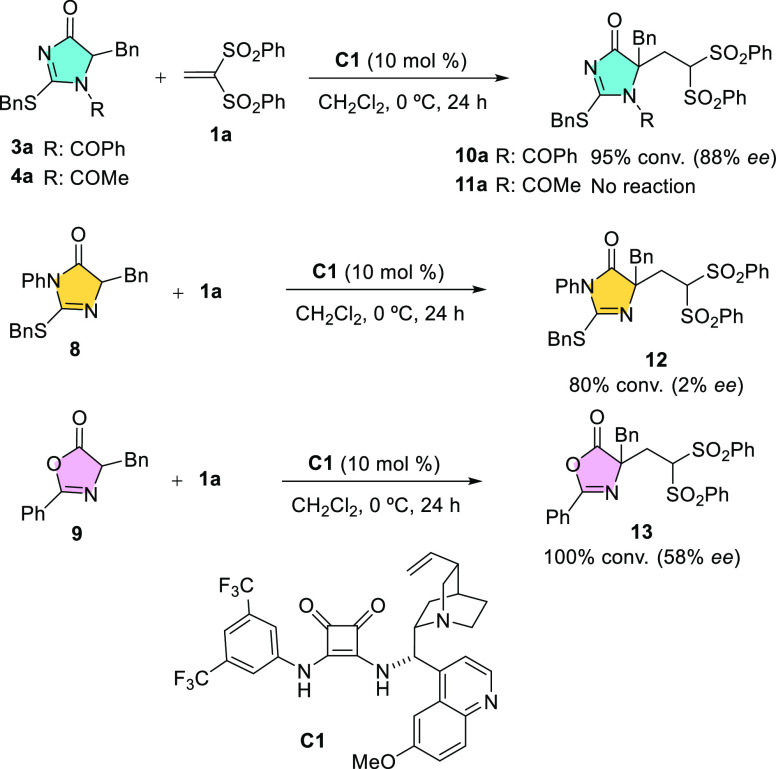
Evaluation as Several Pronucleophiles against the Catalytic Addition Reaction to Bis(sulfone) **1a**

After this brief substrate screening, several other catalysts **C2–C6** with varying structure and functionality were evaluated for the model reaction between **1a** and **3a**. As the results in Table 1 show, catalyst **C2**, which has been developed in our group and presents an additional amide NH available for engaging in H-bonding interactions,^9,20^ afforded an increased 98% ee (entry 2 vs 1). Takemoto’s catalyst **C6**(21) (entry 6) and the related urea and thiourea catalysts **C3**(22) and **C4**(23) (entries 3 and 4) did also promote the reaction, although neither yields nor enantioselectivities were improved. Finally, the ureidoaminal **C5**, which also has an additional NH group and demonstrated highly active and selective catalysts for various reactions,^24^ failed to promote this reaction effectively (entry 5).

**Table 1:**
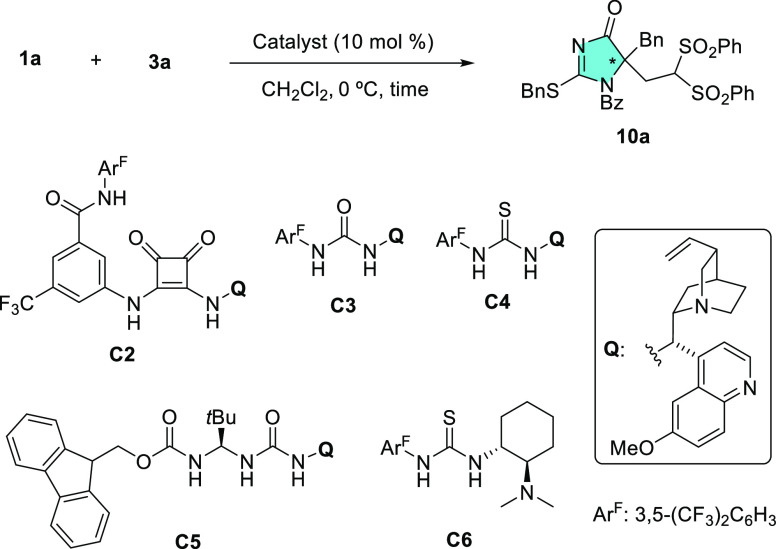
Catalyst Screening for the Addition of **1a** to **3a**

Entry	Catalyst	Time (h)^a^	Conv. (%)^b^	ee (%)^c^
1	**C1**	24	95	88
2	**C2**	24	75	98
4	**C3**	48	82	75
5	**C4**	48	68	67
6	**C5**	24	63	29
7	**C6**	24	88	18

a Reaction conditions: **3a** (0.1 mmol), **1a** (0.12 mmol), and catalyst (10 mol %) in CH_2_Cl_2_ (1.0 mL).

b Conversion determined by ^1^H NMR.

c ee determined by HPLC.

**Scheme 3:**
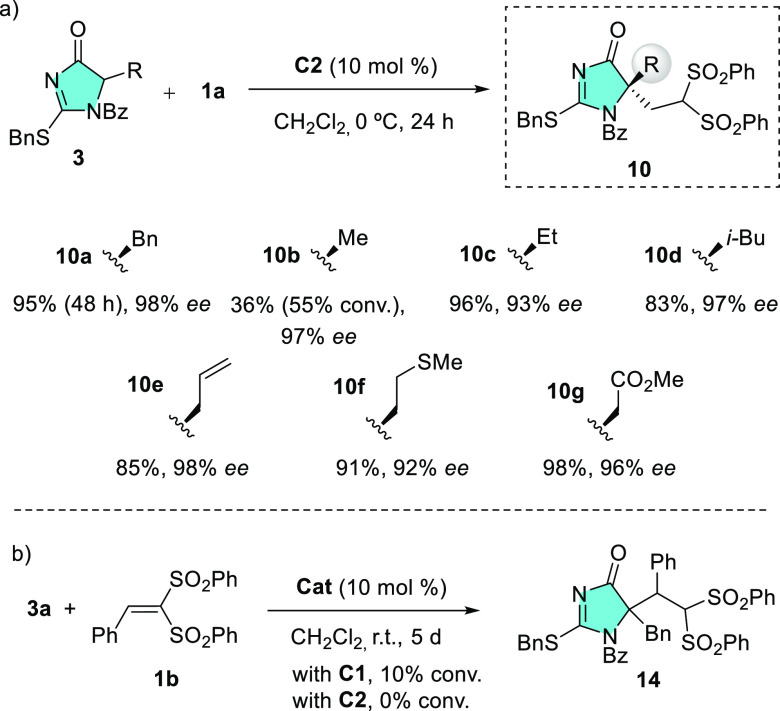
(a) Scope of Heterocycles **3** Suitable for the Catalytic Addition to **1a** and (b) the Attenuated Reactivity of Acceptor **1b**

With **C2** selected as an optimal catalyst, the scope of the reaction was briefly explored. As the results in Scheme 3a show, the reaction of **1a** with **3** bearing simple alkyl or allyl substituents at **C5** proceeded satisfactorily giving rise to products **10b–e** in ee’s in between 93 and 98% and generally high yields (adduct **10b** was an exception). The reactions leading to adducts **10f** and **10g** also worked well, affording the respective product in 91%/98% yield and 92%/96% ee, thus showing that substrates bearing thioether and ester functions are well tolerated. However, as data in Scheme 3b show, phenyl-substituted bis(sulfonyl)ethene **1b** was not reactive enough, and only marginal conversion was attained after prolonged time at room temperature.

### Catalytic Addition Reactions Using Rigidified β-Substituted Ethylene Bis(sulfone) **2**

**Scheme 4:**
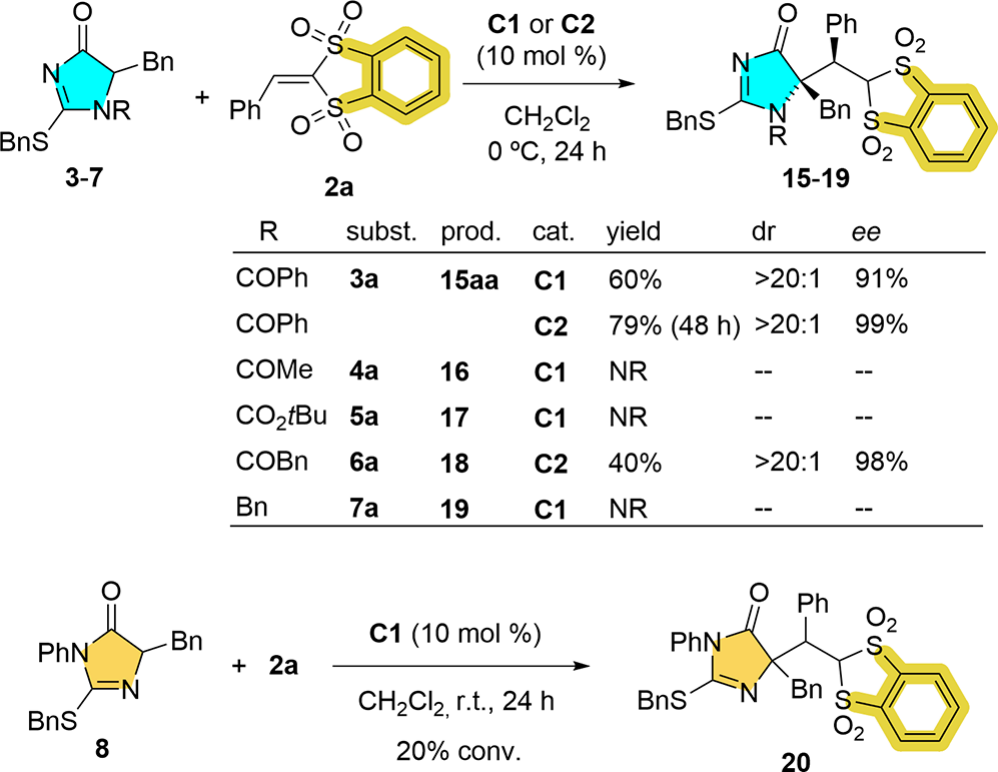
Initial Assessment of Reagent **2a** as a Michael Acceptor in Catalysis

Prompted by this result, our attention turned to the rigidified reagents C. Preparation of 2-benzylidene-2*H*-benzo[*d*][1,3]dithiole 1,1,3,3-tetraoxides **2a** and **2b** in one step from benzodithiole tetroxide was reported by Mayr in 75 and 77% yields, respectively. Following a slightly modified three-step sequence from commercially available *o*-benzenedithiol (Scheme 1), the remaining compounds **2c–f** were obtained in an overall 31–53% yield.^25^ With reagent **2a** at hand, its behavior as a Michael acceptor in the above catalytic reactions was investigated (Scheme 4). Gratifyingly, the reaction of **2a** with **3a** in the presence of 10 mol % **C1** proceeded to almost completion after 24 h at 0 °C, from which 60% of adduct **15aa** of 91% ee could be isolated. Once again, catalyst **C2** imparted almost perfect stereoinduction providing a single enantiomer of **15aa** in 79% yield after 48 h at the same temperature. The *N*-acyl analogues **4–6** and the “tautomeric” dihydroimidazol-4-one 8 were less efficient pronucleophiles against the new reagent **2a** (Scheme 4). Not surprisingly, the *N*-benzyl analogue **7a** was also totally unreactive under the present catalytic conditions.

Encouraged by the good reactivity profile showed by reagent **2a**, the remaining analogues **2b–2f** were also evaluated in combination with a variety of pronucleophiles **3** (Table 2). In the first set of reactions in the presence of **C2**, **2a** was submitted to the reaction with alkyl- and allyl-substituted dihydroimidazolones **3c**, **3d**, and **3e** which led to the corresponding adducts **15ca**, **15da**, and **15ea** as single diastereomer in high yields and enantioselectivities of 96, 92, and 95%, respectively. The thioether- and methyl ester-bearing substrates **3f** and **3g** also led to the addition of adducts **15fa** and **15ga** in high yield and diastereoselectivity, although the latter was obtained with slightly diminished enantioselectivity unless reaction temperature was decreased to −20 °C. The reaction of unsaturated ester-bearing **3h** to afford **15ha** proceeded exceedingly (91% ee), demonstrating that the present catalytic conjugate addition reactions may proceed chemoselectively in the presence of additional Michael acceptor units in the substrate. Then, several aryl-substituted acceptors **2** were screened. *p*-Methoxyphenyl-substituted acceptor **2b** was equally competent to give rise to **15ab** in a highly selective manner. Similarly, the *p*-chlorophenyl-substituted analogue **2c** reacted to completion within 2 days regardless of the temperature with the dihydroimidazolones **3a**, **3b**, and **3e**, affording products **15ac**, **15bc**, and **15ec** in good yields and excellent enantiocontrol. The reactions with 1-naphthyl and 2-naphthyl-bearing vinyl sulfones **2g** and **2h** did also work satisfactorily to produce compounds **15ge** and **15ah** in good yields and high stereoselectivity. Interestingly, **15ah** presented split signals in ^1^H NMR, which were assigned to the existence of rotameric isomers. That is why this compound was characterized as the corresponding hydantoin derivative after hydrolytically removing both the *N*-benzoyl and benzylthio groups (see the Supporting Information for details). On the other hand, bis(sulfones) **2d–f**, bearing a heteroaryl β-substituent, were also tolerated. The furyl and pyridyl derivatives **15ad** and **15af** were obtained in good yields and very high stereoselectivity. The thiophenyl-substituted adducts **15ae** and **15ge** were isolated with somewhat reduced yields and, in the latter case, diminished selectivity too. Finally, the method is applicable at a larger scale without any significant variation in yields or selectivities. For instance, in reactions carried out at a 4 mmol scale, 2.18 g (77%) and 2.43 g (82%) of adducts **15aa** and **15ac**, respectively, were obtained in both cases with almost perfect enantioselectivity of 99% ee (see the Supporting Information for details).

**Table 2:**
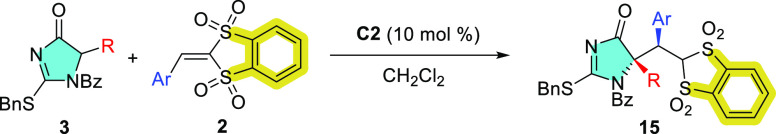

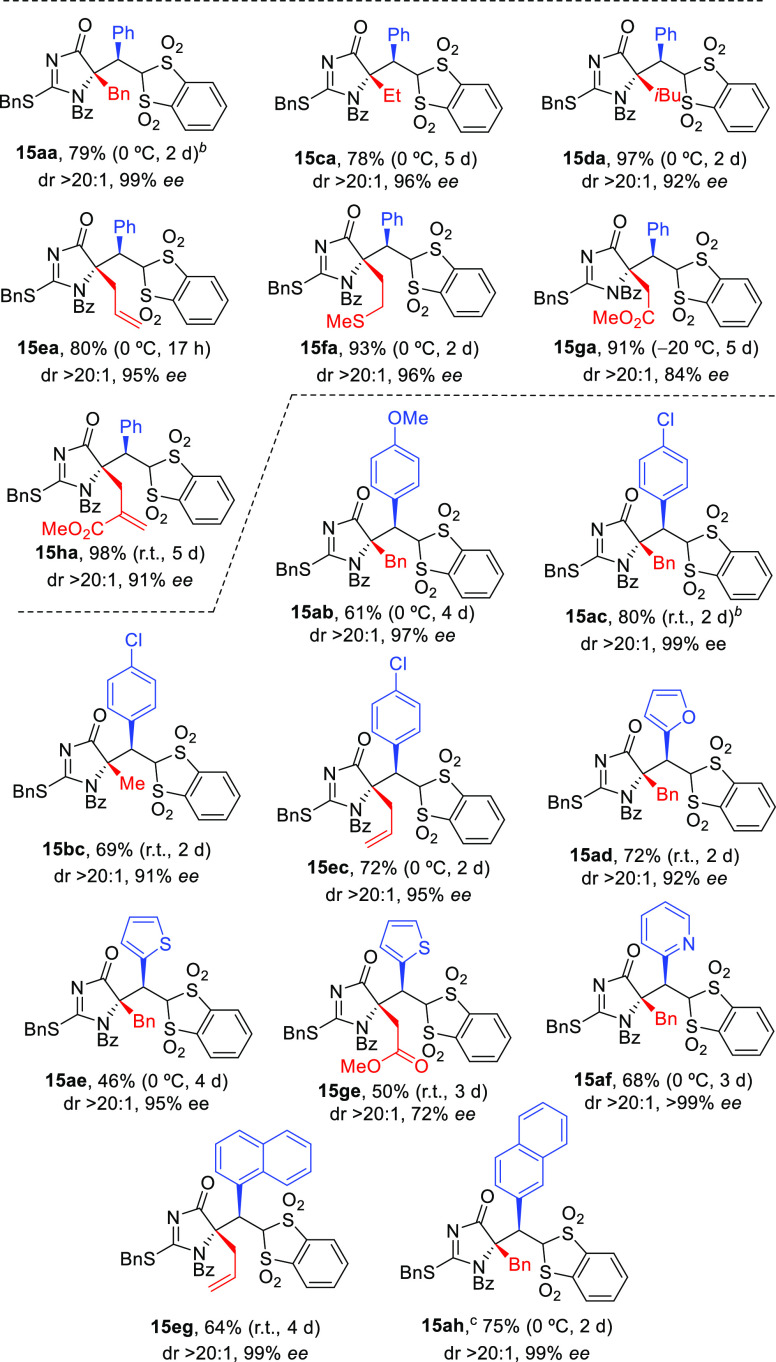
Scope of the Reaction between Hydantoin Surrogate **3** and Acceptor **2** in the Presence of Catalyst **C2**^a^

a Reactions conducted on a 0.1 mmol scale in 1 mL of CH_2_Cl_2_; mol ratio of **3**/**2**/**C2** 1:1.2:0.1. Yield of isolated product after column chromatography. ee’s determined by HPLC analysis using a chiral stationary phase.

b Reaction run at a 4 mmol scale using 5 mol % **C2** as a catalyst.

c Obtained as a mixture of rotamers.

**Scheme 5:**
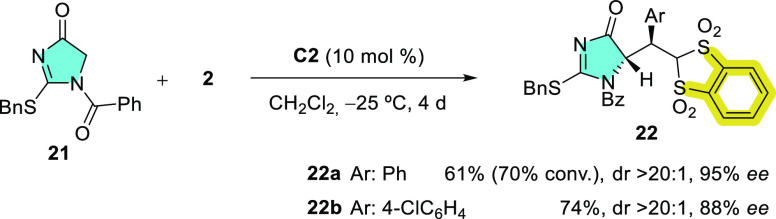
Enantio- and Diastereoselective Addition of 5-Unsubstituted Dihydroimidazol-4-One **21** to Acceptors **2**

Having established **2** as a competent Michael acceptor reagent for the enantioconvergent transformations involving chiral racemic pronucleophiles **3**, the likelihood of the parent unsubstituted dihydroimidazolone **21** participating in such enantioselective transformations was assessed next (Scheme 5). It could be anticipated that a major difficulty would be associated with the configurational integrity of the C_α_ stereocenter in the product **22** in the presence of the basic catalyst. Accordingly, reactions were carried out in cryogenic conditions. As the results in Scheme 5 show, it was delighting to observe that even at −25 °C, the reaction of **21** with bis(sulfone) **2a** in the presence of 10 mol % **C2** proceeded to afford product **22a** as a single diastereomer in reasonably good yield (61, 70% conv.) and 95% ee. Similarly, the reaction with *p*-chlorophenyl derivative **2b** afforded product **22b** in 74% yield and 88% ee. At this point, it is important to note that product **22** did not epimerize during column chromatography purifications on silica gel.

**Scheme 6:**
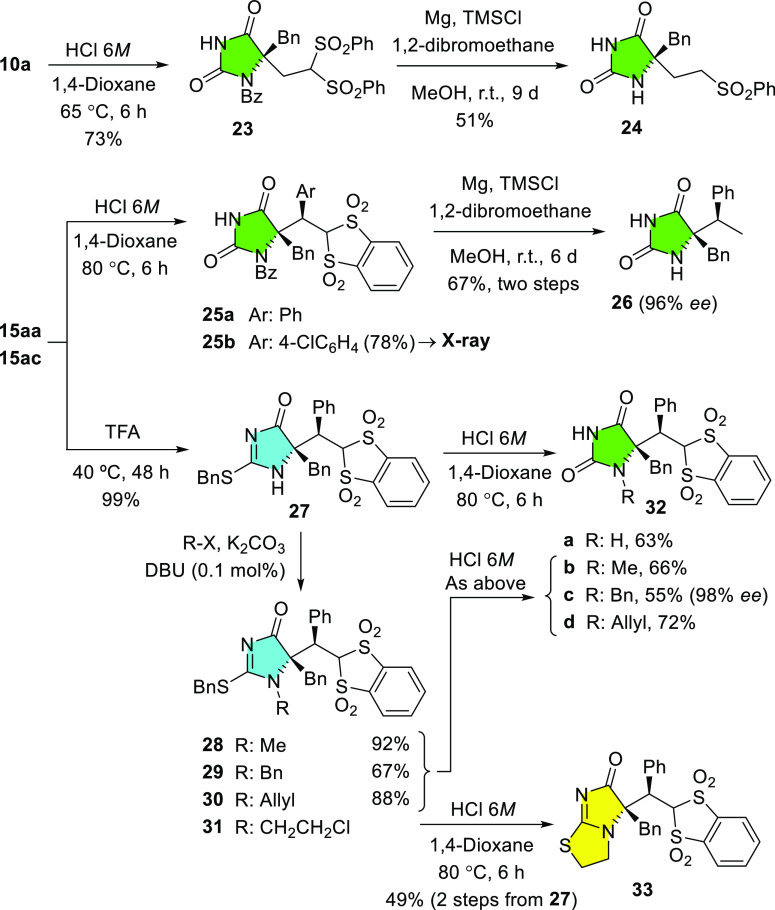
Chemical Elaboration of Adducts into Hydantoins and Derivatives Thereof

Then, some possibilities of further chemical elaboration of enantioenriched adducts were explored, particularly the hydrolysis of the heterocyclic ring and the reductive elimination of the sulfonyl moiety (Scheme 6). For example, treatment of **10a** with 6 M HCl in 1,4-dioxane at 65 °C led to hydantoin **23** in 73% yield. Desulfonylation^26^ of **23** with Mg/TMSCl/1,2-dibromoethane in methanol at room temperature afforded, unexpectedly and selectively, the monodesulfonylation product **24** in 51% yield. This case of selective monodesulfonylation of a bis-sulfonylated adduct is relevant because the alternative and direct route to the monosulfonyl derivative through catalytic addition of the dihydroimidazolone **3a** to phenylsulfonylethene did not work even at 70 °C overnight. Acidic hydrolysis at 80 °C (bath temperature) of adducts **15aa** and **15ac** gave rise to *N*-benzoyl hydantoins **25a** and **25b** in good yields.^27^ An X-ray crystal structure analysis of **25b** allowed us to establish its absolute and relative configurations.^28^ The configuration of the remaining adducts was assigned assuming a uniform reaction mechanism. Double desulfonylation of **25a** under the above conditions yielded the 5,5-disubstituted hydantoin **26** in 67% yield over the two steps from **15aa**. On the other hand, removing the *N*-benzoyl group from **15aa** could be carried out by treatment with TFA at 40 °C, leading to **27** in essentially quantitative yield. With the NH derivative **27** in hand, hydrolysis led to hydantoin **32a**; alternatively, various alkyl and allyl groups could be installed at nitrogen via standard *N*-alkylation protocols leading to **28–31** and thus overcoming the inability of *N*-alkyl dihydroimidazol-4-ones (e.g., **7a**, Scheme 4) to participate in the above catalytic addition reaction. Submission of the *N*-alkyl derivatives **28–30** to acid hydrolysis led to *N*-alkyl hydantoins **32b–d**. Surprisingly, hydrolysis of adduct **31** followed a divergent pathway and afforded bicyclic isothiourea **33**, probably through a chloride anion-promoted *S*-debenzylation/intramolecular *S*-alkylation cascade. Determination of the enantiomeric purity of product **32c** (98% ee) served to prove that the full sequence, including *N*-deprotection, *N*-alkylation, and final hydrolysis, proceeded with preserved stereochemistry.

### Theoretical Rationalization of the Observed Reactivity Trends and Stereoselectivity

**Figure 3:**
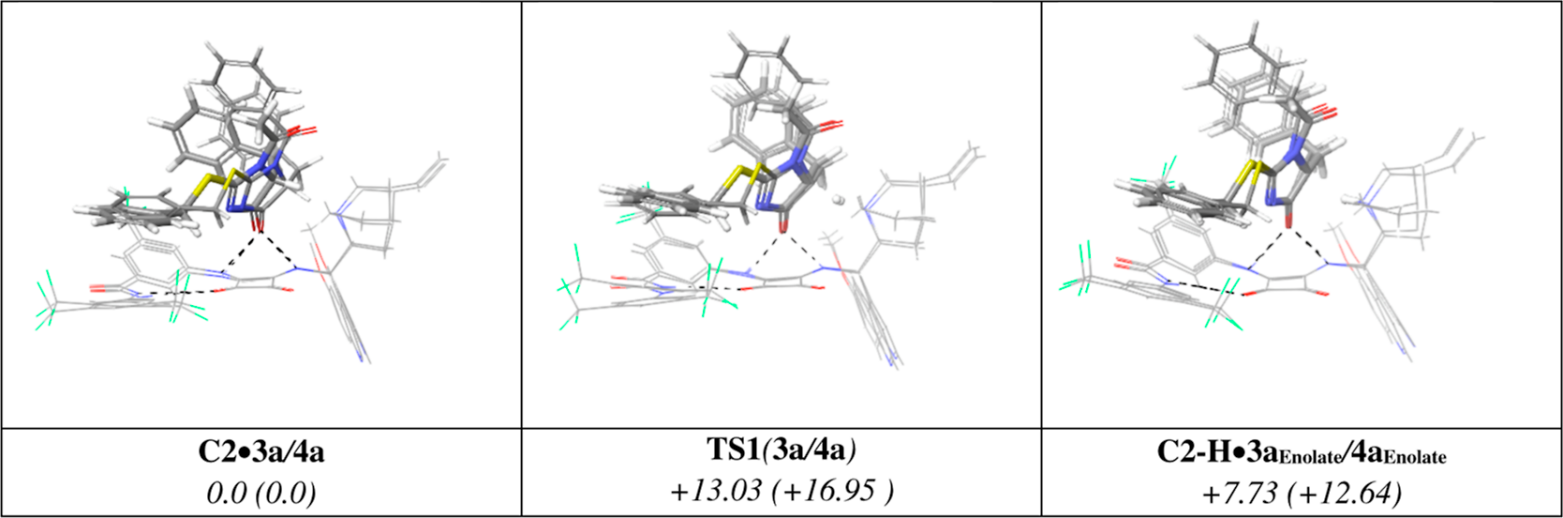
Catalyst–reactant complex, reaction **TS1** for the reactant deprotonation, and protonated catalyst–enolate complex corresponding to the proton transfer step for both **3a** and **4a**. Energies in kcal/mol.

A theoretical analysis was undertaken in order to understand (a) the huge differences in reactivity between the *N*-benzoyl heterocycle **3** and the *N*-acetyl analogue **4** observed experimentally and (b) the stereoselectivity and sense of chiral induction in the above catalytic reactions. To ascertain whether the higher reactivity of **3a** versus **4a** was attributable, as hypothesized above, to differences in the carbon acidities among these two pronucleophiles, we first calculated the p*K*_a_ values for **3a** and **4a** using the Jaguar p*K*_a_ module^29^ as implemented in the Schrodinger 2021-01^30^ program suite. In both water and DMSO as a solvent, the calculated p*K*_a_ of **3a** is smaller than that of **4a**, 11.56 versus 12.51 in water and 19.71 versus 21.15 in DMSO, respectively. These differences are in agreement with our initial gross estimates (vide supra) and correlate well with the observed reactivity trend. Subsequently, the energy barrier was calculated for the deprotonation step of both **3a** and **4a** by the action of catalyst **C2**. In this step, a proton from the α-position of either substrate is transferred to the catalyst quinuclidine nitrogen via **TS1** leading to complexes **C2–H·3a**_**enolate**_ and **C2–H·4a**_**enolate**_, with energy barriers of 13.03 and 16.95 kcal/mol, respectively (Figure 3). The difference between both energy barriers (3.92 kcal/mol) is appreciable and may justify the significant reactivity difference observed experimentally for both substrates.

**Figure 4:**
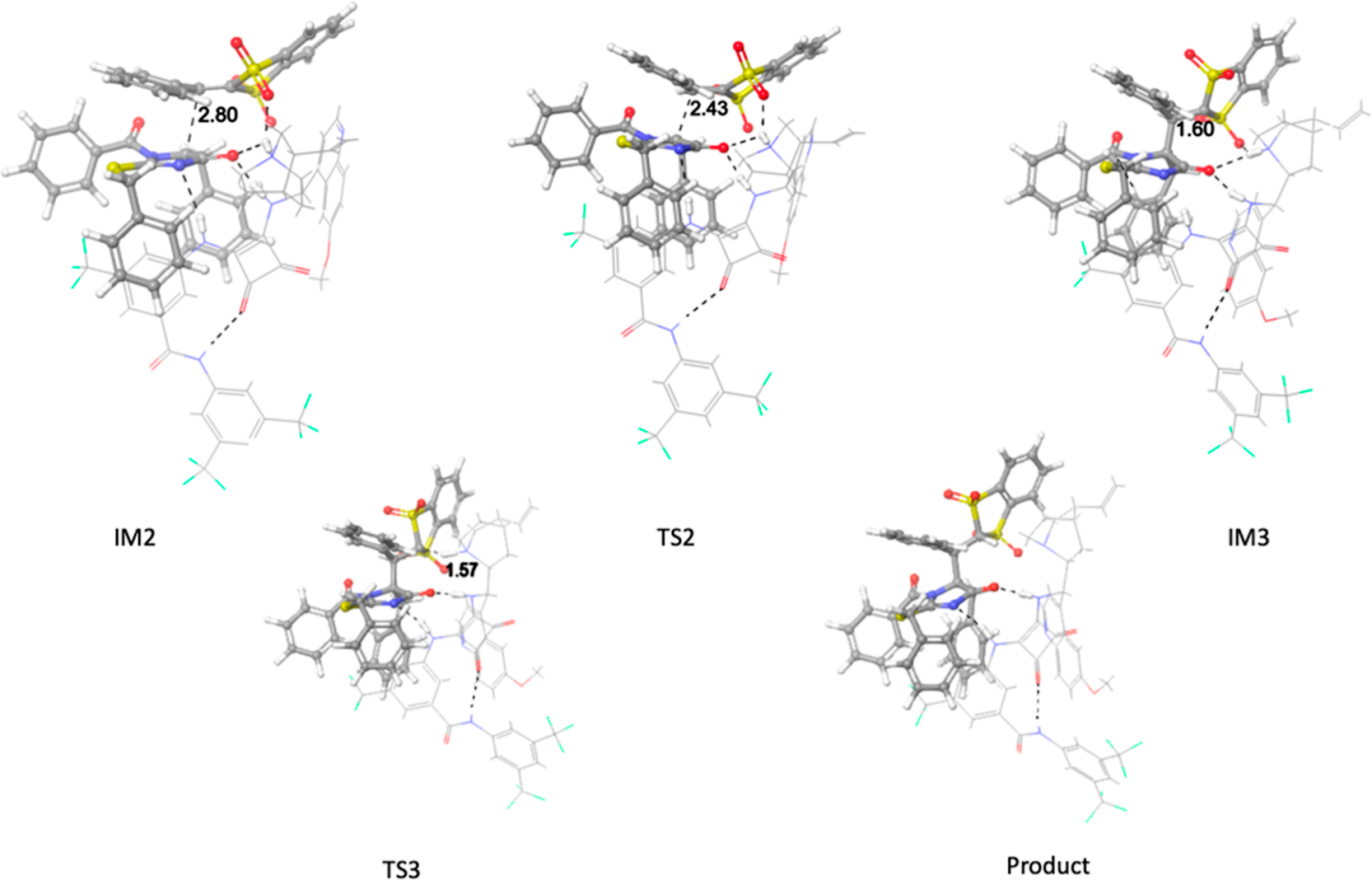
Structures participating in steps 2 and 3 of the reaction. In the first row, the transition state **TS2** of the C–C formation step, with the corresponding intermediates, and in the third row, **TS3** for the third step corresponding to the proton transfer from protonated **C2** to the final product **15aa**.

In an attempt to understand the stereoselectivity of the reaction, we have analyzed the C–C formation step, which will dictate both the product relative and absolute configuration. Four different transition states were located (see Supporting Information for calculations details) that correspond to different orientations of reactants, out of which **TS2** was the lowest in energy (Figure 4). In this transition state, each squaramide NH group of the catalyst interacts with the enolate from **3a** in accordance with the so-called Pápai model. In comparison, transition state **TS2-B** (see Supporting Information), which would lead to the corresponding enantiomeric product, is 4.40 kcal/mol higher in energy. The energy difference could be attributed to the additional H-bond formed between the protonated quinuclidine moiety of the catalyst and the enolate oxygen in **TS2**. The remaining two transition states **TS2-C** and **TS2-D** are 5.9 and 9.3 kcal/mol higher in energy than **TS2** and present a single H-bond interaction between the enolate oxygen and the catalyst (see Supporting Information for details).

**Figure 5:**
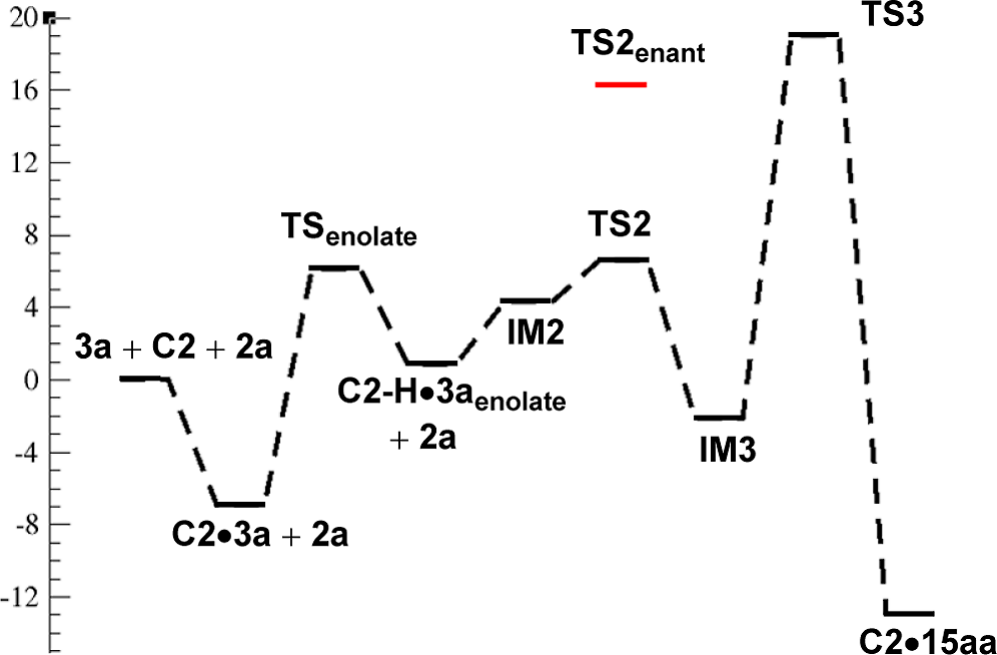
Reaction profile. Relative Gibbs free energy values in kcal mol^–1^ calculated with Orca 5 (see Supporting Information for more details).

In the last step of the catalytic cycle, the proton will be transferred back from the protonated catalyst to the formed Michael adduct delivering product **15aa** via **TS3**. In **TS3**, the product–catalyst interaction involving the dihydroimidazolinone and the squaramide moieties, respectively, changes, and now the squaramide two NH groups interact with one of the dihydroimidazolinone carbonyls only. This new arrangement of the H-bonds causes this transition state to be around 15 kcal/mol higher in energy. Note though that the final proton transfer to the anionic reaction adduct might also occur via other alternative mechanisms. Figure 5 shows collectively the various reaction elementary steps for the lowest in the energy pathway from reactants **3a** and **2a** in the presence of catalyst **C2**.

## Conclusions

In conclusion, the catalytic asymmetric conjugate addition of hydantoin surrogates to vinyl sulfones has been developed using a secondary amide-bearing tertiary amine/squaramide bifunctional catalyst. *N*-Benzoyl 2-(benzylthio)-1,5-dihydro-4*H*-imidazole-4-ones, for example, **3**, are able to act as hydantoin surrogates and react with vinyl bis(sulfone) **1a** smoothly to provide the corresponding adducts in good yield and stereoselectivity. In contrast, the β-substituted vinyl bis(sulfones), such as **1b**, proved to be completely unreactive under the above catalytic conditions. This problem could be circumvented by employing the “rigidified” β-substituted vinyl sulfones **2** instead. Ulterior acid hydrolysis of the heterocycle system in adducts combined with a desulfonylation process allowed to access a variety of 5-substituted hydantoins, including the 5,5-disubstituted quaternary ones, in essentially optically pure form for eventual applications in medicinal chemistry. The suitability of “rigidified” β-substituted vinyl sulfones **2** as Michael acceptors in other unrelated catalytic addition reactions may be foreseen.

## Experimental Section

### General Information

All nonaqueous reactions were performed under an inert atmosphere using oven-dried glassware and were magnetically stirred. For reactions that require heating, an oil bath was used. Yields refer to chromatographically purified samples unless otherwise stated. Wet organic layers were dried over MgSO_4_, and solvents were evaporated under reduced pressure. For trace solvent removal, a vacuum pump (≈0.5 mmHg) was applied. Column chromatography was performed on ROCC 60 silica gel 40–63 μm as the stationary phase and a suitable mixture of solvents (typically hexane: ethyl acetate) as the eluent. Optical rotations were recorded using a Jasco P-2000 polarimeter. Melting points were determined in open capillaries in a Stuart SHP3 melting point apparatus. ^1^H NMR and ^13^C NMR spectra were recorded at 300 or 500 MHz and 75 or 126 MHz, respectively. The chemical shifts are reported in ppm relative to CDCl_3_ (δ = 7.26) and CD_2_Cl_2_ (δ = 5.32) for ^1^H NMR and relative to the central resonances of CDCl_3_ (δ = 77.2) and CD_2_Cl_2_ (δ = 53.8) for ^13^C NMR. Peaks are labeled as singlet (s), broad singlet (bs), doublet (d), triplet (t), quartet (q), double doublet (dd), double triplet (dt), double of doublet of triplets (ddt), quartets of doublets (qd), or multiplet (m). Coupling constants (*J*) are reported in Hertz (Hz). Mass spectra were recorded on an ESI-ion trap mass spectrometer (Agilent 1100 series LC/MSD, SL model) and a UPLC–DAD–QTOF, ultra-high-performance liquid chromatography–mass spectrometer. Enantiomeric (ee) values were determined by HPLC performed on Waters 600-E (equipped with a 2998 photodiode array UV detector) employing Daicel Chiralpack columns (IA, IB, IC, and IF). Infrared spectra were measured employing a Bruker ALPHA-P compact FT-IR spectrometer. The X-ray diffraction analysis was conducted by the General Research Service (SGIker) of UPV/EHU.

All reagents were purchased from commercial suppliers and used without further purification, unless otherwise stated. Substrates **1a**, **1b**, **3a**, **3b**, **3c**, **3d**, **3f**, **4a**, **5a**, **6a**, **7a**, **8**, and **9** were synthesized according to the reported procedures (see the Supporting Information for details). Triethylamine was purified by distillation. Dichloromethane and acetonitrile were dried over CaH_2_, and DMF was dried over molecular sieves. Analytical reagent-grade MeOH and toluene were used without further drying.

### General Procedure for the Catalytic Addition of Hydantoin Surrogates **3** to **1a**

In a 5 mL test tube, the corresponding pronucleophile (0.1 mmol, 1 equiv) was dissolved in CH_2_Cl_2_ (1 mL) at room temperature, and after cooling the solution down to 0 °C, the corresponding vinylic sulfone (37 mg, 0.12 mmol, 1.2 equiv) and catalyst **C2** (8 mg, 0.01 mmol, 10 mol %) were added. The mixture was stirred at 0 °C until the reaction was finished as monitored by ^1^H NMR. The crude product was directly submitted to silica gel flash column chromatography (eluent: hexane/ethyl acetate, from 3:1 to 1:1).

#### (*S*)-1-Benzoyl-5-benzyl-2-(benzylthio)-5-(2,2-bis(phenylsulfonyl)ethyl)-1,5-dihydro-4*H*-imidazole-4-one (**10a**)

The title compound was prepared from 1-benzoyl-5-benzyl-2-(benzylthio)-1,5-dihydro-4*H*-imidazole-4-one (40 mg, 0.1 mmol) according to the general procedure. Silica gel flash column chromatography (eluent: hexane/ethyl acetate, from 3:1 to 1:1). White foam. Yield: 67 mg, 95%. [α]_D_^20^ + 47.0 (*c* = 1, 98% ee, CH_2_Cl_2_). ^1^H NMR (300 MHz, CDCl_3_): δ 8.15–6.94 (m, 25H), 5.67 (dd, *J* = 6.1, 3.0 Hz, 1H), 4.20 (d, *J* = 13.3 Hz, 1H), 4.04 (d, *J* = 13.3 Hz, 1H), 3.54 (d, *J* = 13.6 Hz, 1H), 3.33 (dd, *J* = 16.6, 3.0 Hz, 1H), 3.22 (d, *J* = 13.8 Hz, 1H), 3.19–3.11 (m, 1H). ^13^C{^1^H} NMR (75 MHz, CDCl_3_): δ 186.2, 185.0, 168.2, 138.0, 137.0, 134.9, 134.7, 134.4, 134.0, 133.1, 132.1, 130.3, 130.2, 130.0, 129.8, 129.3, 129.1, 128.9, 128.78, 128.76, 128.6, 128.0, 127.7, 77.9, 73.2, 41.2, 39.7, 31.6. HRMS (ESI) *m*/*z*: [M + H]^+^ calcd for C_38_H_33_N_2_O_6_S_3_, 709.1501; found, 709.1506. IR (cm^–1^): 3062, 3056, 2940, 1725, 1600. The ee value was determined by HPLC analysis (Daicel Chiralpak IC, hexane/isopropanol 30:70), flow rate: 0.5 mL/min, retention times: 43.8 min (major) and 52.0 min (minor).

#### (*S*)-1-Benzoyl-2-(benzylthio)-5-(2,2-bis(phenylsulfonyl)ethyl)-5-methyl-1,5-dihydro-4*H*-imidazole-4-one (**10b**)

The title compound was prepared from 1-benzoyl-2-(benzylthio)-5-methyl-1,5-dihydro-4*H*-imidazole-4-one (32 mg, 0.1 mmol) according to the general procedure. Silica gel flash column chromatography (eluent: hexane/ethyl acetate, from 3:1 to 1:1). Yellow foam. Yield: 23 mg, 36%. (conv. 55%). [α]_D_^20^ – 3.3 (*c* = 1, 97% ee, CH_2_Cl_2_). ^1^H NMR (300 MHz, CDCl_3_): δ 8.14–8.05 (m, 2H), 8.02–7.93 (m, 2H), 7.75–7.36 (m, 12H), 7.24 (m, 4H), 5.56 (dd, *J* = 5.8, 3.1 Hz, 1H), 4.47–4.34 (m, 2H), 3.19 (dd, *J* = 16.5, 3.1 Hz, 1H), 2.99 (dd, *J* = 16.5, 5.8 Hz, 1H), 1.57 (s, 3H). ^13^C{^1^H} NMR (75 MHz, CDCl_3_): δ 186.9, 183.9, 168.0, 138.2, 136.8, 134.9, 134.61, 134.56, 133.3, 133.2, 130.4, 130.0, 129.29, 129.26, 129.1, 128.9, 128.8, 128.1, 77.3, 68.1, 39.6, 31.5, 22.4. HRMS (ESI) *m*/*z*: [M + H]^+^ calcd for C_32_H_29_N_2_O_6_S_3_, 633.1182; found, 633.1192. IR (cm^–1^): 3062, 2931, 1728, 1681. The ee value was determined by HPLC analysis (Daicel Chiralpak IA, hexane/isopropanol 30:70), flow rate: 0.5 mL/min, retention times: 32.8 min (major) and 39.7 min (minor).

#### (*S*)-1-Benzoyl-2-(benzylthio)-5-(2,2-bis(phenylsulfonyl)ethyl)-5-ethyl-1,5-dihydro-4*H*-imidazole-4-one (**10c**)

The title compound was prepared from 1-benzoyl-2-(benzylthio)-5-ethyl-1,5-dihydro-4*H*-imidazole-4-one (34 mg, 0.1 mmol) according to the general procedure. Silica gel flash column chromatography (eluent: hexane/ethyl acetate, from 3:1 to 1:1). White foam. Yield: 62 mg, 96%. [α]_D_^20^ + 19.7 (*c* = 1, 93% ee, CH_2_Cl_2_). ^1^H NMR (300 MHz, CDCl_3_): δ 8.11–8.03 (m, 2H), 7.98–7.92 (m, 2H), 7.74–7.63 (m, 2H), 7.62–7.52 (m, 8H), 7.48–7.35 (m, 2H), 7.23 (m, 4H), 5.56 (dd, *J* = 6.0, 2.9 Hz, 1H), 4.39 (s, 2H), 3.13 (dd, *J* = 16.6, 3.0 Hz, 1H), 3.00 (dd, *J* = 16.6, 6.0 Hz, 1H), 2.27 (dq, *J* = 14.5, 7.3 Hz, 1H), 1.82 (dq, *J* = 14.4, 7.3 Hz, 1H), 0.72 (t, *J* = 7.3 Hz, 3H). ^13^C{^1^H} NMR (75 MHz, CDCl_3_): δ 186.3, 184.8, 167.9, 138.2, 136.9, 134.8, 134.7, 134.6, 133.3, 133.2, 130.3, 130.1, 129.29, 129.26, 129.1, 128.92, 128.85, 128.1, 77.4, 72.8, 39.7, 31.4, 28.9, 8.2. HRMS (ESI) *m*/*z*: [M + H]^+^ calcd for C_33_H_31_N_2_O_6_S_3_, 647.1344; found, 647.1340. IR (cm^–1^): 3062, 2971, 2934, 1726, 1682. The ee value was determined by HPLC analysis (Daicel Chiralpak IF, hexane/isopropanol 30:70), flow rate: 0.5 mL/min, retention times: 44.9 min (major) and 97.4 min (minor).

#### (*S*)-1-Benzoyl-2-(benzylthio)-5-(2,2-bis(phenylsulfonyl)ethyl)-5-isobutyl-1,5-dihydro-4*H*-imidazole-4-one (**10d**)

The title compound was prepared from 1-benzoyl-2-(benzylthio)-5-isobutyl-1,5-dihydro-4*H*-imidazole-4-one (37 mg, 0.1 mmol) according to the general procedure. Silica gel flash column chromatography (eluent: hexane/ethyl acetate, from 3:1 to 1:1). White foam. Yield: 56 mg, 83%. [α]_D_^20^ + 11.5 (*c* = 1, 97% ee, CH_2_Cl_2_). ^1^H NMR (300 MHz, CDCl_3_): δ 8.16–8.04 (m, 2H), 8.04–7.93 (m, 2H), 7.75–7.39 (m, 12H), 7.23 (s, 4H), 5.51 (dd, *J* = 5.9, 2.9 Hz, 1H), 4.39 (s, 2H), 3.14 (dd, *J* = 16.6, 2.9 Hz, 1H), 2.95 (dd, *J* = 16.6, 5.9 Hz, 1H), 2.10 (dd, *J* = 14.2, 4.9 Hz, 1H), 1.53 (dd, *J* = 14.1, 7.8 Hz, 1H), 1.39 (dq, *J* = 19.4, 6.5 Hz, 1H), 0.75 (dd, *J* = 7.3, 6.6 Hz, 6H). ^13^C{^1^H} NMR (75 MHz, CDCl_3_): δ 186.5, 184.4, 167.9, 138.3, 137.2, 134.8, 134.7, 134.6, 133.3, 133.1, 130.2, 130.1, 129.3, 129.2, 129.1, 128.81, 128.76, 128.7, 128.0, 77.5, 71.5, 43.0, 39.6, 32.8, 24.9, 23.8, 22.8. HRMS (ESI) *m*/*z*: [M + H]^+^ calcd For C_35_H_35_N_2_O_6_S_3_, 675.1657; found, 675.1650. IR (cm^–1^): 3062, 2958, 2916, 1728, 1682. The ee value was determined by HPLC analysis (Daicel Chiralpak IF, hexane/isopropanol 30:70), flow rate: 0.5 mL/min, retention times: 44.2 min (major) and 86.0 min (minor).

#### (*S*)-5-Allyl-1-benzoyl-2-(benzylthio)-5-(2,2-bis(phenylsulfonyl)-ethyl)-1,5-dihydro-4*H*-imidazole-4-one (**10e**)

The title compound was prepared from 5-allyl-1-benzoyl-2-(benzylthio)-1,5-dihydro-4*H*-imidazole-4-one (35 mg, 0.1 mmol) according to the general procedure. Silica gel flash column chromatography (eluent: hexane/ethyl acetate, from 3:1 to 1:1). White foam. Yield: 56 mg, 85%. [α]_D_^20^ + 27.3 (*c* = 1, 98% ee, CH_2_Cl_2_). ^1^H NMR (300 MHz, CDCl_3_): δ 8.12–7.15 (m, 20H), 5.56 (dd, *J* = 6.0, 3.0 Hz, 1H), 5.44 (m, 1H), 5.19–5.04 (m, 2H), 4.36 (s, 2H), 3.21 (dd, *J* = 16.6, 3.1 Hz, 1H), 3.05 (dd, *J* = 10.8, 5.8 Hz, 1H), 3.02–2.95 (m, 1H), 2.57 (ddt, *J* = 13.9, 5.4, 1.4 Hz, 1H). ^13^C{^1^H} NMR (75 MHz, CDCl_3_): δ 186.1, 184.9, 168.2, 138.3, 137.1, 135.1, 134.9, 133.5, 133.3, 130.6, 130.3, 130.2, 129.5, 129.3, 129.2, 129.0, 129.0, 128.8, 128.3, 121.9, 77.7, 71.8, 39.9, 39.6, 31.3. HRMS (ESI) *m*/*z*: [M + H]^+^ calcd for C_34_H_31_N_2_O_6_S_3_, 659.1344; found, 659.1346. IR (cm^–1^): 3061, 2923, 1728, 1683. The ee value was determined by HPLC analysis (Daicel Chiralpak IC, hexane/isopropanol 30:70), flow rate: 0.5 mL/min, retention times: 34.6 min (major) and 42.5 min (minor).

#### (*S*)-1-Benzoyl-2-(benzylthio)-5-(2,2-bis(phenylsulfonyl)ethyl)-5-(2-(methylthio)ethyl)-1,5-dihydro-4*H*-imidazole-4-one (**10f**)

The title compound was prepared from 1-benzoyl-2-(benzylthio)-5-(2-(methylthio)ethyl)-1,5-dihydro-4*H*-imidazole-4-one (38 mg, 0.1 mmol) according to the general procedure. Silica gel flash column chromatography (eluent: hexane/ethyl acetate, from 3:1 to 1:1). White foam. Yield: 63 mg, 91%. [α]_D_^20^ + 19.7 (*c* = 1, 92% ee, CH_2_Cl_2_). ^1^H NMR (300 MHz, CDCl_3_): δ 8.18–8.06 (m, 2H), 8.05–7.95 (m, 2H), 7.85–7.38 (m, 12H), 7.25 (m, 4H), 5.57 (dd, *J* = 5.9, 3.0 Hz, 1H), 4.41 (s, 2H), 3.16 (dd, *J* = 16.6, 3.0 Hz, 1H), 3.00 (dd, *J* = 16.6, 5.9 Hz, 1H), 2.51 (ddd, *J* = 14.0, 9.2, 5.4 Hz, 1H), 2.30–2.14 (m, 2H), 2.07 (ddd, *J* = 8.0, 5.6, 2.4 Hz, 1H), 2.02 (s, 3H). ^13^C{^1^H} NMR (75 MHz, CDCl_3_): δ 185.7, 184.7, 168.0, 138.1, 136.9, 135.0, 134.7, 134.6, 133.3, 133.1, 130.3, 130.1, 129.4, 129.3, 129.2, 129.0, 128.90, 128.86, 128.2, 77.3, 71.4, 39.7, 34.3, 31.7, 28.4, 15.6. HRMS (ESI) *m*/*z*: [M + H]^+^ calcd for C_34_H_33_N_2_O_6_S_4_, 693.1221; found, 693.1227. IR (cm^–1^): 3060, 2928, 2849, 1727, 1681. The ee value was determined by HPLC analysis (Daicel Chiralpak IF, hexane/isopropanol 30:70), flow rate: 0.5 mL/min, retention times: 58.9 min (major) and 114.1 min (minor).

#### Methyl (*S*)-2-(1-Benzoyl-2-(benzylthio)-5-(2,2- bis(phenylsulfo-nyl)ethyl)-4-oxo-4,5-dihydro-1*H*-imidazole-5-yl)acetate (**10g**)

The title compound was prepared from methyl 2-(1-benzoyl-2-(benzylthio)-4-oxo-4,5-dihydro-1*H*-imidazole-5-yl)acetate (38 mg, 0.1 mmol) according to the general procedure. Silica gel flash column chromatography (eluent: hexane/ethyl acetate, from 3:1 to 1:1). Yellow foam. Yield: 68 mg, 98%. [α]_D_^20^ – 17.9 (*c* = 1, 96% ee, CH_2_Cl_2_). ^1^H NMR (300 MHz, CDCl_3_): δ 8.12–7.98 (m, 4H), 7.96–7.88 (m, 1H), 7.77–7.37 (m, 12H), 7.24–7.22 (m, 3H), 5.50 (dd, *J* = 4.8, 3.3 Hz, 1H), 4.53 (d, *J* = 13.3 Hz, 1H), 4.34 (d, *J* = 13.3 Hz, 1H), 3.64 (s, 3H), 3.39 (d, *J* = 17.9 Hz, 1H), 3.31 (dd, *J* = 16.6, 3.3 Hz, 1H), 3.11 (d, *J* = 17.8 Hz, 1H), 2.88 (dd, *J* = 16.6, 4.8 Hz, 1H). ^13^C{^1^H} NMR (75 MHz, CDCl_3_): δ 185.4, 184.7, 169.8, 168.2, 137.9, 136.6, 135.1, 135.0, 134.71, 134.65, 133.22, 133.16, 130.6, 130.1, 129.9, 129.4, 129.3, 129.2, 129.1, 128.9, 128.8, 128.1, 77.1, 68.3, 52.3, 39.8, 36.6, 31.6. HRMS (ESI) *m*/*z*: [M + H]^+^ calcd for C_34_H_31_N_2_O_8_S_3_, 691.1237; found, 691.1240. IR (cm^–1^): 3063, 2951, 1731, 1680. The ee value was determined by HPLC analysis (Daicel Chiralpak IC, hexane/isopropanol 30:70), flow rate: 0.5 mL/min, retention times: 47.5 min (minor) and 67.6 min (major).

### General Procedure for the Catalytic Addition of Surrogates **3** to **2**

In a 5 mL test tube, the corresponding dihydroimidazole-5-one (0.1 mmol) was dissolved in 1 mL of CH_2_Cl_2_ at room temperature. Then, the reaction was cooled down to 0 °C, and the vinyl sulfone (1.2 equiv, 0.12 mmol) and 10 mol % of **C2** (8 mg, 0.01 mmol) were added. Once the addition was completed, the mixture was stirred at 0 °C until the reaction was finished as monitored by NMR. The crude was purified directly by silica gel flash column chromatography (eluent: hexane/ethyl acetate, from 3:1 to 1:1).

#### (*S*)-1-Benzoyl-5-benzyl-2-(benzylthio)-5-((*R*)-phenyl(1,1,3,3-tetraoxido-2*H*-benzo[*d*][1,3]dithiol-2-yl)methyl)-1,5-dihydro-4*H*-imidazole-4-one (**15aa**)

The title compound was prepared from 1-benzoyl-5-benzyl-2-(benzylthio)-1,5-dihydro-4*H*-imidazole-4-one (40 mg, 0.1 mmol) and 2-benzylidene-2*H*-benzo[*d*][1,3]dithiole 1,1,3,3-tetraoxide (37 mg, 0.12 mmol, 1.2 equiv) according to the general procedure. Silica gel flash column chromatography (eluent: hexane/ethyl acetate, from 3:1 to 1:1). White foam. Yield: 56 mg, 79%. [α]_D_^20^ + 35.1 (*c* = 1, 99% ee, CH_2_Cl_2_). ^1^H NMR (300 MHz, CDCl_3_): δ 8.19–7.77 (m, 6H), 7.65–7.29 (m, 8H), 7.25–7.15 (m, 8H), 6.95–6.89 (m, 2H), 6.32 (d, *J* = 9.7 Hz, 1H), 5.21 (d, *J* = 9.8 Hz, 1H), 4.30 (d, *J* = 13.2 Hz, 1H), 4.08 (d, *J* = 13.2 Hz, 1H), 3.92 (d, *J* = 12.9 Hz, 1H), 3.77 (d, *J* = 12.9 Hz, 1H). ^13^C{^1^H} NMR (75 MHz, CDCl_3_): δ 186.7, 185.2, 167.3, 137.9, 136.5, 135.2, 135.0, 134.4, 133.8, 133.1, 132.8, 132.62, 132.55, 130.9, 130.0, 129.5, 129.2, 128.7, 128.64, 128.56, 128.4, 128.23, 128.18, 128.0, 127.6, 123.0, 122.2, 77.7, 73.9, 46.6, 41.9, 40.0. HRMS (ESI) *m*/*z*: [M + H]^+^ calcd for C_38_H_31_N_2_O_6_S_3_, 707.1344; found, 707.1339. IR (cm^–1^): 3060, 3025, 2968, 1697, 1652. The ee value was determined by HPLC analysis (Daicel Chiralpak IA, hexane/isopropanol 30:70), flow rate: 0.5 mL/min, retention times: 37.0 min (minor) and 55.5 min (major).

#### (*S*)-1-Benzoyl-2-(benzylthio)-5-ethyl-5-((*R*)-phenyl(1,1,3,3-tetraoxido-2*H*-benzo[*d*][1,3]dithiol-2-yl)methyl)-1,5-dihydro-4*H*-imidazole-4-one (**15ca**)

The title compound was prepared from 1-benzoyl-2-(benzylthio)-5-ethyl-1,5-dihydro-4*H*-imidazole-4-one (34 mg, 0.1 mmol) and 2-benzylidene-2*H*-benzo[*d*][1,3]dithiole 1,1,3,3-tetraoxide (37 mg, 0.12 mmol, 1.2 equiv) according to the general procedure. Silica gel flash column chromatography (eluent: hexane/ethyl acetate, from 3:1 to 1:1). White solid, mp: 115–120 °C. Yield: 50 mg, 78%. [α]_D_^20^ + 24.1 (*c* = 1, 96% ee, CH_2_Cl_2_). ^1^H NMR (300 MHz, CDCl_3_): δ 8.16–8.02 (m, 1H), 8.00–7.69 (m, 4H), 7.51–7.04 (m, 14H), 6.15 (d, *J* = 9.4 Hz, 1H), 4.95 (d, *J* = 9.4 Hz, 1H), 4.27 (d, *J* = 13.4 Hz, 1H), 4.11 (d, *J* = 13.4 Hz, 1H), 2.95–2.77 (m, 2H), 0.76 (t, *J* = 7.2 Hz, 3H). ^13^C{^1^H} NMR (75 MHz, CDCl_3_): δ 186.9, 184.8, 166.9, 138.0, 136.7, 135.2, 135.0, 134.6, 133.12, 133.07, 132.6, 131.3, 129.5, 129.3, 128.92, 128.86, 128.7, 128.5, 128.4, 128.1, 127.9, 123.0, 122.2, 77.8, 73.9, 46.4, 39.7, 30.0, 8.7. HRMS (ESI) *m*/*z*: [M + H]^+^ calcd for C_33_H_29_N_2_O_6_S_3_, 645.1182; found, 645.1192. IR (cm^–1^): 3083, 3022, 2850, 1724, 1681. The ee value was determined by HPLC analysis (Daicel Chiralpak IC, hexane/isopropanol 30:70), flow rate: 0.5 mL/min, retention times: 38.1 min (minor) and 55.8 min (major).

#### (*S*)-1-Benzoyl-2-(benzylthio)-5-isobutyl-5-((*R*)-phenyl-(1,1,3,3-tetraoxido-2*H*-benzo[*d*][1,3]dithiol-2-yl)methyl)-1,5-dihydro-4*H*-imidazole-4-one (**15da**)

The title compound was prepared from 1-benzoyl-2-(benzylthio)-5-isobutyl-1,5-dihydro-4*H*-imidazole-4-one (37 mg, 0.1 mmol) and 2-benzylidene-2*H*-benzo[*d*][1,3]dithiole 1,1,3,3-tetraoxide (37 mg, 0.12 mmol, 1.2 equiv) according to the general procedure. Silica gel flash column chromatography (eluent: hexane/ethyl acetate, from 3:1 to 1:1). White solid, mp 213–217 °C. Yield: 65 mg, 97%. [α]_D_^20^ + 14.3 (*c* = 1, 92% ee, CH_2_Cl_2_). ^1^H NMR (300 MHz, CDCl_3_): δ 8.19–8.06 (m, 1H), 7.97–7.73 (m, 3H), 7.52–7.21 (m, 13H), 7.13 (m, 2H), 6.13 (d, *J* = 9.0 Hz, 1H), 5.00 (d, *J* = 9.0 Hz, 1H), 4.28 (d, *J* = 13.3 Hz, 1H), 4.09 (d, *J* = 13.4 Hz, 1H), 2.85 (dd, *J* = 13.9, 4.7 Hz, 1H), 2.76 (dd, *J* = 13.9, 7.2 Hz, 1H), 1.42 (dt, *J* = 11.5, 6.7 Hz, 1H), 0.95 (d, *J* = 6.6 Hz, 3H), 0.88 (d, *J* = 6.6 Hz, 3H). ^13^C{^1^H} NMR (75 MHz, CDCl_3_): δ 187.2, 184.7, 167.0, 138.2, 136.8, 135.1, 135.0, 134.6, 133.3, 133.0, 132.9, 130.7, 129.6, 129.4, 128.78, 128.75, 128.6, 128.5, 128.3, 128.1, 123.0, 122.3, 76.3, 73.9, 47.7, 44.3, 39.8, 25.8, 24.4, 23.2. HRMS (ESI) *m*/*z*: [M + H]^+^ calcd for C_35_H_33_N_2_O_6_S_3_, 673.1501; found, 673.1492. IR (cm^–1^): 2982, 2868, 1720, 1683. The ee value was determined by HPLC analysis (Daicel Chiralpak IC, hexane/isopropanol 30:70), flow rate: 0.5 mL/min, retention times: 29.4 min (minor) and 75.1 min (major).

#### (*S*)-5-Allyl-1-benzoyl-2-(benzylthio)-5-((*R*)-phenyl(1,1,3,3-tetraoxido-2*H*-benzo[*d*][1,3]dithiol-2-yl)methyl)-1,5-dihydro-4*H*-imidazole-4-one (**15ea**)

The title compound was prepared from 5-allyl-1-benzoyl-2-(benzylthio)-1,5-dihydro-4*H*-imidazole-4-one (35 mg, 0.1 mmol) and 2-benzylidene-2*H*-benzo[*d*][1,3]dithiole 1,1,3,3-tetraoxide (37 mg, 0.12 mmol, 1.2 equiv) according to the general procedure. Silica gel flash column chromatography (eluent: hexane/ethyl acetate, from 3:1 to 1:1). White solid, mp 225–228 °C. Yield: 52.5 mg, 80%. [α]_D_^20^ + 54.9 (*c* = 1, 95% ee, CH_2_Cl_2_). ^1^H NMR (300 MHz, CDCl_3_): δ 8.21–6.99 (m, 19H), 6.18 (d, *J* = 9.7 Hz, 1H), 5.43 (m, 1H), 5.23 (m, 1H), 5.12–4.95 (m, 2H), 4.26 (d, *J* = 13.4 Hz, 1H), 4.06 (d, *J* = 13.4 Hz, 1H), 3.68–3.49 (m, 2H). ^13^C{^1^H} NMR (75 MHz, CDCl_3_): δ 186.3, 184.8, 166.9, 142.7, 137.9, 136.5, 135.3, 135.22, 135.17, 135.1, 134.6, 133.8, 132.9, 132.8, 132.7, 132.6, 130.9, 130.0, 129.5, 129.3, 128.7, 128.6, 128.3, 128.0, 122.9, 122.4, 122.2, 122.1, 121.5, 76.3, 73.7, 46.0, 40.4, 39.5. HRMS (ESI) *m*/*z*: [M + H]^+^ calcd for C_34_H_29_N_2_O_6_S_3_, 657.1188; found, 657.1179. IR (cm^–1^): 2978, 1714, 1694. The ee value was determined by HPLC analysis (Daicel Chiralpak IC, hexane/isopropanol 30:70), flow rate: 0.5 mL/min, retention times: 49.0 min (minor) and 57.9 min (major).

#### (*S*)-1-Benzoyl-2-(benzylthio)-5-(2-(methylthio)ethyl)-5-((*R*)-phenyl(1,1,3,3-tetraoxido-2*H*-benzo[*d*][1,3]dithiol-2-yl)methyl)-1,5-dihydro-4*H*-imidazole-4-one (**15fa**)

The title compound was prepared from 1-benzoyl-2 (benzylthio)-5-(2-(methylthio)ethyl)-1,5-dihydro-4*H*-imidazole-4-one (38 mg, 0.1 mmol) and 2-benzylidene-2*H*-benzo[*d*][1,3]dithiole 1,1,3,3-tetraoxide (37 mg, 0.12 mmol, 1.2 equiv) according to the general procedure. Silica gel flash column chromatography (eluent: hexane/ethyl acetate, from 3:1 to 1:1). White foam. Yield: 64 mg, 93%. [α]_D_^20^ + 30.6 (*c* = 1, 96% ee, CH_2_Cl_2_). ^1^H NMR (300 MHz, CDCl_3_): δ 8.16–8.07 (m, 1H), 8.03–7.77 (m, 4H), 7.51–7.10 (m, 14H), 6.14 (d, *J* = 9.4 Hz, 1H), 4.99 (d, *J* = 9.5 Hz, 1H), 4.28 (d, *J* = 13.3 Hz, 1H), 4.12 (d, *J* = 13.4 Hz, 1H), 3.27–3.09 (m, 2H), 2.37–2.14 (m, 2H), 2.12 (s, 3H). ^13^C{^1^H} NMR (75 MHz, CDCl_3_): δ 186.3, 184.8, 167.0, 138.1, 136.7, 135.2, 135.0, 134.5, 133.3, 133.2, 132.5, 130.6, 129.7, 129.4, 129.0, 128.9, 128.8, 128.4, 128.2, 128.1, 123.1, 122.3, 76.2, 73.9, 46.7, 39.8, 35.5, 28.6, 15.4. HRMS (ESI) *m*/*z*: [M + H]^+^ calcd for C_34_H_31_N_2_O_6_S_4_, 691.1065; found, 691.1061. IR (cm^–1^): 3060, 3029, 2915, 1723, 1682. The ee value was determined by HPLC analysis (Daicel Chiralpak IC, hexane/isopropanol 30:70), flow rate: 0.5 mL/min, retention times: 40.6 min (minor) and 74.2 min (major).

#### Methyl 2-((*S*)-1-benzoyl-2-(benzylthio)-4-oxo-5-((*R*)-phenyl(1,1,3,3-tetraoxido-2*H*-benzo[*d*][1,3]dithiol-2-yl)methyl)-4,5-dihydro-1*H*-imidazole-5-yl)acetate (**15ga**)

The title compound was prepared from methyl 2-(1-benzoyl-2-(benzylthio)-4-oxo-4,5-dihydro-1*H*-imidazole-5-yl)acetate (38 mg, 0.1 mmol) and 2-benzylidene-2*H*-benzo[*d*][1,3]dithiole 1,1,3,3-tetraoxide (37 mg, 0.12 mmol, 1.2 equiv) according to the general procedure. Silica gel flash column chromatography (eluent: hexane/ethyl acetate, from 3:1 to 1:1). White foam. Yield: 62 mg, 91%. [α]_D_^20^ – 6.5 (*c* = 1, 84% ee, CH_2_Cl_2_) (−20 °C). ^1^H NMR (300 MHz, CDCl_3_): δ 8.16–8.08 (m, 1H), 7.98–7.78 (m, 3H), 7.54–7.26 (m, 9H), 7.25–7.04 (m, 6H), 5.94 (d, *J* = 10.1 Hz, 1H), 5.11 (d, *J* = 10.1 Hz, 1H), 4.25–4.16 (m, 2H), 4.07 (d, *J* = 13.1 Hz, 1H), 3.89 (d, *J* = 16.6 Hz, 1H), 3.65 (s, 3H). ^13^C{^1^H} NMR (75 MHz, CDCl_3_): δ 185.6, 185.2, 169.7, 167.1, 138.1, 136.2, 135.4, 135.1, 134.2, 133.0, 132.91, 132.87, 129.8, 129.5, 129.3, 129.0, 128.8, 128.7, 128.6, 128.4, 128.1, 127.6, 123.0, 122.4, 73.5, 72.4, 52.3, 47.1, 40.1, 38.7. HRMS (ESI) *m*/*z*: [M + H]^+^ calcd for C_34_H_29_N_2_O_8_S_3_, 689.1086; found, 689.1092. IR (cm^–1^): 2952, 2936, 1725, 1679. The ee value was determined by HPLC analysis (Daicel Chiralpak IC, hexane/isopropanol 30:70), flow rate: 0.5 mL/min, retention times: 63.9 min (minor) and 80.6 min (major).

#### Methyl 2-(((*S*)-1-benzoyl-2-(benzylthio)-4-oxo-5-((*R*)-phenyl(1,1,3,3-tetraoxido-2*H*-benzo[*d*][1,3]dithiol-2-yl)methyl)-4,5-dihydro-1*H*-imidazole-5-yl)methyl)acrylate (**15ha**)

The title compound was prepared from a sample of **3h** containing its dialkylated analogue **3h′** (mol ratio of **3h**/**3h′** 2:1; 41 mg, 0.1 mmol) and 2-benzylidene-2*H*-benzo[*d*][1,3]dithiole 1,1,3,3-tetraoxide (37 mg, 0.12 mmol, 1.2 equiv) according to the general procedure. Silica gel flash column chromatography (eluent: hexane/ethyl acetate, from 3:1 to 1:1). White foam. Yield: 47 mg, 98%. [α]_D_^20^ + 20.1 (*c* = 1, 91% ee, CH_2_Cl_2_). ^1^H NMR (300 MHz, CDCl_3_): δ 8.17–8.08 (m, 1H), 7.95–7.78 (m, 3H), 7.64–7.19 (m, 13H), 7.15–7.06 (m, 2H), 6.35 (d, *J* = 9.7 Hz, 1H), 6.25 (d, *J* = 1.4 Hz, 1H), 5.80 (d, *J* = 1.3 Hz, 1H), 5.14 (d, *J* = 9.7 Hz, 1H), 4.13 (q, *J* = 13.1 Hz, 2H), 4.04–3.90 (m, 2H), 3.65 (s, 3H). ^13^C{^1^H} NMR (75 MHz, CDCl_3_): δ 185.6, 184.8, 167.0, 166.9, 138.2, 136.6, 135.4, 135.2, 135.0, 134.7, 134.3, 133.6, 133.4, 132.9, 132.8, 131.8, 130.8, 130.0, 129.6, 129.5, 129.4, 128.83, 128.77, 128.6, 128.5, 128.4, 128.2, 128.1, 128.0, 123.0, 122.7, 122.6, 122.3, 76.0, 74.0, 52.1, 46.3, 40.0, 37.8. HRMS (ESI) *m*/*z*: [M + H]^+^ calcd for C_36_H_31_N_2_O_8_S_3_, 715.1237; found, 715.1244. IR (cm^–1^): 1717, 1683. The ee value was determined by HPLC analysis (Daicel Chiralpak IA, hexane/isopropanol 30:70, flow rate: 0.5 mL/min, retention times: 56.8 min (minor) and 134.3 min (major).

#### (*S*)-1-Benzoyl-5-benzyl-2-(benzylthio)-5-((*R*)-(4-methoxyphenyl)(1,1,3,3-tetraoxido-2*H*-benzo[*d*][1,3]dithiol-2-yl)methyl)-1,5-dihydro-4*H*-imidazole-4-one (**15ab**)

The title compound was prepared from 1-benzoyl-5-benzyl-2-(benzylthio)-1,5-dihydro-4*H*-imidazole-4-one (40 mg, 0.1 mmol) and 2-(4-methoxybenzylidene)-2*H*-benzo[*d*][1,3]dithiole 1,1,3,3-tetraoxide (40 mg, 0.12 mmol, 1.2 equiv) according to the general procedure. Silica gel flash column chromatography (eluent: hexane/ethyl acetate, from 3:1 to 1:1). White foam. Yield: 45 mg, 61%. [α]_D_^20^ + 28.0 (*c* = 1, 97% ee, CH_2_Cl_2_). ^1^H NMR (300 MHz, CDCl_3_): δ 8.20–8.11 (m, 1H), 8.04–7.79 (m, 3H), 7.47–7.09 (m, 14H), 7.02–6.91 (m, 2H), 6.91–6.68 (m, 3H), 6.30 (d, *J* = 9.8 Hz, 1H), 5.21 (d, *J* = 9.9 Hz, 1H), 4.34 (d, *J* = 13.2 Hz, 1H), 4.08 (d, *J* = 13.2 Hz, 1H), 3.97 (d, *J* = 12.8 Hz, 1H), 3.84 (s, 1H), 3.80 (s, 3H). ^13^C{^1^H} NMR (75 MHz, CDCl_3_): δ 186.9, 185.3, 167.3, 160.5, 138.1, 136.7, 135.2, 135.0, 134.6, 134.5, 133.90, 132.88, 132.7, 130.1, 129.5, 129.3, 128.74, 128.66, 128.1, 127.6, 123.0, 122.7, 122.3, 113.8, 113.7, 78.1, 74.1, 55.4, 46.0, 41.8, 40.2. HRMS (ESI) *m*/*z*: [M + H]^+^ calcd for C_39_H_33_N_2_O_7_S_3_, 737.1444; found, 737.1441. IR (cm^–1^): 3060, 3029, 2929, 2836, 1720, 1682. The ee value was determined by HPLC analysis (Daicel Chiralpak IA, hexane/isopropanol 50:50), flow rate: 0.5 mL/min, retention times: 66.4 min (minor) and 121.7 min (major).

#### (*S*)-1-Benzoyl-5-benzyl-2-(benzylthio)-5-((*R*)-(4-chlorophenyl)(1,1,3,3-tetraoxido-2*H*-benzo[*d*][1,3]dithiol-2-yl)methyl)-1,5-dihydro-4*H*-imidazole-4-one (**15ac**)

The title compound was prepared from 1-benzoyl-5-benzyl-2-(benzylthio)-1,5-dihydro-4*H*-imidazole-4-one (40 mg, 0.1 mmol) and 2-(4-chlorobenzylidene)-2*H*-benzo[*d*][1,3]dithiole 1,1,3,3-tetraoxide (40.8 mg, 0.12 mmol, 1.2 equiv) according to the general procedure. Silica gel flash column chromatography (eluent: hexane/ethyl acetate, from 3:1 to 1:1). White foam. Yield: 59 mg, 80%. [α]_D_^20^ + 35.9 (*c* = 1, 99% ee, CH_2_Cl_2_). ^1^H NMR (300 MHz, CDCl_3_): δ 8.18–8.11 (m, 1H), 8.00–7.81 (m, 3H), 7.56–7.12 (m, 17H), 7.01–6.91 (m, 2H), 6.26 (d, *J* = 9.7 Hz, 1H), 5.22 (d, *J* = 9.7 Hz, 1H), 4.30 (d, *J* = 13.2 Hz, 1H), 4.01 (d, *J* = 13.3 Hz, 1H), 3.95 (d, *J* = 12.9 Hz, 1H), 3.79 (d, *J* = 12.9 Hz, 1H). ^13^C{^1^H} NMR (75 MHz, CDCl_3_): δ 186.2, 185.3, 167.1, 137.3, 136.0, 135.3, 135.1, 134.2, 134.1, 133.4, 132.9, 132.1, 129.8, 129.5, 129.2, 129.1, 128.6, 128.5, 128.1, 128.0, 127.9, 127.6, 122.8, 122.1, 77.4, 73.3, 46.2, 41.3, 39.9. HRMS (ESI) *m*/*z*: [M + H]^+^ calcd for C_38_H_30_ClN_2_O_6_S_3_, 741.0955, found, 741.0955. IR (cm^–1^): 3061, 3029, 2931, 1722, 1682. The ee value was determined by HPLC analysis (Daicel Chiralpak IC, hexane/isopropanol 30:70), flow rate: 0.5 mL/min, retention times: 33.4 min (minor) and 37.6 min (major).

#### (*S*)-1-Benzoyl-2-(benzylthio)-5-((*R*)-(4-chlorophenyl)(1,1,3,3-tetraoxido-2*H*-benzo[*d*][1,3]dithiol-2-yl)methyl)-5-methyl-1,5-dihydro-4*H*-imidazole-4-one (**15bc**)

The title compound was prepared from 1-benzoyl-2-(benzylthio)-5-methyl-1,5-dihydro-4*H*-imidazole-4-one (32 mg, 0.1 mmol) and 2-(4-chlorobenzylidene)-2*H*-benzo[*d*][1,3]dithiole 1,1,3,3-tetraoxide (40.8 mg, 0.12 mmol, 1.2 equiv) according to the general procedure. Silica gel flash column chromatography (eluent: hexane/ethyl acetate, from 3:1 to 1:1). White foam. Yield: 46 mg, 69%. [α]_D_^20^ + 13.4 (*c* = 1, 91% ee, CH_2_Cl_2_). ^1^H NMR (300 MHz, CDCl_3_): δ 8.17–8.07 (m, 1H), 7.98–7.79 (m, 3H), 7.56–7.45 (m, 1H), 7.37–7.10 (m, 11H), 6.86 (m, 2H), 6.15 (d, *J* = 10.0 Hz, 1H), 5.01 (d, *J* = 9.9 Hz, 1H), 4.30–4.16 (m, 2H), 2.27 (s, 3H). ^13^C{^1^H} NMR (75 MHz, CDCl_3_): δ 187.6, 184.6, 167.5, 138.4, 137.3, 136.2, 135.8, 135.7, 135.0, 134.8, 134.0, 133.1, 130.4, 129.9, 129.7, 129.6, 129.5, 129.06, 128.93, 128.8, 123.6, 122.9, 73.9, 73.6, 46.2, 40.4, 24.8. HRMS (ESI) *m*/*z*: [M + H]^+^ calcd for C_32_H_26_ClN_2_O_6_S_3_, 665.0636; found, 665.0634. IR (cm^–1^): 3065, 2946, 1724, 1665. The ee value was determined by HPLC analysis (Daicel Chiralpak IF, hexane/ethanol 30:70), flow rate: 0.5 mL/min, retention times: 32.6 min (minor) and 37.2 min (major).

#### (*S*)-5-Allyl-1-benzoyl-2-(benzylthio)-5-((*R*)-(4-chlorophenyl)(1,1,3,3-tetraoxido-2*H*-benzo[*d*][1,3]dithiol-2-yl)methyl)-1,5-dihydro-4*H*-imidazole-4-one (**15ec**)

The title compound was prepared from 5-allyl-1-benzoyl-2-(benzylthio)-1,5-dihydro-4*H*-imidazole-4-one (35 mg, 0.1 mmol) and 2-(4-chlorobenzylidene)-2*H*-benzo[*d*][1,3]dithiole 1,1,3,3-tetraoxide (40.8 mg, 0.12 mmol, 1.2 equiv) according to the general procedure. Silica gel flash column chromatography (eluent: hexane/ethyl acetate, from 3:1 to 1:1). White foam. Yield: 50 mg, 72%. [α]_D_^20^ + 16.8 (*c* = 1, 95% ee, CH_2_Cl_2_). ^1^H NMR (300 MHz, CDCl_3_): δ 8.18–8.06 (m, 1H), 7.98–7.78 (m, 3H), 7.49–7.04 (m, 14H), 6.12 (d, *J* = 9.6 Hz, 1H), 5.55–5.34 (m, 1H), 5.23 (dt, *J* = 17.1, 1.7 Hz, 1H), 5.05 (dd, *J* = 9.8, 1.9 Hz, 2H), 4.22 (d, *J* = 13.3 Hz, 1H), 4.09 (d, *J* = 13.3 Hz, 1H), 3.61 (ddt, *J* = 13.4, 5.5, 1.3 Hz, 1H), 3.50 (dd, *J* = 13.4, 9.2 Hz, 1H). ^13^C{^1^H} NMR (75 MHz, CDCl_3_): δ 186.1, 185.1, 167.0, 137.9, 136.6, 135.7, 135.3, 135.2, 134.4, 134.3, 133.2, 132.6, 130.0, 129.5, 128.9, 128.79, 128.76, 128.5, 128.4, 128.2, 123.1, 122.3, 121.7, 76.3, 73.4, 45.7, 40.0, 39.7. HRMS (ESI) *m*/*z*: [M + H]^+^ calcd for C_34_H_28_ClN_2_O_6_S_3_, 691.0793; found, 691.0788. IR (cm^–1^): 3062, 3030, 1723, 1684. The ee value was determined by HPLC analysis (Daicel Chiralpak IA, hexane/isopropanol 50:50), flow rate: 0.5 mL/min, retention times: 45.4 min (minor) and 84.5 min (major).

#### (*S*)-1-Benzoyl-5-benzyl-2-(benzylthio)-5-((*S*)-furan-2-yl(1,1, 3,3-tetraoxido-2*H*-benzo[*d*][1,3]dithiol-2-yl)methyl)-1,5-dihy dro-4*H*-imidazole-4-one (**15ad**)

The title compound was prepared from 1-benzoyl-5-benzyl-2-(benzylthio)-1,5-dihydro-4*H*-imidazole-4-one (40 mg, 0.1 mmol) and 2-(furan-2-ylmethylene)-2*H*-benzo[*d*][1,3]dithiole 1,1,3,3-tetraoxide (35.5 mg, 0.12 mmol, 1.2 equiv) according to the general procedure. Silica gel flash column chromatography (eluent: hexane/ethyl acetate, from 3:1 to 1:1). Brown foam. Yield: 50 mg, 72%. [α]_D_^20^ + 76.0 (*c* = 1, 92% ee, CH_2_Cl_2_). ^1^H NMR (300 MHz, CDCl_3_): δ 8.15 (m, 1H), 8.01–7.88 (m, 3H), 7.53–7.18 (m, 12H), 7.04–6.97 (m, 2H), 6.94–6.72 (m, 2H), 6.51 (d, *J* = 3.3 Hz, 1H), 6.38 (dd, *J* = 3.3, 1.8 Hz, 1H), 6.27 (dd, *J* = 9.6, 1.3 Hz, 1H), 5.46 (d, *J* = 9.6 Hz, 1H), 4.23 (d, *J* = 13.1 Hz, 1H), 4.11–4.01 (m, 2H), 3.84 (d, *J* = 13.1 Hz, 1H). ^13^C{^1^H} NMR (75 MHz, CDCl_3_): δ 185.9, 184.6, 167.3, 145.3, 143.7, 138.2, 137.0, 135.2, 135.1, 135.0, 134.3, 134.2, 132.9, 130.1, 129.4, 128.73, 128.67, 128.0, 127.7, 125.6, 125.3, 123.2, 122.5, 122.4, 122.1, 114.7, 113.4, 76.1, 72.9, 41.4, 40.0, 39.8. HRMS (ESI) *m*/*z*: [M + H]^+^ calcd for C_36_H_29_N_2_O_7_S_3_, 697.1131; found, 697.1138. IR (cm^–1^): 2924, 1730, 1682. The ee value was determined by HPLC analysis (Daicel Chiralpak IC, hexane/isopropanol 30:70), flow rate: 0.5 mL/min, retention times: 66.9 min (minor) and 83.4 min (major).

#### (*S*)-1-Benzoyl-5-benzyl-2-(benzylthio)-5-((*S*)-(1,1,3,3-tetraoxido-2*H*-benzo[*d*][1,3]dithiol-2-yl)(thiophen-2-yl)methyl)-1,5-dihydro-4*H*-imidazole-4-one (**15ae**)

The title compound was prepared from 1-benzoyl-5-benzyl-2-(benzylthio)-1,5-dihydro-4*H*-imidazole-4-one (40 mg, 0.1 mmol) and 2-(thiophen-2-ylmethylene)-2*H*-benzo[*d*][1,3]dithiole 1,1,3,3-tetraoxide (37.6 mg, 0.12 mmol, 1.2 equiv) according to the general procedure. Silica gel flash column chromatography (eluent: hexane/ethyl acetate, from 3:1 to 1:1). White foam. Yield: 33 mg, 46%. [α]_D_^20^ + 12.2 (*c* = 1, 95% ee, CH_2_Cl_2_). ^1^H NMR (300 MHz, CDCl_3_): δ 8.20–8.06 (m, 1H), 7.96–7.85 (m, 3H), 7.62–6.83 (m, 18H), 6.26 (d, *J* = 9.5 Hz, 1H), 5.57 (d, *J* = 9.6 Hz, 1H), 4.27 (d, *J* = 13.2 Hz, 1H), 4.10–3.93 (m, 2H), 3.79 (d, *J* = 13.0 Hz, 1H). ^13^C{^1^H} NMR (75 MHz, CDCl_3_): δ 186.4, 185.5, 167.4, 138.0, 136.4, 135.3, 135.1, 134.5, 133.9, 133.0, 132.7, 130.1, 129.4, 128.7, 128.3, 128.0, 127.7, 126.8, 123.0, 122.3, 78.2, 74.2, 41.2, 40.0. HRMS (ESI) *m*/*z*: [M + H]^+^ calcd for C_36_H_29_N_2_O_6_S_4_, 713.0903; found, 713.0905. IR (cm^–1^): 3061, 3029, 2934, 1721, 1681. The ee value was determined by HPLC analysis (Daicel Chiralpak IC, hexane/isopropanol 30:70), flow rate: 0.5 mL/min, retention times: 113.0 min (minor) and 136.3 min (major).

#### Methyl 2-((*S*)-1-Benzoyl-2-(benzylthio)-4-oxo-5-((*S*)-(1,1,3,3-tetraoxido-2*H*-benzo[*d*][1,3]dithiol-2-yl)(thiophen-2-yl)methyl)-4,5-dihydro-1*H*-imidazole-5-yl)acetate (**15ge**)

The title compound was prepared from methyl 2-(1-benzoyl-2-(benzylthio)-4-oxo-4,5-dihydro-1*H*-imidazole-5-yl)acetate (38 mg, 0.1 mmol) and 2-(thiophen-2-ylmethylene)-2*H*-benzo[*d*][1,3]dithiole 1,1,3,3-tetraoxide (37.6 mg, 0.12 mmol, 1.2 equiv) according to the general procedure. Silica gel flash column chromatography (eluent: hexane/ethyl acetate, from 3:1 to 1:1). White foam. Yield: 34.5 mg, 50%. [α]_D_^20^ – 5.1 (*c* = 1, 72% ee, CH_2_Cl_2_). ^1^H NMR (300 MHz, CDCl_3_): δ 8.20–8.04 (m, 1H), 8.00–7.77 (m, 3H), 7.55–6.74 (m, 13H), 5.93 (d, *J* = 9.8 Hz, 1H), 5.43 (d, *J* = 9.8 Hz, 1H), 4.27–4.05 (m, 3H), 3.82 (d, *J* = 16.6 Hz, 1H), 3.66 (s, 3H). ^13^C{^1^H} NMR (75 MHz, CDCl_3_): δ 185.9, 184.9, 169.7, 167.3, 138.1, 135.9, 135.5, 135.2, 134.3, 133.1, 132.9, 129.6, 129.0, 128.8, 128.6, 128.1, 126.4, 123.0, 122.5, 73.5, 73.0, 52.4, 41.3, 40.0, 37.9. HRMS (ESI) *m*/*z*: [M + H]^+^ calcd for C_32_H_27_N_2_O_8_S_4_, 695.0650; found, 695.0647. IR (cm^–1^): 3092, 2953, 1727, 1680. The ee value was determined by HPLC analysis (Daicel Chiralpak IC, hexane/isopropanol 30:70), flow rate: 0.5 mL/min, retention times: 71.4 min (minor) and 89.6 min (major).

#### (*S*)-1-Benzoyl-5-benzyl-2-(benzylthio)-5-((*S*)-pyridin-2-yl(1,1,3,3-tetraoxido-2*H*-benzo[*d*][1,3]dithiol-2-yl)methyl)-1,5-dihydro-4*H*-imidazole-4-one (**15af**)

The title compound was prepared from 1-benzoyl-5-benzyl-2-(benzylthio)-1,5-dihydro-4*H*-imidazole-4-one (40 mg, 0.1 mmol) and 2-(pyridin-2-ylmethylene)-2*H*-benzo[*d*][1,3]dithiole 1,1,3,3-tetraoxide (37 mg, 0.12 mmol, 1.2 equiv) according to the general procedure. Silica gel flash column chromatography (eluent: hexane/ethyl acetate, from 3:1 to 1:1). White foam. Yield: 48 mg, 68%. [α]_D_^20^ + 70.9 (*c* = 1, >99% ee, CH_2_Cl_2_). ^1^H NMR (300 MHz, CDCl_3_): δ 8.50 (m, 1H), 8.21–8.11 (m, 1H), 8.03–7.79 (m, 3H), 7.70–6.77 (m, 18H), 6.46 (d, *J* = 9.2 Hz, 1H), 5.36 (d, *J* = 9.2 Hz, 1H), 4.31 (d, *J* = 13.2 Hz, 1H), 4.09 (d, *J* = 13.2 Hz, 1H), 4.00 (d, *J* = 13.0 Hz, 1H), 3.80 (d, *J* = 13.0 Hz, 1H). ^13^C{^1^H} NMR (75 MHz, CDCl_3_): δ 185.5, 183.1, 167.3, 152.8, 149.4, 138.0, 137.0, 136.6, 135.2, 135.1, 134.4, 134.3, 132.9, 132.7, 130.3, 129.4, 128.7, 128.6, 127.9, 127.6, 126.8, 123.9, 123.1, 122.3, 76.3, 74.5, 48.5, 42.0, 39.8. HRMS (ESI) *m*/*z*: [M + H]^+^ calcd for C_37_H_30_N_3_O_6_S_3_, 708.1291; found, 708.1299. IR (cm^–1^): 3059, 2919, 2849, 1733, 1677. The ee value was determined by HPLC analysis (Daicel Chiralpak IC, hexane/isopropanol 30:70), flow rate: 0.5 mL/min, retention times: 86.3 min (major) and 110.8 min (minor).

#### (*S*)-5-Allyl-1-benzoyl-2-(benzylthio)-5-((*R*)-naphthalen-1-yl(1,1,3,3-tetraoxido-2*H*-benzo[*d*][1,3]dithiol-2-yl)methyl)-1,5-dihydro-4*H*-imidazole-4-one (**15eg**)

The title compound was prepared from 5-allyl-1-benzoyl-2-(benzylthio)-1,5-dihydro-4*H*-imidazole-4-one (35 mg, 0.1 mmol, 1 equiv) and 2-(naphthalen-1-ylmethylene)-2*H*-benzo[*d*][1,3]dithiole 1,1,3,3-tetraoxide (43 mg, 0.12 mmol, 1.2 equiv) according to the general procedure. Yellow foam. Yield: 45.3 mg, 0.064 mmol, 64% (81% conv, 4 d, r.t.). [α]_D_^20^ – 88.1 (*c* = 1, 99% ee, CH_2_Cl_2_). ^1^H NMR (300 MHz, CDCl_3_): δ 8.25 (m, 1H), 8.19–8.07 (m, 2H), 8.06–7.50 (m, 9H), 7.49–7.28 (m, 4H), 7.23 (m, 2H), 7.08 (m, *J* = 2.9 Hz, 3H), 6.50 (d, *J* = 9.0 Hz, 1H), 5.99 (d, *J* = 9.0 Hz, 1H), 5.53–5.34 (m, 1H), 5.25 (d, *J* = 16.6 Hz, 1H), 5.03 (dd, *J* = 10.1, 2.2 Hz, 1H), 4.19 (d, *J* = 13.5 Hz, 1H), 4.03 (d, *J* = 13.5 Hz, 1H), 3.78 (dd, *J* = 13.0, 8.9 Hz, 1H), 3.64 (dd, *J* = 13.0, 5.6 Hz, 1H). ^13^C{^1^H} NMR (75 MHz, CDCl_3_): δ 187.1, 184.8, 166.7, 140.8, 137.9, 136.2, 135.2, 135.1, 134.7, 134.4, 134.0, 132.6, 132.5, 132.3, 130.04 130.00, 129.2, 128.6, 128.5, 128.0, 127.8, 127.2, 126.7, 126.2, 125.2, 124.5, 122.9, 122.1, 121.5, 76.6, 73.9, 41.0, 39.6, 39.5. HRMS (ESI) *m*/*z*: [M + H]^+^ calcd for C_38_H_31_N_2_O_6_S_3_, 707.1339; found, 707.1342. IR (cm^–1^): 3059, 2928, 1720, 1686. The ee value was determined by HPLC analysis (Daicel Chiralpak IB, hexane/isopropanol 50:50), flow rate: 0.5 mL/min, retention times: 36.2 (minor) and 40.8 (major).

#### (*S*)-1-Benzoyl-5-benzyl-2-(benzylthio)-5-((*R*)-naphthalen-2-yl(1,1,3,3-tetraoxido-2*H*-benzo[*d*][1,3]dithiol-2-yl)methyl)-1,5-dihydro-4*H*-imidazole-4-one (**15ah**)

The title compound was prepared from 1-benzoyl-5 benzyl-2-(benzylthio)-1,5-dihydro-4*H*-imidazole-4-one (120 mg, 0.3 mmol, 1 equiv) and 2-(naphthalen-2-ylmethylene)-2*H*-benzo[*d*][1,3]dithiole 1,1,3,3-tetraoxide (129 mg, 0.36 mmol, 1.2 equiv) according to the general procedure. Yellow foam. Yield: 171 mg, 0.22 mmol, 75% (0 °C, 48 h). The obtained product exhibited two sets of signals of similar intensities in ^1^H NMR, which were attributed to rotational isomers due to severe steric constrain. Thus, obtained material was converted into the corresponding hydantoin upon TFA-promoted *N-*debenzoylation and subsequent acidic hydrolysis and characterized as it. See the Supporting Information for details about these transformations and final product characterization.

#### (*S*)-5-Benzyl-2-(benzylthio)-5-((*R*)-phenyl(1,1,3,3-tetraoxido-2*H*-benzo[*d*][1,3]dithiol-2-yl)methyl)-1-(2-phenylacetyl)-1,5-dihydro-4*H*-imidazole-4-one (**18**)

The title compound was prepared from 5-benzyl-2-(benzylthio)-1-(2-phenylacetyl)-1,5-dihydro-4*H*-imidazole-4-one (41.5 mg, 0.1 mmol) and 2-benzylidene-2*H*-benzo[*d*][1,3]dithiole 1,1,3,3-tetraoxide (37 mg, 0.12 mmol, 1.2 equiv) according to the general procedure. Reaction time for >95% conversion was 9 days. Silica gel flash column chromatography (eluent: hexane/ethyl acetate, from 3:1 to 1:1). White foam. Yield: 65.6 mg, 91%. [α]_D_^20^ + 28.1 (*c* = 1, 98% ee, CH_2_Cl_2_). ^1^H NMR (300 MHz, CDCl_3_): δ 8.15–8.03 (m, 1H), 7.96–7.74 (m, 3H), 7.50–6.99 (m, 20H), 6.20 (d, *J* = 10.0 Hz, 1H), 5.14 (d, *J* = 10.0 Hz, 1H), 4.25 (s, 2H), 4.16 (d, *J* = 13.3 Hz, 1H), 3.91 (d, *J* = 13.2 Hz, 1H), 3.53 (d, *J* = 17.0 Hz, 1H), 3.45–3.32 (m, 1H). ^13^C{^1^H} NMR (75 MHz, CDCl_3_): δ 186.5, 168.8, 137.9, 136.6, 135.2, 135.0, 134.4, 134.0, 132.2, 131.7, 130.7, 129.9, 129.6, 129.5, 129.4, 128.9, 128.8, 128.5, 128.4, 128.1, 128.0, 127.9, 127.6, 122.9, 122.2, 78.3, 73.6, 46.2, 44.3, 41.1, 39.4. HRMS (ESI) *m*/*z*: [M + H]^+^ calcd for C_39_H_33_N_2_O_6_S_3_, 721.1495; found, 721.1486. IR (cm^–1^): 3029, 2928, 1718, 1701. The ee value was determined by HPLC analysis (Daicel Chiralpak IA, hexane/isopropanol 50:50), flow rate: 0.5 mL/min, retention times: 51.8 min (minor) and 60.7 min (major).

### Catalytic Addition Reaction of Unsubstituted Hydantoin Surrogates **21** to **2**

The same procedure as above was followed using 1-benzoyl-2-(benzylthio)-1,5-dihydro-4*H*-imidazole-4-one as pronucleophile.

#### (*S*)-1-Benzoyl-2-(benzylthio)-5-((*R*)-phenyl(1,1,3,3-tetraoxido-2*H*-benzo[*d*][1,3]dithiol-2-yl)methyl)-1,5-dihydro-4*H*-imidazole-4-one (**22a**)

The title compound was prepared from 1-benzoyl-2-(benzylthio)-1,5-dihydro-4*H*-imidazole-4-one (31 mg, 0.1 mmol) and 2-benzylidene-2*H*-benzo[*d*][1,3]dithiole 1,1,3,3-tetraoxide (37 mg, 0.12 mmol, 1.2 equiv) according to the general procedure. Silica gel flash column chromatography (eluent: hexane/ethyl acetate, from 3:1 to 1:1). White solid, mp 228–230 °C. Yield: 37.6 mg, 61%. [α]_D_^20^ + 6.7 (*c* = 1, 95% ee, CH_2_Cl_2_) (reaction at −25 °C). ^1^H NMR (300 MHz, CDCl_3_): δ 8.07–8.01 (m, 1H), 7.92–7.81 (m, 3H), 7.68–7.16 (m, 15H), 6.18 (d, *J* = 11.4 Hz, 1H), 5.44 (d, *J* = 5.0 Hz, 1H), 4.26 (q, *J* = 13.4 Hz, 2H), 4.10 (dd, *J* = 11.5, 4.9 Hz, 1H). ^13^C{^1^H} NMR (75 MHz, CD_2_Cl_2_): δ 187.0, 183.4, 167.3, 138.6, 137.2, 136.1, 135.9, 135.7, 133.9, 132.9, 130.4, 129.9, 129.7, 129.2, 128.9, 128.4, 123.0, 71.8, 64.3, 41.3, 38.8. HRMS (ESI) *m*/*z*: [M + H]^+^ calcd for C_31_H_25_N_2_O_6_S_3_, 617.0875; found, 617.0878. IR (cm^–1^): 3035, 2968, 2855, 1725, 1668. The ee value was determined by HPLC analysis (Daicel Chiralpak IF, hexane/ethanol 30:70), flow rate: 0.5 mL/min, retention times: 46.4 min (minor) and 78.0 min (major).

#### (*S*)-1-Benzoyl-2-(benzylthio)-5-((*R*)-(4-chlorophenyl) (1,1,3, 3-tetraoxido-2*H*-benzo[*d*][1,3]dithiol-2-yl)methyl)-1,5-dihydro-4*H*-imidazole-4-one (**22b**)

The title compound was prepared from 1-benzoyl-2-(benzylthio)-1,5-dihydro-4*H*-imidazole-4-one (31 mg, 0.1 mmol) and 2-(4-chlorobenzylidene)-2*H*-benzo[*d*][1,3]dithiole 1,1,3,3-tetraoxide (40.8 mg, 0.12 mmol, 1.2 equiv) according to the general procedure. Silica gel flash column chromatography (eluent: hexane/ethyl acetate, from 3:1 to 1:1). White solid, mp 237–240 °C. Yield: 48 mg, 74%. [α]_D_^20^ + 50.29 (*c* = 1, 88% ee, CH_2_Cl_2_). ^1^H NMR (300 MHz, CD_2_Cl_2_): δ 8.10–8.02 (m, 1H), 8.01–7.78 (m, 4H), 7.71–7.50 (m, 6H), 7.41–7.16 (m, 7H), 6.10 (d, *J* = 11.5 Hz, 1H), 5.38 (d, *J* = 4.9 Hz, 1H), 4.26 (d, *J* = 2.7 Hz, 2H), 4.07 (dd, *J* = 11.5, 4.9 Hz, 1H). ^13^C{^1^H} NMR (126 MHz, CD_2_Cl_2_): δ 187.2, 183.2, 167.2, 138.5, 137.2, 136.5, 136.2, 136.0, 135.5, 134.0, 132.7, 130.0, 129.7, 129.3, 128.9, 128.5, 128.0, 123.1, 71.6, 64.1, 40.8, 38.9. HRMS (ESI) *m*/*z*: [M + H]^+^ calcd for C_31_H_24_ClN_2_O_6_S_3_, 651.0480; found, 651.0488. IR (cm^–1^): 3059, 2945, 1723, 1672. The ee value was determined by HPLC analysis (Daicel Chiralpak IF, hexane/ethanol 30:70), flow rate: 0.5 mL/min, retention times: 40.4 min (minor) and 76.3 min (major).

### Elaboration of Adducts

#### Hydrolysis of **10a** to Hydantoin **23**

An aqueous solution of HCl 6 M (2.76 mL) was added dropwise to a solution of **10a** (1.07 g, 1.51 mmol) in 1,4-dioxane (15 mL) at 0 °C. Once the addition was complete, the reaction was stirred at 65 °C in an oil bath for 3 h. Then, an additional 2.76 mL of 6 M HCl was added dropwise, and the mixture was stirred at 65 °C for an additional 3 h. Afterward, the reaction was cooled to 0 °C, and saturated NaHCO_3_ was added until basic pH was obtained. The aqueous layer was extracted with dichloromethane twice, and the combined organic layers were dried over MgSO_4_ and the solvent evaporated under reduced pressure. The crude product was purified by silica gel flash column chromatography (hexane/EtOAc, 3:1 to 1:1) to obtain (*S*)-1-benzoyl-5-benzyl-5-(2,2-bis(phenylsulfonyl)ethyl) imidazolidine-2,4-dione (**23**) as a white solid. mp: 154–158 °C. Yield: 0.66 g, 73%. [α]_D_^20^ – 35.0 (*c* = 1, CH_2_Cl_2_). ^1^H NMR (300 MHz, CDCl_3_) 8.10–8.01 (m, 3H), 7.99–7.19 (m, 16H), 7.10–6.99 (m, 2H), 5.45 (dd, *J* = 5.3, 4.2 Hz, 1H), 3.64–3.42 (m, 2H), 3.23 (d, *J* = 13.9 Hz, 1H), 3.15 (dd, *J* = 16.6, 5.3 Hz, 1H). ^13^C{^1^H} NMR (75 MHz, CDCl_3_): δ 173.0, 169.6, 151.6, 137.8, 136.3, 135.1, 134.7, 134.1, 133.2, 132.0, 130.5, 129.79, 129.76, 129.3, 129.2, 128.9, 128.3, 128.0, 127.8, 78.2, 69.9, 39.2, 31.8. HRMS (ESI) *m*/*z*: [M + H]^+^ calcd For C_31_H_27_N_2_O_7_S_2_, 603.1254; found, 603.1252. IR (cm^–1^): 3270, 3063, 2930, 1799, 1734, 1681.

#### Hydrolysis of Adducts **15aa**/**15ab** to Hydantoins **25a**/**25b**

The same procedure as above was followed, but in this case, the mixture was stirred at 80 °C in an oil bath for 6 h. Compound **25a** was not isolated, and the crude material was used in the next transformation into **26** (vide infra).

##### (*S*)-1-Benzoyl-5-benzyl-5-((*R*)-(4-chlorophenyl) (1,1,3,3-tetraoxido-2*H*-benzo[*d*][1,3]dithiol-2-yl)methyl)imidazolidine-2,4-dione (**25b**)

The title compound was prepared from (*S*)-1-benzoyl-5-benzyl-2-(benzylthio)-5-((*R*)-(4-chlorophenyl) (1,1,3,3-tetraoxido-2*H*-benzo[*d*][1,3]dithiol-2-yl)methyl)-1,5-dihydro-4*H*-imidazole-4-one (150 mg, 0.20 mmol) according to the general procedure. Silica gel flash column chromatography (eluent: hexane/ethyl acetate, from 3:1 to 1:1). White solid, mp 213–216 °C. Yield: 99 mg, 78%. [α]_D_^20^ + 13.3 (*c* = 1, CH_2_Cl_2_). ^1^H NMR (300 MHz, CD_2_Cl_2_): δ 8.19–8.11 (m, 1H), 8.02–7.84 (m, 3H), 7.49–7.16 (m, 13H), 6.84–6.72 (m, 2H), 6.09 (d, *J* = 9.8 Hz, 1H), 5.22 (d, *J* = 9.8 Hz, 1H), 4.23 (d, *J* = 13.4 Hz, 1H), 4.07 (d, *J* = 13.5 Hz, 1H). ^13^C{^1^H} NMR (75 MHz, CD_2_Cl_2_): δ 172.7, 169.2, 150.6, 137.7, 136.4, 135.9, 135.8, 135.7, 134.8, 133.9, 132.1, 130.1, 129.8, 129.5, 129.3, 129.2, 128.9, 128.3, 128.0, 127.7, 123.2, 122.5, 74.5, 45.7, 40.3, 29.9. HRMS (ESI) *m*/*z*: [M + H]^+^ calcd for C_31_H_24_ClN_2_O_7_S_2_, 635.0708; found, 635.0701. IR (cm^–1^): 3086, 2923, 1798, 1736, 1680.

#### Synthesis of **24** (Monodesulfonylation of **23**)

Compound **23** (805 mg, 1.36 mmol, 1 equiv) was dissolved in MeOH (40 mL) and Mg turnings (667 mg, 27.2 mmol, 20 equiv), and TMSCl (0.35 mL, 2.72 mmol, 2 equiv) and 1,2-dibromomethane (0.48 mL, 5.44 mmol, 4 equiv) were added successively. The reaction mixture was then stirred for 9 days at room temperature. The solid formed was filtered through celite and washed with dichloromethane. The obtained filtrate was concentrated under reduced pressure and partitioned between water and dichloromethane. The aqueous layer was extracted three times with dichloromethane, and the combined organic layer was dried over MgSO_4_. The solvent was evaporated, and the crude colorless oil was purified by silica gel flash column chromatography (hexane/AcOEt, 3:1) to obtain (*S*)-5-benzyl-5-(2-(phenylsulfonyl)ethyl)imidazolidine-2,4-dione (**24**) as a white solid. Yield: 249 mg, 51%. mp: 189–194 °C. [α]_D_^20^ + 15.2. 0 (*c* = 1, CH_2_Cl_2_). ^1^H NMR (300 MHz, CD_2_Cl_2_): δ 8.24 (s, 1H), 7.92–7.55 (m, 5H), 7.31–7.24 (m, 3H), 7.13 (m, 2H), 6.14 (bs, 1H), 3.29–3.10 (m, 2H), 3.08 (d, *J* = 13.7 Hz, 1H), 2.90 (d, *J* = 13.7 Hz, 1H), 2.22 (m, 2H). ^13^C{^1^H} NMR (75 MHz, CD_2_Cl_2_): δ 175.3, 156.3, 139.1, 134.7, 134.0, 130.7, 130.1, 129.1, 128.6, 128.2, 66.7, 51.4, 43.3, 29.7. HRMS (ESI) *m*/*z*: [M + H]^+^ calcd for C_18_H_19_N_2_O_4_S, 359.1060; found, 359.1066. IR (cm^–1^): 3031, 2923, 1748, 1715.

#### Synthesis of Hydantoin **26** (Double Desulfonylation of **25a**)

Compound **15aa** (352 mg, 0.585 mmol) was submitted to the same conditions used for the hydrolysis of **15ab**, and the crude material **25a** was then submitted directly to desulfonylation under the above conditions at room temperature for 6 days. After the usual work-up and aftermath purification of the crude material by flash column chromatography (hexane/AcOEt, 3:1), pure compound **26** was obtained. (*S*)-5-Benzyl-5-((*R*)-1-phenylethyl)imidazolidine-2,4-dione (**26**). White solid, mp: 244–249 °C. Yield: 115 mg, 67%. [α]_D_^20^ – 55.45 (*c* = 1, 96% ee, CH_2_Cl_2_). ^1^H NMR (300 MHz, CDCl_3_): δ 7.43–7.18 (m, 8H), 7.07 (s, 1H), 7.04–6.94 (m, 2H), 5.20 (bs, 1H), 3.32 (q, *J* = 7.0 Hz, 1H), 3.12 (d, *J* = 13.7 Hz, 1H), 2.46 (d, *J* = 13.7 Hz, 1H), 1.37 (d, *J* = 7.0 Hz, 3H). ^13^C{^1^H} NMR (126 MHz, CDCl_3_): δ 175.3, 155.5, 140.4, 134.0, 130.6, 130.2, 129.1, 128.7, 128.64, 128.62, 128.0, 127.6, 71.3, 45.6, 42.7, 15.7. HRMS (ESI) *m*/*z*: [M + H]^+^ calcd for C_18_H_19_N_2_O_2_, 295.1441; found, 295.1448. IR (cm^–1^): 3061, 3029, 2969, 2926, 1761, 1704. The ee value was determined by HPLC analysis (Daicel Chiralpak IC, hexane/isopropanol 90:10), flow rate: 0.5 mL/min, retention times: 11.4 min (minor) and 16.6 min (major).

#### Synthesis of **27**

A solution of compound **15aa** (0.55 g, 0.78 mmol) in TFA (7.8 mL) was stirred at 40 °C for 48 h. Afterward, saturated NaHCO_3_ was added to the reaction mixture until the pH ≥ 7 and extracted with dichloromethane, and the organic solvent was evaporated under reduced pressure to obtain compound **27**, which was used in the next step with no further purification. White foam. Yield: 0.465 g, 0.77 mmol, 99%. [α]_D_^20^ – 146.1 (*c* = 1, CH_2_Cl_2_). ^1^H NMR (300 MHz, CDCl_3_): δ 7.83 (m, 5H), 7.66–7.36 (m, 6H), 7.22 (s, 1H), 7.17–7.03 (m, 5H), 6.96–6.85 (m, 2H), 6.81–6.70 (m, 1H), 4.56–4.44 (m, 2H), 4.33 (d, *J* = 10.3 Hz, 1H), 4.25 (d, *J* = 14.2 Hz, 1H), 2.64 (d, *J* = 12.7 Hz, 1H), 2.52 (d, *J* = 12.7 Hz, 1H). ^13^C{^1^H} NMR (75 MHz, CDCl_3_): δ 182.8, 161.0, 143.1, 138.4, 137.2, 136.8, 135.8, 135.6, 135.3, 135.1, 134.4, 132.9, 132.3, 130.7, 130.3, 129.6, 129.4, 129.3, 129.0, 128.6, 128.5, 128.0, 127.3, 127.2, 122.7, 122.5, 77.7, 76.7, 46.0, 43.8, 34.5. HRMS (ESI) *m*/*z*: [M + H]^+^ calcd for C_31_H_27_N_2_O_5_S_3_, 603.1077; found, 603.1088. IR (cm^–1^): 3341, 3028, 2913, 1743, 1562.

#### *N*-Alkylation of **27**

To a solution of **27** in CH_2_Cl_2_, 1.2 equiv of the corresponding halide compound was added, and the reaction mixture was cooled to 0 °C. Afterward, 1 equiv of K_2_CO_3_ and 0.1 equiv of DBU were added, and the mixture was stirred at room temperature until the reaction was over as monitored by ^1^H NMR (reaction times in the range of 1–2 days). Once finished, the reaction mixture was cooled to 0 °C, and HCl 0.1 M was added until neutral pH was obtained. The phases were separated, and the aqueous one was extracted three times with CH_2_Cl_2_. The combined organic layers were dried over MgSO_4_ and concentrated under reduced pressure. The crude product was purified by silica gel flash column chromatography (hexane/EtOAc, 3:1 to 1:1), affording the desired pure product.

##### (*S*)-5-Benzyl-2-(benzylthio)-1-methyl-5-((*R*)-phenyl(1,1,3,3-tetraoxido-2*H*-benzo[*d*][1,3]dithiol-2-yl)methyl)-1,5-dihydro-4*H*-imidazole-4-one (**28**)

The title compound was prepared from **27** (0.181 g, 0.298 mmol, 1 equiv), methyl iodide (23.5 μL, 0.36 mmol, 1.2 equiv), K_2_CO_3_ (43 mg, 0.298 mmol, 1 equiv), and DBU (4.5 μL, 0.03 mmol, 0.1 equiv) in CH_2_Cl_2_ (5 mL) according to the general procedure (reaction time 1 day). Silica gel flash column chromatography (eluent: hexane/ethyl acetate, from 3:1 to 1:1). White foam. Yield: 0.169 g, 0.274 mmol, 92%. ^1^H NMR (300 MHz, CD_2_Cl_2_): δ 8.05 (s, 1H), 7.93–7.75 (m, 4H), 7.64–7.38 (m, 6H), 7.19–7.00 (m, 5H), 6.89–6.71 (m, 3H), 4.54 (d, *J* = 14.2 Hz, 1H), 4.42 (d, *J* = 10.3 Hz, 1H), 4.30 (d, *J* = 14.2 Hz, 1H), 4.23 (d, *J* = 10.3 Hz, 1H), 2.55 (d, *J* = 12.5 Hz, 1H), 2.48 (s, 3H), 2.40 (d, *J* = 12.5 Hz, 1H). ^13^C{^1^H} NMR (75 MHz, CD_2_Cl_2_): δ 181.0, 164.2, 138.8, 137.4, 137.2, 135.6, 135.4, 134.8, 132.7, 130.5, 129.5, 129.4, 128.7, 128.6, 127.8, 127.5, 127.4, 122.8, 122.7, 77.4, 77.1, 45.8, 44.1, 34.6, 26.2. HRMS (ESI) *m*/*z*: [M + H]^+^ calcd for C_32_H_29_N_2_O_5_S_3_, 617.1233; found, 617.1243. IR (cm^–1^): 3060, 3028, 2919, 1731.

##### (*S*)-1,5-Dibenzyl-2-(benzylthio)-5-((*R*)-phenyl(1,1,3,3-tetraoxido-2*H*-benzo[*d*][1,3]dithiol-2-yl)methyl)-1,5-dihydro-4*H*-imidazole-4-one (**29**)

The title compound was prepared from **27** (0.5 g, 0.83 mmol, 1 equiv), benzyl bromide (1.2 mL, 1 mmol, 1.2 equiv), K_2_CO_3_ (0.115 g, 0.83 mmol, 1 equiv), and DBU (12.6 μL, 0.083 mmol, 0.1 equiv) in CH_2_Cl_2_ (11 mL) according to the general procedure (reaction time 16 h). Silica gel flash column chromatography (eluent: hexane/ethyl acetate, from 3:1 to 1:1). White foam. Yield: 0.39 g, 67%. [α]_D_^20^ – 155.6 (*c* = 1, 98% ee, CH_2_Cl_2_). ^1^H NMR (300 MHz, CDCl_3_): δ 8.13–7.74 (m, 5H), 7.61–7.34 (m, 6H), 7.23–6.89 (m, 10H), 6.68–6.60 (m, 1H), 6.38–6.30 (m, 2H), 4.81 (d, *J* = 16.3 Hz, 1H), 4.58 (d, *J* = 14.5 Hz, 1H), 4.34 (d, *J* = 10.3 Hz, 1H), 4.21 (d, *J* = 10.3 Hz, 1H), 4.17 (d, *J* = 1.3 Hz, 1H), 4.11 (s, 1H), 2.68 (d, *J* = 12.8 Hz, 1H), 2.56 (d, *J* = 12.8 Hz, 1H). ^13^C{^1^H} NMR (75 MHz, CDCl_3_): δ 181.1, 164.0, 138.2, 136.7, 136.5, 134.93, 134.88, 134.7, 134.3, 131.9, 130.4, 129.0, 128.7, 128.3, 128.11, 128.08, 128.0, 127.11, 127.07, 126.9, 126.8, 122.2, 76.6, 76.4, 46.4, 44.7, 43.4, 34.3. HRMS (ESI) *m*/*z*: [M + H]^+^ calcd for C_38_H_33_N_2_O_5_S_3_, 693.1546; found, 693.1543. IR (cm^–1^): 3028, 2917, 1734, 1557.

##### (*S*)-1-Allyl-5-benzyl-2-(benzylthio)-5-((*R*)-phenyl(1,1,3,3-tetraoxido-2*H*-benzo[*d*][1,3]dithiol-2-yl)methyl)-1,5-dihydro-4*H*-imidazole-4-one (**30**)

The title compound was prepared from **27** (0.141 g, 0.23 mmol), 3-bromoprop-1-ene (25 μL, 0.28 mmol, 1.2 equiv), K_2_CO_3_ (34 mg, 0.23 mmol, 1 equiv), and DBU (3.45 μL, 0.02 mmol, 0.1 equiv) in CH_2_Cl_2_ (5 mL) according to the general procedure (reaction time 16 h). Silica gel flash column chromatography (eluent: hexane/ethyl acetate, from 3:1 to 1:1). White solid, mp: 214–217 °C. Yield: 0.130 g, 88%. ^1^H NMR (300 MHz, CDCl_3_): δ 8.41–7.37 (m, 11H), 7.20–6.99 (m, 5H), 6.93–6.84 (m, 2H), 6.76–6.66 (m, 1H), 4.87–4.73 (m, 2H), 4.67–4.52 (m, 2H), 4.32 (d, *J* = 0.9 Hz, 2H), 4.26 (d, *J* = 14.3 Hz, 1H), 4.06–3.95 (m, 1H), 3.61–3.49 (m, 1H), 2.62 (d, *J* = 12.6 Hz, 1H), 2.48 (d, *J* = 12.6 Hz, 1H). ^13^C{^1^H} NMR (75 MHz, CDCl_3_): δ 180.4, 163.8, 138.4, 136.81, 136.78, 134.9, 134.7, 134.3, 132.0, 131.3, 130.4, 129.0, 129.0, 128.3, 128.2, 127.8, 126.98, 126.95, 122.3, 117.9, 76.7, 76.5, 45.7, 43.6, 42.9, 34.4. HRMS (ESI) *m*/*z*: [M + H]^+^ calcd for C_34_H_31_N_2_O_5_S_3_, 643.1390; found, 643.1383. IR (cm^–1^): 3082, 3028, 2906, 1723.

##### (*S*)-5-Benzyl-2-(benzylthio)-1-(2-chloroethyl)-5-((*R*)-phenyl(1,1,3,3-tetraoxido-2*H*-benzo[*d*][1,3]dithiol-2-yl)methyl)-1,5-dihydro-4*H*-imidazole-4-one (**31**)

(*S*)-5-Benzyl-2-(benzylthio)-5-((*R*)-phenyl(1,1,3,3-tetraoxido-2*H*-benzo[*d*-1,3]dithiol-2-yl)methyl)-1,5-dihydro-4*H*-imidazole-4-one **27** (121 mg, 0.2 mmol, 1 equiv), 1-bromo-2-chloroethane (20 μL, 0.24 mmol, 1.2 equiv), K_2_CO_3_ (29 mg, 0.2 mmol, 1 equiv), and DBU (3 μL, 0.02 mmol, 0.1 equiv) in DCE (4 mL) were mixed according to the general procedure (reaction stirred at 60 °C for 2 days). Upon the usual work-up, compound **31** was obtained with traces of an unknown side product that could not be eliminated after chromatography. This material was employed without further purification in the next hydrolytic step.

#### Hydrolysis of Compounds **27–30** to Hydantoins **32a–d**

The same procedure as for the hydrolysis of adducts **15** was followed.

##### (*S*)-5-Benzyl-5-(phenyl(1,1,3,3-tetraoxido-2*H*-benzo[*d*][1,3]dithiol-2-yl)methyl)imidazolidine-2,4-dione (**32a**)

The title compound was prepared from 5-benzyl-2-(benzylthio)-5-(phenyl(1,1,3,3-tetraoxido-2*H*-benzo[*d*][1,3]dithiol-2-yl)methyl)-1,5-dihydro-4*H*-imidazole-4-one (0.198 g, 0.33 mmol, 1 equiv) and HCl (6 M) (1.3 mL, 7.59 mmol, 23 equiv) in 1,4-dioxane (12 mL) according to the general procedure. Silica gel flash column chromatography (eluent: hexane/ethyl acetate, from 3:1 to 1:1). White solid, mp: 249–254 °C. Yield: 0.103 g, 63%. [α]_D_^20^ – 50.6 (*c* = 1, CH_2_Cl_2_). ^1^H NMR (300 MHz, CD_2_Cl_2_): δ 8.08–7.80 (m, 4H), 7.48 (m, 5H), 7.37–7.21 (m, 5H), 7.14–7.04 (m, 2H), 5.42 (d, *J* = 10.0 Hz, 1H), 4.40 (d, *J* = 10.0 Hz, 1H), 3.28 (d, *J* = 13.6 Hz, 1H), 2.64 (d, *J* = 13.6 Hz, 1H). ^13^C{^1^H} NMR (126 MHz, CD_2_Cl_2_): δ 182.3, 174.0, 138.5, 136.8, 136.2, 136.0, 133.1, 131.3, 130.9, 130.3, 129.5, 129.2, 128.5, 123.12, 123.09, 76.0, 73.3, 47.9, 43.4. HRMS (ESI) *m*/*z*: [M + H]^+^ calcd for C_24_H_21_N_2_O_6_S_2_, 497.0836; found, 497.0824. IR (cm^–1^): 3063, 3030, 2948, 1732, 1698.

##### (*R*)-5-Benzyl-1-methyl-5-(phenyl(1,1,3,3-tetraoxido-2*H*-benzo [*d*][1,3]dithiol-2-yl)methyl)imidazolidine-2,4-dione (**32b**)

The title compound was prepared from **28** (0.215 g, 0.35 mmol, 1 equiv) and 6 M HCl (1.3 mL, 8.05 mmol, 23 equiv) in 1,4-dioxane (12 mL) according to the general procedure. Silica gel flash column chromatography (eluent: hexane/ethyl acetate, from 3:1 to 1:1). White solid, mp 269–272 °C. Yield: 0.118 g, 66%. [α]_D_^20^ – 57.3 (*c* = 1, CH_2_Cl_2_). ^1^H NMR (300 MHz, CD_2_Cl_2_): δ 8.03–7.77 (m, 3H), 7.49 (m, 6H), 7.28–7.13 (m, 3H), 7.06–6.94 (m, 2H), 5.33 (d, *J* = 10.5 Hz, 2H), 4.45 (d, *J* = 10.1 Hz, 1H), 3.11 (d, *J* = 13.5 Hz, 1H), 2.79 (s, 3H), 2.46 (d, *J* = 13.5 Hz, 1H). ^13^C{^1^H} NMR (126 MHz, CD_2_Cl_2_): δ 185.2, 174.0, 138.4, 136.9, 136.0, 135.9, 133.0, 131.6, 130.6, 130.2, 129.6, 128.9, 128.4, 123.01, 122.99, 76.3, 71.1, 47.3, 43.9, 27.7. HRMS (ESI) *m*/*z*: [M + H]^+^ calcd for C_25_H_23_N_2_O_6_S_2_, 511.0992; found, 511.0992. IR (cm^–1^): 3063, 2930, 1731, 1684.

##### (*S*)-1,5-Dibenzyl-5-((*R*)-phenyl(1,1,3,3-tetraoxido-2*H*-benzo[*d*][1,3]dithiol-2-yl)methyl)imidazolidine-2,4-dione (**32c**)

The title compound was prepared from **29** (0.25 g, 0.36 mmol) and 6 M HCl (1.65 mL, 8.28 mmol, 23 equiv) in 1,4-dioxane (4 mL) according to the general procedure. Silica gel flash column chromatography (eluent: hexane/ethyl acetate, from 3:1 to 1:1). White solid, mp 227–231 °C. Yield: 0.12 g, 0.2 mmol, 55%. [α]_D_^20^ – 55.6 (*c* = 1, 98% ee, CH_2_Cl_2_). ^1^H NMR (300 MHz, CDCl_3_): δ 8.06–7.72 (m, 4H), 7.40 (s, 5H), 7.25–6.83 (m, 11H), 5.35 (d, *J* = 10.1 Hz, 1H), 4.79–4.64 (m, 2H), 4.43 (d, *J* = 10.0 Hz, 1H), 3.31 (d, *J* = 13.7 Hz, 1H), 2.60 (d, *J* = 13.7 Hz, 1H). ^13^C{^1^H} NMR (75 MHz, CDCl_3_): δ 184.1, 173.7, 137.9, 136.3, 135.3, 135.2, 134.7, 131.9, 130.8, 130.2, 129.8, 129.1, 128.6, 128.4, 127.9, 127.5, 122.7, 122.5, 75.6, 70.0, 47.8, 45.4, 43.0. HRMS (ESI) *m*/*z*: [M + H]^+^ calcd for C_31_H_27_N_2_O_6_S_2_, 587.1305; found, 587.1298. IR (cm^–1^): 3319, 3063, 3030, 2929, 1733, 1480. The ee value was determined by HPLC analysis (Daicel Chiralpak IA, hexane/isopropanol 30:70), flow rate: 0.5 mL/min, retention times: 60.6 min (major) and 77.2 min (minor).

##### (*R*)-Allyl-5-benzyl-5-(phenyl(1,1,3,3-tetraoxido-2*H*-benzo[*d*][1,3]dithiol-2-yl)methyl)imidazolidine-2,4-dione (**32d**)

The title compound was prepared from **30** (67 mg, 0.1 mmol, 1 equiv) and 6 M HCl (0.5 mL, 0.23 mmol, 23 equiv) in 1,4-dioxane (4 mL) according to the general procedure. Silica gel flash column chromatography (eluent: hexane/ethyl acetate, from 3:1 to 1:1). White solid, mp: 261–266 °C. Yield: 39 mg, 72%. [α]_D_^20^ – 44.5 (*c* = 1, CH_2_Cl_2_). ^1^H NMR (500 MHz, CD_2_Cl_2_): δ 8.03–7.98 (m, 1H), 7.92–7.79 (m, 3H), 7.48 (m, 6H), 7.26–7.17 (m, 3H), 7.07–6.99 (m, 2H), 5.36 (d, *J* = 10.1 Hz, 1H), 5.07 (m, 1H), 4.90 (dd, *J* = 10.2, 1.4 Hz, 1H), 4.81 (dd, *J* = 17.1, 1.5 Hz, 1H), 4.41 (d, *J* = 10.1 Hz, 1H), 4.09 (ddt, *J* = 15.1, 5.7, 1.5 Hz, 1H), 4.01 (ddt, *J* = 15.1, 6.4, 1.4 Hz, 1H), 3.20 (d, *J* = 13.6 Hz, 1H), 2.54 (d, *J* = 13.7 Hz, 1H). ^13^C{^1^H} NMR (126 MHz, CD_2_Cl_2_): δ 184.3, 173.5, 138.2, 136.7, 136.0, 135.9, 132.9, 131.4, 130.85, 130.83, 130.1, 129.4, 129.0, 128.3, 123.0, 122.9, 118.6, 76.0, 71.0, 47.6, 44.3, 43.6. HRMS (ESI) *m*/*z*: [M + H]^+^ calcd for C_27_H_25_N_2_O_6_S_2_, 537.1149; found, 537.1156. IR (cm^–1^): 3030, 2927, 1732, 1716.

#### Synthesis of **33**

Aqueous HCl (6 M, 0.36 mL) was added dropwise to a solution of crude material **31** obtained in the previous step in 1,4-dioxane (6 mL) at 0 °C. Once the addition was complete, the reaction was stirred for 6 h at 80 °C. Then, the second portion of 6 M HCl (0.40 mL) was added dropwise, and the mixture was stirred at 80 °C for an additional 9 h. Afterward, the reaction was cooled to 0 °C, and saturated NaHCO_3_ was added until basic pH was obtained. The aqueous layer was extracted with dichloromethane twice, and the combined organic layers were dried over MgSO_4_ and the solvent evaporated under reduced pressure. The crude product was purified by silica gel flash column chromatography (eluent: hexane/ethyl acetate, from 3:1 to 1:1) to obtain product **33**. White solid, mp 253–256 °C. Yield from **27**: 53 mg, 49%. [α]_D_^20^ – 54.5 (*c* = 1, CH_2_Cl_2_). ^1^H NMR (300 MHz, CD_2_Cl_2_): δ 8.15–7.74 (m, 5H), 7.60–7.38 (m, 4H), 7.18 (m, 3H), 7.08–6.95 (m, 2H), 5.20 (d, *J* = 10.4 Hz, 1H), 4.32 (d, *J* = 10.4 Hz, 1H), 3.55 (m, 1H), 3.43 (m, 1H), 3.07 (m, 1H), 2.91 (m, 1H), 2.61 (d, *J* = 12.7 Hz, 1H), 2.43 (d, *J* = 12.7 Hz, 1H). ^13^C{^1^H} NMR (126 MHz, CD_2_Cl_2_): δ 177.1, 170.1, 138.7, 137.1, 135.8, 135.6, 134.9, 132.6, 130.8, 129.5, 127.9, 127.6, 122.9, 122.6, 86.3, 77.6, 46.1, 44.2, 40.1, 34.3. HRMS (ESI) *m*/*z*: [M + H]^+^ calcd for C_26_H_23_N_2_O_5_S_3_, 539.0764; found, 539.0770. IR (cm^–1^): 3060, 3035, 2906, 2850, 1725, 1596.

## Data Availability

Data availability statement: The data underlying this study are available in the published article and its online Supporting Information.

## Supporting Information Available

Crystallographic data for **25b**, experimental and computational details, and copies of NMR spectra and HPLC chromatograms (PDF)

## Dedication

Dedicated to Prof. Joan Bosch on occasion of his retirement.
