# Supplementary material for: Rigidified Bis(sulfonyl)ethylenes as Effective Michael Acceptors for Asymmetric Catalysis: Application to the Enantioselective Synthesis of Quaternary Hydantoins
Source: J Org Chem. 2023 Jan 11;88(2):972–87. doi: 10.1021/acs.joc.2c02403 (PMC10013931; doi:10.1021/acs.joc.2c02403)
Supplement: Supplementary file 1 — jo2c02403_si_001.pdf [file jo2c02403_si_001.pdf]

SUPPORTING INFORMATION

Rigidified Bis(sulfonyl)ethylenes as Effective Michael Acceptors for Asymmetric Catalysis. Application to the Enantioselective Synthesis of Quaternary Hydantoins.

Leire Villaescusa, Iker Hernández, Laura Azcune, Ainhoa Rudi, José M. Mercero, Aitor Landa,* Mikel Oiarbide,* and Claudio Palomo*

Table of Contents

1. Synthesis of the starting materials. S2

1.1. Synthesis of β-substituted ethylene bis(sulfones) **2a-2h**. S2

1.2. Synthesis of the dihydroimidazol-4-ones **3e**, **3g**, **6a** and **21**. S6

1.3. Synthesis of methyl 2-((1-benzoyl-2-(benzylthio)-4-oxo-4,5-dihydro-1*H*-imidazol-5-yl)methyl)acrylate **3h**. S10

2. General procedure for the catalytic addition of hydantoin surrogates **8** and **9** to **1a**. S11

2.1. Synthesis of 5-benzyl-2-(benzylthio)-5-(2,2-bis(phenylsulfonyl)ethyl)-3-phenyl-3,5-dihydro-4*H*-imidazol-4-one (**12**). S11

2.2. Synthesis of 4-benzyl-4-(2,2-bis(phenylsulfonyl)ethyl)-2-phenyloxazol-5(4*H*)-one (**13**). S12

3. General procedure for the catalytic addition of **3a** to **2** at 4 mmol scale. S13

4. Derivatization of adduct **15ah** into hydantoin **S-2**. S14

5. ^{1}H and ^{13}C NMR spectra. S19

6. HPLC chromatograms. S78

7. High Resolution Mass Spectra (HRMS) S109

8. X-Ray analysis: ORTEP diagram of compound **25b** S159

9. Computational details. S162

1. Synthesis of the starting materials.

All reagents were purchased from commercial suppliers and used without further purification, unless otherwise stated. Substrates **1a**, **1b**, **3a**, **3b**, **3c**, **3d**, **3f**, **4a**, **5a**, **6a**, **7a**, **8** and **9** were synthesized according to the reported procedures and showed NMR data identical to those reported in the literature.[1]

1.1. Synthesis of β-substituted ethylene bis(sulfones) 2a-2f.

1.1.1. Synthesis of 2*H*-benzo[*d*][1,3]dithiole 1,1,3,3-tetraoxide.

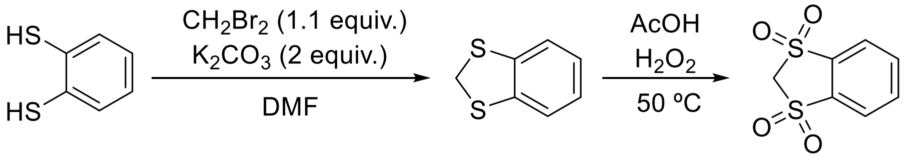

Step 1:[2] To a solution of commercially available 1,2-benzenedithiol (2.22 g, 15.6 mmol, 1 equiv.) in 25 mL of DMF K_2CO_3 (4.31 g, 31.2 mmol, 2 equiv.) was added at 0 ºC and stirred at the same temperature for 10 minutes. Afterwards, CH_2Br_2 (1.1 mL 17.2 mmol, 1.1 equiv.) was added slowly within 25 minutes. The resulting mixture was stirred at room temperature for 2 hours. Then, water was added and extracted with EtOAc three times. The combined organic layers were washed with water and brine, dried over $MgSO_4$ and concentrated under reduced pressure. The obtained crude product was purified by flash column chromatography on silica gel (Hex/EtOAc 5:1). Yield: 2.21 g, 14.35 mmol, 92%.

Step 2:[3] H_2O_2 (35% aq., 16 mL) was added to a solution of the pure 1,3-benzodithiole (2.21 g, 14.35 mmol, 1 equiv.) in acetic acid (30 mL) at room temperature and the reaction mixture was stirred for 2 hours. Afterwards, the temperature of the reaction was raised to 50 ºC in an oil bath and the stirring was continued overnight. After being 3 h at 50 ºC the mixture was turned homogeneous and a colourless precipitate was observed the next day. Once the precipitate was formed, the reaction mixture was cooled down to 0 ºC and the precipitate was filtered and washed with water (3 x 50 mL). The crude product was

[1] (a) Etxabe, J.; Izquierdo, J.; Landa, A.; Oiarbide, M.; Palomo, C. *Angew. Chem. Int. Ed.* **2015**, *54*, 6883–6886. (b) Izquierdo, J.; Etxabe, J.; Duñabeitia, E.; Landa, A.; Oiarbide, M.; Palomo, C. *Chem. Eur. J.* **2018**, *24*, 7217–7227.
[2] Mąkosza, M.; Sypniewski, M. *Phosphorus, Sulfur, and Silicon*, **1993**, *80*, 89–94.
[3] Kündig, E.P.; Cunningham Jr., A. F. *Tetrahedron,* **1988**, *44*, 22, 6855–6860.

recrystallized from ethanol to afford the desired white solid. Yield: 2.29 g, 10.49 mmol, 73%.

1.1.2. Synthesis of 2*H*-benzo[*d*][1,3]dithiole 1,1,3,3-tetraoxides 2a-2f.[4]

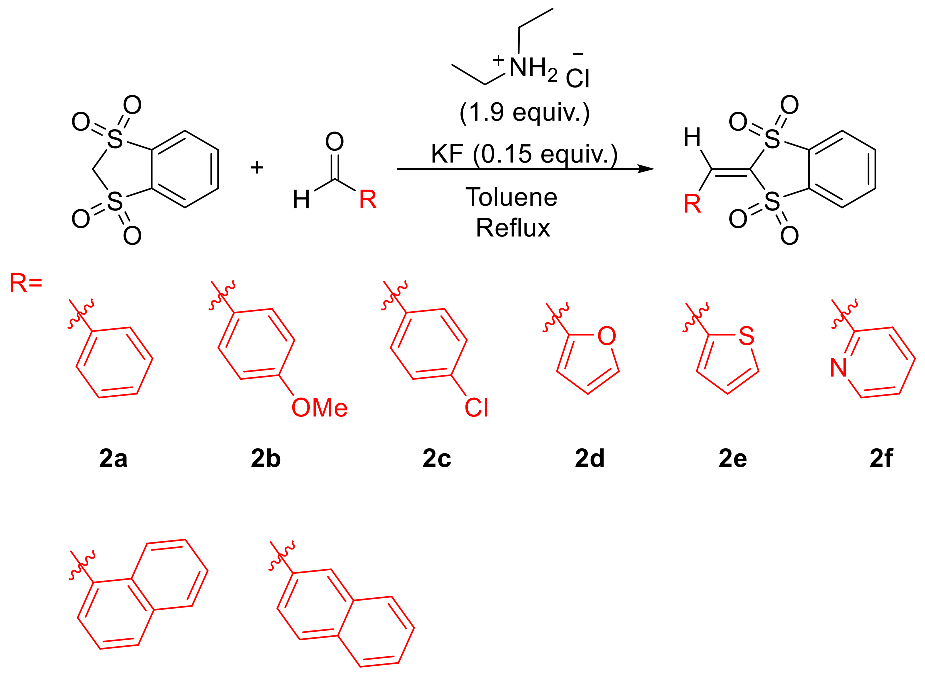

To a solution of 2*H*-benzo[*d*][1,3]dithiole 1,1,3,3-tetraoxide (872 mg, 4 mmol, 1 equiv.) in 110 mL of toluene, 5 equivalents of the corresponding aldehyde (20 mmol), 1.9 equiv. of the diethylamine hydrochloride (833 mg, 7.6 mmol) and 0.15 equivalents of KF (56 mg 0.6 mmol) were added. The reaction mixture was stirred at reflux (150 ºC) until consumption of the dithiole (around 6 hours, monitored by ^{1}H NMR). The crude product was then concentrated under reduced pressure, dissolved in dichloromethane, washed with water (3 x 50 mL) and dried over $MgSO_4$. The white-yellow solid obtained was crushed with diethyl ether and filtered to afford the desired pure product.

2-Benzylidene-2*H*-benzo[*d*][1,3]dithiole 1,1,3,3-tetraoxide (2a)

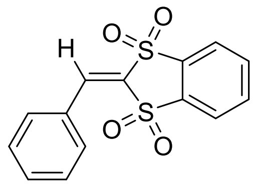

The title compound was prepared from benzaldehyde (2.0 mL, 20 mmol, 5 equiv.) according to the general procedure. White solid, m.p.= 218-219 ºC. Yield: 1.09 g, 3.56 mmol, 89%. ^{1}H NMR (300 MHz, $CDCl_3$) δ 8.13 – 8.04 (m, 3H), 8.00 – 7.91 (m, 4H), 7.70 –

[4] Asahara, H., Mayr, H. *Chem. Asian. J.* **2012**, *7*, 1401–1407.

7.54 (m, 3H). ^{13}C NMR (75 MHz, $CDCl_3$) δ 142.9, 137.5, 137.1, 135.3, 135.2, 134.0, 133.9, 132.6, 129.44, 129.35, 122.5, 122.2. HRMS (ESI) m/z: $[M+Na]^+$ Calcd. for $C_{14}H_{10}O_4S_2Na$ 328.9918; Found 328.9924. IR (cm^{-1}) = 3085, 3010, 1591, 1566.

2-(4-Methoxybenzylidene)-2*H*-benzo[*d*][1,3]dithiole 1,1,3,3-tetraoxide (2b)

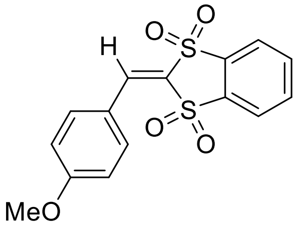

The title compound was prepared from 4-methoxybenzaldehyde (2.43 mL, 20 mmol, 5 equiv.) according to the general procedure. Yellow solid, m.p.= 222-223 ºC. Yield: 1.06 g, 3.16 mmol, 79%. ^1H NMR (300 MHz, $CDCl_3$) δ 8.19 – 7.81 (m, 7H), 7.13 – 6.95 (m, 2H), 3.92 (s, 3H). ^{13}C NMR (75 MHz, $CDCl_3$) δ 164.7, 142.2, 137.9, 137.7, 135.6, 135.0, 134.9, 130.6, 122.5, 122.4, 122.1, 115.1, 55.8. HRMS (ESI) m/z: $[M+H]^+$ Calcd. for $C_{15}H_{13}O_5S_2$ 337.0204; Found 337.0204. IR (cm^{-1}) = 2989, 1748, 1585, 1555, 1506.

2-(4-Chlorobenzylidene)-2*H*-benzo[*d*][1,3]dithiole 1,1,3,3-tetraoxide (2c)

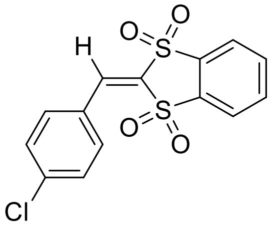

The title compound was prepared from 4-chlorobenzaldehyde (2.81 g, 20 mmol, 5 equiv.) according to the general procedure. White solid, m.p. = 250-253 ºC. Yield: 0.89 g, 2.6 mmol, 65%. ^1H NMR (300 MHz, $CDCl_3$) δ 8.11 – 7.82 (m, 7H), 7.56 – 7.50 (m, 2H). ^{13}C NMR (75 MHz, $CDCl_3$) δ 141.3, 135.4, 135.3, 133.8, 129.8, 122.6, 122.3. HRMS (ESI) m/z: $[M+Na]^+$ Calcd. for $C_{14}H_9ClO_4S_2Na$ 362.9529, Found: 362.9535. IR (cm^{-1}) = 3088, 1738, 1584.

2-(Furan-2-ylmethylene)-2*H*-benzo[*d*][1,3]dithiole 1,1,3,3-tetraoxide (2d)

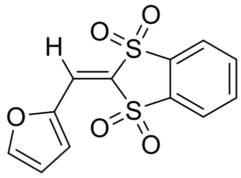

The title compound was prepared from furan-2-carbaldehyde (1.66 mL, 20 mmol, 5 equiv.) according to the general procedure. Yellow solid, m.p.= 278-282 ºC. Yield: 0.65 g, 2.17 mmol, 54%. ^1H NMR (300 MHz, $CDCl_3$) δ 8.16 – 7.98 (m, 2H), 7.98 – 7.87 (m, 3H), 7.70 (s, 1H), 7.52 – 7.39 (m, 1H), 6.73 (dd, J = 3.7, 1.7, 1H).^{13}C NMR (75 MHz, $CDCl_3$) δ 150.3, 135.3, 135.0, 125.6, 125.3, 122.5, 122.1, 114.7. HRMS (ESI) m/z: $[M+H]^+$ Calcd. for $C_{12}H_9O_5S_2$ 296.9891; Found 296.9895. IR (cm^{-1}) = 3086, 3011, 1608, 1528.

2-(Thiophen-2-ylmethylene)-2*H*-benzo[*d*][1,3]dithiole 1,1,3,3-tetraoxide (2e)

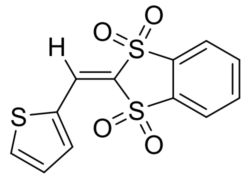

The title compound was prepared from thiophene-2-carbaldehyde (1.87 mL, 20 mmol, 5 equiv.) according to the general procedure. Yellow solid, m.p.= 255-259 ºC. Yield: 0.99 g, 3.18 mmol, 80%. ^{1}H NMR (300 MHz, $CDCl_3$) δ 8.13 – 7.89 (m, 7H), 7.29 (dd, *J* = 5.0, 3.9 Hz, 1H). ^{13}C NMR (75 MHz, $CDCl_3$) δ 139.4, 138.1, 135.2, 135.1, 132.9, 129.7, 122.4, 122.2. HRMS (ESI) m/z: [M+H]$^+$ Calcd. for $C_{12}H_9O_4S_3$ 312.9663; Found 312.9667. IR (cm^{-1}) = 3084, 2994, 1586, 1565.

2-(Pyridin-2-ylmethylene)-2*H*-benzo[*d*][1,3]dithiole 1,1,3,3-tetraoxide (2f)

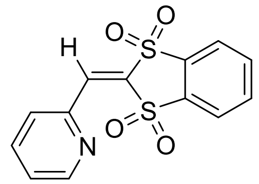

The title compound was prepared from picolinaldehyde (1.9 mL, 20 mmol, 5 equiv.) according to the general procedure. Green solid, m.p.= 179-180ºC. Yield: 0.57 g, 1.87 mmol, 47%. ^{1}H NMR (300 MHz, $CDCl_3$) δ 8.92 (dt, *J* = 4.7, 1.2 Hz, 1H), 8.33 – 7.72 (m, 7H), 7.48 (m, 1H). ^{13}C NMR (126 MHz, $CDCl_3$) δ 150.7, 140.7, 137.2, 135.5, 134.8, 128.2, 126.8, 123.0, 122.0. HRMS (ESI) m/z: [M+H]$^+$ Calcd. for $C_{13}H_{10}NO_4S_2$ 308.0051; Found 308.0057. IR (cm^{-1}) = 3083, 3013, 1613, 1560.

2-(naphthalen-1-ylmethylene)-2*H*-benzo[*d*][1,3]dithiole 1,1,3,3-tetraoxide (2g)

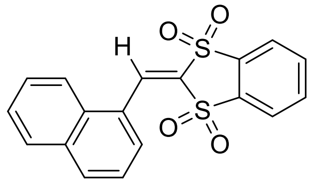

The title compound was prepared from 1-naphthaldehyde (2.71 mL, 20 mmol, 5 equiv.) according to the general procedure. Yellow solid, m.p.= 254-257 ºC. Yield: 1.16 g, 3.25 mmol, 81%. ^{1}H NMR (300 MHz, CD_2Cl_2) δ 8.90 (s, 1H), 8.30 – 7.53 (m, 11H). ^{13}C{^{1}H} NMR (75 MHz, CD_2Cl_2) δ 141.3, 135.9, 135.8, 134.7, 131.8, 129.7, 128.7, 127.6, 125.9, 124.0, 122.9, 122.6. HRMS (ESI) m/z: [M+H]$^+$ Calcd. for $C_{18}H_{13}O_4S_2$ 357.0250; Found 357.0252. IR (cm^{-1})= 3084, 1603, 1566.

2-(Naphthalen-2-ylmethylene)-2*H*-benzo[*d*][1,3]dithiole 1,1,3,3-tetraoxide (2h)

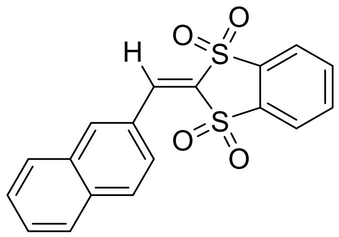

The title compound was prepared from 2-naphthaldehyde (3.12 g, 20 mmol, 5 equiv.) according to the general procedure. Yellow solid, m.p.= 227-229 ºC. Yield: 1.20 g, 3.36 mmol, 84%. ^{1}H NMR (300 MHz, CD_2Cl_2) δ 8.41 (bs, 1H), 8.20 (bs, 1H), 8.08 (m, 2H), 8.03 – 7.91 (m, 6H), 7.65

(m, 2H). ^{13}C NMR (75 MHz, CD_2Cl_2) δ 143.2, 137.9, 137.6, 136.0, 135.8, 135.7, 134.3, 133.2, 130.1, 130.0, 129.5, 128.4, 128.0, 127.6, 127.5, 122.9, 122.5. HRMS (ESI) m/z: $[M+H]^+$ Calcd. for $C_{18}H_{13}O_4S_2$ 357.0250; Found 357.0252. IR (cm^{-1}) = 3081, 1738, 1591, 1565.

1.2. Synthesis of the dihydroimidazol-4-ones 3e, 3g, 6a and 21. [5]

1.2.1. Synthesis of R-substituted 1-benzoyl-2-thioxoimidazolidin-4-ones for 3e, 3g, and 6a.

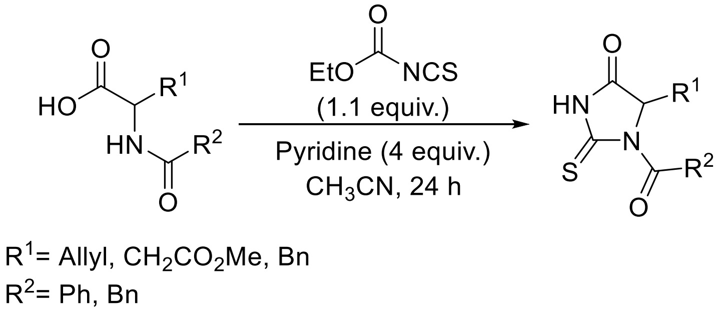

Pyridine (4 equiv.) and ethoxycarbonyl isothiocyanate (1.1 equiv.) were added at room temperature to the suspension of the corresponding N-Benzoyl amino acid in CH_3CN (5 mL/mmol). The reaction was then stirred for 24 h at different temperatures depending on the R^2 substituent until it was over (monitored by ^{1}H NMR). Afterwards, the solvent was evaporated under reduced pressure and the obtained crude product was purified by silica gel flash column chromatography (hexane/ethyl acetate from 3:1 to 1:1) to give the desired pure product.

5-Allyl-1-benzoyl-2-thioxoimidazolidin-4-one.

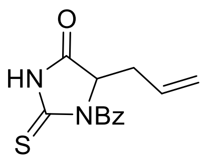

The title compound was prepared from 2-benzamidopent-4-enoic acid (1.52 g, 6.93 mmol, 1 equiv.), pyridine (2.13 mL, 27.7 mmol, 4 equiv.) and ethyl carbonisothiocyanatidate (898.9 µL, 7.62 mmol, 1.1 equiv.) in CH_3CN (35 mL) at 40 ºC in an oil bath according to the general procedure. Silica gel flash column chromatography (eluent: hexane/ethyl acetate from 3:1 to 1:1). Yellow solid, m.p.= 138-140 ºC. Yield: 1.39 g, 5.33 mmol, 77%. ^{1}H NMR (300

[5] Fort the synthesis of dihydroimidazolons **3a**, **3b**, **3c**, **3d** and **3f**, see: Izquierdo, J.; Etxabe, J.; Duñabeitia, E.; Landa, A.; Oiarbide, M.; Palomo, C. *Chem. Eur. J.*, **2018**, *24*, 7217–7227.

MHz, $CDCl_3$) δ 8.38 (bs, 1H), 7.82 – 7.37 (m, 5H), 5.78 (m, 1H), 5.32 – 5.17 (m, 2H), 5.13 (dd, J = 5.1, 3.8 Hz, 1H), 2.98 – 2.79 (m, 2H). ^{13}C NMR (75 MHz, $CDCl_3$) δ 180.4, 171.9, 169.9, 133.6, 133.2, 129.8, 129.7, 128.8, 128.3, 121.9, 62.9, 33.2.

Methyl 2-(3-benzoyl-5-oxo-2-thioxoimidazolidin-4-yl)acetate.

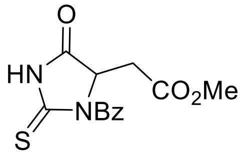

The title compound was prepared from 2-benzamido-4-methoxy-4-oxobutanoic acid (2 g, 7.96 mmol, 1 equiv.), pyridine (2.45 mL, 31.84 mmol, 4 equiv.) and ethyl carbonisothiocyanatidate (1.04 mL, 8.76 mmol, 1.1 equiv.) in CH_3CN (40 mL) at 40 ºC in an oil bath according to the general procedure. Silica gel flash column chromatography (eluent: hexane/ethyl acetate from 3:1 to 1:1). Yellow solid, m.p. = 114-117 ºC. Yield: 0.97 g, 3.30 mmol, 42%. ^1H NMR (300 MHz, $CDCl_3$) δ 8.68 (bs, 1H), 7.73 – 7.34 (m, 5H), 5.08 (dd, J = 4.3, 3.5 Hz, 1H), 3.72 (s, 3H), 3.22 (t, J = 4.1 Hz, 2H). ^{13}C NMR (75 MHz, $CDCl_3$) δ 180.8, 171.5, 170.1, 169.9, 133.8, 133.2, 129.7, 128.3, 59.4, 52.6, 32.4.

5-Benzyl-1-(2-phenylacetyl)-2-thioxoimidazolidin-4-one.

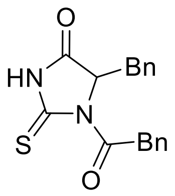

The title compound was prepared from (2-phenylacetyl)phenylalanine (1.69 g, 5.98 mmol, 1 equiv.), pyridine (1.93 mL, 23.92 mmol, 4 equiv.) and ethyl carbonisothiocyanatidate (0.78 mL, 6.58 mmol, 1.1 equiv.) in CH_3CN (35 mL) at room temperature according to the general procedure. Silica gel flash column chromatography (eluent: hexane/ethyl acetate from 3:1 to 1:1). White solid, m.p.= 146-150 ºC. Yield: 1.47 g, 4.55 mmol, 76%. ^1H NMR (300 MHz, $CDCl_3$) δ 8.26 (bs, 1H), 7.50 – 7.12 (m, 8H), 7.03 – 6.91 (m, 2H), 5.05 (dd, J = 6.0, 2.6 Hz, 1H), 4.60 (d, J = 3.6 Hz, 2H), 3.53 (dd, J = 14.0, 6.0 Hz, 1H), 3.29 (dd, J = 14.0, 2.5 Hz, 1H). ^{13}C NMR (75 MHz, $CDCl_3$) δ 179.7, 171.8, 171.7, 133.2, 133.1, 130.0, 129.6, 129.2, 128.8, 128.7, 127.9, 127.8, 127.5, 64.6, 44.9, 35.1.

1.2.2. Synthesis of 1-benzoyl-2-thioxoimidazolidin-4-one for 21.[6]

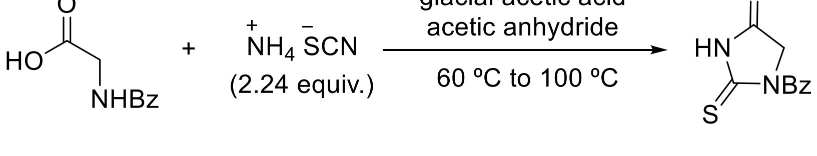

[6] Papeo, G.; Posteri, H.; Borghi, D.; Varasi, M. *Org.Lett.* **2005**, *7*, 5641–5644.

Hippuric acid (4 g, 22.32 mmol) and ammonium thiocyanate (3.8 g, 50 mmol, 2.24 equiv.) were grounded together in a mortar and suspended in a mixture of glacial acetic acid (2 mL) and acetic anhydride (18 mL). The reaction was then heated in an oil bath for 1 h at 60 ºC and for another hour at 100 ºC. The red solution obtained was cooled, diluted with 100 mL of cold water and stirred overnight. Afterwards, the precipitate was filtered and washed with water. If more purification was needed, the obtained solid was dissolved in ethyl acetate and purified by silica gel flash chromatography (eluent: hexane/ethyl acetate, from 3:1 to 1:1). Orange solid, m.p.= 159-161 ºC. Yield: 3.86 g, 17.53 mmol, 78%. ^{1}H NMR (300 MHz, $CDCl_3$) δ 8.55 (s, 1H), 7.73 – 7.36 (m, 5H), 4.67 (s, 2H). ^{13}C NMR (75 MHz, $CDCl_3$) δ 180.1, 169.4, 168.2, 133.3, 132.9, 129.2, 128.4, 52.7.

1.2.3. Synthesis of S-benzyl dihydroimidazol-4-ones 3e, 3g, 6a and 21.

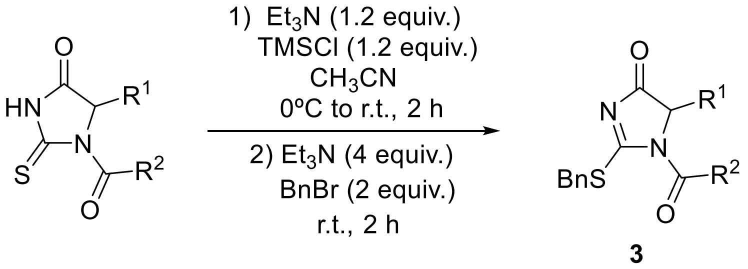

3e R^1= Allyl, R^2= Ph
3g R^1= CH_2CO_2Me, R^2= Ph
6a R^1= Bn, R^2= Bn
21 R^1= H, R^2= Ph

The corresponding thiohydantoin (1 equiv.) was dissolved in freshly distilled anhydrous CH_3CN (2 mL/mmol) and cooled down to 0ºC. Subsequently, the reaction mixture was treated with freshly distilled triethylamine (1.2 equiv.) and, after 5 min, freshly distilled TMSCl (1.2 equiv.) was added causing a snappy precipitation. The mixture was then warmed up to room temperature and stirred for 2 hours. Then, freshly distilled triethylamine (4 equiv.) and benzyl bromide (2 equiv.) were added. After 2-3 h the reaction was finished and the mixture was diluted with dichloromethane and washed with water. The organic layers were combined, dried over Mg_2SO_4, filtered and concentrated under reduced pressure. The crude product was purified by silica gel flash column chromatography (hexane/ethyl acetate 3:1 to1:1).

5-Allyl-1-benzoyl-2-(benzylthio)-1,5-dihydro-4*H*-imidazol-4-one (3e).

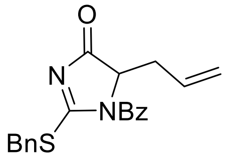

The title compound was prepared from 5-allyl-1-benzoyl-2-thioxoimidazolidin-4-one (3.4 g, 13.06 mmol, 1 equiv.), TMSCl (1.9 mL, 15.7 mmol, 1.2 equiv.), Et_3N (2.2 mL, 15.7 mmol, 1.2 equiv.) and afterwards Et_3N (7.4 mL, 52.2 mmol, 4 equiv.), and BnBr (3.1 mL, 26.1 mmol, 2 equiv.) in CH_3CN (26 mL) according to the general procedure. Silica gel flash column chromatography (eluent: hexane/ethyl acetate from 3:1 to 1:1). Orange solid, m.p.= 110-112 ºC. Yield: 3.23 g, 9.22 mmol, 71%. ^{1}H NMR (300 MHz, $CDCl_3$) δ 7.65 – 7.23 (m, 10H), 5.47 (m, 1H), 5.11 (dt, *J* = 9.8, 1.5 Hz, 1H), 4.99 (m, 1H), 4.69 (dd, *J* = 5.8, 2.9 Hz, 1H), 4.60 – 4.34 (m, 2H), 2.61 (m, 1H), 2.21 – 2.03 (m, 1H). ^{13}C NMR (75 MHz, $CDCl_3$) δ 185.9, 184.6, 167.1, 135.1, 133.4, 132.8, 129.5, 129.1, 128.8, 127.9, 127.6, 121.2, 64.0, 38.3, 34.4. HRMS (ESI) m/z: $[M+H]^+$ Calcd. for $C_{20}H_{19}N_2O_2S$ 351.1162; Found 351.1170. IR (cm^{-1}) = 3031, 1720, 1679,

Methyl 2-(1-benzoyl-2-(benzylthio)-4-oxo-4,5-dihydro-1*H*-imidazol-5-yl)acetate (3g).

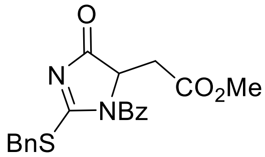

The title compound was prepared from methyl 2-(3-benzoyl-5-oxo-2-thioxoimidazolidin-4-yl)acetate (2.81 mmol, 0.821 g, 1 equiv.), TMSCl (0.42 mL, 3.38 mmol, 1.2 equiv.), Et_3N (0.47 mL, 3.38 mmol, 1.2 equiv.) and afterwards Et_3N (1.6 mL, 11.24 mmol, 4 equiv.) and BnBr (0.65 mL, 5.6 mmol, 2 equiv.) in CH_3CN (8 mL) according to the general procedure. Silica gel flash column chromatography (eluent: hexane/ethyl acetate from 3:1 to 1:1). Yield: 0.66 g, 1.73 mmol, 61%. Yellow solid, m.p. = 126-130 ºC. ^{1}H NMR (300 MHz, $CDCl_3$) δ 7.64 – 7.22 (m, 10H), 4.77 (dd, *J* = 5.5, 3.2 Hz, 1H), 4.56 – 4.39 (m, 2H), 3.62 (s, 3H), 2.98 (dd, *J* = 17.3, 3.3 Hz, 1H), 2.69 (dd, *J* = 17.3, 5.5 Hz, 1H). ^{13}C NMR (75 MHz, $CDCl_3$) δ 185.3, 183.8, 169.0, 166.9, 135.0, 133.8, 133.0, 132.9, 130.0, 129.4, 129.1, 128.8, 128.0, 127.9, 60.3, 52.2, 38.5, 33.7. HRMS (ESI) m/z: $[M+H]^+$ Calcd. for $C_{20}H_{19}N_2O_4S$ 383.1060; Found 383.1068. IR (cm^{-1}) = 3348, 2924, 1732, 1683.

5-Benzyl-1-(2-phenylacetyl)-2-thioxoimidazolidin-4-one (6a).

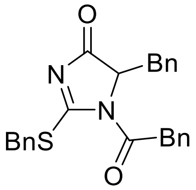

The title compound was prepared from 5-benzyl-1-(2-phenylacetyl)-2-thioxoimidazolidin-4-one (4.29 mmol, 1.42 g, 1 equiv.), TMSCl (0.67 mL, 5.27 mmol, 1.2 equiv.), Et_3N (0.73 mL, 5.27 mmol, 1.2 equiv.) and afterwards Et_3N (2.45 mL, 17.56 mmol, 4 equiv.) and BnBr (1.04

mL, 8.78 mmol, 2 equiv.) in CH_3CN (9 mL) according to the general procedure. Silica gel flash column chromatography (eluent: hexane/ethyl acetate from 3:1 to 1:1). White solid, m.p.= 111-114 ºC. Yield: 31%. ^{1}H NMR (300 MHz, $CDCl_3$) δ 7.59 – 7.05 (m, 15H), 4.64 (dd, J = 5.3, 3.8 Hz, 1H), 4.39 – 4.19 (m, 2H), 3.90 – 3.74 (m, 2H), 3.44 (qd, J = 14.3, 4.6 Hz, 2H). ^{13}C NMR (75 MHz, $CDCl_3$) δ 185.7, 184.3, 168.4, 135.1, 133.2, 131.6, 129.5, 129.4, 129.2, 129.0, 128.8, 128.1, 128.0, 127.9, 64.0, 42.8, 38.1, 38.1. HRMS (ESI) m/z: $[M+H]^+$ Calcd. for $C_{25}H_{23}N_2O_2S$ 415.1475; Found 415.1484. IR (cm^{-1}) = 3053, 1724, 1672.

1-Benzoyl-2-(benzylthio)-1,5-dihydro-4*H*-imidazol-4-one (21).

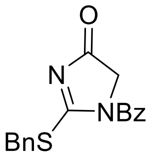

The title compound was prepared from 1-benzoyl-2-thioxoimidazolidin-4-one (13.5 mmol, 3 g, 1 equiv.), TMSCl (2 mL, 16.2 mmol, 1.2 equiv.), Et_3N (2.25 mL, 16.2 mmol, 1.2 equiv.) and afterwards Et_3N (7.6 mL, 54 mmol, 4 equiv.) and BnBr (3.16 mL, 27 mmol, 2 equiv.) in CH_3CN (26 mL) according to the general procedure. Silica gel flash column chromatography (eluent: hexane/ethyl acetate from 3:1 to 1:1). Brown solid, m.p.= 87-89 ºC. Yield: 3.39 g, 10.92 mmol, 81%. ^{1}H NMR (300 MHz, $CDCl_3$) δ 7.64 – 7.28 (m, 10H), 4.50 (s, 2H), 4.32 (s, 2H). ^{13}C NMR (75 MHz, $CDCl_3$) δ 186.3, 181.7, 167.0, 135.2, 133.2, 132.6, 129.6, 129.1, 128.9, 128.5, 128.0, 54.6, 38.3. HRMS (ESI) m/z: $[M+H]^+$ Calcd. for $C_{17}H_{15}N_2O_2S$ 311.0849; Found 311.0857. IR (cm^{-1}) = 3059, 1714, 1657.

1.3. Synthesis of methyl 2-((1-benzoyl-2-(benzylthio)-4-oxo-4,5-dihydro-1*H*-imidazol-5-yl)methyl)acrylate 3h.

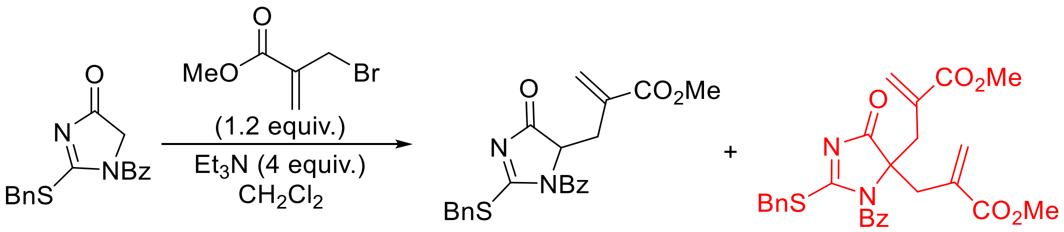

21 **3h** **3h'**

To a solution of 1-benzoyl-2-(benzylthio)-1,5-dihydro-4*H*-imidazol-4-one **21** (1.5 g, 4.8 mmol, 1 equiv.) in DCM (20 mL), 1.2 equivalents of methyl 2-(bromomethyl)acrylate

(0.7 mL, 5.8 mmol) were added and the reaction mixture was cooled to 0 ºC. Afterwards, 2 equivalents of Et_3N (1.4 mL, 9.7 mmol) were added dropwise and the mixture was stirred at room temperature 5 days. The crude product was treated employing silica gel column chromatography (eluent: hexane/ethyl acetate 1:1) affording the mixture of the products **3h** and **3h'** in a mol ratio of **3h**/**3h'** 2:1. Yellow solid. The title compound **3h** could not be separated from compound **3h'** by column chromatography so for the following asymmetric addition reaction the obtained mixture was used as starting material. ^{1}H NMR **3h** (300 MHz, $CDCl_3$) δ 7.72 – 7.12 (m, 10H), 6.19 (d, *J* = 1.0 Hz, 1H), 5.63 (q, *J* = 1.0 Hz, 1H), 4.90 (dd, *J* = 5.9, 4.9 Hz, 1H), 4.44 (d, *J* = 0.7 Hz, 2H), 3.64 (s, 3H), 2.75 (dd, *J* = 14.1, 5.9, 1H), 2.61 (dd, *J* = 14.1, 4.8 Hz, 1H).

2. General procedure for the catalytic addition of hydantoin surrogates 8 and 9 to 1a.

2.1. Synthesis of 5-benzyl-2-(benzylthio)-5-(2,2-bis(phenylsulfonyl)ethyl)-3-phenyl-3,5-dihydro-4*H*-imidazol-4-one (12).

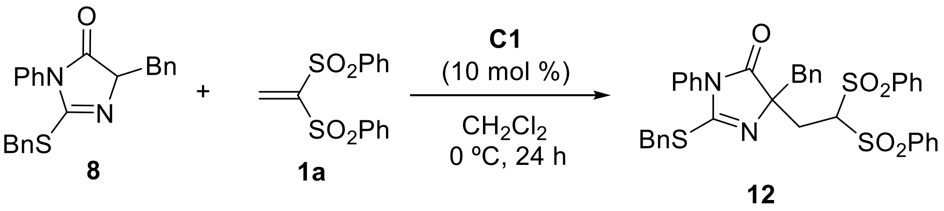

In a 5 mL test tube, 5-benzyl-2-(benzylthio)-3-phenyl-3,5-dihydro-4*H*-imidazol-4-one (37.2 mg, 0.1 mmol, 1 equiv.) was dissolved in CH_2Cl_2 (1 mL) at room temperature and after cooling the solution down to 0 ºC, the corresponding vinylic sulfone (0.12 mmol, 37 mg, 1.2 equiv.) and the catalyst **C1** (0.01 mmol, 6 mg, 10 mol %) were added. The mixture was stirred at 0 ºC for 24 hours and the crude product was directly submitted to silica gel flash column chromatography (eluent: hexane/ethyl acetate, from 3:1 to 1:1). White foam, yield: 49 mg, 0.071 mmol, 71%. 2% *ee*. ^{1}H NMR (300 MHz, $CDCl_3$) δ 8.10 – 7.00 (m, 23H), 6.73 – 6.63 (m, 2H), 5.39 (t, *J* = 4.9 Hz, 1H), 4.36 (s, 2H), 3.12 – 2.95 (m, 2H), 2.90 (d, *J* = 4.9 Hz, 2H). ^{13}C NMR (75 MHz, $CDCl_3$) δ 180.7, 162.9, 140.5, 138.2, 136.7, 136.0, 135.0, 134.8, 134.6, 134.2, 131.7, 130.7, 130.5, 129.9, 129.7, 129.32, 129.28, 129.25, 129.20, 129.1, 128.8, 128.5, 127.93, 127.88, 127.8, 127.3, 79.2, 72.9, 45.5, 34.7, 31.4. IR (cm^{-1}) = 3062, 3031, 2917, 1740. HRMS (ESI) m/z: $[M+H]^+$ Calcd. for $C_{37}H_{33}N_2O_5S_3$ 681.1546; Found 681.1540. The *ee* value was determined by HPLC

analysis (Daicel Chiralpak IA, hexane/isopropanol 50:50, flow rate= 0.5 mL/min, retention times: 17.7 min (major) and 21.8 min (minor).

2.2. Synthesis of 4-benzyl-4-(2,2-bis(phenylsulfonyl)ethyl)-2-phenyloxazol-5(4*H*)-one (13).

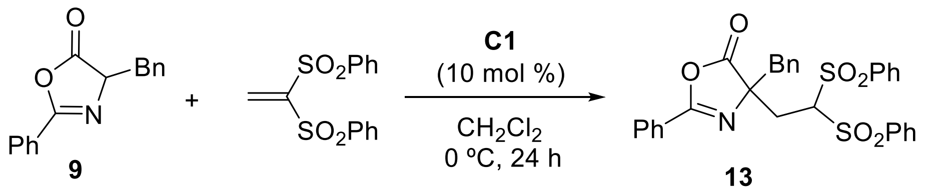

In a 5 mL test tube, from 4-benzyl-2-phenyloxazol-5(4*H*)-one (25.1 mg, 0.1 mmol, 1 equiv.) was dissolved in CH_2Cl_2 (1 mL) at room temperature and after cooling the solution down to 0 ºC, the corresponding vinylic sulfone (0.12 mmol, 37 mg, 1.2 equiv.) and the catalyst **C1** (0.01 mmol, 6 mg, 10 mol %) were added. The mixture was stirred at 0 ºC for 24 hours and the crude product was directly submitted to silica gel flash column chromatography (eluent: hexane/ethyl acetate, 3:1). White foam, yield: 51 mg, 0.089 mmol, 89%. 58% *ee*. ^{1}H NMR (300 MHz, $CDCl_3$) δ 8.09 – 7.02 (m, 20H), 5.16 (dd, *J* = 6.6, 3.5 Hz, 1H), 3.15 (s, 2H), 3.04 – 2.80 (m, 2H).^{13}C NMR (75 MHz, $CDCl_3$) δ 178.6, 161.8, 140.5, 137.5, 137.0, 134.9, 134.8, 133.2, 130.5, 130.1, 130.0, 129.3, 129.1, 128.9, 128.6, 128.3, 128.2, 127.7, 127.1, 125.4, 79.4, 71.2, 45.5, 31.7. HRMS (ESI) m/z: $[M+H]^+$ Calcd. for $C_{30}H_{26}NO_6S_2$ 560.1196; Found 560.1205. IR (cm^{-1}) = 3067, 3031, 2933, 1798, 1732, 1649. The *ee* value was determined by HPLC analysis (Daicel Chiralpak IA, hexane/isopropanol 60:40, flow rate= 0.5 mL/min, retention times: 22.0 min (major) and 26.4 min (minor).

3. General procedure for the catalytic addition of 3a to 2 at 4 mmol scale.

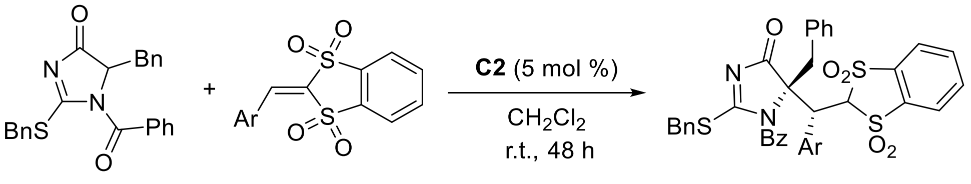

In a 100 mL round bottom flask, 1-benzoyl-2-(benzylthio)-5-methyl-1,5-dihydro-4*H*-imidazol-4-one **3a** (1.6 g, 4 mmol, 1 equiv.) was dissolved in 40 mL of CH_2Cl_2 at room temperature and after cooling the solution down to 0 ºC, the corresponding vinylic sulfone (4.8 mmol, 1.2 equiv.) and 5 mol % of catalyst **C2** (163 mg, 0.2 mmol) were added. The mixture was stirred at room temperature for 48 hours and the obtained crude product was directly submitted to silica gel flash column chromatography (eluent: hexane/ethyl acetate, from 3:1 to 1:1).

(*S*)-1-Benzoyl-5-benzyl-2-(benzylthio)-5-((*R*)-phenyl(1,1,3,3-tetraoxido-2*H*-benzo[*d*][1,3]dithiol-2-yl)methyl)-2,5-dihydro-4*H*-imidazol-4-one (15aa).

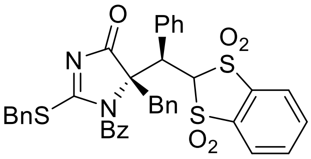

The title compound was prepared from 2-benzylidene-2*H*-bezo[*d*][1,3]dithiole 1,1,3,3-tetraoxide (1.47 g, 4.8 mmol, 1.2 equiv.) according to the general procedure. Silica gel flash column chromatography (eluent: hexane/ethyl acetate, from 3:1 to 1:1). White foam. Yield: 2.18 g, 3.08 mmol, 77% (99% *ee*).

(*S*)-1-Benzoyl-5-benzyl-2-(benzylthio)-5-((*R*)-(4-chlorophenyl)(1,1,3,3-tetraoxido-2*H*-benzo[*d*][1,3]dithiol-2-yl)methyl)-1,5-dihydro-4*H*-imidazol-4-one (15ac).

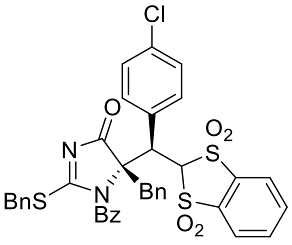

The title compound was prepared from 2-(4-chlorobenzylidene)-2*H*-benzo[*d*][1,3]dithiole 1,1,3,3-tetraoxide (1.64 g, 4.8 mmol, 1.2 equiv.) according to the general procedure. Silica gel flash column chromatography (eluent: hexane/ethyl acetate, from 3:1 to 1:1). White foam. Yield: 2.43 g, 3.28 mmol, 82% (99% *ee*).

4. Derivatization of adduct 15ah into hydantoin S-2.

Compound **15ah** obtained as described in the Article Experimental Section (Yellow foam. Yield: 171 mg, 0.22 mmol, 75% (0 ºC, 48 h) exhibited two sets of signals of similar intensities in ^{1}H NMR which were attributed to rotational isomers.

See below the ^{1}H NMR of the crude material before and after purification by silica gel column chromatography.

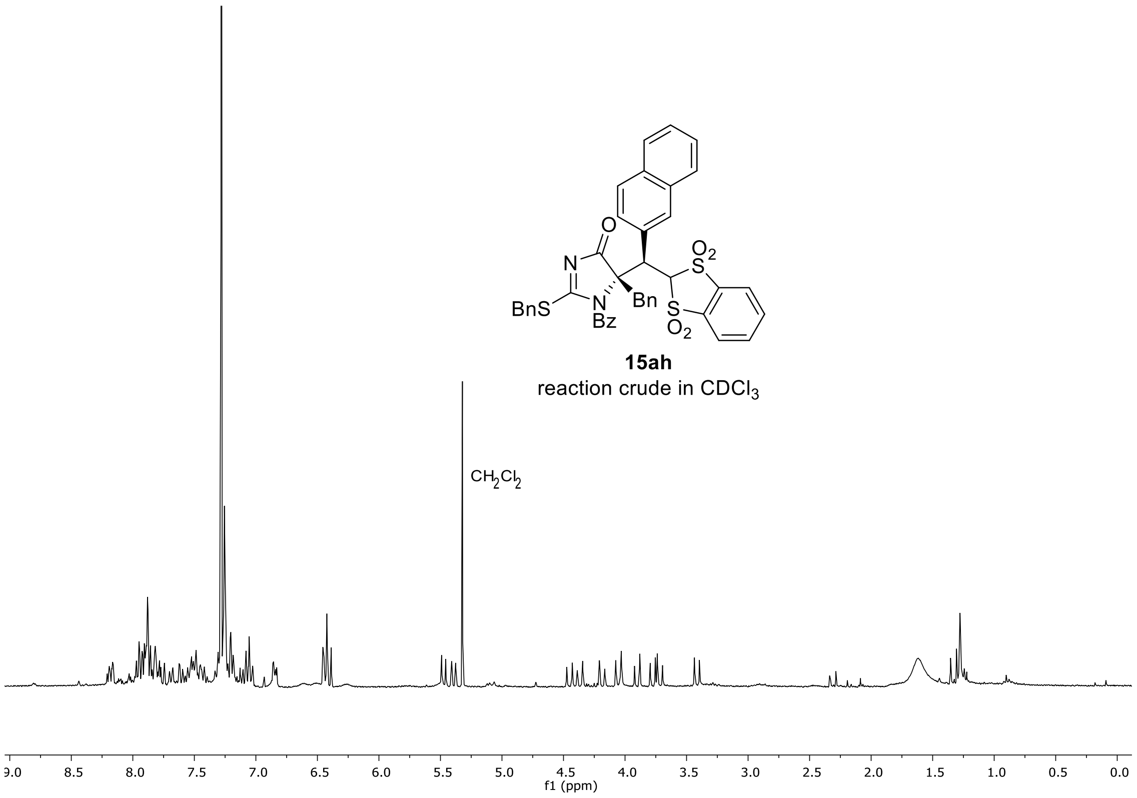

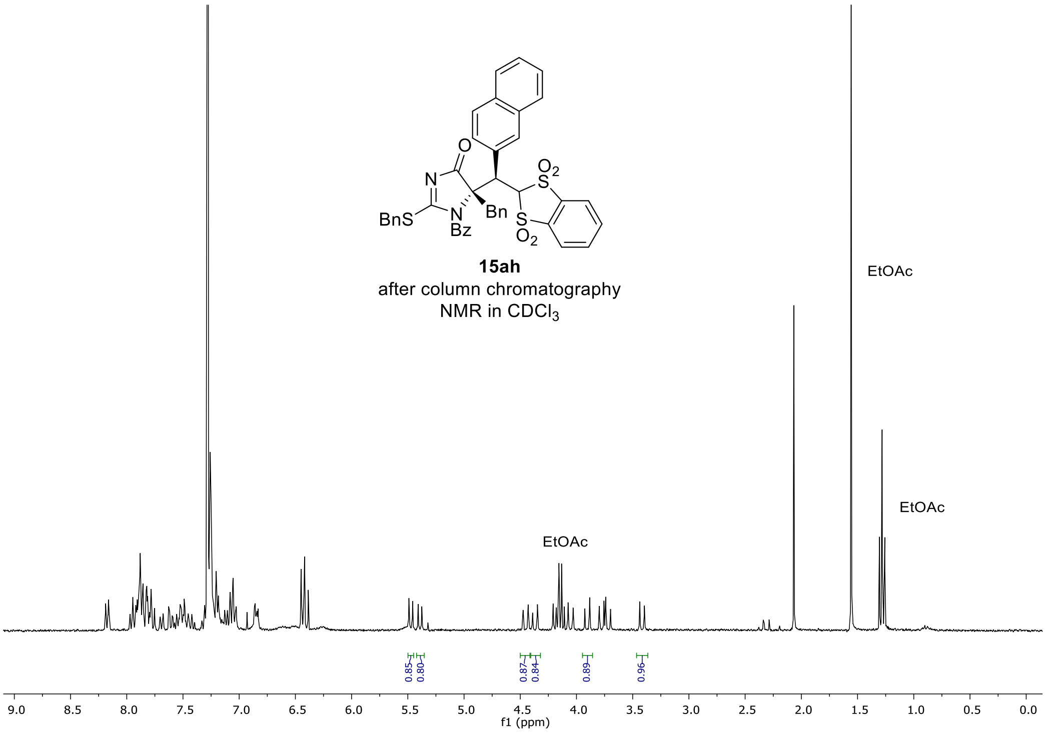

Accordingly, this material was converted into the corresponding hydantoin **S-2** using the following two-step procedure:

Conversion of compound 15ah into hydantoin S-2

Step 1: A solution of compound **15ah** (171 mg, 0.22 mmol, 1 equiv.) in TFA (4 mL) was stirred at 40 ºC for 48 h. Afterwards saturated $NaHCO_3$ was added to the reaction mixture until the pH ≥ 7, extracted with dichloromethane and the organic solvent was evaporated under reduced pressure to obtain compound **S-1**, which was used in the next step without further purification (white foam). Note that single set of signals is now observed in the ^{1}H NMR spectrum.

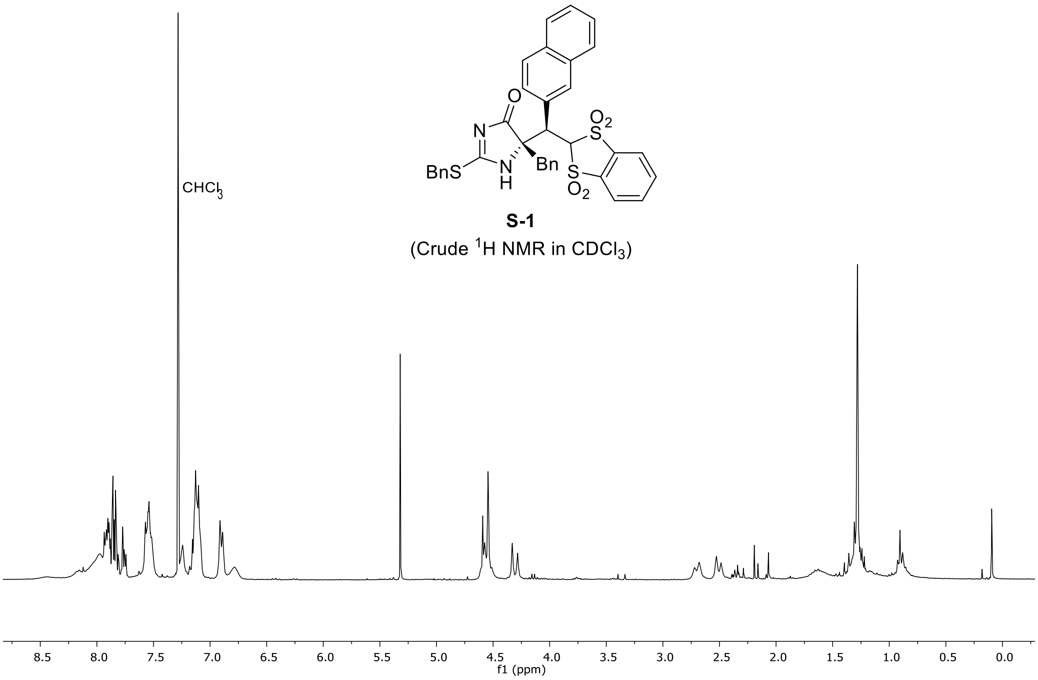

Step 2: To a solution of the crude material from the above reaction (142 mg, 0.21 mmol, 1 equiv.) in 1,4-dioxane (10 mL) at 0 ºC aqueous 6M HCl (0.40 mL) was added dropwise. Once the addition was complete, the reaction was stirred for 6 h at 80 ºC. Then, a second portion of 6M HCl (0.40 mL) was added dropwise and the mixture was stirred at 80 ºC for an additional 9 h. Then, the reaction was cooled to 0 ºC and saturated $NaHCO_3$ was added until basic pH was obtained. The aqueous layer was extracted with dichloromethane twice and the combined organic layers were dried over $MgSO_4$ and the solvent was evaporated under reduced pressure. ^{1}H NMR analysis of thus obtained crude material showed a single set of signals, indicating no rotational isomers were present any more, and that diastereomeric ratio was ≥20:1. The crude product was purified by silica gel flash column chromatography (eluent: hexane/ethyl acetate, from 3:1 to 1:1) to obtain *(S)-5-benzyl-5-((R)-naphthalen-2-yl(1,1,3,3-tetraoxido-2H-benzo[d][1,3]dithiol-2-yl)methyl)imidazolidine-2,4-dione* (**S-2**). White solid, m.p.= 215-221 ºC. Yield: 76 mg, 0.14 mmol, 64% (over two steps). $[\alpha]_D^{20}$= –67.0 (*c*=1, 99% *ee*, CH_2Cl_2). ^{1}H NMR (300 MHz, CD_2Cl_2) δ 8.06 (bs, 1H), 8.02 – 7.53 (m, 12H), 7.30 – 7.19 (m, 3H), 7.14 – 7.05 (m, 2H), 5.53 (s, 1H), 4.61 (d, *J* = 10.1 Hz, 1H), 3.22 (d, *J* = 13.6 Hz, 1H), 2.62 (d, *J* = 13.7 Hz, 1H). ^{13}C{^{1}H} NMR (75 MHz, CD_2Cl_2) δ 183.0, 174.4, 138.1, 136.0, 135.9, 134.0, 133.5, 132.9, 130.9, 129.0, 128.8, 128.4, 128.3, 127.8, 127.5, 123.0, 122.8, 76.1, 48.2, 43.9, 30.3. IR (cm^{-1}) = 3150, 3057, 2947, 1730, 1505. The *ee* value was determined

by HPLC analysis (Daicel Chiralpak ID, hexane/ethanol 30:70, flow rate= 0.5 mL/min, retention times: 17.5 (minor) and 32.4 (major).

^{1}H NMR (300 MHz, $CDCl_3$) of **S-2** (Crude material and after column chromatography)

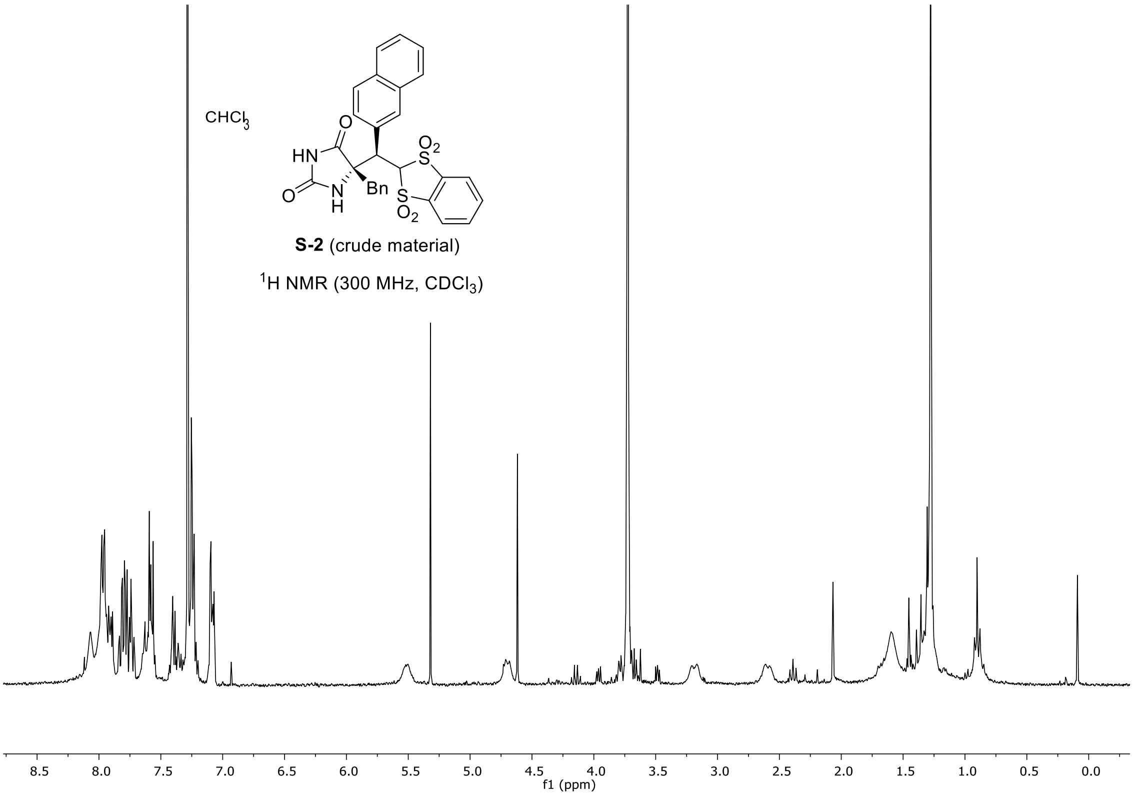

^{1}H NMR (300 MHz, CD_2Cl_2) of **S-2**:

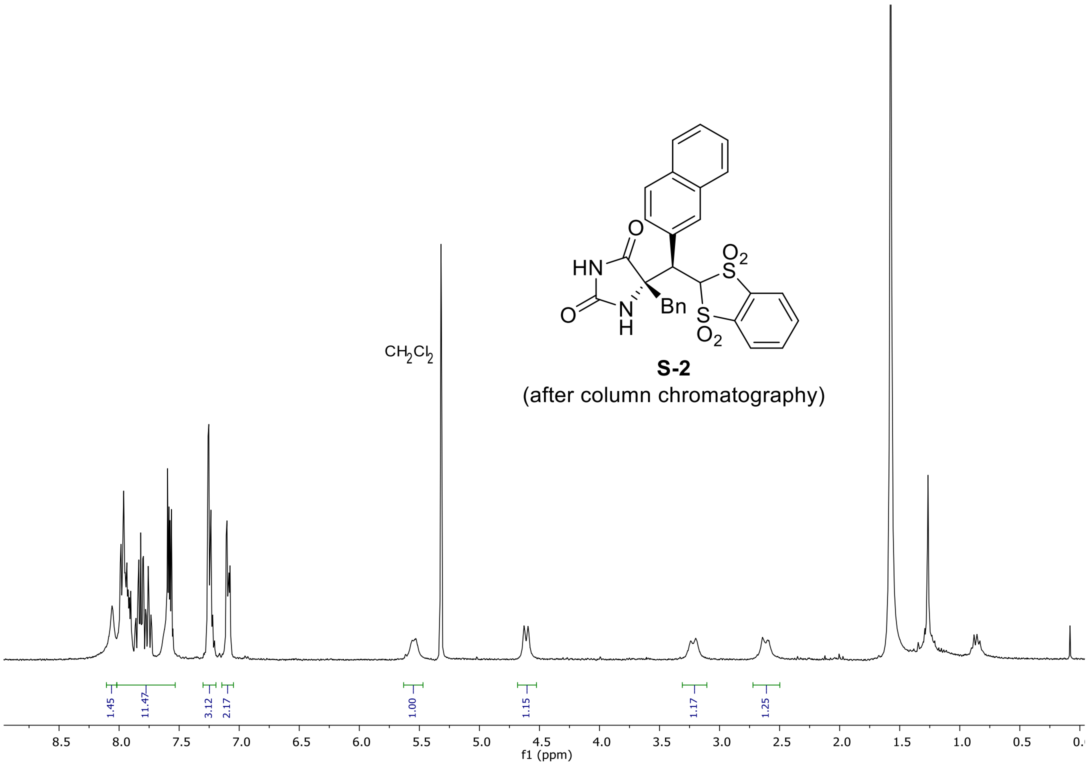

$^{13}C\{^{1}H\}$ NMR (75 MHz, CD_2Cl_2) of **S-2**:

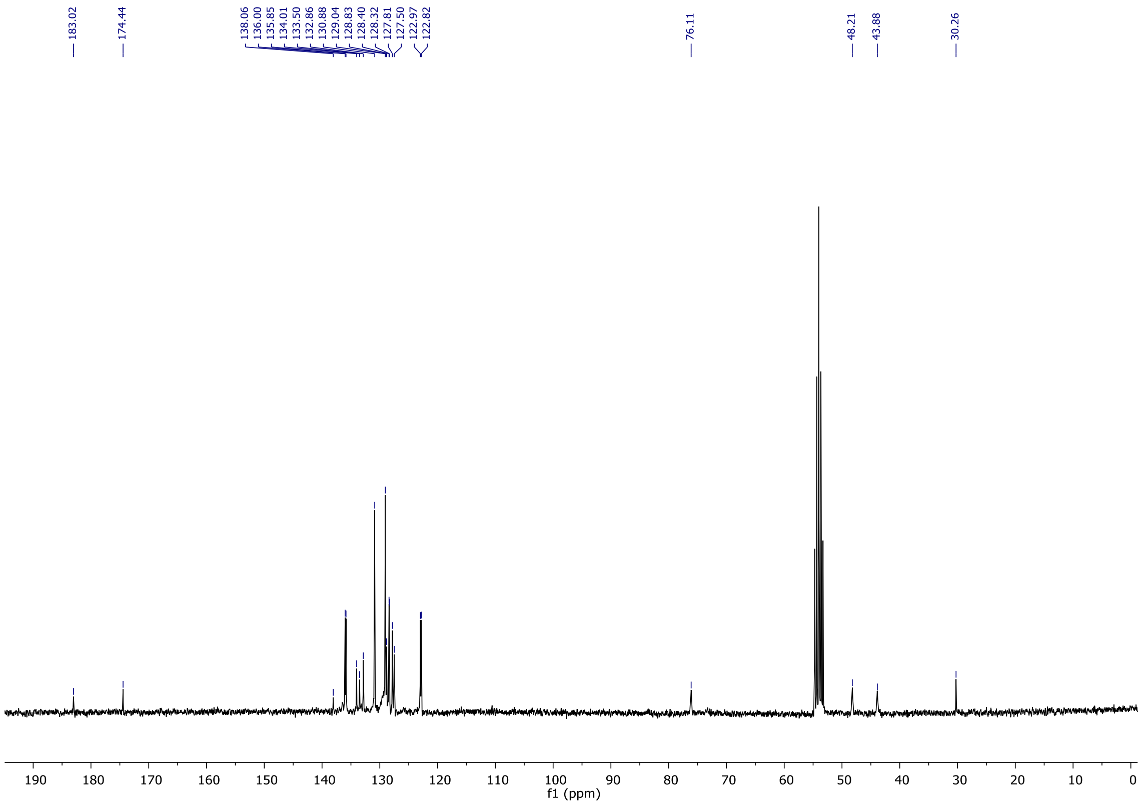

5. ^{1}H and ^{13}C NMR spectra.

^{1}H NMR (300 MHz, $CDCl_3$) of **2a**:

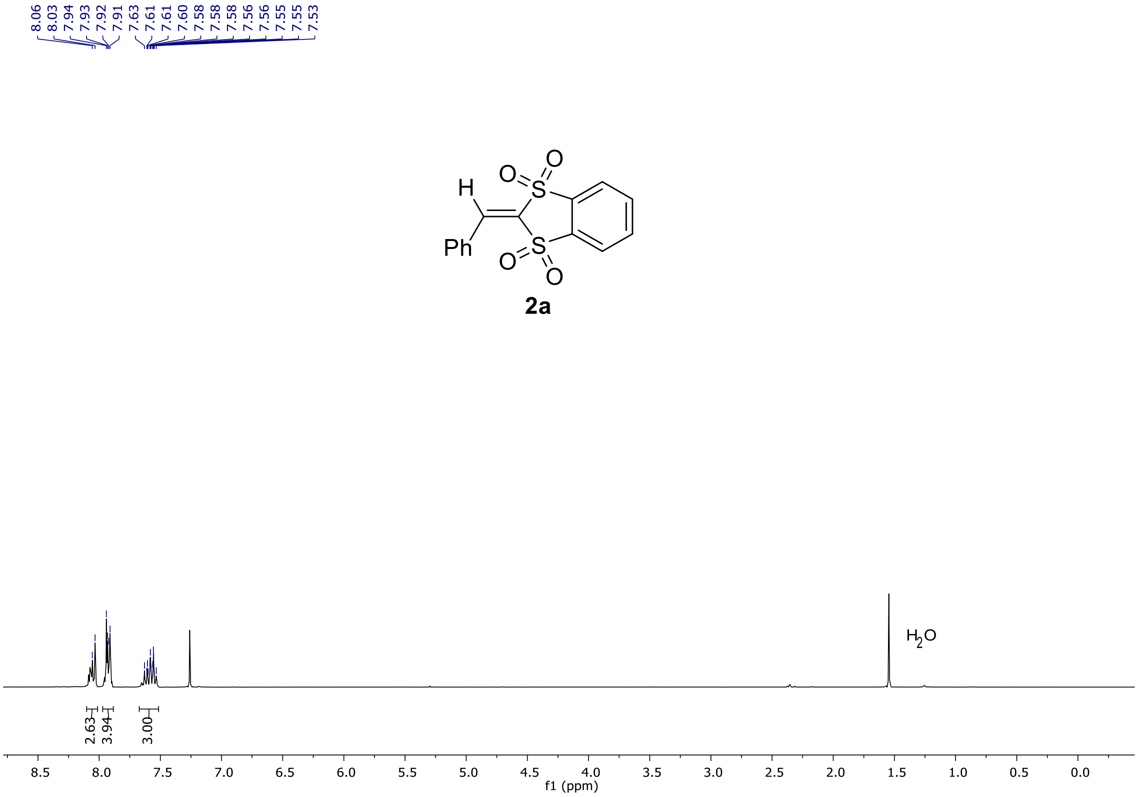

^{13}C{^{1}H} NMR (75 MHz, $CDCl_3$) of **2a**:

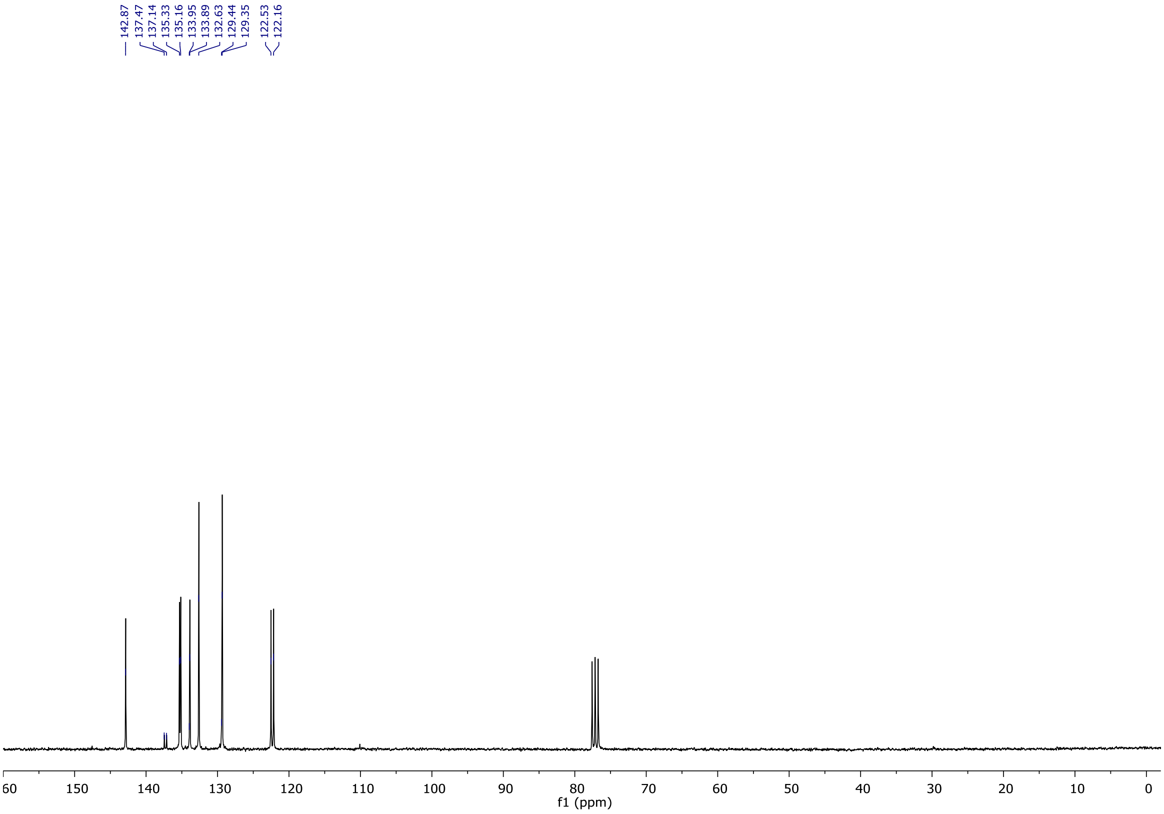

^{1}H NMR (300 MHz, $CDCl_3$) of **2b**:

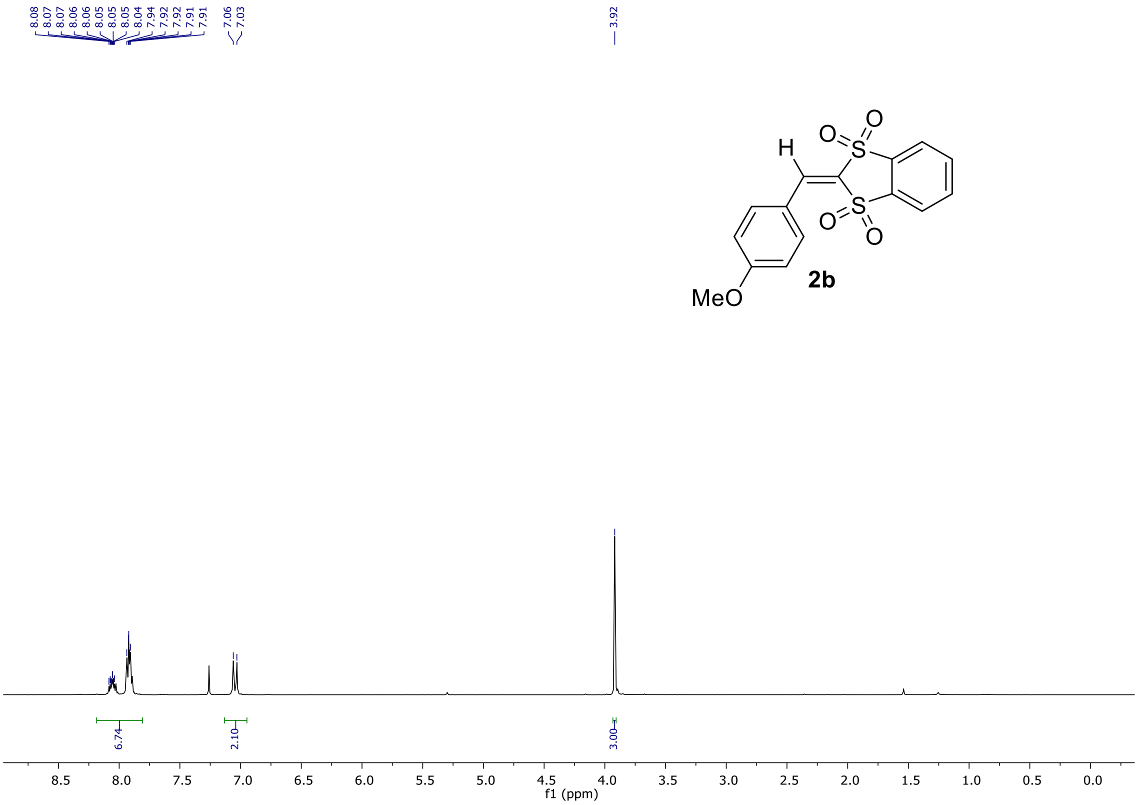

$^{13}C\{^{1}H\}$ NMR (75 MHz, $CDCl_3$) of **2b**:

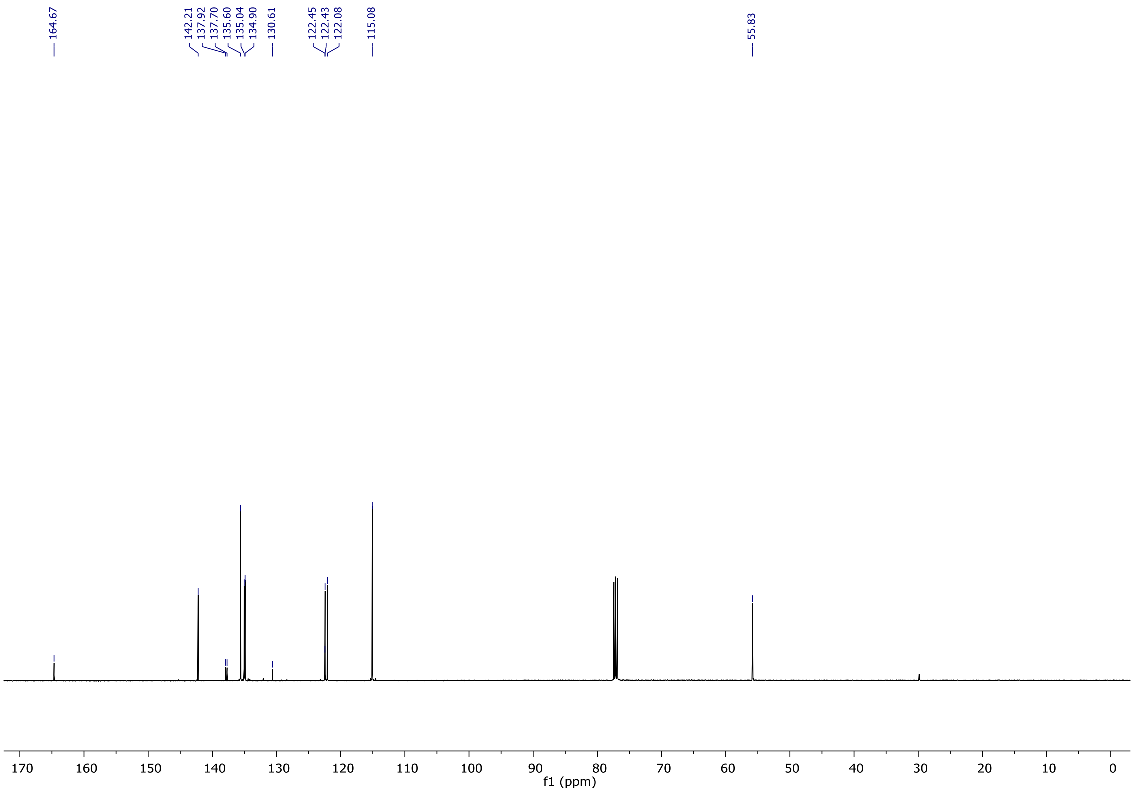

^{1}H NMR (300 MHz, $CDCl_3$) of **2c**:

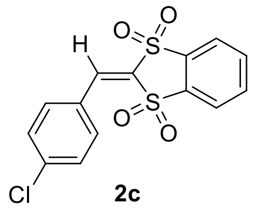

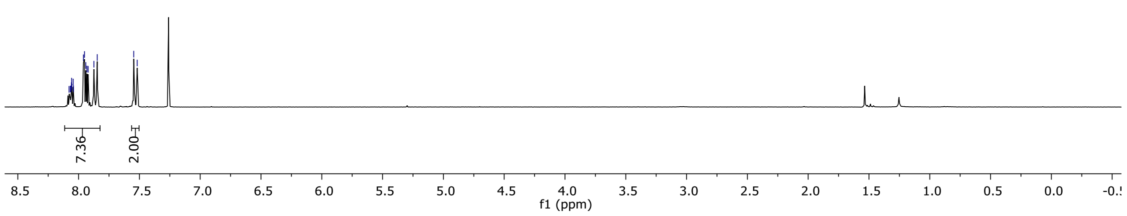

$^{13}C\{^{1}H\}$ NMR (75 MHz, $CDCl_3$) of **2c**:

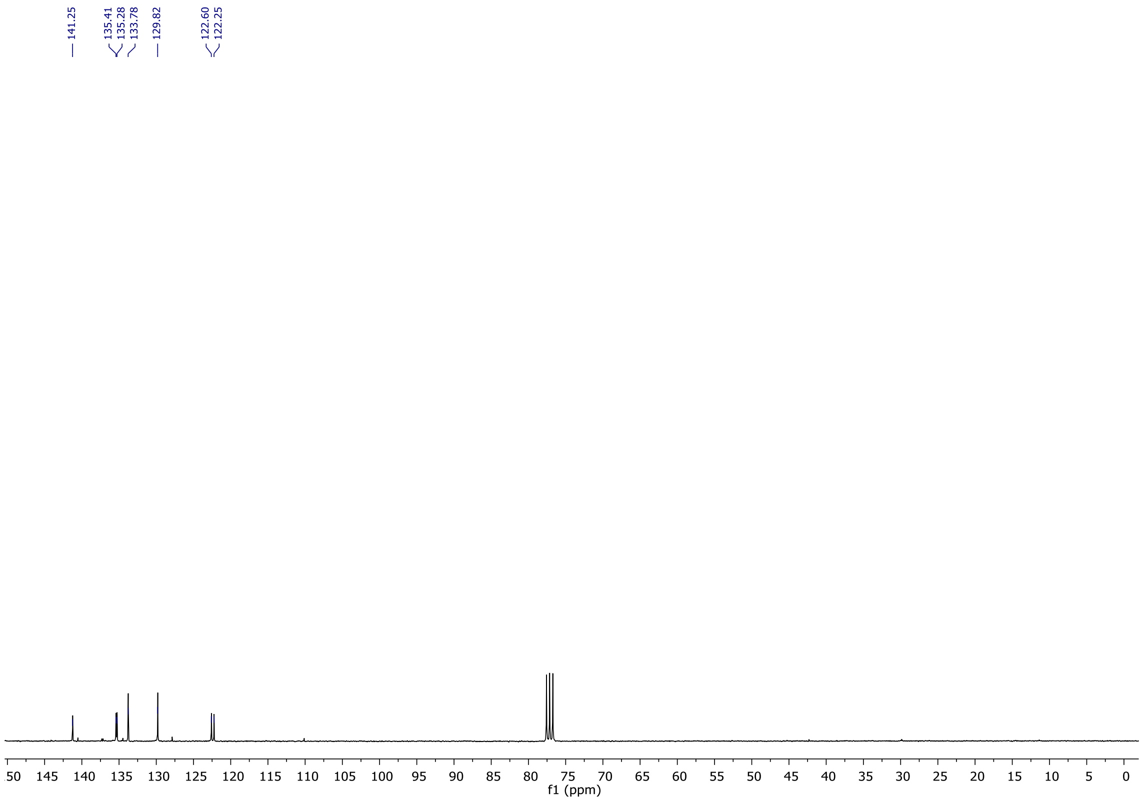

^{1}H NMR (300 MHz, $CDCl_3$) of **2d**:

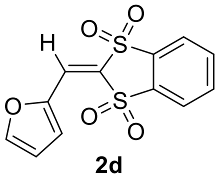

2d

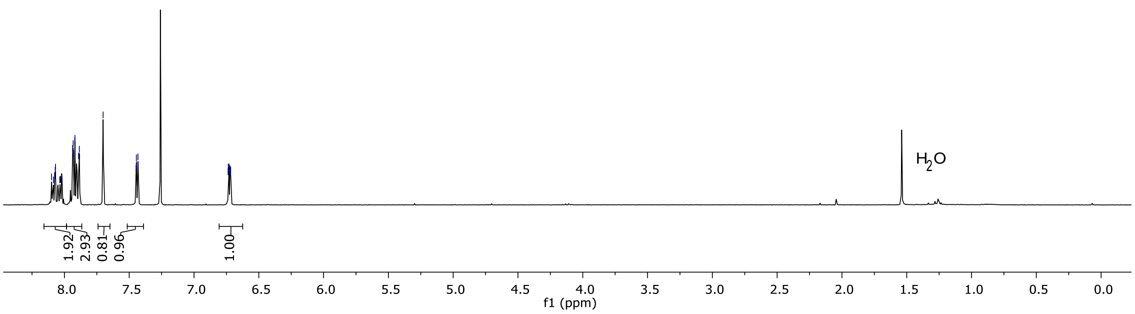

$^{13}C\{^{1}H\}$ NMR (75 MHz, $CDCl_3$) of **2d**:

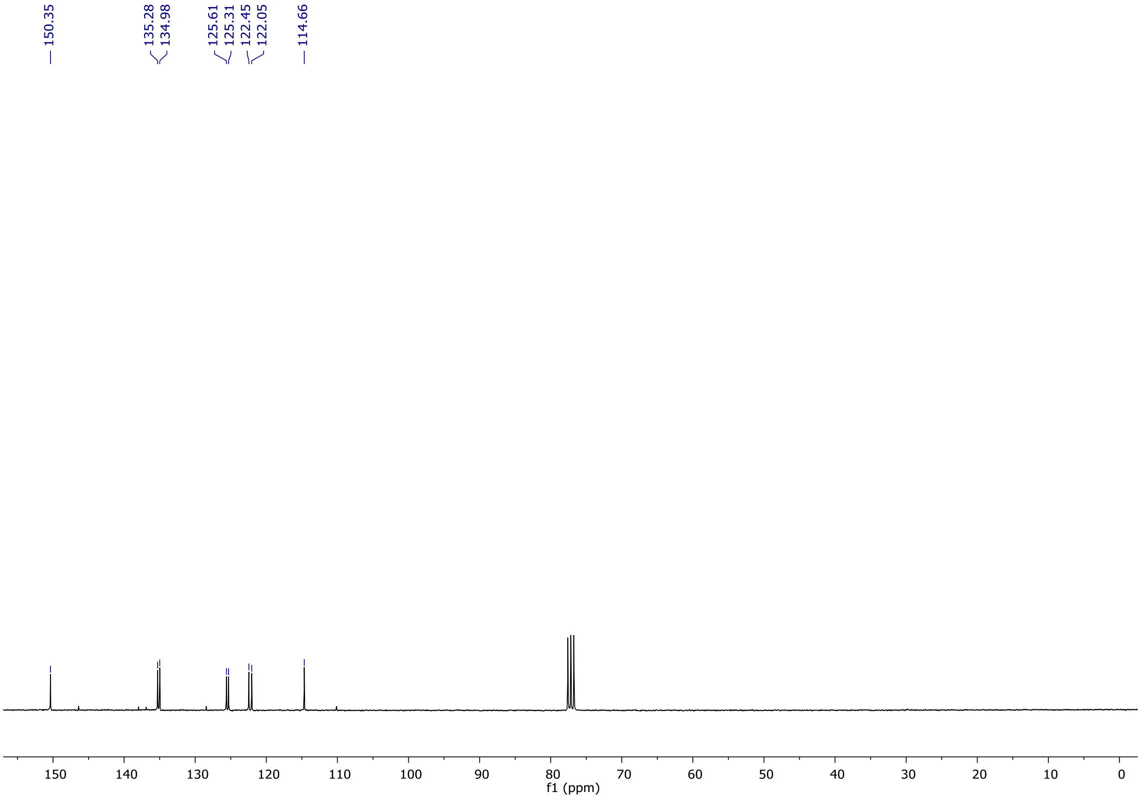

^{1}H NMR (300 MHz, $CDCl_3$) of **2e**:

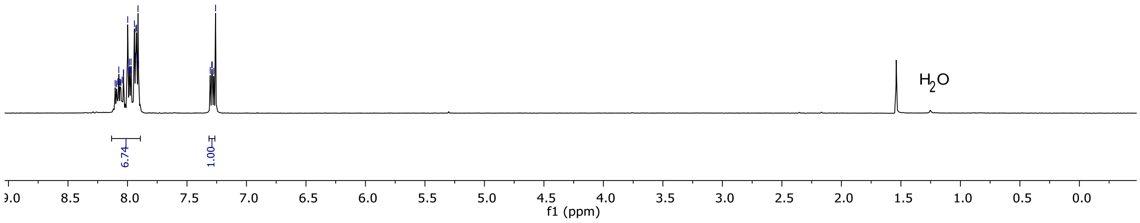

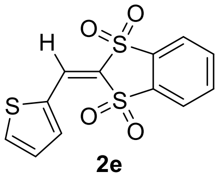

2e

^{13}C{^{1}H} NMR (75 MHz, $CDCl_3$) of **2e**:

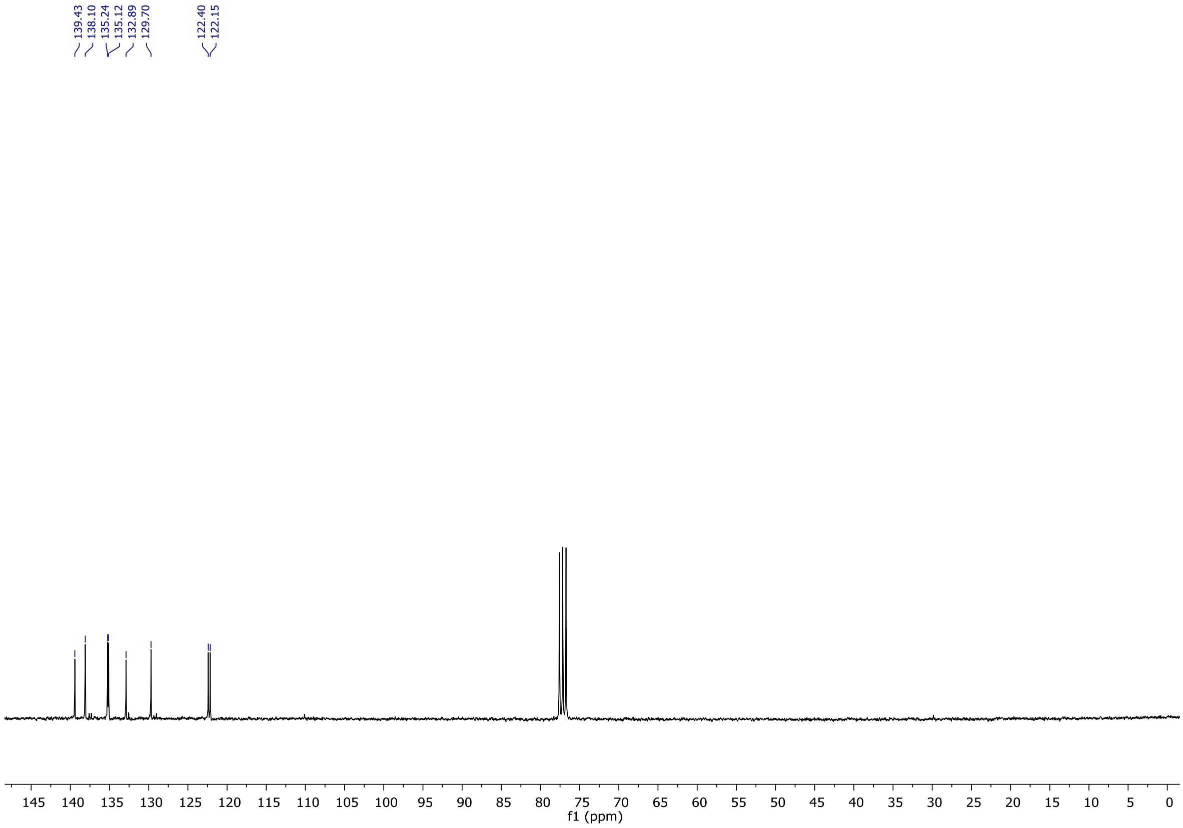

^{1}H NMR (300 MHz, $CDCl_3$) of **2f**:

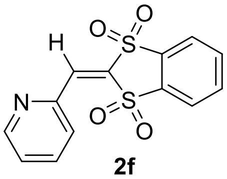

2f

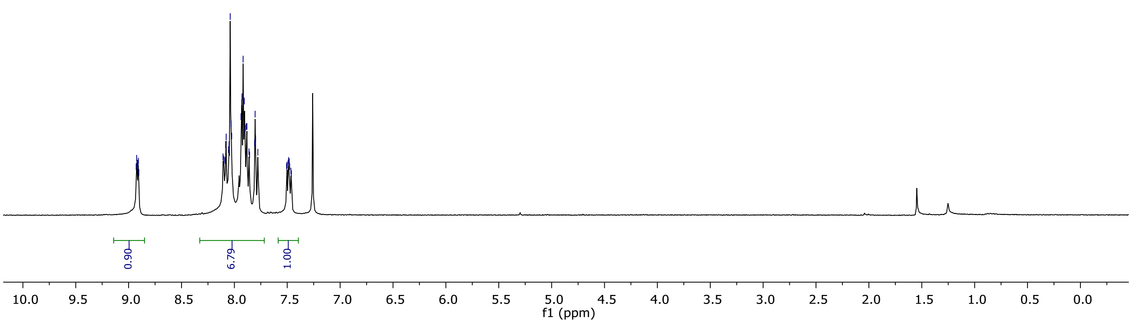

^{13}C{^{1}H} NMR (126 MHz, $CDCl_3$) of **2f**:

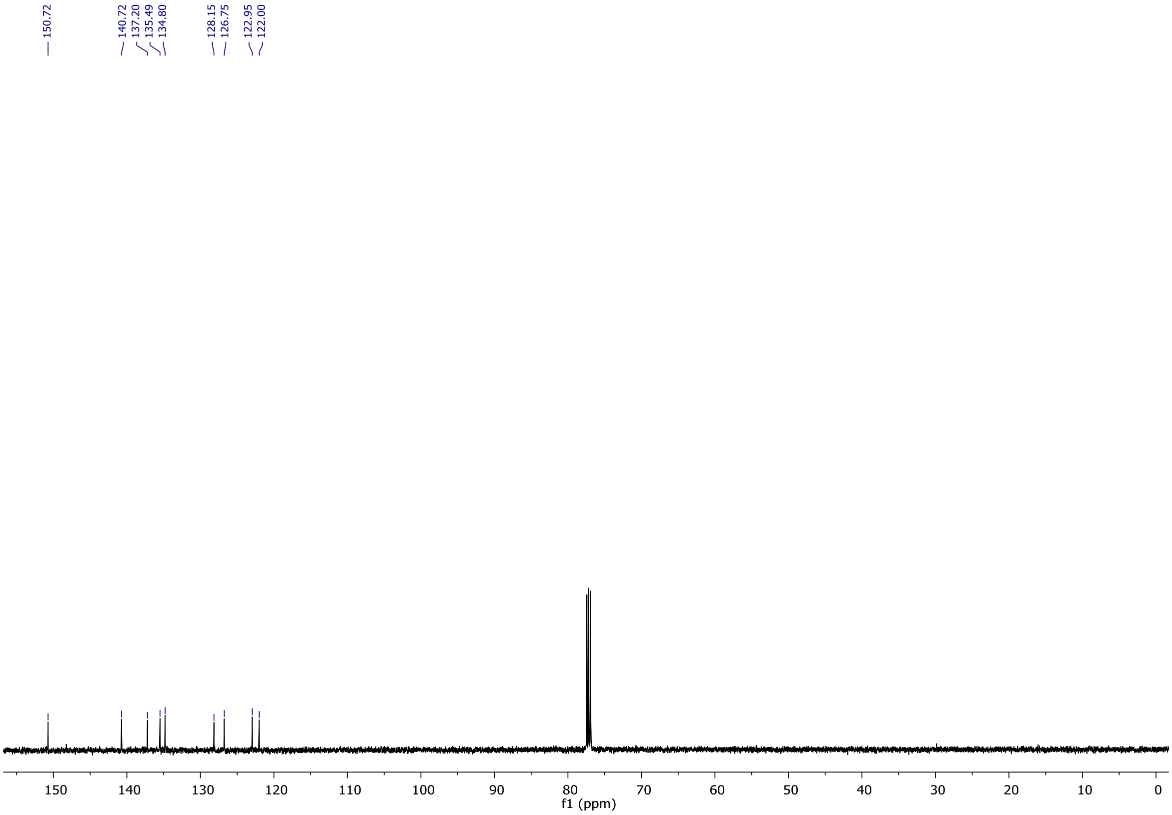

^{1}H NMR (300 MHz, CD_2Cl_2) of **2g**:

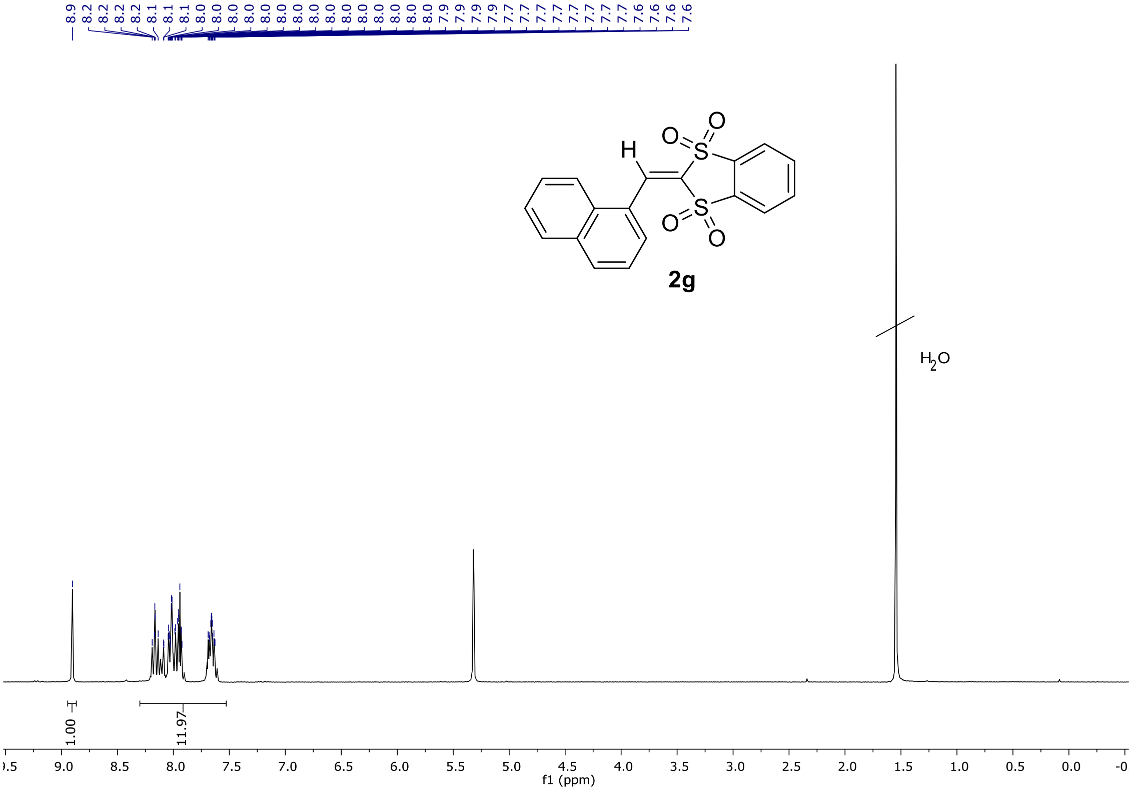

^{13}C{^{1}H} NMR (75 MHz, CD_2Cl_2) of **2g**:

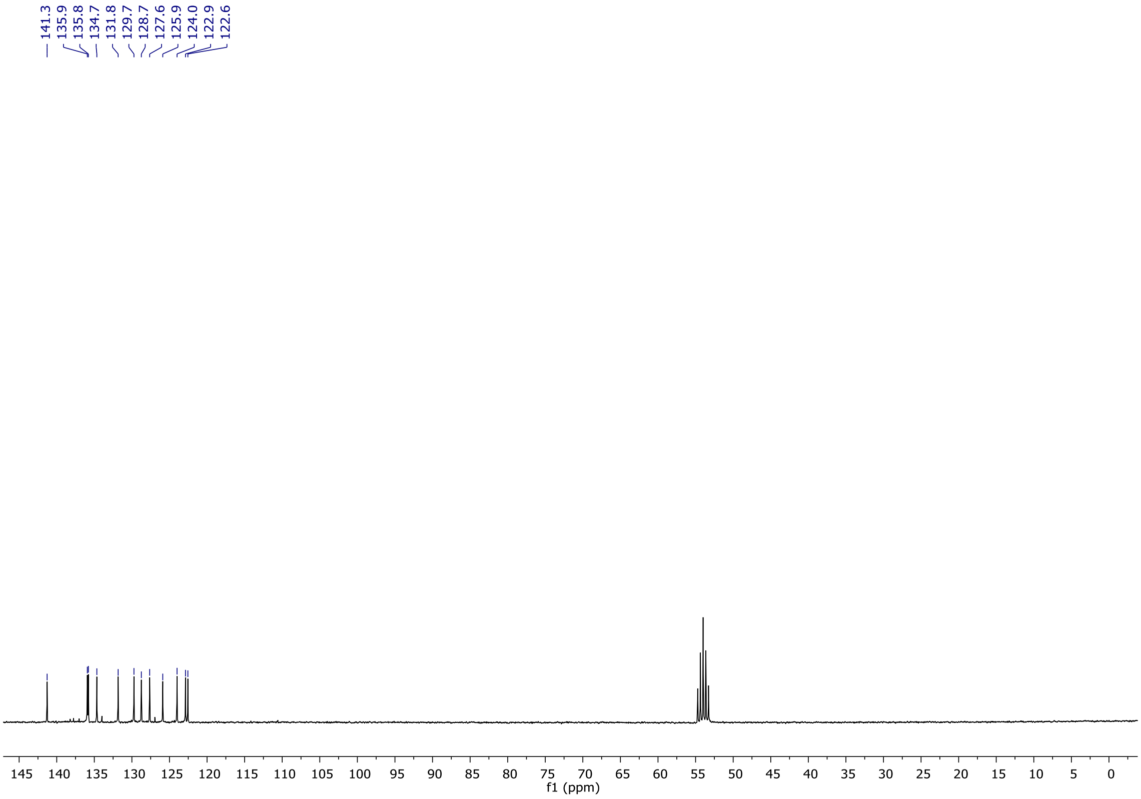

^{1}H NMR (300 MHz, CD_2Cl_2) of **2h**:

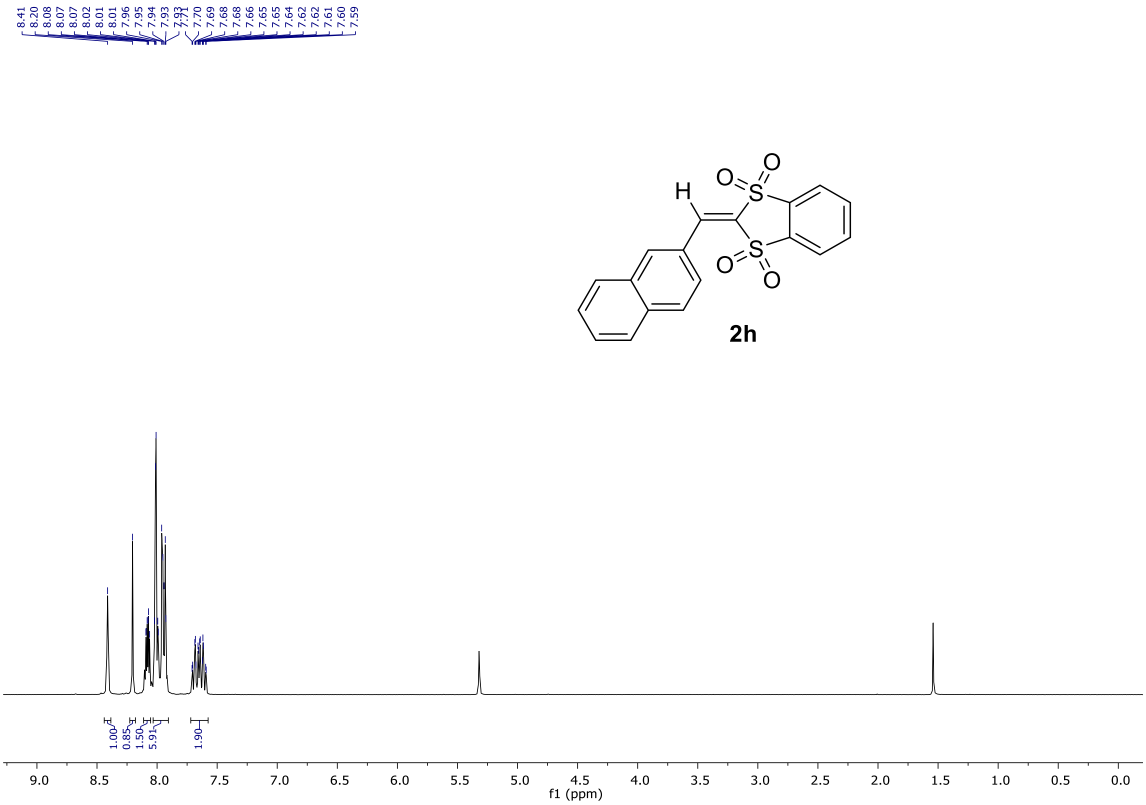

^{13}C{^{1}H} NMR (75 MHz, CD_2Cl_2) of **2h**:

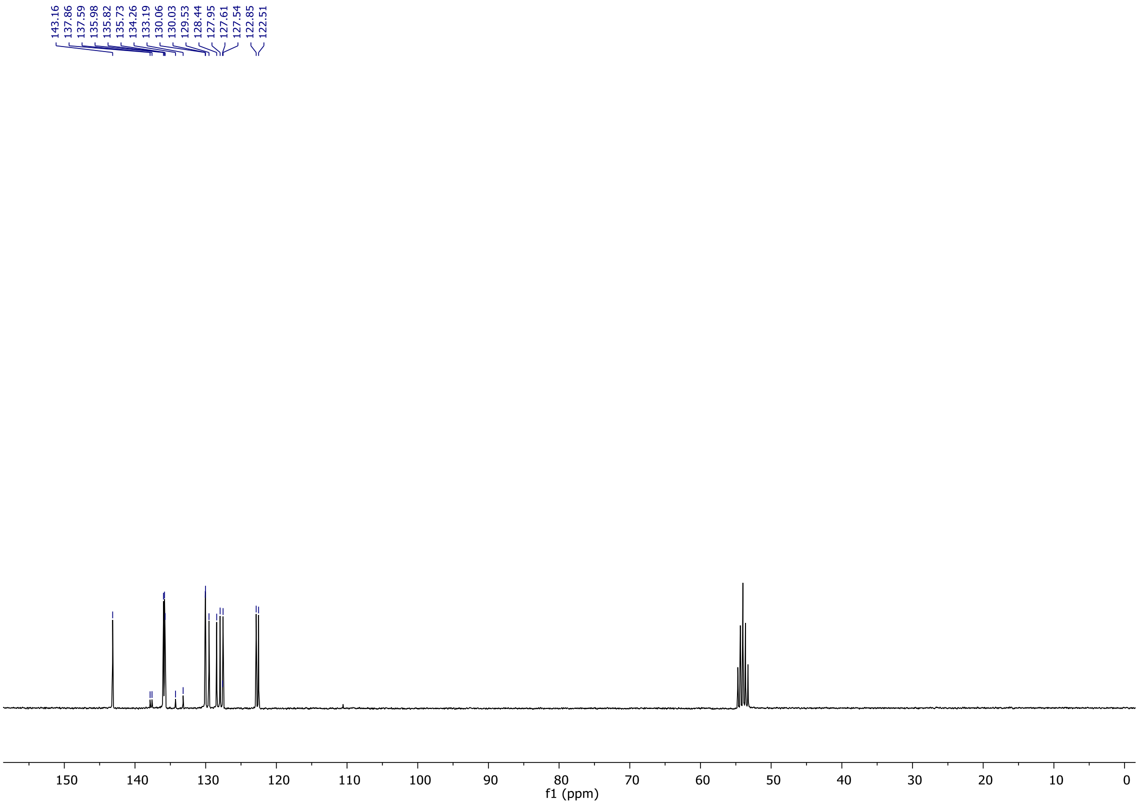

^{1}H NMR (300 MHz, $CDCl_3$) of **5-Allyl-1-benzoyl-2-thioxoimidazolidin-4-one**:

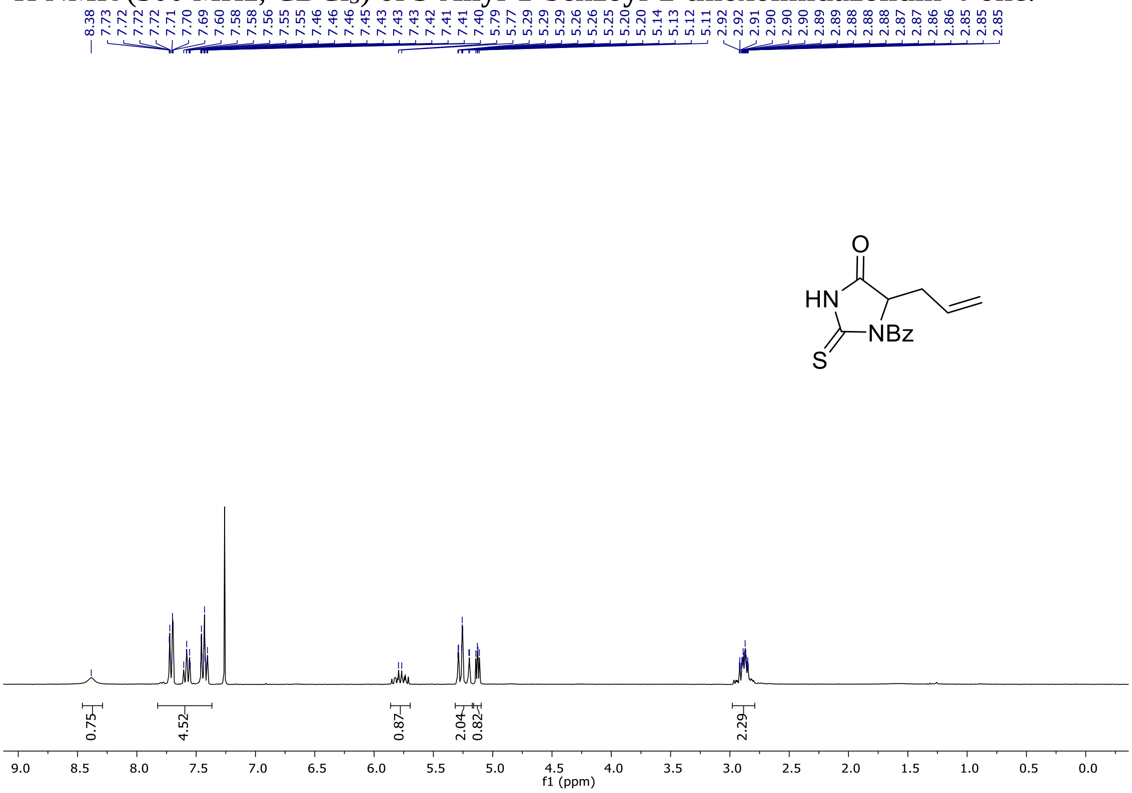

$^{13}C\{^{1}H\}$ NMR (75 MHz, $CDCl_3$) of **5-Allyl-1-benzoyl-2-thioxoimidazolidin-4-one**:

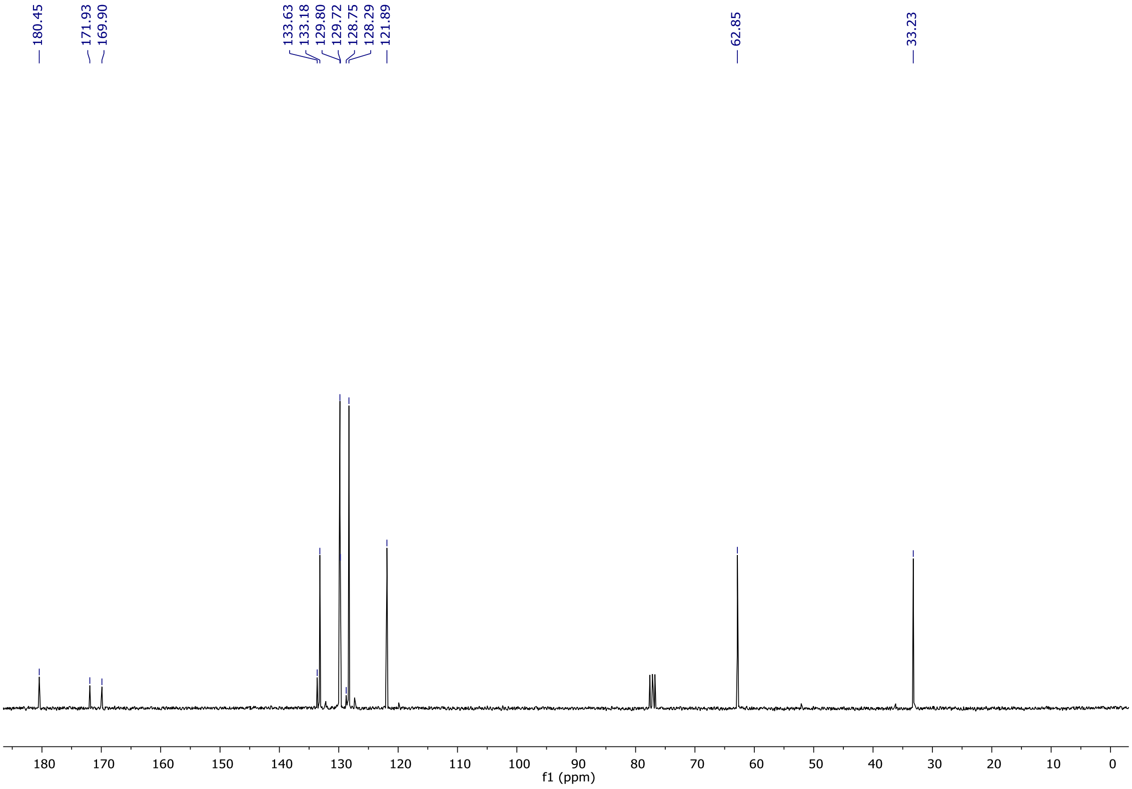

^{1}H NMR (300 MHz, $CDCl_3$) of **methyl 2-(3-benzoyl-5-oxo-2-thioxoimidazolidin-4-yl)acetate**:

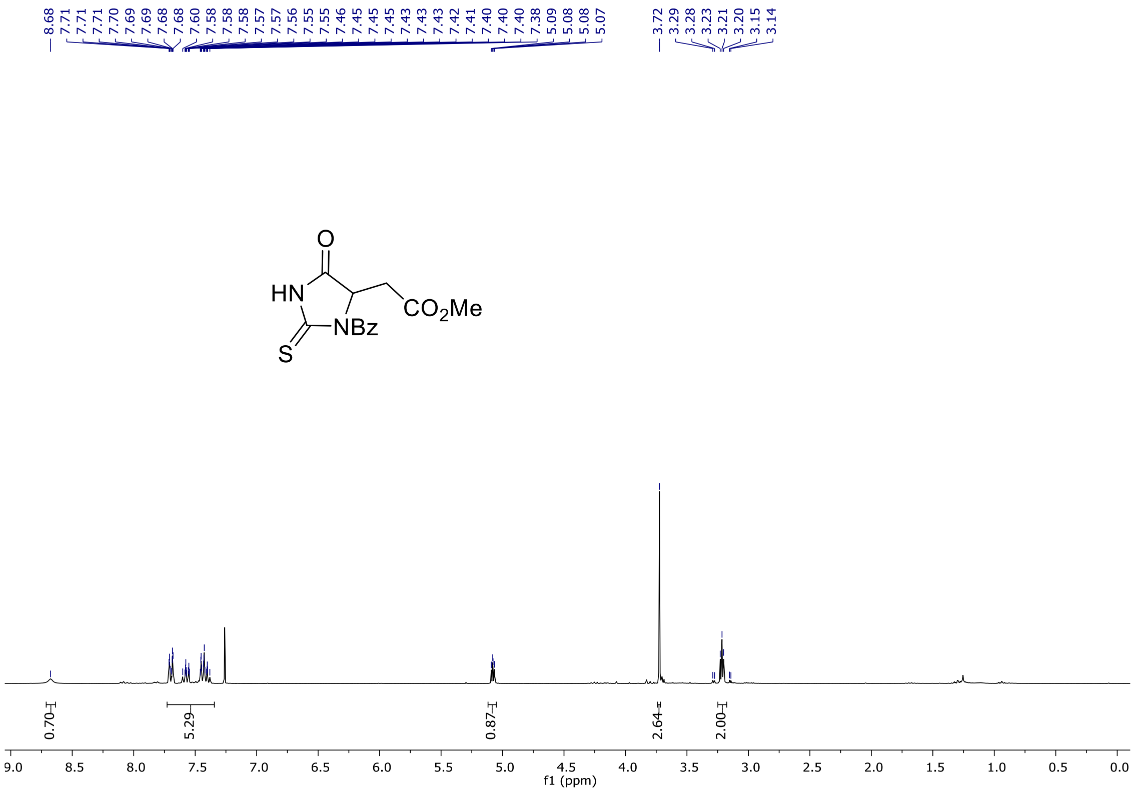

$^{13}C\{^{1}H\}$ NMR (75 MHz, $CDCl_3$) of **methyl 2-(3-benzoyl-5-oxo-2-thioxoimidazolidin-4-yl)acetate:**

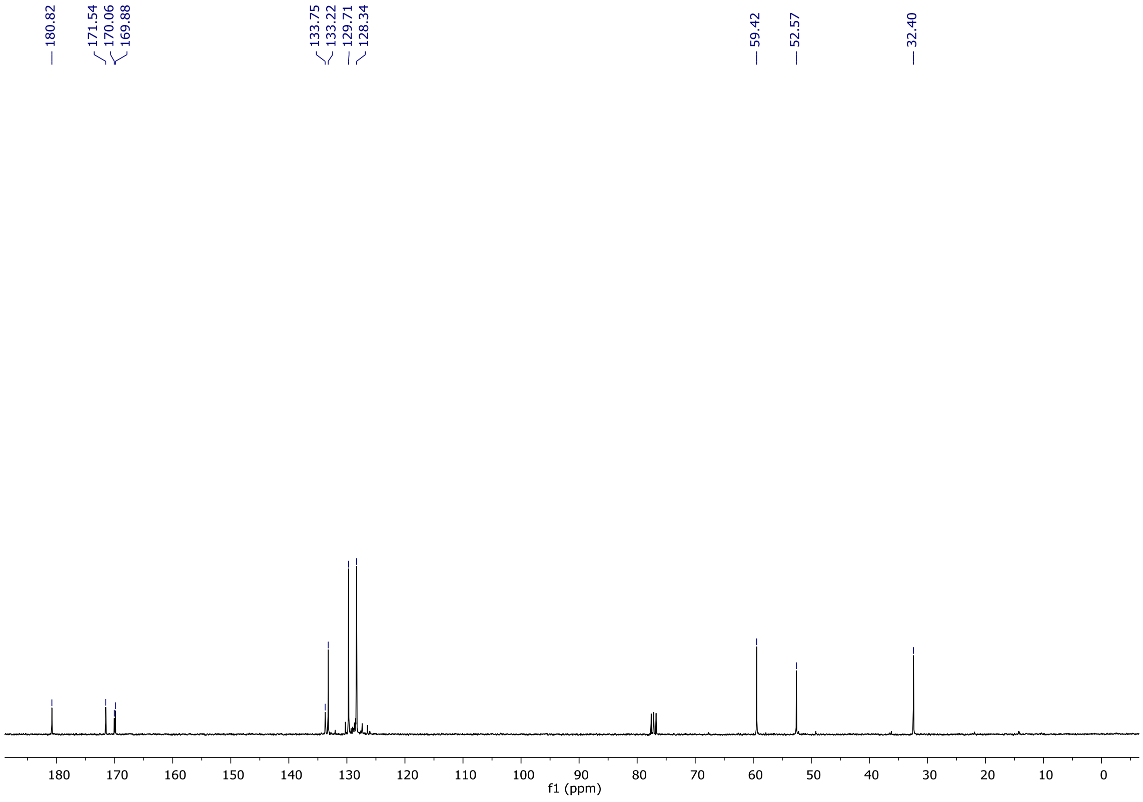

^{1}H NMR (300 MHz, $CDCl_3$) of **1-Benzoyl-2-thioxoimidazolidin-4-one**:

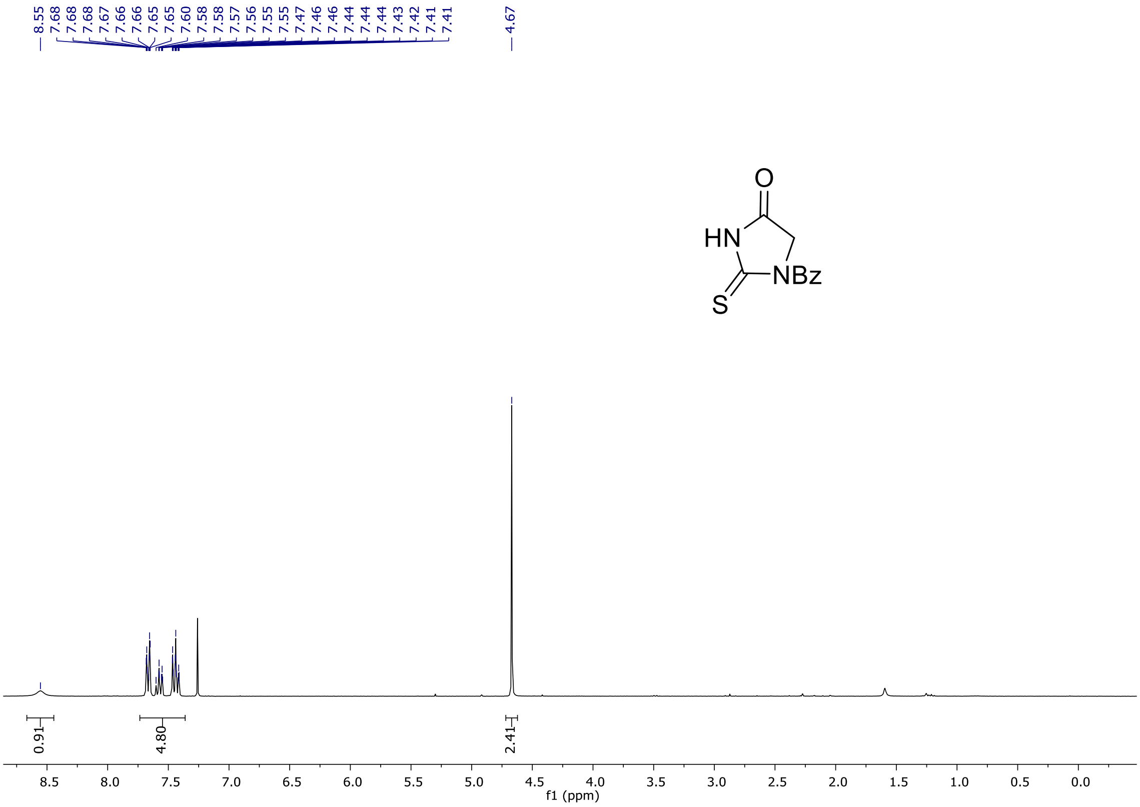

$^{13}C\{^{1}H\}$ NMR (75 MHz, $CDCl_3$) of **1-Benzoyl-2-thioxoimidazolidin-4-one**:

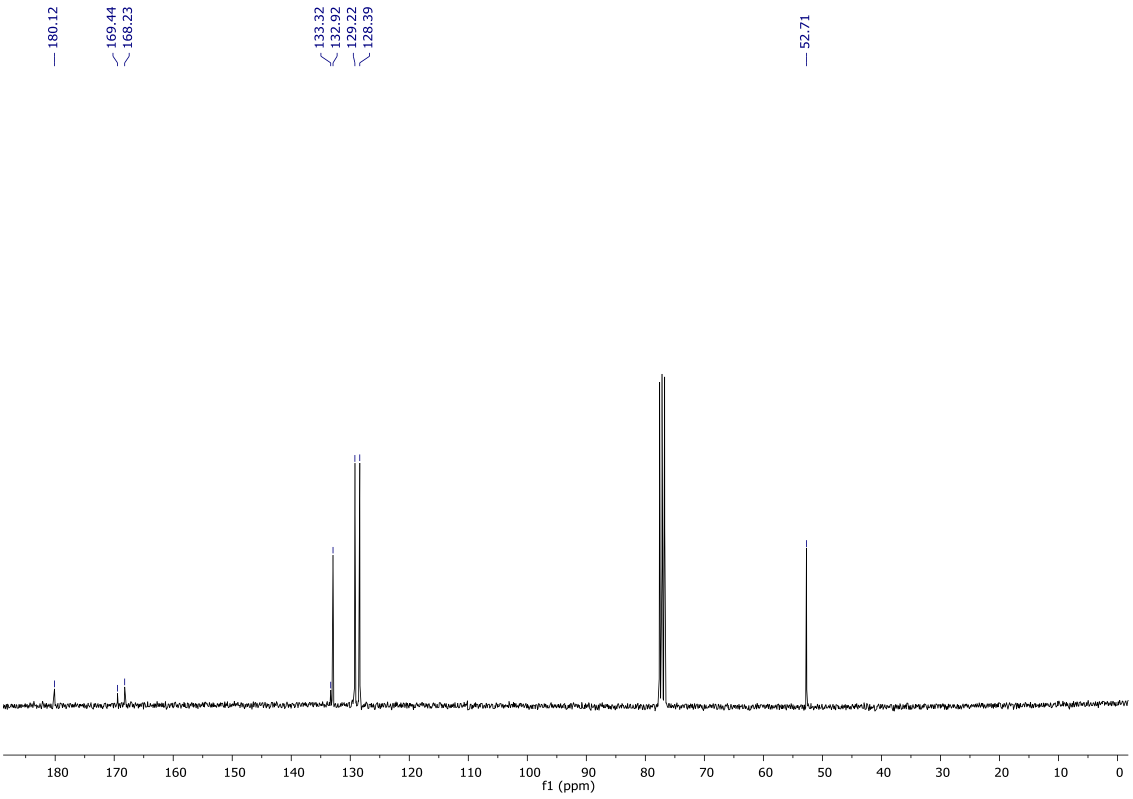

^{1}H NMR (300 MHz, $CDCl_3$) of **5-Benzyl-1-(2-phenylacetyl)-2-thioxoimidazolidin-4-one**:

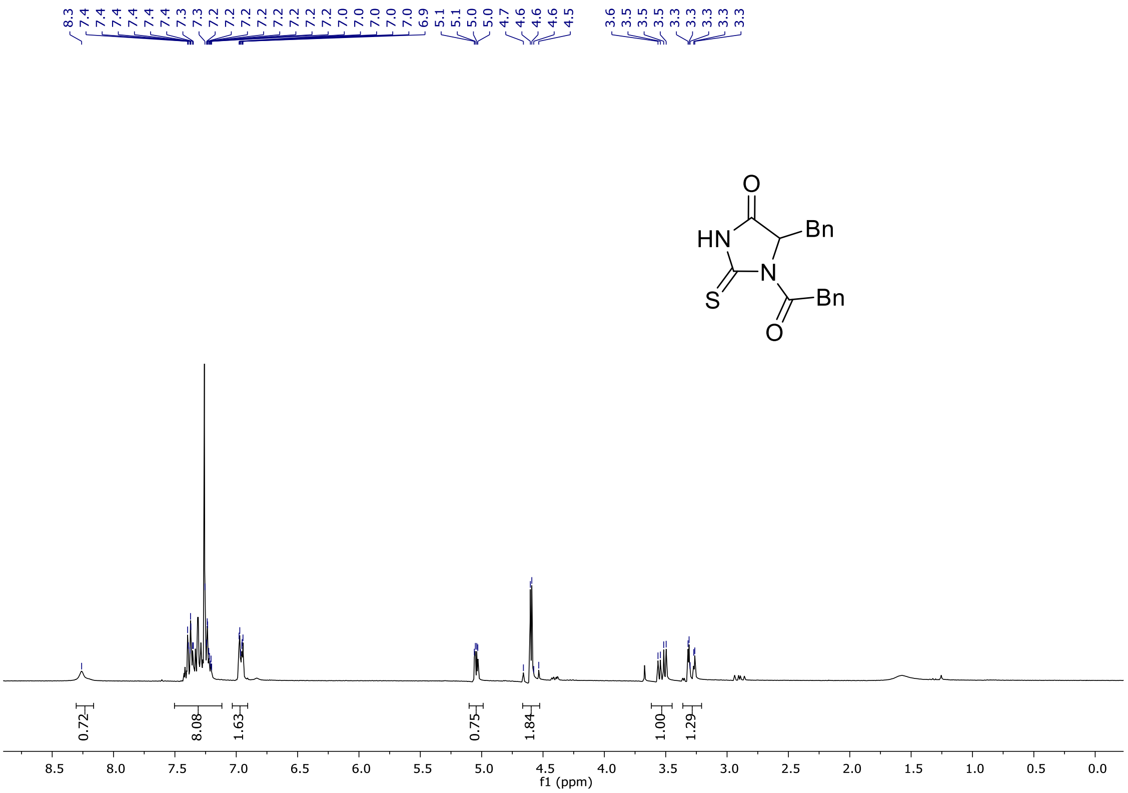

^{13}C{^{1}H} NMR (75 MHz, $CDCl_3$) of **5-Benzyl-1-(2-phenylacetyl)-2-thioxoimidazolidin-4-one**:

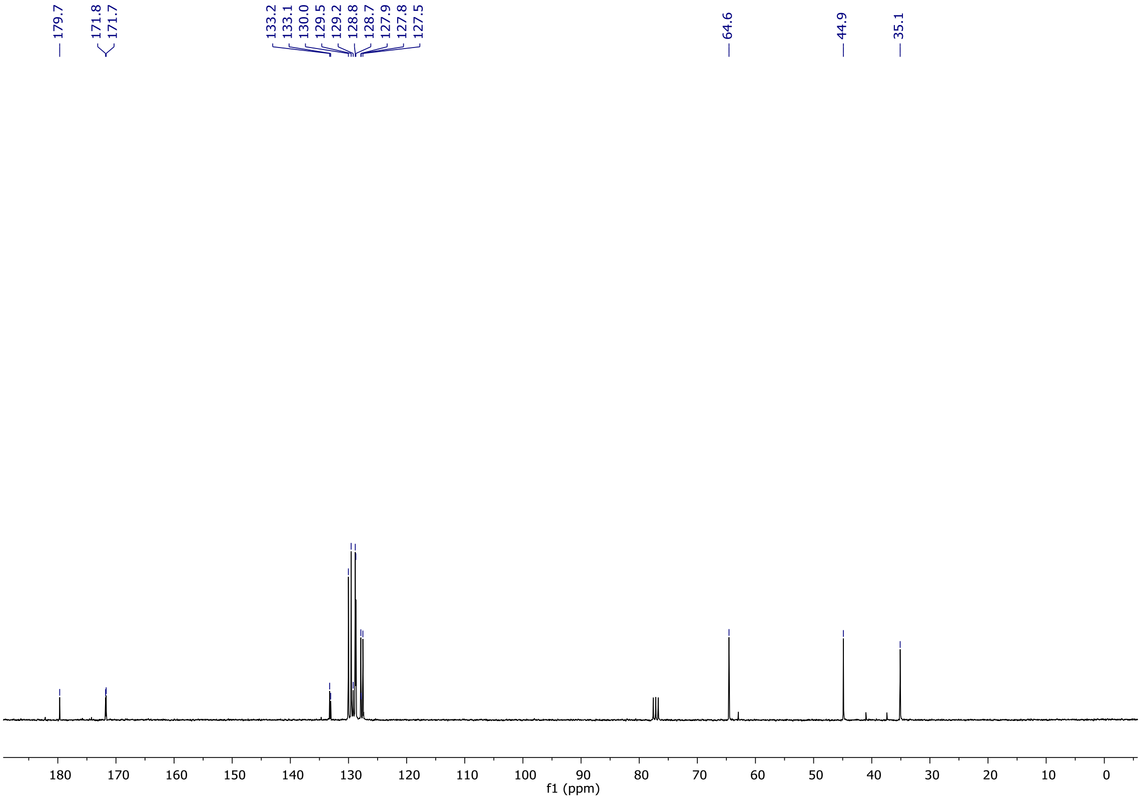

^{1}H NMR (300 MHz, $CDCl_3$) of **3e**:

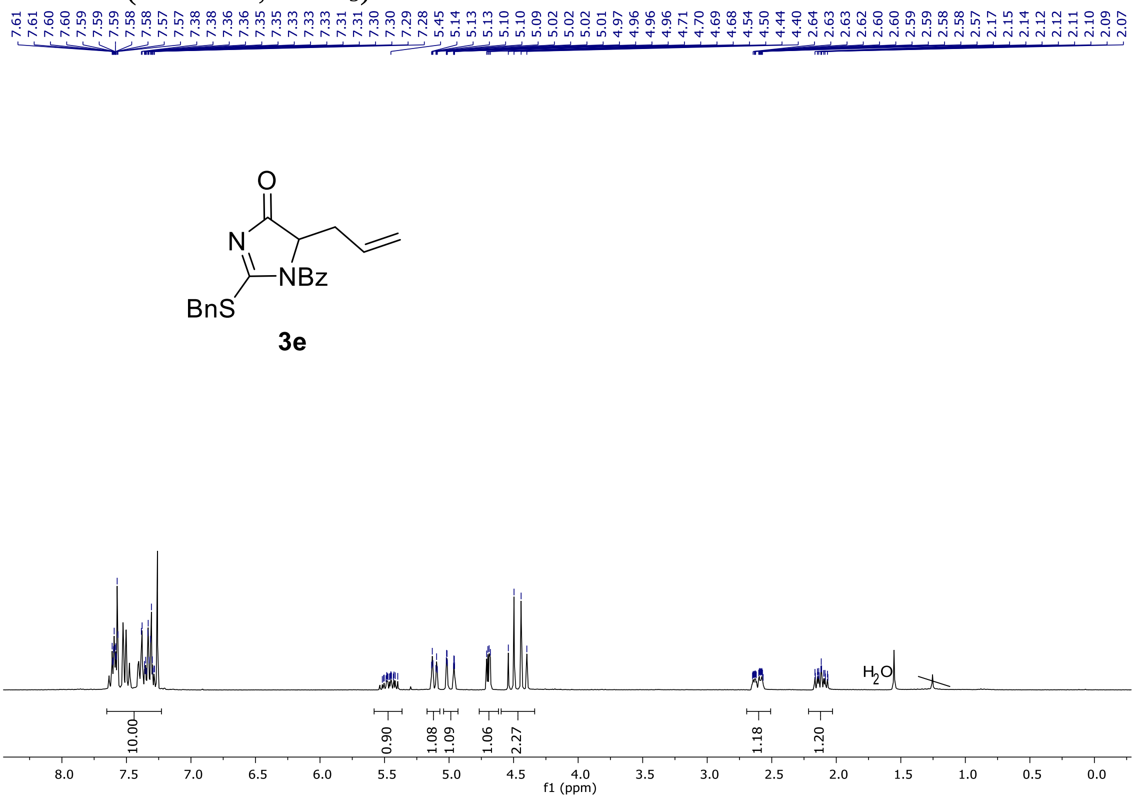

^{13}C{^{1}H} NMR (75 MHz, $CDCl_3$) of **3e**:

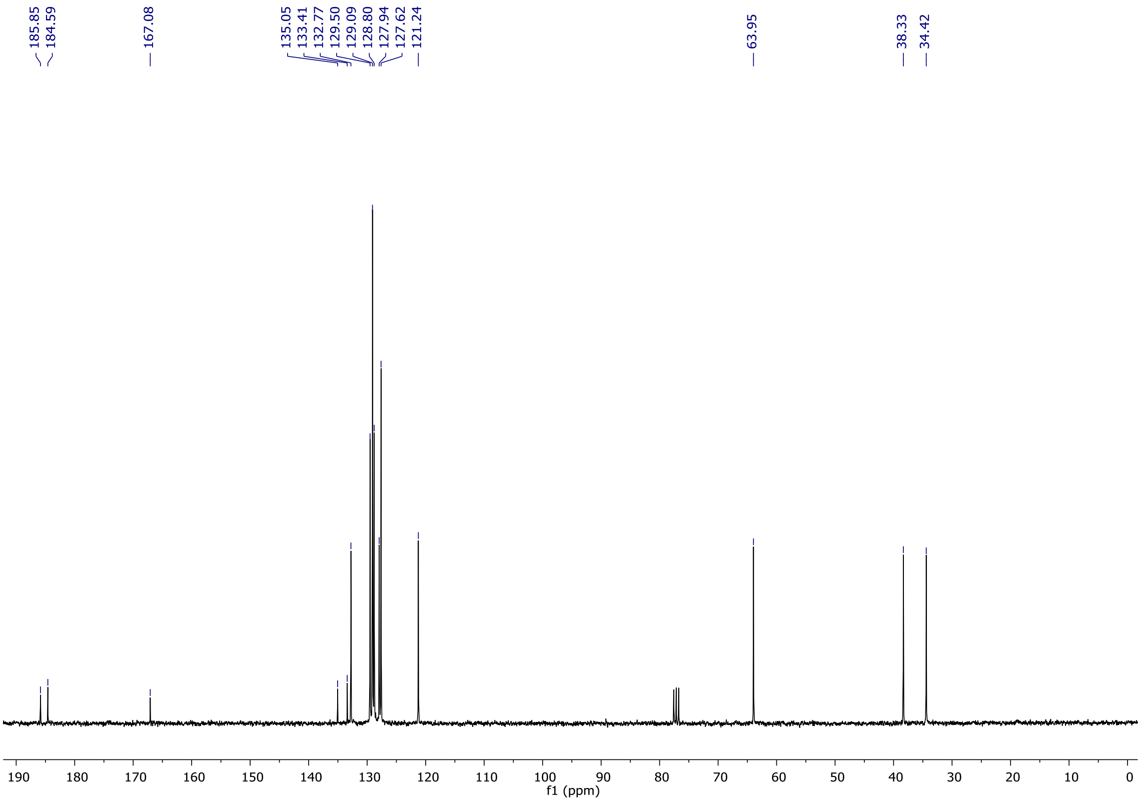

^{1}H NMR (300 MHz, $CDCl_3$) of **3g**:

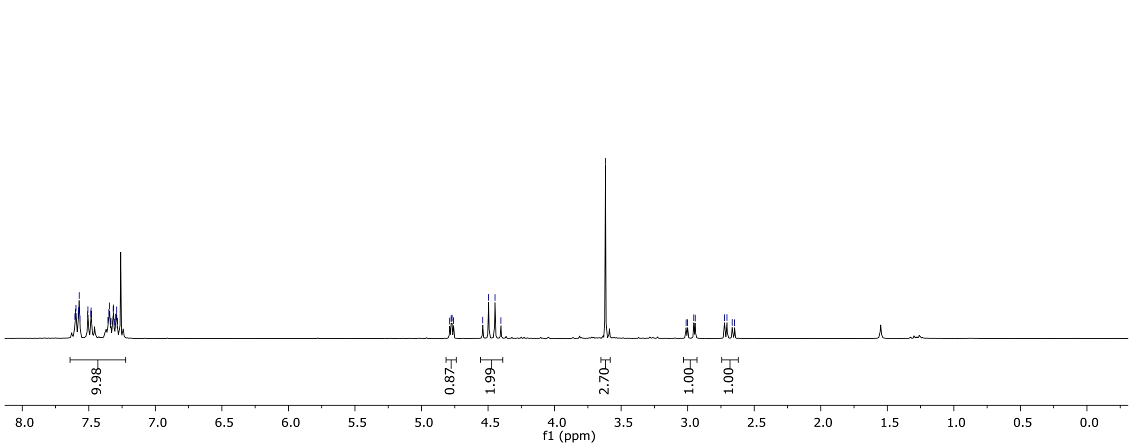

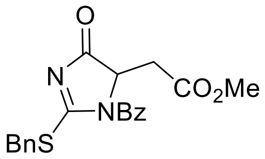

3g

$^{13}C\{^{1}H\}$ NMR (75 MHz, $CDCl_3$) of **3g**:

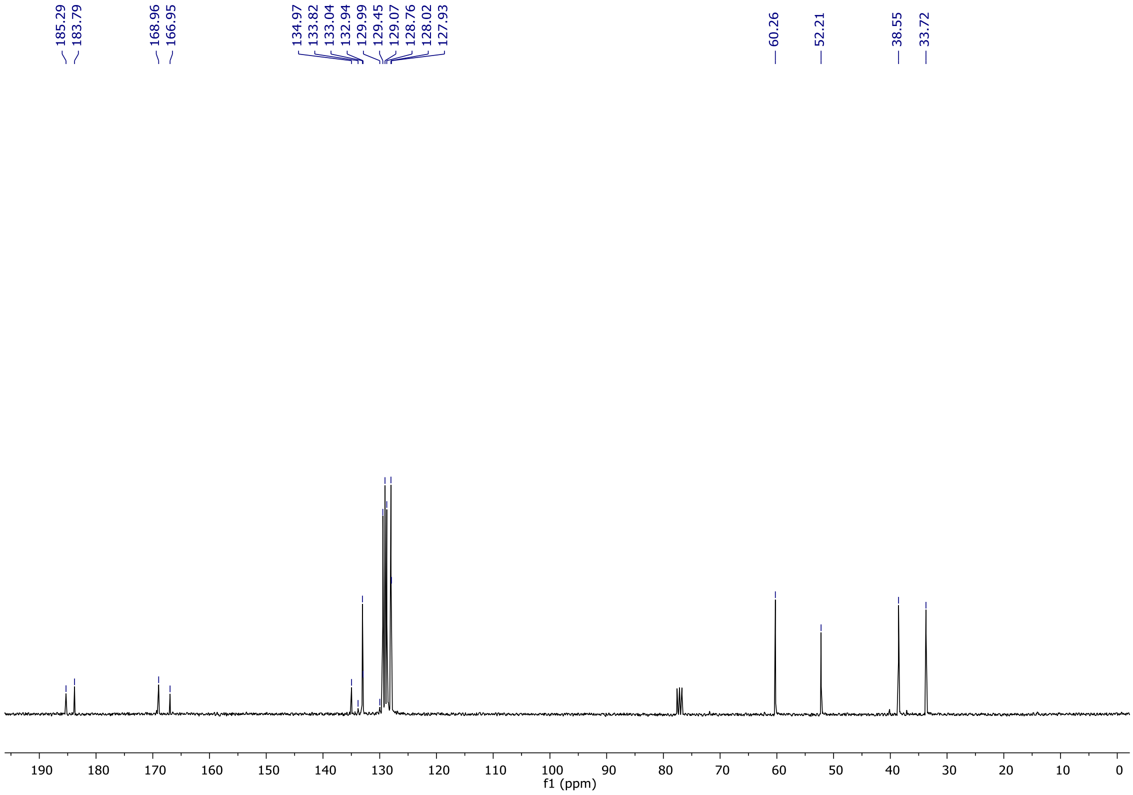

^{1}H NMR (300 MHz, $CDCl_3$) of **3h/3h'**:

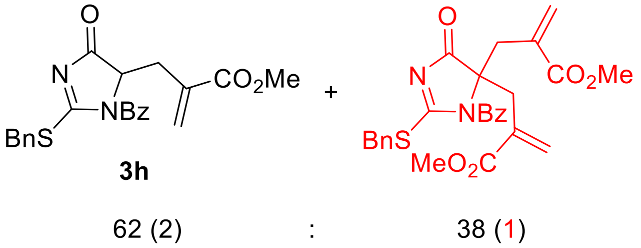

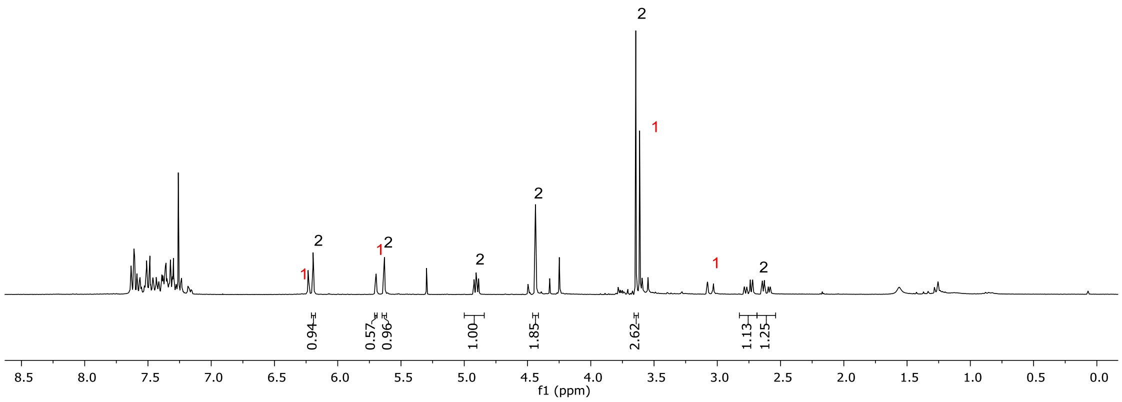

^{1}H NMR (300 MHz, $CDCl_3$) of **6a**:

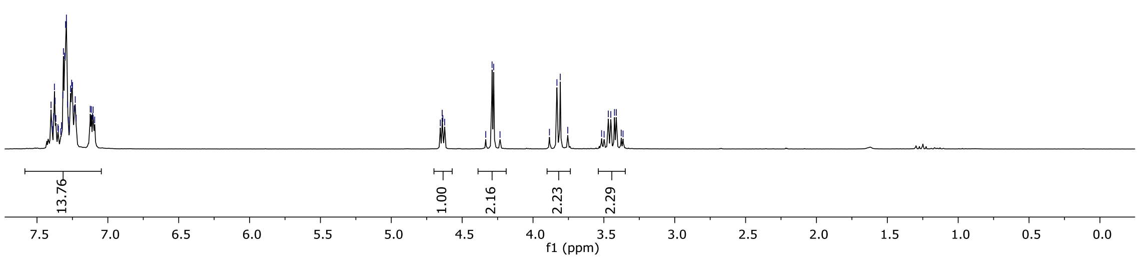

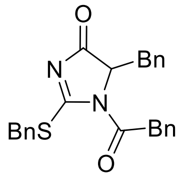

6a

$^{13}C\{^{1}H\}$ NMR (75 MHz, $CDCl_3$) of **6a**:

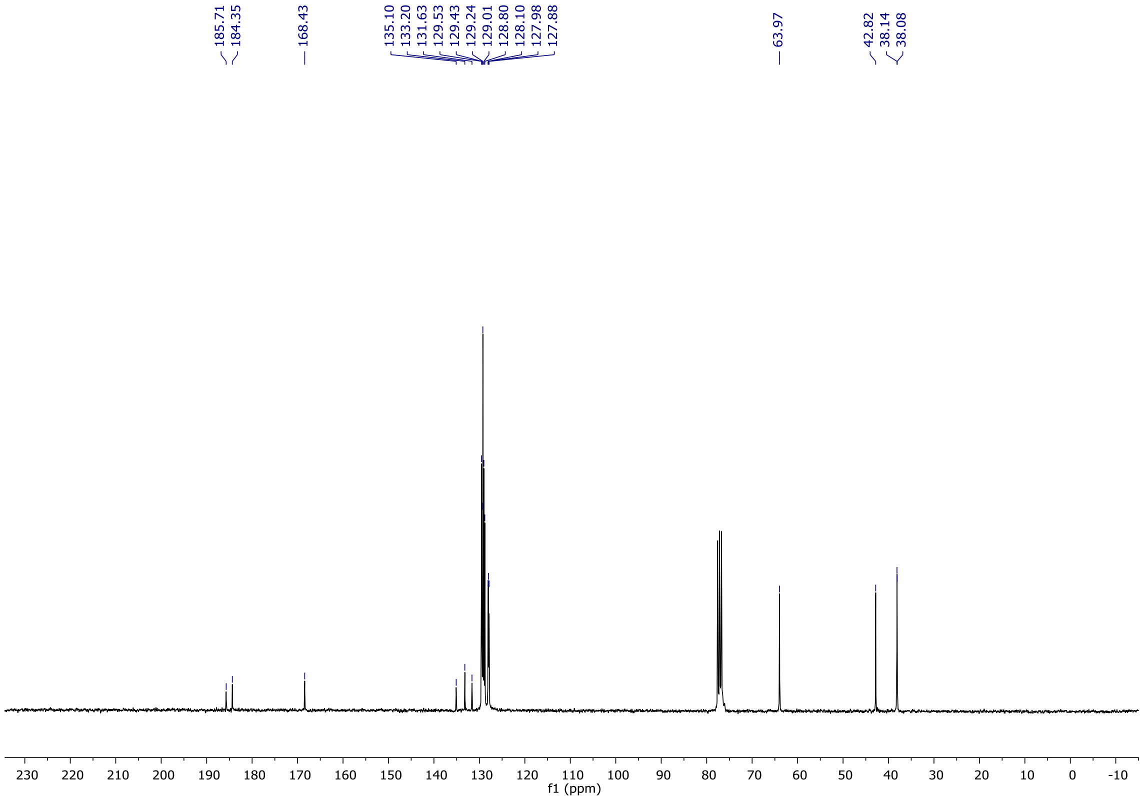

^{1}H NMR (300 MHz, $CDCl_3$) of **10a:**

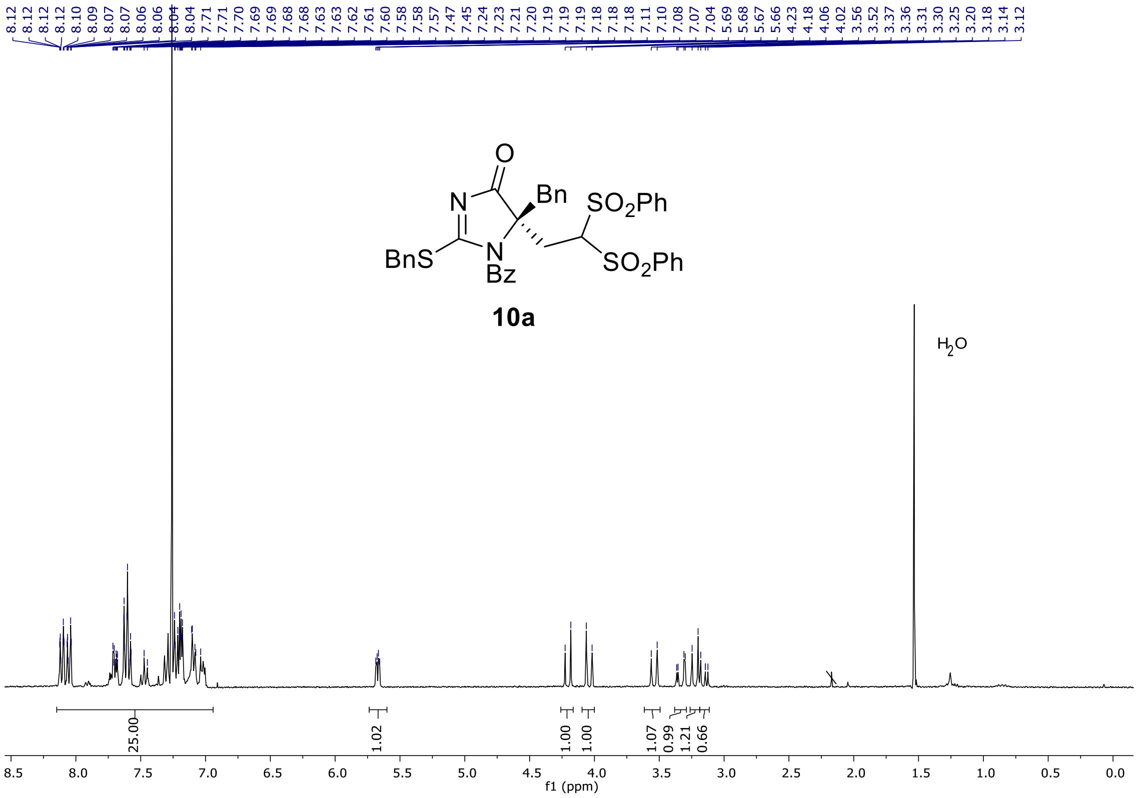

^{13}C{^{1}H} NMR (75 MHz, $CDCl_3$) of **10a**:

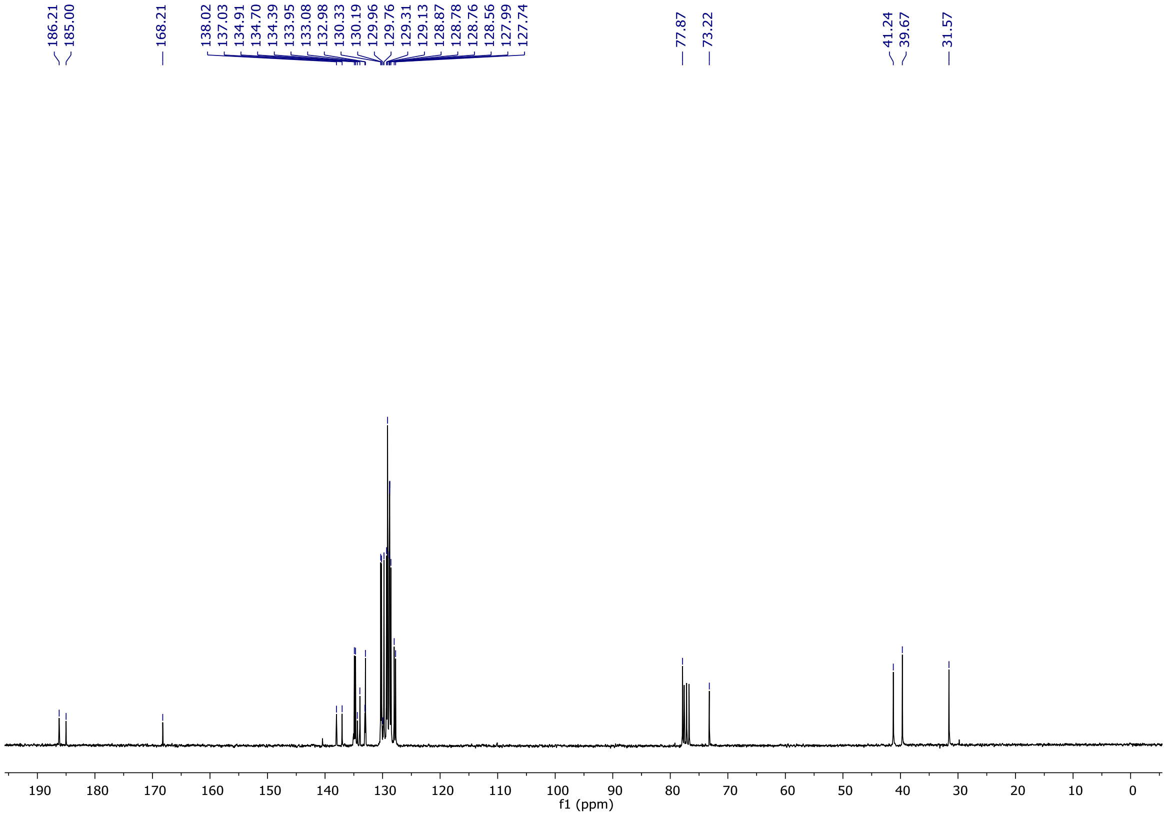

^{1}H NMR (300 MHz, $CDCl_3$) of **10b:**

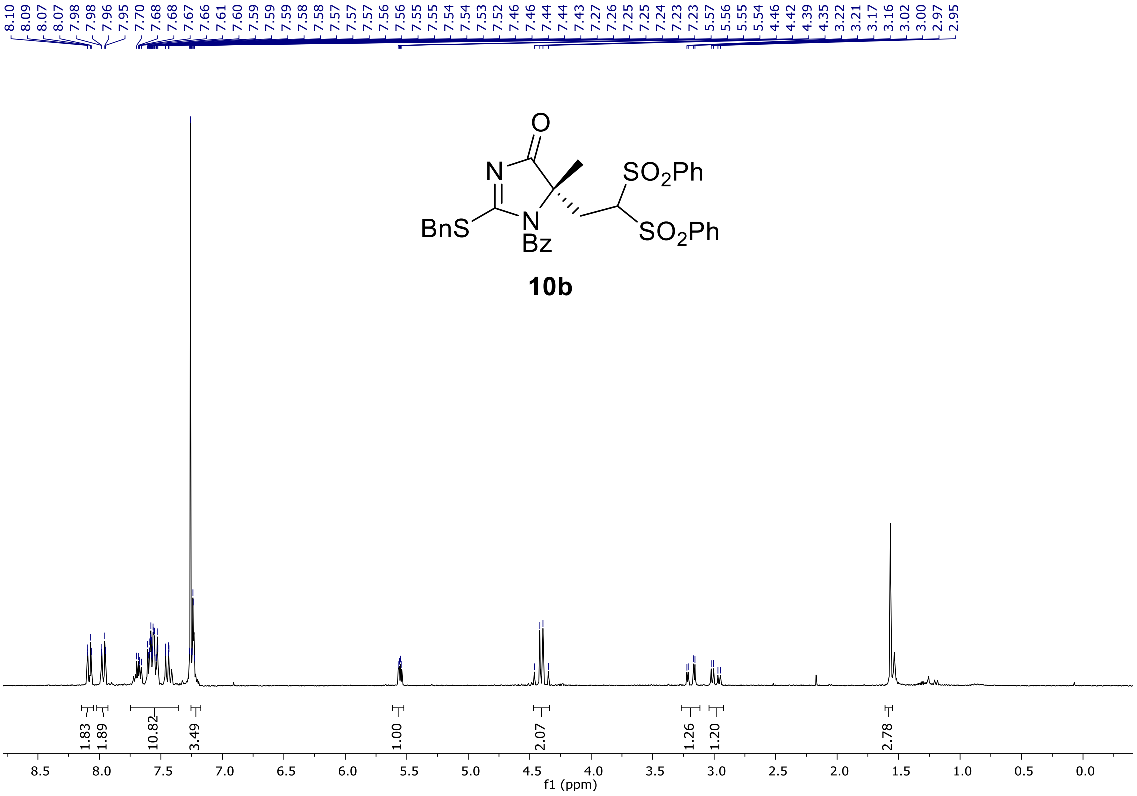

^{13}C{^{1}H} NMR (75 MHz, $CDCl_3$) of **10b**:

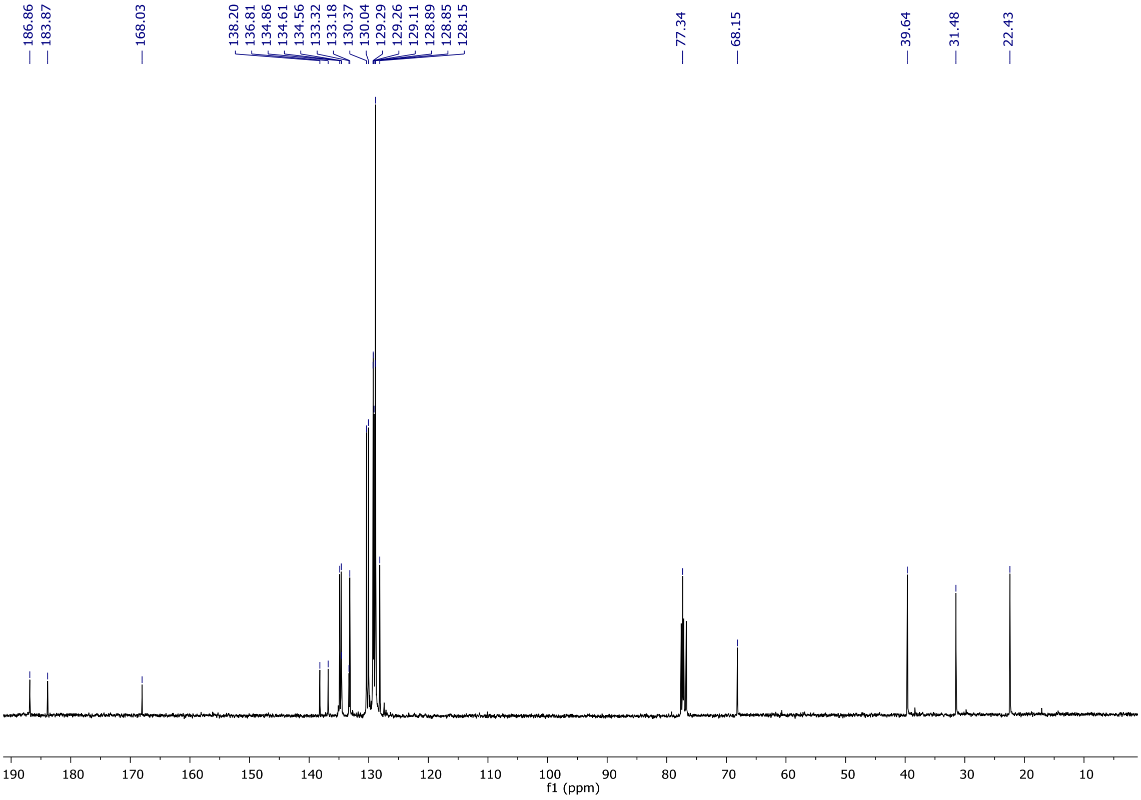

^{1}H NMR (300 MHz, $CDCl_3$) of **10c**:

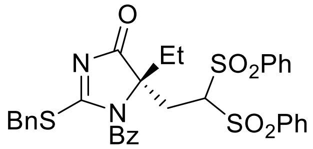

10c

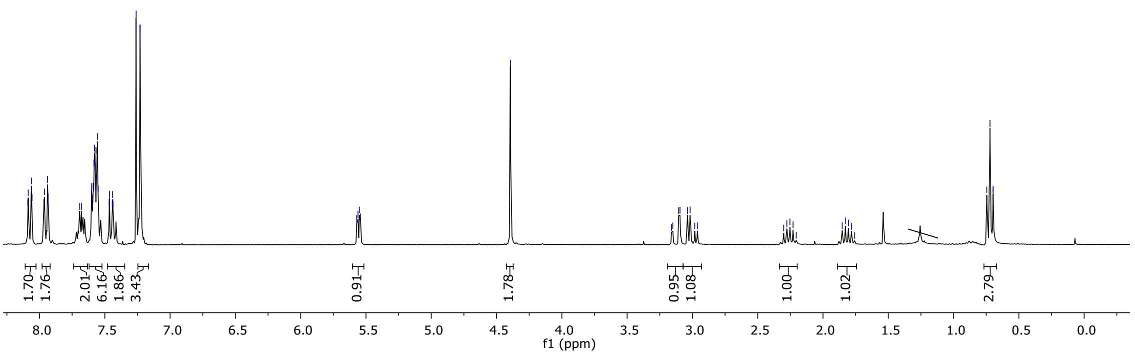

^{13}C{^{1}H} NMR (75 MHz, $CDCl_3$) of **10c**:

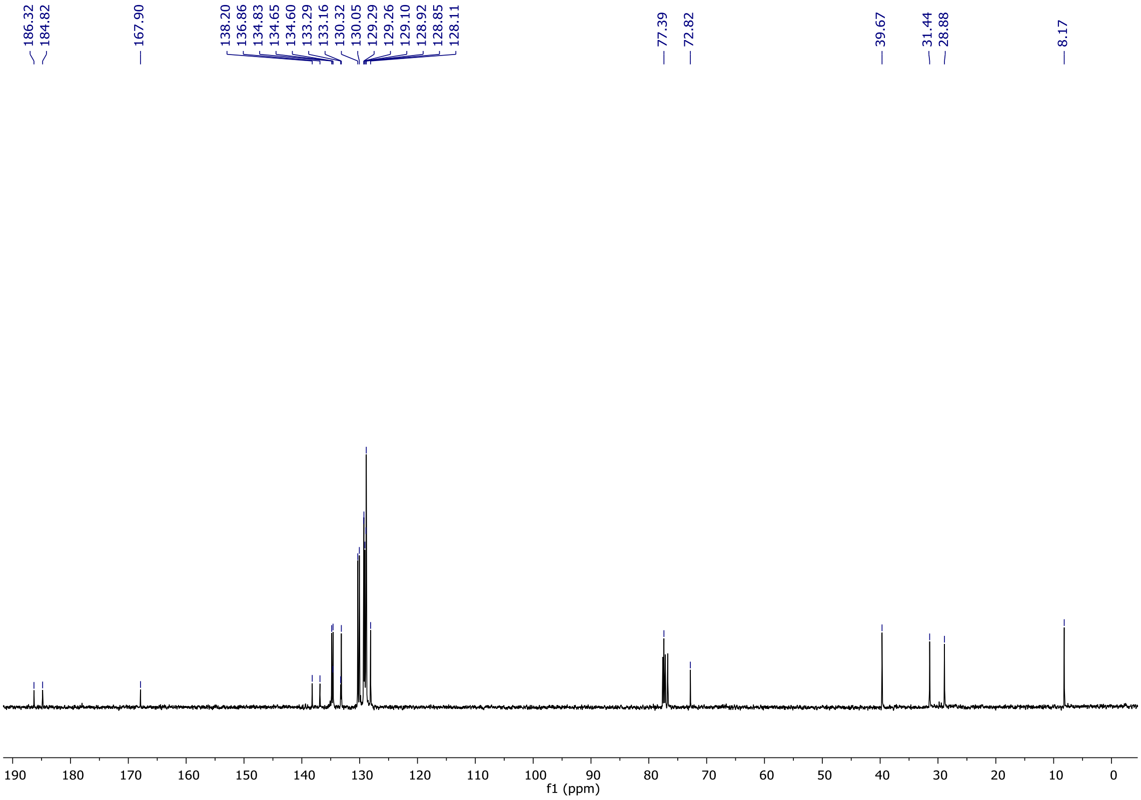

^{1}H NMR (300 MHz, $CDCl_3$) of **10d**:

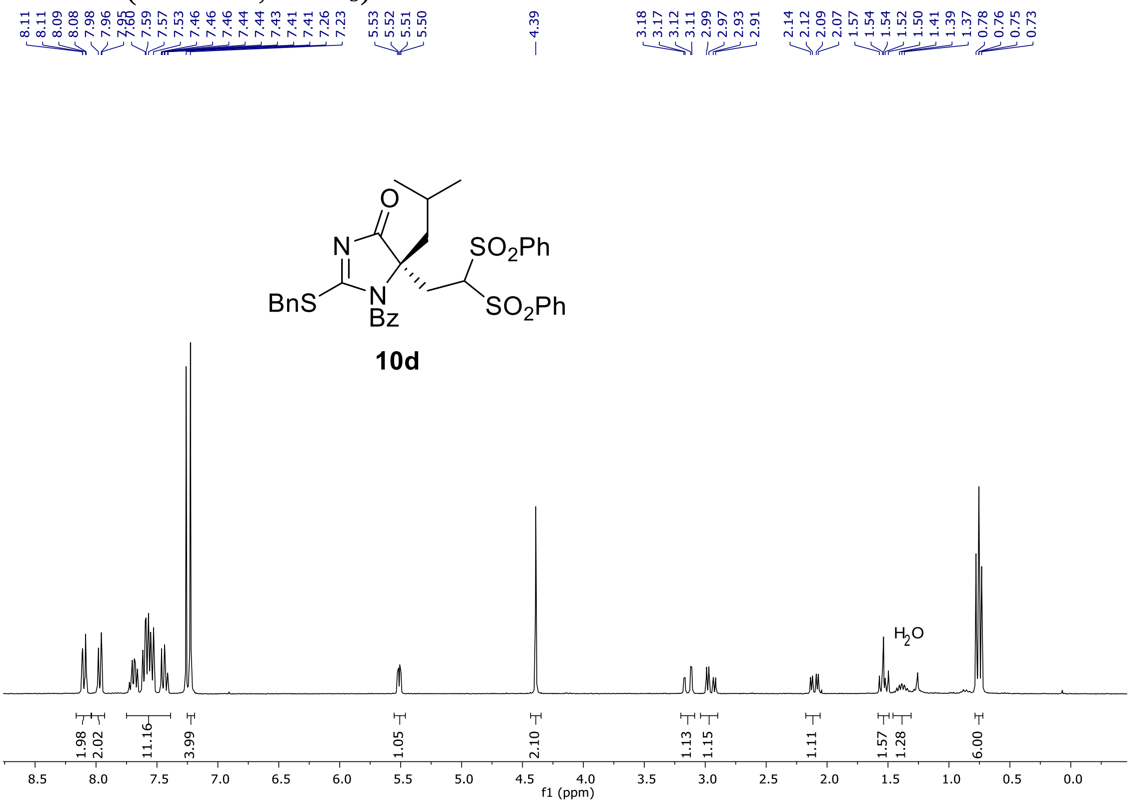

$^{13}C\{^{1}H\}$ NMR (75 MHz, $CDCl_3$) of **10d**:

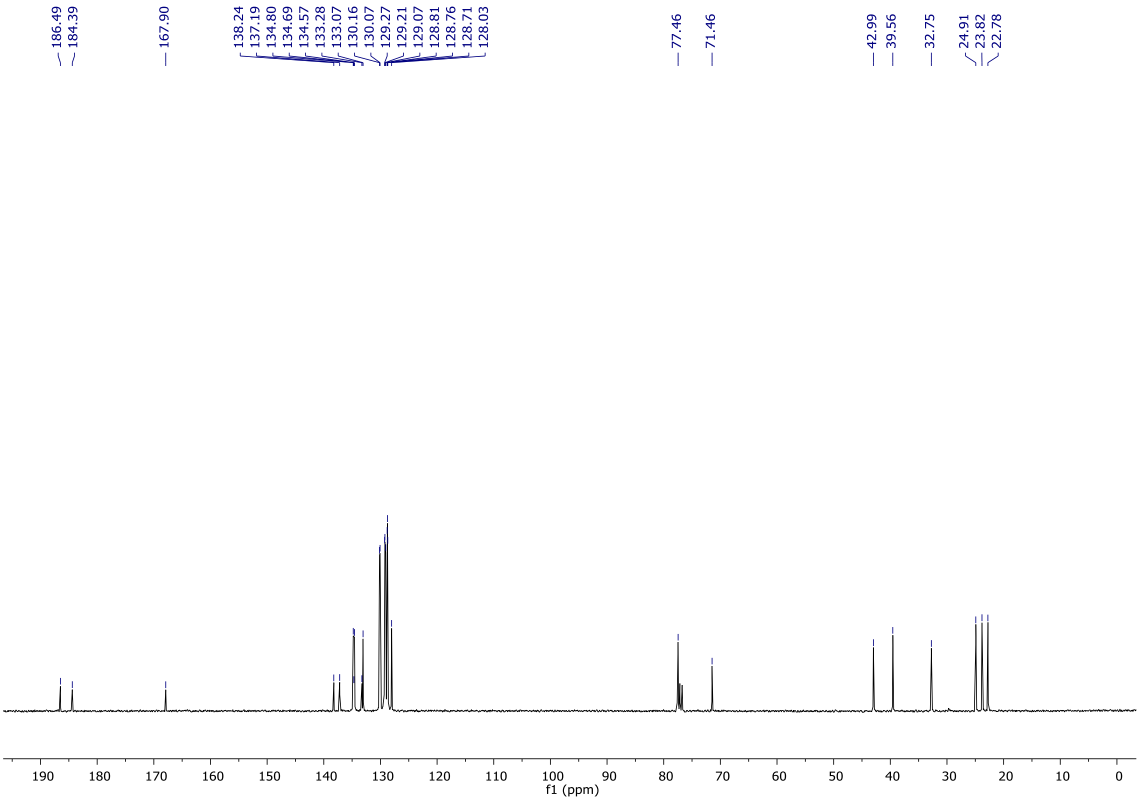

^{1}H NMR (300 MHz, $CDCl_3$) of **10e**:

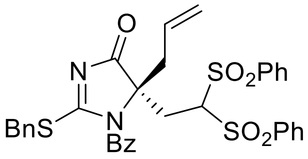

10e

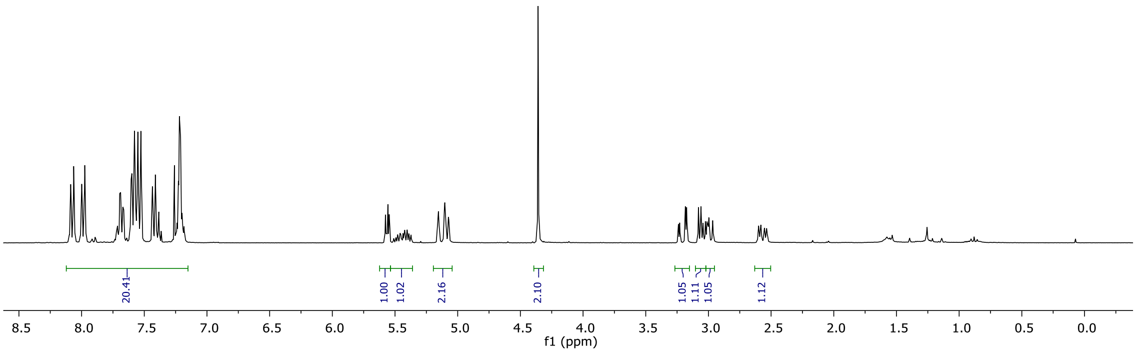

$^{13}C\{^{1}H\}$ NMR (75 MHz, $CDCl_3$) of **10e**:

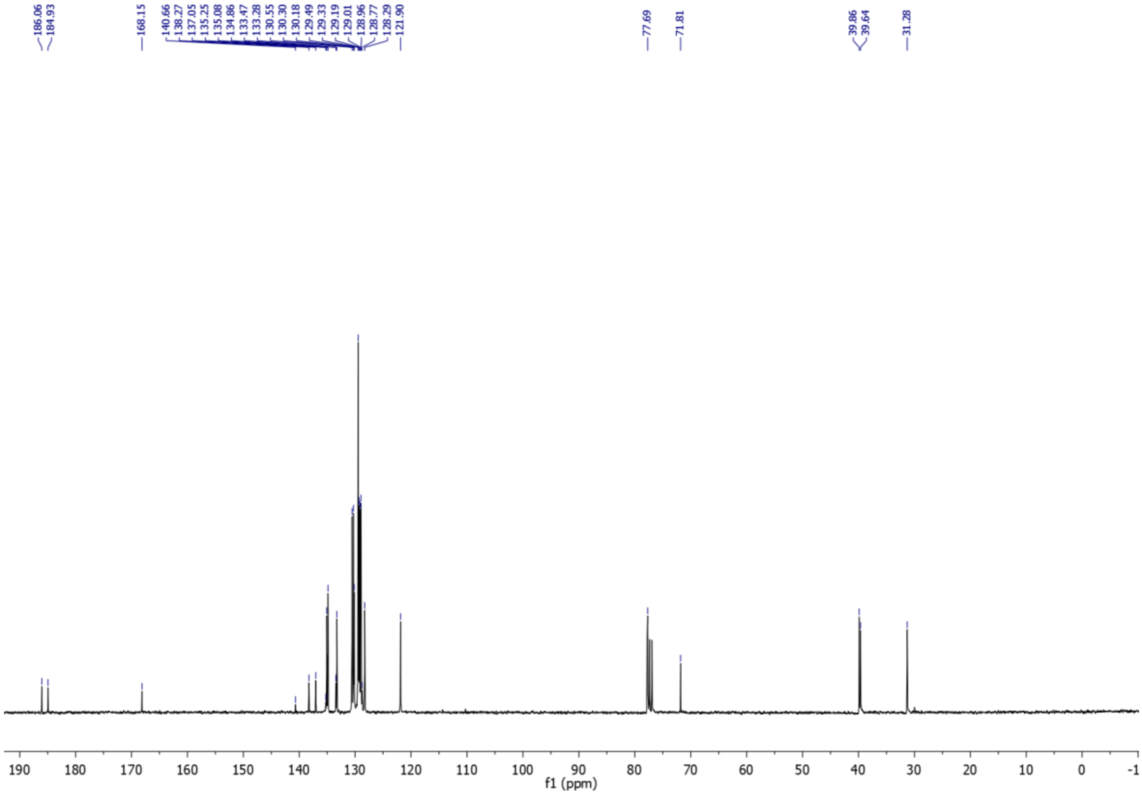

^{1}H NMR (300 MHz, $CDCl_3$) of **10f**:

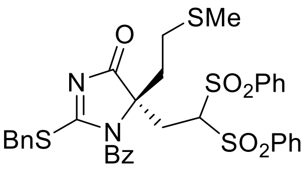

10f

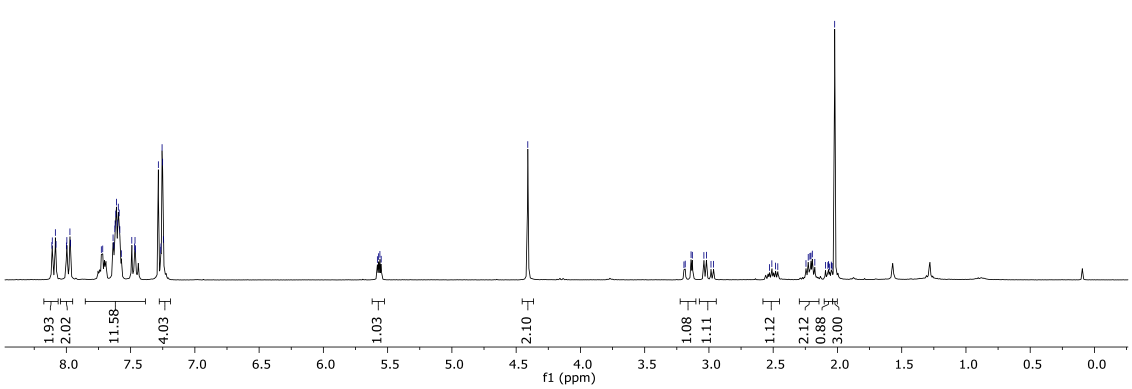

$^{13}C\{^{1}H\}$ NMR (75 MHz, $CDCl_3$) of **10f**:

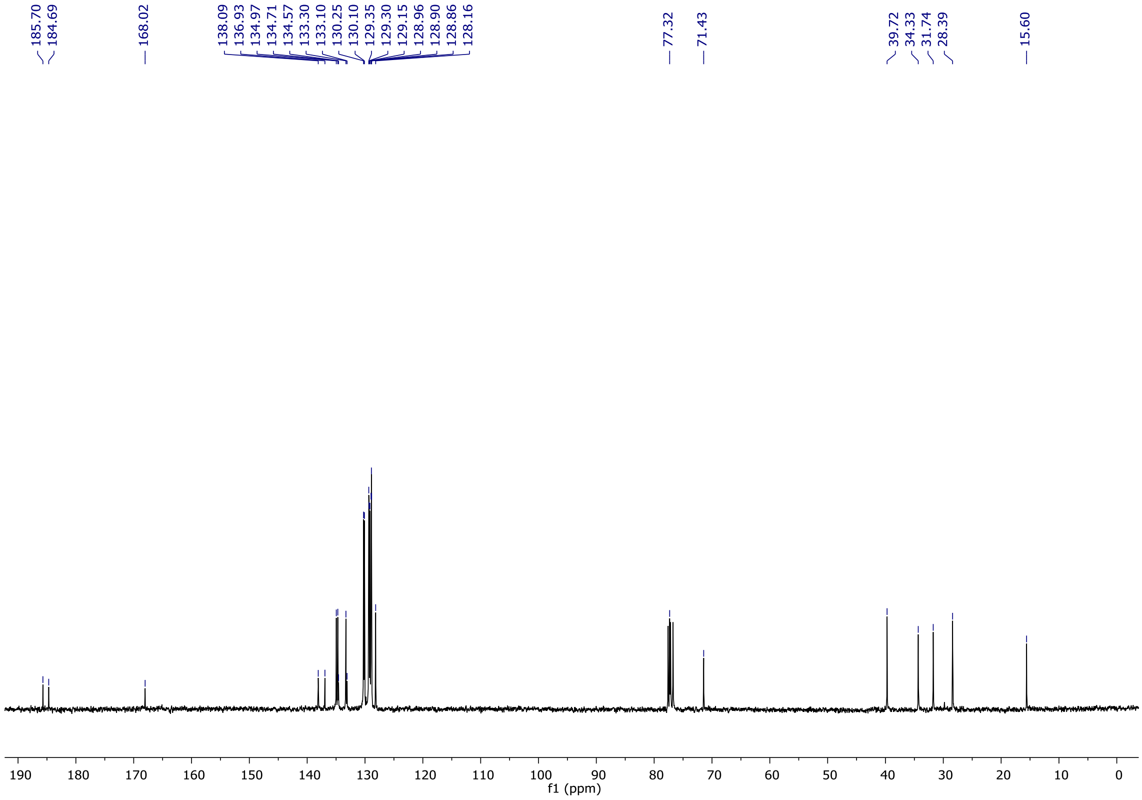

^{1}H NMR (300 MHz, $CDCl_3$) of **10g**:

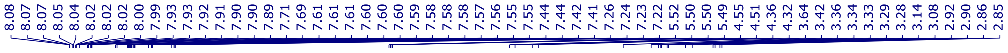

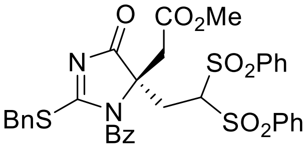

10g

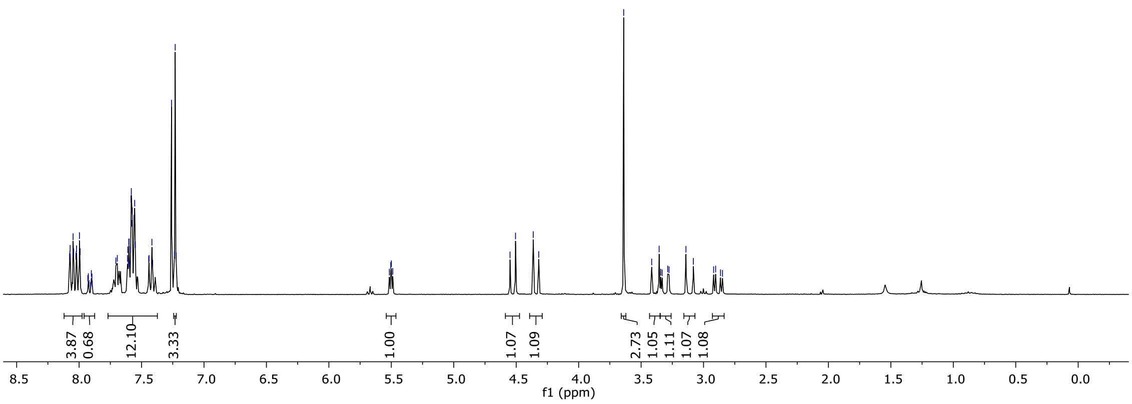

^{13}C{^{1}H} NMR (75 MHz, $CDCl_3$) of **10g**:

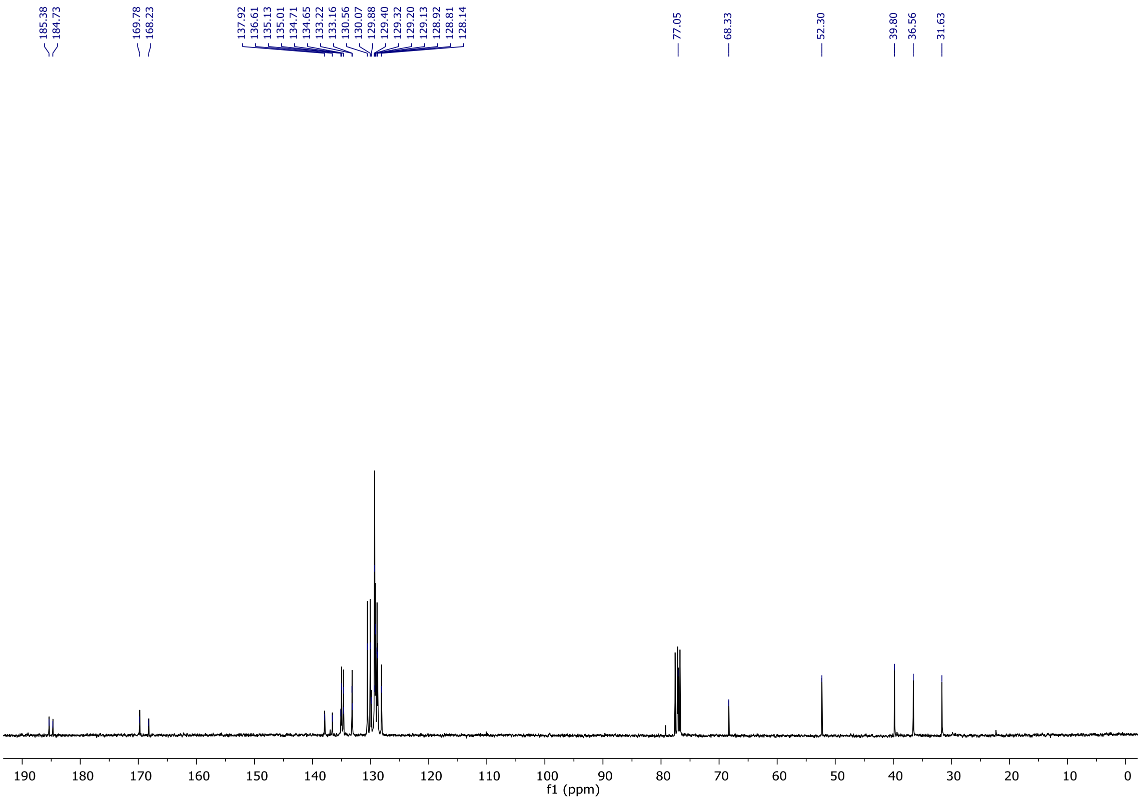

^{1}H NMR (300 MHz, $CDCl_3$) of **12**:

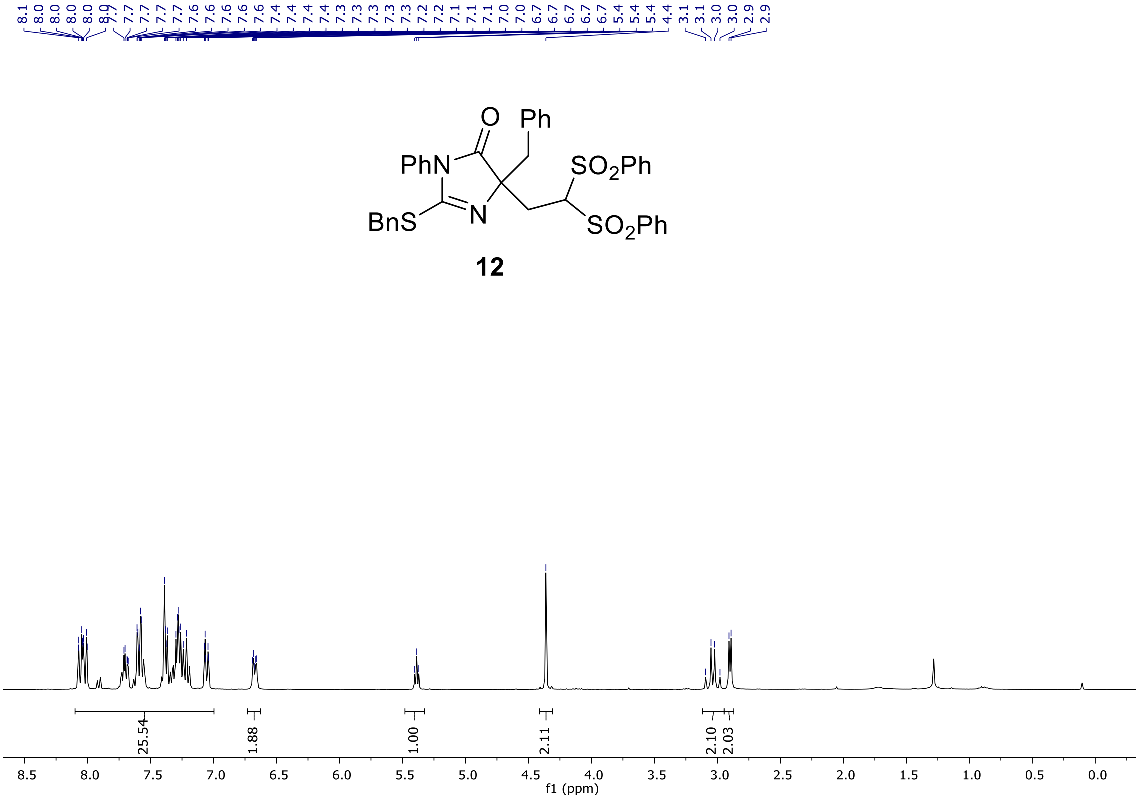

$^{13}C\{^{1}H\}$ NMR (75 MHz, $CDCl_3$) of **12**:

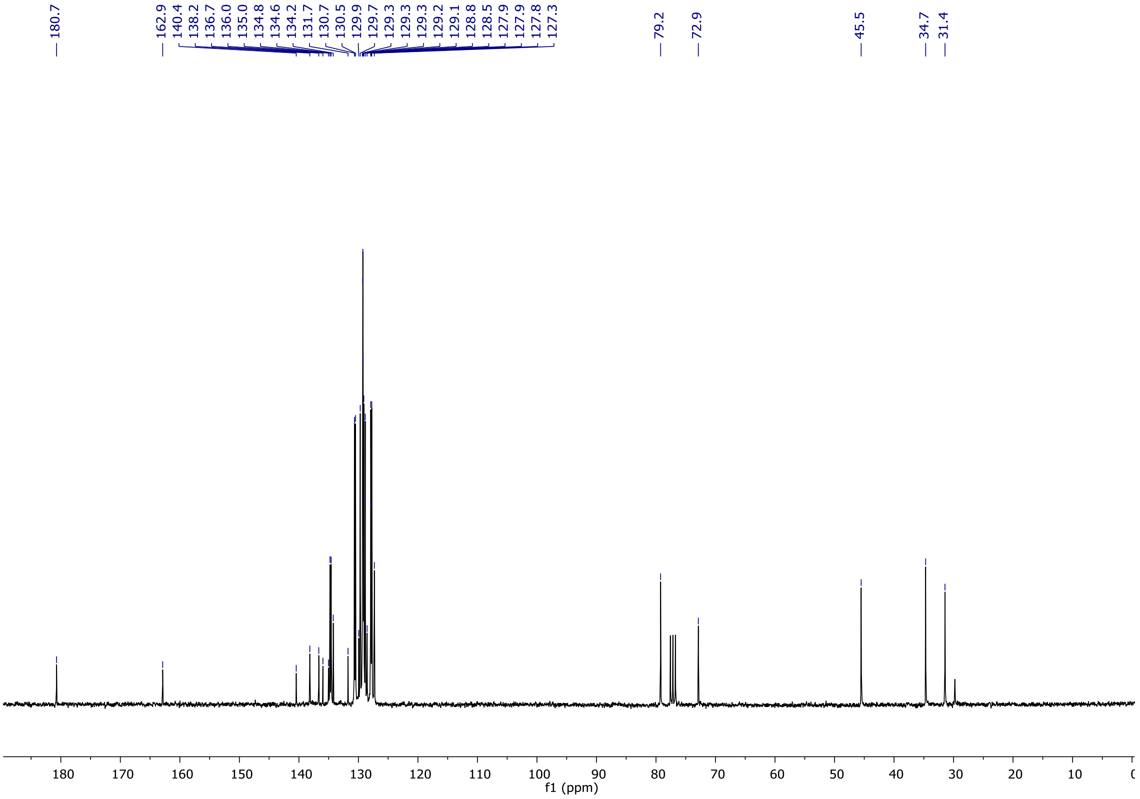

^{1}H NMR (300 MHz, $CDCl_3$) of **13**:

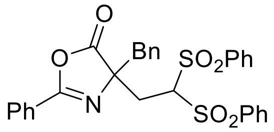

13

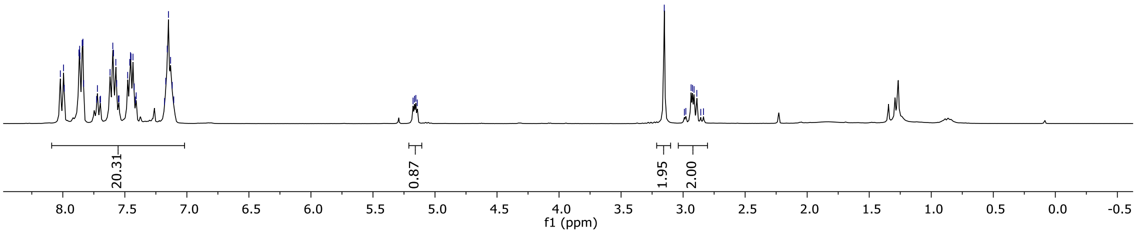

^{13}C{^{1}H} NMR (75 MHz, $CDCl_3$) of **13**:

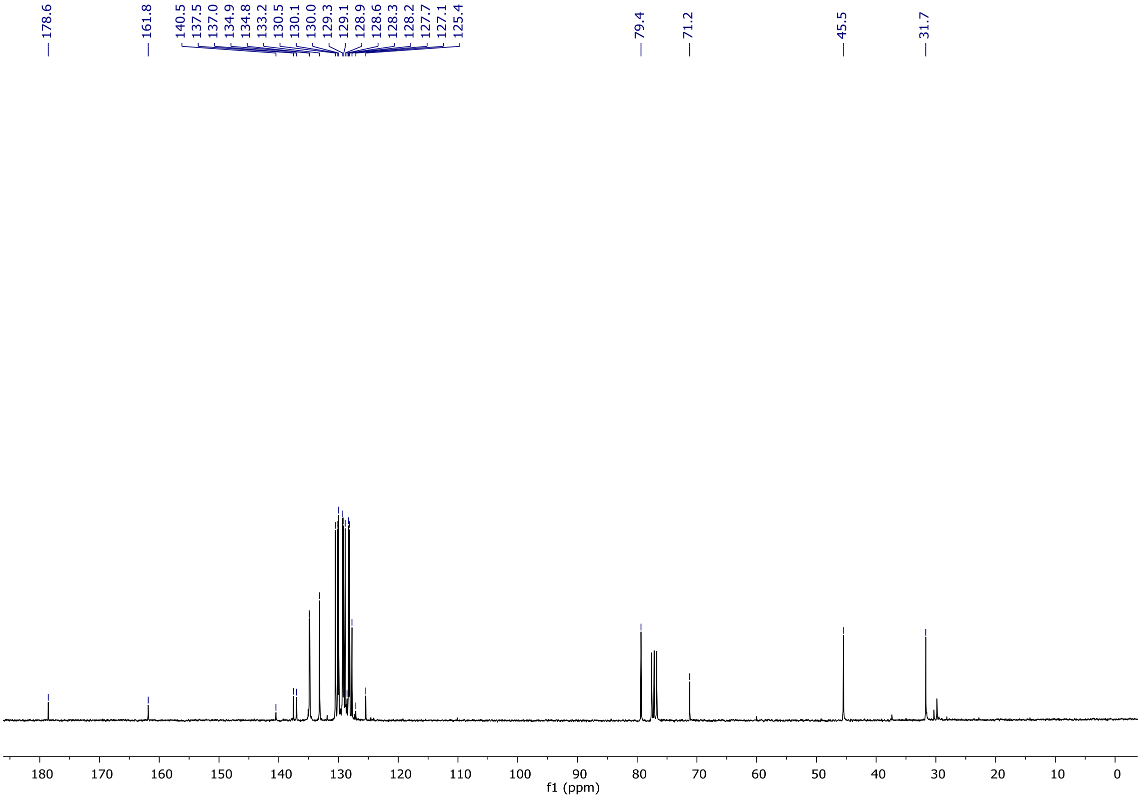

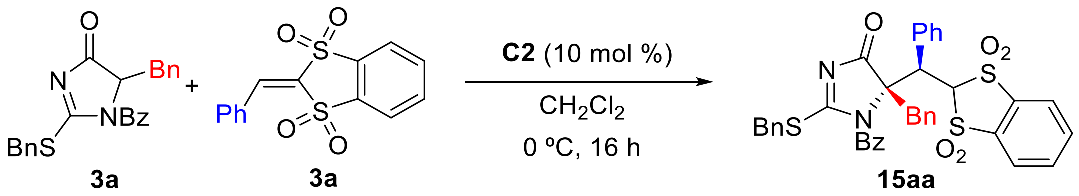

Crude reaction ^{1}H NMR (300 MHz, $CDCl_3$) of **15aa**:

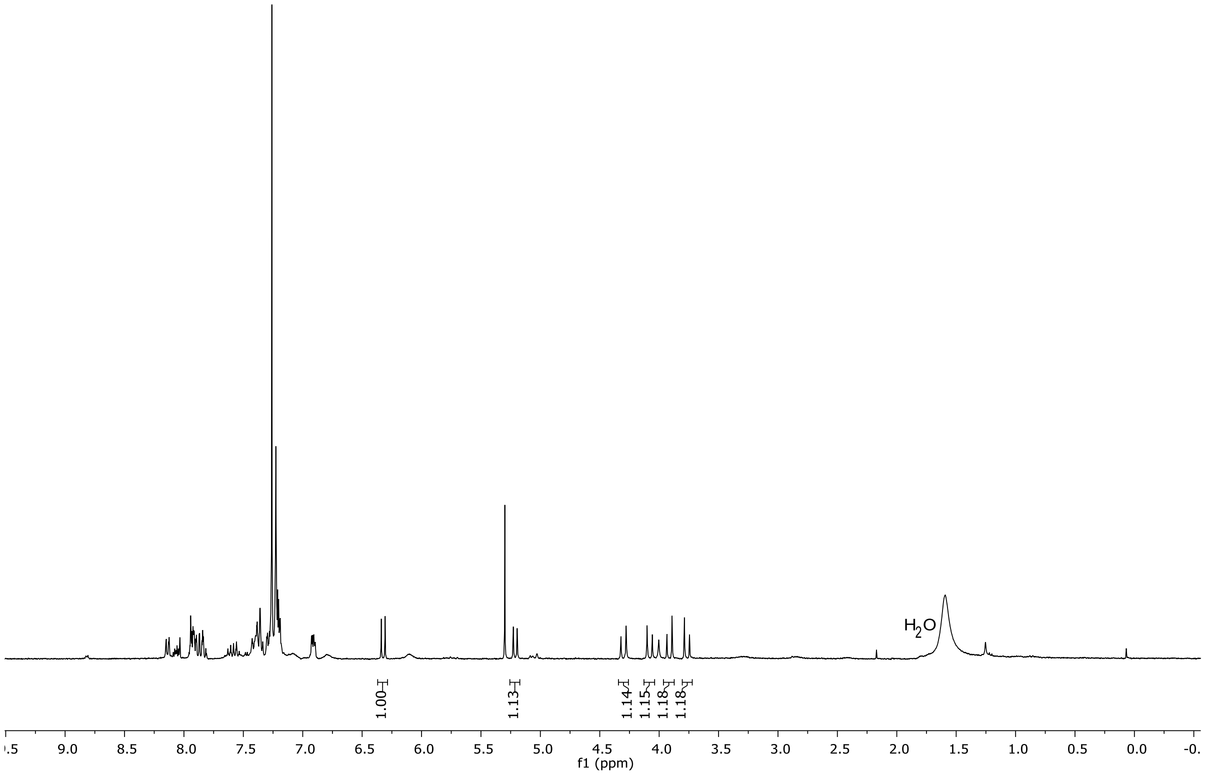

^{1}H NMR (300 MHz, $CDCl_3$) of **15aa**:

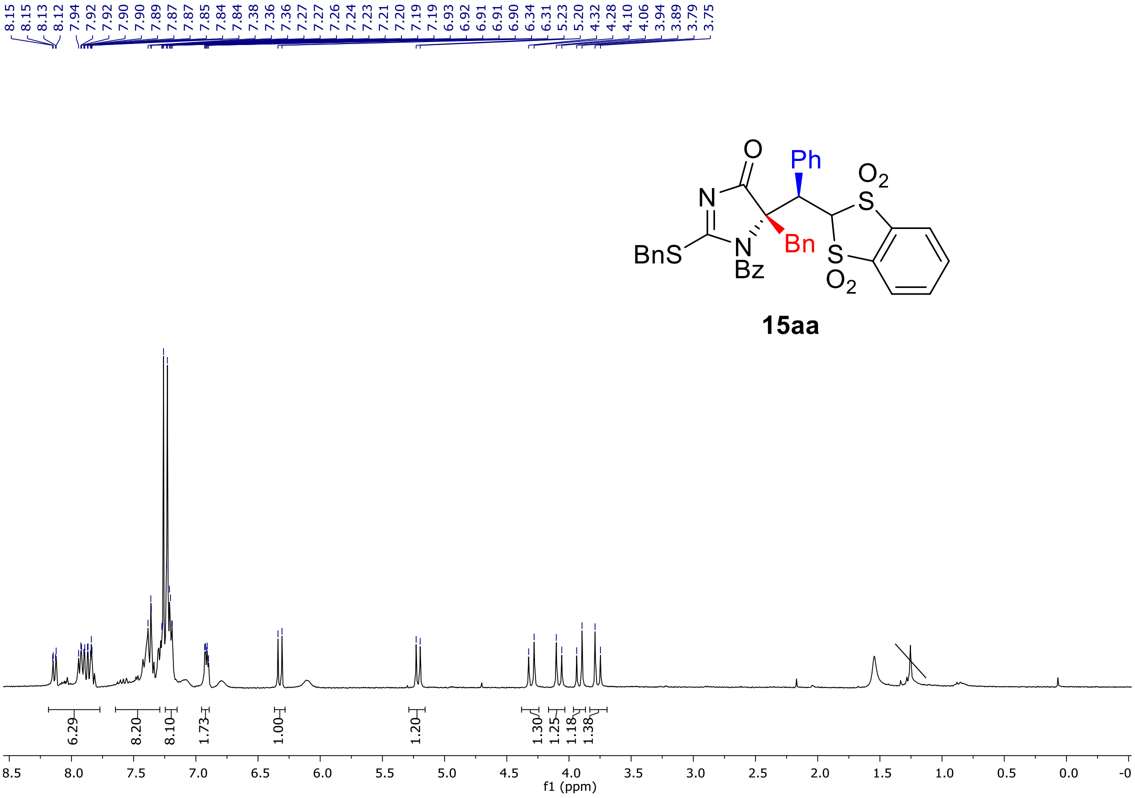

15aa

^{13}C{^{1}H} NMR (75 MHz, $CDCl_3$) of **15aa**:

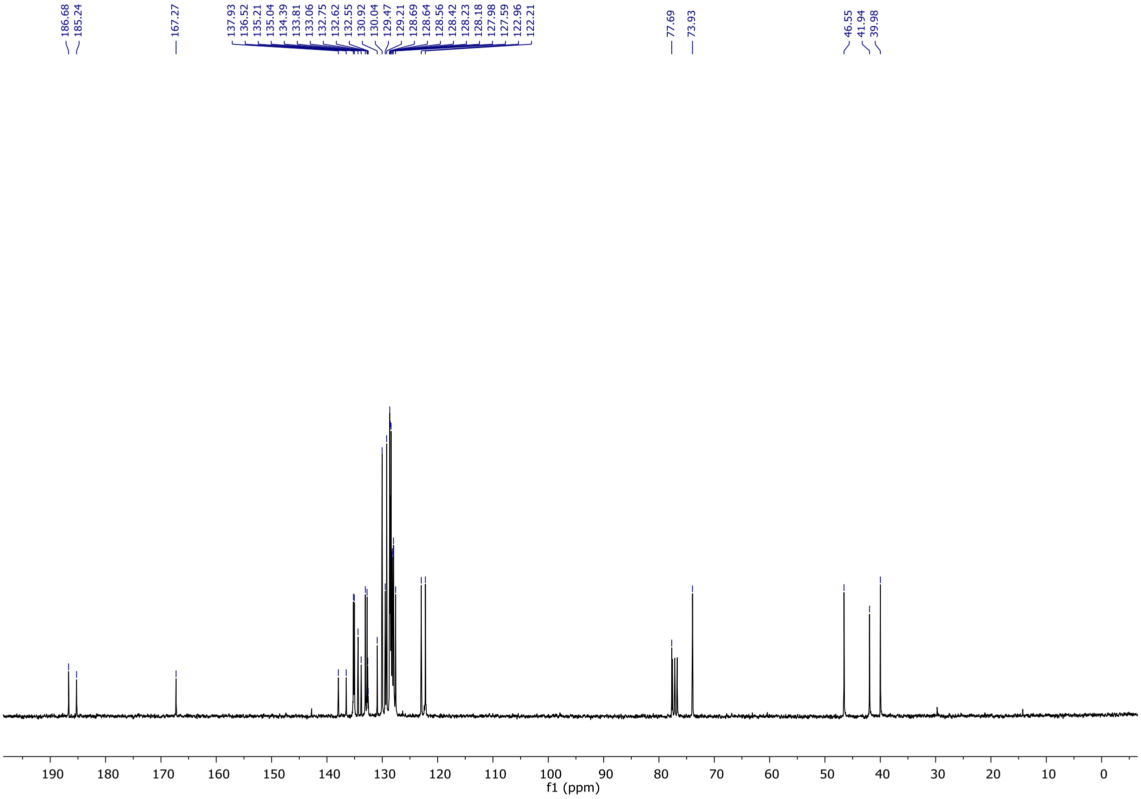

^{1}H NMR (300 MHz, $CDCl_3$) of **15ca**:

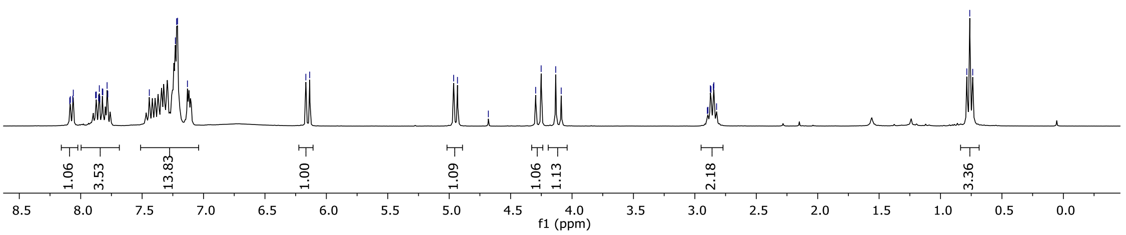

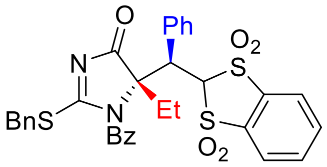

15ca

^{13}C{^{1}H} NMR (75 MHz, $CDCl_3$) of **15ca**:

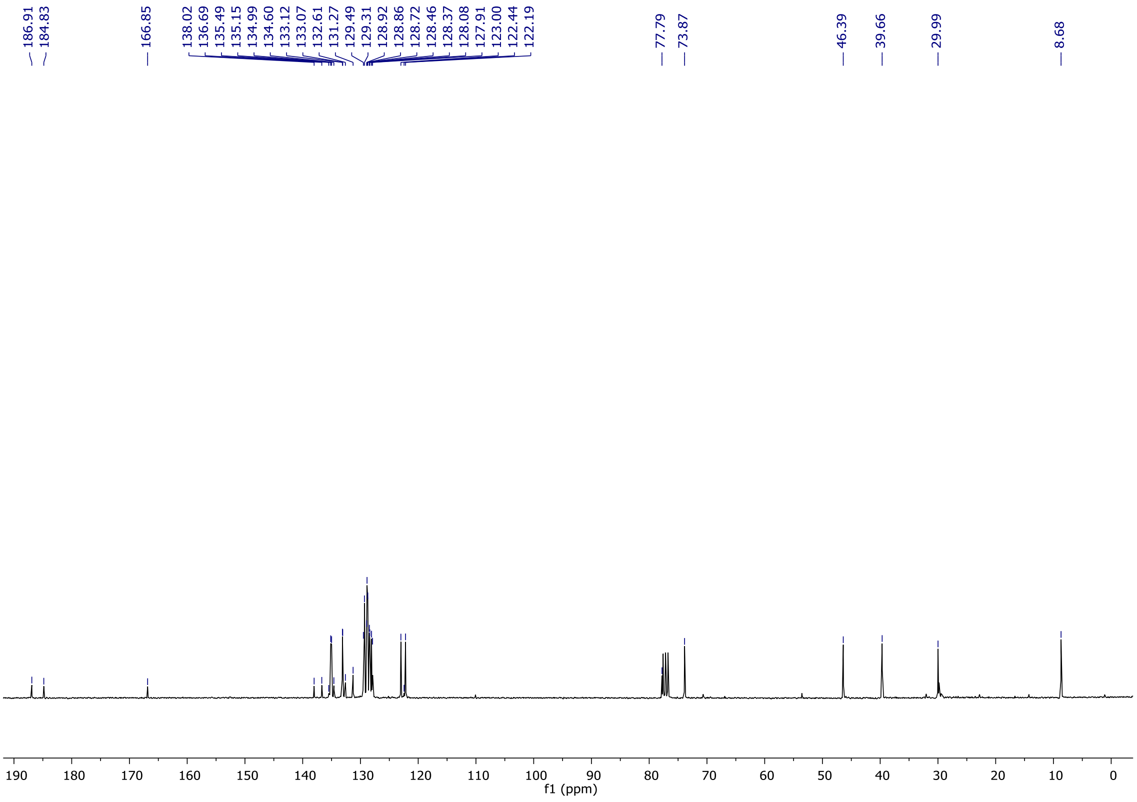

^{1}H NMR (300 MHz, $CDCl_3$) of **15da**:

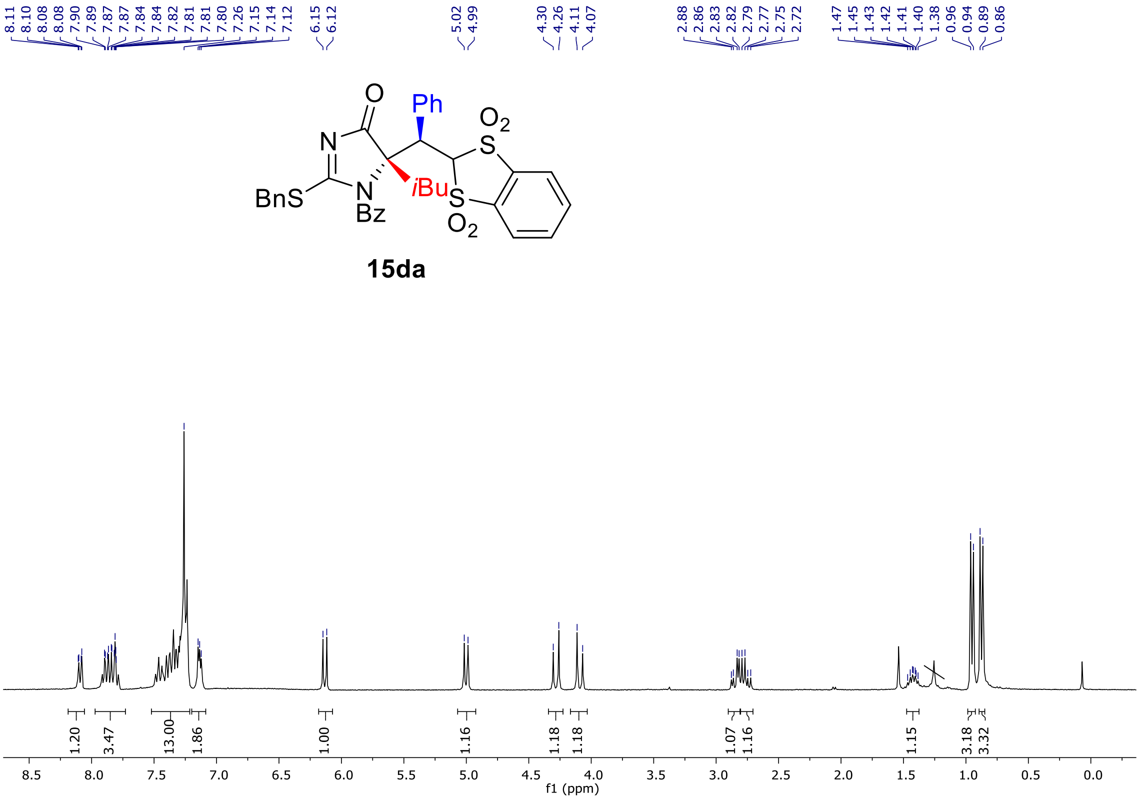

^{13}C{^{1}H} NMR (75 MHz, $CDCl_3$) of **15da**:

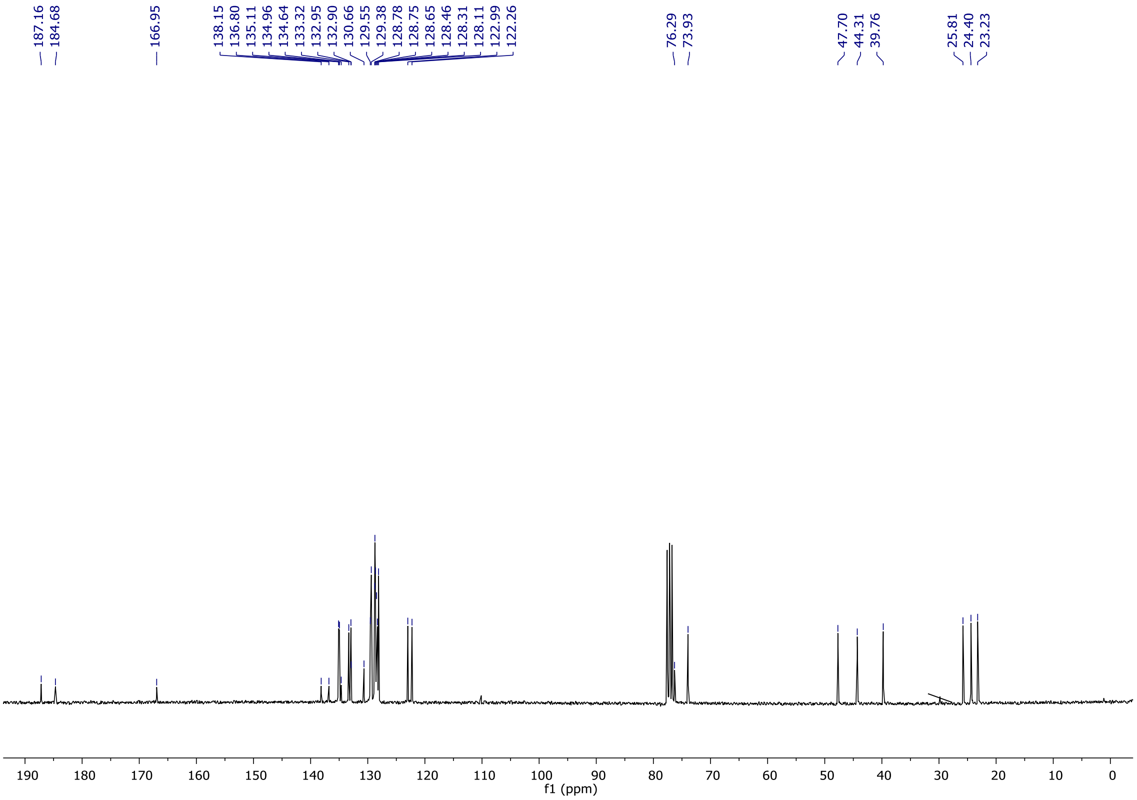

^{1}H NMR (300 MHz, $CDCl_3$) of **15ea**:

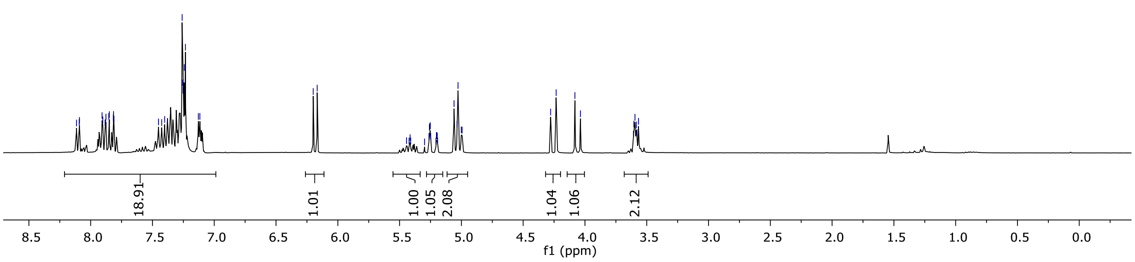

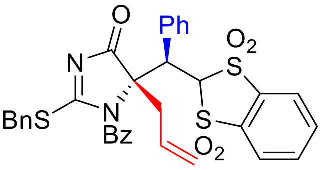

15ea

$^{13}C\{^{1}H\}$ NMR (75 MHz, $CDCl_3$) of **15ea**:

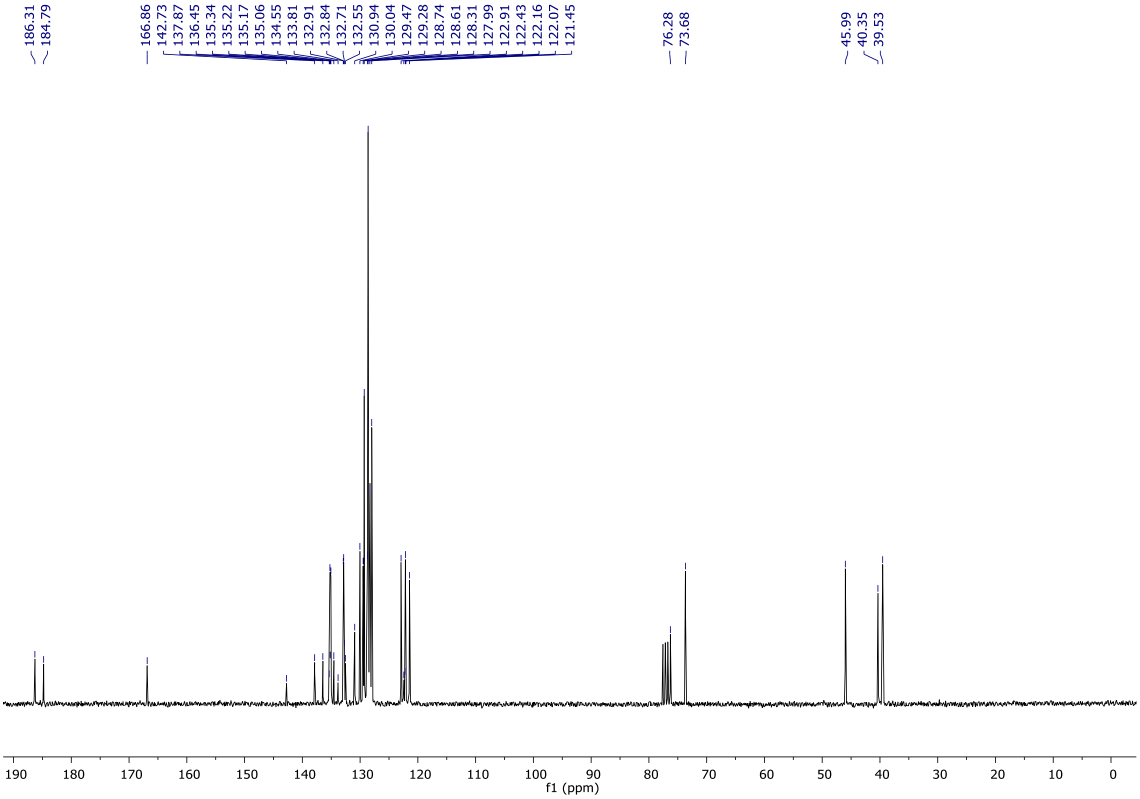

^{1}H NMR (300 MHz, $CDCl_3$) of **15fa**:

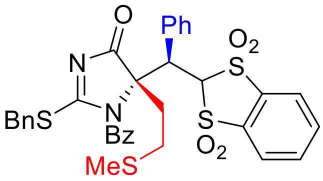

15fa

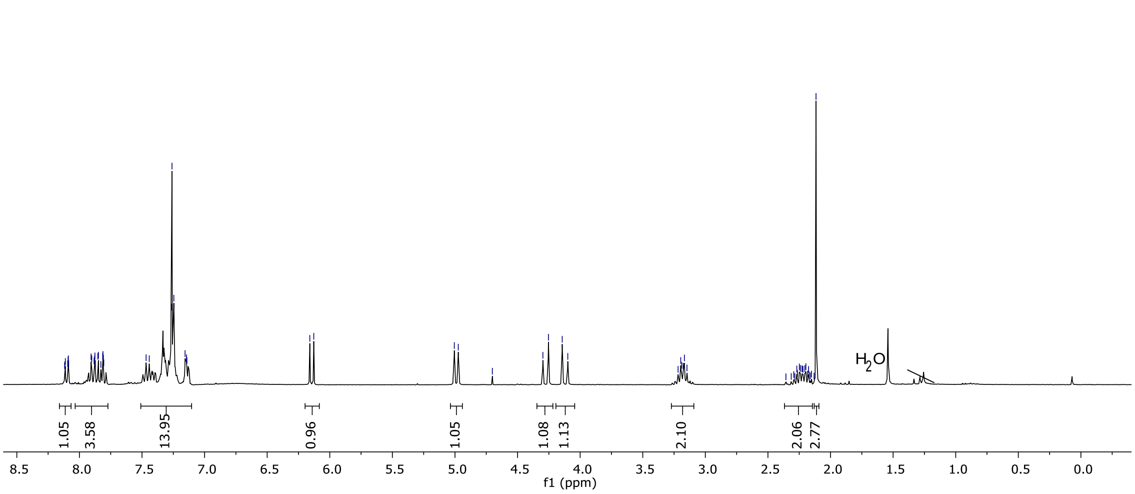

$^{13}C\{^{1}H\}$ NMR (75 MHz, $CDCl_3$) of **15fa**:

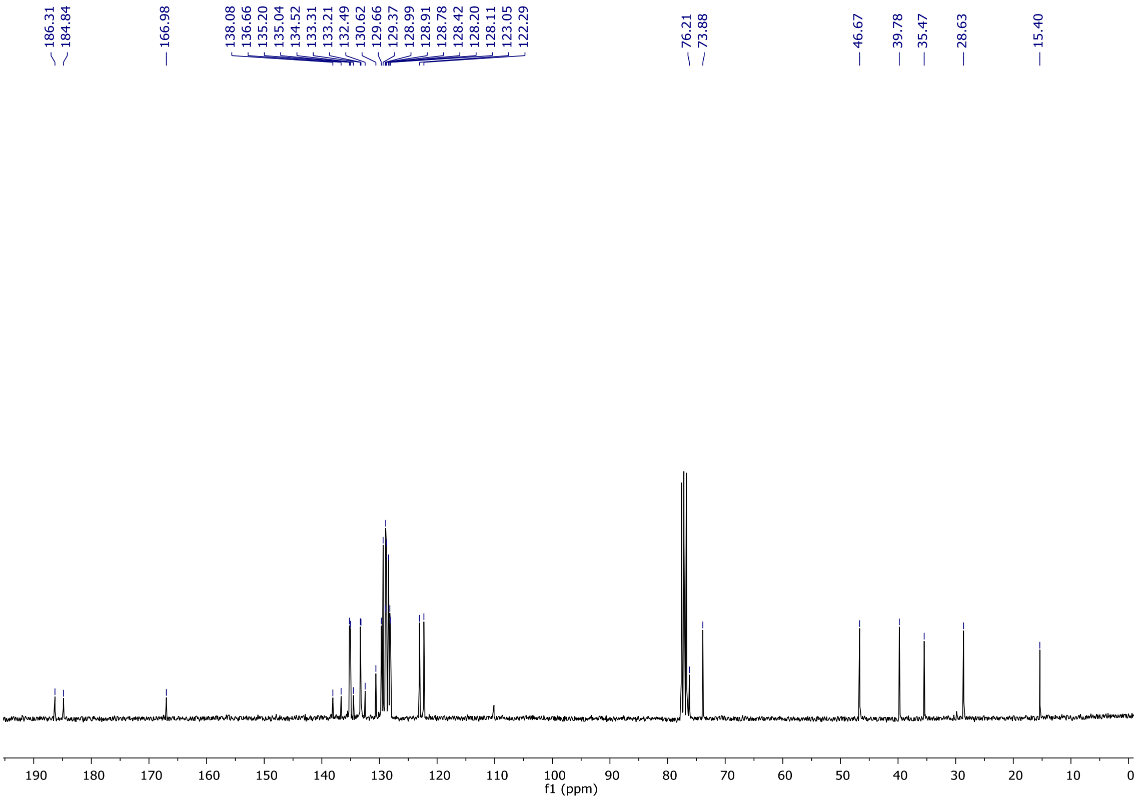

^{1}H NMR (300 MHz, $CDCl_3$) of **15ga**:

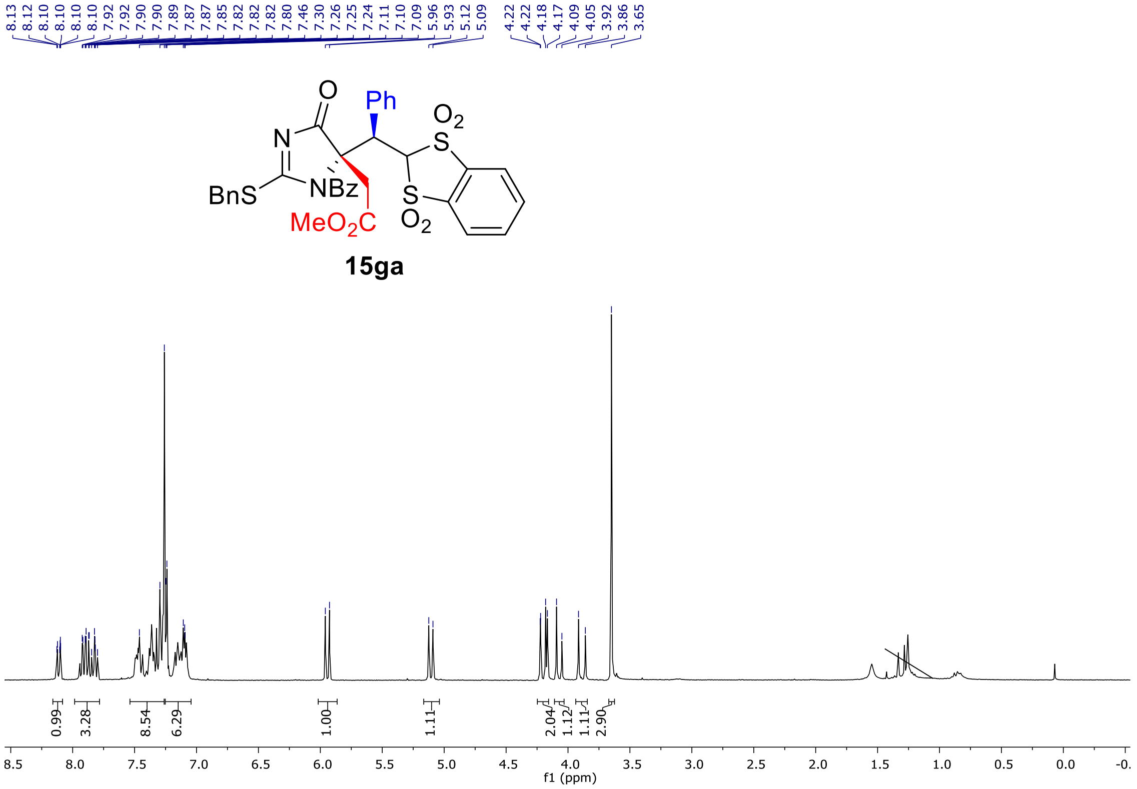

^{13}C{^{1}H} NMR (75 MHz, $CDCl_3$) of **15ga**:

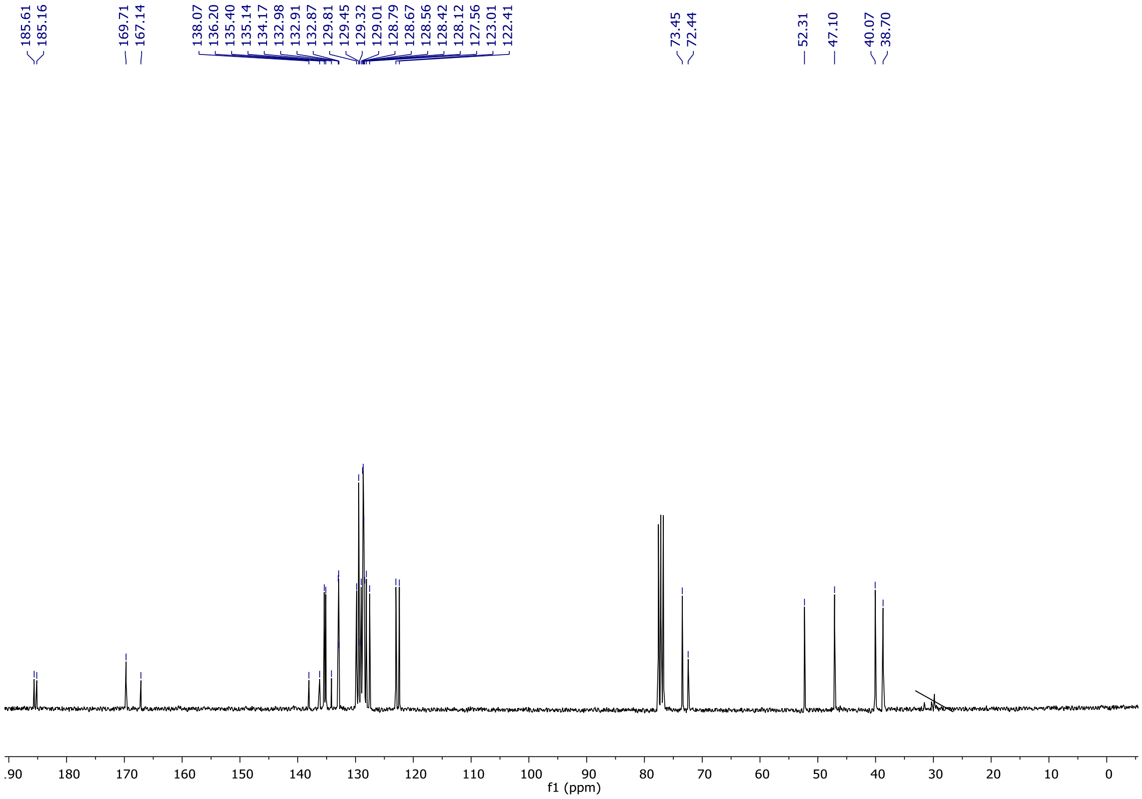

^{1}H NMR (300 MHz, $CDCl_3$) of **15ha**:

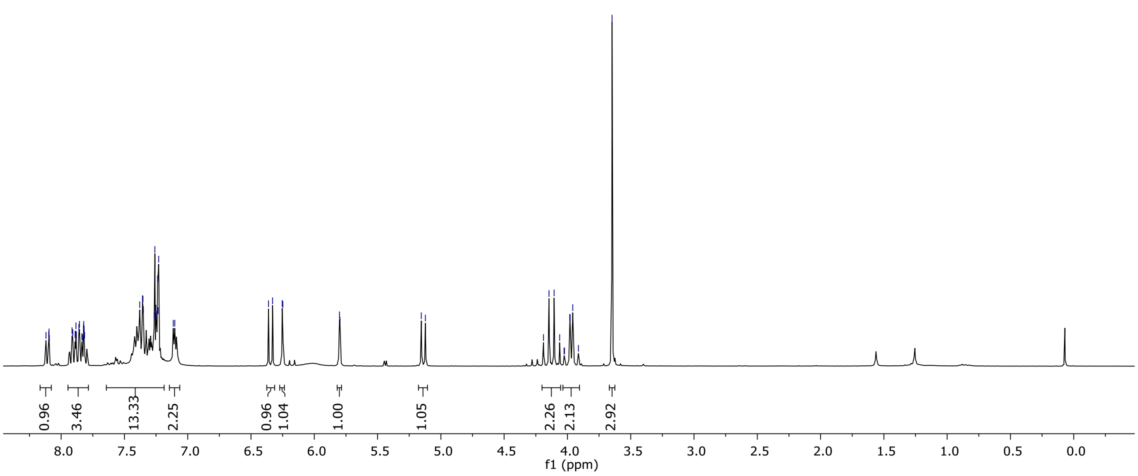

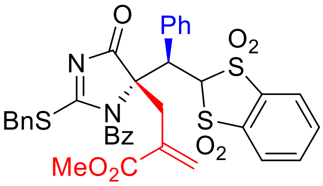

15ha

$^{13}C\{^{1}H\}$ NMR (75 MHz, $CDCl_3$) of **15ha**:

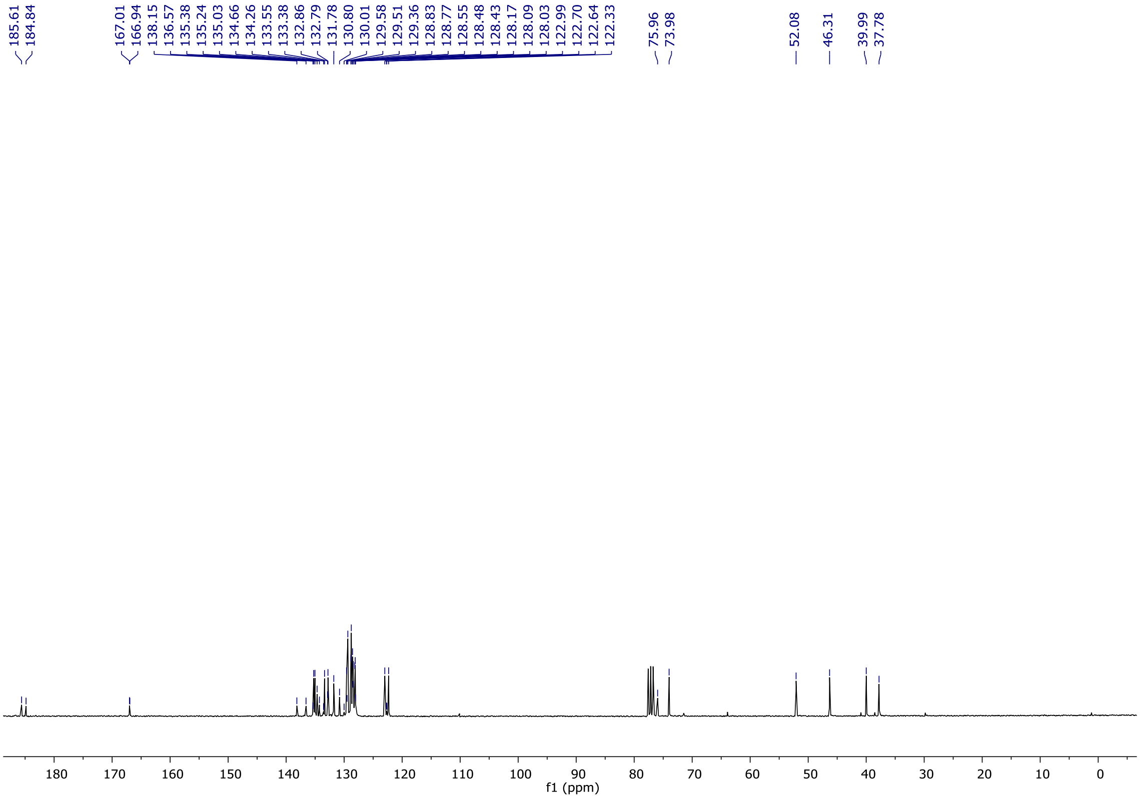

^{1}H NMR (300 MHz, $CDCl_3$) of **15ab**:

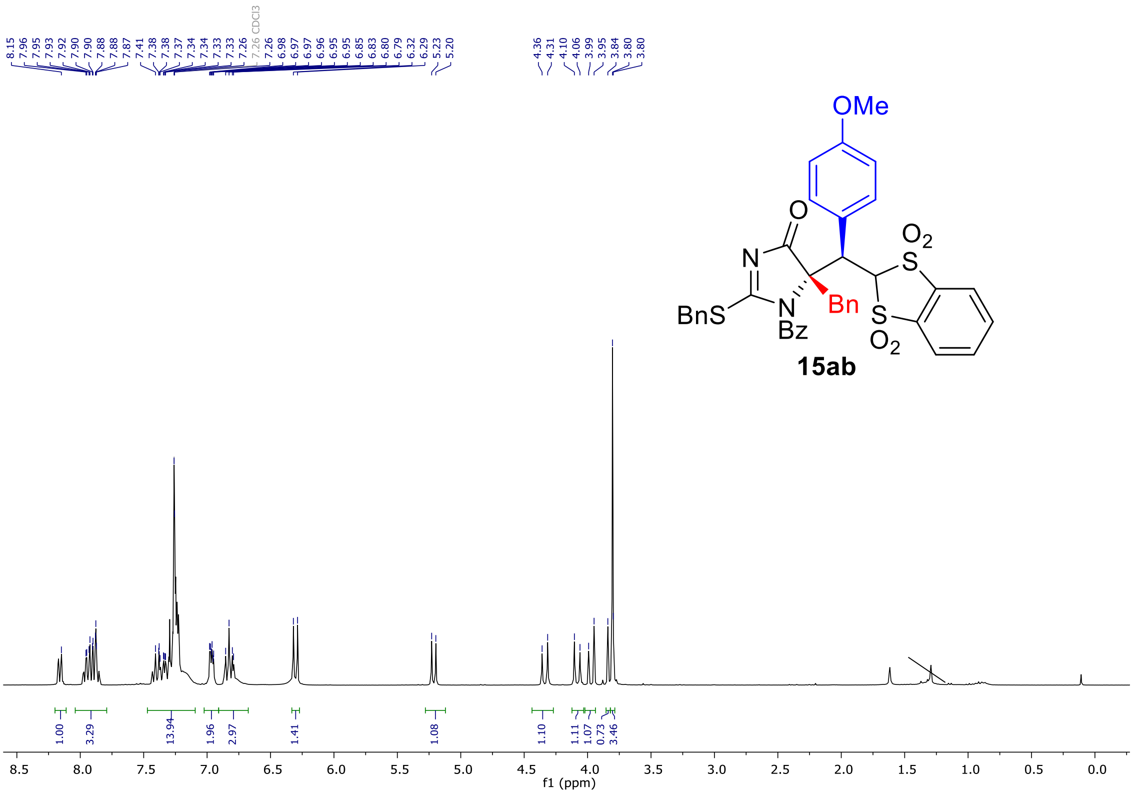

$^{13}C\{^{1}H\}$ NMR (75 MHz, $CDCl_3$) of **15ab**:

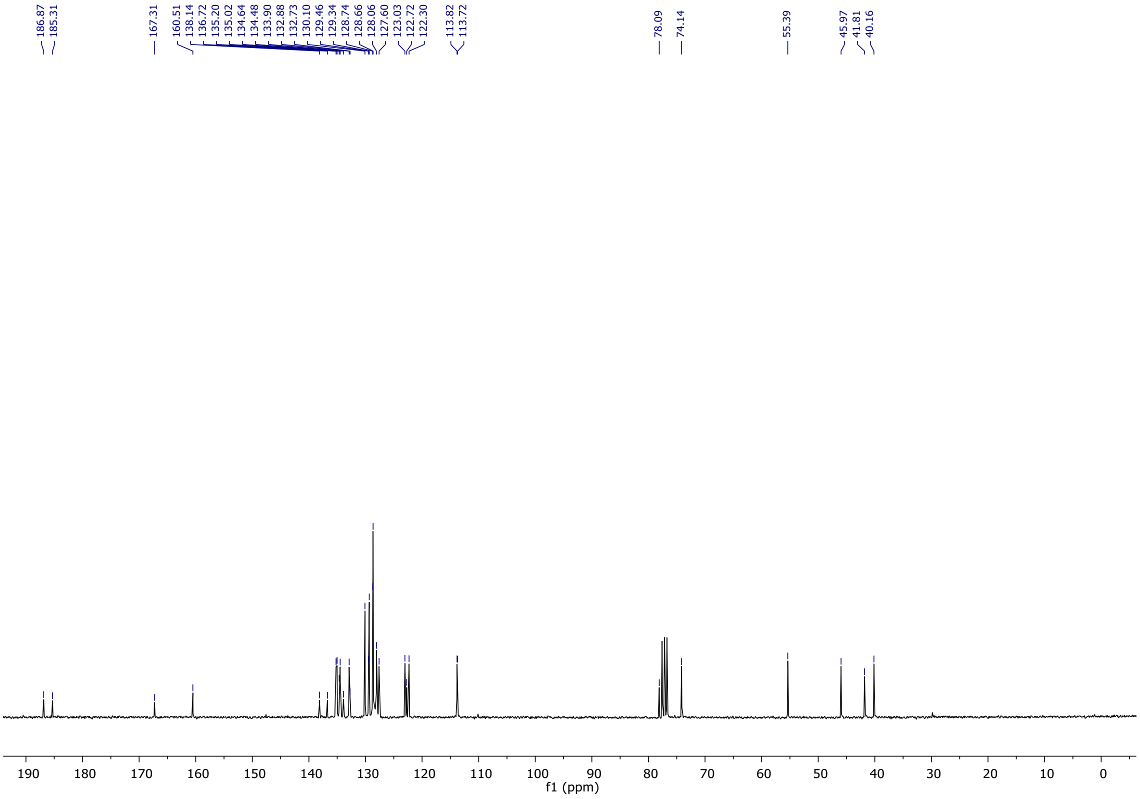

^{1}H NMR (300 MHz, $CDCl_3$) of **15ac**:

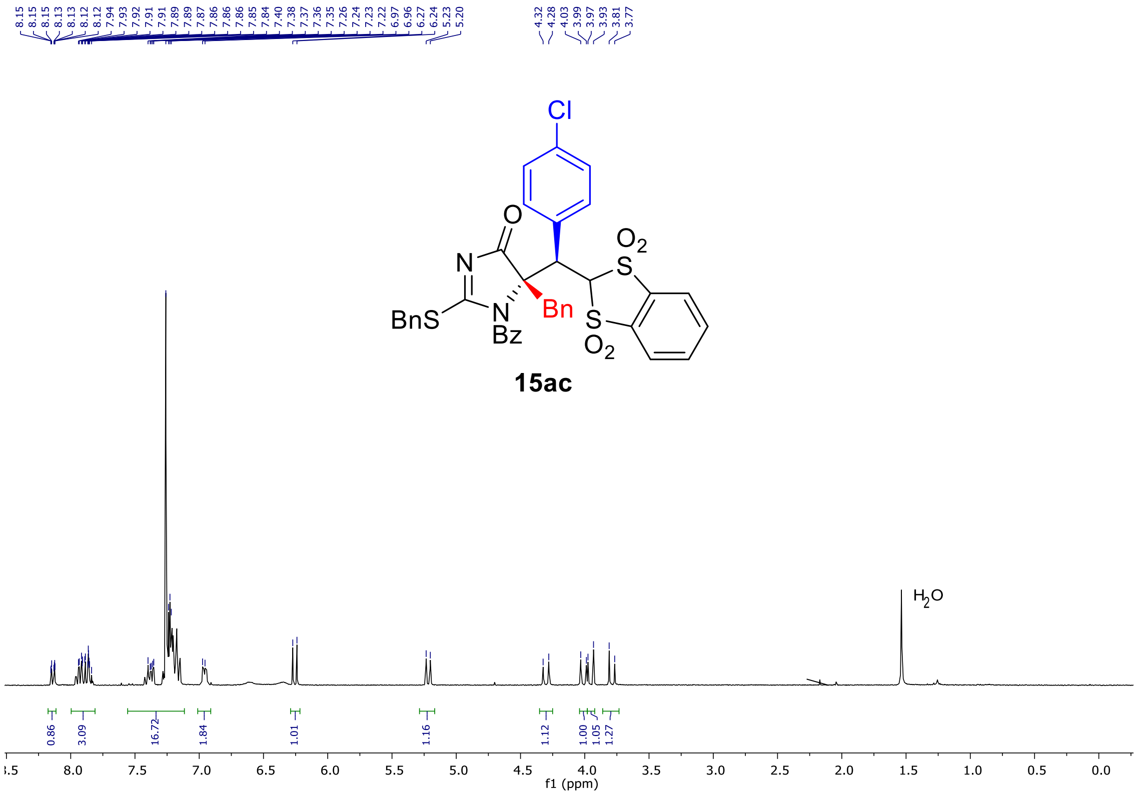

^{13}C{^{1}H} NMR (75 MHz, $CDCl_3$) of **15ac**:

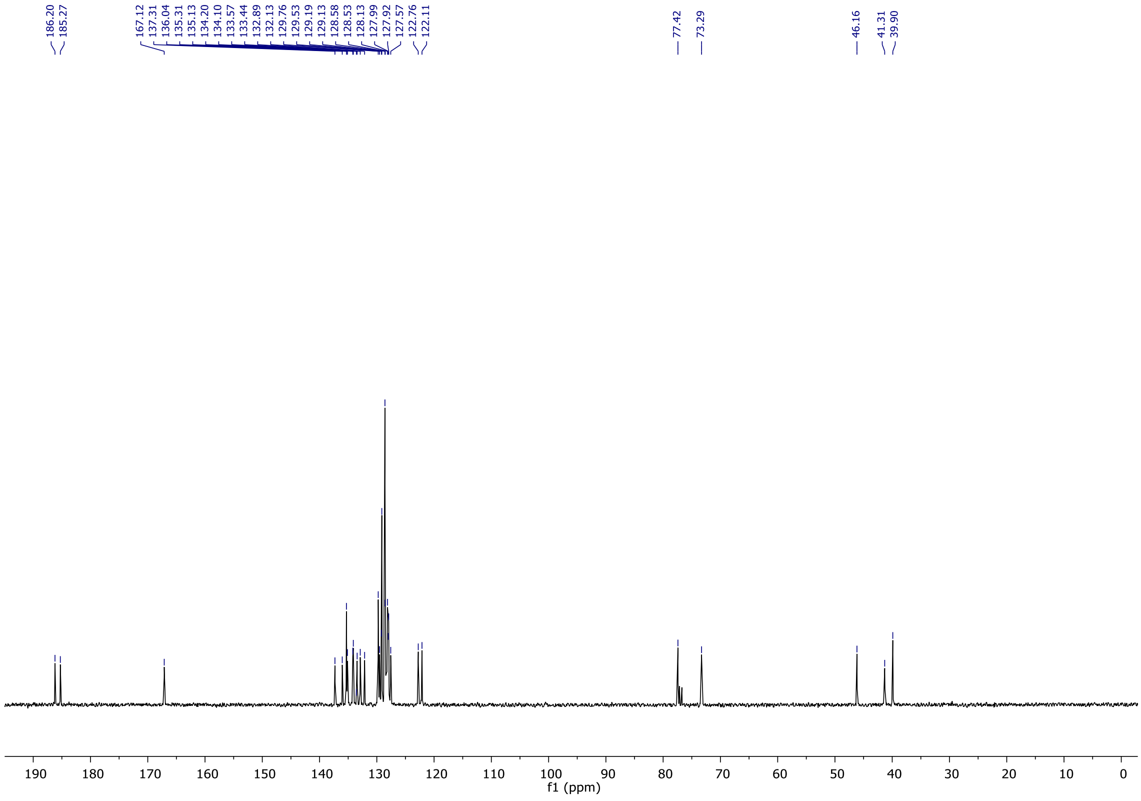

^{1}H NMR (300 MHz, $CDCl_3$) of **15bc**:

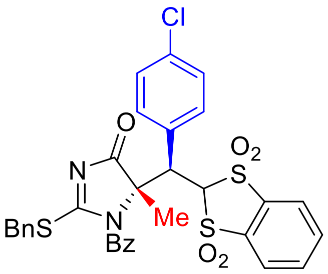

15bc

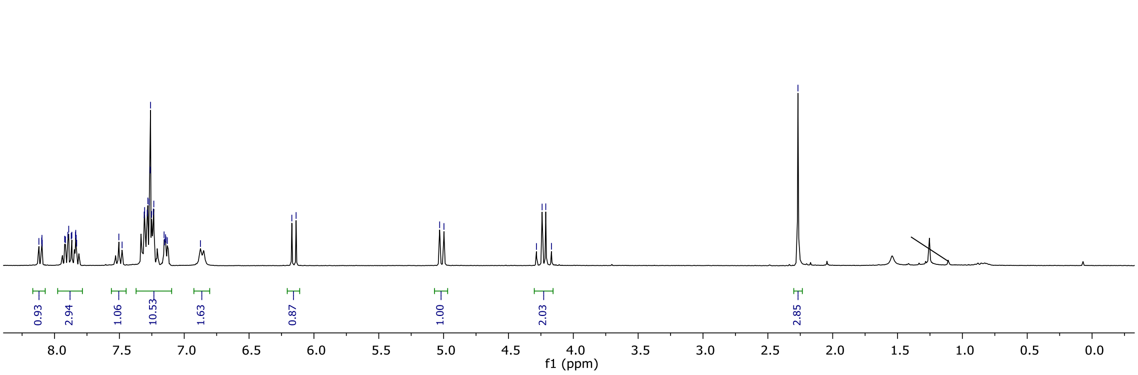

^{13}C{^{1}H} NMR (75 MHz, $CDCl_3$) of **15bc**:

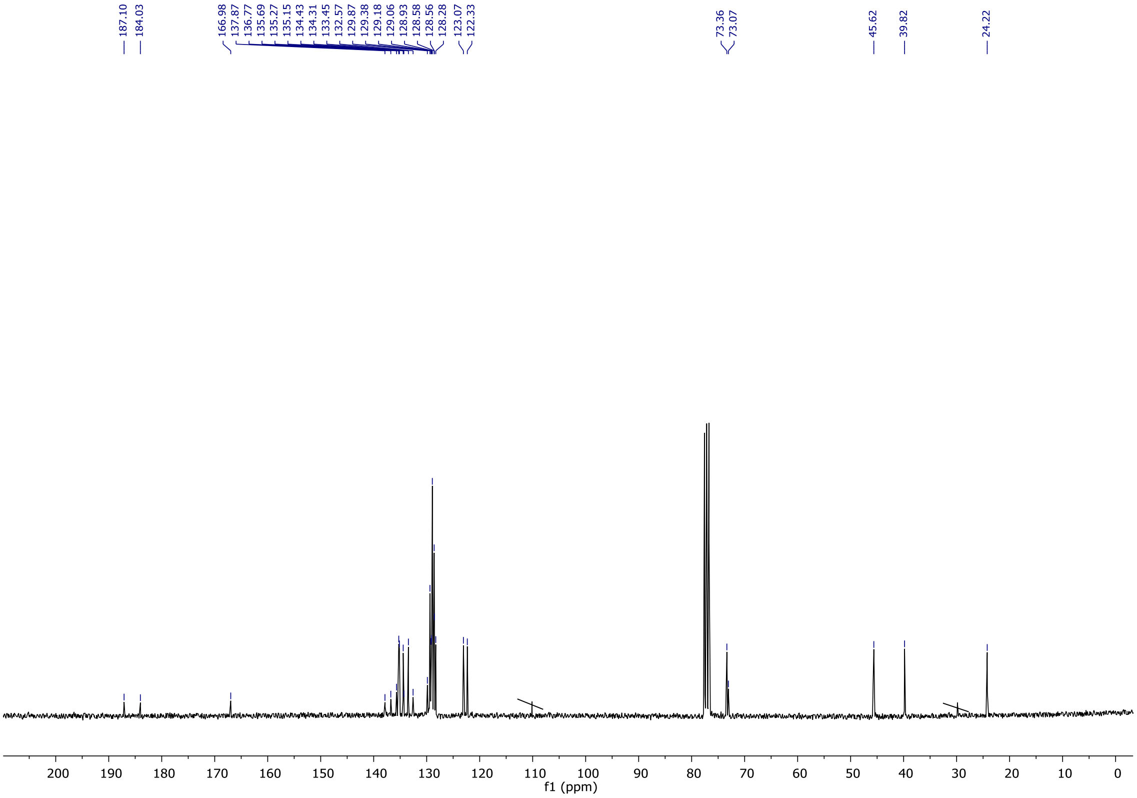

^{1}H NMR (300 MHz, $CDCl_3$) of **15ec**:

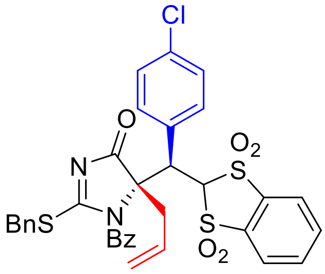

15ec

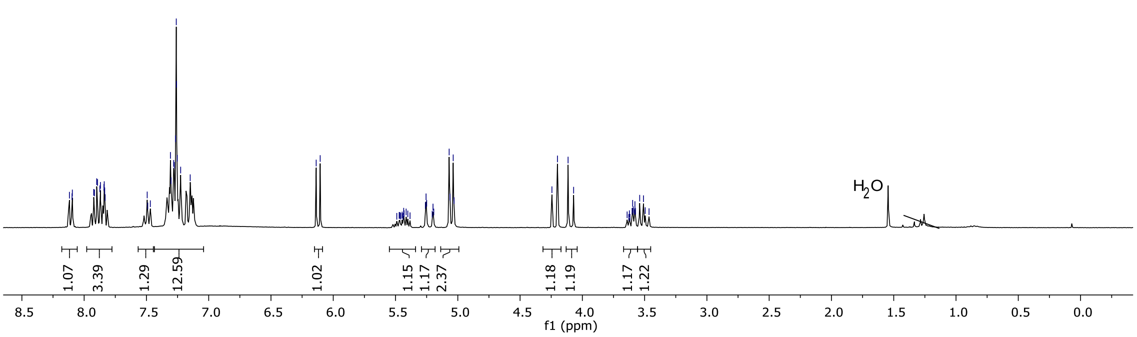

^{13}C{^{1}H} NMR (75 MHz, $CDCl_3$) of **15ec**:

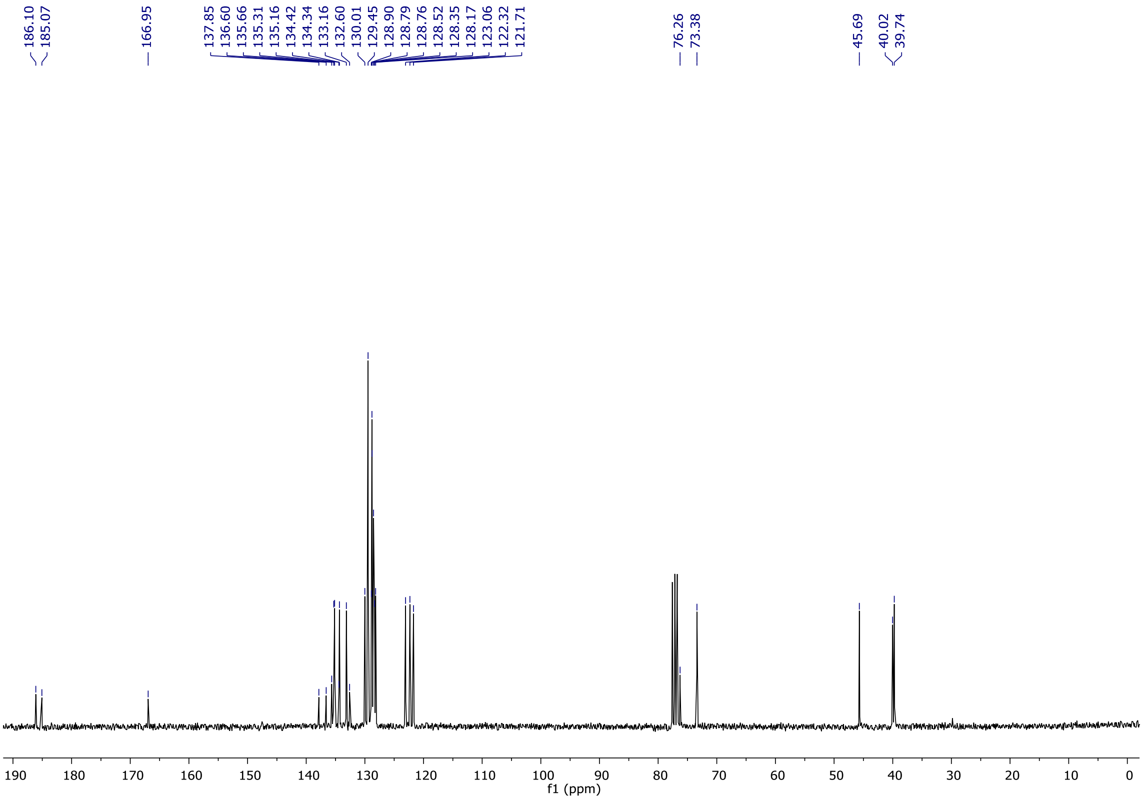

^{1}H NMR (300 MHz, $CDCl_3$) of **15ad**:

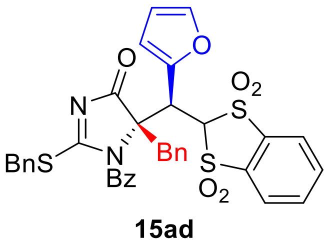

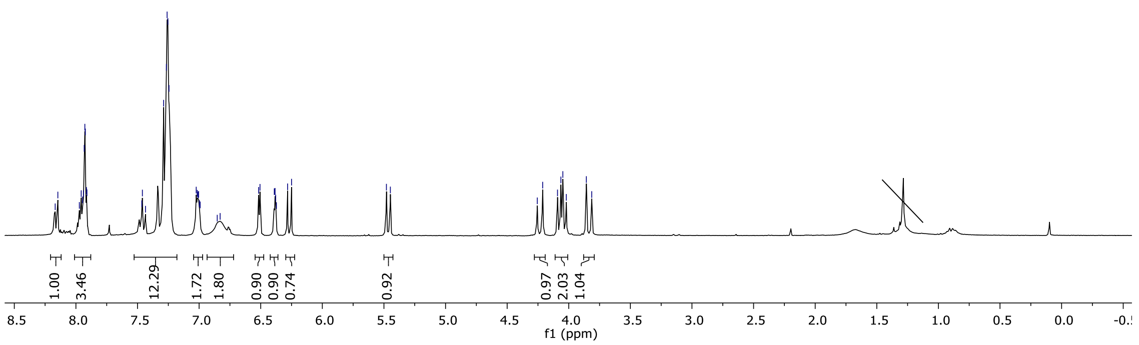

^{13}C{^{1}H} NMR (75 MHz, $CDCl_3$) of **15ad**:

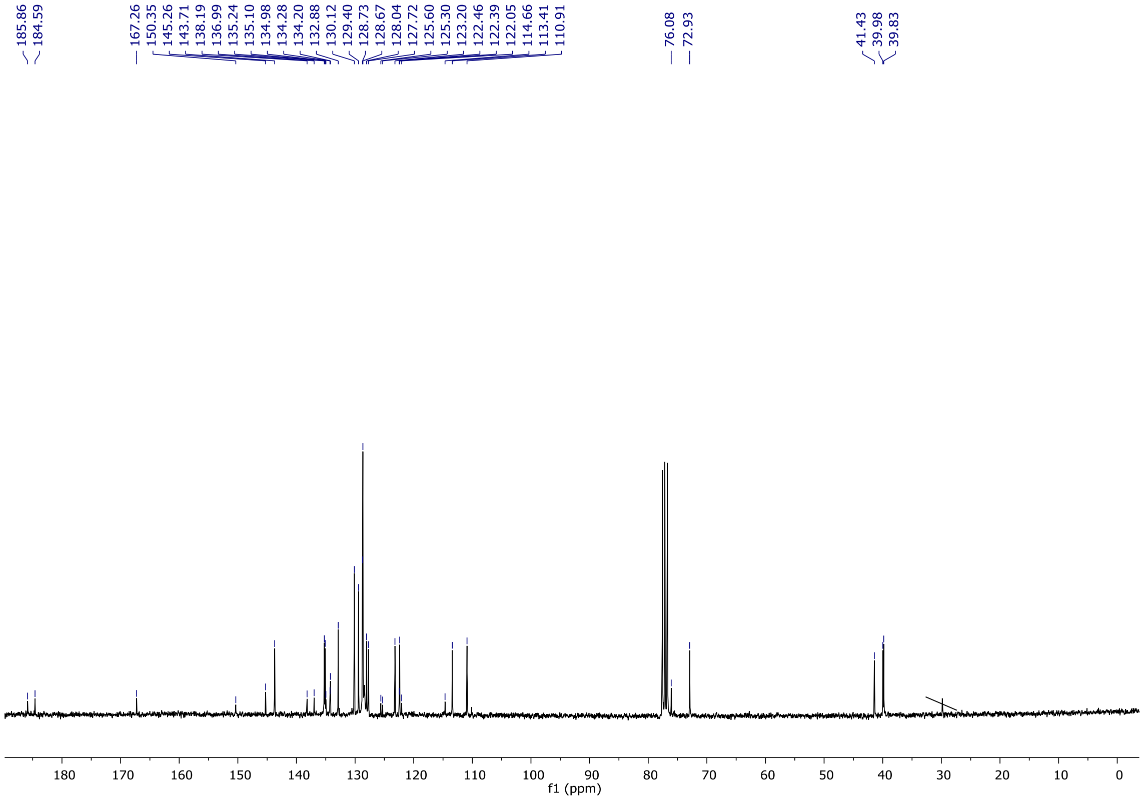

^{1}H NMR (300 MHz, $CDCl_3$) of **15ae**:

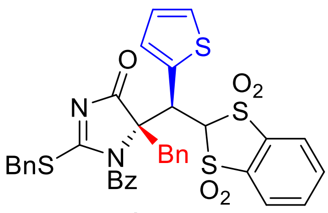

15ae

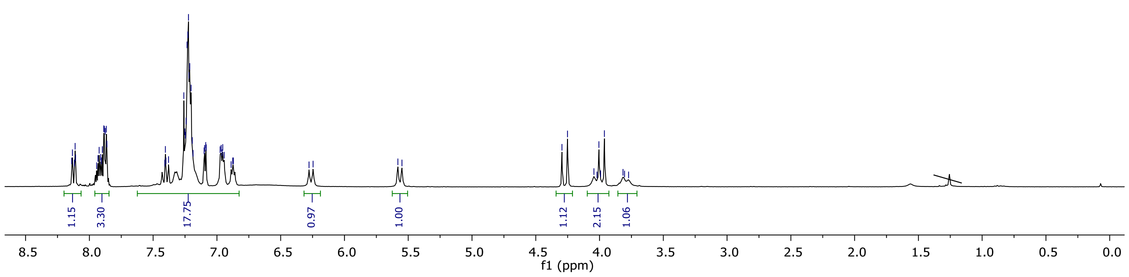

^{13}C{^{1}H} NMR (75 MHz, $CDCl_3$) of **15ae**:

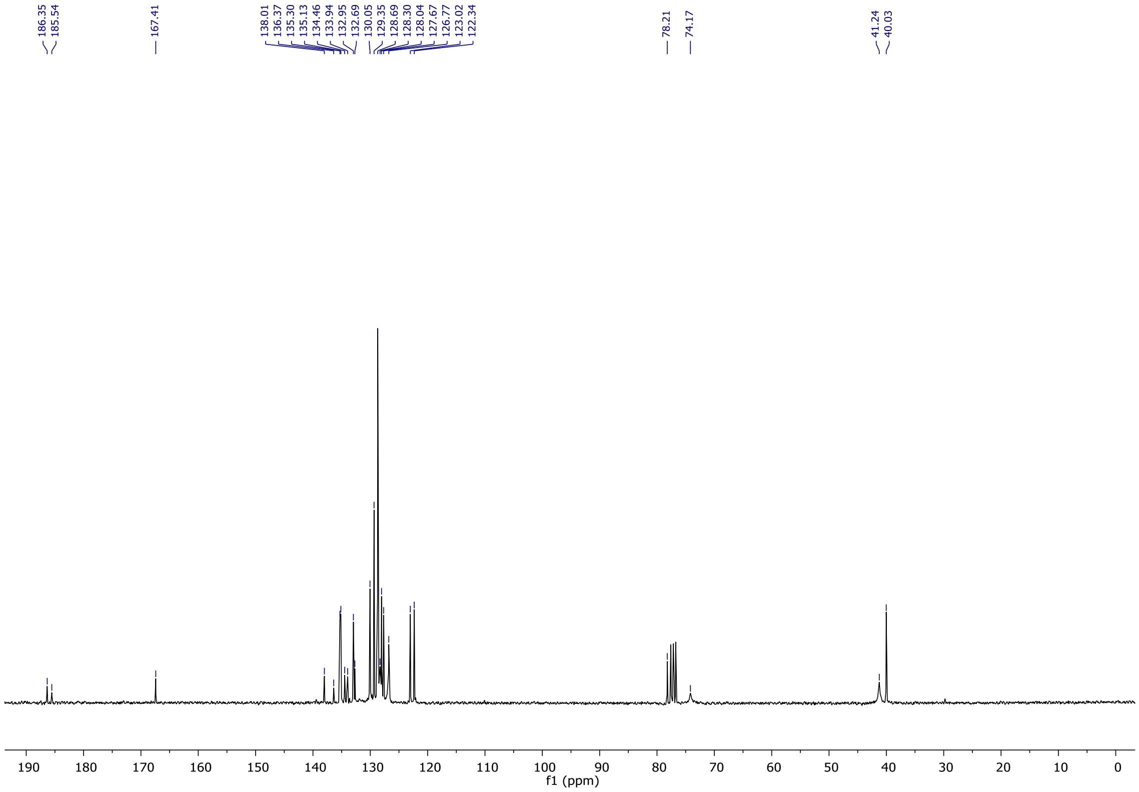

^{1}H NMR (300 MHz, $CDCl_3$) of **15ge**:

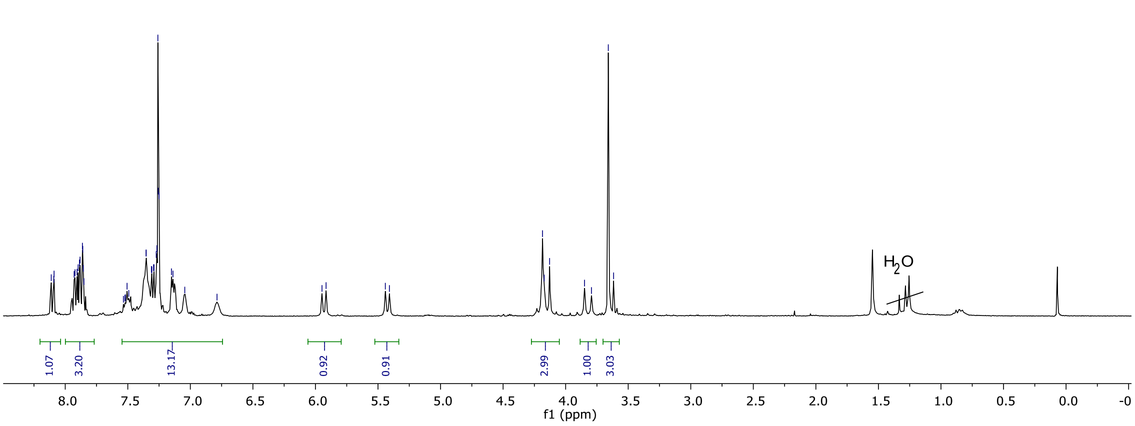

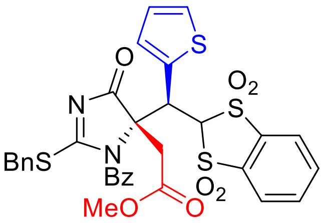

15ge

$^{13}C\{^{1}H\}$ NMR (75 MHz, $CDCl_3$) of **15ge**:

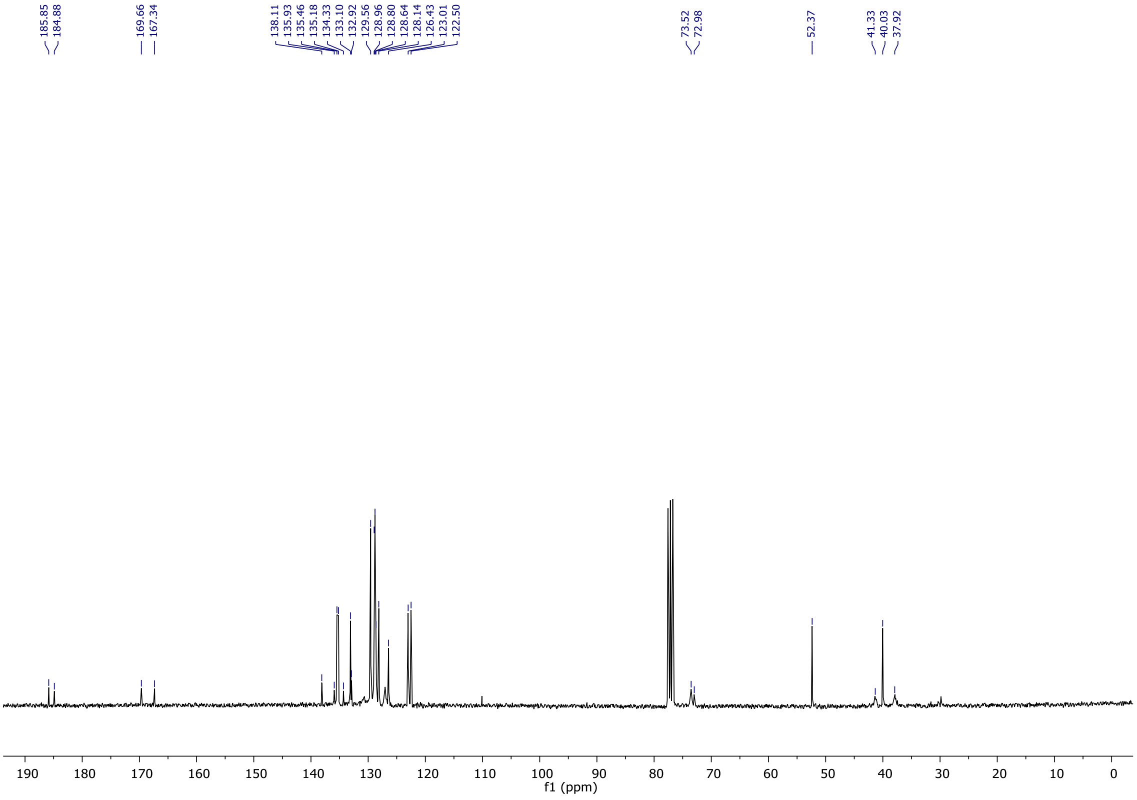

^{1}H NMR (300 MHz, $CDCl_3$) of **15af**:

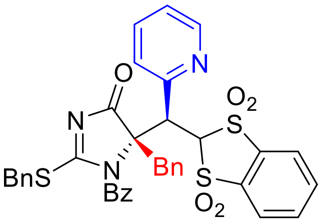

15af

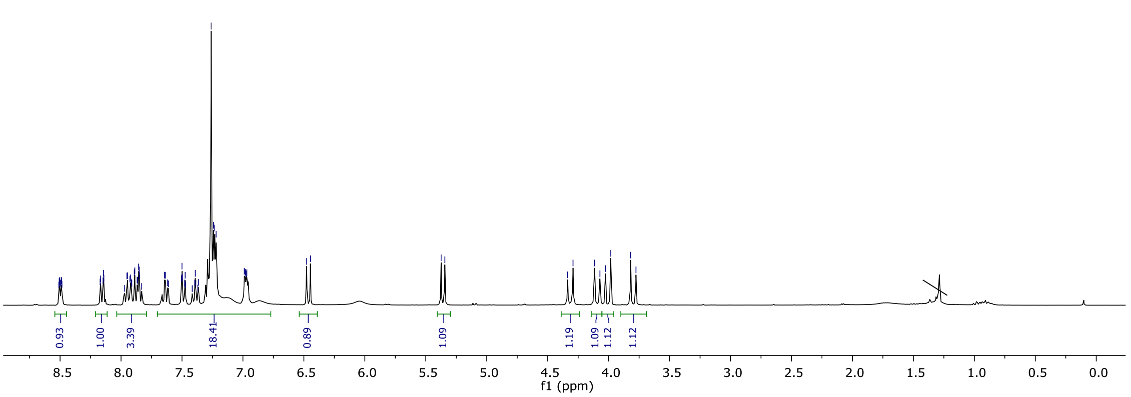

$^{13}C\{^{1}H\}$ NMR (75 MHz, $CDCl_3$) of **15af**:

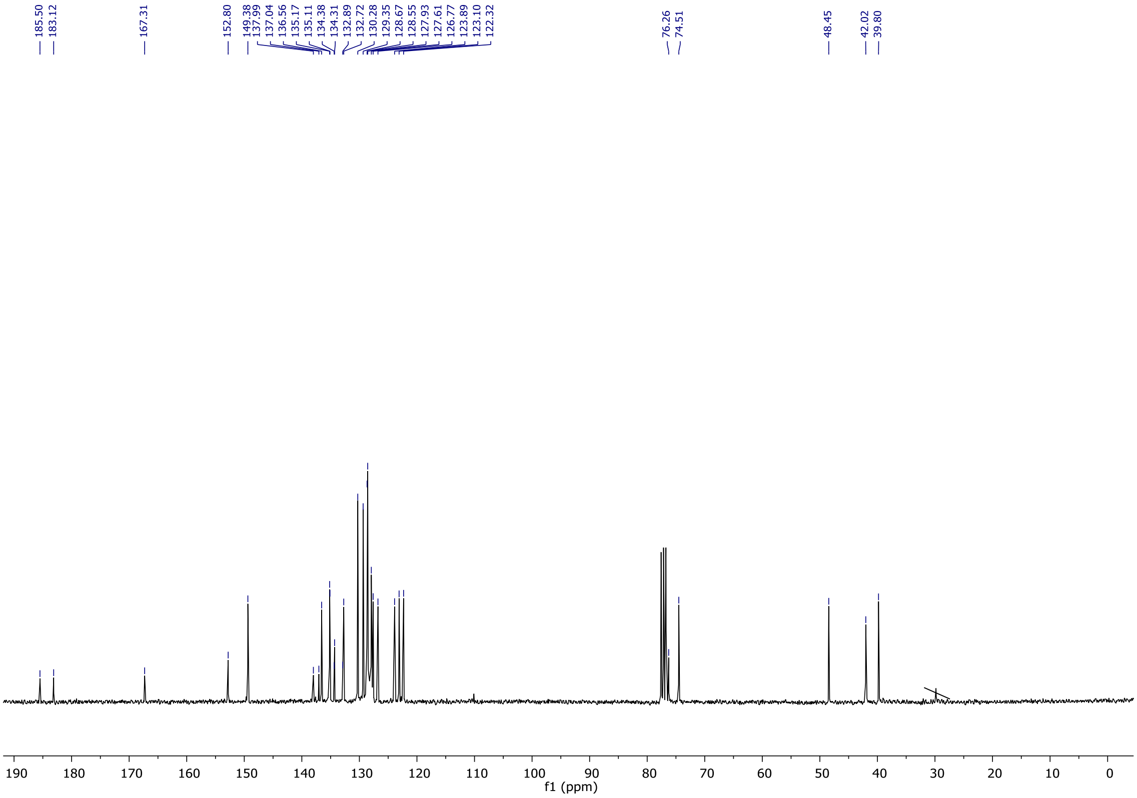

^{1}H NMR (300 MHz, $CDCl_3$) of **15eg**

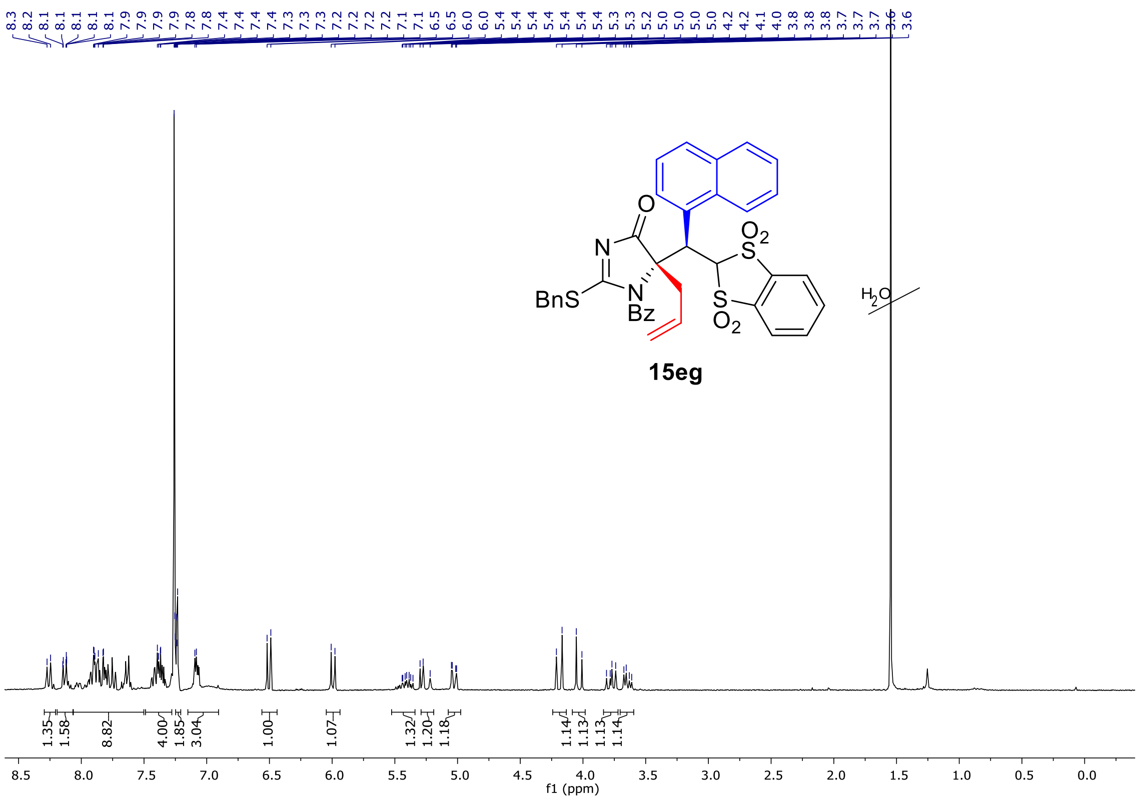

$^{13}C\{^{1}H\}$ NMR (75 MHz, $CDCl_3$) of **15eg:**

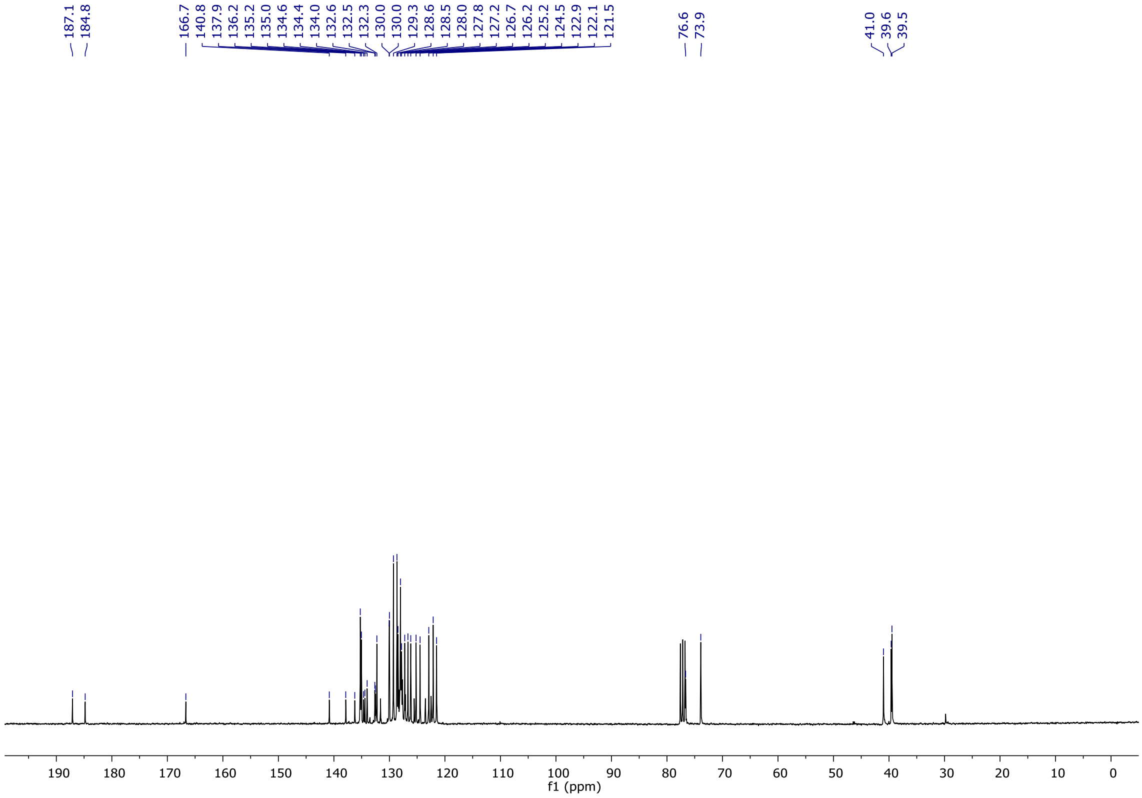

^{1}H NMR (300 MHz, $CDCl_3$) of **18**:

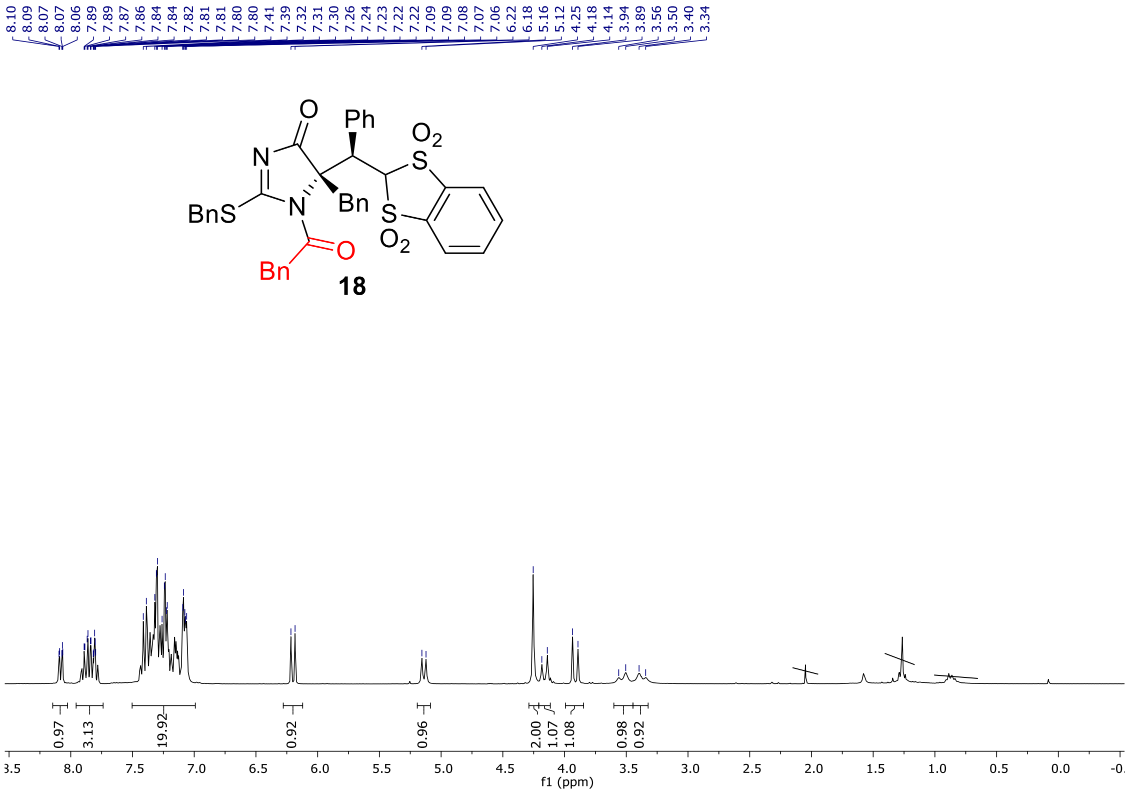

^{13}C{^{1}H} NMR (75 MHz, $CDCl_3$) of **18**:

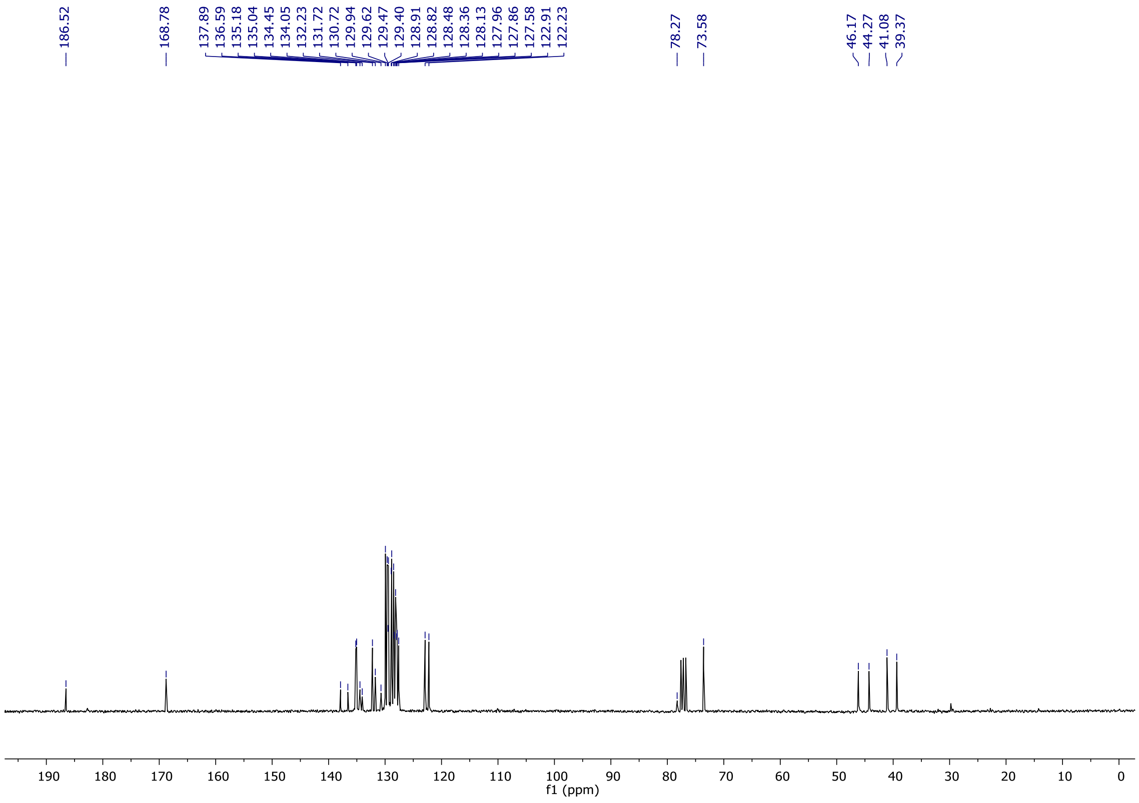

^{1}H NMR (300 MHz, $CDCl_3$) of **21**:

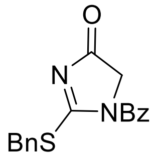

21

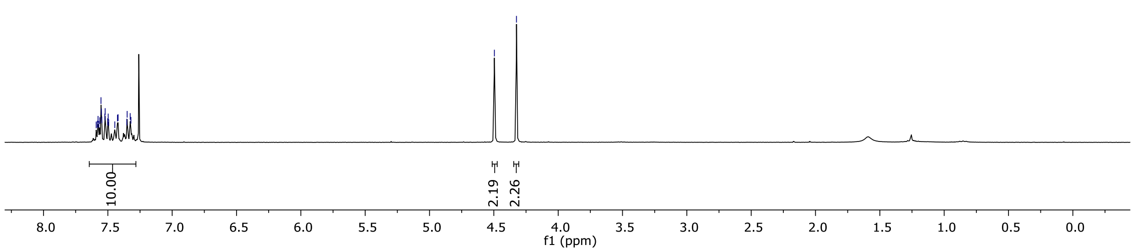

$^{13}C\{^{1}H\}$ NMR (75 MHz, $CDCl_3$) of **21**:

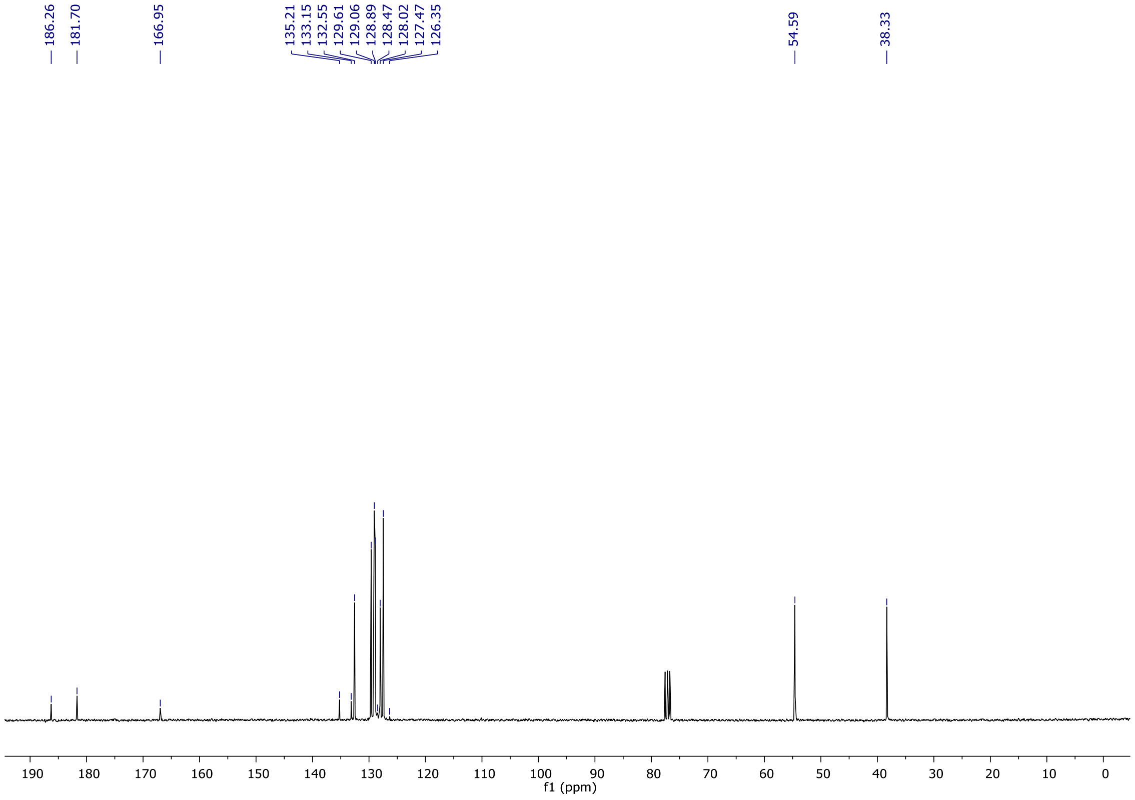

^{1}H NMR (300 MHz, $CDCl_3$) of **22a**:

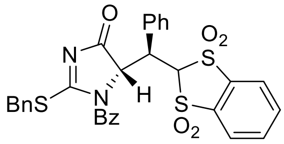

22a

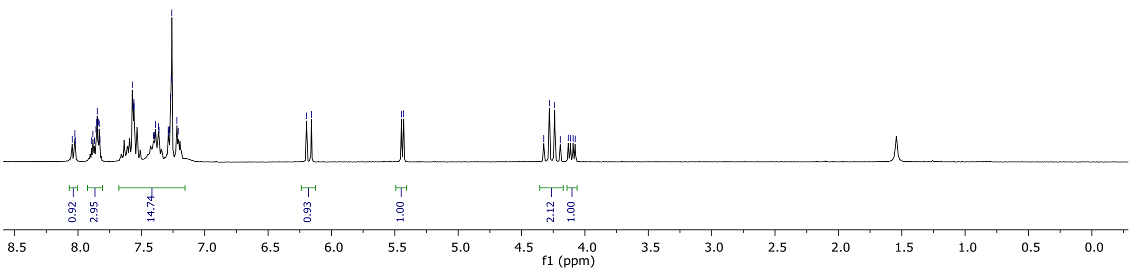

$^{13}C\{^{1}H\}$ NMR (75 MHz, CD_2Cl_2) of **22a**:

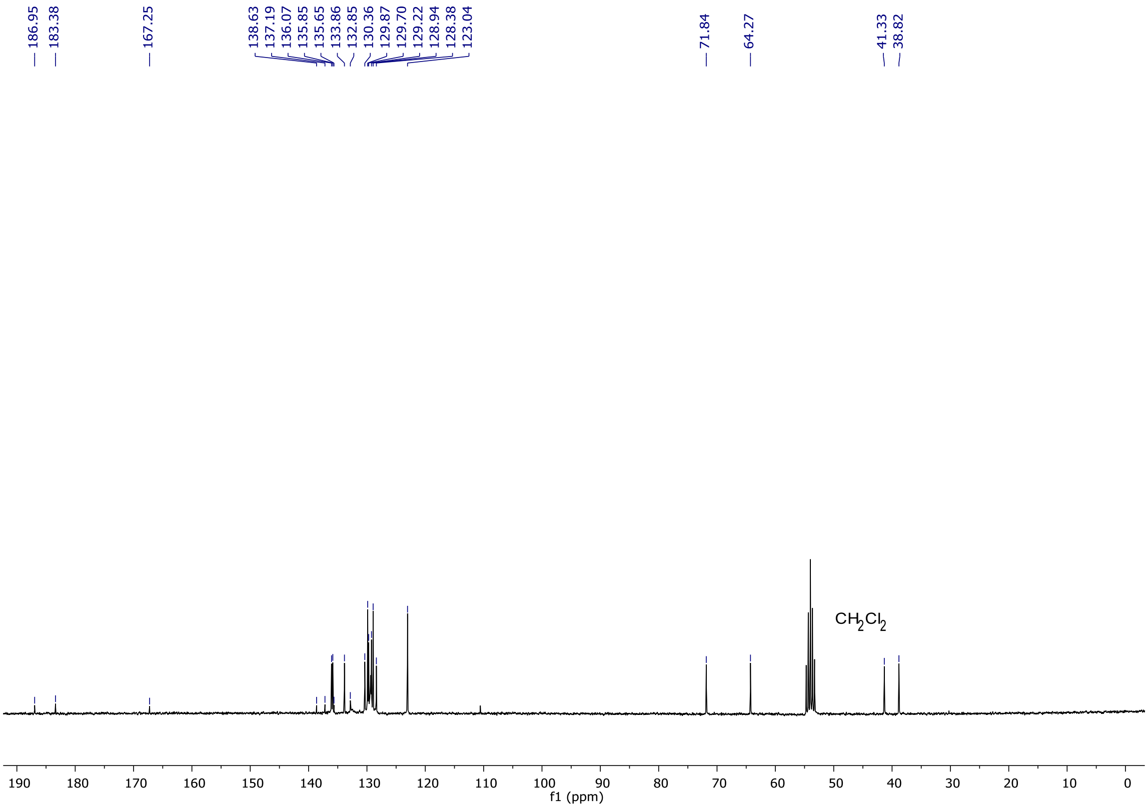

^{1}H NMR (300 MHz, CD_2Cl_2) of **22b**:

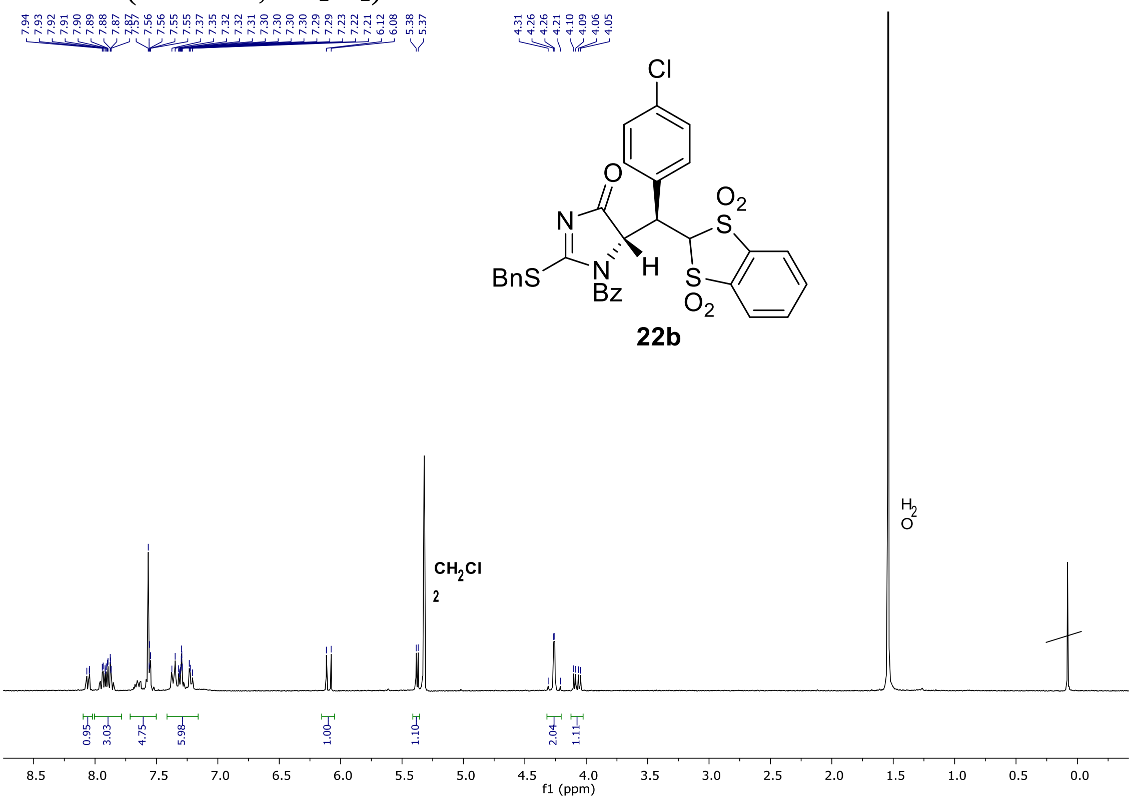

^{13}C{^{1}H} NMR (126 MHz, CD_2Cl_2) of **22b**:

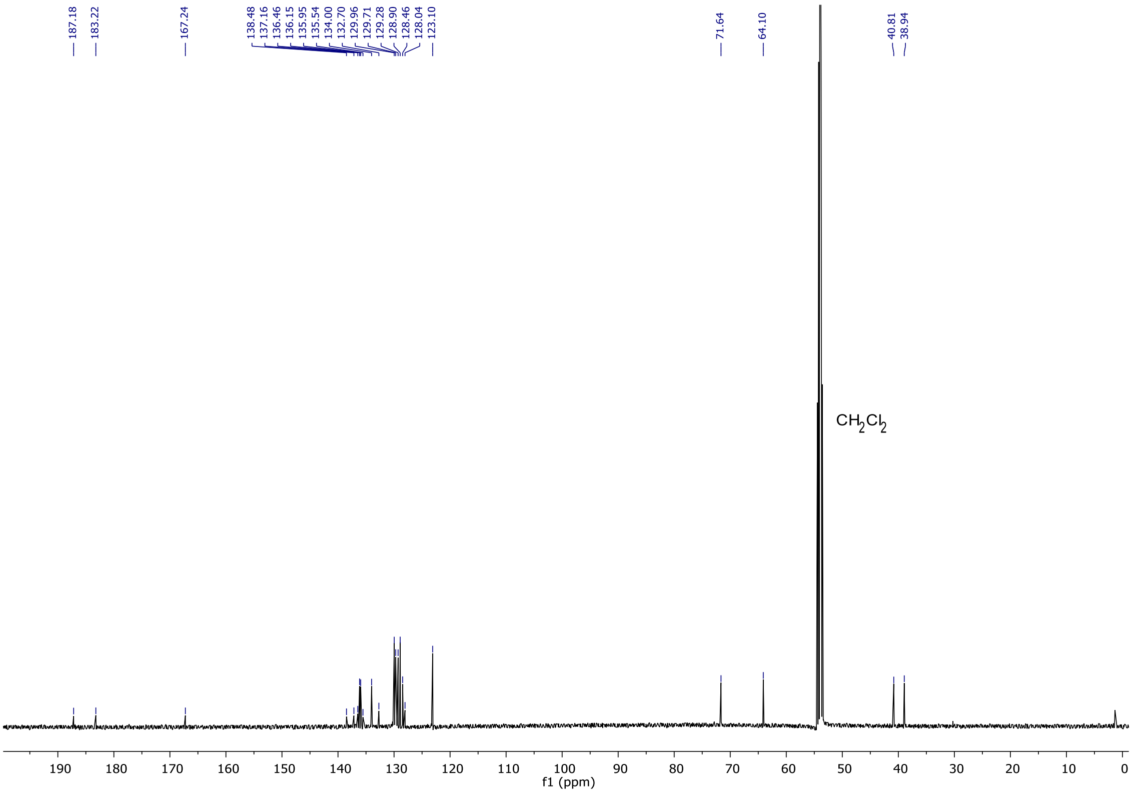

^{1}H NMR (300 MHz, CDCl$_3$) of **23**:

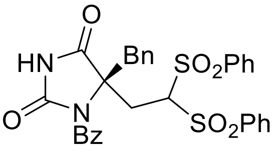

23

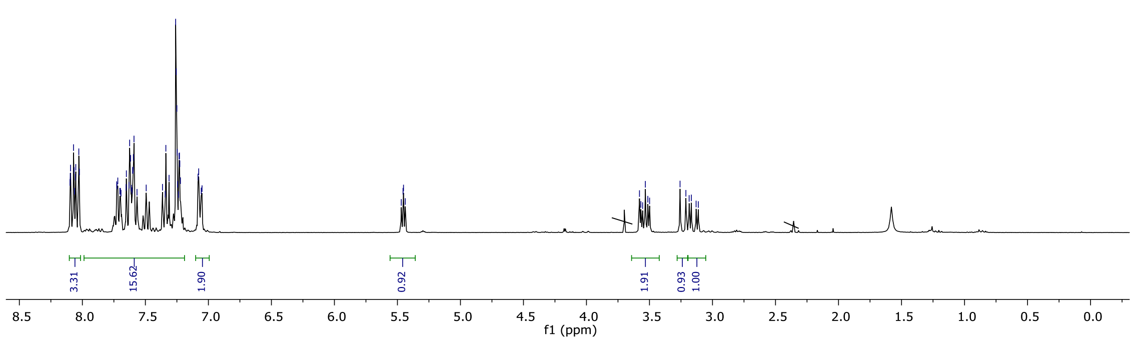

^{13}C{^{1}H} NMR (75 MHz, CDCl$_3$) of **23**:

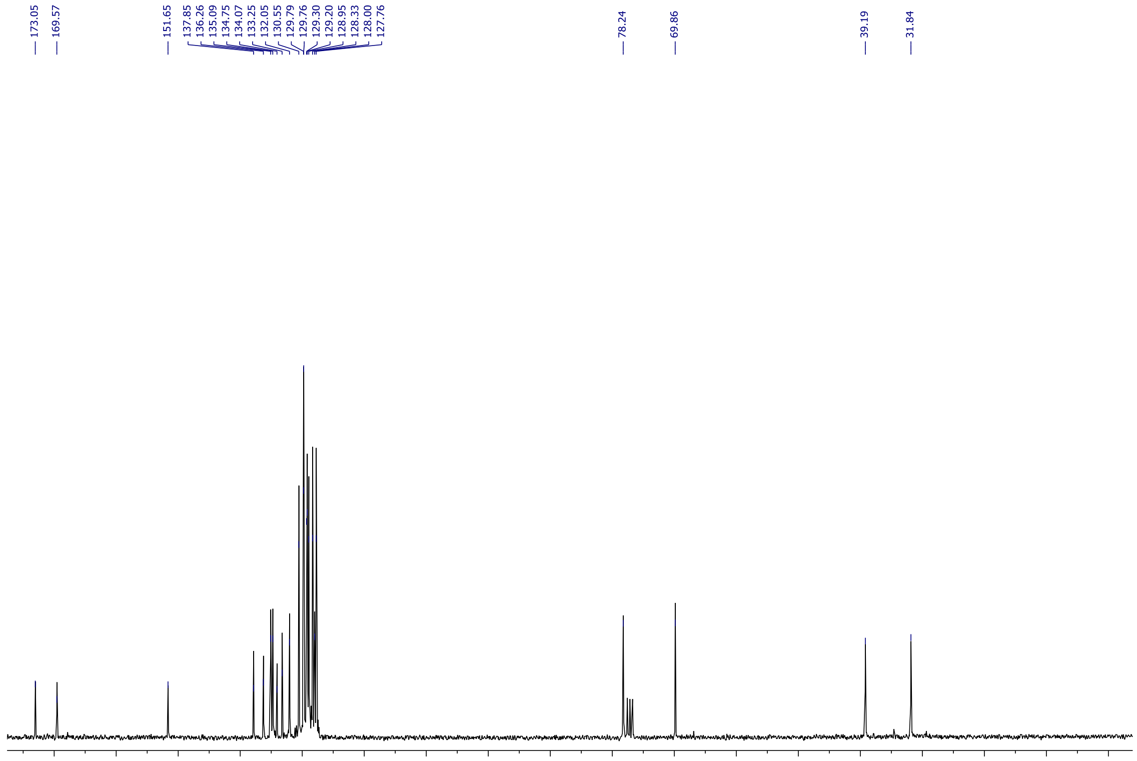

^{1}H NMR (300 MHz, CD_2Cl_2) of **24**:

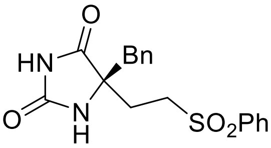

24

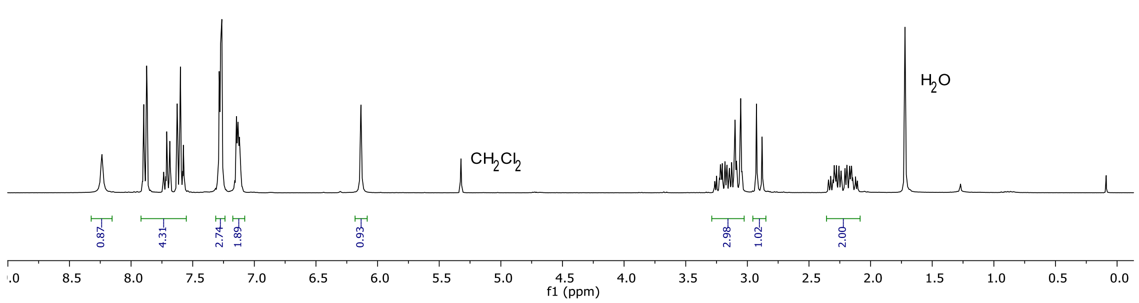

^{13}C{^{1}H} NMR (75 MHz, CD_2Cl_2) of **24**:

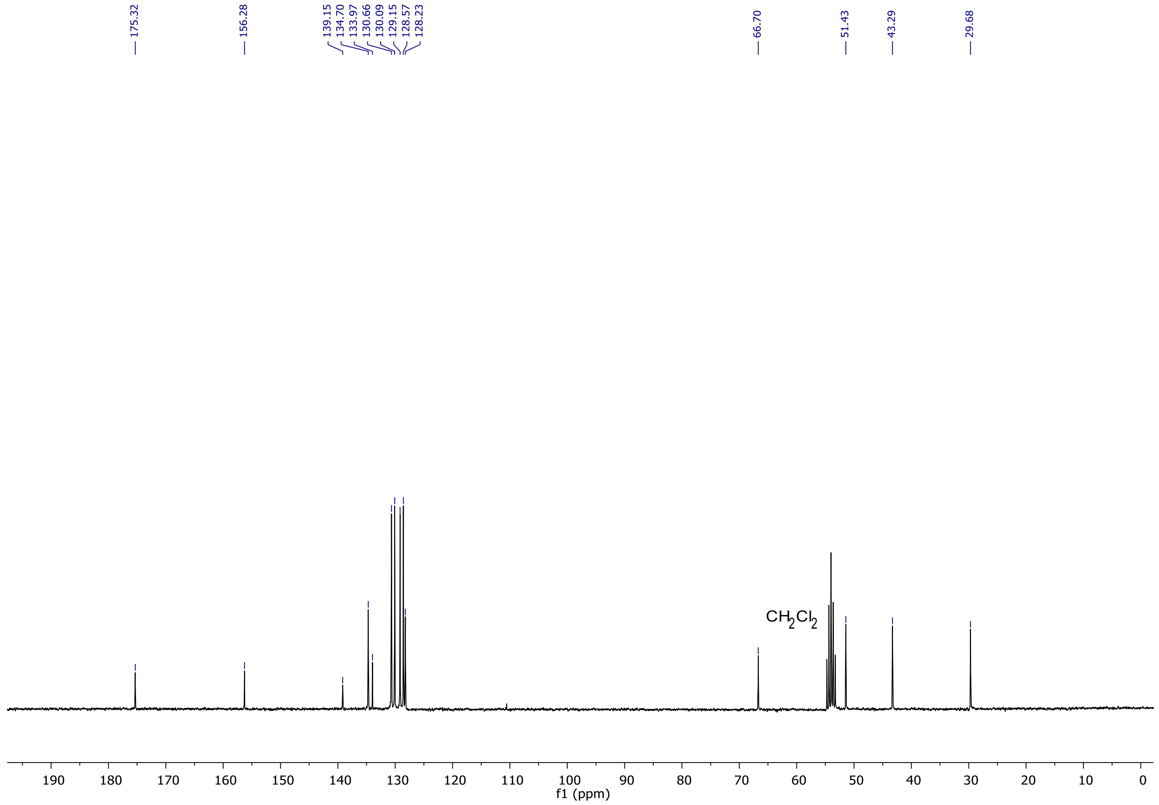

^{1}H NMR (300 MHz, CD_2Cl_2) of **25b**:

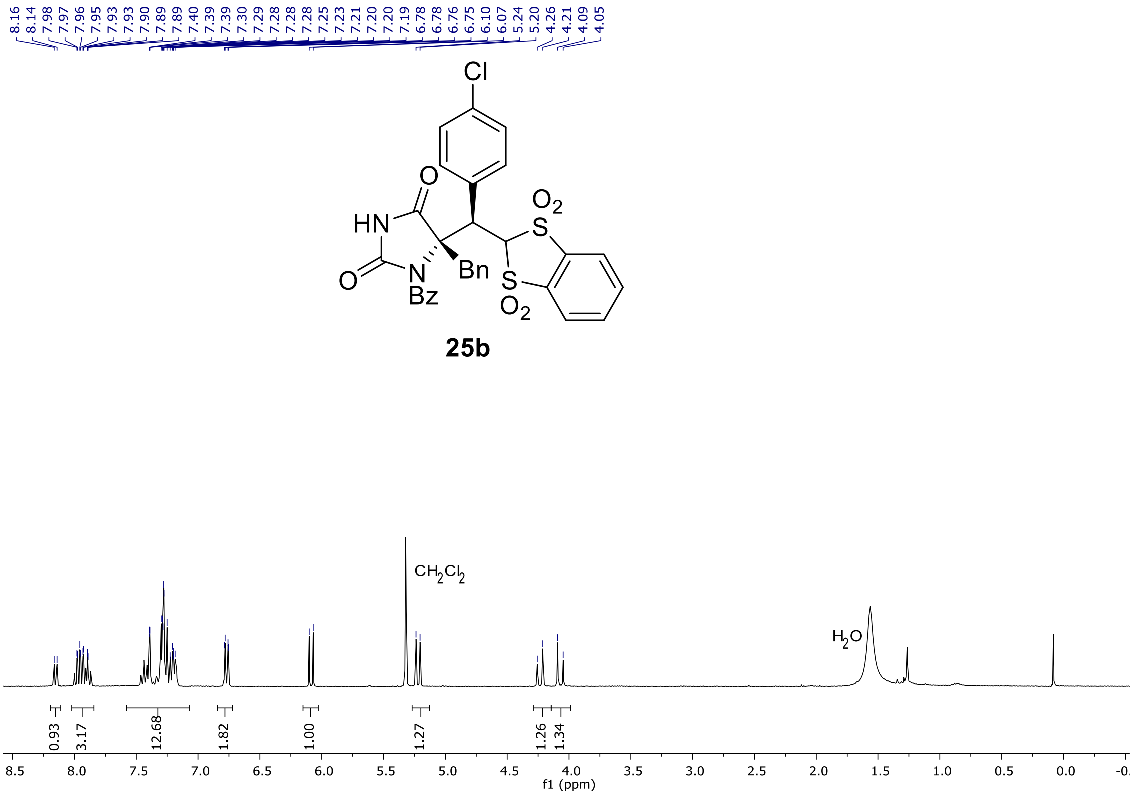

25b

$^{13}C\{^{1}H\}$ NMR (75 MHz, CD_2Cl_2) of **25b**:

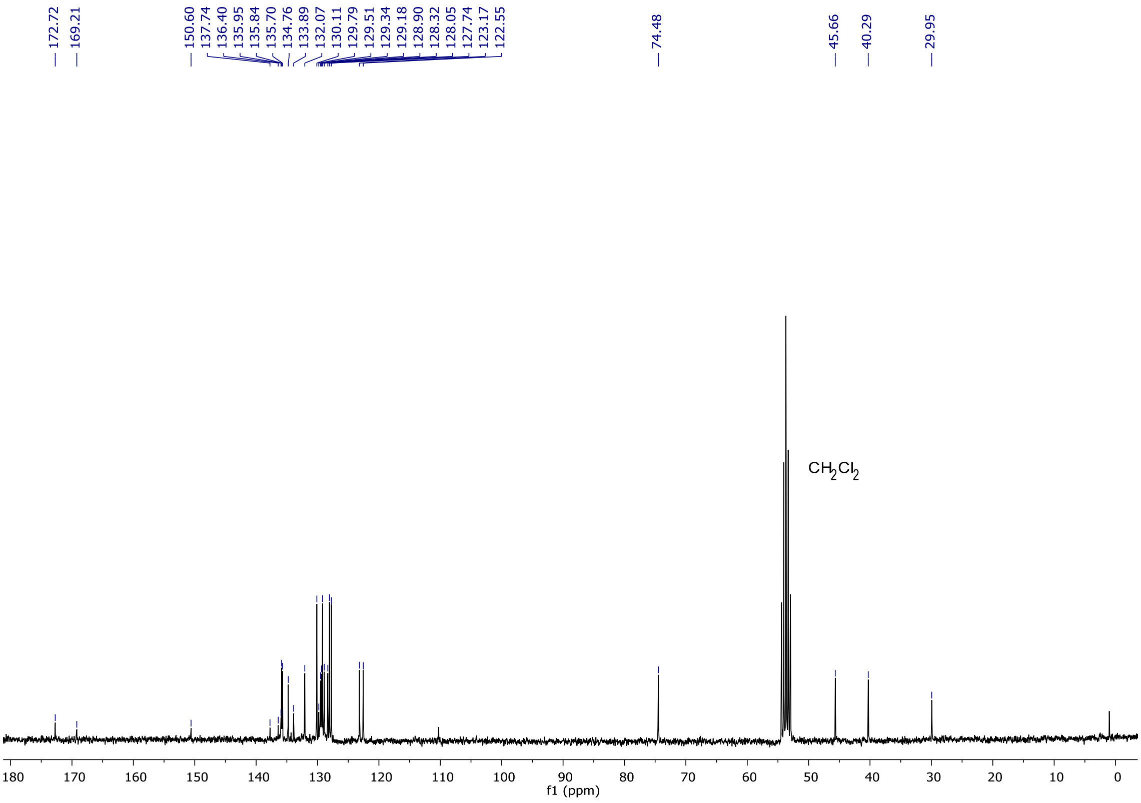

^{1}H NMR (300 MHz, $CDCl_3$) of **26**:

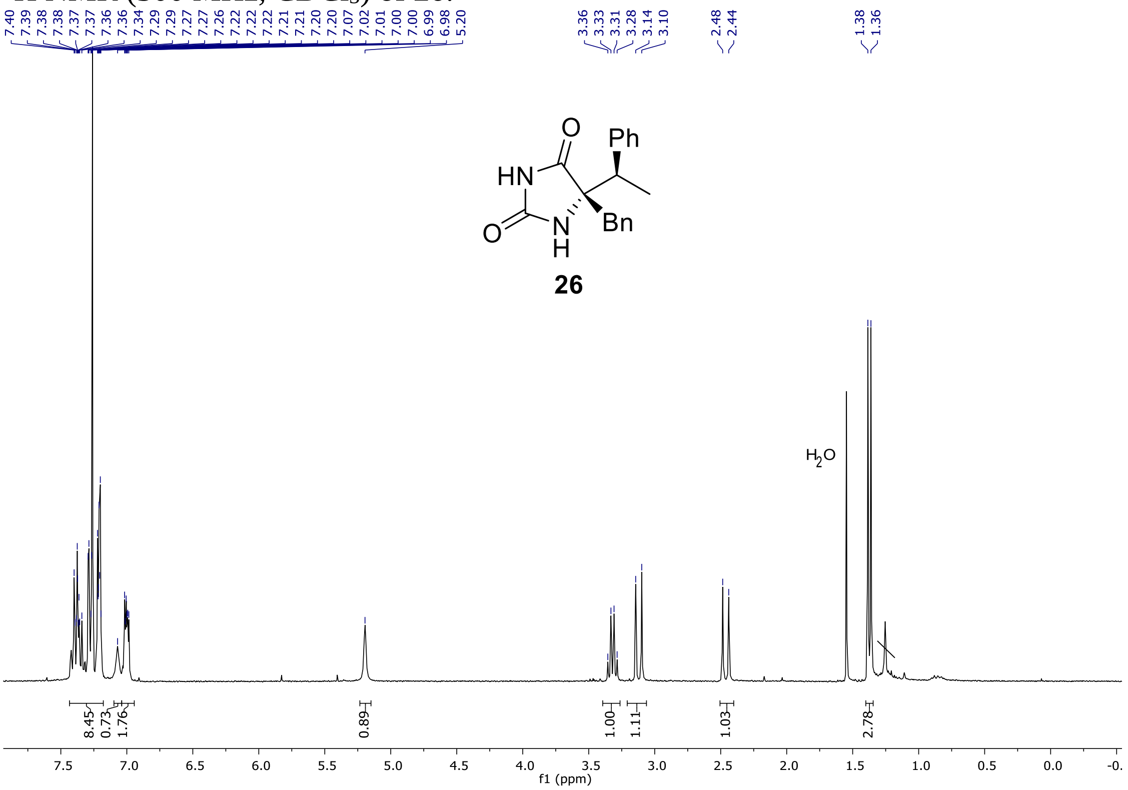

^{13}C{^{1}H} NMR (126 MHz, $CDCl_3$) of **26**:

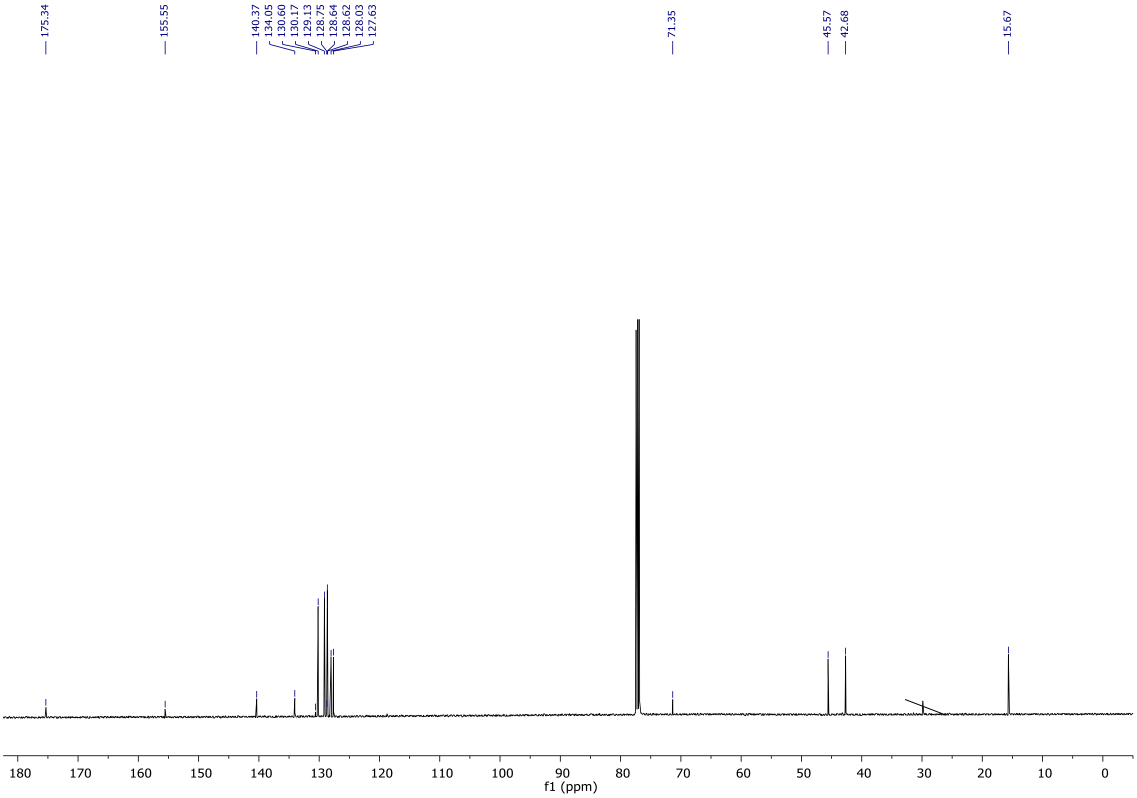

^{1}H NMR (300 MHz, $CDCl_3$) of **27**:

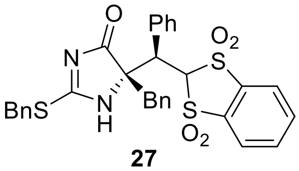

27

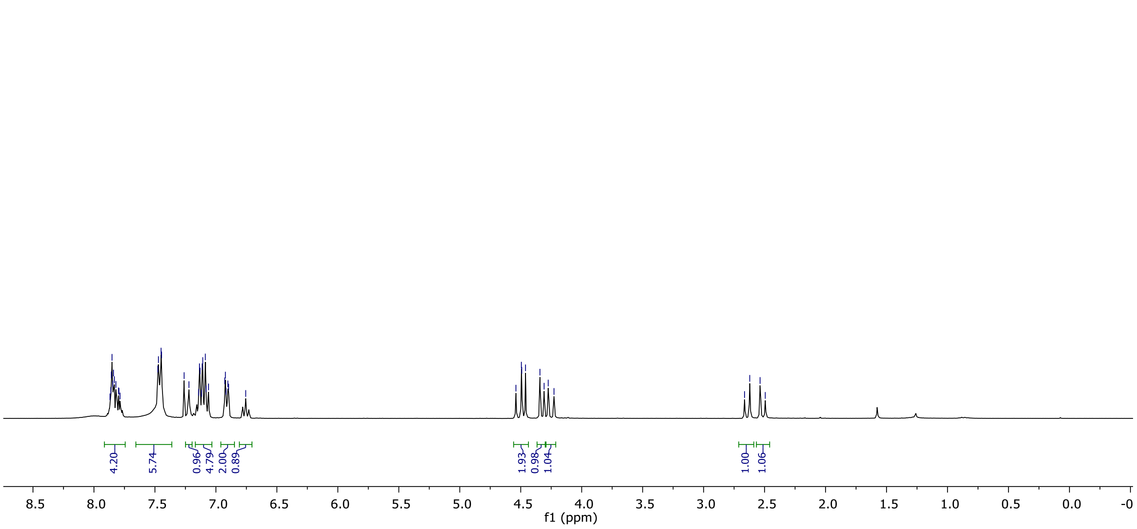

^{13}C{^{1}H} NMR (75 MHz, $CDCl_3$) of **27**:

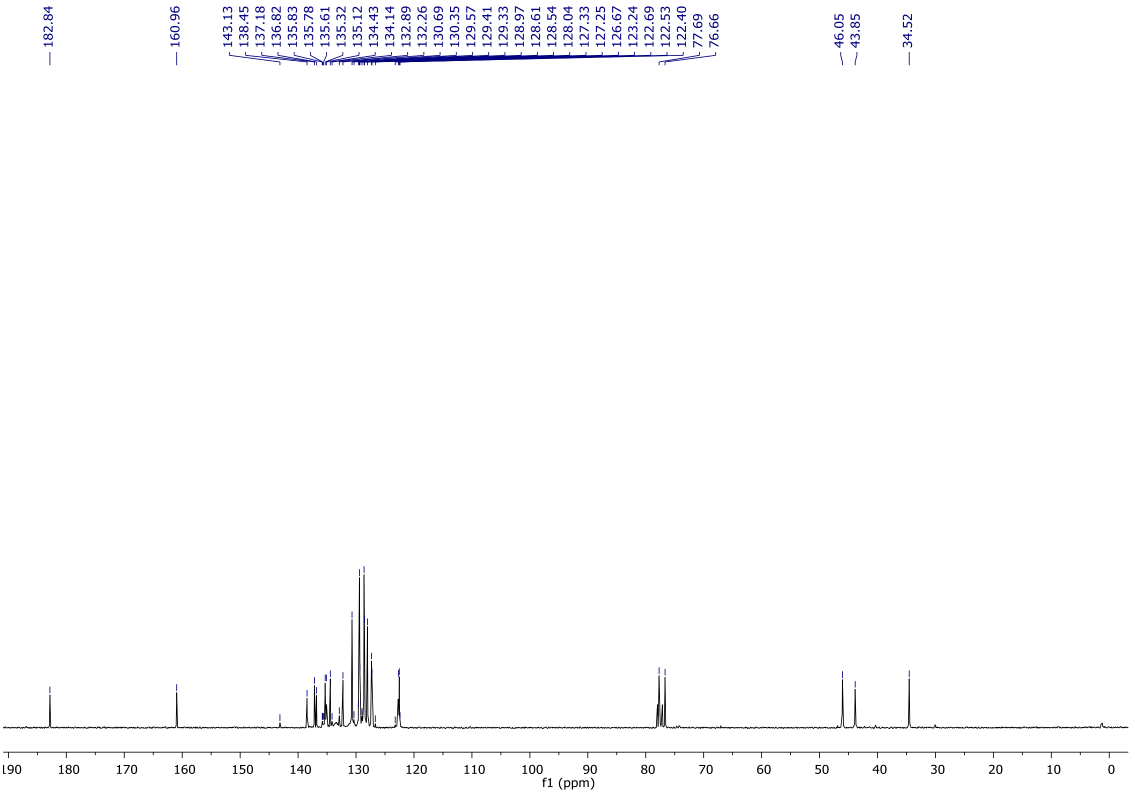

^{1}H NMR (300 MHz, CD_2Cl_2) of **28**:

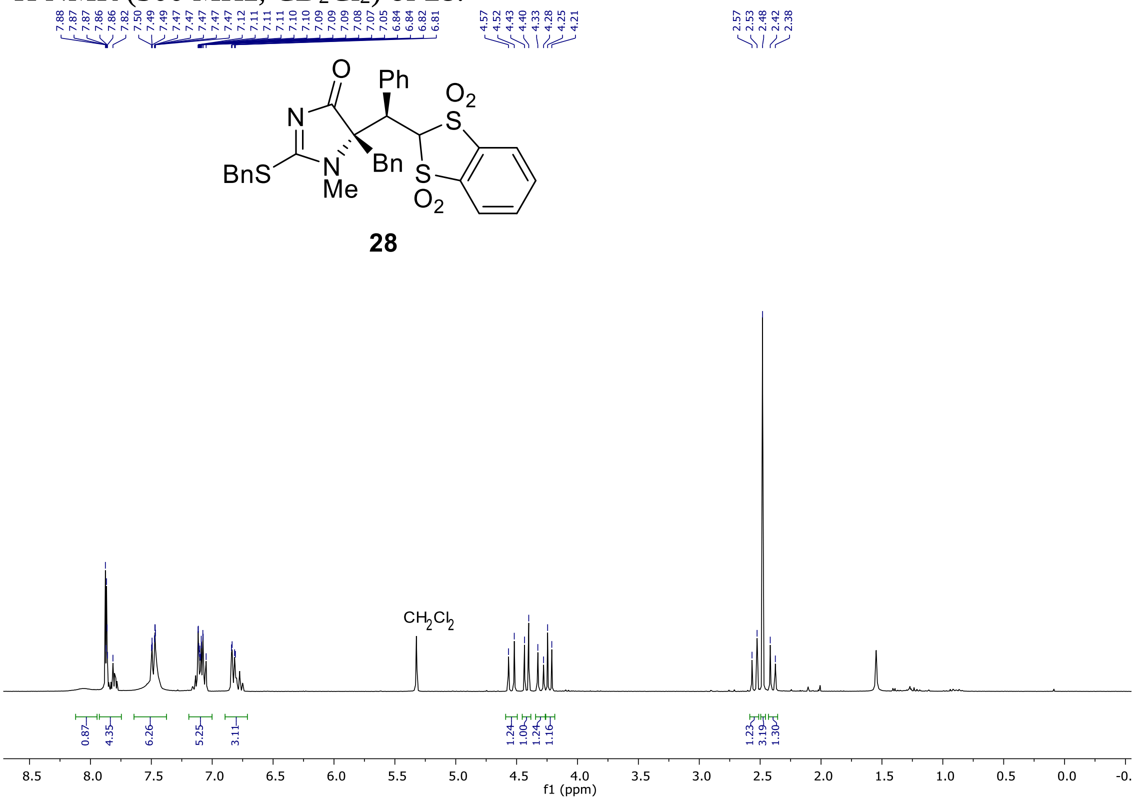

^{13}C{^{1}H} NMR (75 MHz, CD_2Cl_2) of **28**:

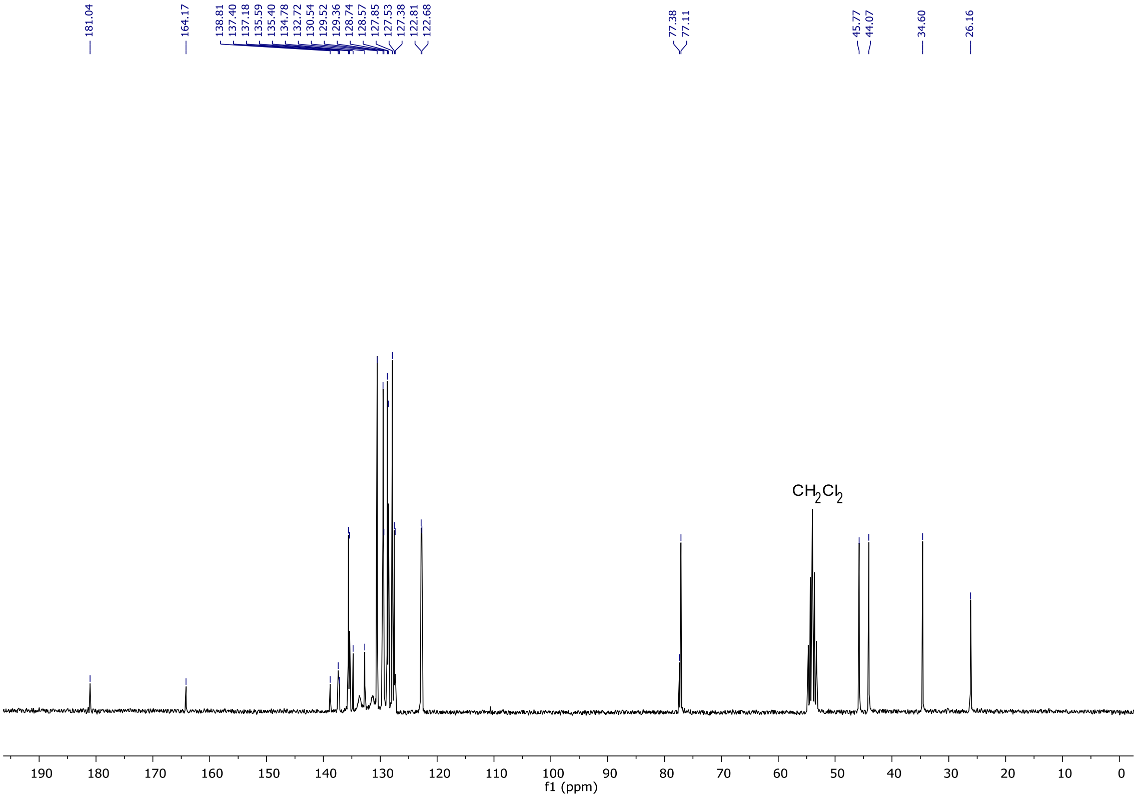

^{1}H NMR (300 MHz, $CDCl_3$) of **29**:

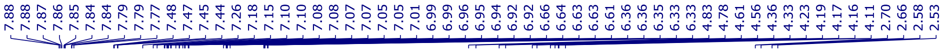

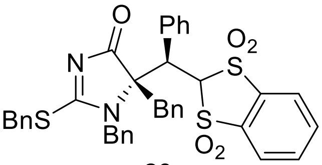

29

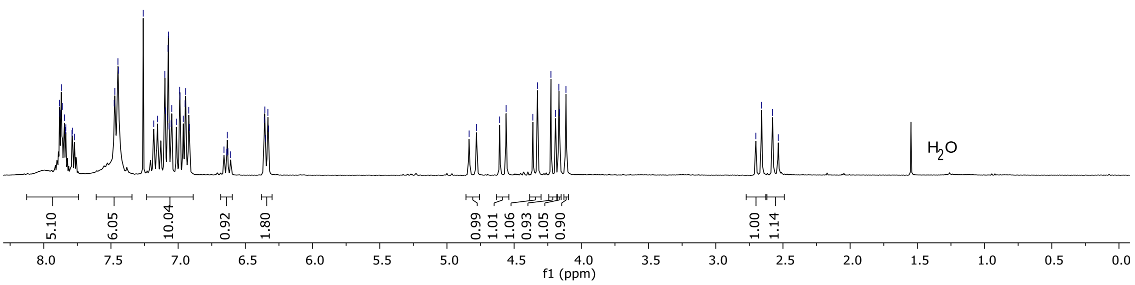

^{13}C{^{1}H} NMR (75 MHz, $CDCl_3$) of **29**:

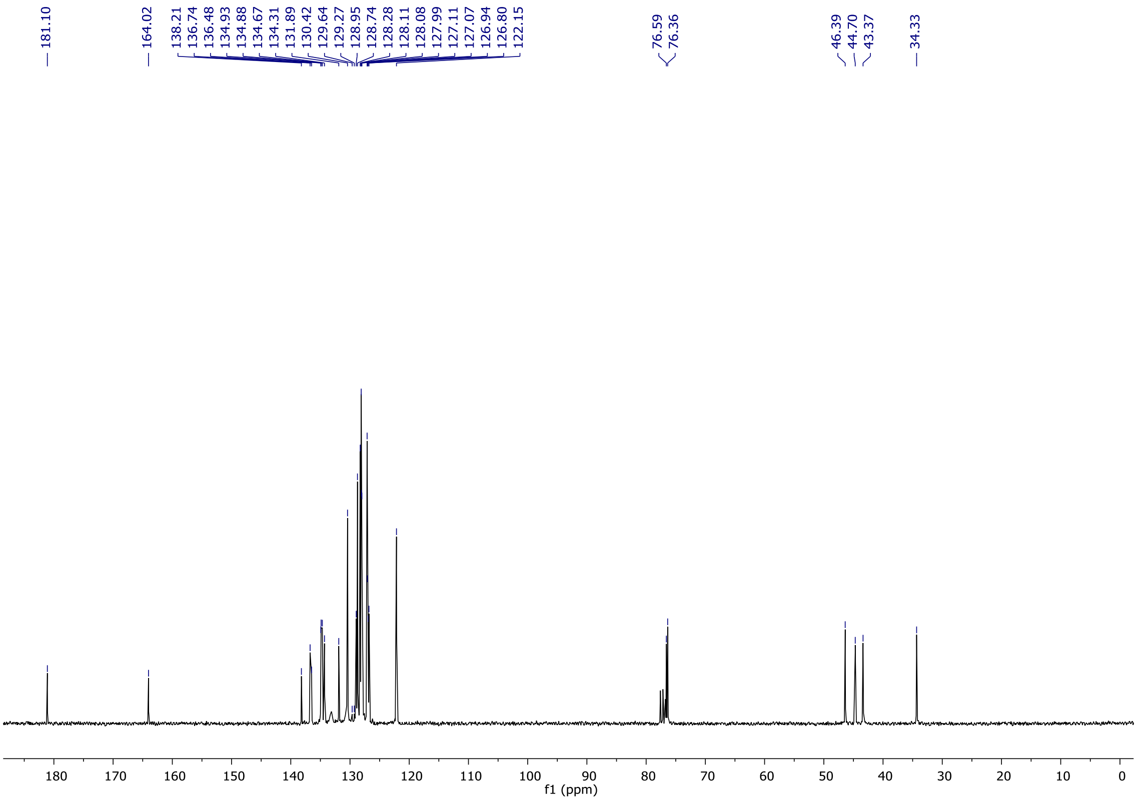

^{1}H NMR (300 MHz, $CDCl_3$) of **30**:

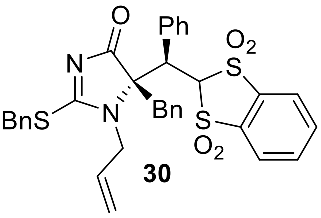

30

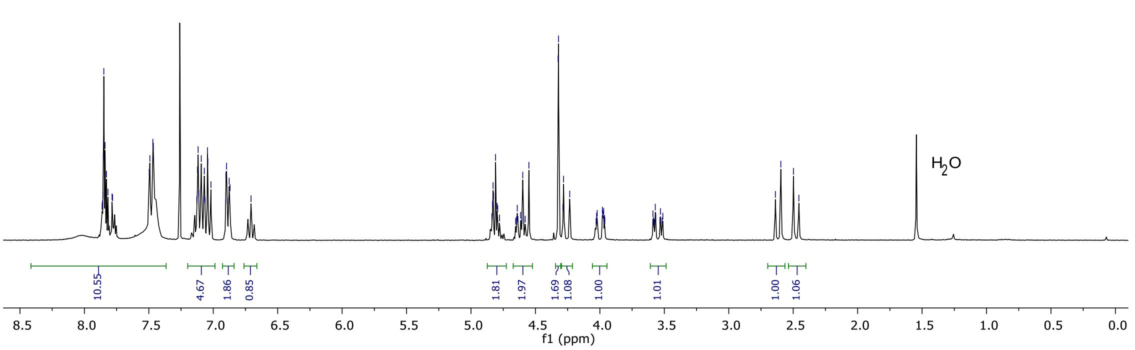

^{13}C{^{1}H} NMR (75 MHz, $CDCl_3$) of **30**:

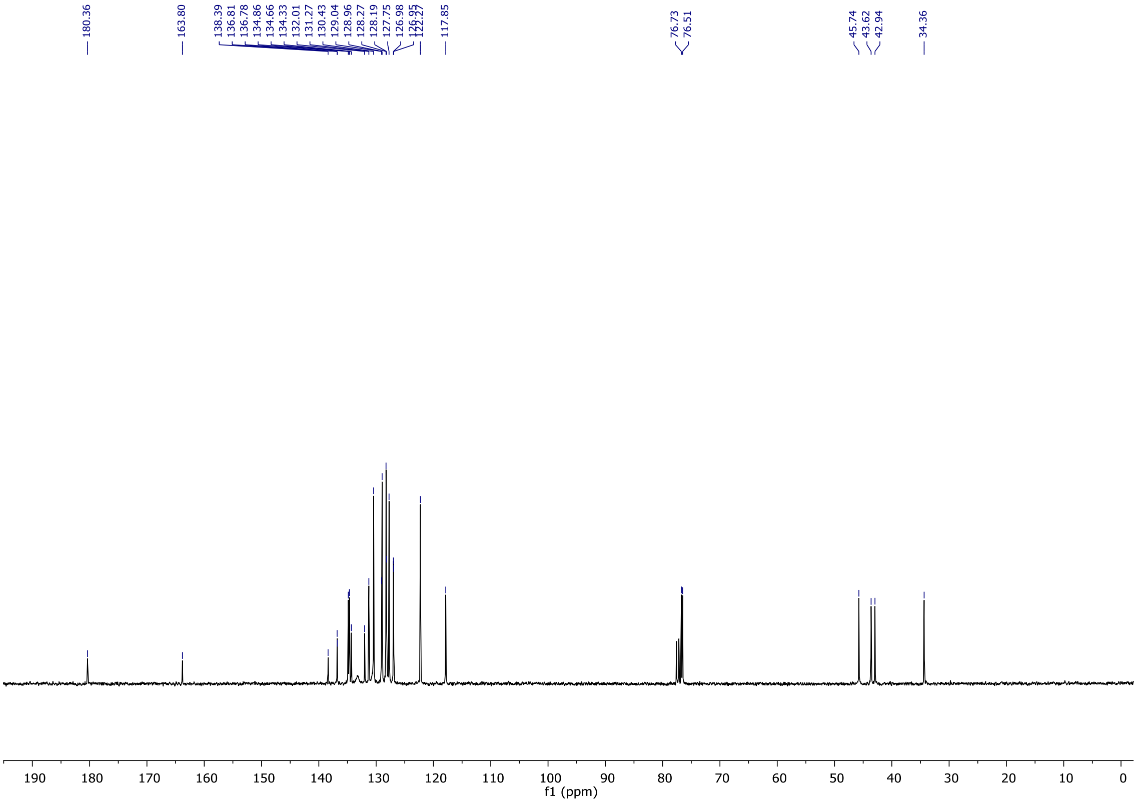

^{1}H NMR (300 MHz, CD_2Cl_2) of **32a**:

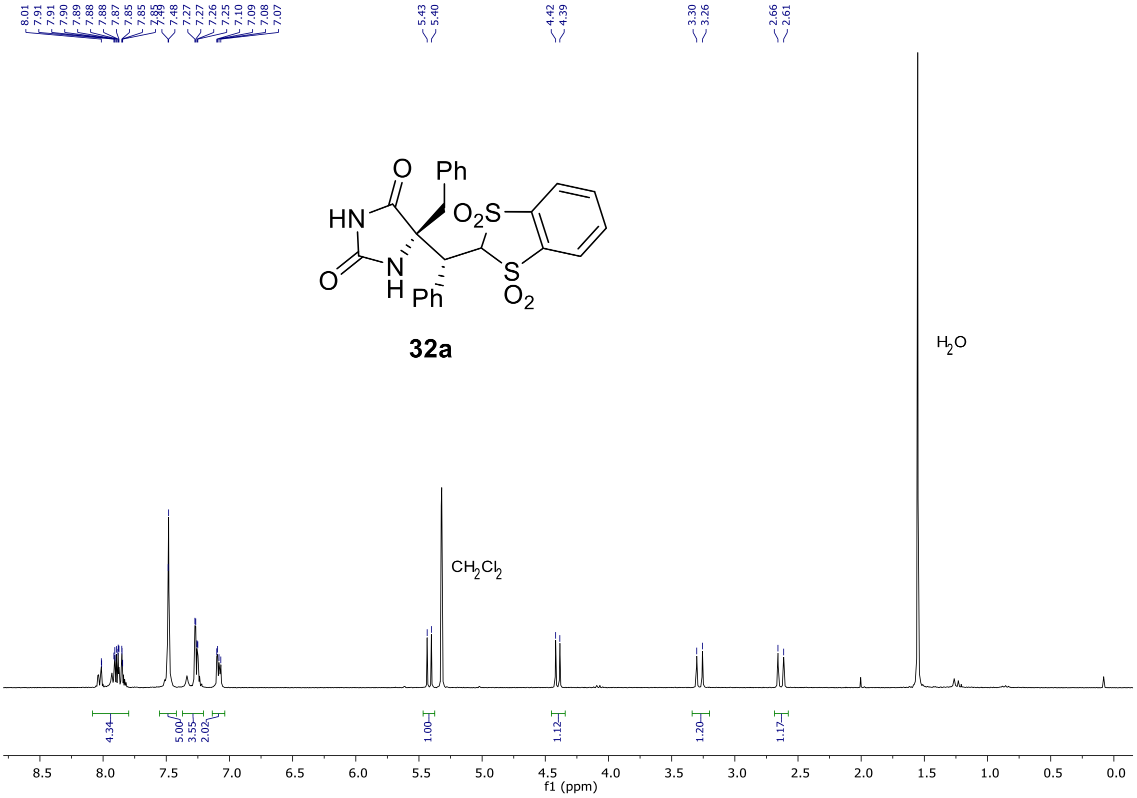

^{13}C{^{1}H} NMR (126 MHz, CD_2Cl_2) of **32a**:

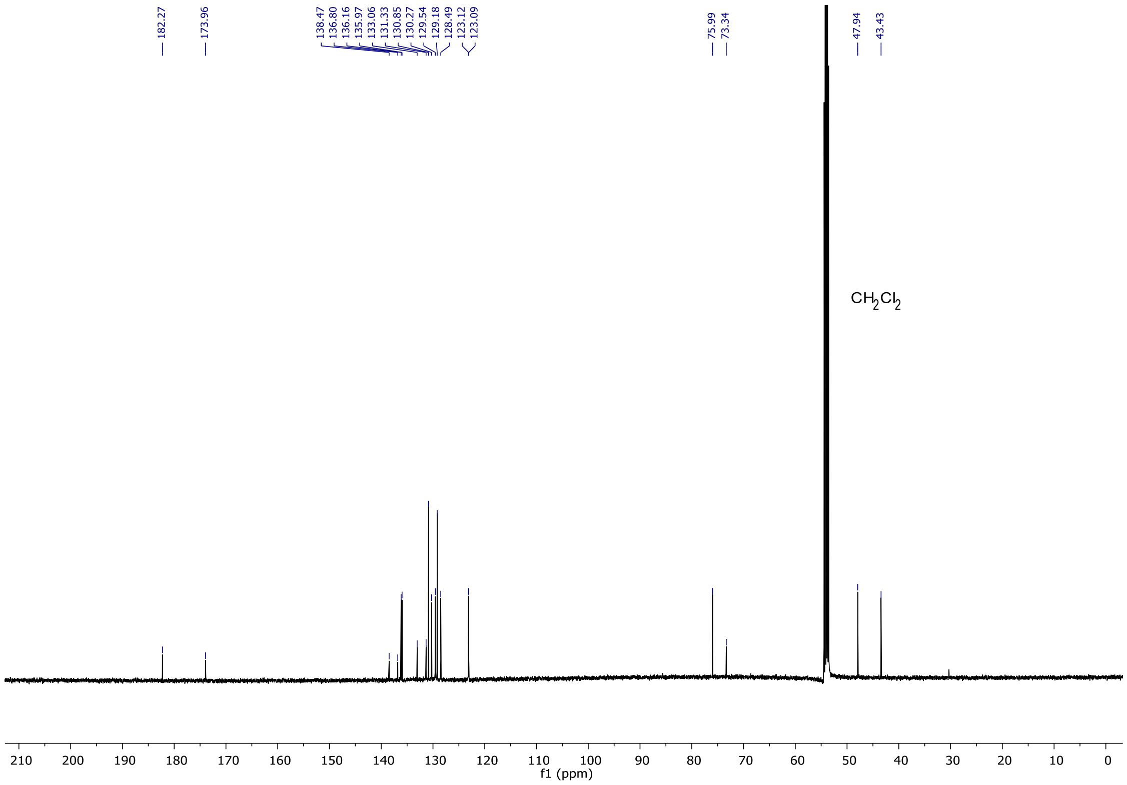

^{1}H NMR (300 MHz, CD_2Cl_2) of **32b**:

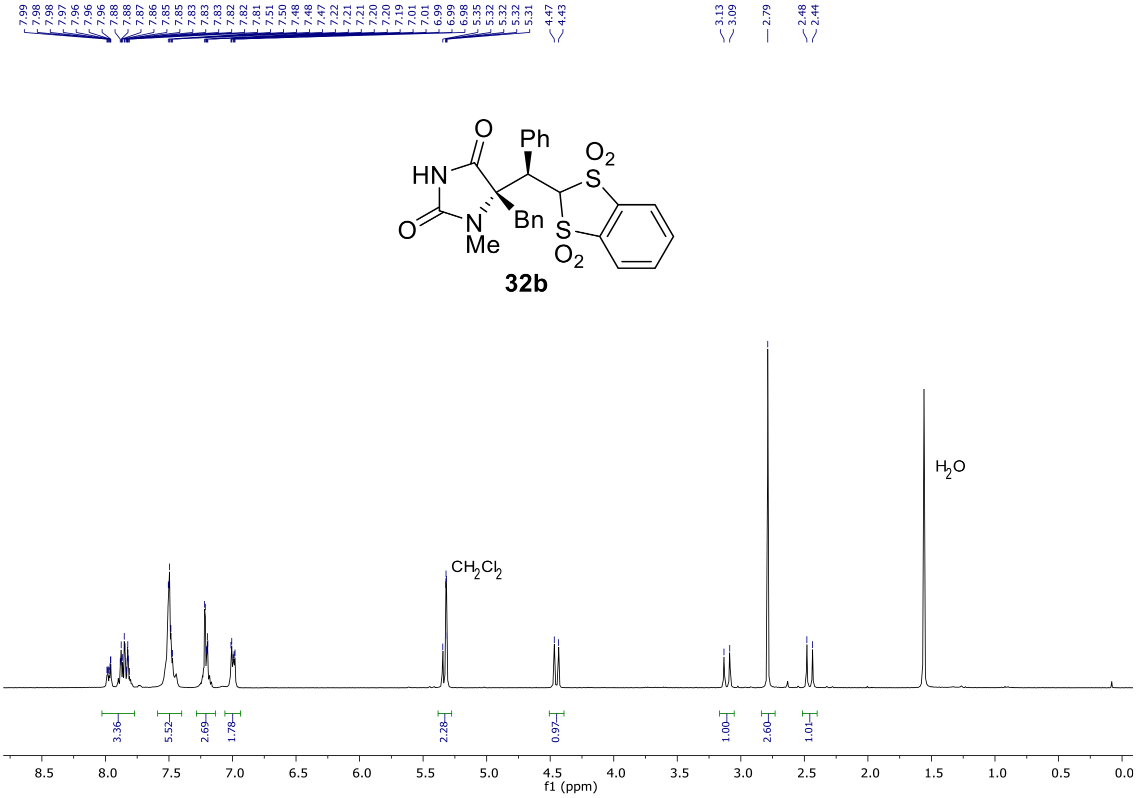

^{13}C{^{1}H} NMR (126 MHz, CD_2Cl_2) of **32b**:

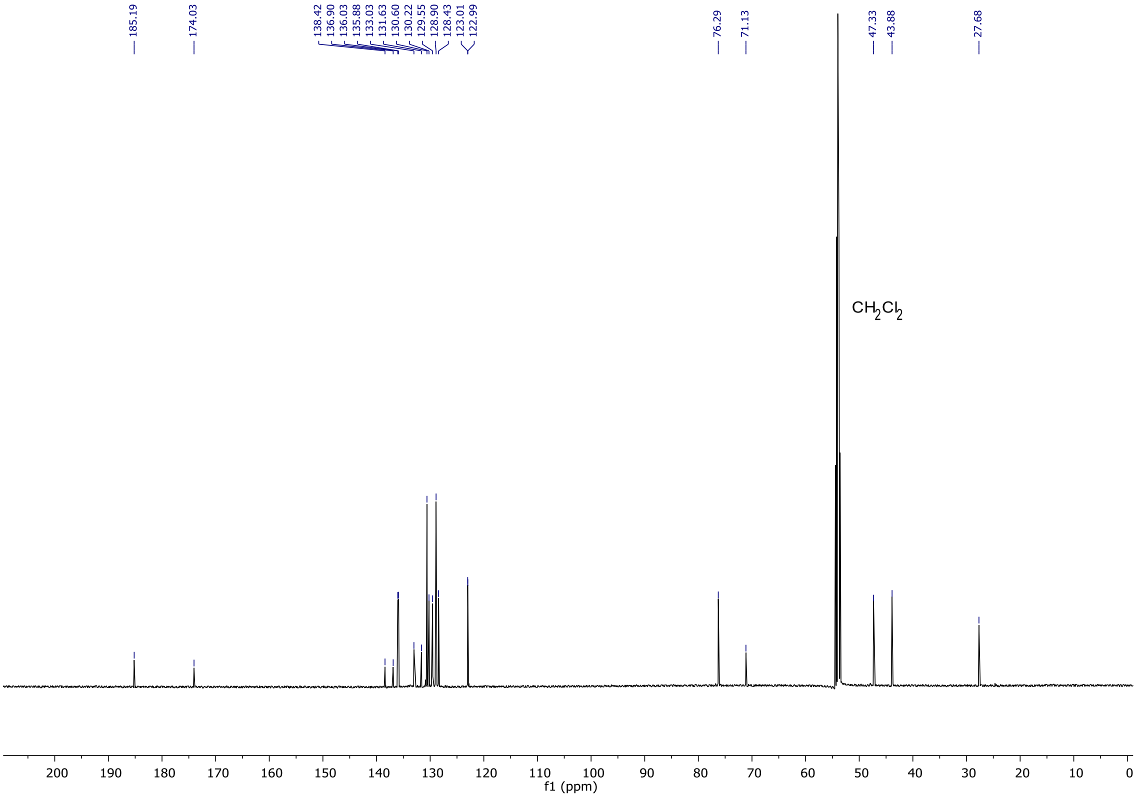

^{1}H NMR (300 MHz, $CDCl_3$) of **32c**:

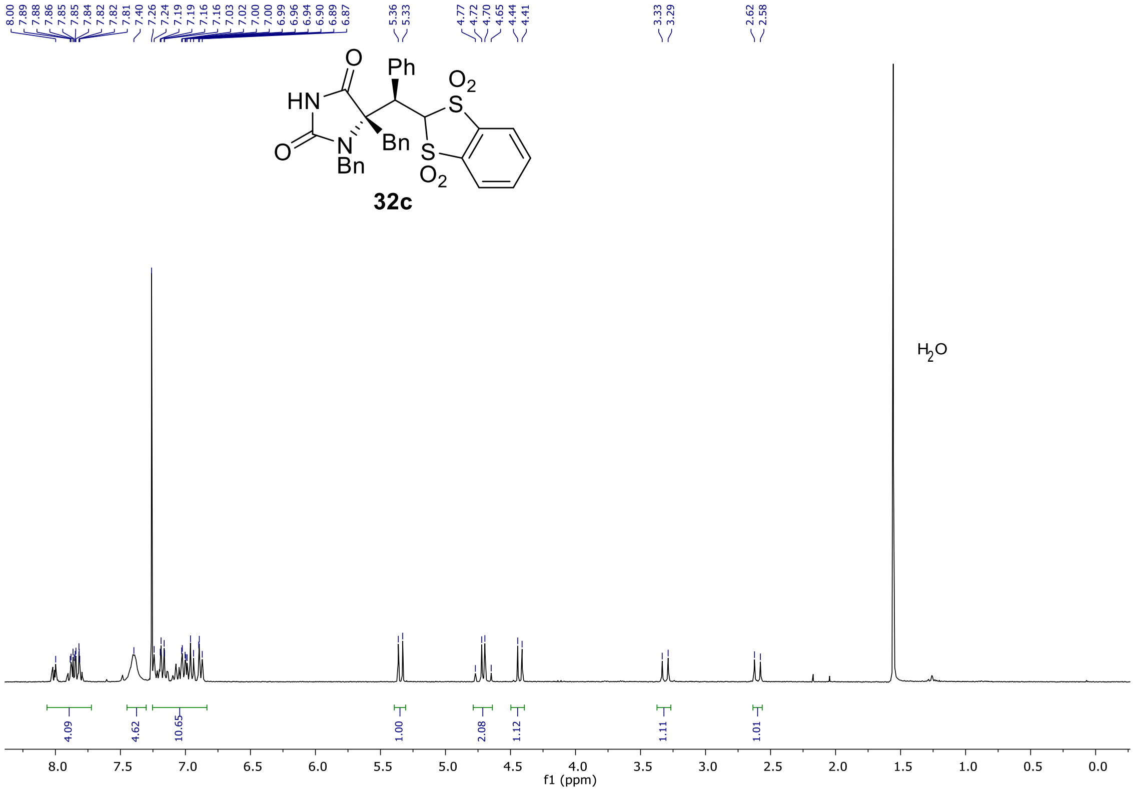

^{13}C{^{1}H} NMR (75 MHz, $CDCl_3$) of **32c**:

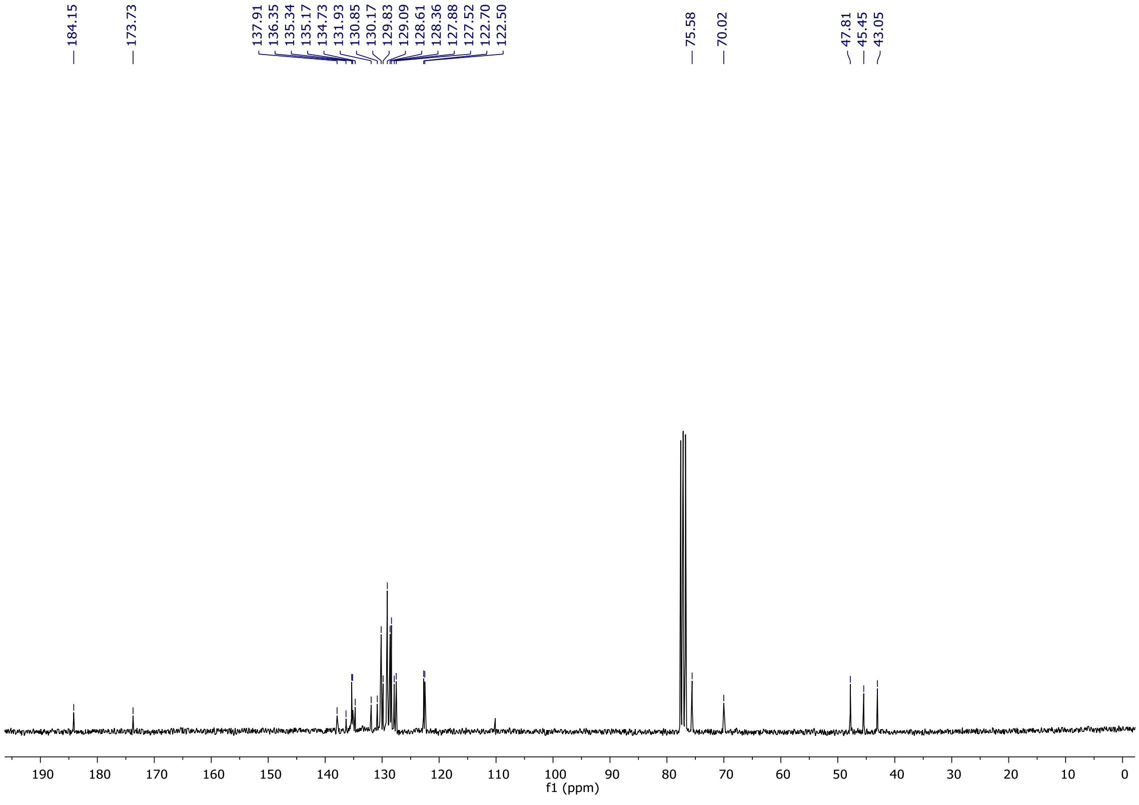

^{1}H NMR (500 MHz, CD_2Cl_2) of **32d**:

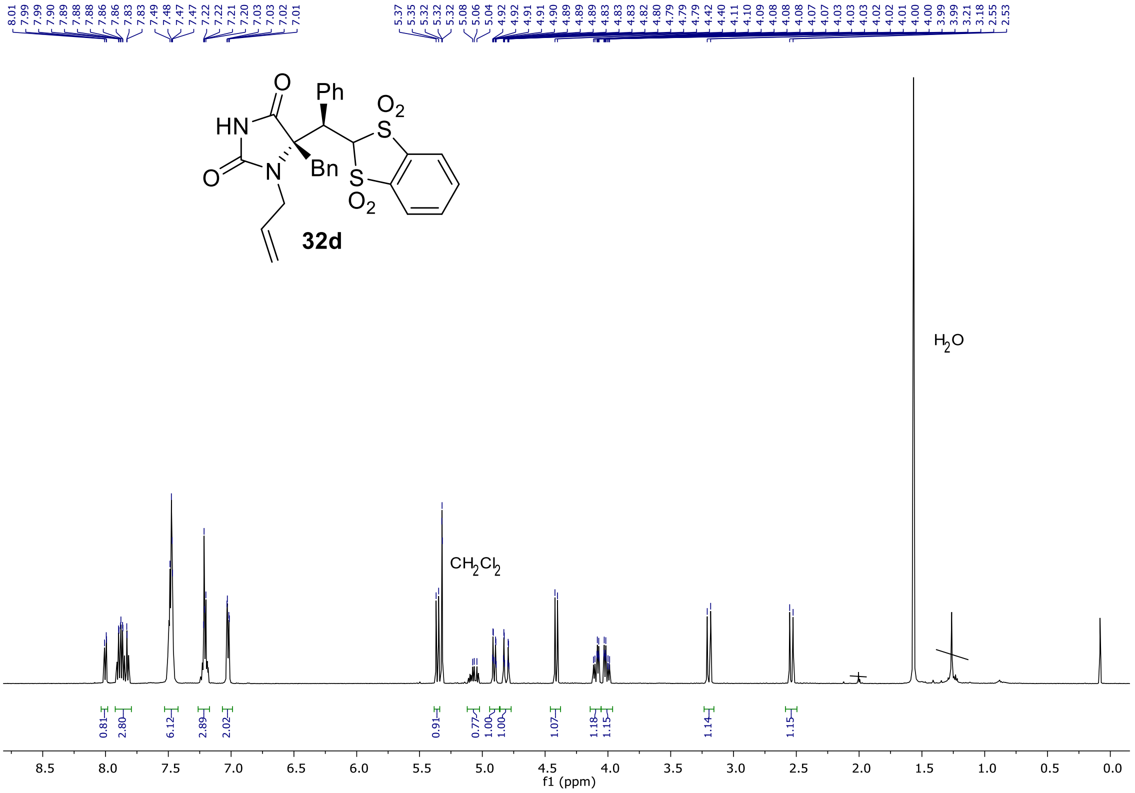

^{13}C{^{1}H} NMR (126 MHz, CD_2Cl_2) of **32d**:

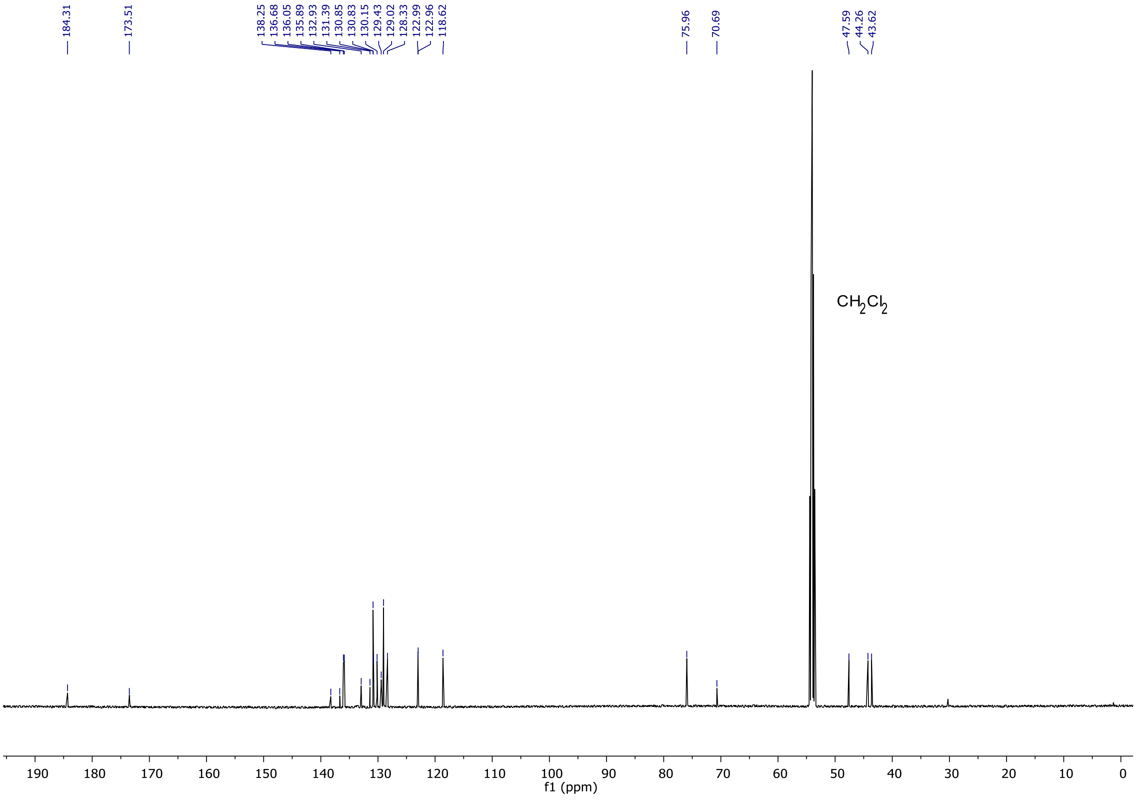

^{1}H NMR (300 MHz, CD_2Cl_2) of **33**:

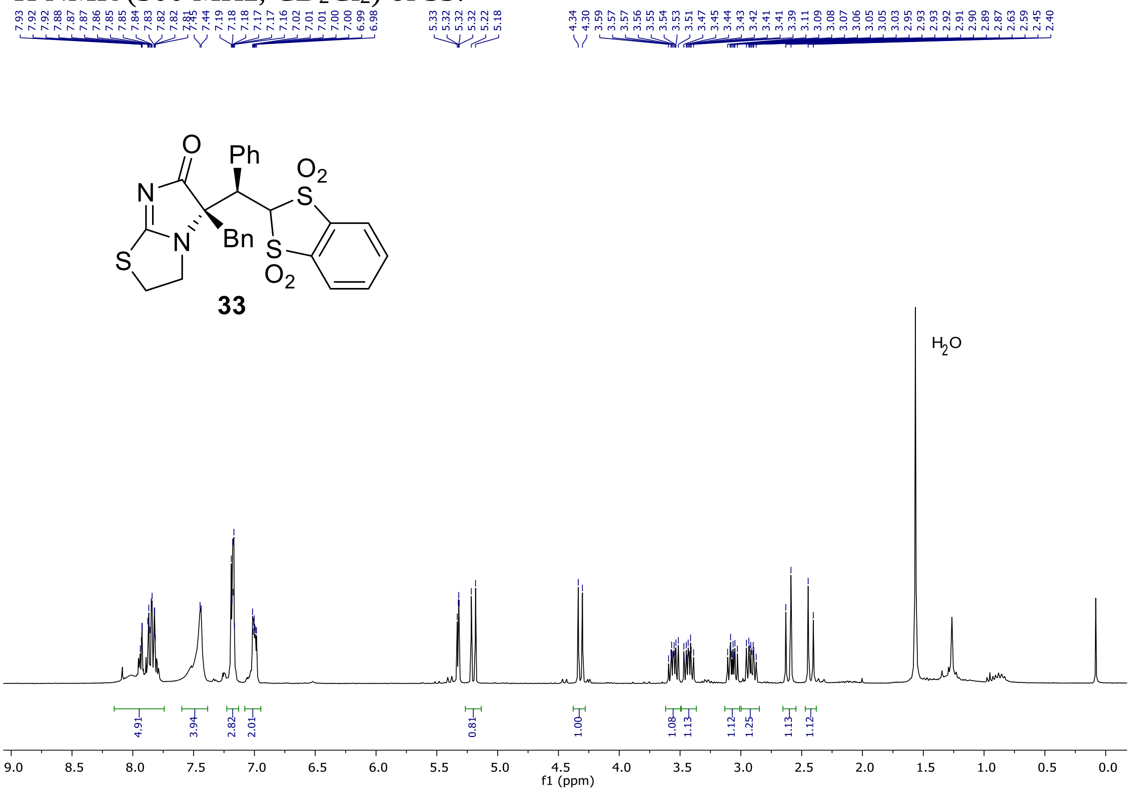

$^{13}C\{^{1}H\}$ NMR (126 MHz, CD_2Cl_2) of **33**:

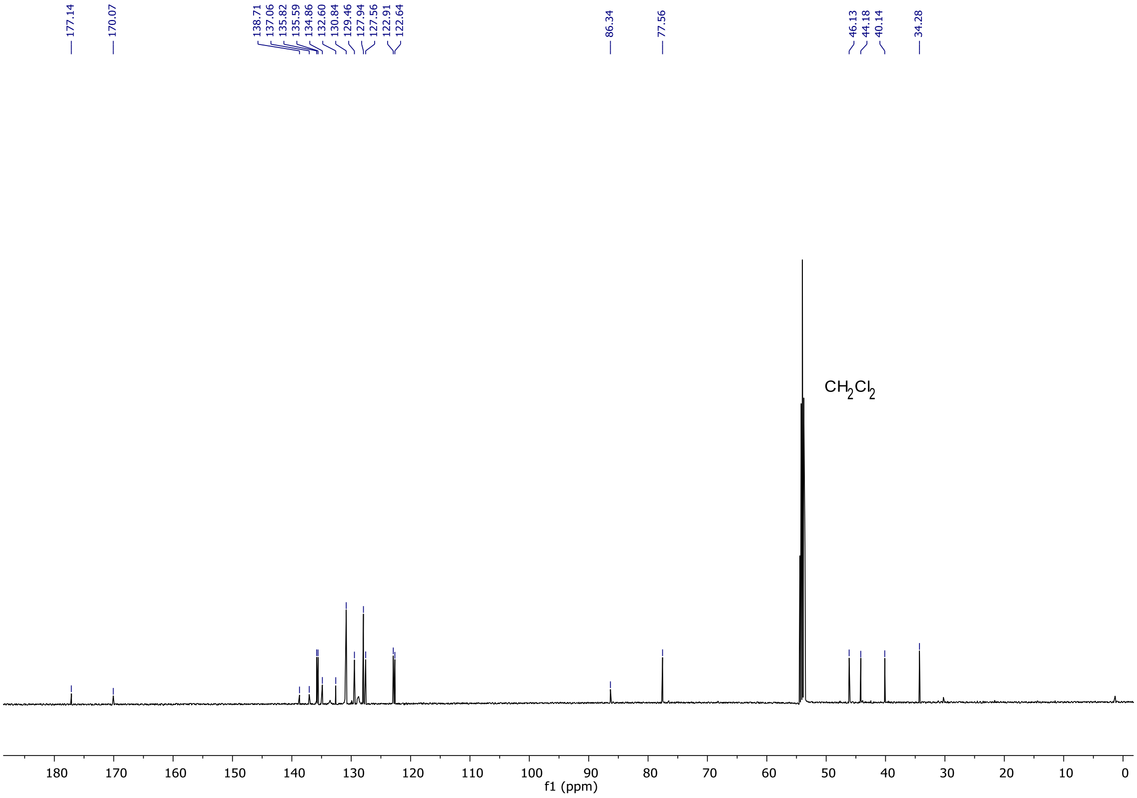

6. HPLC chromatograms.

(*S*)-1-Benzoyl-5-benzyl-2-(benzylthio)-5-(2,2-bis(phenylsulfonyl)ethyl)-1,5-dihydro-4*H*-imidazol-4-one (10a).

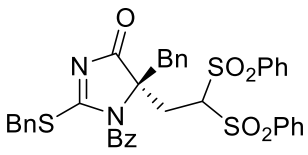

10a

The enantiomeric purity was determined by HPLC analysis (Daicel Chiralpak IC, hexane/isopropanol 30:70, flow rate= 0.5 mL/min, retention times: 43.8 min (major) and 52.0 min (minor).

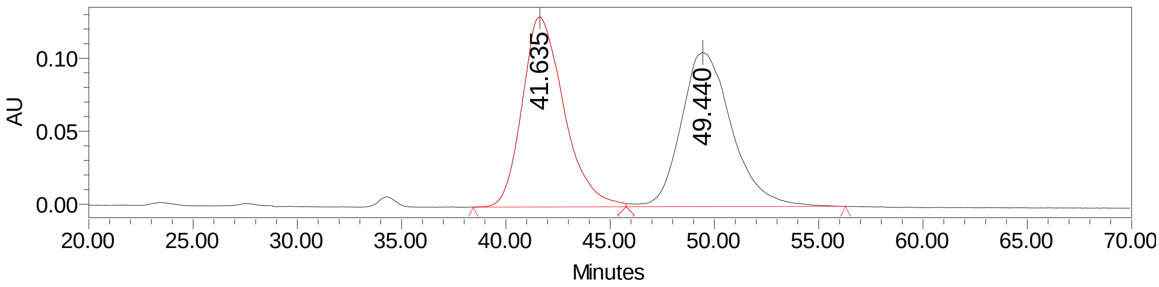

	Retention Time	% Area
1	41.635	50.40
2	49.440	49.60

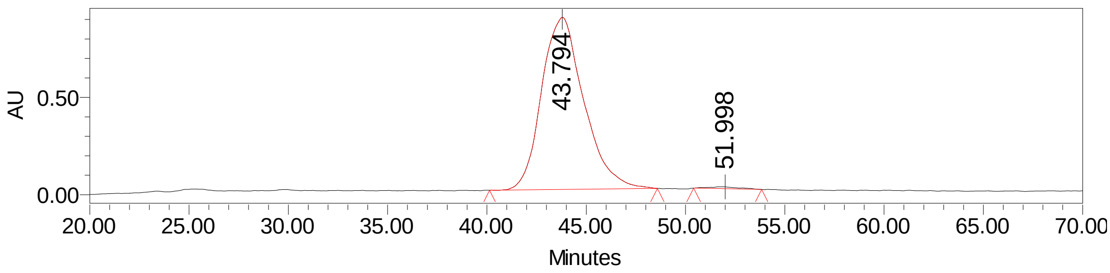

	Retention Time	% Area
1	43.794	99.22
2	51.998	0.78

(*S*)-1-Benzoyl-2-(benzylthio)-5-(2,2-bis(phenylsulfonyl)ethyl)-5-methyl-1,5-dihydro-4*H*-imidazol-4-one (10b).

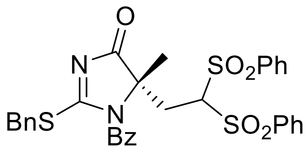

10b

The enantiomeric purity was determined by HPLC analysis (Daicel Chiralpak IA, hexane/isopropanol 30:70, flow rate= 0.5 mL/min, retention times: 32.8 min (major) and 39.7 min (minor).

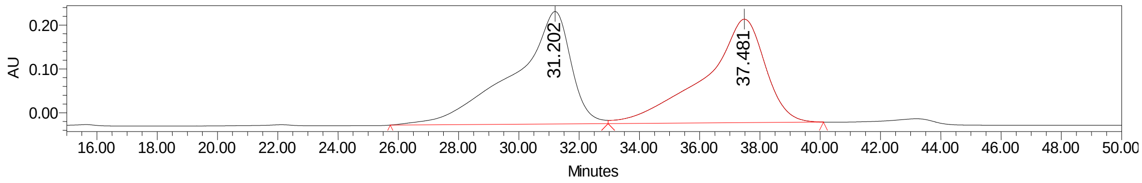

	Retention Time	% Area
1	31.203	50.39
2	37.486	49.61

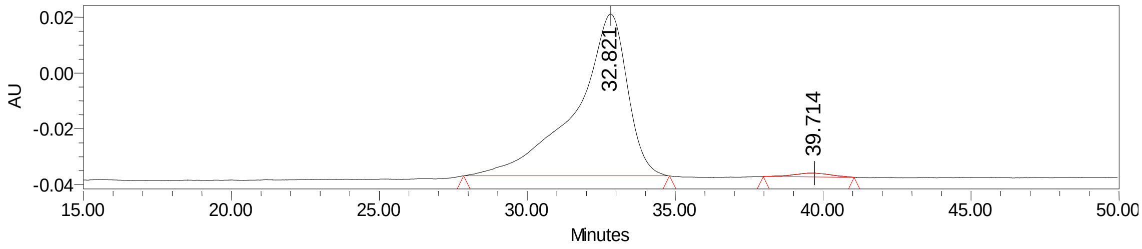

	Retention Time	% Area
1	32.821	98.34
2	39.714	1.66

(*S*)-1-Benzoyl-2-(benzylthio)-5-(2,2-bis(phenylsulfonyl)ethyl)-5-ethyl-1,5-dihydro-4*H*-imidazol-4-one (10c).

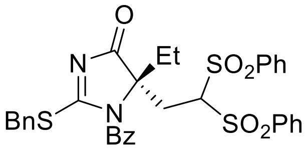

10c

The enantiomeric purity was determined by HPLC analysis (Daicel Chiralpak IF, hexane/isopropanol 30:70, flow rate= 0.5 mL/min, retention times: 44.9 min (major) and 97.4 min (minor).

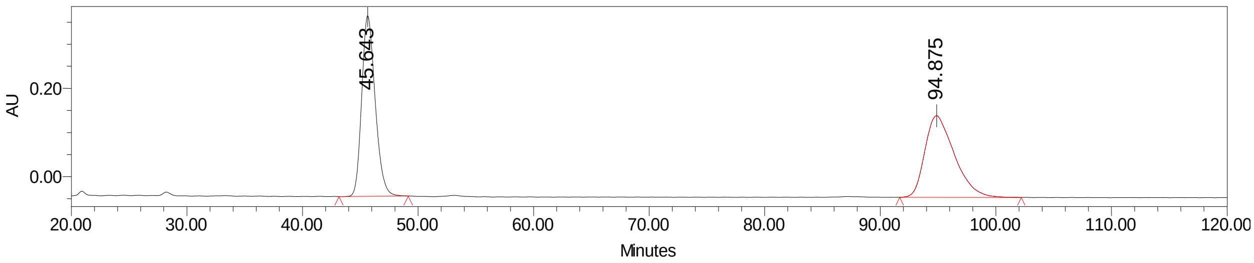

	Retention Time	% Area
1	45.643	50.12
2	94.875	49.88

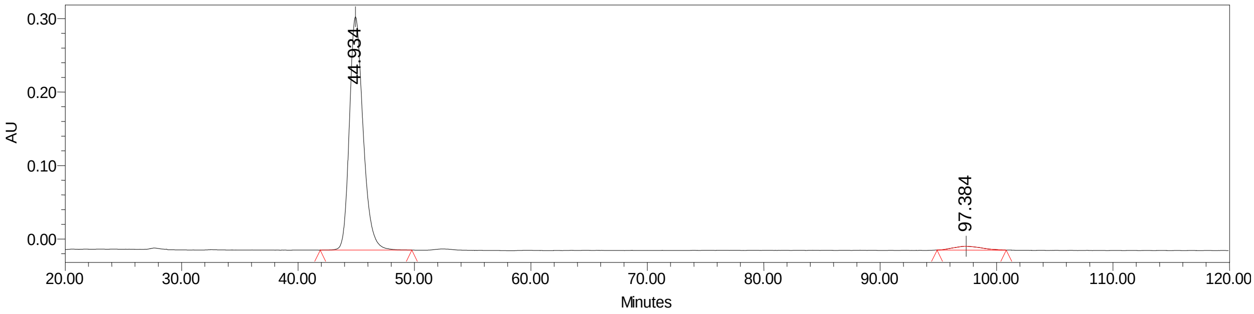

	Retention Time	% Area
1	44.934	96.71
2	97.384	3.29

(*S*)-1-Benzoyl-2-(benzylthio)-5-(2,2-bis(phenylsulfonyl)ethyl)-5-isobutyl-1,5-dihydro-4*H*-imidazol-4-one (10d).

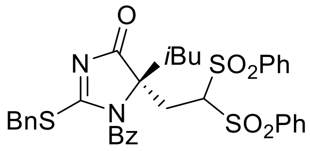

10d

The enantiomeric purity was determined by HPLC analysis (Daicel Chiralpak IF, hexane/isopropanol 30:70, flow rate= 0.5 mL/min, retention times: 44.2 min (major) and 86.0 min (minor).

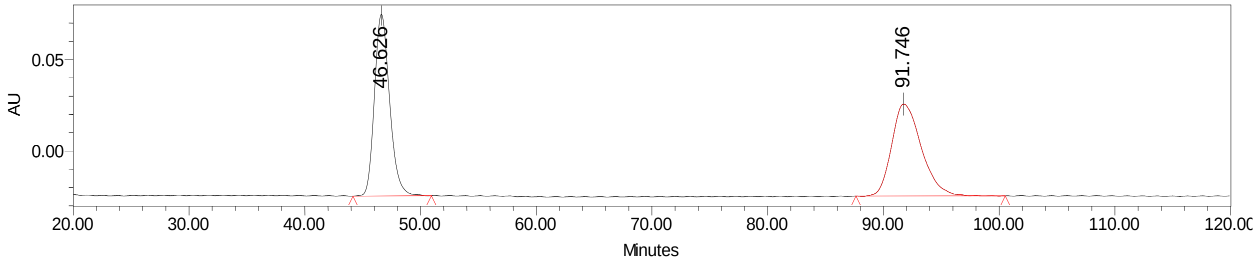

	Retention Time	% Area
1	46.626	50.17
2	91.746	49.83

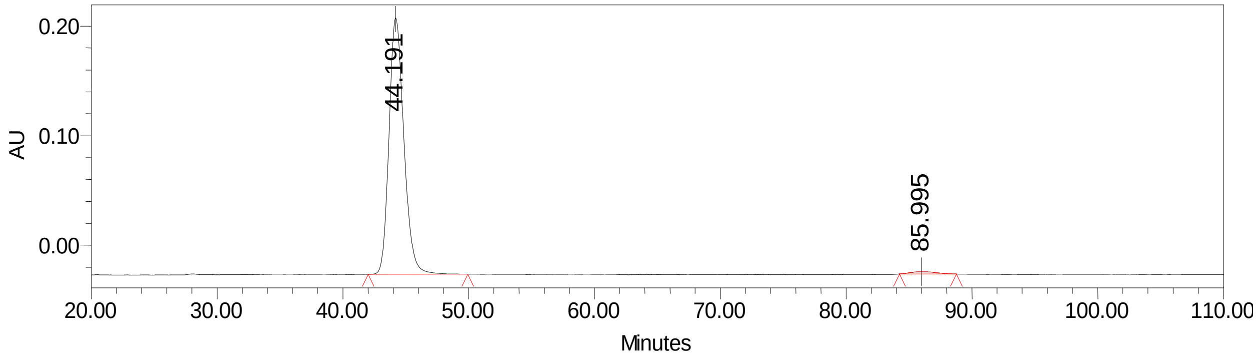

	Retention Time	% Area
1	44.191	98.55
2	85.995	1.45

(*S*)-5-Allyl-1-benzoyl-2-(benzylthio)-5-(2,2-bis(phenylsulfonyl)ethyl)-1,5-dihydro-4*H*-imidazol-4-one (10e).

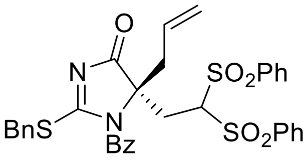

10e

The enantiomeric purity was determined by HPLC analysis (Daicel Chiralpak IC, hexane/isopropanol 30:70, flow rate= 0.5 mL/min, retention times: 34.6 min (major) and 42.5 min (minor).

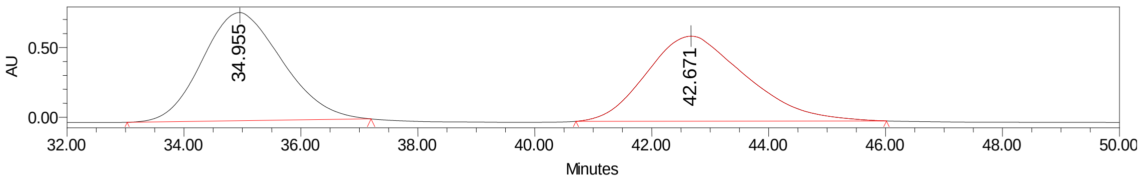

	Retention Time	% Area
1	34.955	50.59
2	42.671	49.41

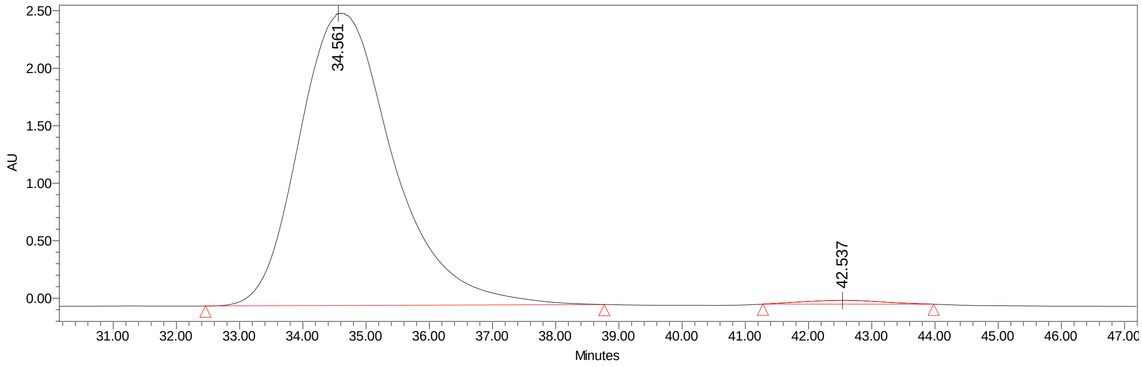

	Retention Time	% Area
1	34.561	98.89
2	42.537	1.11

(*S*)-1-Benzoyl-2-(benzylthio)-5-(2,2-bis(phenylsulfonyl)ethyl)-5-(2-(methylthio)ethyl)-1,5-dihydro-4*H*-imidazol-4-one (10f).

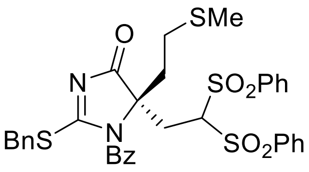

10f

The enantiomeric purity was determined by HPLC analysis (Daicel Chiralpak IF, hexane/isopropanol 30:70, flow rate= 0.5 mL/min, retention times: 58.9 min (major) and 114.1 min (minor).

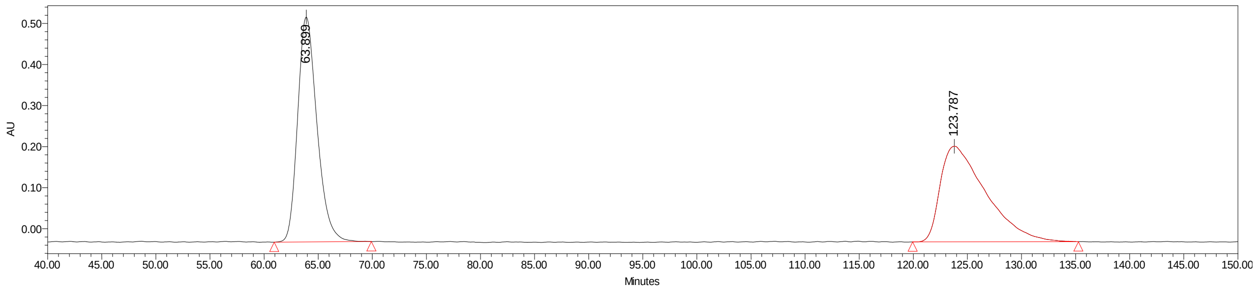

	Retention Time	% Area
1	63.899	50.10
2	123.787	49.90

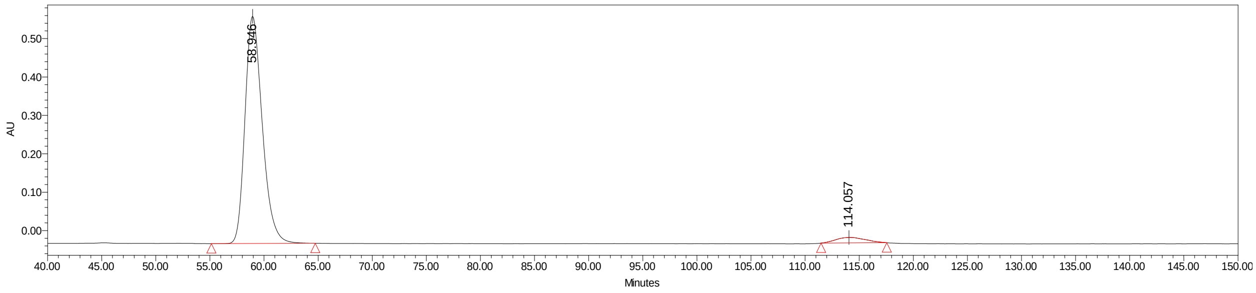

	Retention Time	% Area
1	58.946	95.98
2	114.057	4.02

Methyl (*S*)-2-(1-benzoyl-2-(benzylthio)-5-(2,2-bis(phenylsulfonyl)ethyl)-4-oxo-4,5-dihydro-1*H*-imidazol-5-yl)acetate (10g).

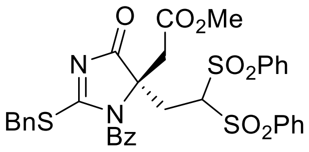

10g

The enantiomeric purity was determined by HPLC analysis (Daicel Chiralpak IC, hexane/isopropanol 30:70, flow rate= 0.5 mL/min, retention times: 47.5 min (minor) and 67.6 min (major).

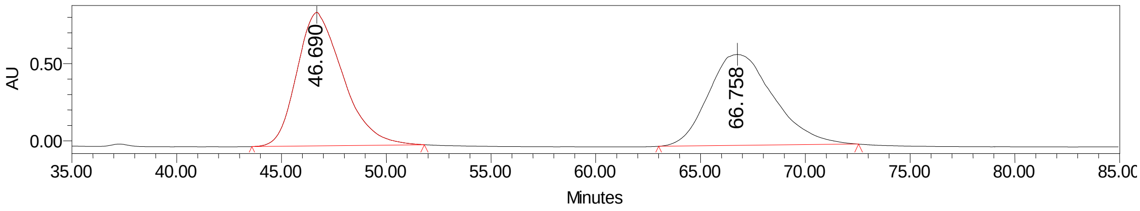

	Retention Time	% Area
1	46.690	50.73
2	66.758	49.27

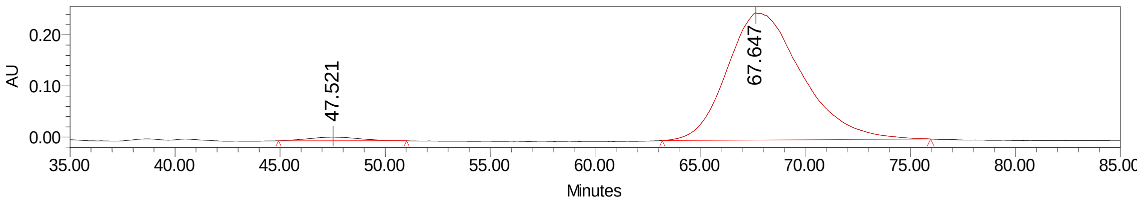

	Retention Time	% Area
1	47.521	1.90
2	67.647	98.10

(*S*)-5-Benzyl-2-(benzylthio)-5-(2,2-bis(phenylsulfonyl)ethyl)-3-phenyl-3,5-dihydro-4*H*-imidazol-4-one (12).

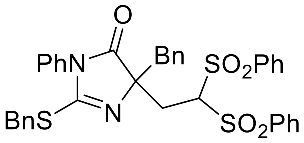

12

The enantiomeric purity was determined by HPLC analysis (Daicel Chiralpak IA, hexane/isopropanol 50:50, flow rate= 0.5 mL/min, retention times: 17.7 min (major) and 21.8 min (minor).

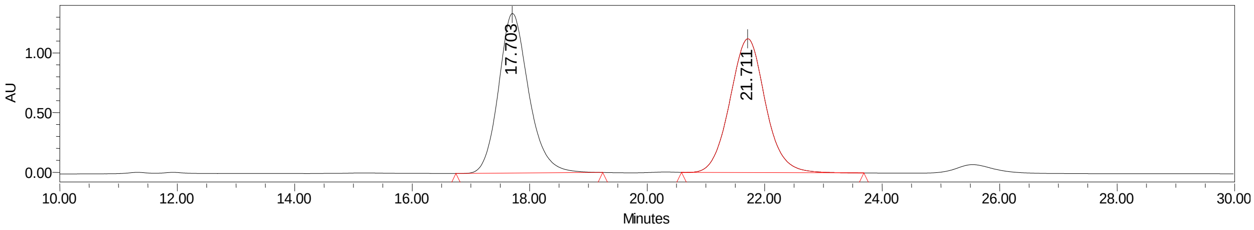

	Retention Time	% Area
1	17.703	50.28
2	21.711	49.72

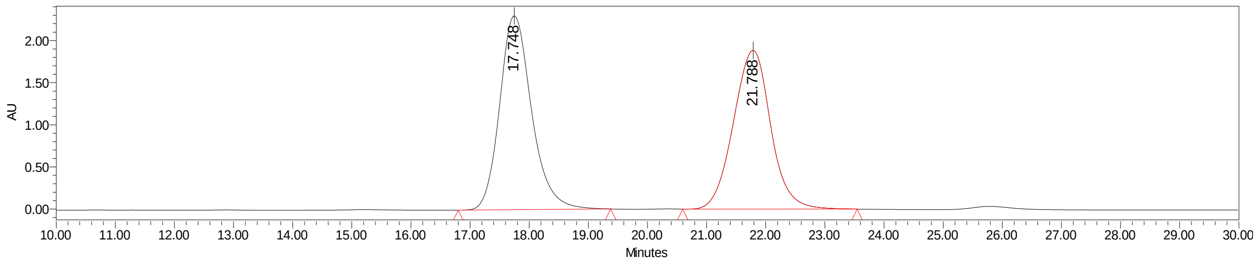

	Retention Time	% Area
1	17.748	51.11
2	21.788	48.89

(*S*)-4-Benzyl-4-(2,2-bis(phenylsulfonyl)ethyl)-2-phenyloxazol-5(4*H*)-one (13).

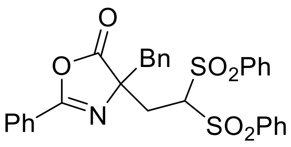

13

The enantiomeric purity was determined by HPLC analysis (Daicel Chiralpak IA, hexane/isopropanol 60:40, flow rate= 0.5 mL/min, retention times: 22.0 min (major) and 26.4 min (minor).

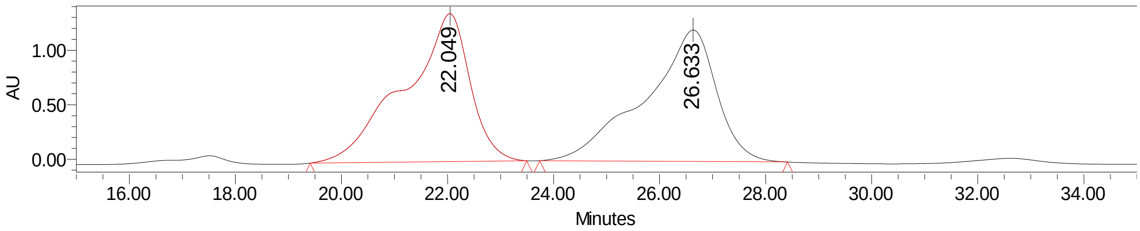

	Retention Time	% Area
1	22.049	50.72
2	26.633	49.28

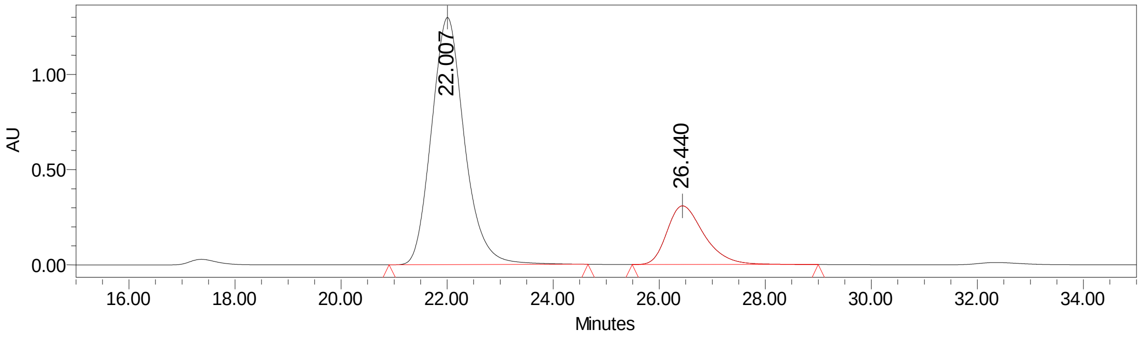

	Retention Time	% Area
1	22.007	78.86
2	26.440	21.14

(*S*)-1-Benzoyl-5-benzyl-2-(benzylthio)-5-((*R*)-phenyl(1,1,3,3-tetraoxido-2*H*-benzo[*d*][1,3]dithiol-2-yl)methyl)-1,5-dihydro-4*H*-imidazol-4-one (15aa).

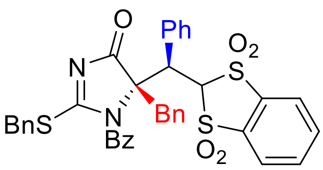

15aa

The enantiomeric purity was determined by HPLC analysis (Daicel Chiralpak IA, hexane/isopropanol 30:70, flow rate= 0.5 mL/min, retention times: 37.0 min (minor) and 55.5 min (major).

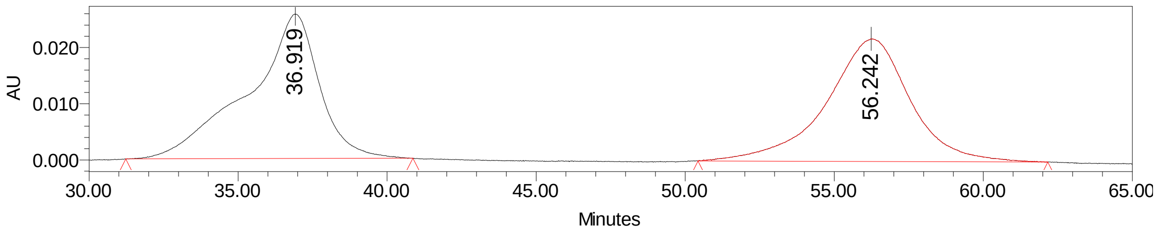

	Retention Time	% Area
1	36.919	49.98
2	56.242	50.05

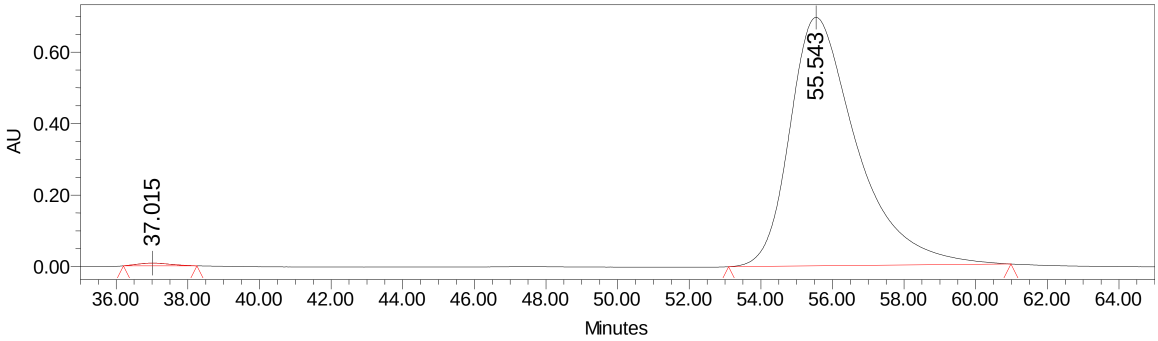

	Retention Time	% Area
1	37.015	0.54
2	55.543	99.46

(*S*)-1-Benzoyl-2-(benzylthio)-5-ethyl-5-((*R*)-phenyl(1,1,3,3-tetraoxido-2*H*-benzo[*d*][1,3]dithiol-2-yl)methyl)-1,5-dihydro-4*H*-imidazol-4-one (15ca).

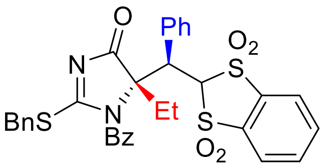

15ca

The enantiomeric purity was determined by HPLC analysis (Daicel Chiralpak IC, hexane/isopropanol 30:70, flow rate= 0.5 mL/min, retention times: 38.1 min (minor) and 55.8 min (major).

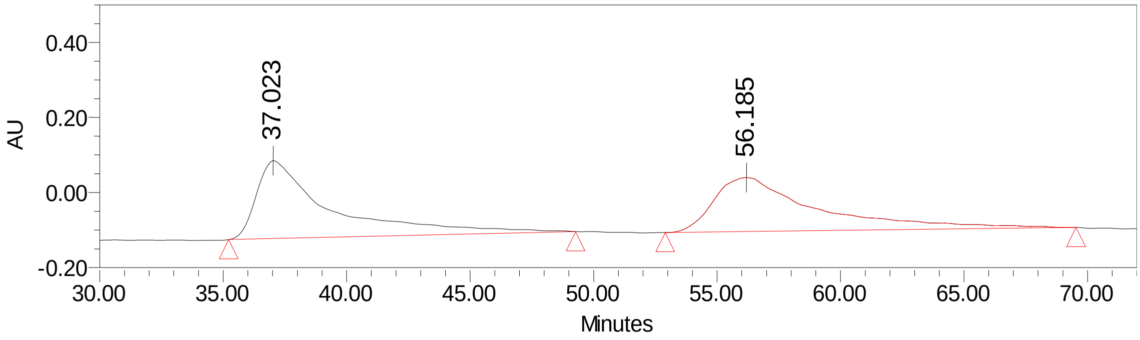

	Retention Time	% Area
1	37.023	50.99
2	56.185	49.01

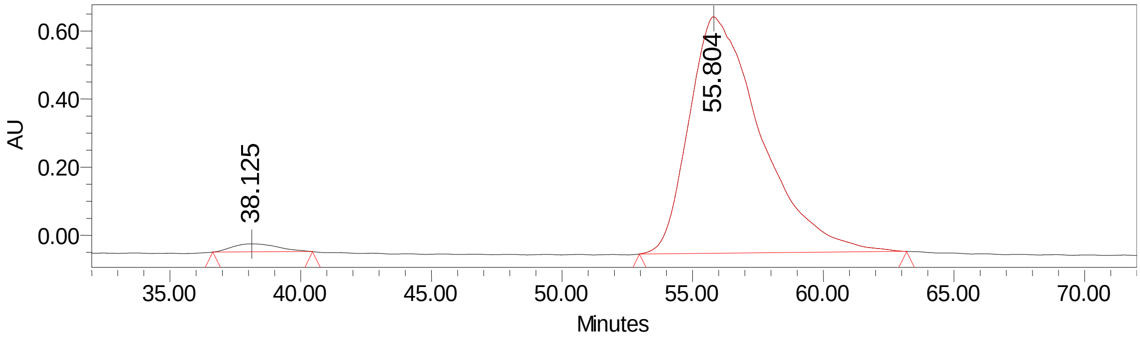

	Retention Time	% Area
1	38.125	2.12
2	55.804	97.88

(*S*)-1-Benzoyl-2-(benzylthio)-5-isobutyl-5-((*R*)-phenyl(1,1,3,3-tetraoxido-2*H*-benzo[*d*][1,3]dithiol-2-yl)methyl)-1,5-dihydro-4*H*-imidazol-4-one (15da).

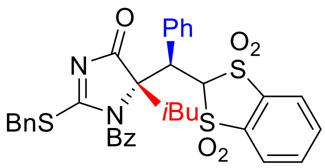

15da

The enantiomeric purity was determined by HPLC analysis (Daicel Chiralpak IC, hexane/isopropanol 30:70, flow rate= 0.5 mL/min, retention times: 29.4 min (minor) and 75.1 min (major).

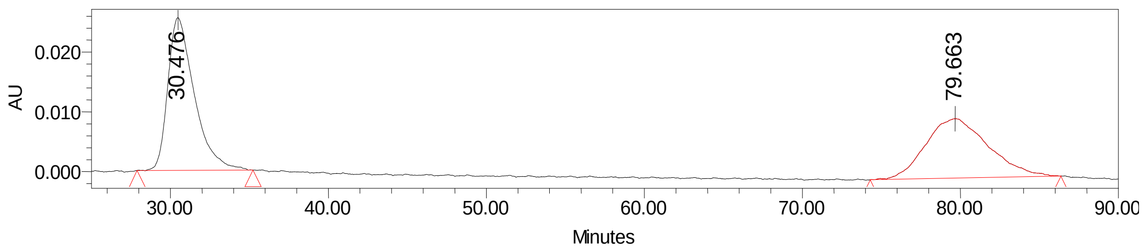

	Retention Time	% Area
1	30.476	52.06
2	79.663	47.94

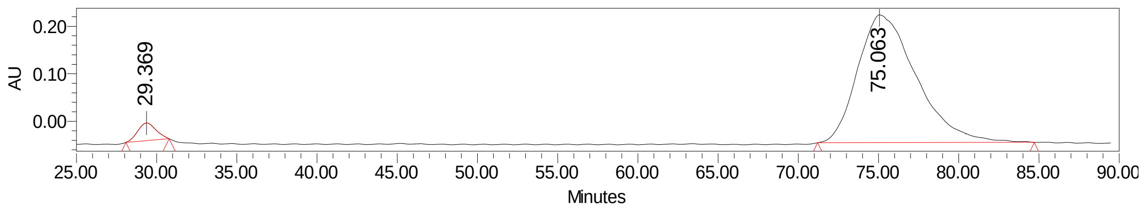

	Retention Time	% Area
1	29.369	4.14
2	75.063	95.86

(*S*)-5-Allyl-1-benzoyl-2-(benzylthio)-5-((*R*)-phenyl(1,1,3,3-tetraoxido-2*H*-benzo[*d*][1,3]dithiol-2-yl)methyl)-1,5-dihydro-4*H*-imidazol-4-one (15ea).

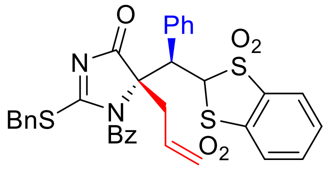

15ea

The enantiomeric purity was determined by HPLC analysis (Daicel Chiralpak IC, hexane/isopropanol 30:70, flow rate= 0.5 mL/min, retention times: 49.0 min (minor) and 57.9 min (major).

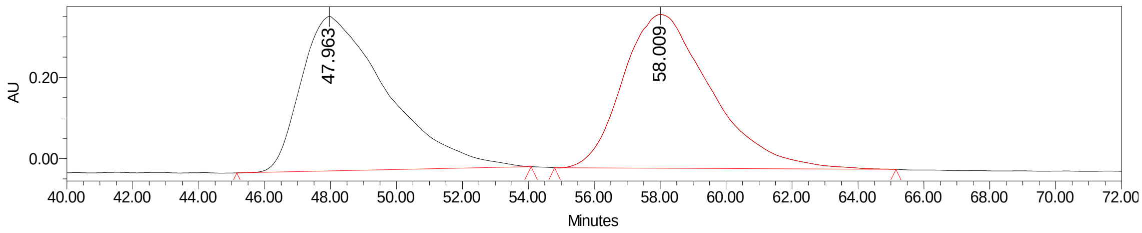

	Retention Time	% Area
1	47.963	49.42
2	58.009	50.58

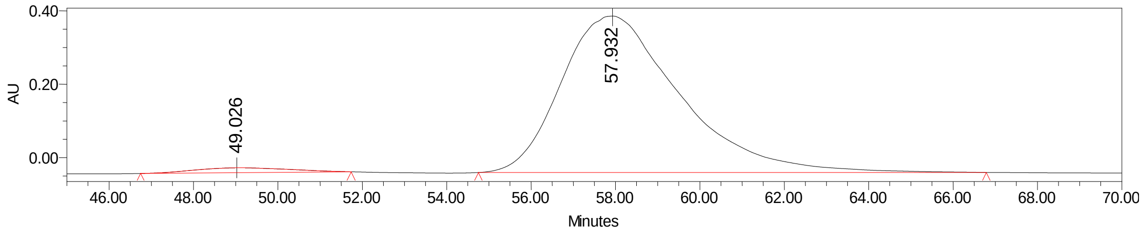

	Retention Time	% Area
1	49.026	2.42
2	57.932	97.58

(*S*)-1-Benzoyl-2-(benzylthio)-5-(2-(methylthio)ethyl)-5-((*R*)-phenyl(1,1,3,3-tetraoxido-2*H*-benzo[*d*][1,3]dithiol-2-yl)methyl)-1,5-dihydro-4*H*-imidazol-4-one (15fa).

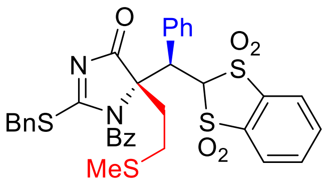

15fa

The enantiomeric purity was determined by HPLC analysis (Daicel Chiralpak IC, hexane/isopropanol 30:70, flow rate= 0.5 mL/min, retention times: 40.6 min (minor) and 74.2 min (major).

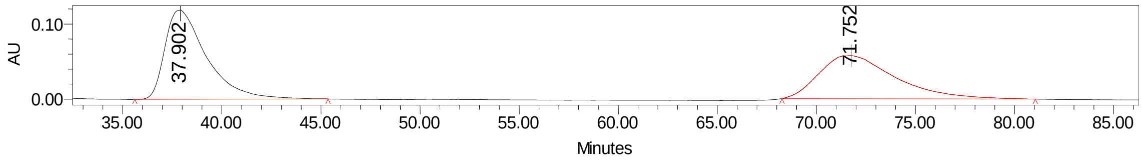

	Retention Time	% Area
1	37.902	52.96
2	71.752	47.04

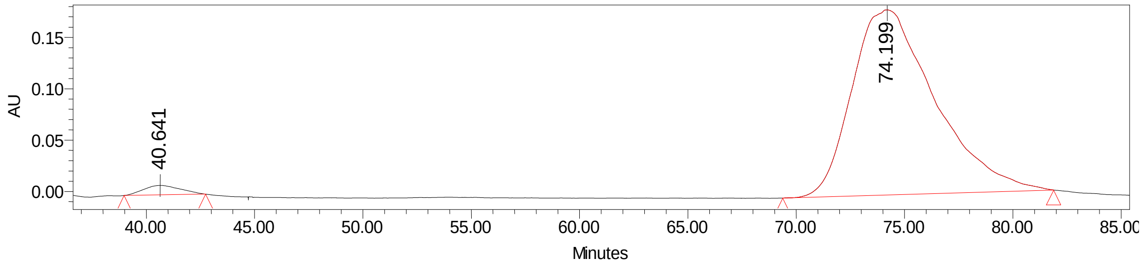

	Retention Time	% Area
1	40.641	2.23
2	74.199	97.77

Methyl 2-((*S*)-1-benzoyl-2-(benzylthio)-4-oxo-5-((*R*)-phenyl(1,1,3,3-tetraoxido-2*H*-benzo[*d*][1,3]dithiol-2-yl)methyl)-4,5-dihydro-1*H*-imidazol-5-yl)acetate (15ga).

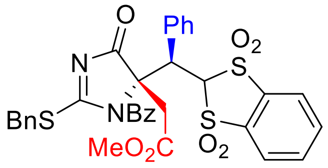

15ga

The enantiomeric purity was determined by HPLC analysis (Daicel Chiralpak IC, hexane/isopropanol 30:70, flow rate= 0.5 mL/min, retention times: 63.9 min (minor) and 80.6 min (major).

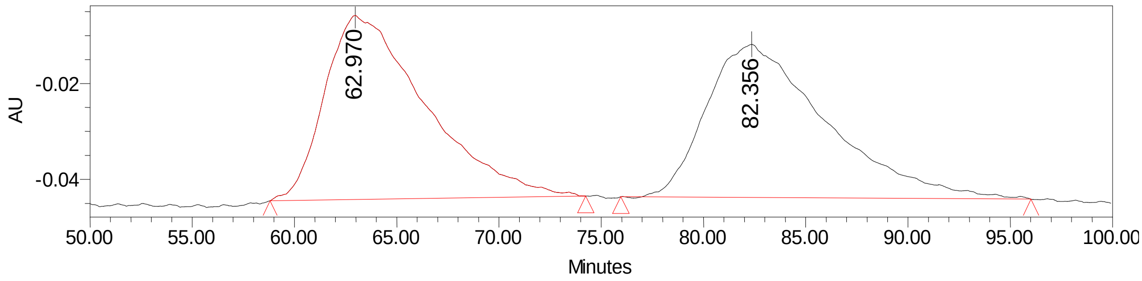

	Retention Time	% Area
1	62.970	50.15
2	82.356	49.85

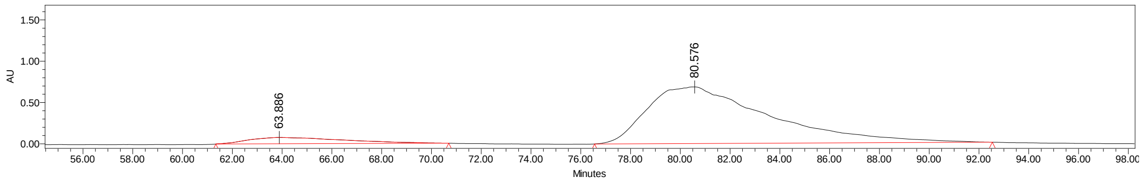

	Retention Time	% Area
1	63.886	8.05
2	80.576	91.95

Methyl 2-(((*S*)-1-benzoyl-2-(benzylthio)-4-oxo-5-((*R*)-phenyl(1,1,3,3-tetraoxido-2*H*-benzo[*d*][1,3]dithiol-2-yl)methyl)-4,5-dihydro-1*H*-imidazol-5-yl)methyl)acrylate (15ha).

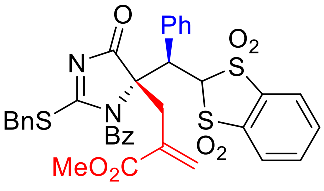

15ha

The enantiomeric purity was determined by HPLC analysis (Daicel Chiralpak IA, hexane/ethanol 30:70, flow rate= 0.5 mL/min, retention times: 56.8 min (minor) and 134.3 min (major).

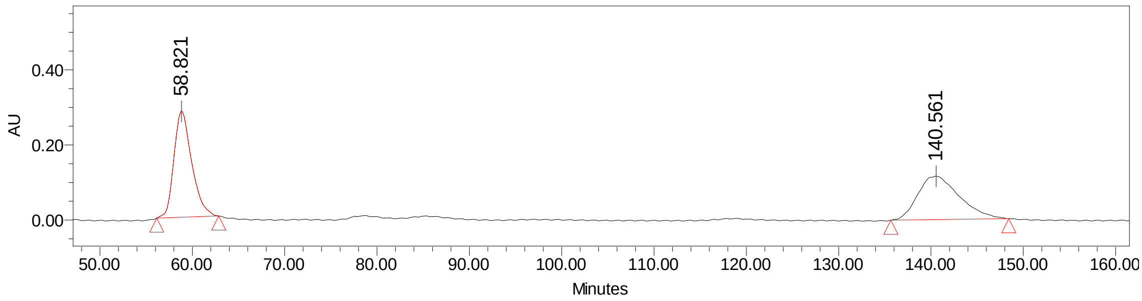

	Retention Time	% Area
1	58.821	51.05
2	140.561	48.95

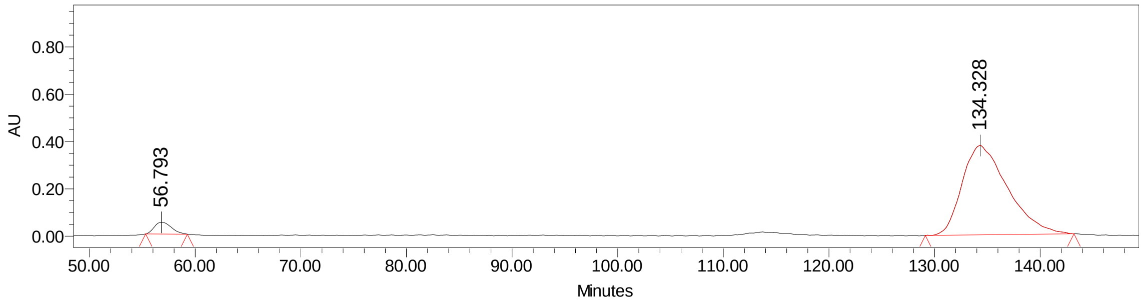

	Retention Time	% Area
1	56.793	4.72
2	134.328	95.28

(*S*)-1-Benzoyl-5-benzyl-2-(benzylthio)-5-((*R*)-(4-methoxyphenyl)(1,1,3,3-tetraoxido-2*H*-benzo[*d*][1,3]dithiol-2-yl)methyl)-1,5-dihydro-4*H*-imidazol-4-one (15ab).

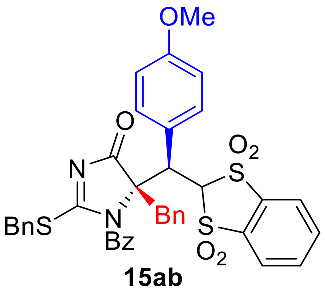

15ab

The enantiomeric purity was determined by HPLC analysis (Daicel Chiralpak IA, hexane/isopropanol 50:50, flow rate= 0.5 mL/min, retention times: 66.4 min (minor) and 121.7 min (major).

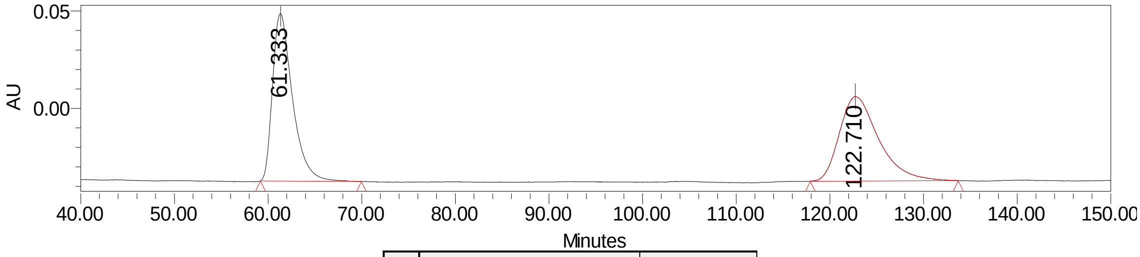

	Retention Time	% Area
1	61.333	50.17
2	122.710	49.83

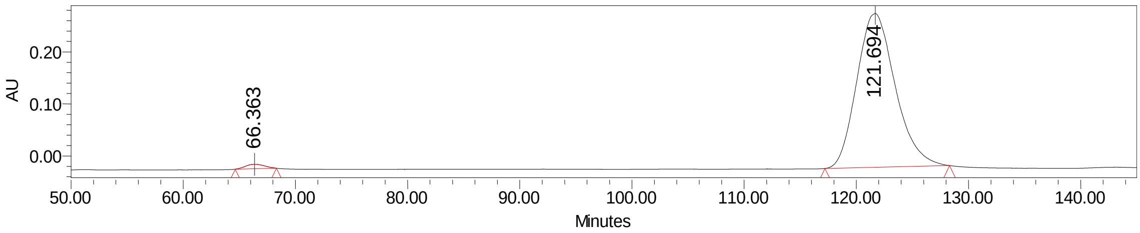

	Retention Time	% Area
1	66.362	1.33
2	121.688	98.67

(*S*)-1-Benzoyl-5-benzyl-2-(benzylthio)-5-((*R*)-(4-chlorophenyl)(1,1,3,3-tetraoxido-2*H*-benzo[*d*][1,3]dithiol-2-yl)methyl)-1,5-dihydro-4*H*-imidazol-4-one (15ac).

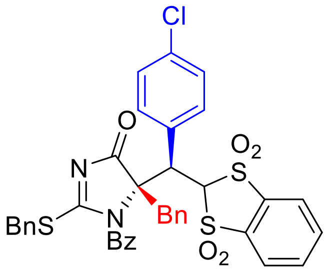

15ac

The enantiomeric purity was determined by HPLC analysis (Daicel Chiralpak IC, hexane/isopropanol 30:70, flow rate= 0.5 mL/min, retention times: 33.4 min (minor) and 37.6 min (major).

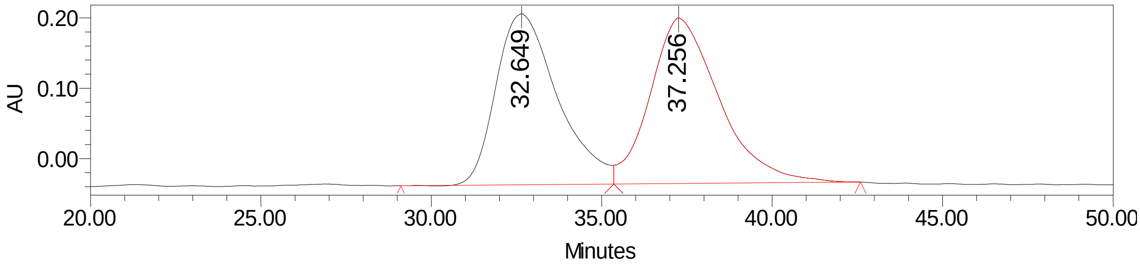

	Retention Time	% Area
1	32.649	48.06
2	37.256	51.94

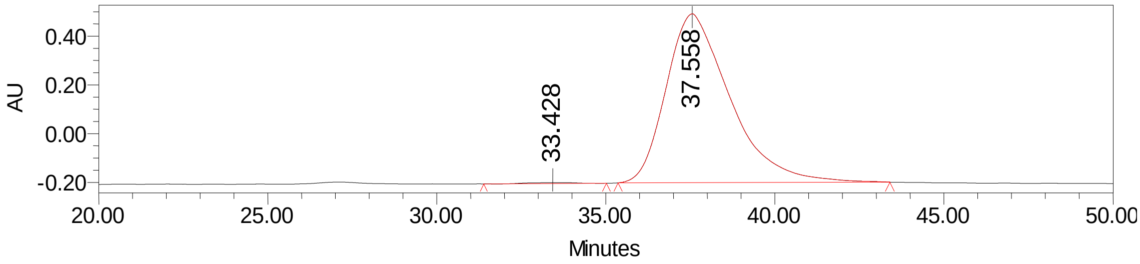

	Retention Time	% Area
1	33.428	0.44
2	37.558	99.56

(*S*)-1-Benzoyl-2-(benzylthio)-5-((4-chlorophenyl)(1,1,3,3-tetraoxido-2*H*-benzo[*d*][1,3]dithiol-2-yl)methyl)-5-methyl-1,5-dihydro-4*H*-imidazol-4-one (15bc).

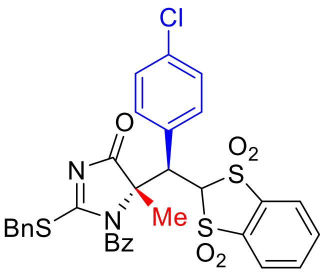

15bc

The enantiomeric purity was determined by HPLC analysis (Daicel Chiralpak IF, hexane/ethanol 30:70, flow rate= 0.5 mL/min, retention times: 32.6 min (minor) and 37.2 min (major).

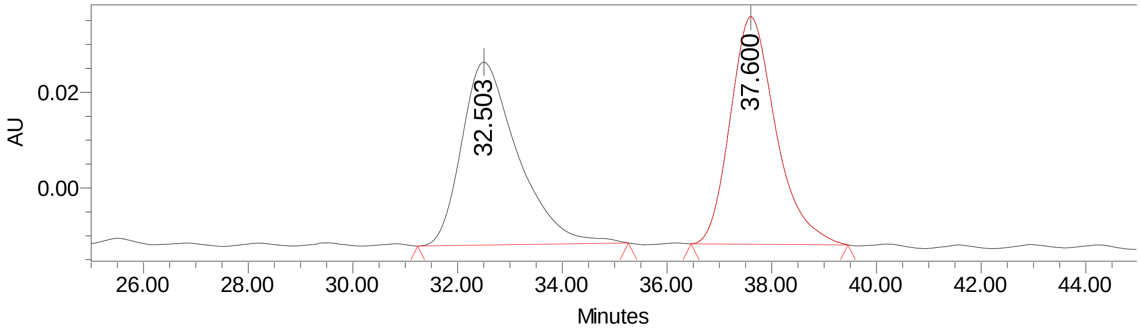

	Retention Time	% Area
1	32.503	49.71
2	37.600	50.29

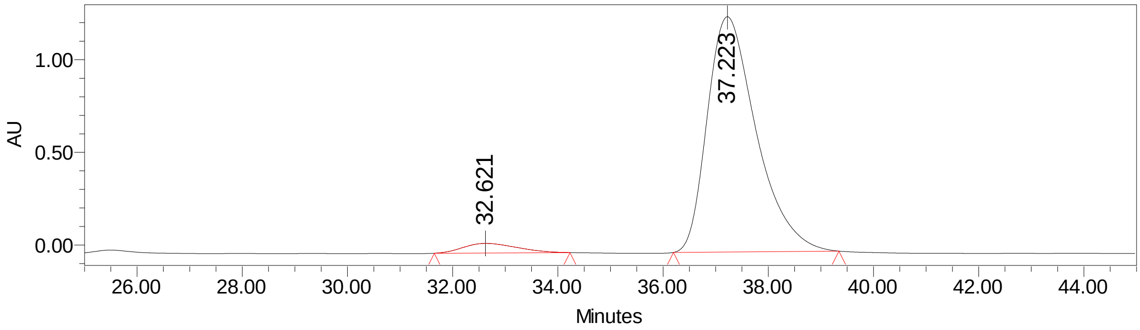

	Retention Time	% Area
1	32.621	4.27
2	37.223	95.73

(*S*)-5-Allyl-1-benzoyl-2-(benzylthio)-5-((*R*)-(4-chlorophenyl)(1,1,3,3-tetraoxido-2*H*-benzo[*d*][1,3]dithiol-2-yl)methyl)-1,5-dihydro-4*H*-imidazol-4-one (15ec).

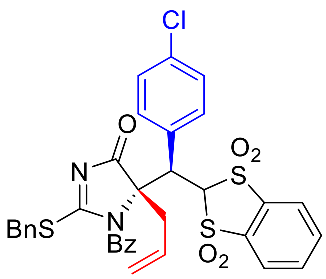

15ec

The enantiomeric purity was determined by HPLC analysis (Daicel Chiralpak IA, hexane/isopropanol 50:50, flow rate= 0.5 mL/min, retention times: 45.4 min (minor) and 84.5 min (major).

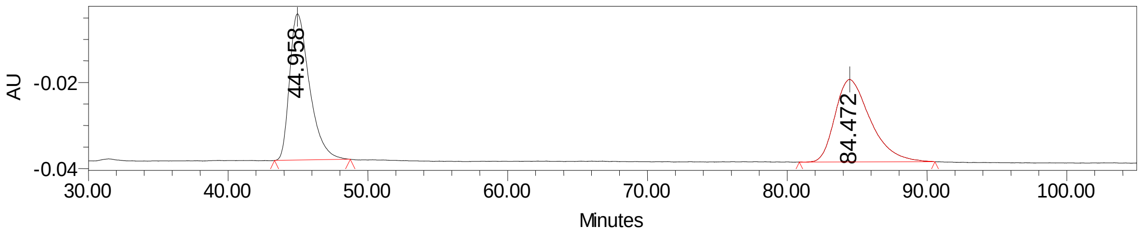

	Retention Time	% Area
1	44.958	49.94
2	84.472	50.06

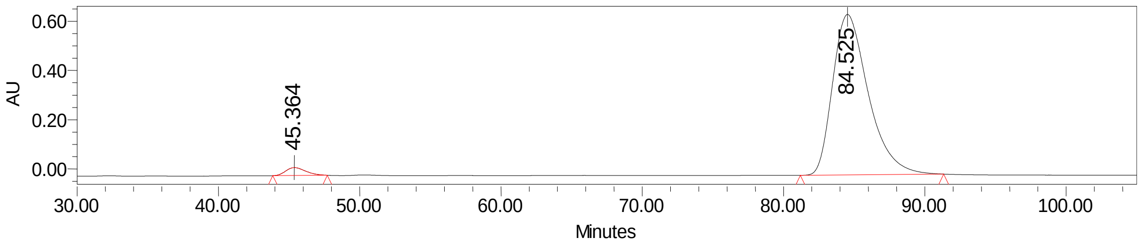

	Retention Time	% Area
1	45.364	2.72
2	84.525	97.28

(*S*)-1-Benzoyl-5-benzyl-2-(benzylthio)-5-((*S*)-furan-2-yl(1,1,3,3-tetraoxido-2*H*-benzo[*d*][1,3]dithiol-2-yl)methyl)-1,5-dihydro-4*H*-imidazol-4-one (15ad).

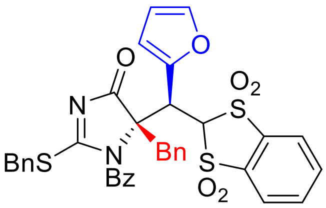

15ad

The enantiomeric purity was determined by HPLC analysis (Daicel Chiralpak IC, hexane/isopropanol 30:70, flow rate= 0.5 mL/min, retention times: 66.9 min (minor) and 83.4 min (major).

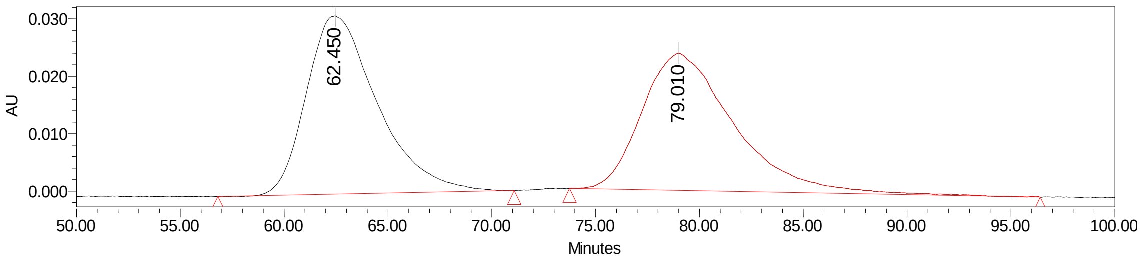

	Retention Time	% Area
1	62.450	50.14
2	79.010	49.86

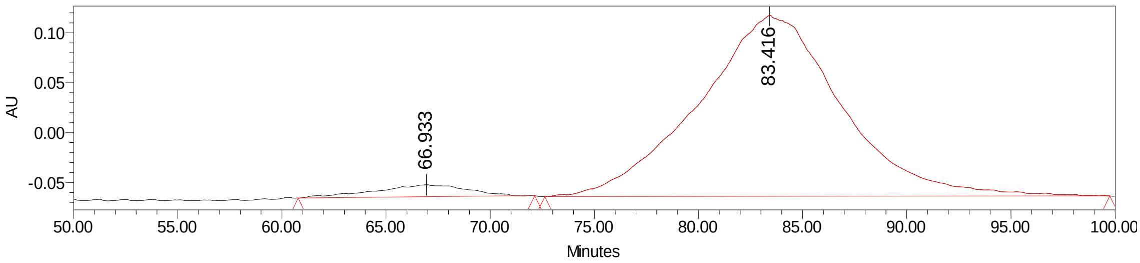

	Retention Time	% Area
1	66.933	4.12
2	83.416	95.88

(*S*)-1-Benzoyl-5-benzyl-2-(benzylthio)-5-((*S*)-(1,1,3,3-tetraoxido-2*H*-benzo[*d*][1,3]dithiol-2-yl)(thiophen-2-yl)methyl)-1,5-dihydro-4*H*-imidazol-4-one (15ae).

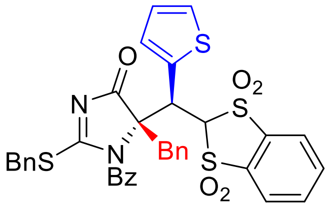

15ae

The enantiomeric purity was determined by HPLC analysis (Daicel Chiralpak IC, hexane/isopropanol 30:70, flow rate= 0.5 mL/min, retention times: 113.0 min (minor) and 136.3 min (major).

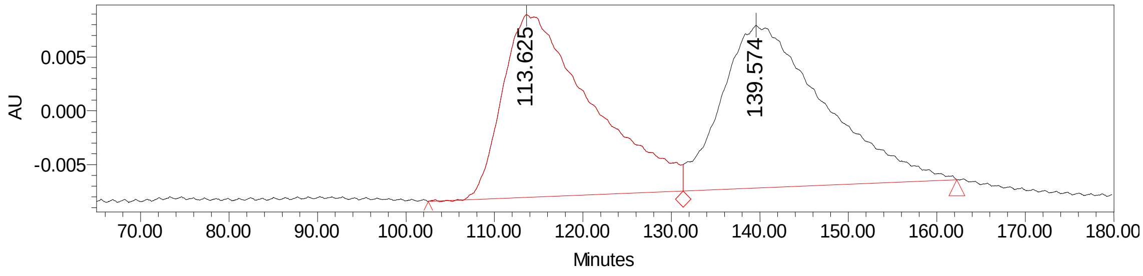

	Retention Time	% Area
1	113.625	49.41
2	139.574	50.59

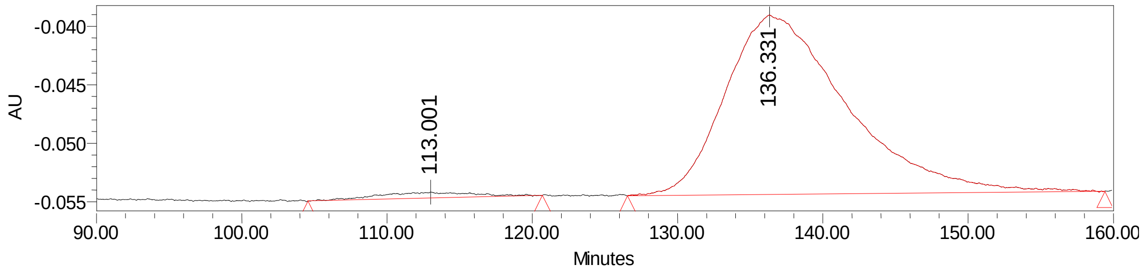

	Retention Time	% Area
1	113.001	2.41
2	136.331	97.59

Methyl 2-((*S*)-1-benzoyl-2-(benzylthio)-4-oxo-5-((*S*)-(1,1,3,3-tetraoxido-2*H*-benzo[*d*][1,3]dithiol-2-yl)(thiophen-2-yl)methyl)-4,5-dihydro-1*H*-imidazol-5-yl)acetate (15ge).

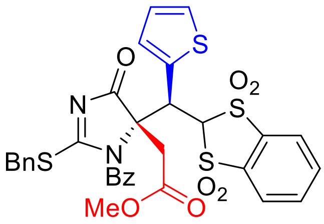

15ge

The enantiomeric purity was determined by HPLC analysis (Daicel Chiralpak IC, hexane/isopropanol 30:70, flow rate= 0.5 mL/min, retention times: 71.4 min (minor) and 89.6 min (major).

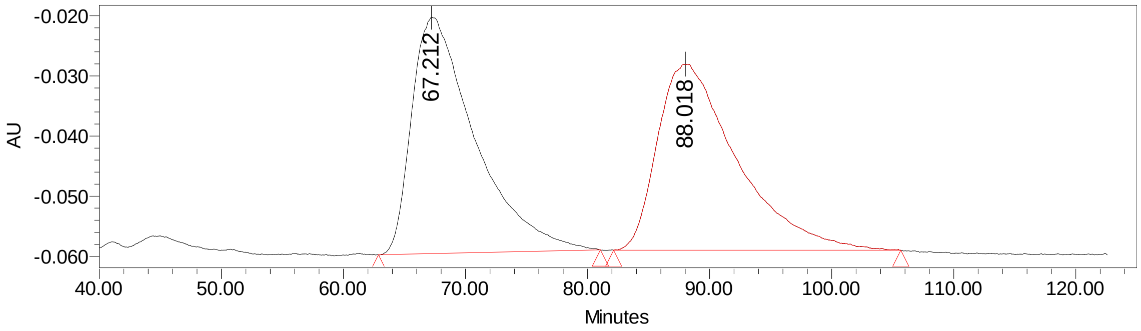

	Retention Time	% Area
1	67.212	50.29
2	88.018	49.71

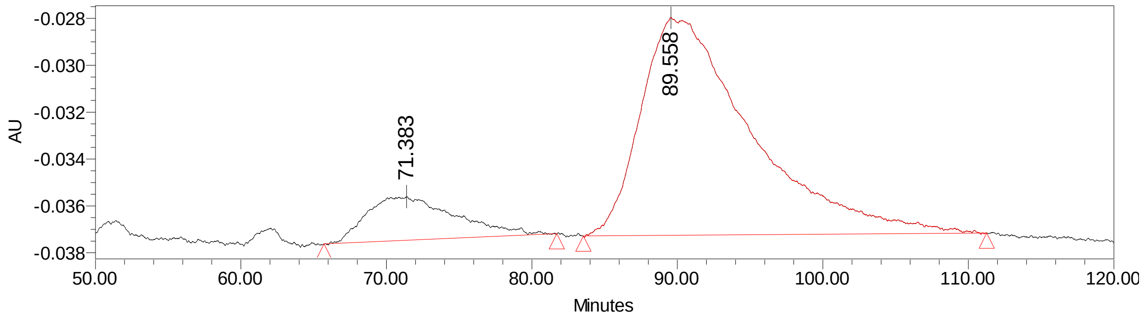

	Retention Time	% Area
1	71.383	13.82
2	89.558	86.18

(*S*)-1-Benzoyl-5-benzyl-2-(benzylthio)-5-((*S*)-pyridin-2-yl(1,1,3,3-tetraoxido-2*H*-benzo[*d*][1,3]dithiol-2-yl)methyl)-1,5-dihydro-4*H*-imidazol-4-one (15af).

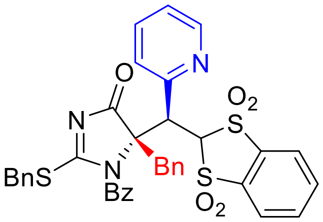

15af

The enantiomeric purity was determined by HPLC analysis (Daicel Chiralpak IC, hexane/isopropanol 30:70, flow rate= 0.5 mL/min, retention times: 86.3 min (major) and 110.8 min (minor).

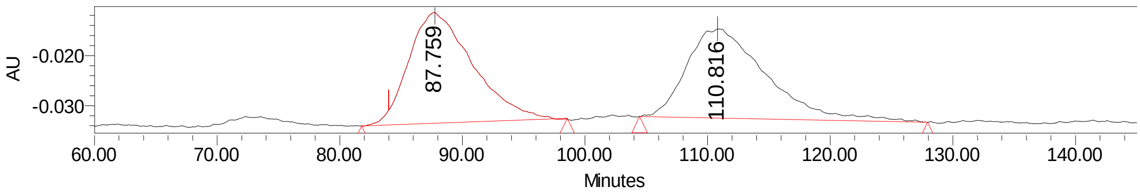

	Retention Time	% Area
1	87.759	50.03
2	110.816	49.97

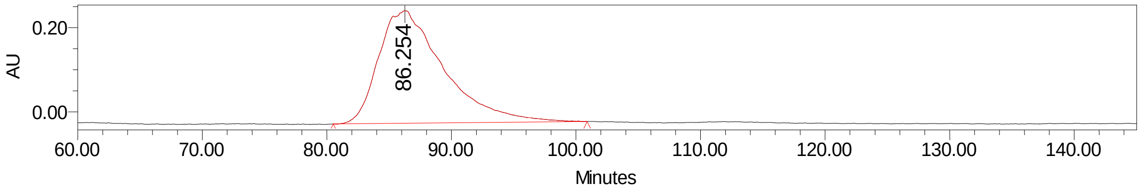

	Retention Time	% Area
1	86.254	100.00

(*S*)-5-Allyl-1-benzoyl-2-(benzylthio)-5-((*R*)-naphthalen-1-yl(1,1,3,3-tetraoxido-2*H*-benzo[*d*][1,3]dithiol-2-yl)methyl)-1,5-dihydro-4*H*-imidazol-4-one (15eg).

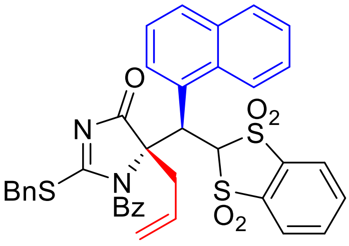

15eg

The enantiomeric purity was determined by HPLC analysis (Daicel Chiralpak IB, hexane/isopropanol 50:50, flow rate= 0.5 mL/min, retention times: 36.2 (minor) and 40.8 (major).

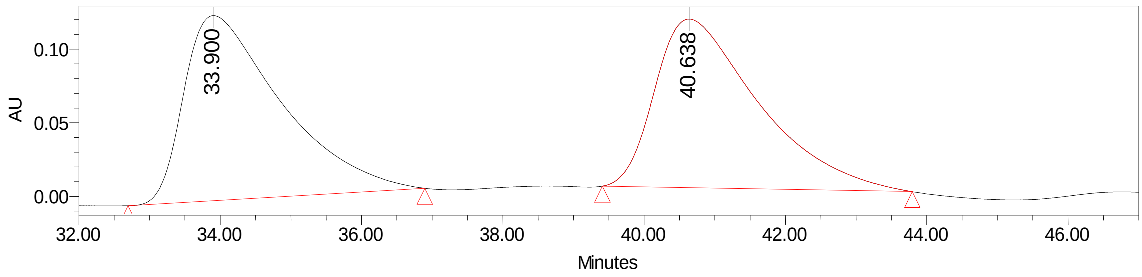

	Retention Time	% Area
1	33.900	50.70
2	40.638	49.30

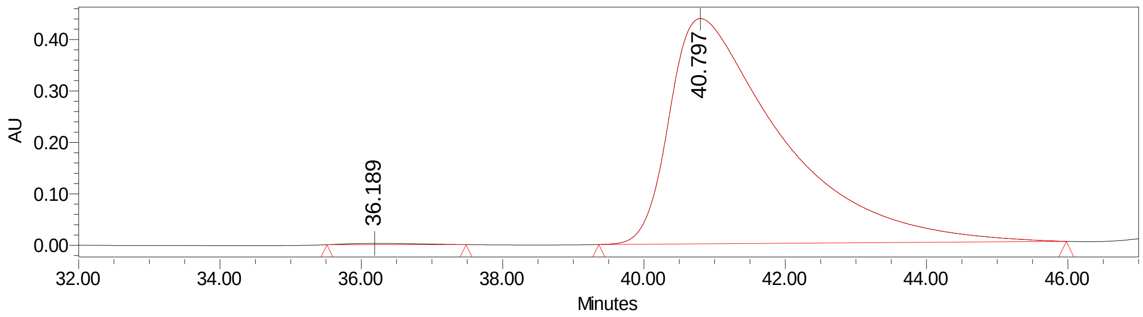

	Retention Time	% Area
1	36.189	0.35
2	40.797	99.65

(*S*)-5-Benzyl-5-((R)-naphthalen-2-yl(1,1,3,3-tetraoxido-2H-benzo[d][1,3]dithiol-2-yl)methyl)imidazolidine-2,4-dione S-2

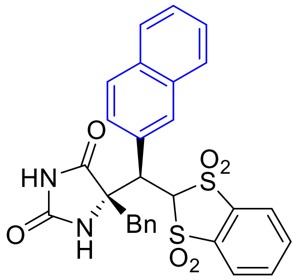

S-2 (derived from **15ah**)

The enantiomeric purity was determined by HPLC analysis (Daicel Chiralpak ID, hexane/ethanol 30:70, flow rate= 0.5 mL/min, retention times: 17,5 min (min.) and 32,4 min (major.).

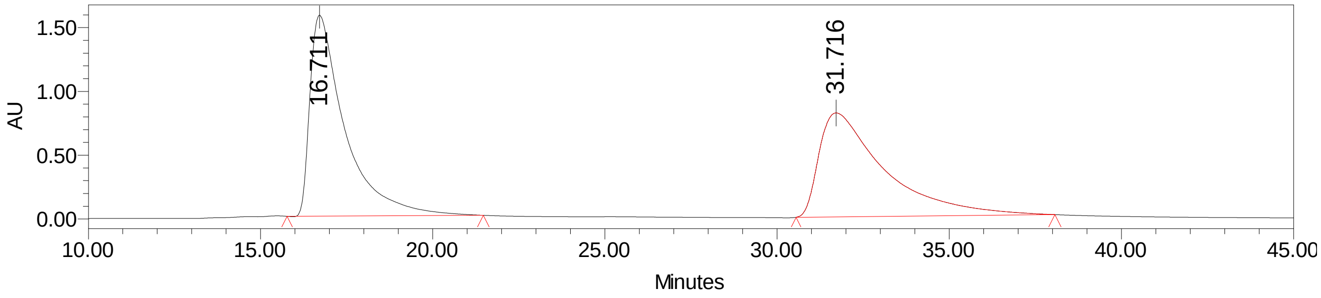

	Retention Time	% Area
1	16.711	50.82
2	31.716	49.18

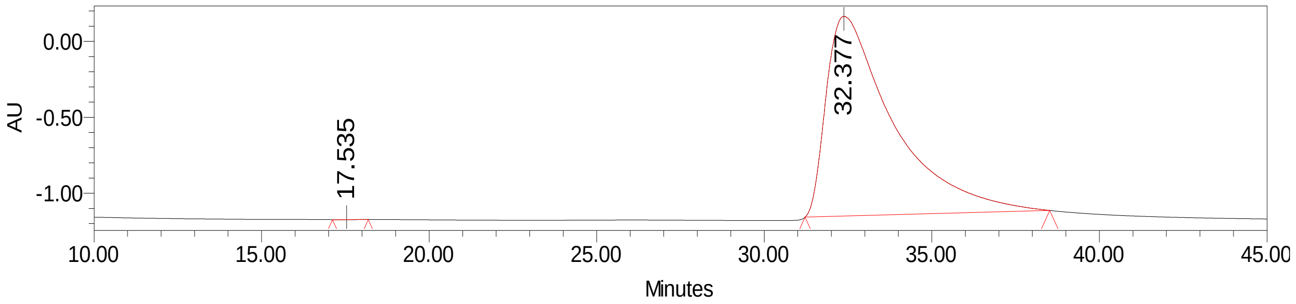

	Retention Time	% Area
1	17.535	0.01
2	32.377	99.99

(*S*)-5-Benzyl-2-(benzylthio)-5-((*R*)-phenyl(1,1,3,3-tetraoxido-2*H*-benzo[*d*][1,3]dithiol-2-yl)methyl)-1-(2-phenylacetyl)-1,5-dihydro-4*H*-imidazol-4-one (18).

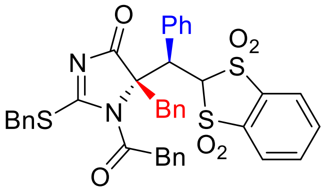

18

The enantiomeric purity was determined by HPLC analysis (Daicel Chiralpak IA, hexane/isopropanol 50:50, flow rate= 0.5 mL/min, retention times: 51.8 min (minor) and 60.7 min (major).

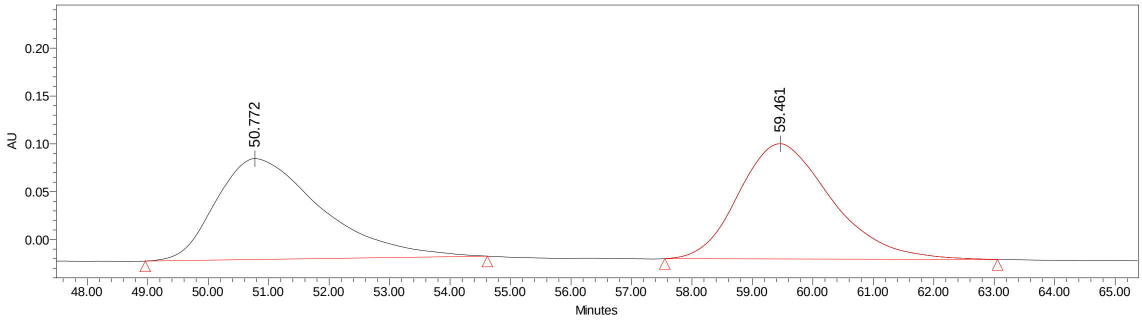

	Retention Time	% Area
1	50.772	49.71
2	59.461	50.29

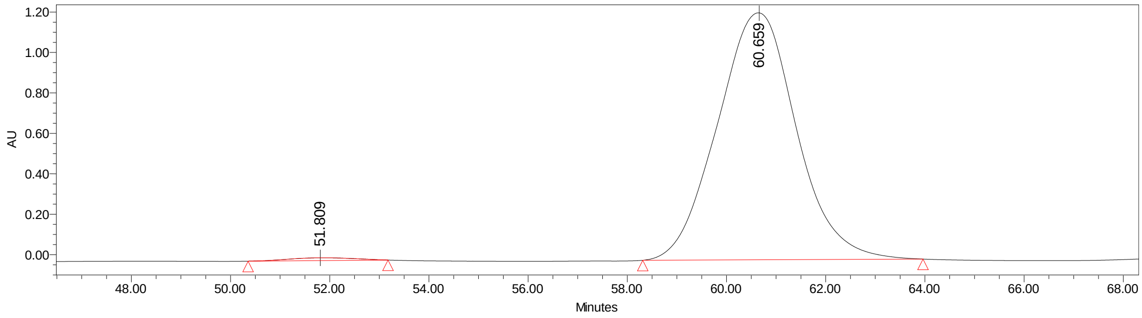

	Retention Time	% Area
1	51.809	0.93
2	60.659	99.07

(*S*)-1-Benzoyl-2-(benzylthio)-5-((*R*)-phenyl(1,1,3,3-tetraoxido-2*H*-benzo[*d*][1,3]dithiol-2-yl)methyl)-1,5-dihydro-4*H*-imidazol-4-one (22a)

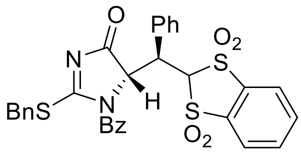

22a

The enantiomeric purity was determined by HPLC analysis (Daicel Chiralpak IF, hexane/ethanol 30:70, flow rate= 0.5 mL/min, retention times: 46.4 min (minor) and 78.0 min (major).

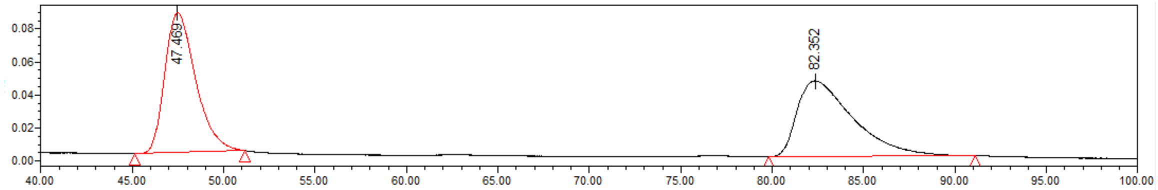

	Retention Time	% Area
1	47.469	50.36
2	82.352	49.64

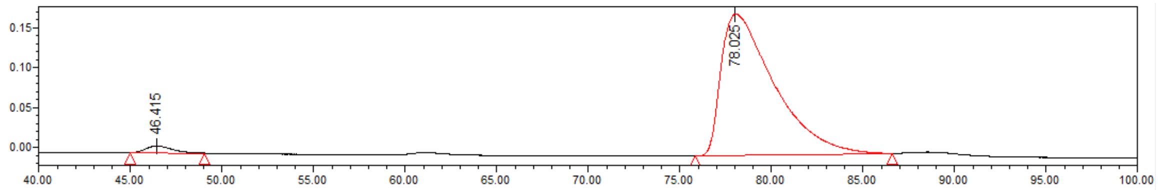

	Retention Time	% Area
1	46.415	2.40
2	78.025	97.60

(*S*)-1-Benzoyl-2-(benzylthio)-5-((*R*)-(4-chlorophenyl)(1,1,3,3-tetraoxido-2*H*-benzo[*d*][1,3]dithiol-2-yl)methyl)-1,5-dihydro-4*H*-imidazol-4-one (22b)

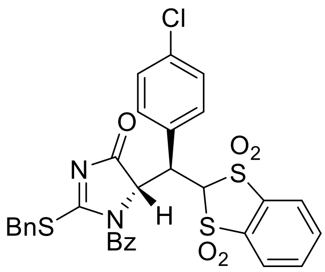

22b

The enantiomeric purity was determined by HPLC analysis (Daicel Chiralpak IF, hexane/ethanol 30:70, flow rate= 0.5 mL/min, retention times: 40.4 min (minor) and 76.3 min (major).

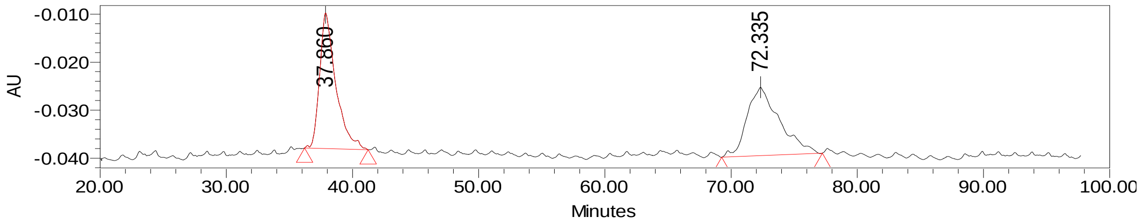

	Retention Time	% Area
1	37.860	49.72
2	72.335	50.28

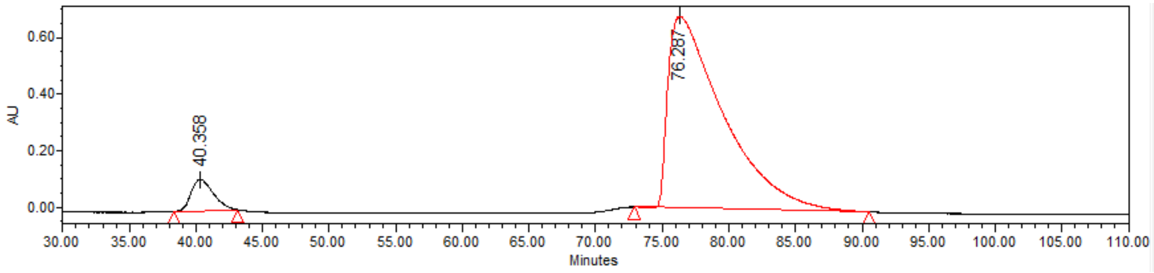

	Retention Time	% Area
1	40.358	6.15
2	76.287	93.85

(*S*)-5-Benzyl-5-((*R*)-1-phenylethyl)imidazolidine-2,4-dione (26).

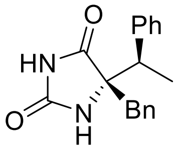

26

The enantiomeric purity was determined by HPLC analysis (Daicel Chiralpak IC, hexane/isopropanol 90:10, flow rate= 0.5 mL/min, retention times: 11.4 min (minor) and 16.6 min (major).

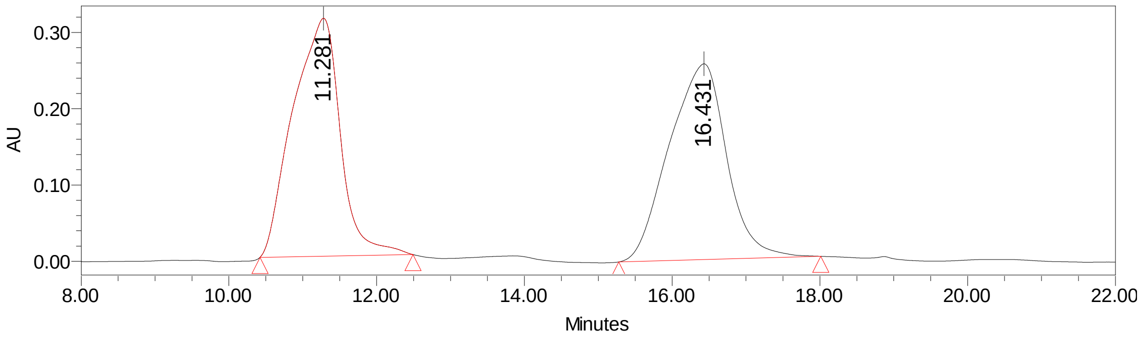

	Retention Time	% Area
1	11.281	50.12
2	16.431	49.88

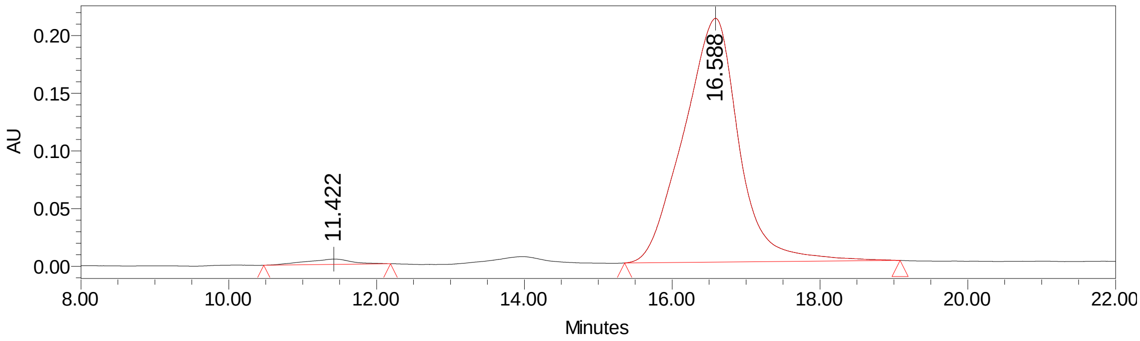

	Retention Time	% Area
1	11.422	1.80
2	16.588	98.20

(*S*)-1,5-Dibenzyl-5-((*R*)-phenyl(1,1,3,3-tetraoxido-2*H*-benzo[*d*][1,3]dithiol-2-yl)methyl)imidazolidine-2,4-dione (32c).

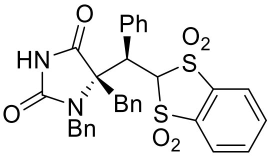

32c

The enantiomeric purity was determined by HPLC analysis (Daicel Chiralpak IA, hexane/isopropanol 30:70, flow rate= 0.5 mL/min, retention times: 60.6 min (major) and 77.2 min (minor).

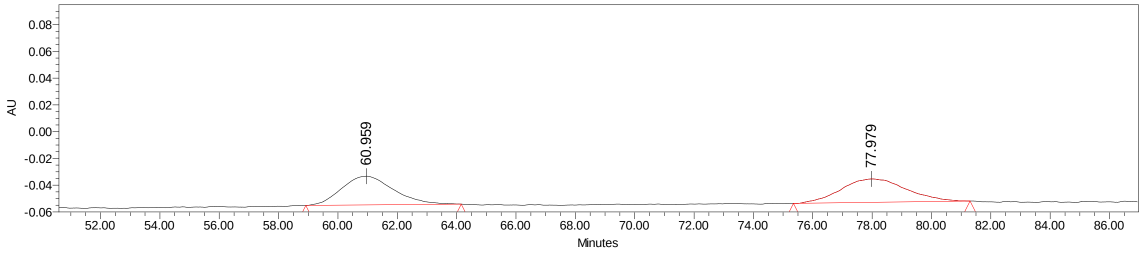

	Retention Time	% Area
1	60.959	49.33
2	77.979	50.67

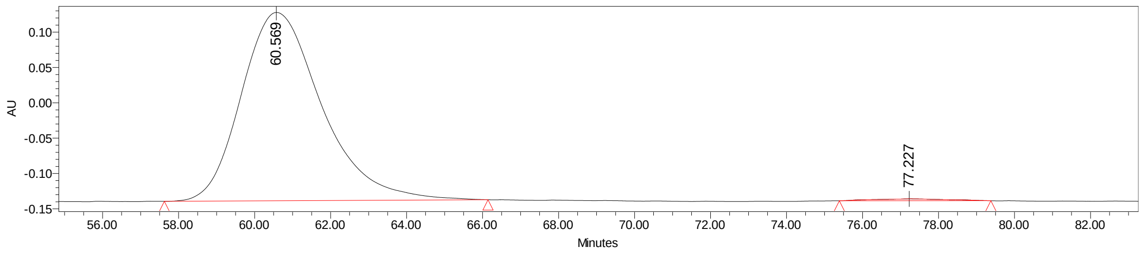

	Retention Time	% Area
1	60.569	99.09
2	77.227	0.91

7. High Resolution Mass Spectra (HRMS)

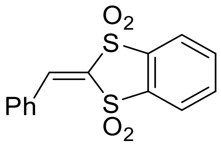

2a

HRMS (ESI) m/z: $[M + Na]^+$ calcd. for $C_{14}H_{10}O_4S_2Na$ 328.9918; found 328.9924

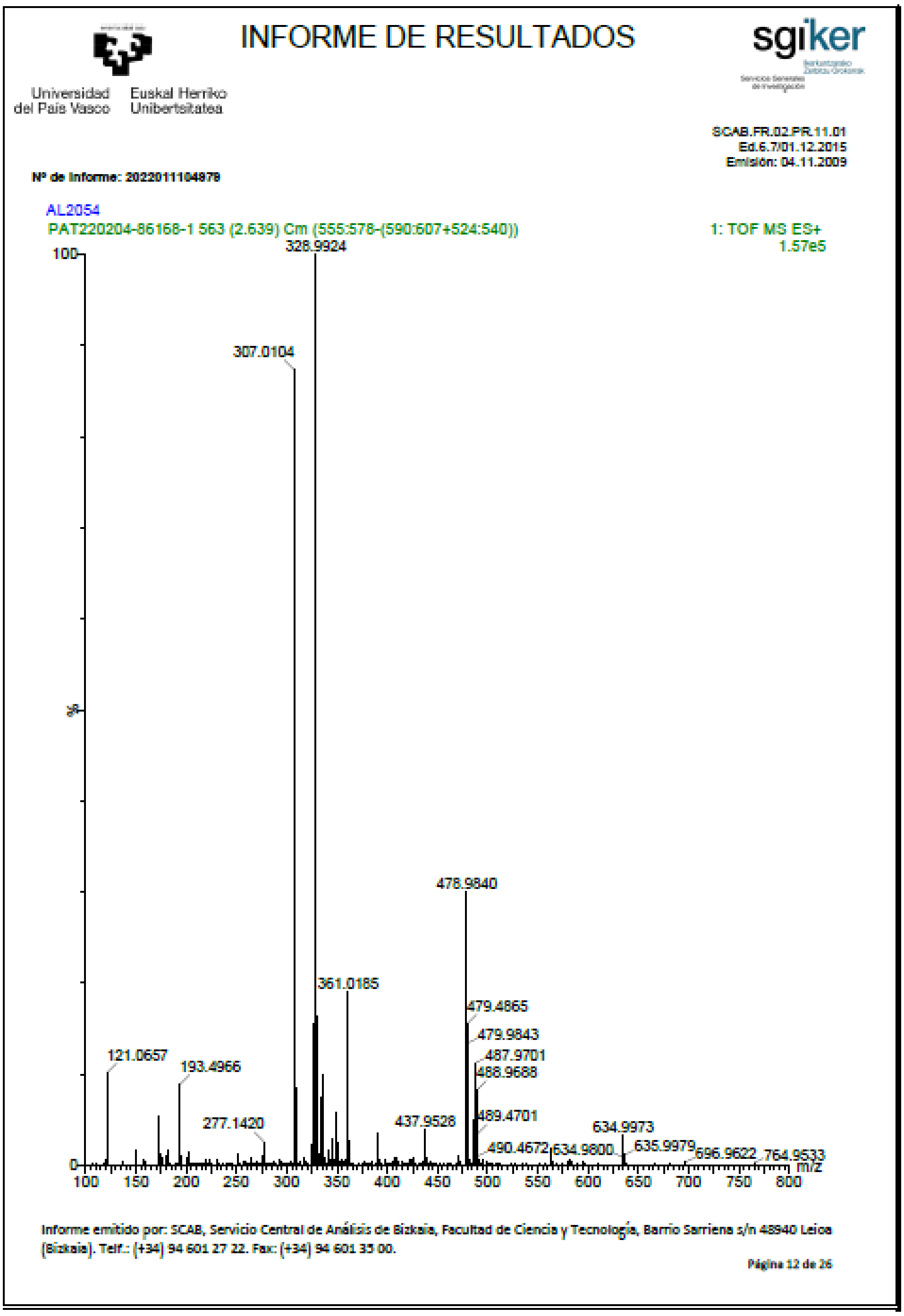

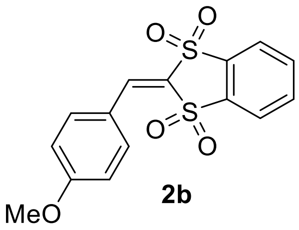

2b

HRMS (ESI) m/z: [M + H] $^{+}$ calcd. for $C_{15}H_{13}O_5S_2$ 337.0204; found 337.0204.

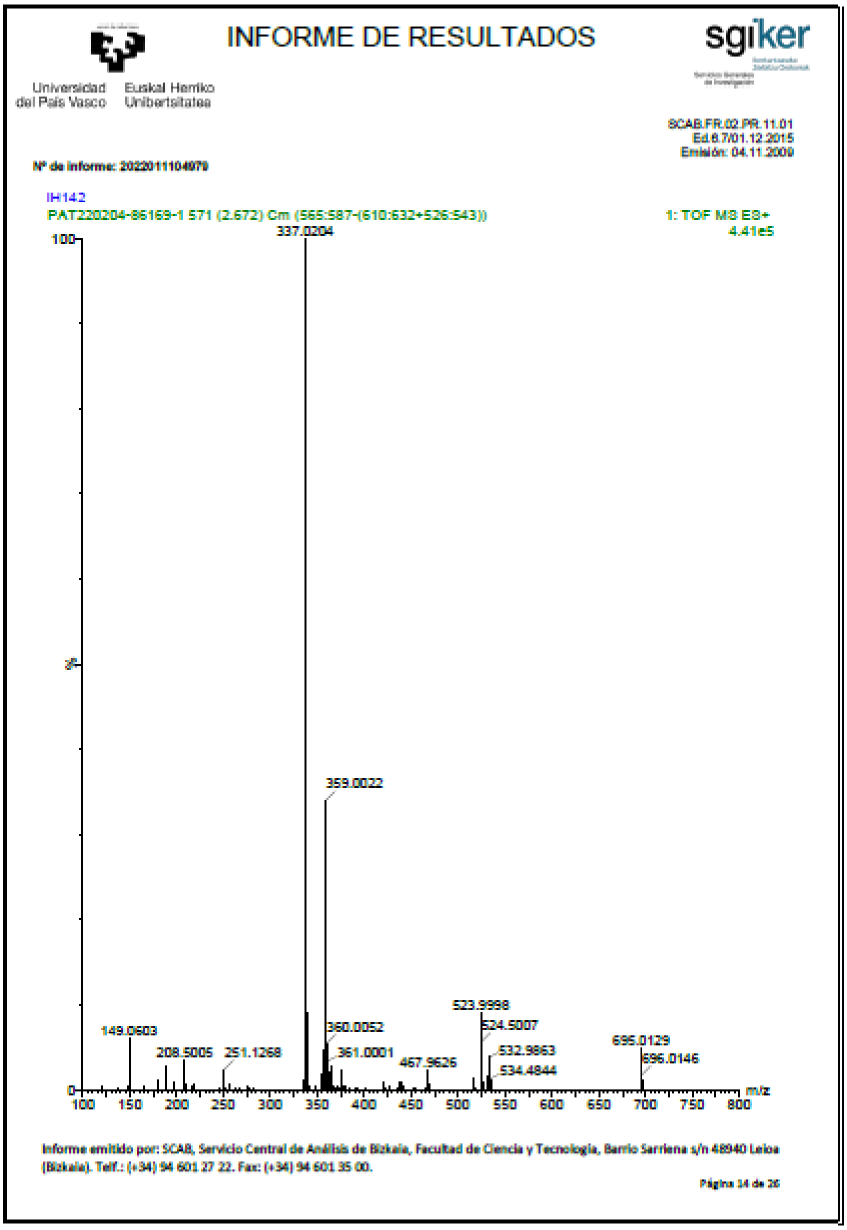

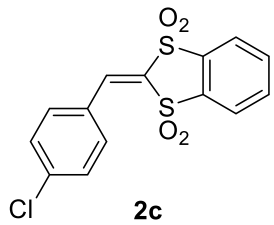

2c

HRMS (ESI) m/z: [M + Na] $^{+}$ calcd. for $C_{14}H_9ClO_4S_2Na$ 362.9529; found 362.9535.

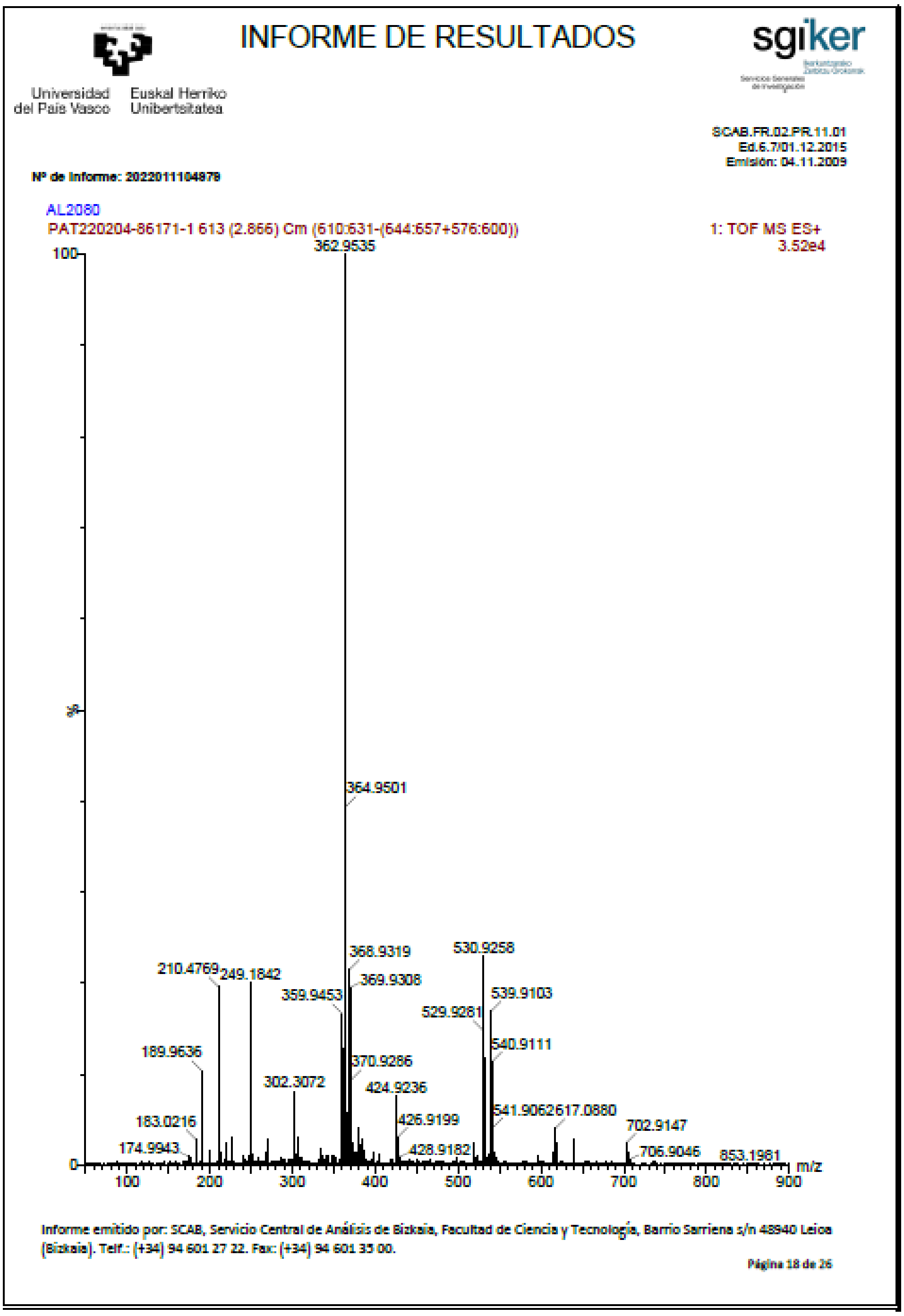

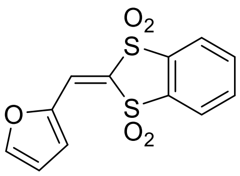

2d

HRMS (ESI) m/z: [M + H] $^+$ calcd. for $C_{12}H_9O_5S_2$ 296.9891; found 296.9895.

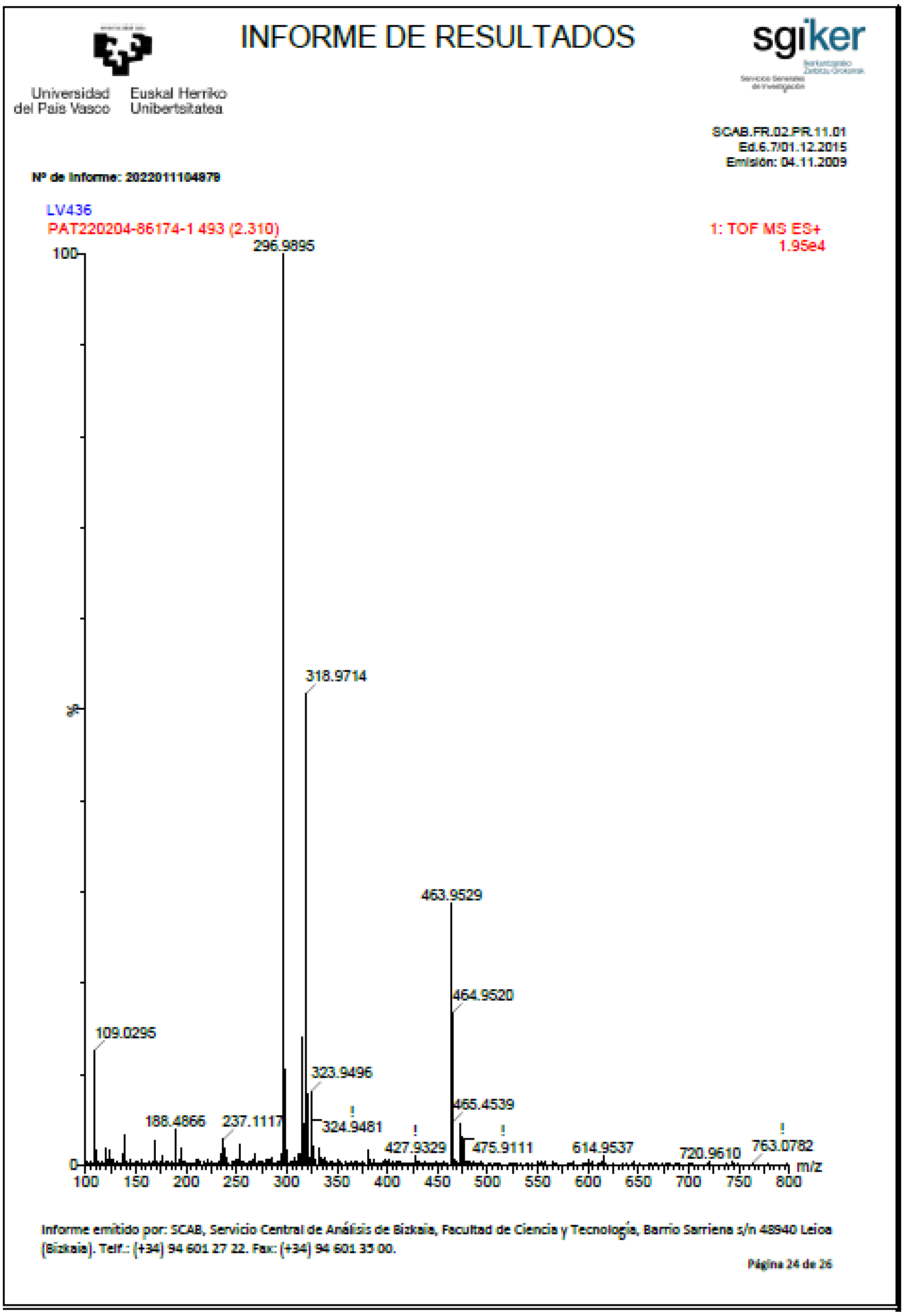

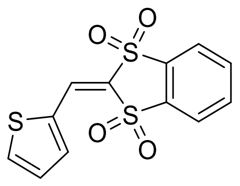

2e

HRMS (ESI) m/z: [M + H] $^+$ calcd. for $C_{12}H_9O_4S_3$ 312.9663; found 312.9667.

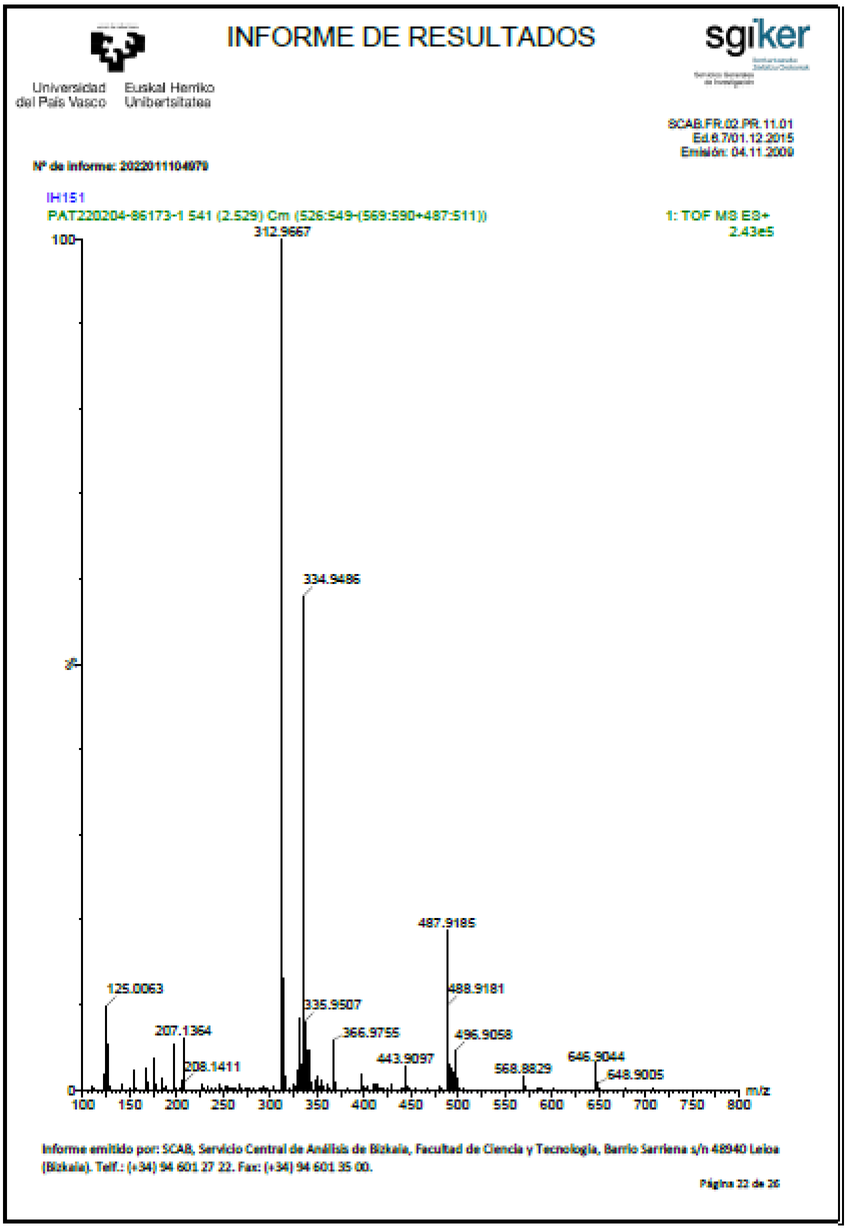

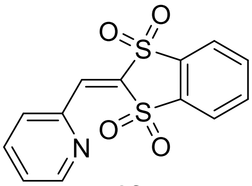

2f

HRMS (ESI) m/z: [M + H] $^{+}$ calcd. for $C_{13}H_{10}NO_4S_2$ 308.0051; found 308.0057.

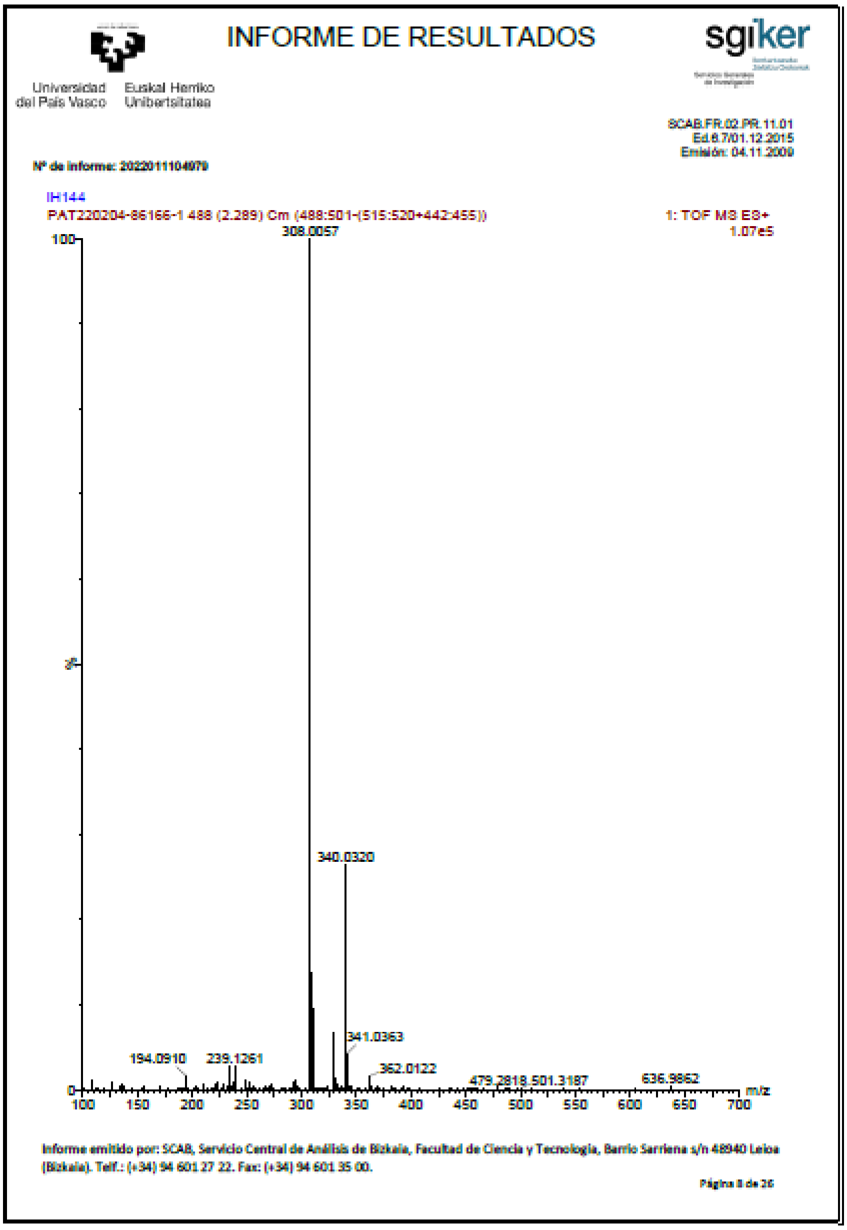

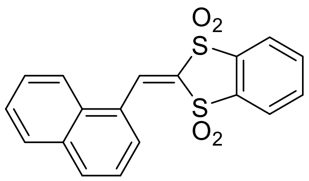

2g

HRMS (ESI) m/z: $[M+H]^+$ calcd. for $C_{18}H_{13}O_4S_2$ 357.0250; found 357.0252.

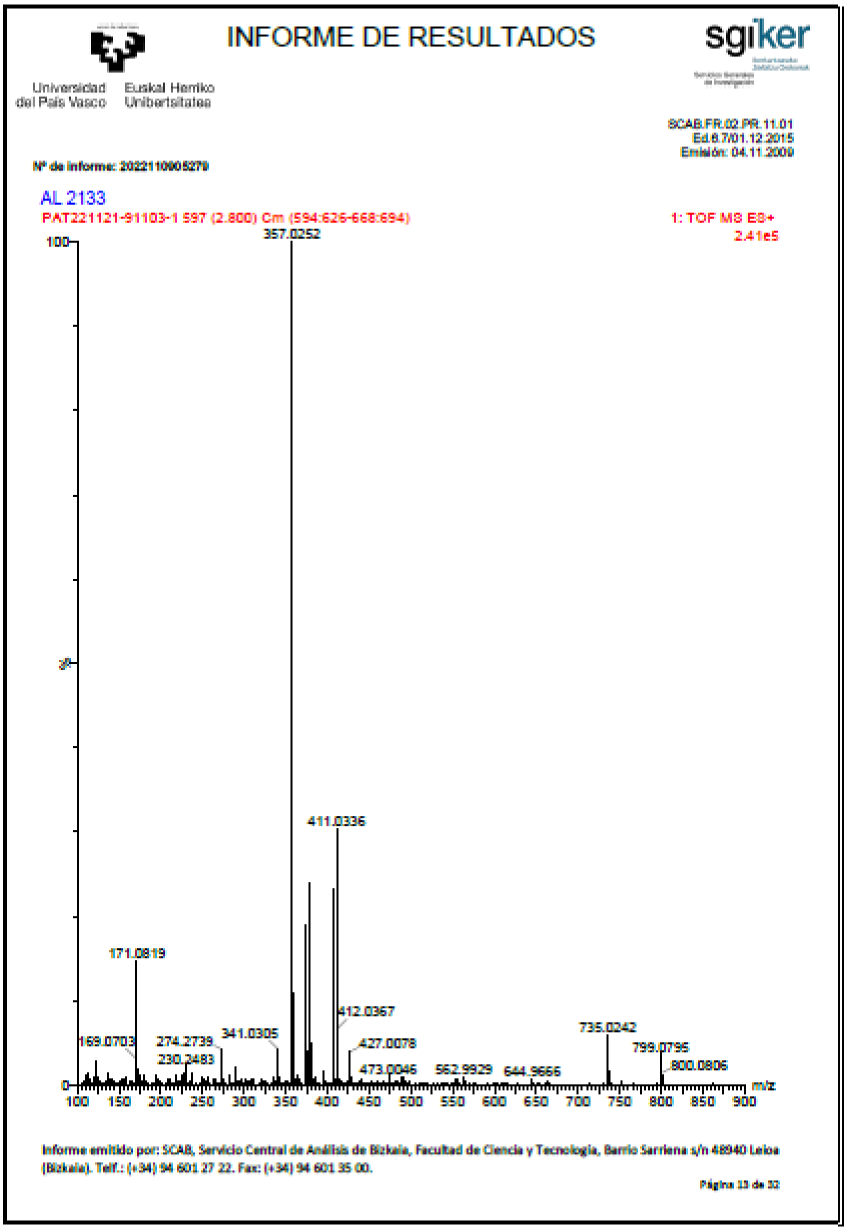

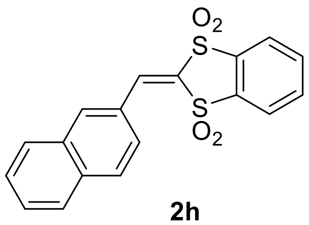

2h

HRMS (ESI) m/z: $[M+H]^+$ calcd. for $C_{18}H_{13}O_4S_2$ 357.0250; found 357.0252.

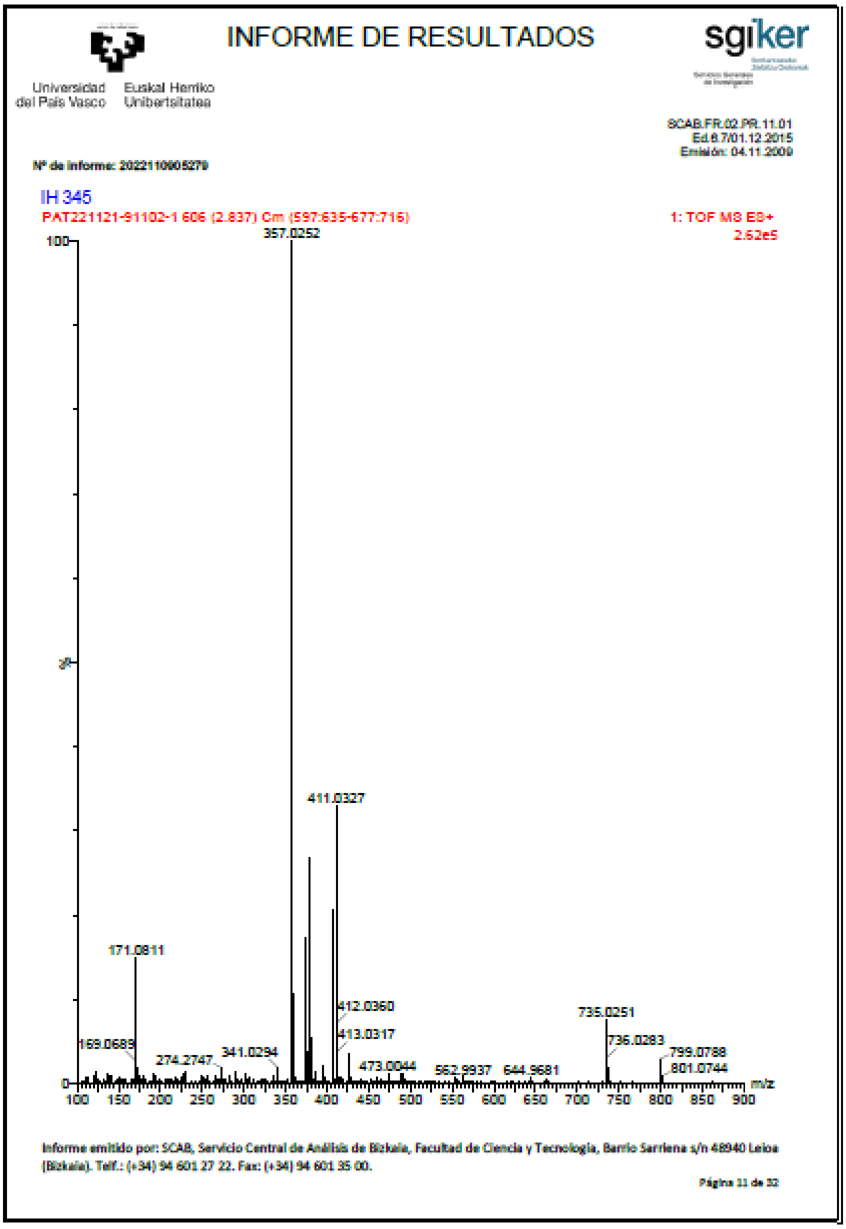

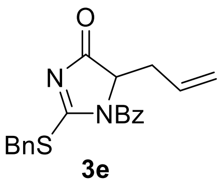

3e

HRMS (ESI) m/z: $[M + H]^+$ calcd. for $C_{20}H_{19}N_2O_2S$ 351.1162; found 351.1170.

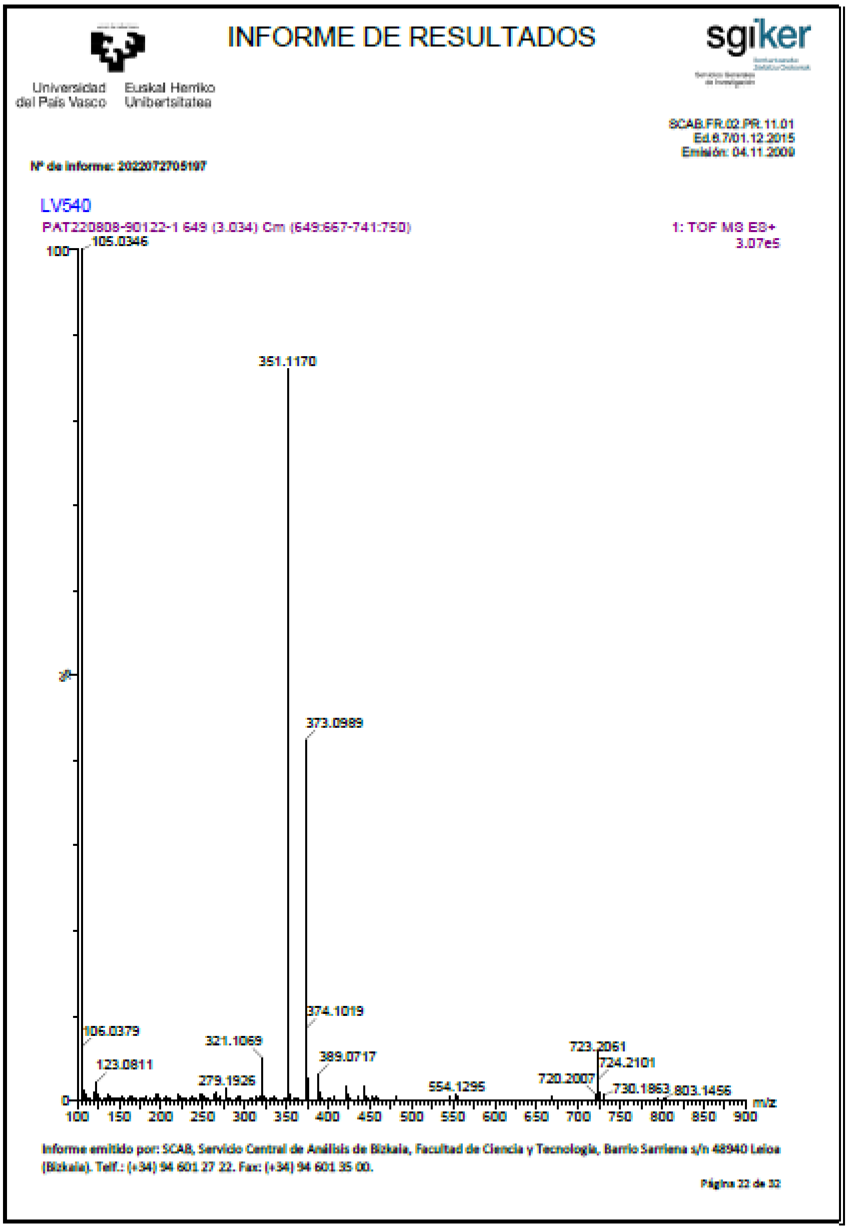

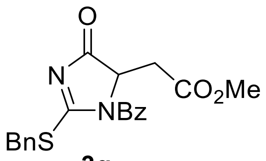

3g

HRMS (ESI) m/z: [M + H] $^{+}$ calcd. for $C_{20}H_{19}N_2O_4S$ 383.1060; found 383.1068.

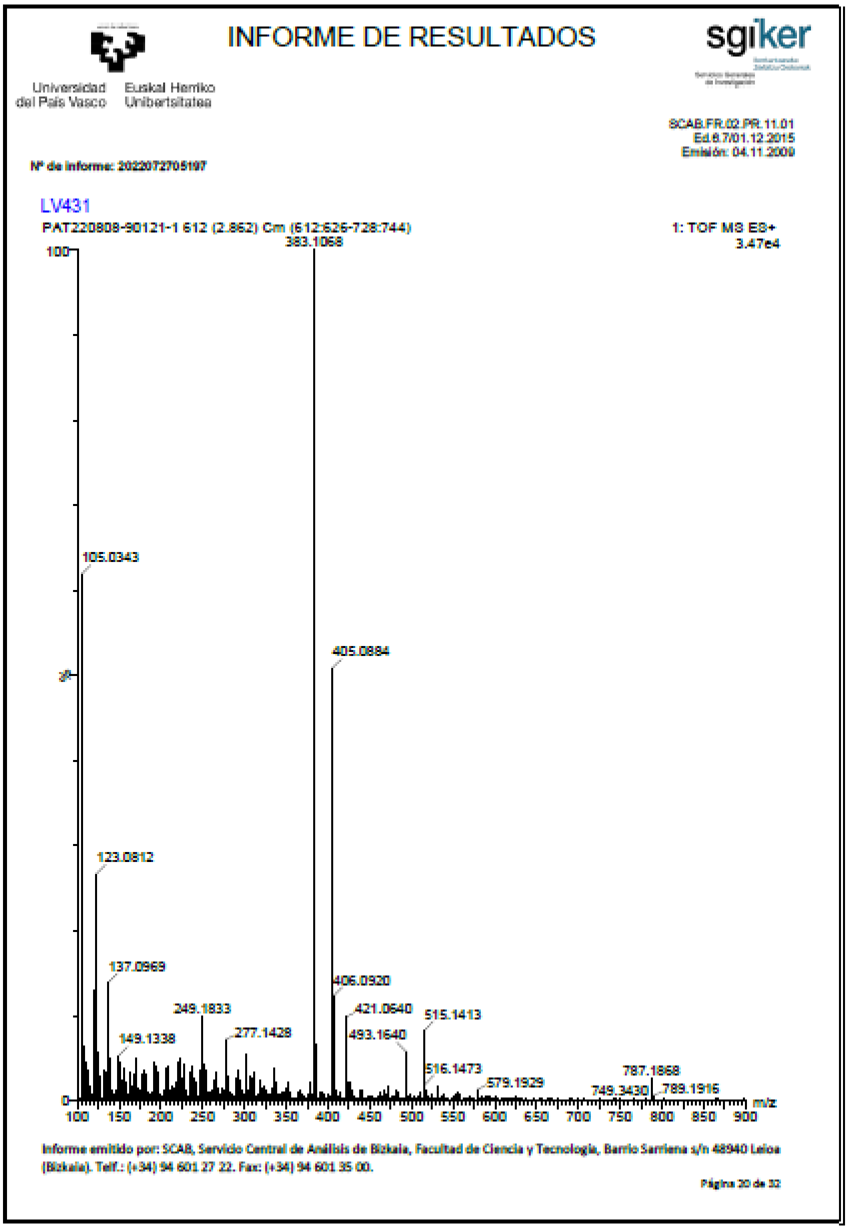

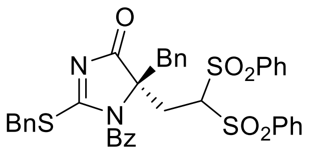

10a

HRMS (ESI) m/z: [M + H]$^+$ calcd. for $C_{38}H_{33}N_2O_6S_3$ 709.1501; found 709.1506
.

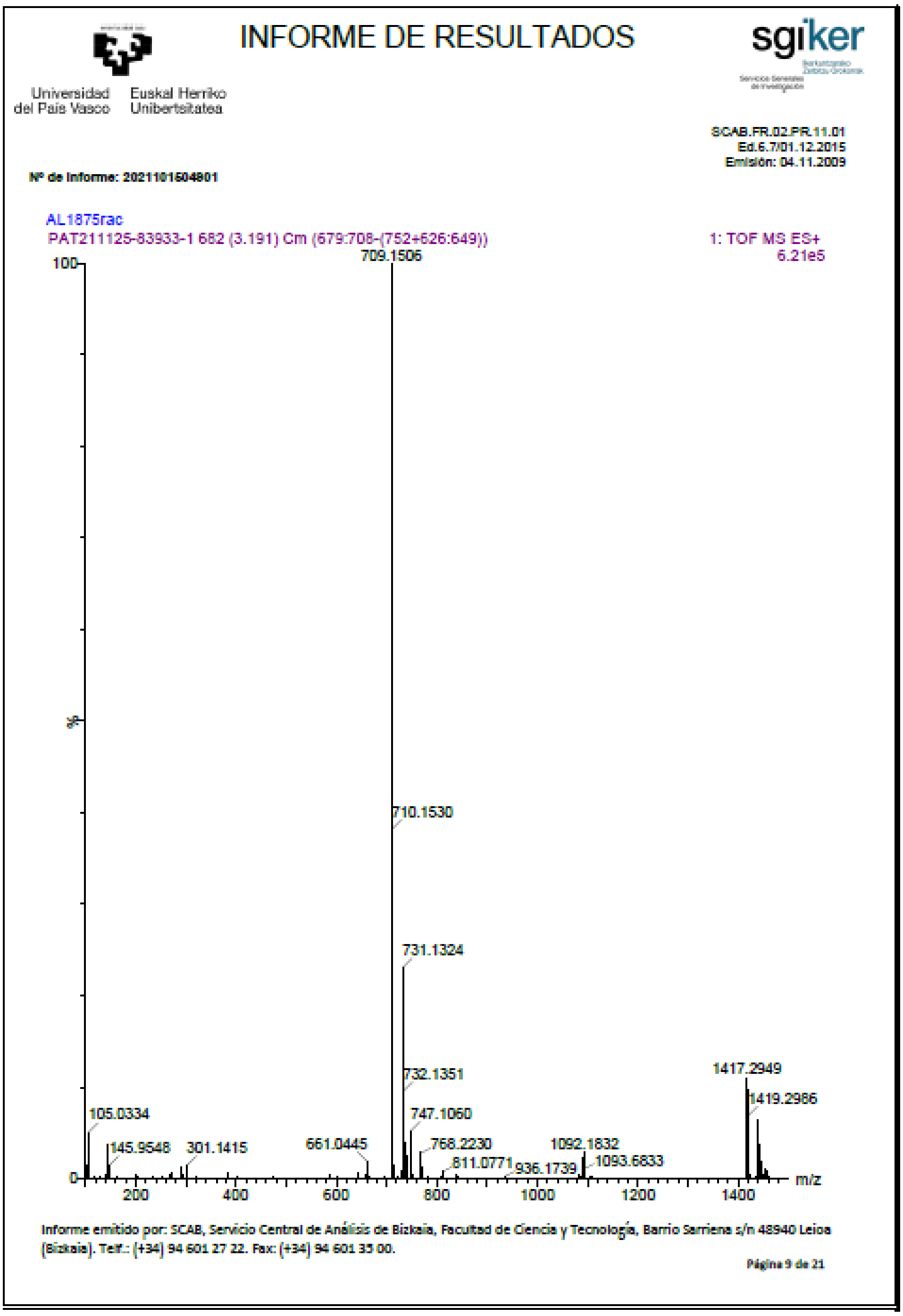

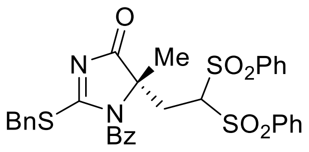

10b

HRMS (ESI) m/z: [M + H] $^{+}$ calcd. for $C_{32}H_{29}N_2O_6S_3$ 633.1182; found 633.1192.

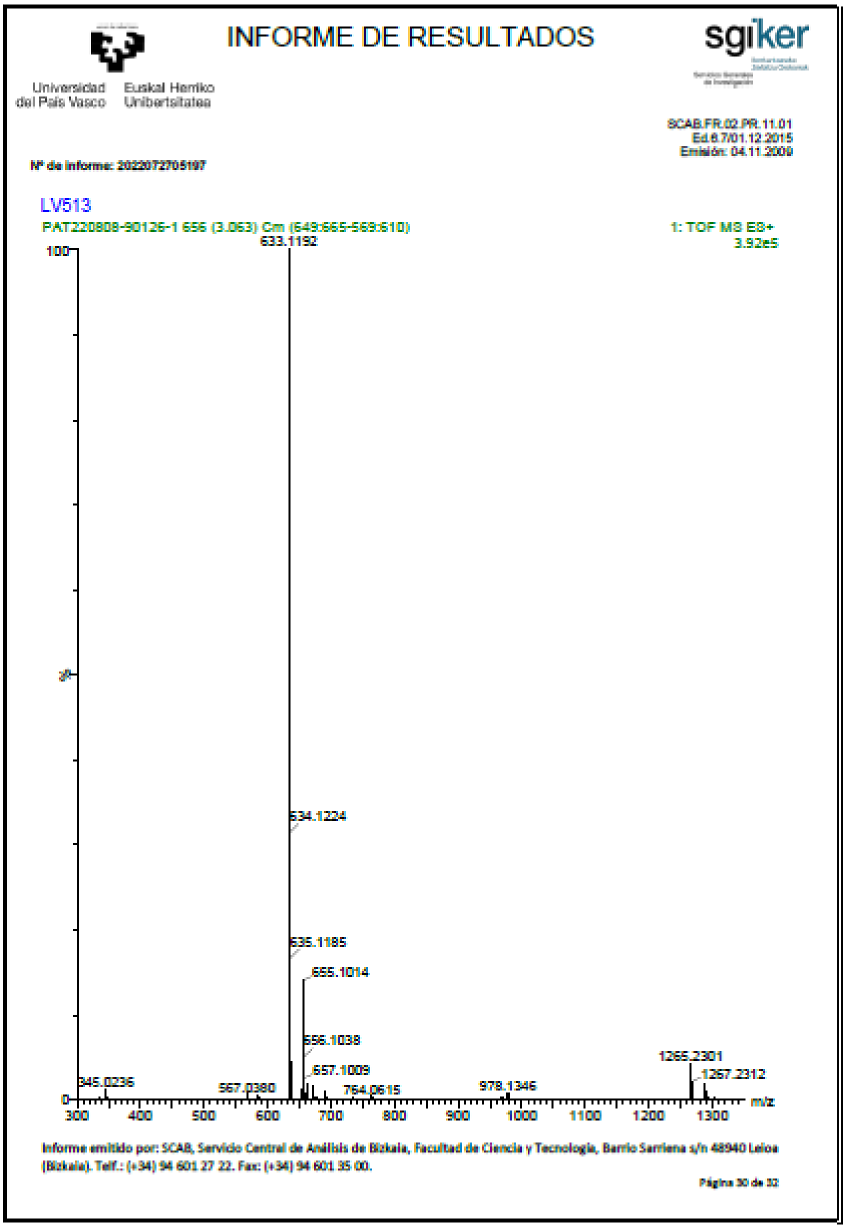

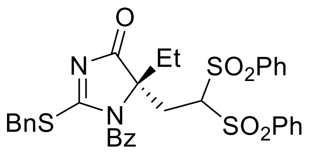

10c

HRMS (ESI) m/z: [M + H]$^{+}$ calcd. for $C_{33}H_{31}N_2O_6S_3$ 647.1344; found 647.1340.

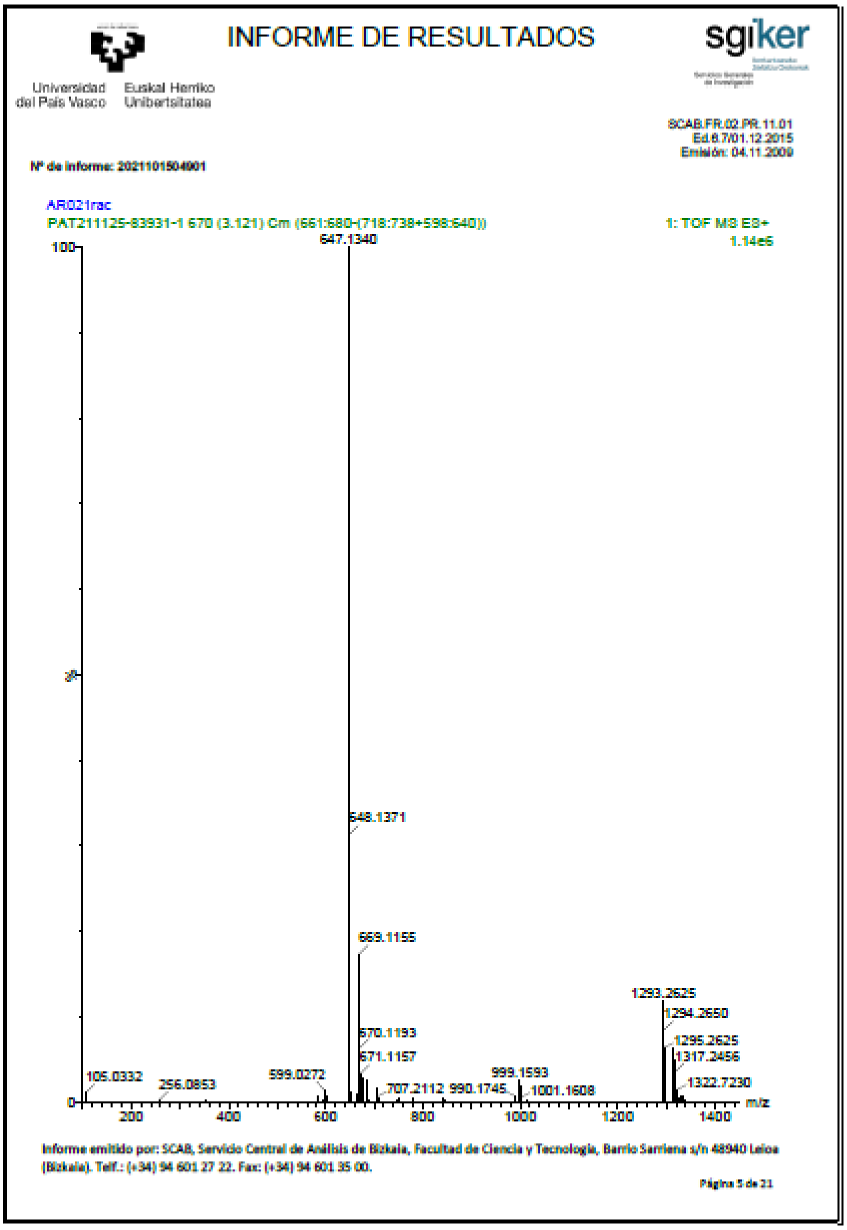

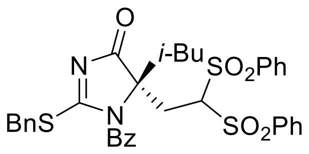

10d

HRMS (ESI) m/z: [M + H] $^{+}$ calcd. for $C_{35}H_{35}N_2O_6S_3$ 675.1657; found 675.1650.

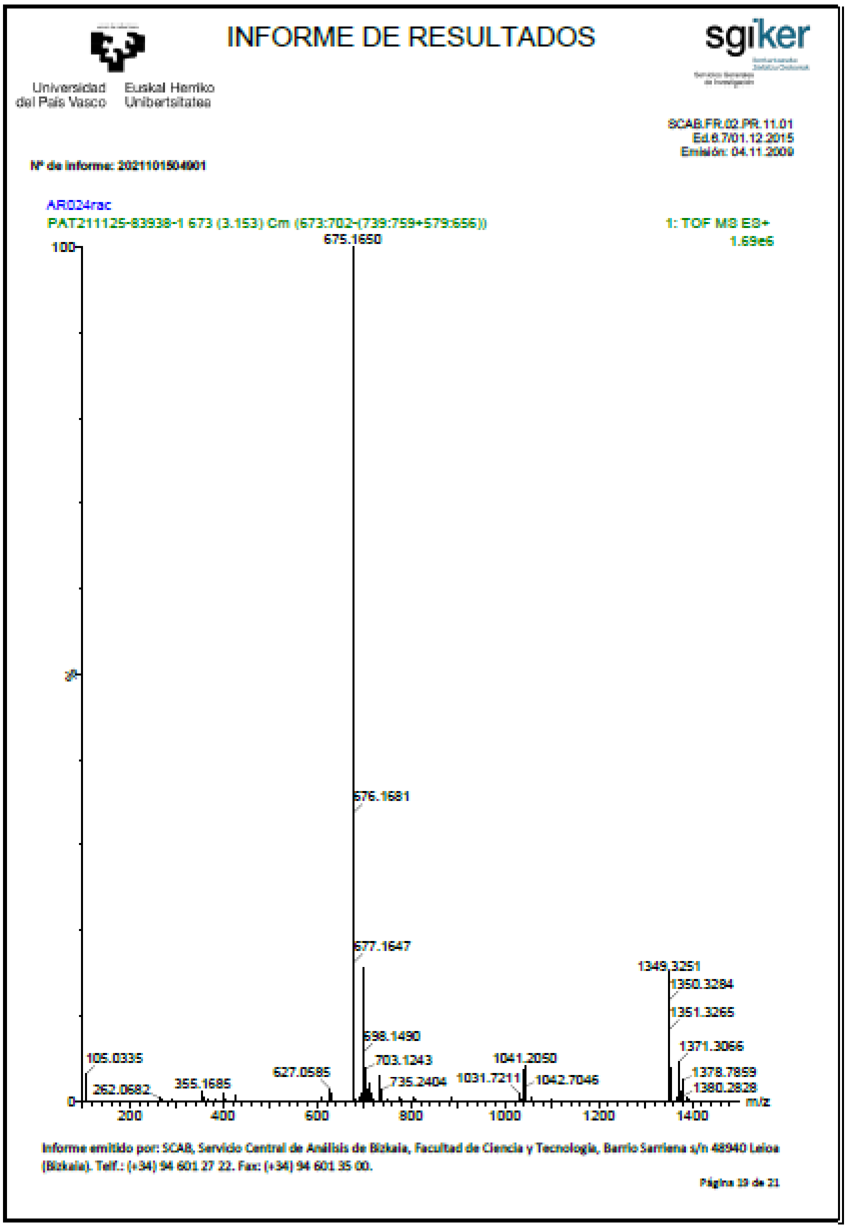

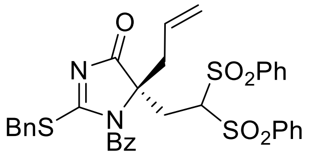

10e

HRMS (ESI) m/z: [M + H]$^+$ calcd. for $C_{34}H_{31}N_2O_6S_3$ 659.1344; found 659.1346.

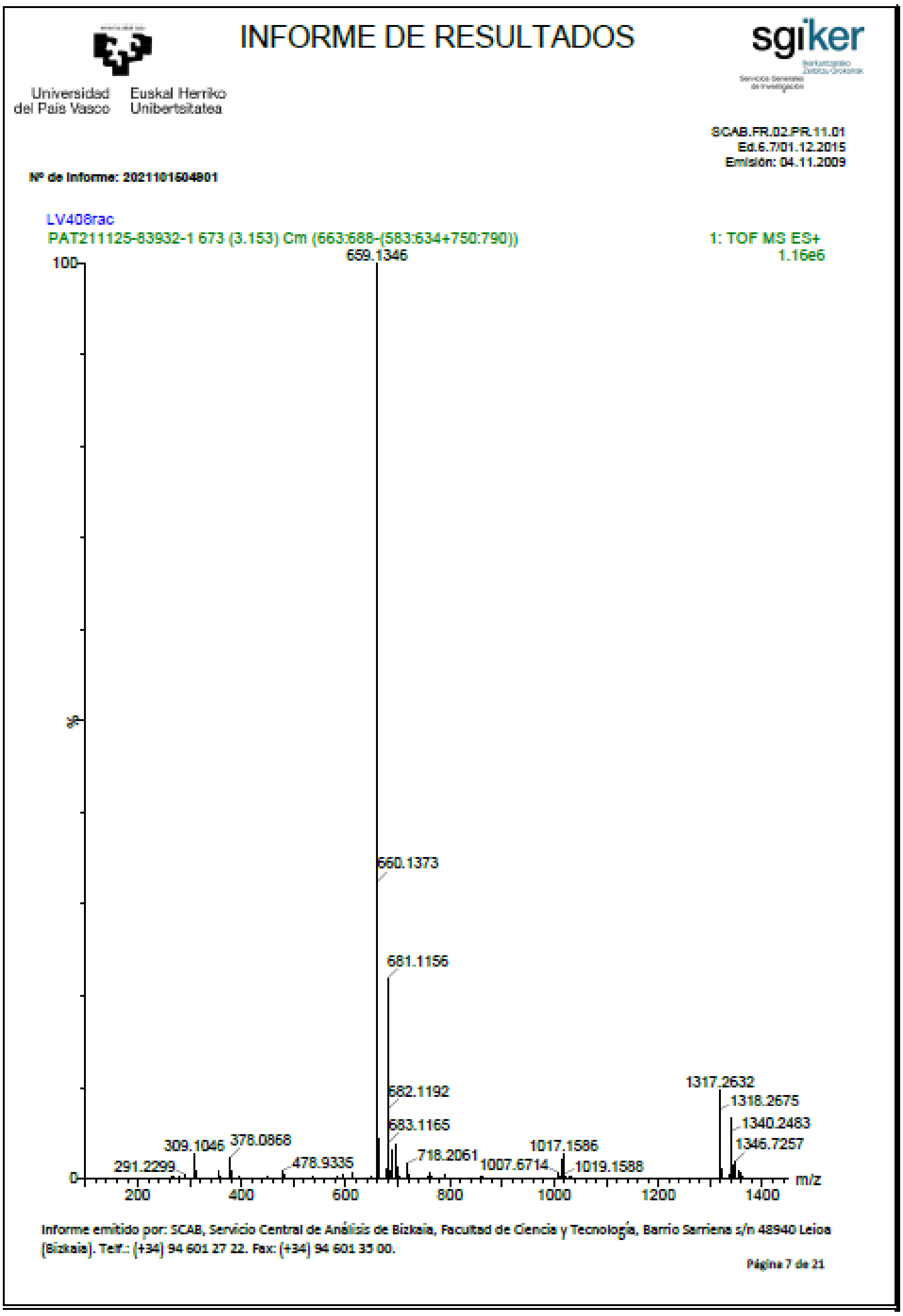

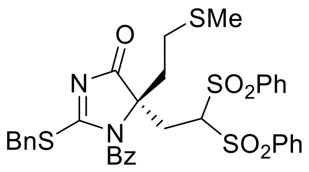

10f

HRMS (ESI) m/z: [M + H]$^{+}$ calcd. for $C_{34}H_{33}N_{2}O_{6}S_{4}$ 693.1221; found 693.1227.

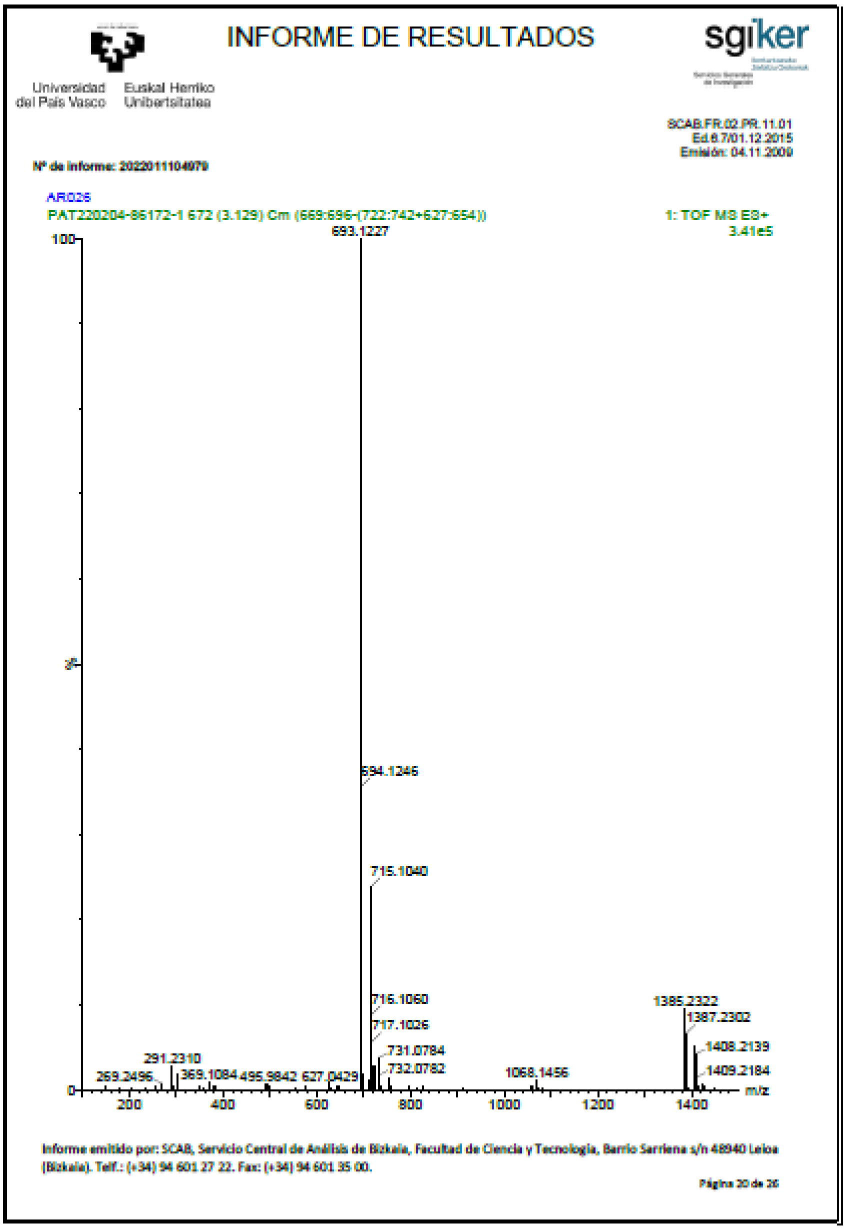

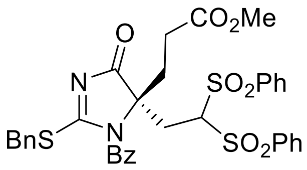

10g

HRMS (ESI) m/z: [M + H]$^+$ calcd. for $C_{34}H_{31}N_2O_8S_3$ 691.1237; found 691.1240.

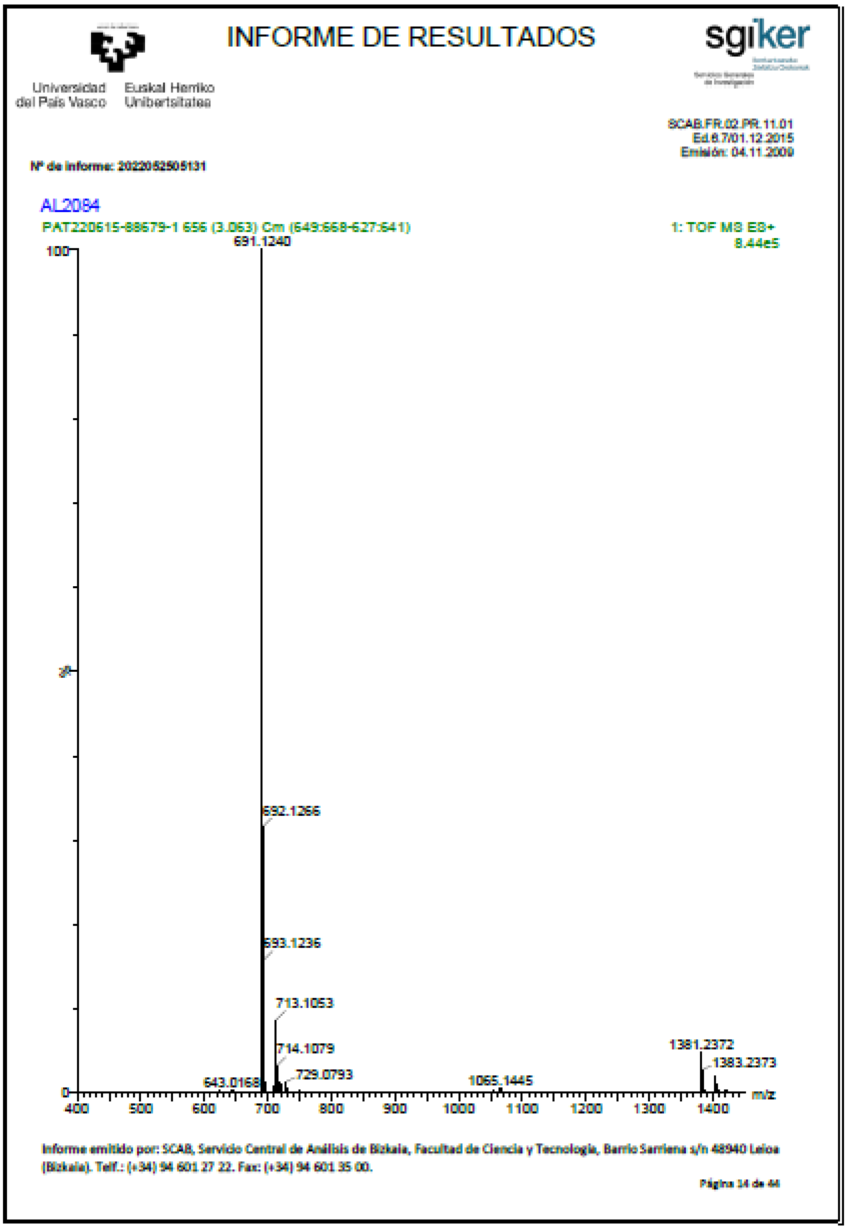

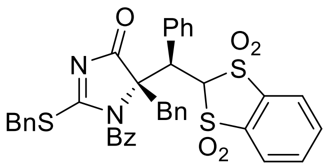

15aa

HRMS (ESI) m/z: [M + H]$^{+}$ calcd. for $C_{38}H_{31}N_{2}O_{6}S_{3}$ 707.1344; found 707.1339.

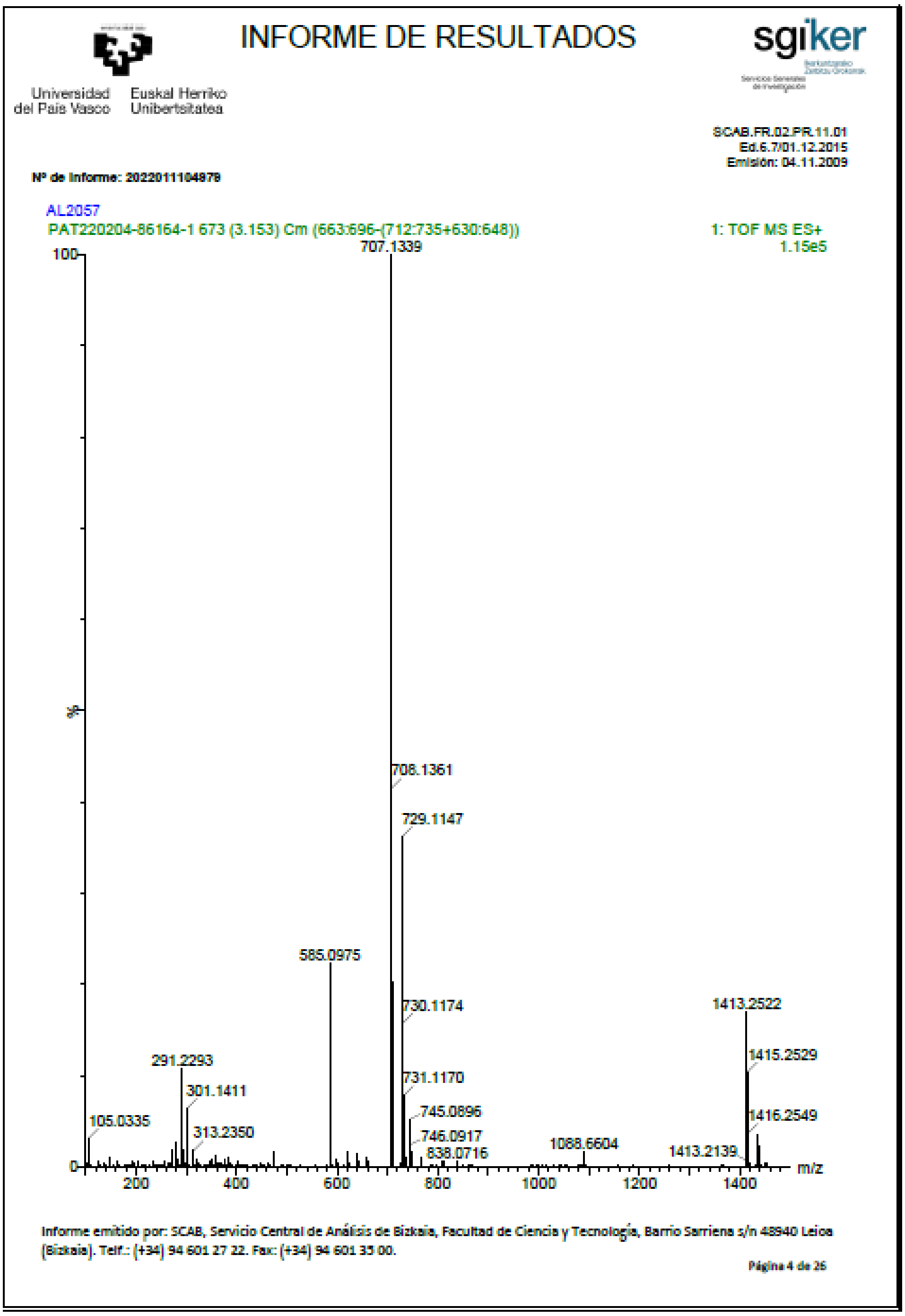

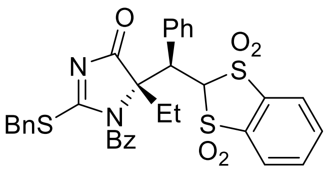

15ca

HRMS (ESI) m/z: [M + H]$^+$ calcd. for $C_{33}H_{29}N_2O_6S_3$ 645.1182; found 645.1192.

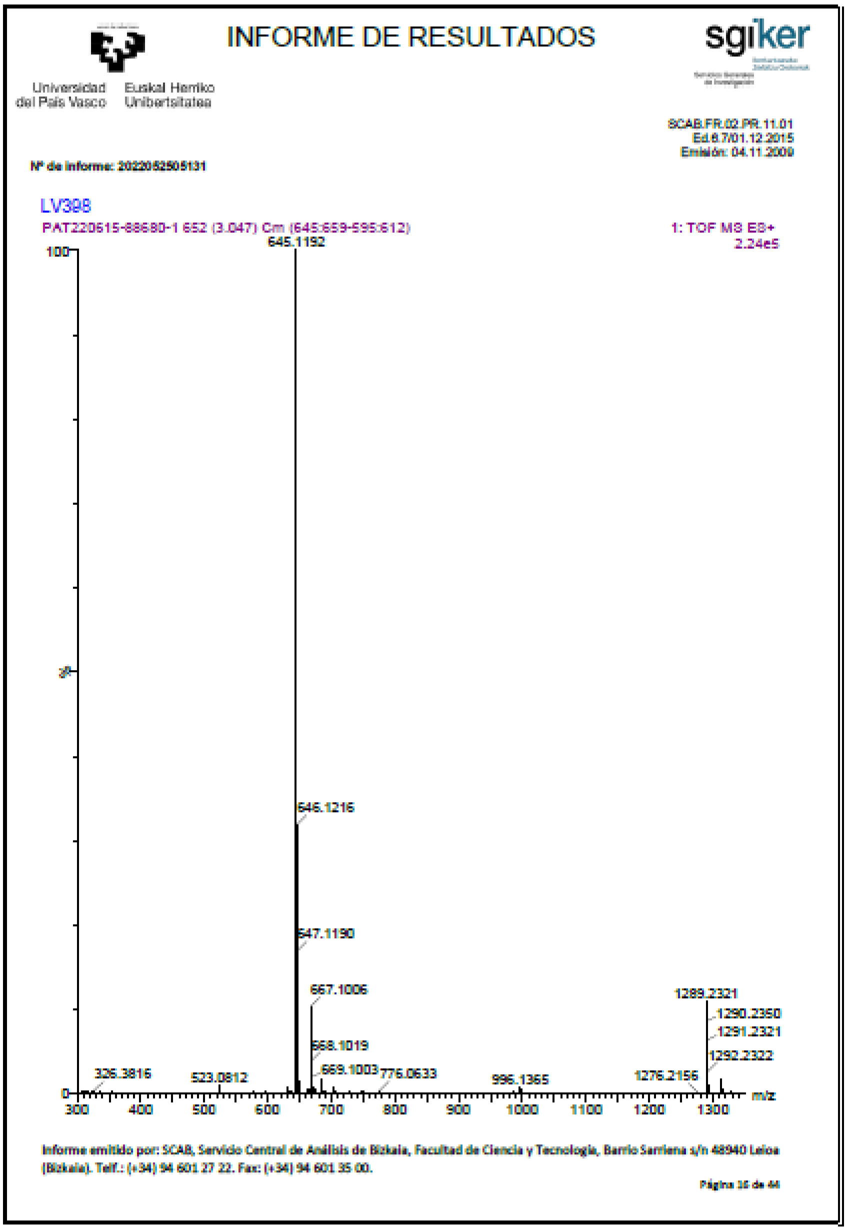

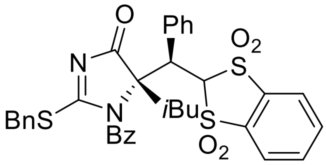

15da

HRMS (ESI) m/z: [M + H]$^+$ calcd. for $C_{35}H_{33}N_2O_6S_3$ 673.1501; found 673.1492.

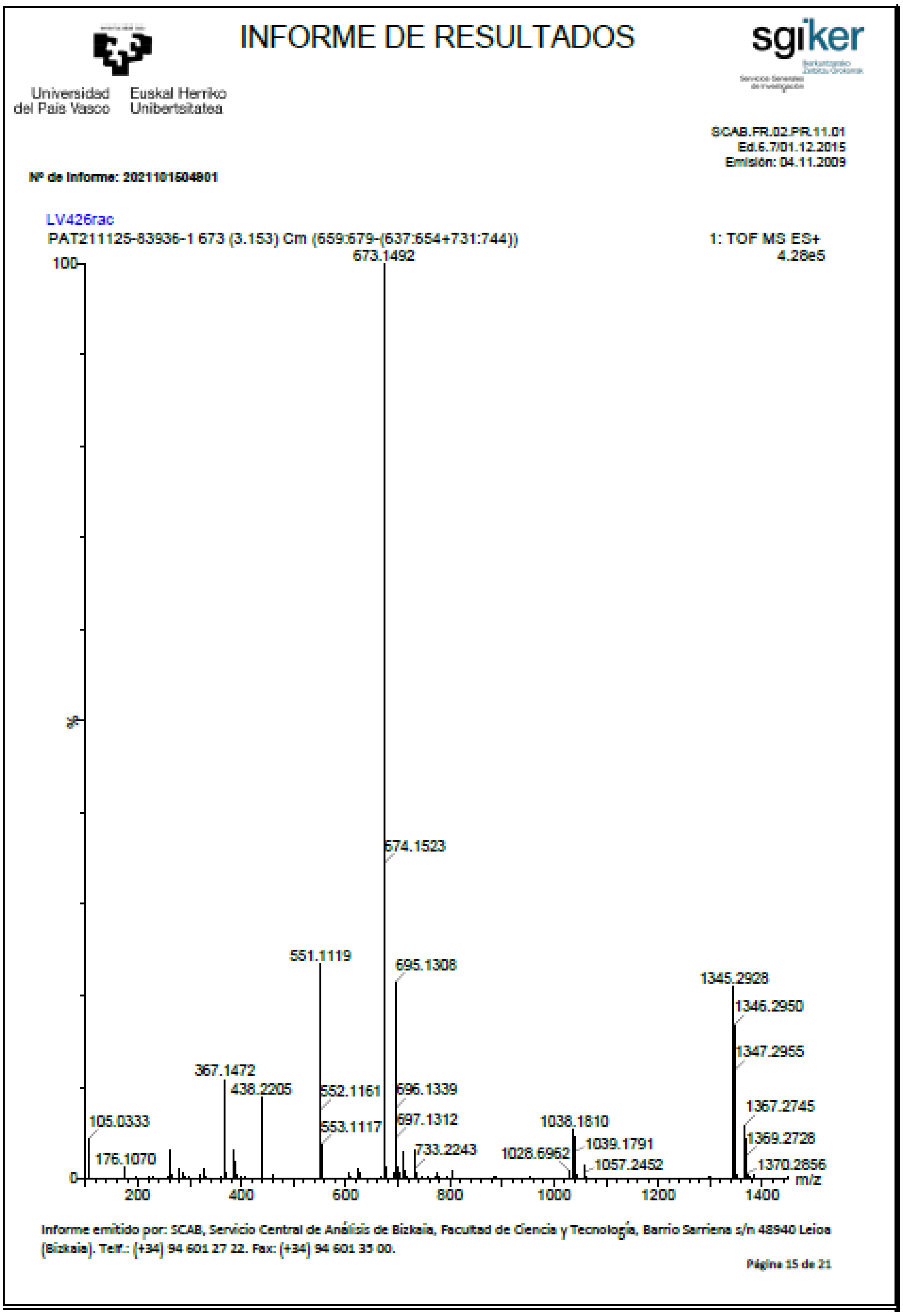

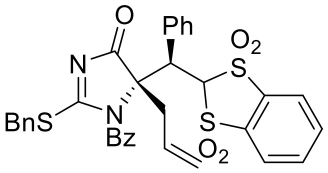

15ea

HRMS (ESI) m/z: $[M + H]^+$ calcd. for $C_{34}H_{29}N_2O_6S_3$ 657.1188; found 657.1179.

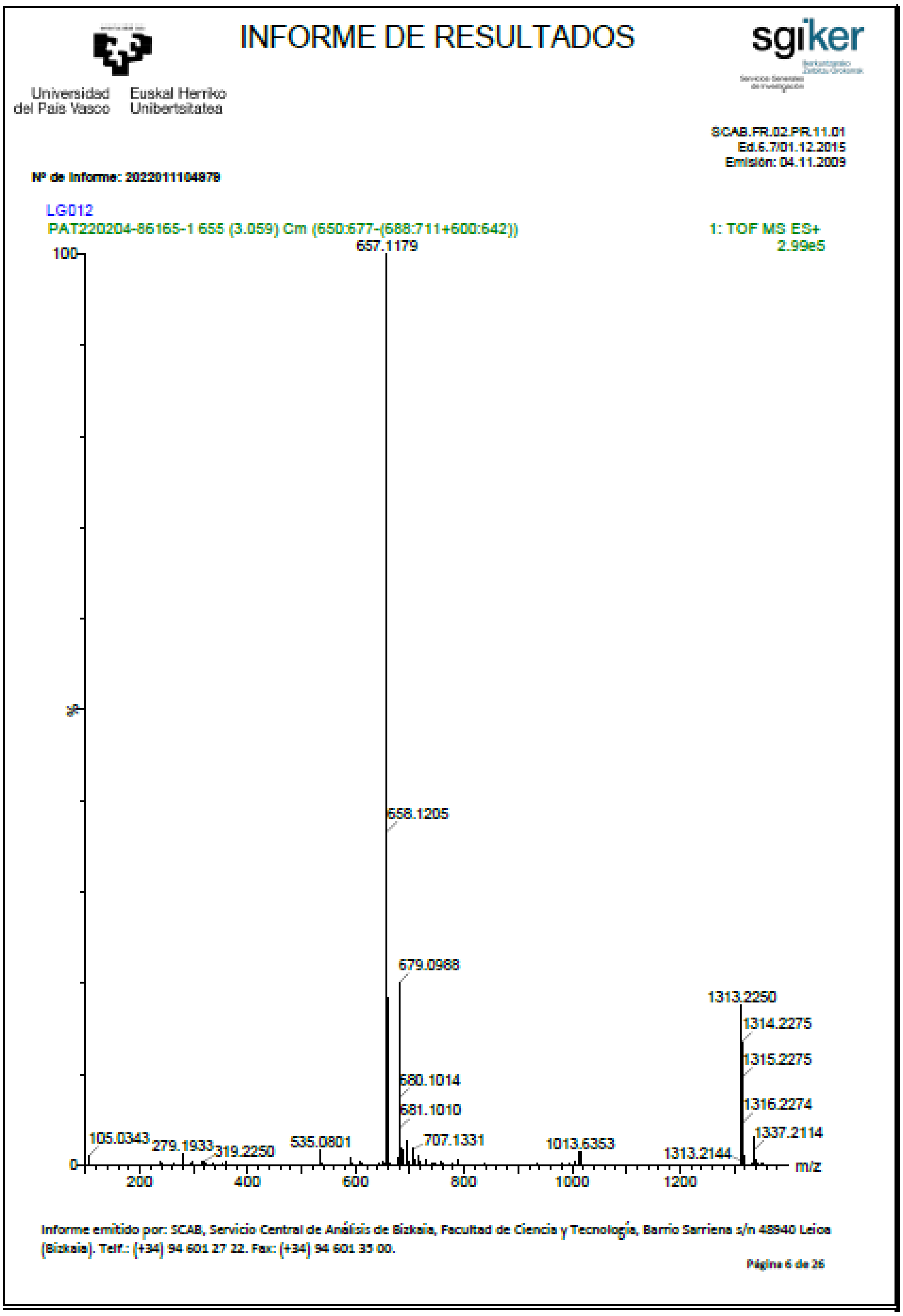

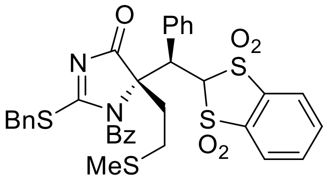

15fa

HRMS (ESI) m/z: [M + H] $^+$ calcd. for $C_{34}H_{31}N_2O_6S_4$ 691.1065; found 691.1061.

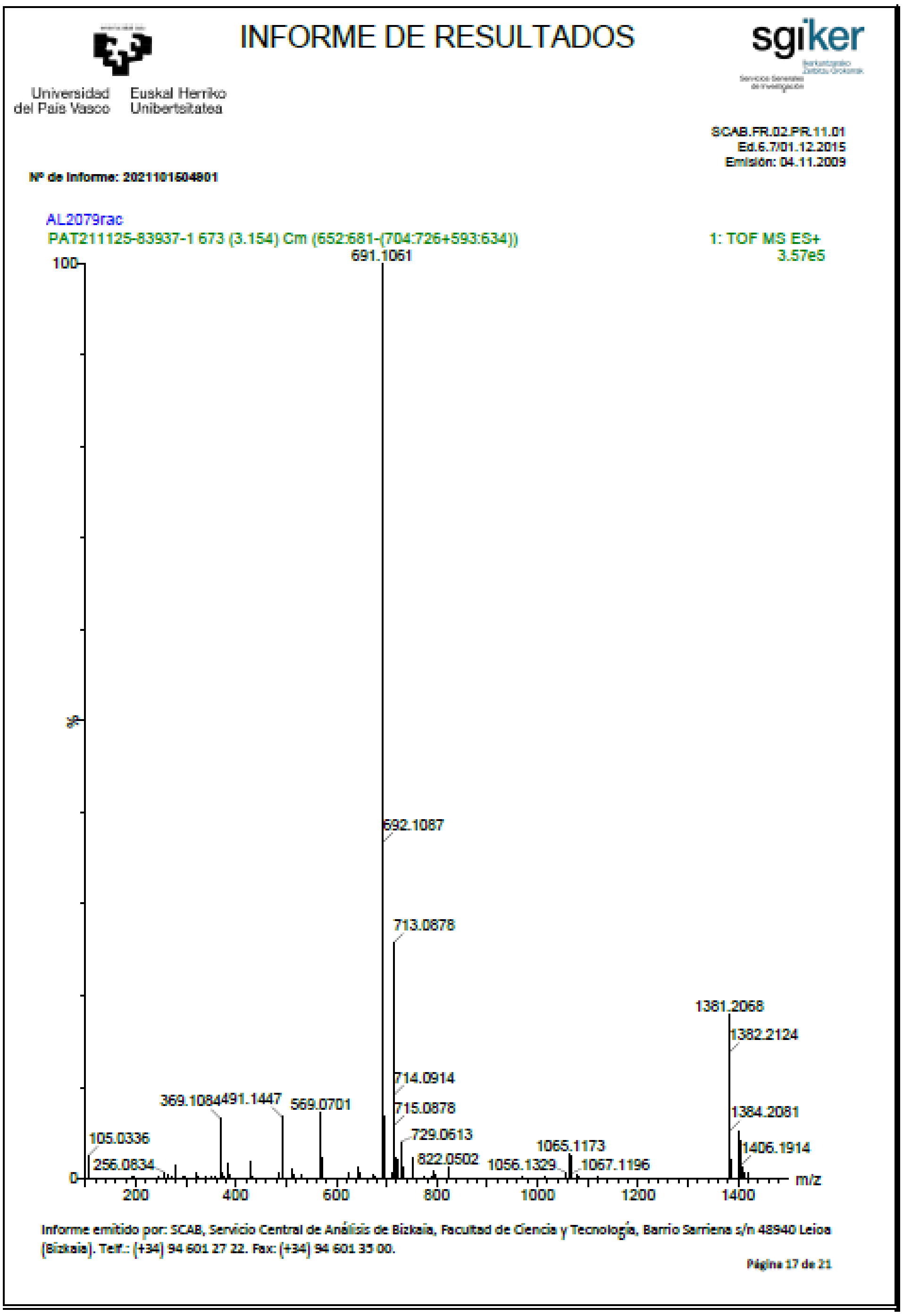

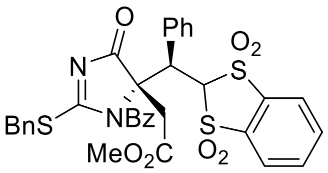

15ga

HRMS (ESI) m/z: [M + H]$^{+}$ calcd. for $C_{34}H_{29}N_2O_8S_3$ 689.1086; found 689.1092.

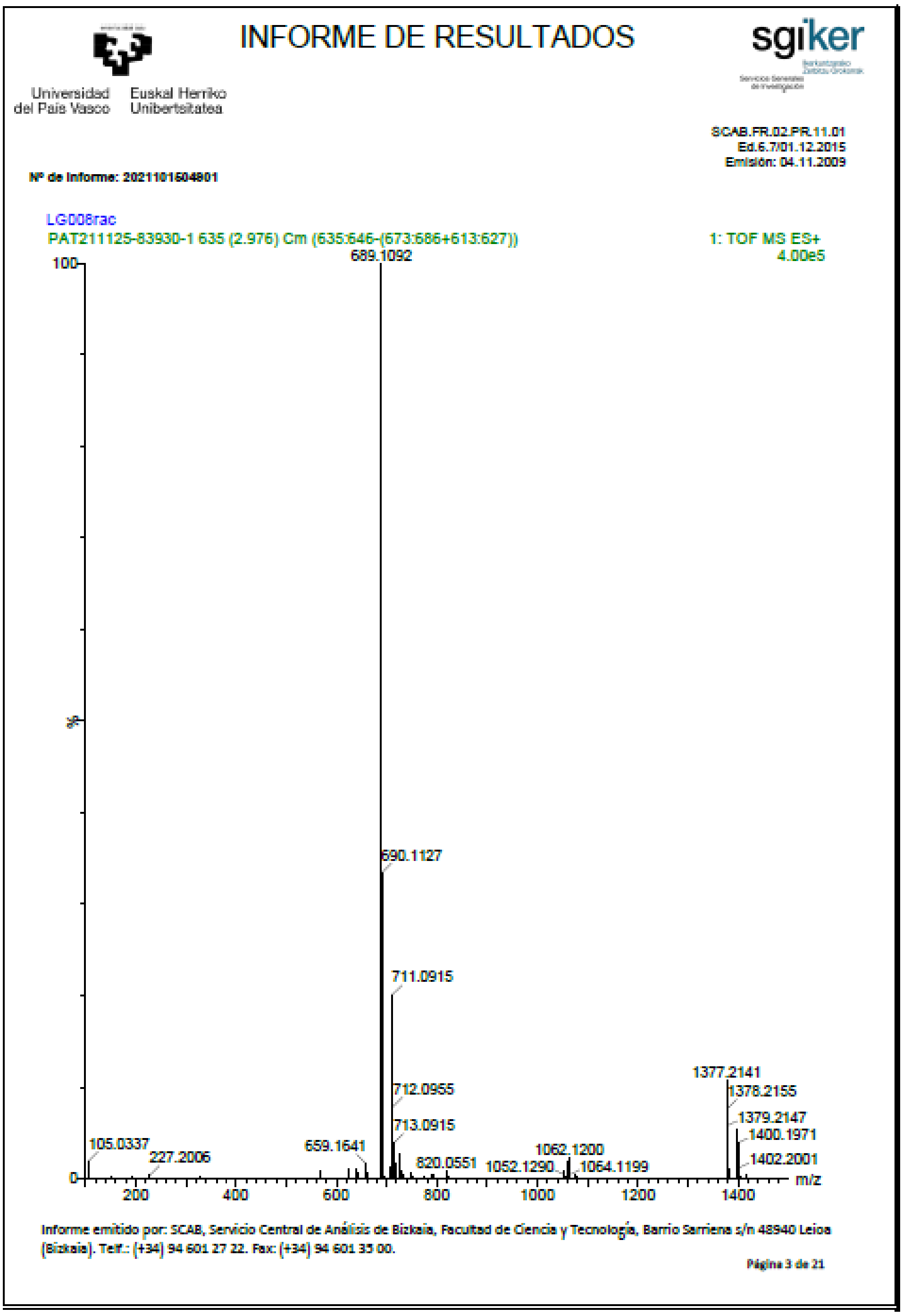

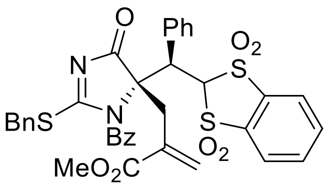

15ha

HRMS (ESI) m/z: [M + H] $^{+}$ calcd. for $C_{36}H_{31}N_2O_8S_3$ 715.1237; found 715.1244.

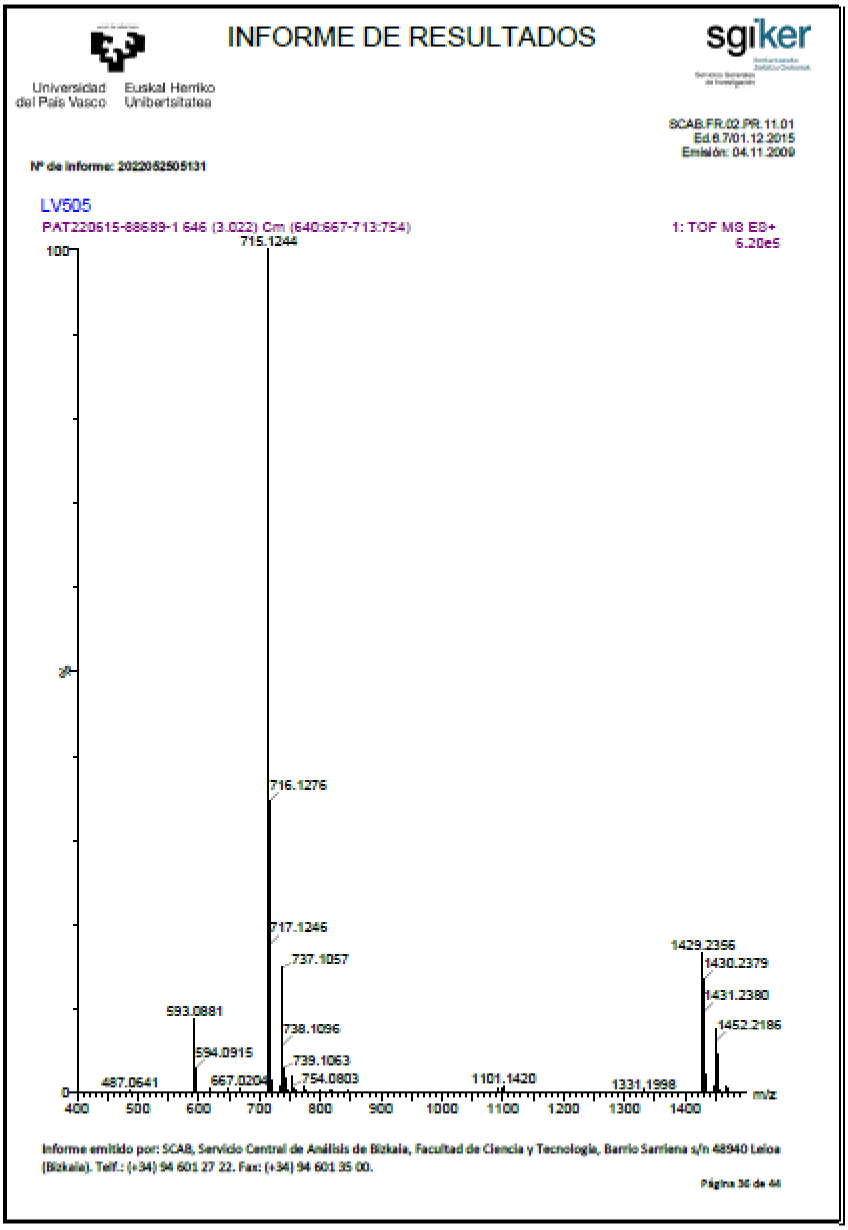

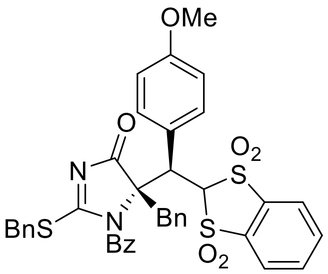

15ab

HRMS (ESI) m/z: [M + H]$^{+}$ calcd. for $C_{39}H_{33}N_{2}O_{7}S_{3}$ 737.1444; found 737.1441.

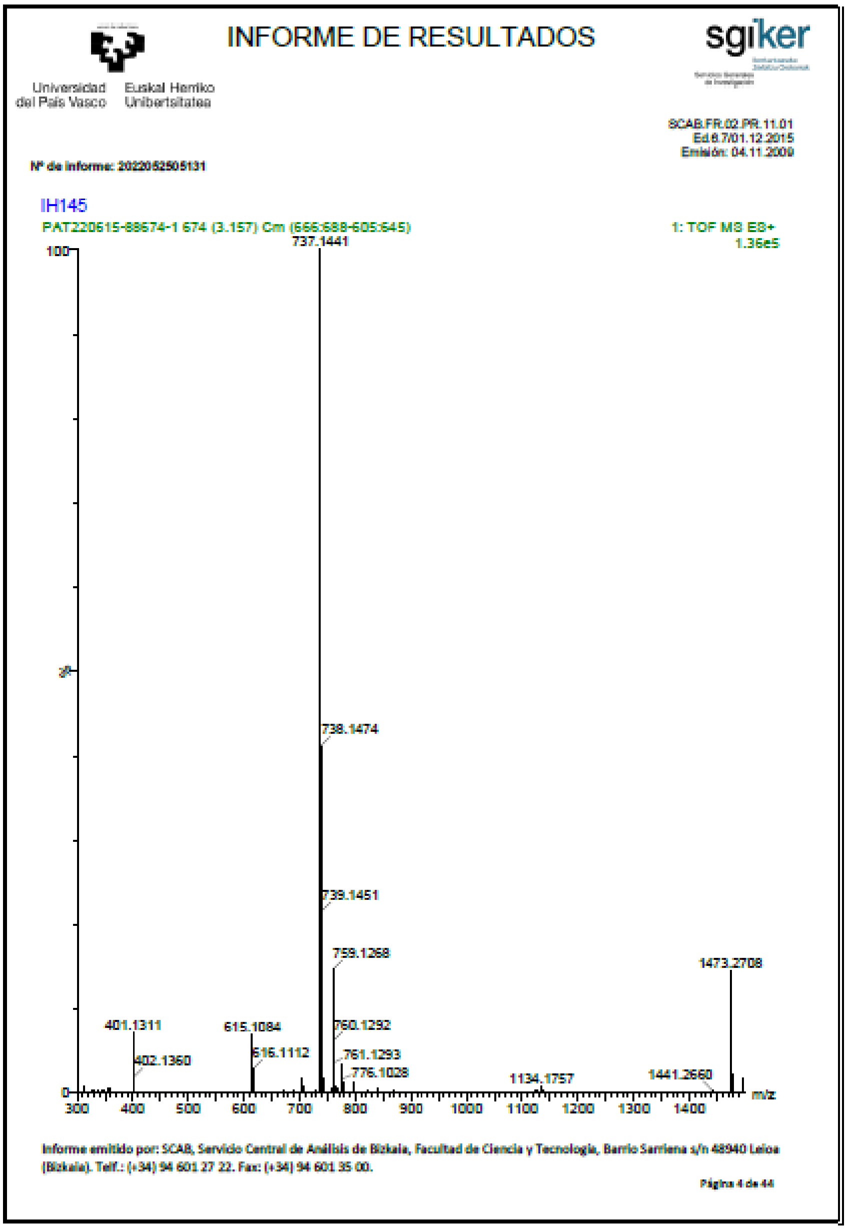

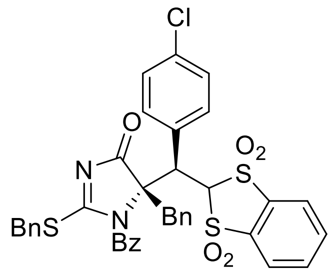

15ac

HRMS (ESI) m/z: [M + H]$^{+}$ calcd. for $C_{38}H_{30}ClN_2O_6S_3$ 741.0955; found 741.0955.

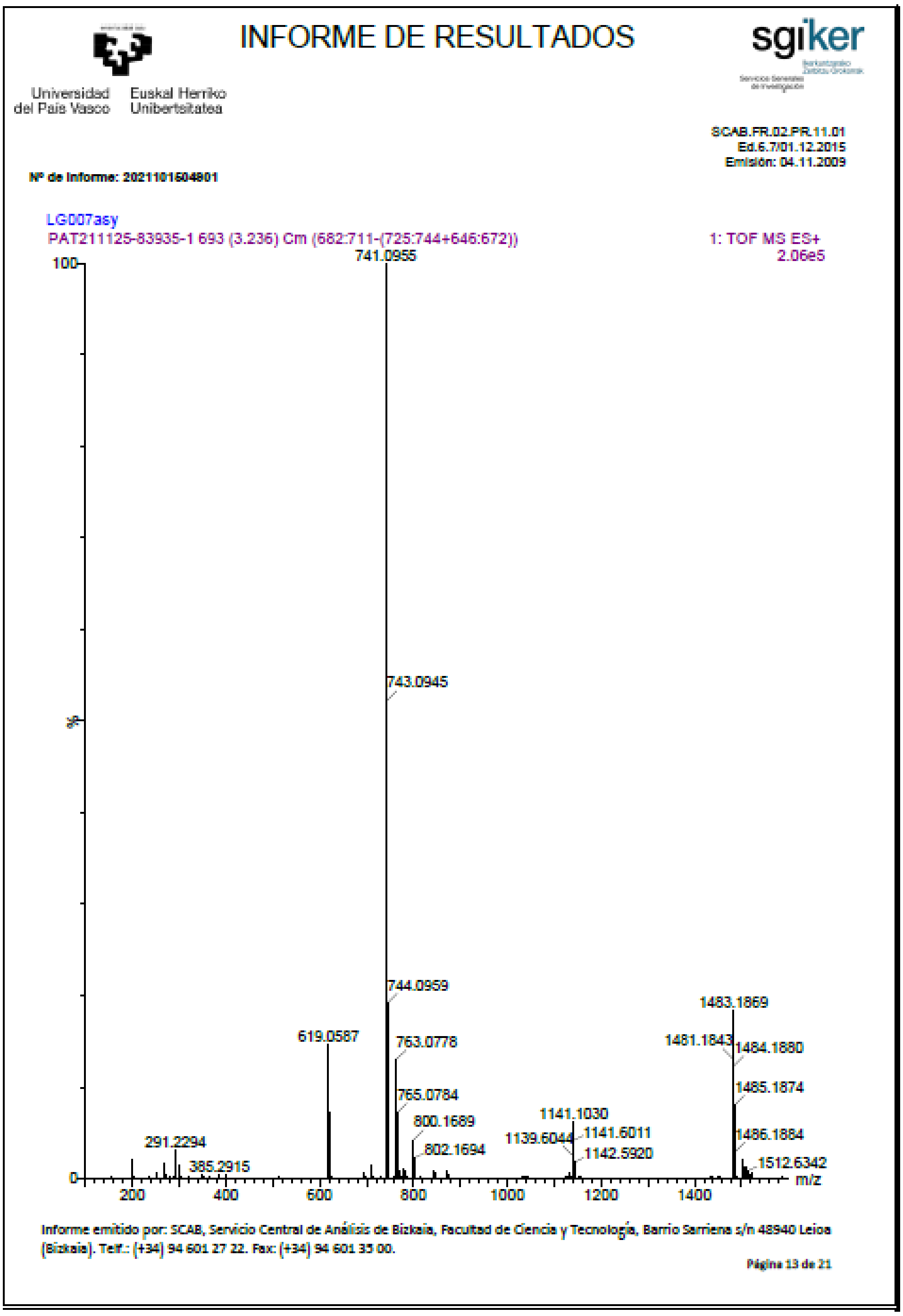

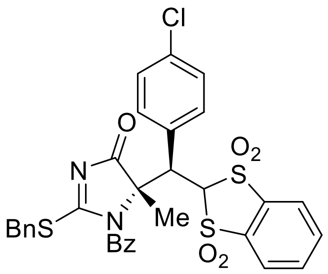

15bc

HRMS (ESI) m/z: [M + H] $^{+}$ calcd. for $C_{32}H_{26}ClN_2O_6S_3$ 665.0636; found 665.0634.

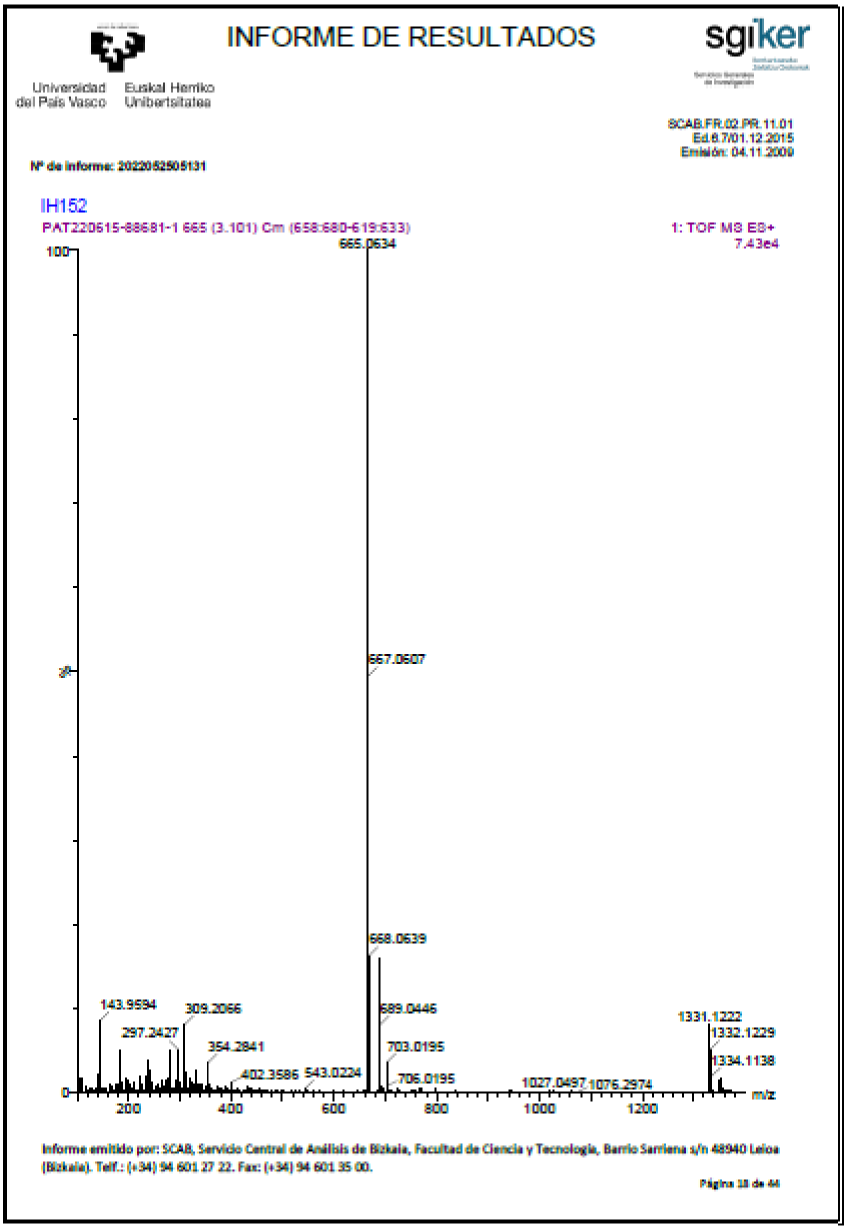

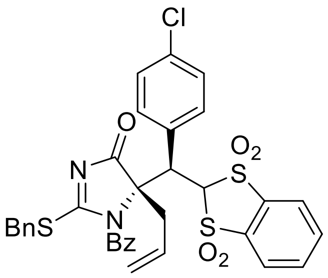

15ec

HRMS (ESI) m/z: [M + H]$^+$ calcd. for $C_{34}H_{28}ClN_2O_6S_3$ 691.0793; found 691.0788.

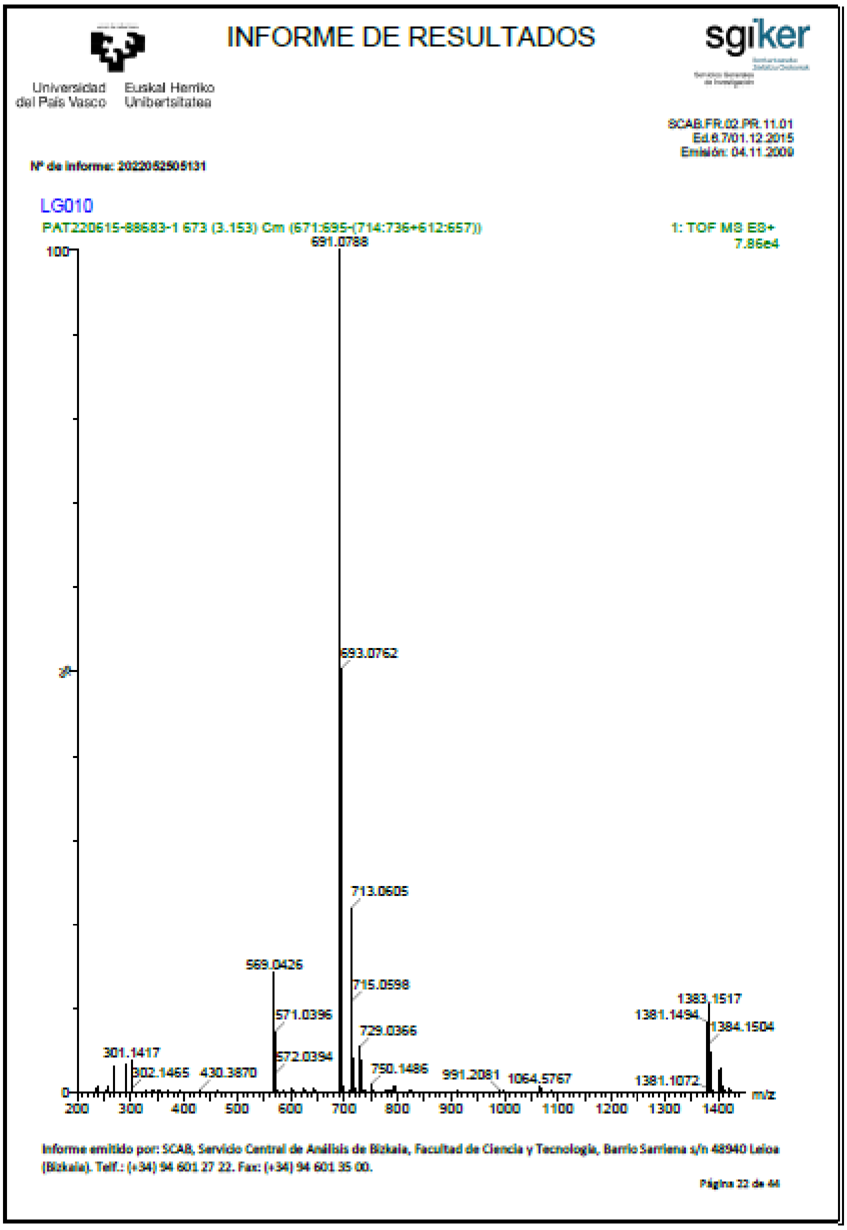

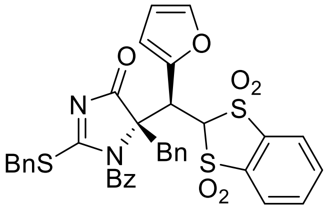

15ad

HRMS (ESI) m/z: [M + H]$^+$ calcd. for $C_{36}H_{29}N_2O_7S_3$ 697.1131; found 697.1138.

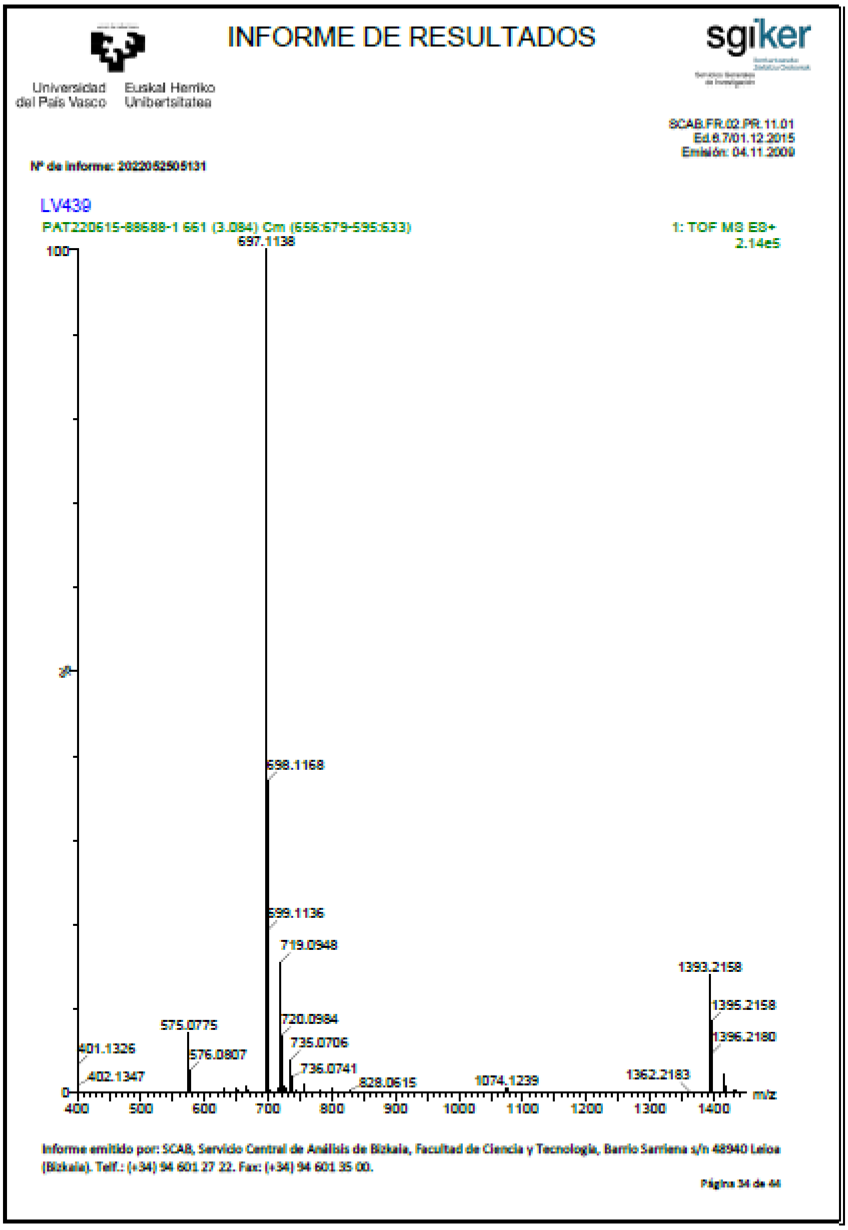

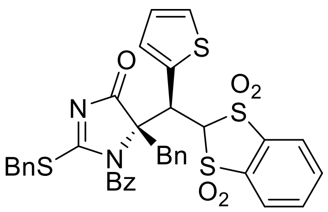

15ae

HRMS (ESI) m/z: $[M + H]^+$ calcd. for $C_{36}H_{29}N_2O_6S_4$ 713.0903; found 713.0905.

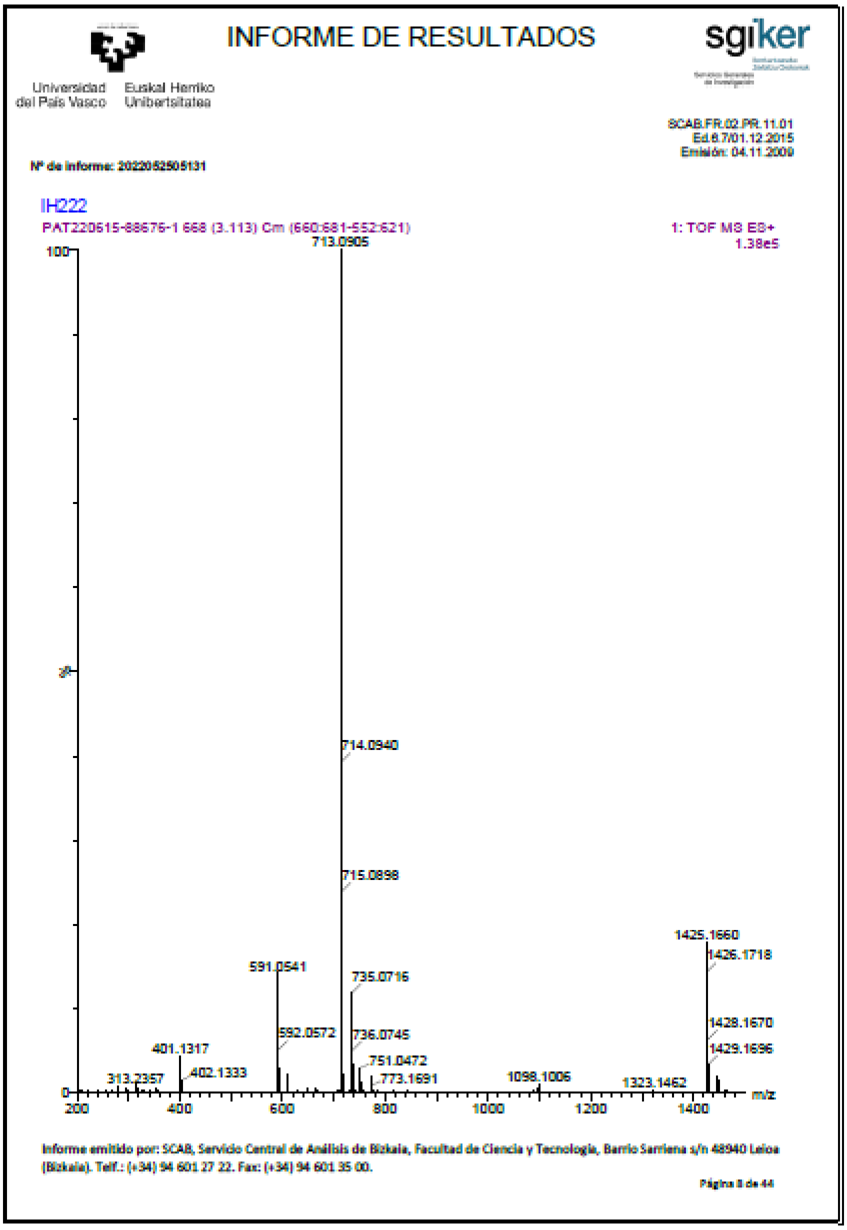

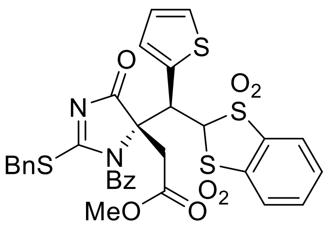

15ge

HRMS (ESI) m/z: [M + H] $^{+}$ calcd. for $C_{32}H_{27}N_2O_8S_4$ 695.0650; found 695.0647.

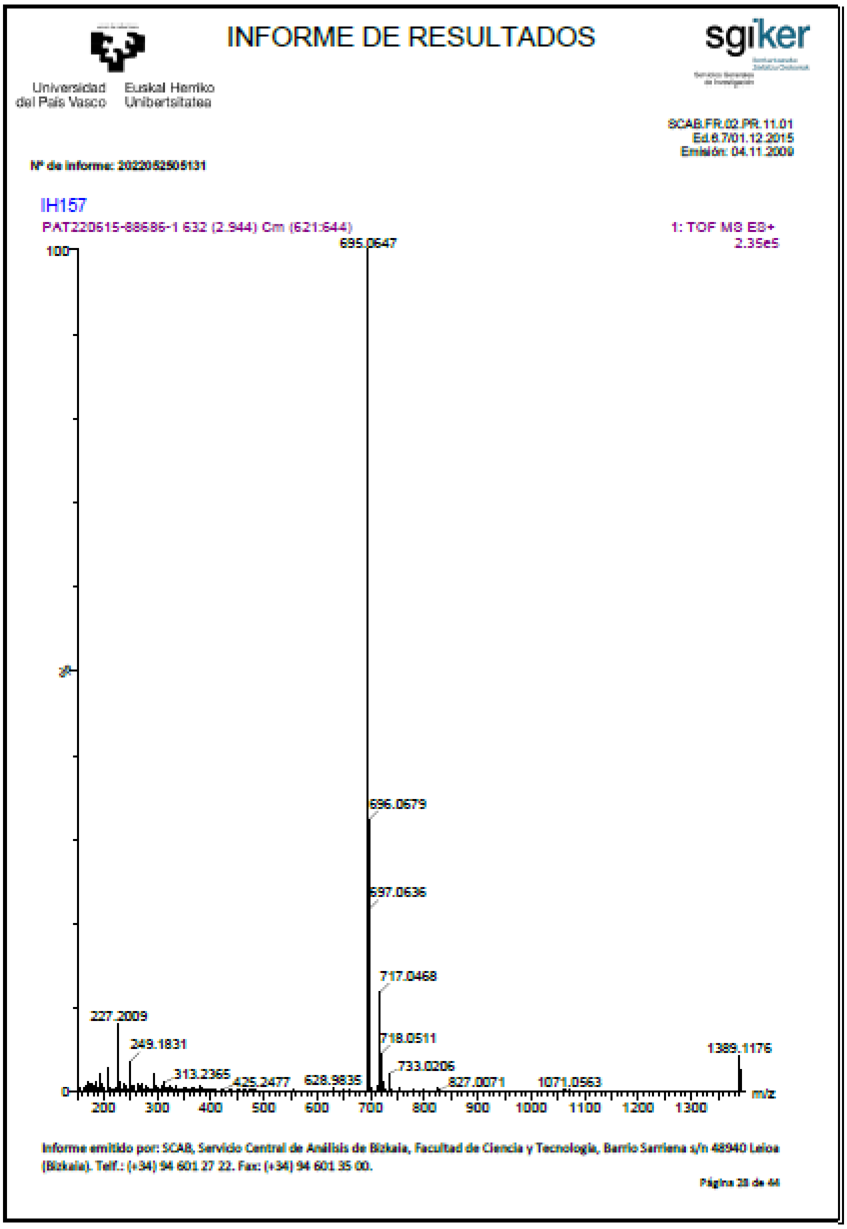

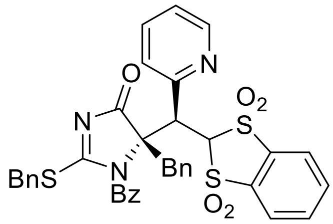

15af

HRMS (ESI) m/z: [M + H]$^+$ calcd. for $C_{37}H_{30}N_3O_6S_3$ 708.1291; found 708.1299.

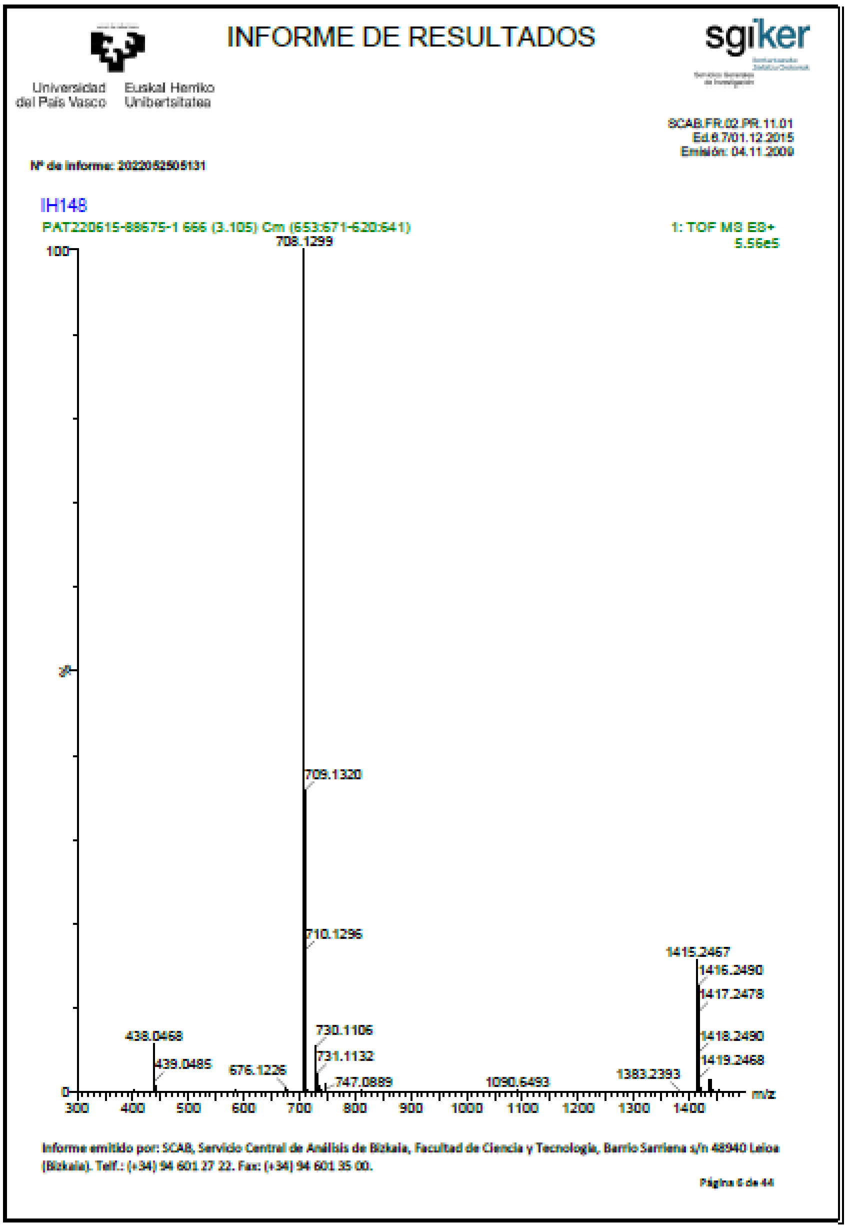

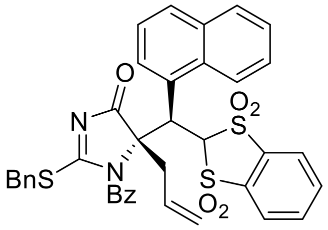

15eg

HRMS (ESI) m/z: [M + H]+ Calcd for $C_{38}H_{31}N_2O_6S_3$ 707.1339; Found 707.1342.

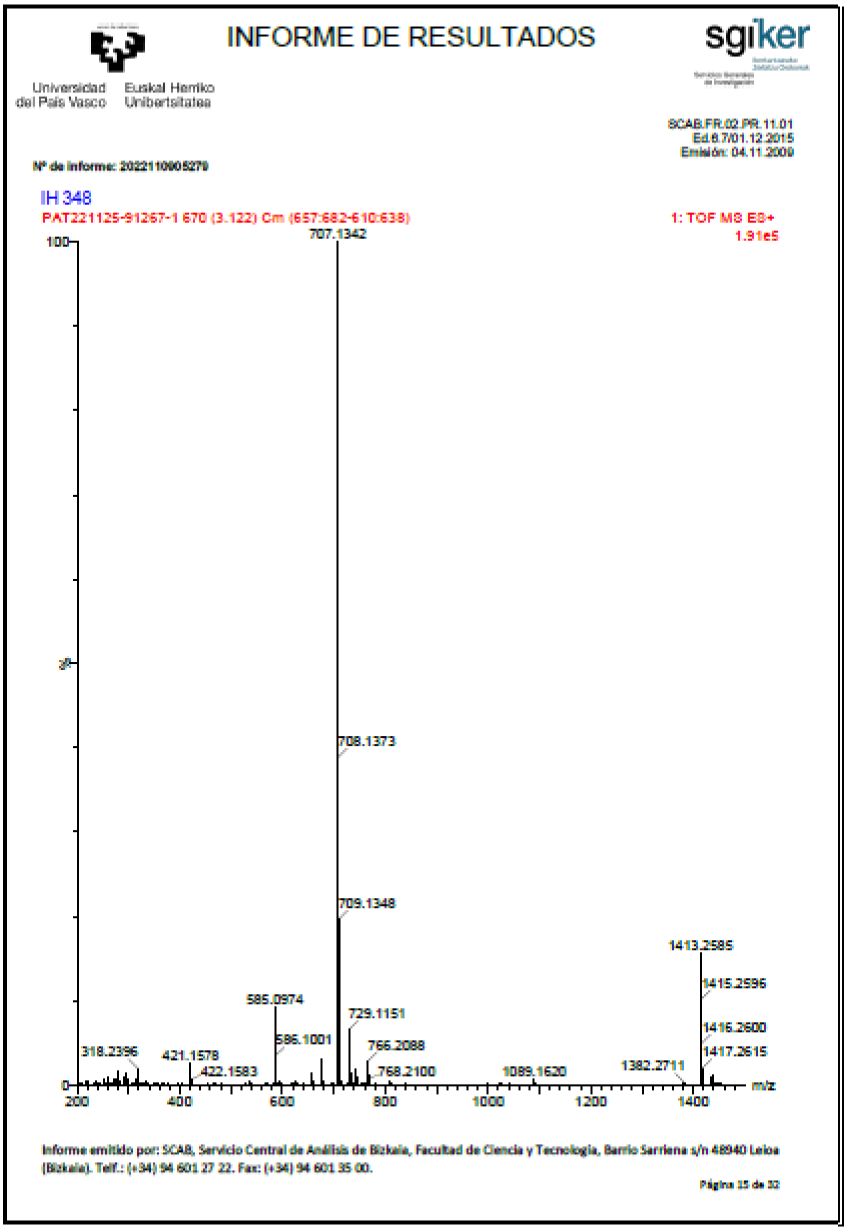

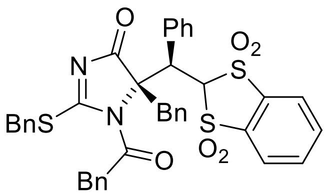

18

HRMS (ESI) m/z: [M + H] $^{+}$ calcd. for $C_{39}H_{33}N_2O_6S_3$ 721.1495; found 721.1486.

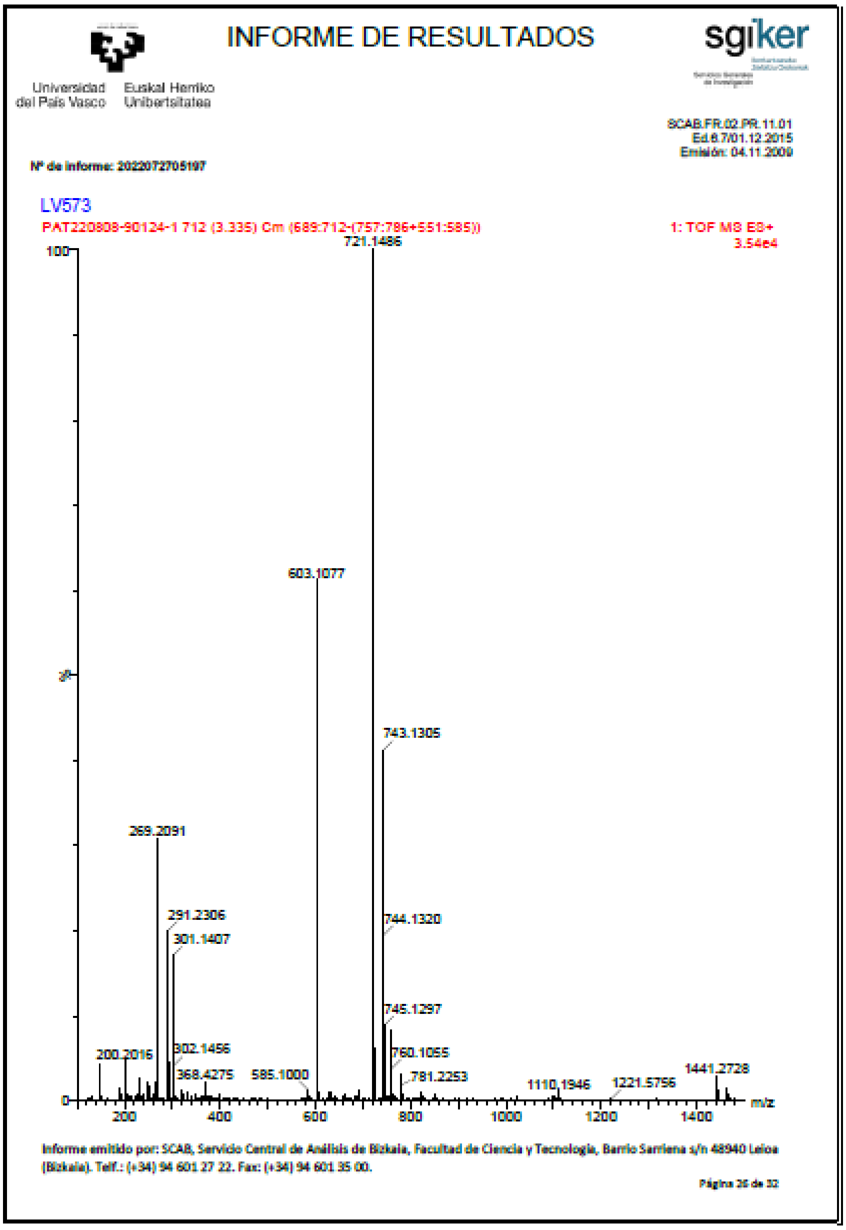

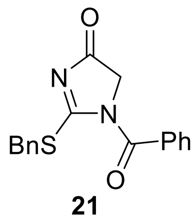

21

HRMS (ESI) m/z: [M + H] $^+$ calcd. for $C_{17}H_{15}N_2O_2S$ 311.0849; found 311.0857.

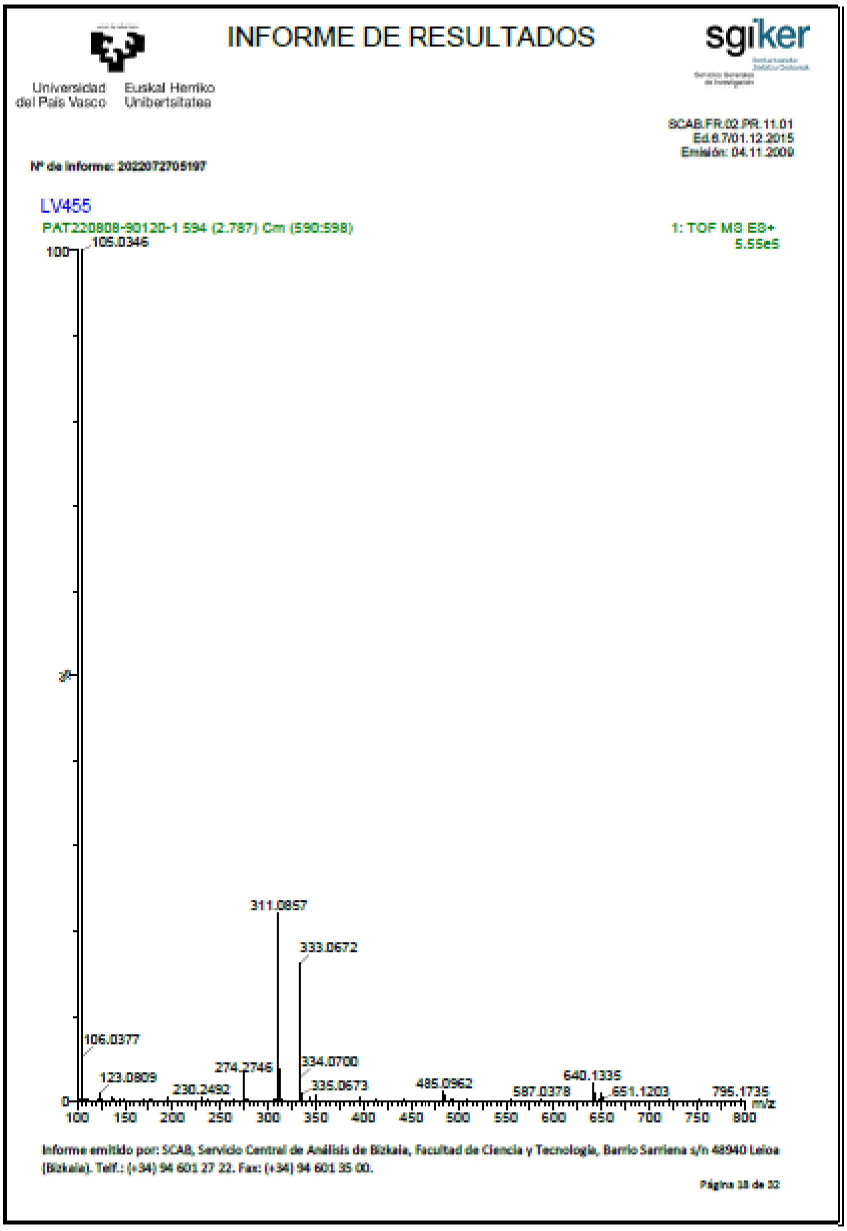

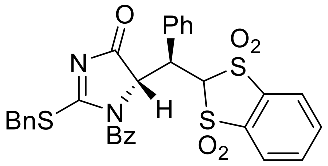

22a

HRMS (ESI) m/z: [M + H]$^+$ calcd. for $C_{31}H_{25}N_2O_6S_3$ 617.0875; found 617.0878.

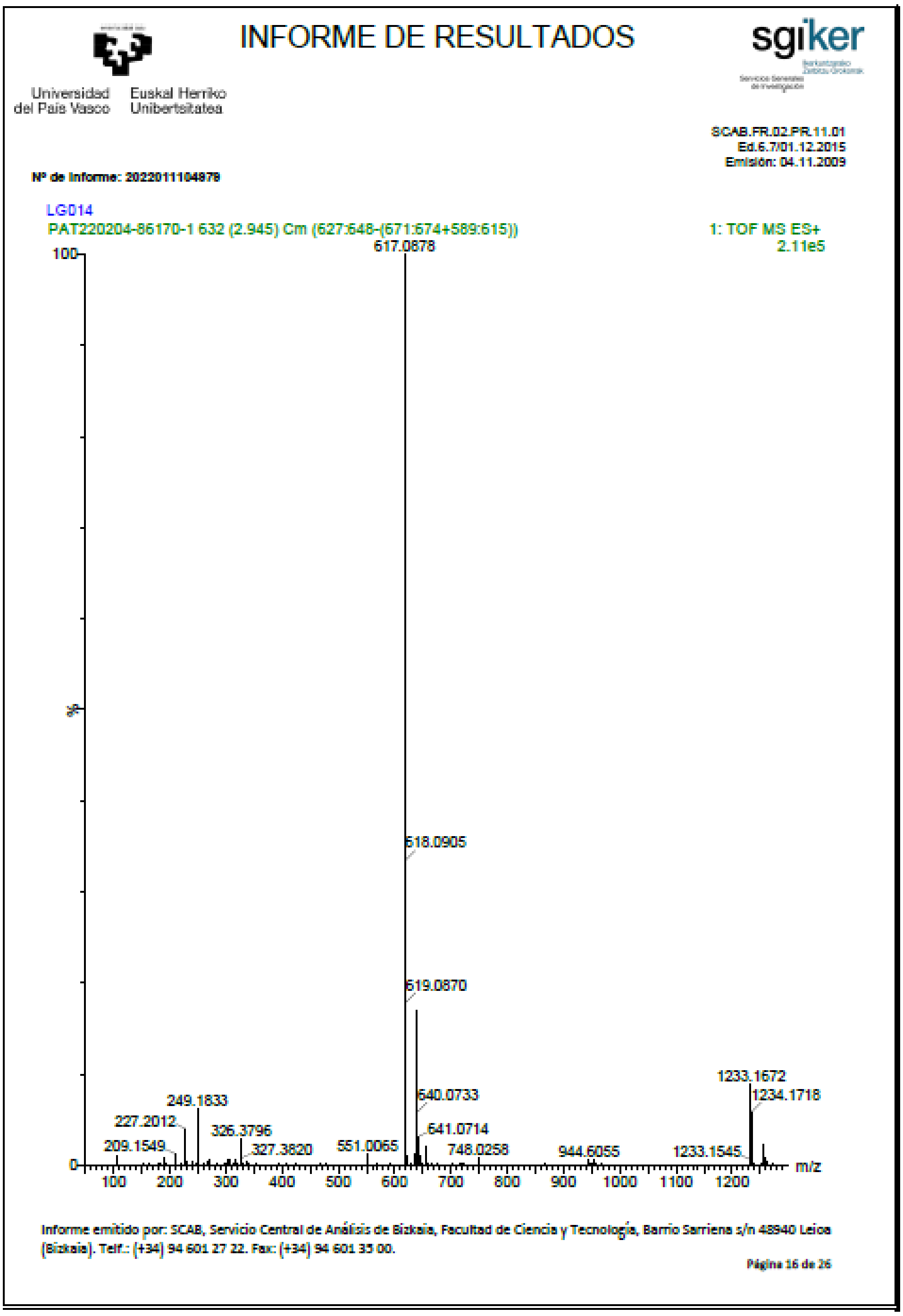

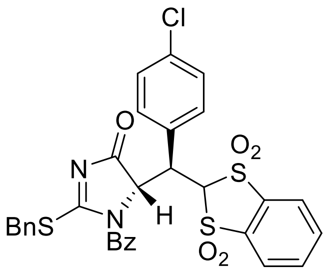

22b

HRMS (ESI) m/z: $[M + H]^+$ calcd. for $C_{31}H_{24}ClN_2O_6S_3$ 651.0480; found 651.0488.

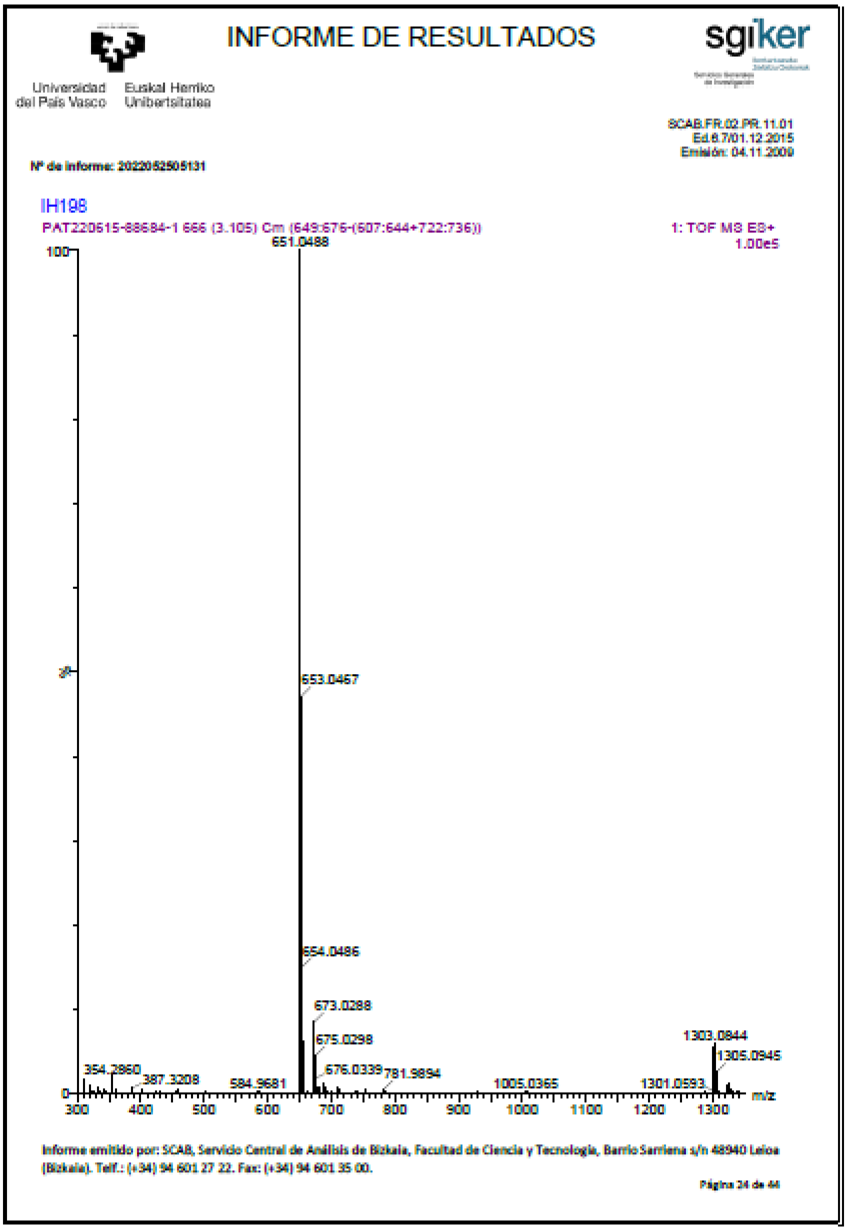

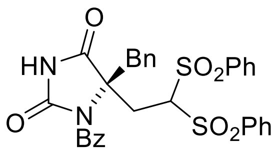

23

HRMS (ESI) m/z: [M + H]$^+$ calcd. for $C_{31}H_{27}N_2O_7S_2$ 603.1254; found 603.1252.

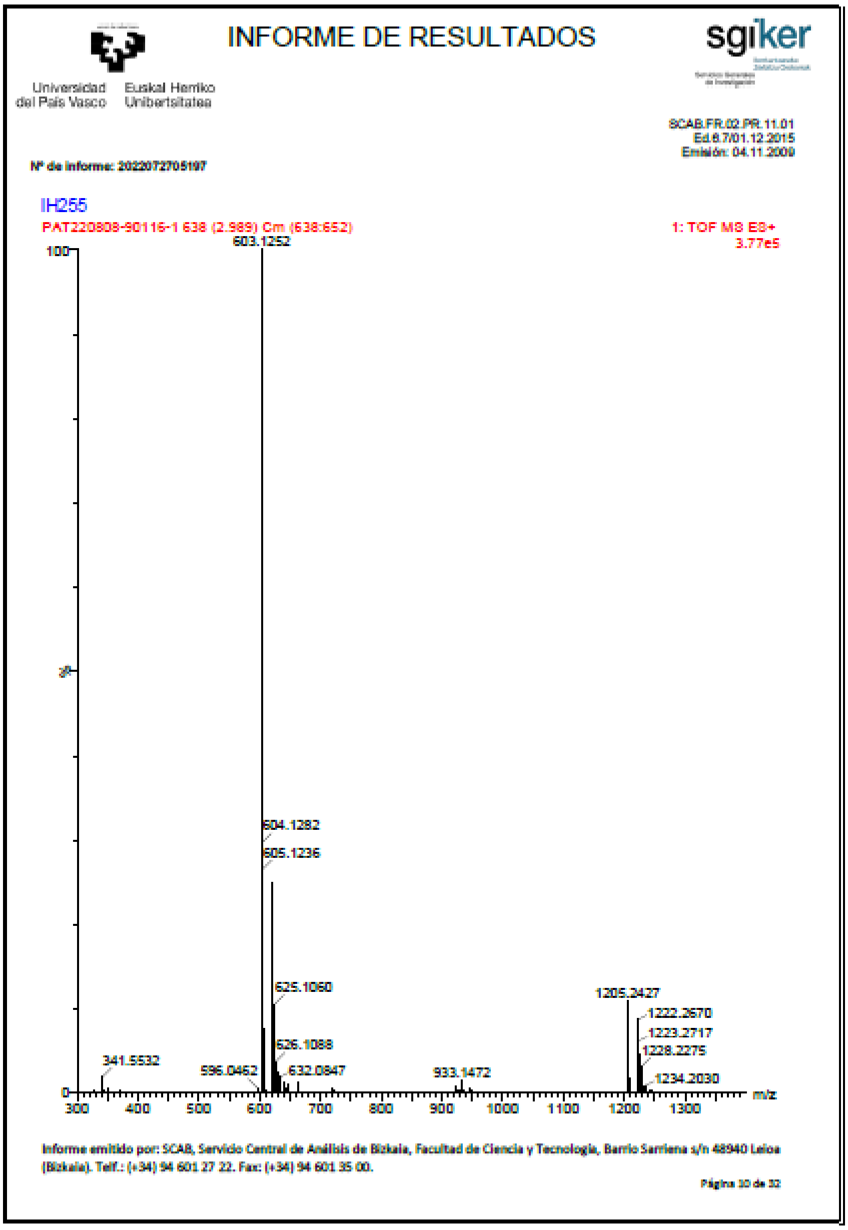

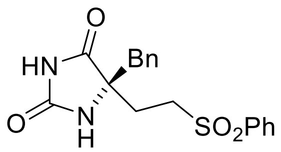

24

HRMS (ESI) m/z: [M + H]$^+$ calcd. for $C_{18}H_{19}N_2O_4S$ 359.1060; found 359.1066.

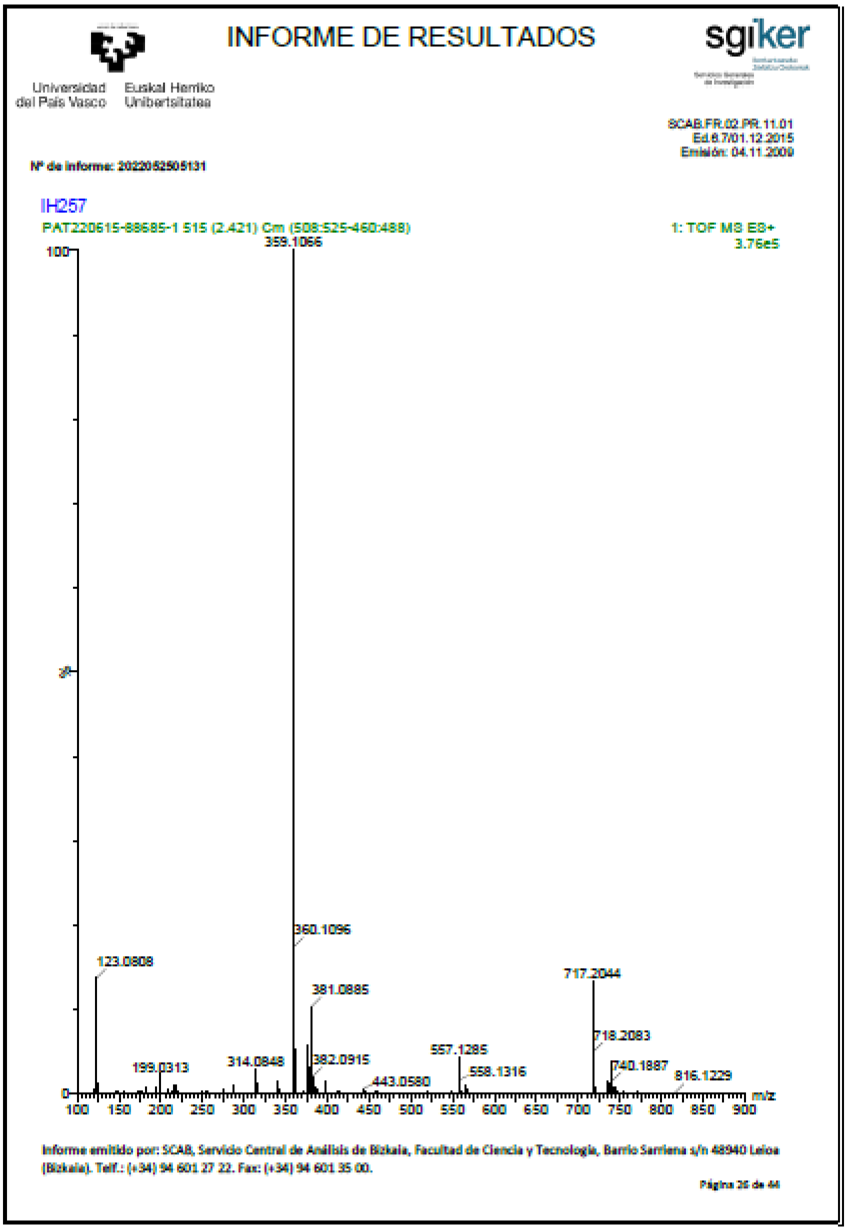

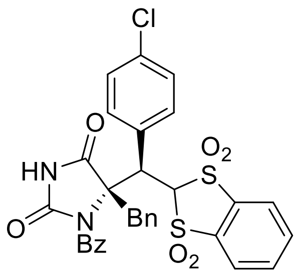

25b

HRMS (ESI) m/z: [M + H]$^+$ calcd. for $C_{31}H_{24}ClN_2O_7S_2$ 635.0708; found 635.0701.

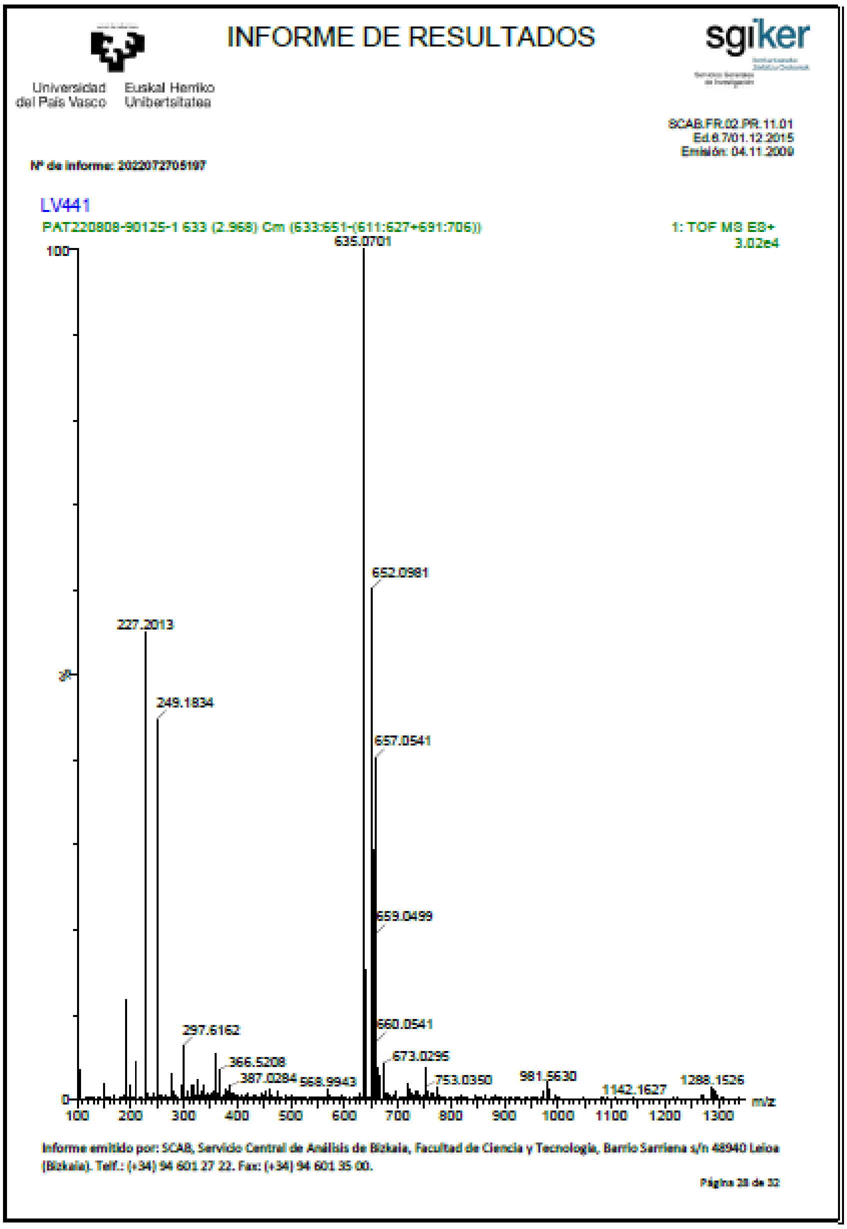

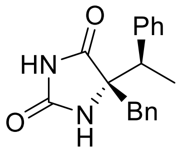

26

HRMS (ESI) m/z: [M + H] $^{+}$ calcd. for $C_{18}H_{19}N_2O_2$ 295.1441; found 295.1448.

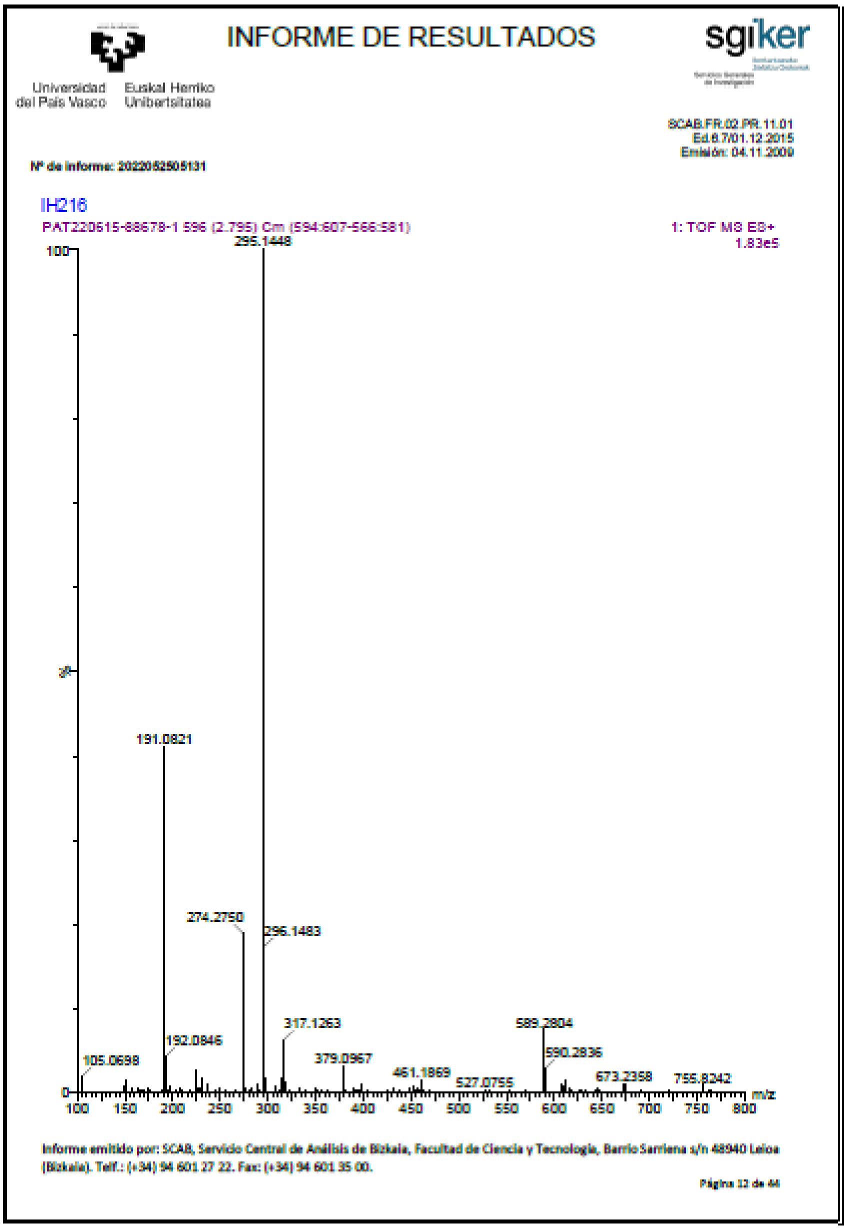

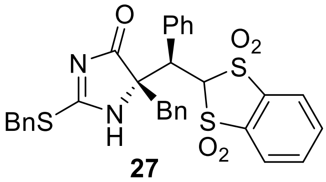

27

HRMS (ESI) m/z: $[M + H]^+$ calcd. for $C_{31}H_{27}N_2O_5S_3$ 603.1077; found 603.1088.

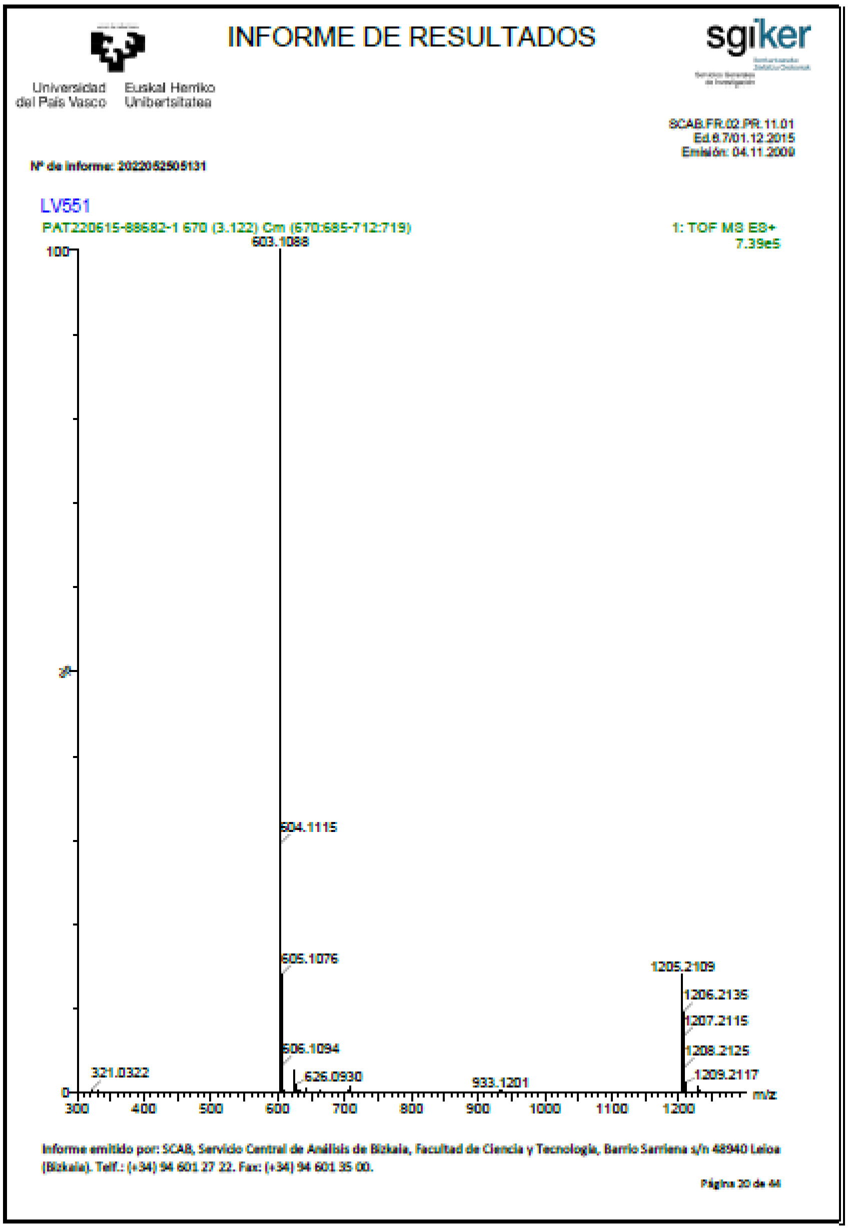

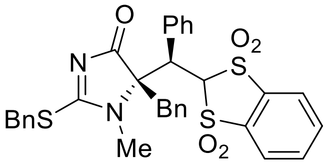

28

HRMS (ESI) m/z: [M + H]$^+$ calcd. for $C_{32}H_{29}N_2O_5S_3$ 617.1233; found 617.1243.

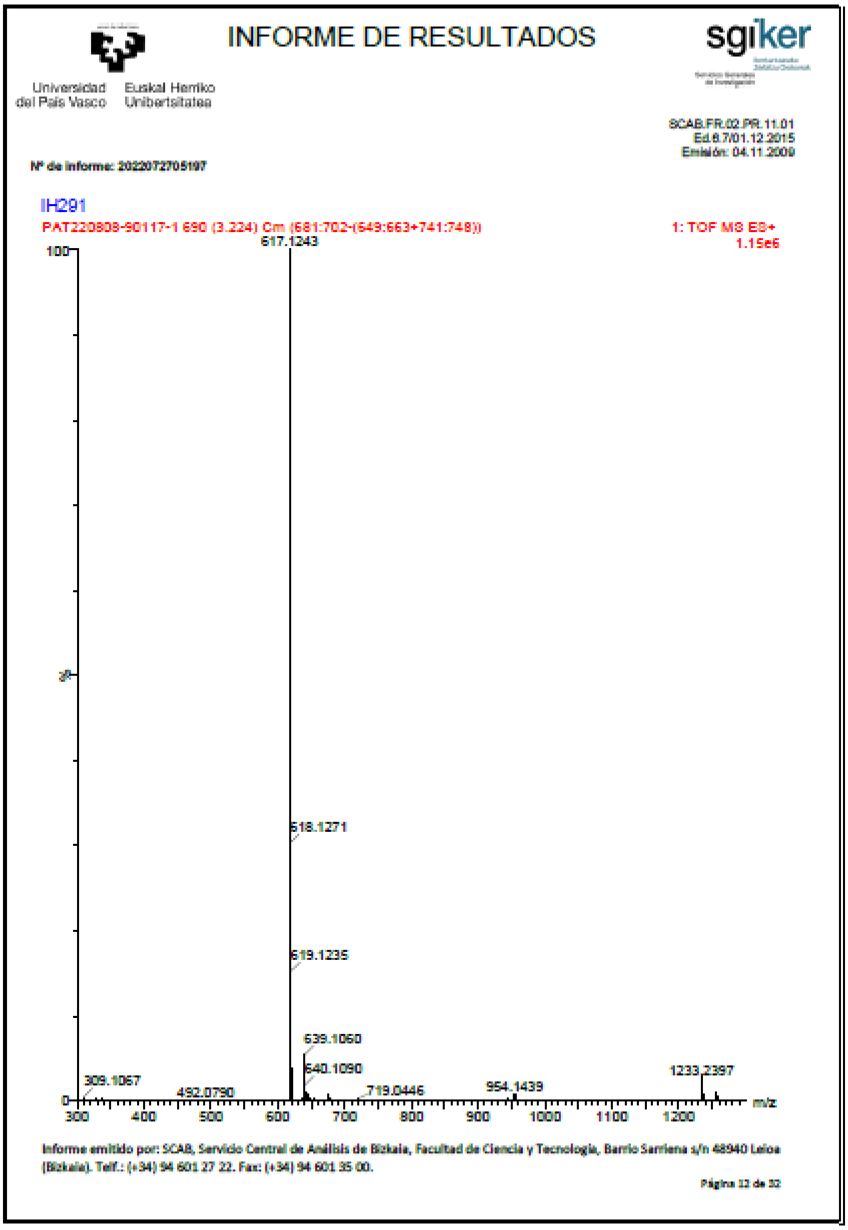

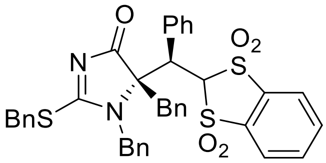

29

HRMS (ESI) m/z: $[M + H]^+$ calcd. for $C_{38}H_{33}N_2O_5S_3$ 693.1546; found 693.1543.

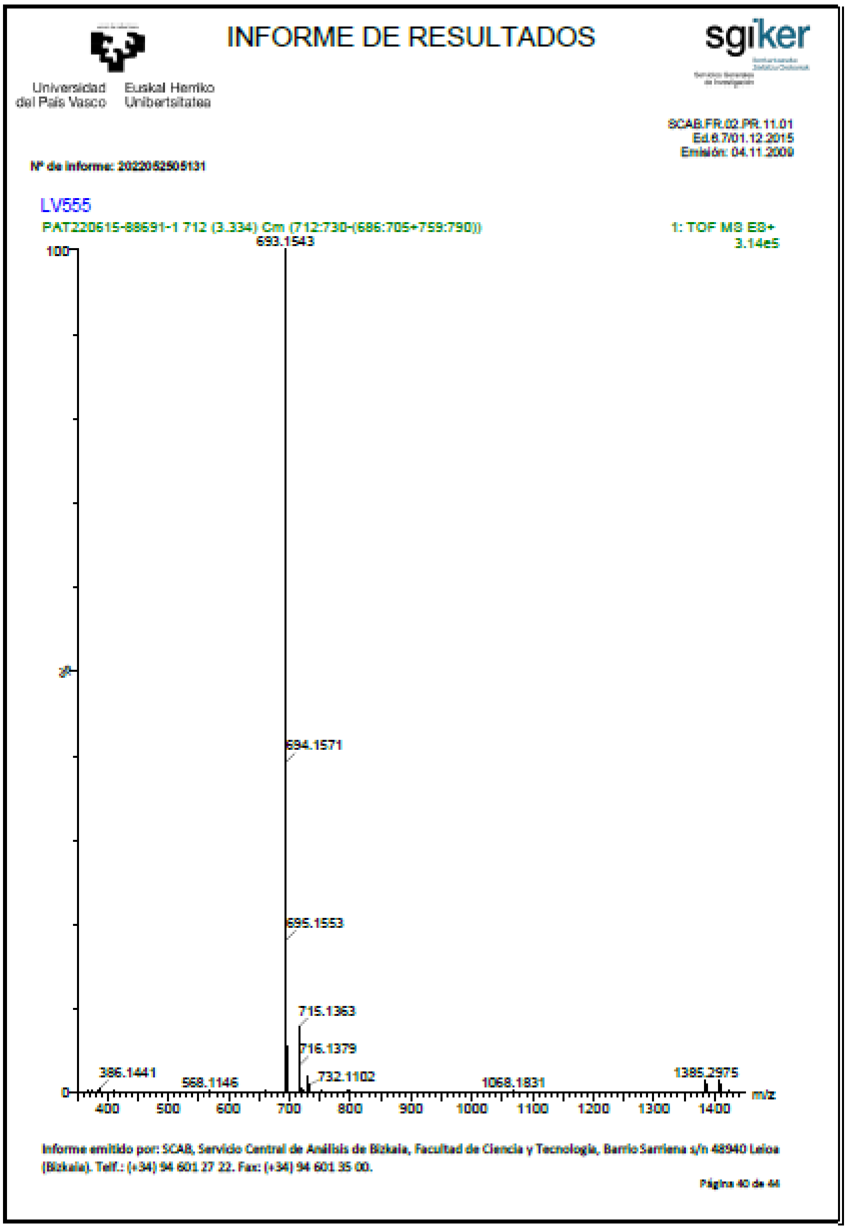

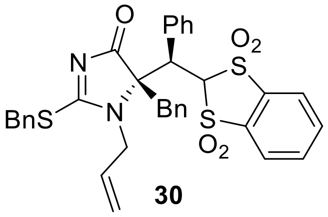

30

HRMS (ESI) m/z: [M + H]$^+$ calcd. for $C_{34}H_{31}N_2O_5S_3$ 643.1390; found 643.1383.

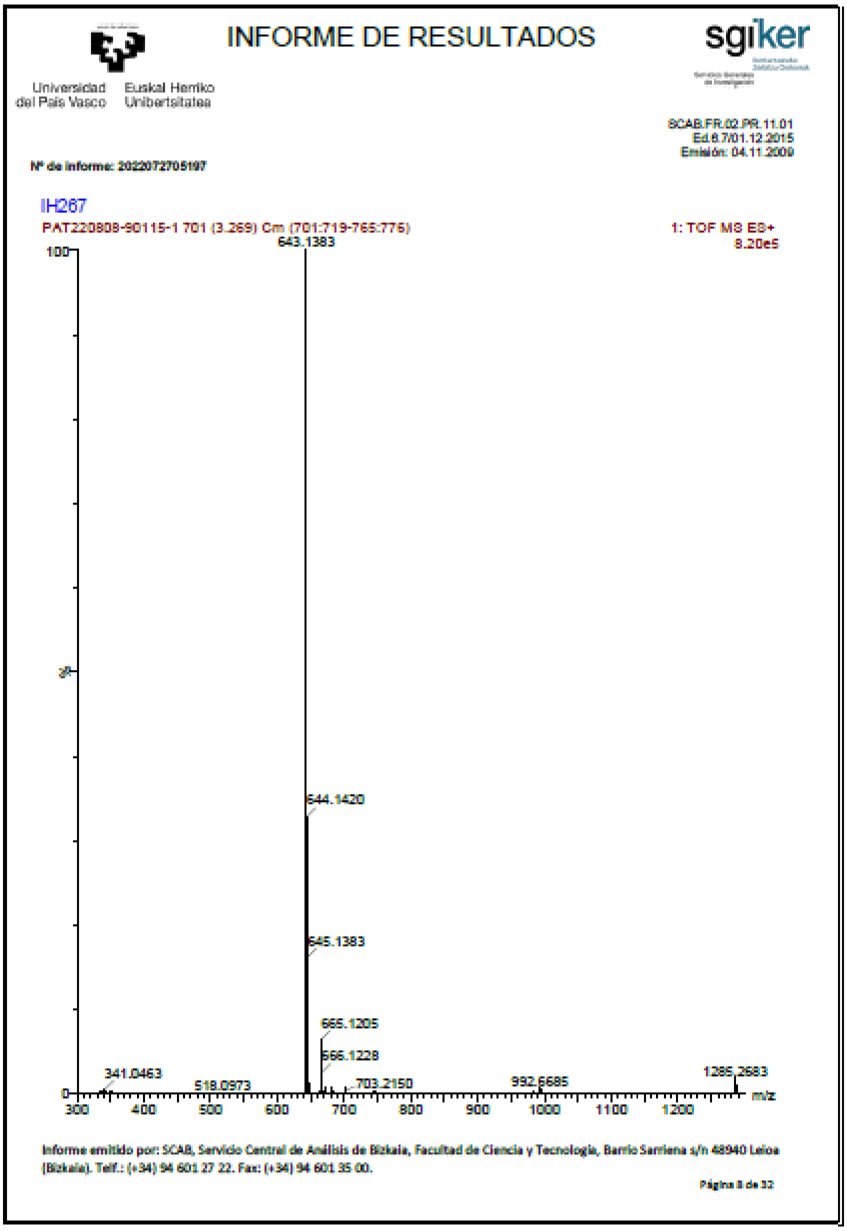

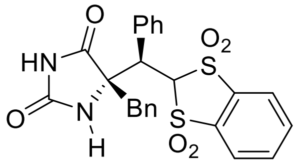

32a

HRMS (ESI) m/z: $[M + H]^+$ calcd. for $C_{24}H_{21}N_2O_6S_2$ 497.0836; found 497.0824.

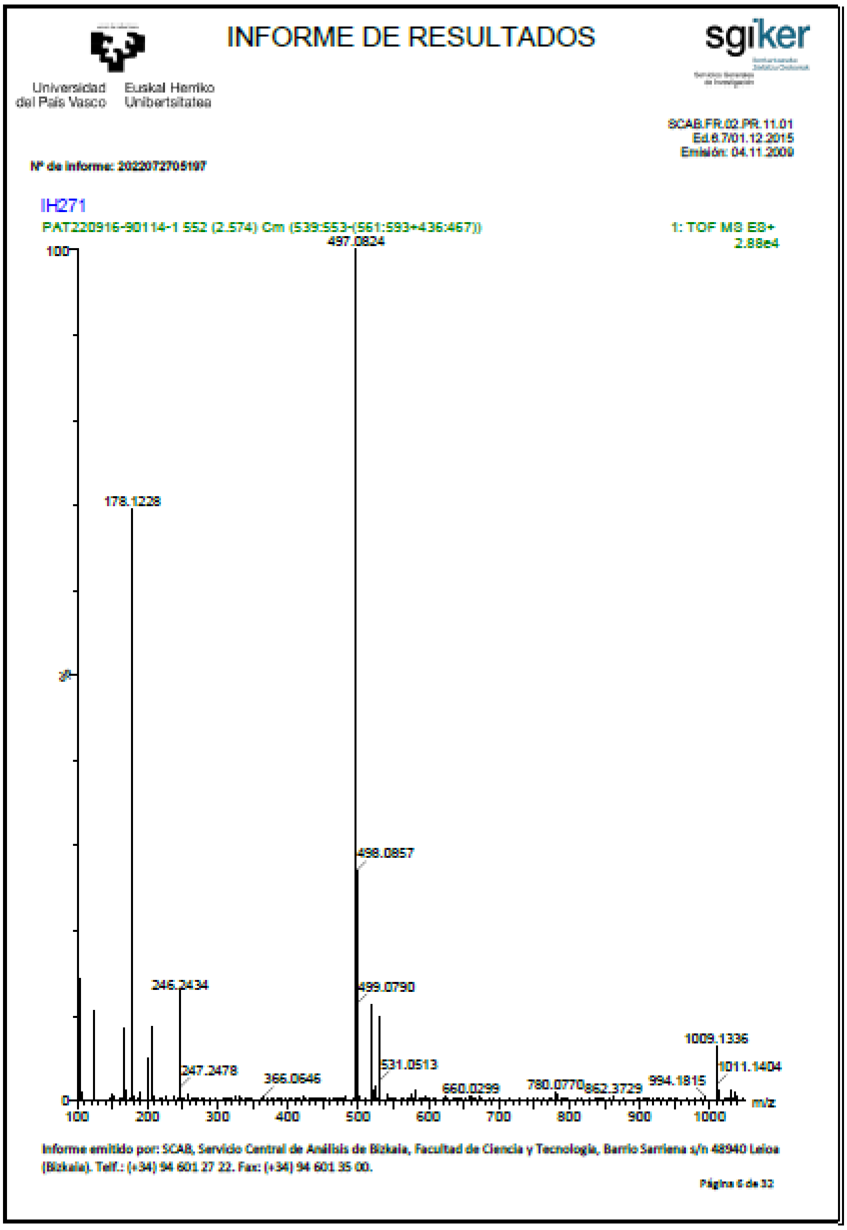

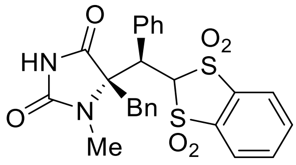

32b

HRMS (ESI) m/z: [M + H] $^{+}$ calcd. for $C_{25}H_{23}N_2O_6S_2$ 511.0992; found 511.0992.

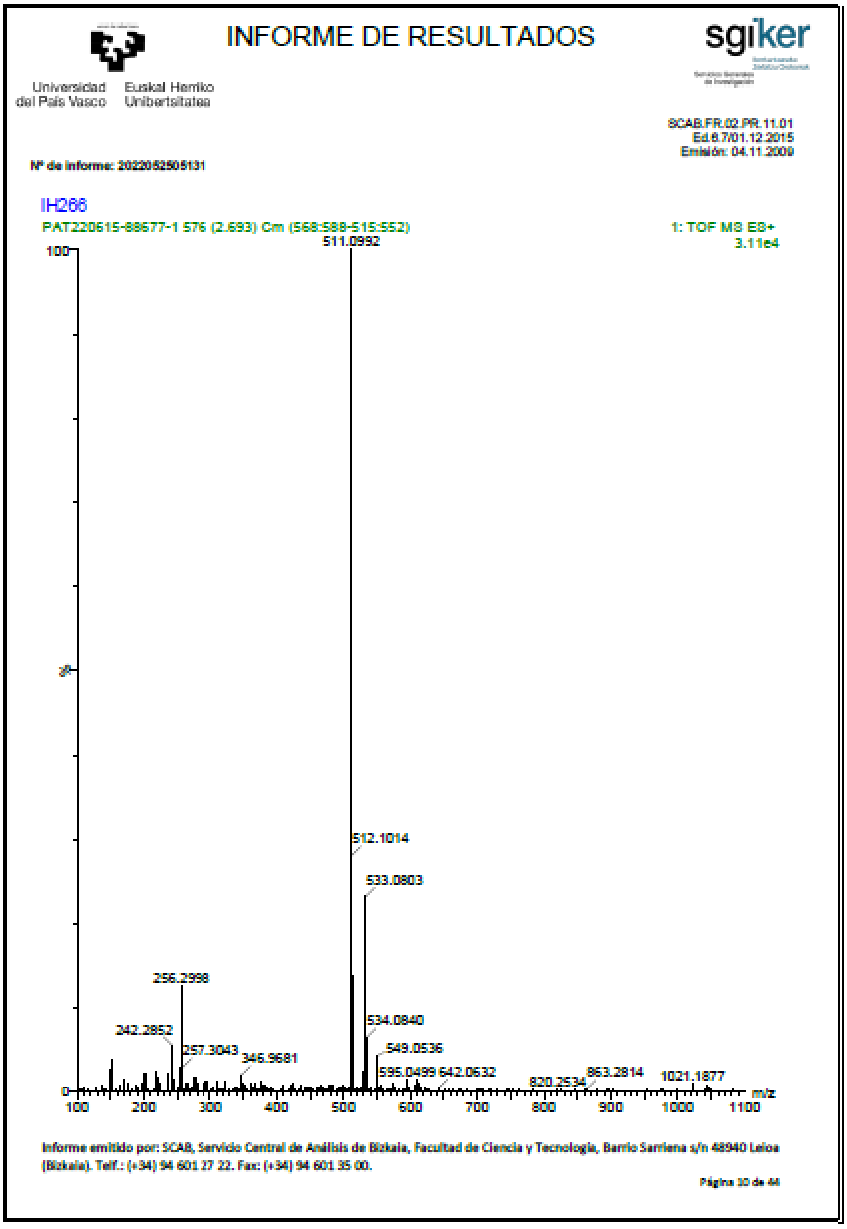

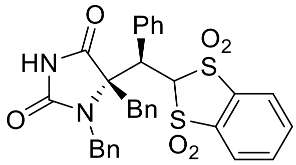

32c

HRMS (ESI) m/z: [M + H] $^+$ calcd. for $C_{31}H_{27}N_2O_6S_2$ 587.1305; found 587.1298.

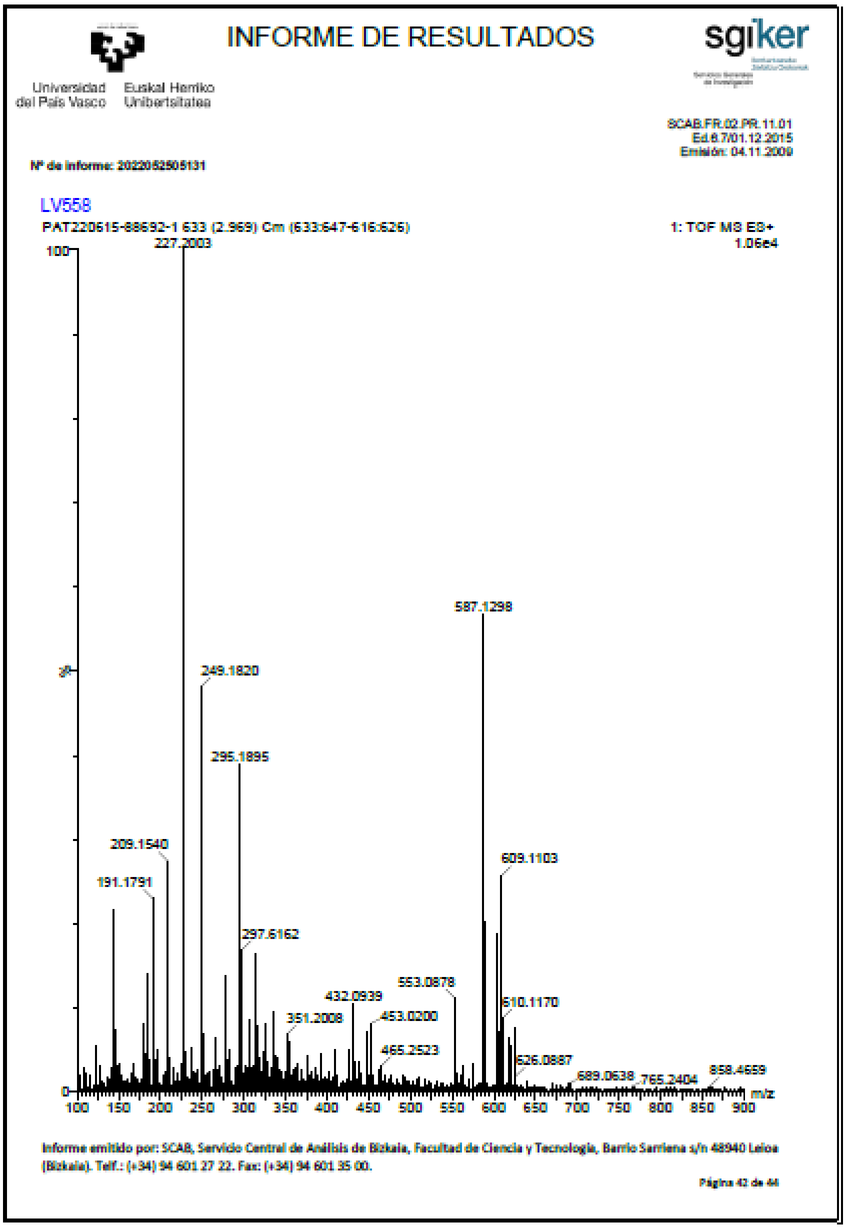

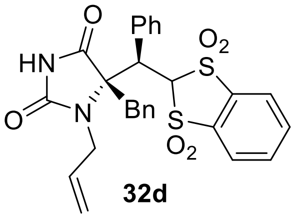

32d

HRMS (ESI) m/z: [M + H]$^+$ calcd. for $C_{27}H_{25}N_2O_6S_2$ 537.1149; found 537.1156.

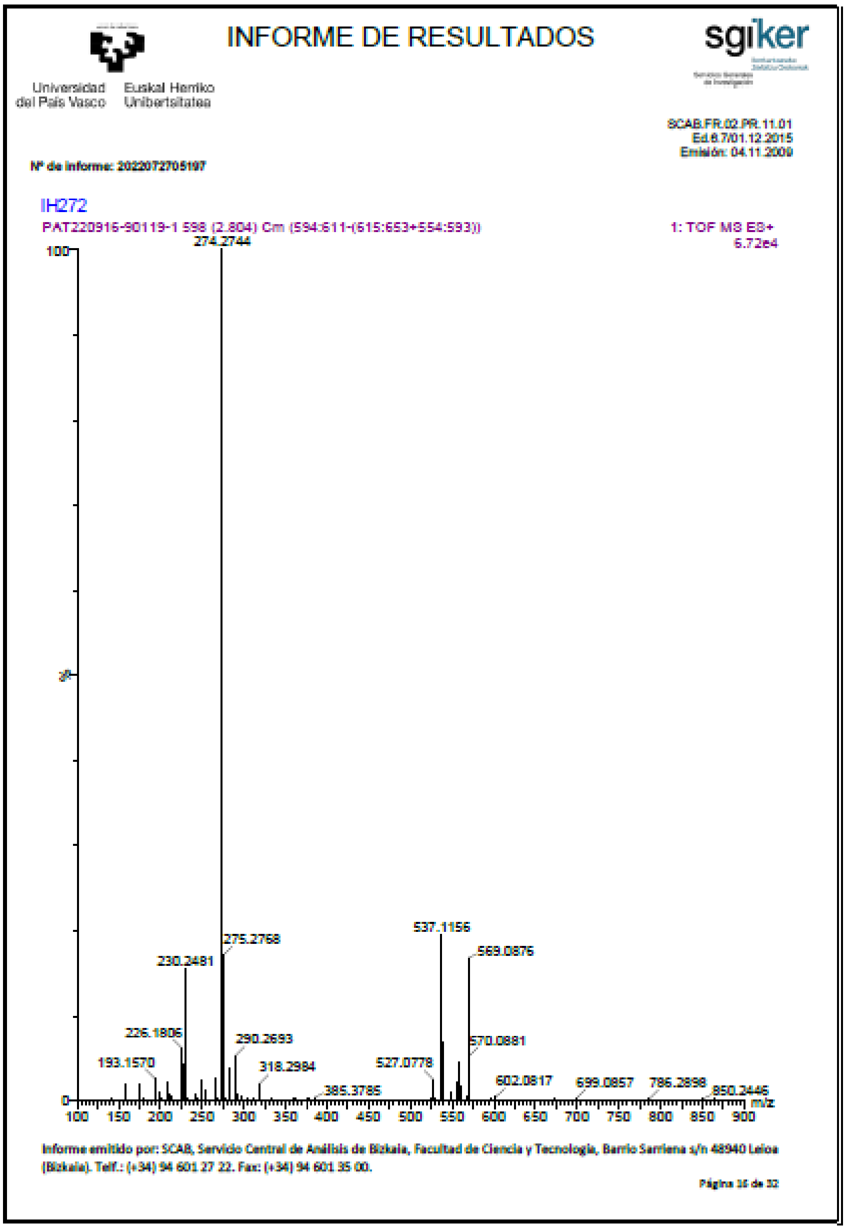

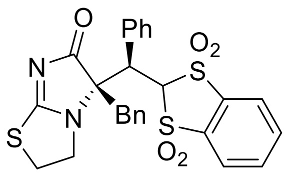

33

HRMS (ESI) m/z: $[M + H]^+$ calcd. for $C_{26}H_{23}N_2O_5S_3$ 539.0764; found 539.0770.

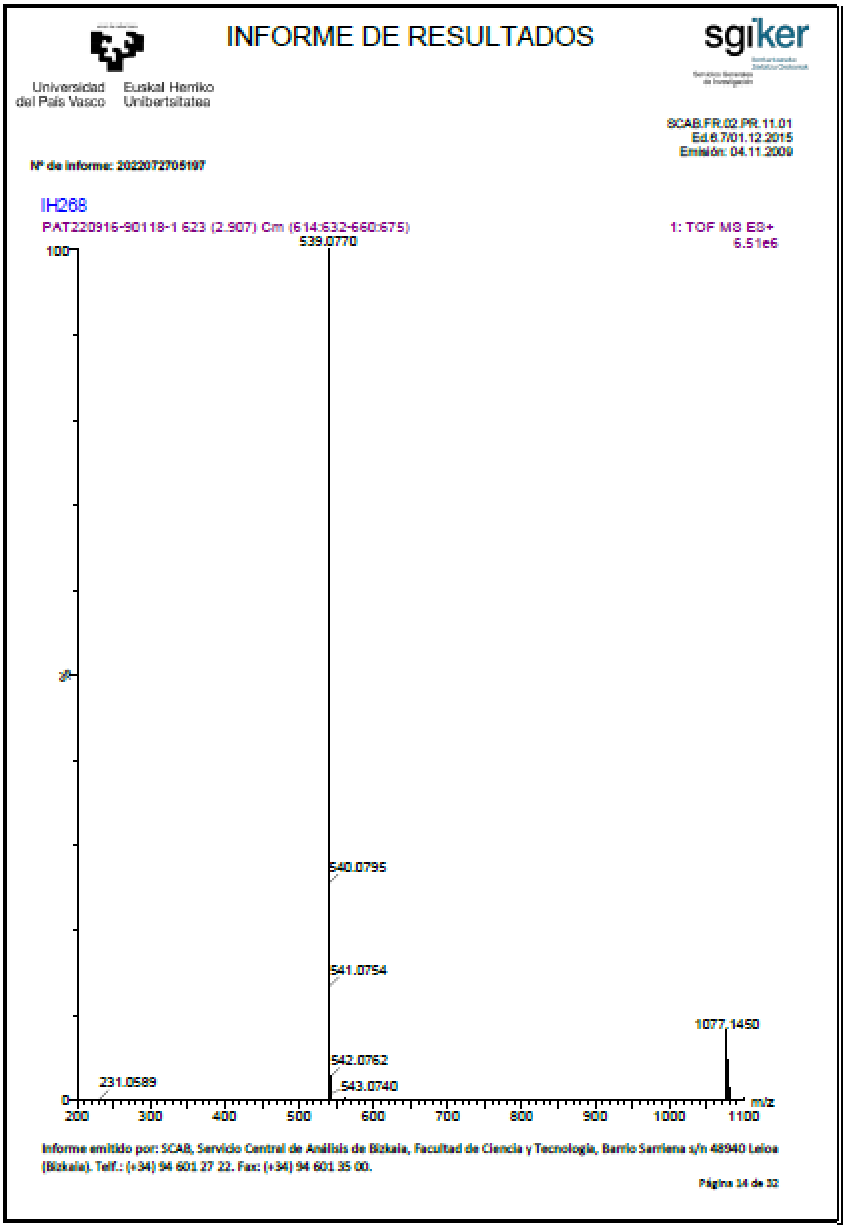

8. X-Ray analysis: ORTEP diagram of compound 25b

Method for sample preparation:
An analytical, filtered solution of chromatographically purified **25b** in CH_2Cl_2 was placed onto a small vial, which was introduced in a bigger vial containing n-hexane. The external vial was covered with a film provided with a small orifice so that solvent could evaporate slowly.

Instrumentation:

X-Ray analysis measurement was carried out using a four-circle\k-geometry Agilent SuperNova diffractometer provided with a Hybrid Pixel Array detector of the type HyPix with area resol mean of 10 based on '\w scans' method.

Crystal parameters:

Table 1 Crystal data and structure refinement for b20220004_LV411DUCuC.

Identification code	b20220004_LV411DUCuC
Empirical formula	$C_{31}H_{23}ClN_2O_7S_2$
Formula weight	635.08
Temperature/K	170.00(10)
Crystal system	tetragonal
Space group	$P4_1$
a/Å	12.24977(7)
b/Å	12.24977(7)
c/Å	45.9223(4)
α/°	90.0
β/°	90.0
γ/°	90.0
Volume/Å^3	6890.95(8)
Z	8
ρ_{calc}g/cm^3	1.224
μ/mm^{-1}	2.488
F(000)	2624.0
Crystal size/mm^3	0.17 × 0.15 × 0.06
Radiation	CuKα (λ = 1.54184)
2Θ range for data collection/°	7.216 to 137.996
Index ranges	$-14 \le h \le 14$, $-14 \le k \le 14$, $-54 \le l \le 55$
Reflections collected	84753
Independent reflections	12415 [R_{int} = 0.0759, R_{sigma} = 0.0515]
Data/restraints/parameters	12415/145/837
Goodness-of-fit on F^2	1.038
Final R indexes [I>=2σ (I)]	R_1 = 0.0453, wR_2 = 0.1158

Final R indexes [all data]	$R_1 = 0.0507$, $wR_2 = 0.1189$	
Largest diff. peak/hole / e Å^{-3}	0.21/-0.26	
Flack parameter	0.013(9)	
Friedel coverage		94%
Flack x		0.013(9)
Hooft y		0.030(8)
P2(wrong)		<10-99

[a] Esquema de pesado: $1/[\sigma^2(F_o^2)+(0.0783P)^2]$ donde $P = [Max(F_o^2,0)+2F_c^2]/3$.
[b] Expresión de extinción secundaria tipo SHELXL: $F_c^* = kF_c[1+0.001F_c^2\lambda^3/sen(2\theta)]^{-1/4}$

CCDC 2183172 contains the supplementary crystallographic data for the structural analysis of **25b**. These data can be obtained free of charge from The Cambridge Crystallographic Data Centre via http://www.ccdc.cam.ac.uk/deposit/.

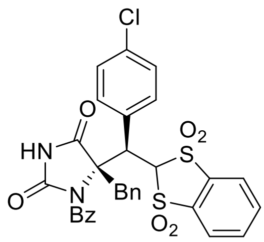

25b

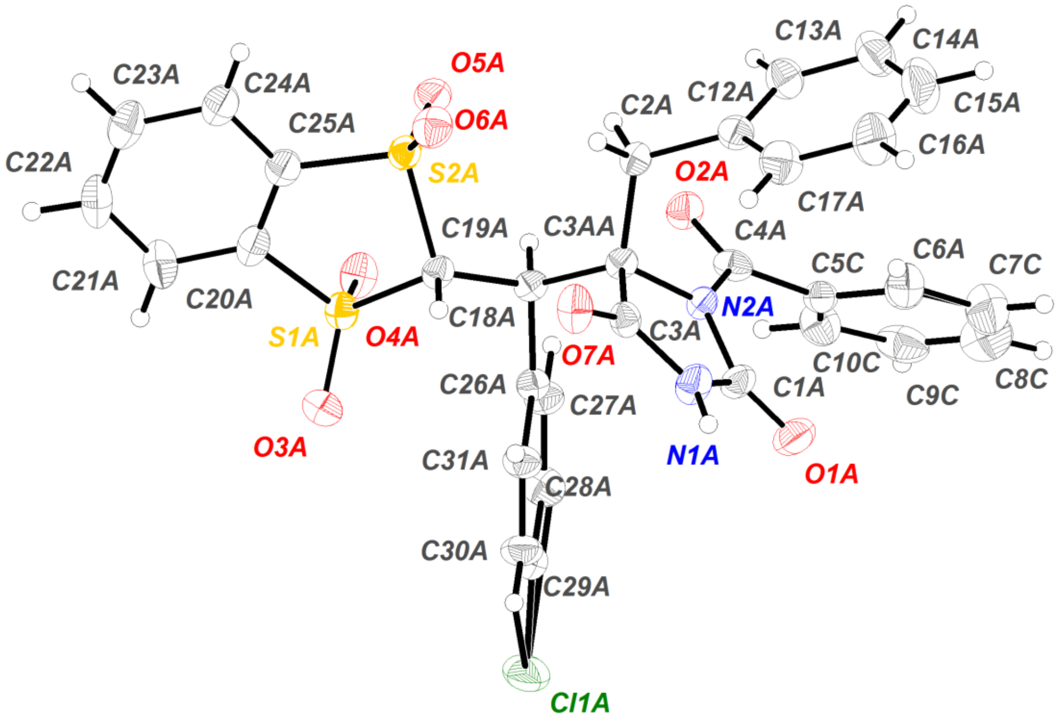

Figure S-1. Thermal ellipsoid plot (50% contour probability levels) for crystal structure CCDC 2183172.

9. Computational details.

All structures where optimized using the M06-2X[7] functional, with the Def2-SVP[8] basis set including D3Zero correction (Atom-pairwise dispersion correction with zero damping) and the CPCM(CH_2Cl_2)[9] solvation model as implemented in Orca 5[10]. Energies given in the manuscript where refined using the Def2-TZVPP basis set, and Free energy corrections included at 273 K. Def2-SVP geometries, Def2-SVP and Def2-TZVPP energies and Free Energy corrections are given below. Vibrational Frequencies where also calculated, and transition states where confirmed to exhibit a single imaginary frequency corresponding to the reaction coordinate. IRC calculations were also performed to confirm that the corresponding transitions states directly connected the desired intermediates of the potential energy surface.

[7] Y. Zhao & D.G. Truhlar. The M06 suite of density functionals for main group thermochemistry, thermochemical kinetics, noncovalent interactions, excited states, and transition elements: Two new functionals and systematic testing of four M06-class functionals and 12 other functionals. *Theor Chem Acc.* **2006**, *120* (1–3), 215–241. doi:10.1007/s00214-007-0310-x

[8] a) A. Schaefer, H. Horn, and R. Ahlrichs, Fully optimized contracted Gaussian-basis sets for atoms Li to Kr, *J. Chem. Phys.* **1992**, *97*, 2571-77. DOI: 10.1063/1.463096. b) A. Schaefer, C. Huber, and R. Ahlrichs, Fully optimized contracted Gaussian-basis sets of triple zeta valence quality for atoms Li to Kr, *J. Chem. Phys.* **1994**, *100*, 5829-35. DOI: 10.1063/1.467146

[9] Barone, V.; Cossi, M., *J. Phys. Chem. A*, **1998**, *102*, 1995.

[10] F. Neese, F. Wennmohs, U. Becker, and C. Riplinger, The ORCA quantum chemistry program package *J. Chem. Phys*. **2020**, *152*, 224108; https://doi.org/10.1063/5.0004608.

Coordinates energies of the significant structures

132

C2•4a DZ=-4405.294136590138,TZ=-4409.965262919227,Free E.Corr=0.93761001

N	1.08767407798996	3.32851186650489	1.55174132456423
C	1.15048940184358	1.11027513903570	3.86931018995127
H	0.64658596972834	1.13822695900935	2.89484572704336
C	3.41900396959555	0.60357530436320	-0.66730071358398
C	3.86964437496477	1.66393370226381	-1.51034842304555
C	4.47467555798908	1.34327563197095	-2.76181272600375
C	4.60707379414844	-0.02737399650494	-3.13391517196303
C	4.15946184691919	-1.02639538784265	-2.31796748748254
C	3.55050050270527	-0.71194021095027	-1.06828181146317
N	4.93692055029053	2.28043347616098	-3.63088670356419
C	4.82310386005353	3.54219657944085	-3.29927807807742
C	4.24273040006516	3.97603045842649	-2.08260252154035
C	3.75758617887064	3.05423338168605	-1.18300024881185
C	3.14539965490019	3.54294103507577	0.11914549706951
C	1.63635429460259	3.22212776284015	0.19217223301358
C	1.22384185615629	4.68257510492970	2.10090931125894
C	0.69540217618750	5.74467752814655	1.10585790918168
C	-0.10344828488003	5.01281217245553	0.01770802535446
C	0.86675802524193	4.14905548857360	-0.79664026880103
C	-1.14512192785023	4.10965164915814	0.71438513853812
C	-0.34094579308104	3.00868303913865	1.47031386960083
C	-2.15182829500575	3.51882630767944	-0.22852855351752
C	-3.46531630713829	3.74455784386990	-0.17654228926584
O	3.12895518779638	-1.77071871587792	-0.35631956791721
C	2.44167078466137	-1.53205269432758	0.85563659697954
N	3.93094242587977	3.08868700192925	1.26417491059886
C	4.58913487023053	3.96004040655336	2.00800635905069
C	4.71589238762532	5.43449325989590	1.95773980103728
O	4.32910646178100	6.31680491925081	1.22749701028136
C	5.41660846640821	3.88128050631408	3.14485764795546
C	5.43160897403123	5.35397224755424	3.27942443696485
O	5.78715480109389	6.19460373332979	4.08969580366234
N	5.82100993744343	2.78863174876721	3.83458218031237
C	6.39470467229071	2.82846721811841	5.10437206812174
C	7.02761521362090	3.99228287006824	5.54512771141899
C	7.39529278637588	4.13956574868732	6.87869754900566
C	7.30967393450212	3.05661129601950	7.75198657233851
C	6.79841652843301	1.85268153417684	7.26922658400206
C	6.30622137411013	1.72924521870534	5.96972466942363
C	7.79906074314629	5.47838405659950	7.43318319420214
O	8.40446023336634	5.57977594087970	8.48346256117522
C	6.71727888652361	0.68811839243872	8.21817876179024
F	6.65363949603764	-0.48069073277657	7.57375398460447
N	7.35881445091509	6.53530092455363	6.68503631977228
C	7.41785775830691	7.89157691461395	7.00428987191541
C	7.98011157765692	8.39611199562487	8.18299293579740

C	7.89198163864895	9.76272921868086	8.44546357506942
C	7.27867184632026	10.64884016594643	7.56633628578167
C	6.74774749488229	10.13135794196496	6.38429682971573
C	6.81991674338621	8.77671137332503	6.09279086501790
C	8.40060802932648	10.26493045510442	9.76939628533369
F	7.47253244773676	10.12411060319370	10.72555744523295
C	5.99319010191291	11.03877030585468	5.45143862329623
F	4.69318477959760	11.08752216469254	5.77528700575241
F	7.77664807919733	0.64006222884450	9.02666356831458
F	5.63621606497588	0.76244603910210	9.00511668813721
F	8.72032899796687	11.55875936463285	9.72702780664148
F	9.47931043966578	9.59534658529763	10.17830103934991
F	6.44787181522076	12.29151138512495	5.49364634526264
F	6.05881830037339	10.62542982966973	4.18554674333338
N	0.71413195216254	2.23196112839957	4.71002893603369
C	1.84419952723689	2.93903278425696	5.06876605884687
N	2.94523773881410	2.52969869413687	4.49628893827382
C	2.62376601852738	1.44951584378335	3.70891151523265
C	0.95975846749413	-0.26668284774800	4.53329932288330
C	1.53304390570282	-0.30985682056773	5.92621698784027
C	0.77041229650191	0.11868524125535	7.02115179063272
C	1.32265094723224	0.16122059548963	8.30190871746746
C	2.64337068219487	-0.24100917711732	8.50600731399589
C	3.40327969958675	-0.69275725374710	7.42593179497821
C	2.85487255084686	-0.71731677216322	6.14370627046362
C	-0.64030033495951	2.38507921509196	5.03776106853230
C	-1.04750514485363	3.51747752297180	5.93308416650926
S	1.87803132606126	4.23195208117975	6.23013390258891
C	3.69689144953596	4.46899155942934	6.31694880674545
C	4.02093478397613	5.59076543278448	7.26277879210110
C	3.79412102013943	6.92220818672807	6.88901983127053
C	4.12708492404319	7.96288939623484	7.75497335721659
C	4.70749590785761	7.68319980110267	8.99454409485037
C	4.93789023645713	6.35918052360265	9.37135758529966
C	4.58498009804147	5.31574762143811	8.51326038328049
O	3.42781979532775	0.82662658174670	3.03685508041345
O	-1.42948299338599	1.59556897117233	4.57241567567100
H	5.36452345385535	1.91470061925829	3.56801201010729
H	7.19615717891205	4.79885701864430	4.83983540212005
H	7.63158777603134	3.16767025378839	8.78839100284931
H	5.84581208558793	0.79793890410894	5.63263037799226
H	6.77983646256980	6.32757013003660	5.86918255965277
H	8.45375291332605	7.72550925850035	8.89473288004190
H	7.21721759919256	11.71373610508867	7.79190123162089
H	6.38311869054515	8.38234750250522	5.17156327066047
H	3.24356495120456	4.63626886766523	0.12469470012467
H	5.20264134535782	4.28483472971327	-4.00848164480067
H	4.18846270094115	5.04408609727766	-1.86143453434101
H	2.95463627758938	0.81811634148464	0.29339101612946
H	4.24806993220735	-2.07789854098195	-2.59424410881709

H	5.07483191776559	-0.24535811952210	-4.09485050699679
H	1.47771362895169	2.17780513629910	-0.10717825869853
H	0.64845896560364	4.70401104906842	3.04040081119860
H	2.26951301571600	4.87141843737450	2.38065072611953
H	1.56201101589888	4.80220813627536	-1.34676028461186
H	0.33013388541941	3.55514345046672	-1.54993828803314
H	0.05949586421903	6.47792901892201	1.62244014826433
H	1.53090180601649	6.29766028421642	0.64780989233907
H	-0.61350314059896	5.73357960051965	-0.63718022803717
H	-0.74451020661726	2.88008567851088	2.48417399197709
H	-0.44200646203827	2.03934419138486	0.95496856663689
H	-1.68809569579694	4.72993173146789	1.44608577366350
H	-1.75752856719202	2.84753185080271	-1.00240998606859
H	-4.15233967991273	3.28437520061905	-0.88999182174039
H	-3.89531732886366	4.40005441306114	0.58692703079305
H	3.93545899603395	2.10722076831474	1.53832580202539
H	3.07289541992222	-0.99120182682724	1.57575596887799
H	1.52597618976730	-0.94357650745837	0.67565662124518
H	2.16991597886139	-2.51287430798228	1.25970633921646
H	4.13260136487020	3.51683611687987	6.64583817894784
H	4.03758226560473	4.68948449138292	5.29901175991179
H	4.76319018933466	4.27744295530168	8.80580163624105
H	5.39771004515220	6.13574386696440	10.33561072890771
H	4.99632221774986	8.49906882287217	9.66040379162962
H	3.96072018809772	8.99726353119703	7.44837302996277
H	3.36403947569487	7.14005563163002	5.90774662477872
H	1.46265282042568	-0.99605850900336	3.88115148435106
H	-0.11334905702571	-0.49346323694515	4.54206025675336
H	-0.27345964319989	0.40779846200567	6.86961751597164
H	0.71647113611765	0.50174135625833	9.14323483014607
H	3.08106017383265	-0.21025791261901	9.50508101173940
H	4.42695125884915	-1.03076307042226	7.58310514669970
H	3.45603128318518	-1.06215826396480	5.29819480954279
H	-2.13892397393918	3.49424996038299	6.01251715911747
H	-0.60231566091761	3.39462224940113	6.93070279586647
H	-0.72517123696494	4.48344761831840	5.52003105985588

132

TS1-4a DZ=-4405.269740699564, TZ=-4409.938368456408, Free E. Corr.=0.93772425

N	0.72344155561929	3.13400287855556	1.61176278585053
C	1.45938001800300	1.83217339743770	3.91485552457009
H	1.19935302513568	2.52275405657078	2.61639125565495
C	3.73083832438777	0.69897312451699	-0.44170391776523
C	3.98053469387747	1.81989895131911	-1.28897893953528
C	4.69582234989821	1.61776878970055	-2.50690282079301
C	5.13271010108546	0.30150705609512	-2.84141290864067
C	4.88261086492805	-0.75723129898699	-2.01628977823090
C	4.17064446069118	-0.56112945773538	-0.79714374236894
N	4.99352137874167	2.62060252919125	-3.37419096915194
C	4.61446996101393	3.83659370588123	-3.07085604374748
C	3.90749328992176	4.15575828891198	-1.88601446085632

C	3.57600223020987	3.16185632154282	-0.99362513606206
C	2.83643378999241	3.53384277961341	0.28475350897746
C	1.41429565095606	2.94274479175289	0.30447236590905
C	0.64806055424915	4.56533454682420	2.00042905417358
C	0.07218509979899	5.39066310363647	0.83234515767777
C	-0.56195482382224	4.42200929155273	-0.17537098689150
C	0.54891899215227	3.58116213494548	-0.81557966710385
C	-1.52945114894876	3.49936070343171	0.59610711333284
C	-0.65548210024379	2.58480682830624	1.48599254074448
C	-2.43348715767556	2.69681029252083	-0.29583436434847
C	-3.76257973069747	2.79802050382483	-0.30578166682715
O	3.97550693471244	-1.66800427748548	-0.06213960179844
C	3.22553911903530	-1.56000834306513	1.13186077416377
N	3.62614681426568	3.18521768398945	1.45398315430823
C	4.35608701397976	4.08581748456197	2.10278592814869
C	4.39326917176892	5.56058280349413	2.10620071834507
O	3.82194124833606	6.43926308832369	1.50309203405399
C	5.35341897214311	3.99723508529772	3.08881512504187
C	5.35796610123196	5.46506830297939	3.27145567094301
O	5.84285785754427	6.27683982428384	4.03965032056542
N	5.90005658218145	2.89830499736435	3.65464989768586
C	6.50911976571196	2.92290607117000	4.91195416190192
C	7.25606162646730	4.02576161207960	5.31645347502651
C	7.64349849566153	4.15902830761582	6.65033285914510
C	7.42814502797313	3.11942533962541	7.55244859322050
C	6.76261697131723	1.97384633521607	7.10846607783158
C	6.28301701819910	1.86939732349686	5.80811894229158
C	8.14563161757883	5.46981588824972	7.18795121540508
O	8.80269323768439	5.54146727873227	8.20872367433893
C	6.42046542384242	0.91700462600061	8.12236447660586
F	5.89727030909248	-0.17596288745313	7.56443904368485
N	7.70530503376295	6.54757782116362	6.46790878732621
C	7.74625423802496	7.89152842354352	6.84040645511329
C	8.35778037987778	8.36235382658038	8.00920905813877
C	8.24372077955251	9.71214725915843	8.33649981448133
C	7.56485730519454	10.61712649075034	7.52678842883082
C	6.99092703022357	10.13647367274503	6.35018535493739
C	7.07920532419758	8.79642276554902	5.99982002625168
C	8.79281136016082	10.17765072722146	9.65727240981851
F	7.87979830254363	10.04758380955239	10.63048091255686
C	6.17843978059464	11.06410610058709	5.48876381452630
F	4.89542973534494	11.08741482470310	5.87693473231833
F	7.48743749128012	0.53696158559942	8.82780160597230
F	5.52191642042115	1.37757445708693	9.00314121394987
F	9.14467946553946	11.46391467027990	9.62923076897126
F	9.86085786225508	9.47491237793159	10.03547507190275
F	6.62164094982295	12.32039906202685	5.54972453852446
F	6.18547260332788	10.69333009679757	4.20811932072096
N	1.09873029194732	2.71144728965829	5.02172465205706
C	2.27713925872203	3.21549489481446	5.52912278297460

N	3.34139566876268	2.72784359782659	4.95556714065387
C	2.90632282565175	1.81346894754315	4.00551775524665
C	0.78388319948936	0.46475608985739	3.86897512798245
C	0.82746098090875	-0.20522222841108	5.22702674265351
C	-0.32046412028876	-0.30360066382507	6.02079424269337
C	-0.25442353505759	-0.84395465165413	7.30815765993346
C	0.96420063230155	-1.28864283597065	7.81994351172348
C	2.11680902115329	-1.19793414091111	7.03477575690214
C	2.04712690389419	-0.66331158612769	5.74935266128369
C	-0.22760090712019	2.95607471648210	5.39155433638760
C	-0.50317461556014	3.65218765019381	6.69450903717178
S	2.39405538288473	4.46558284349988	6.74938261779717
C	4.20930660992860	4.70972581580238	6.72677020290093
C	4.55619800158926	5.87536149860424	7.61228114318180
C	4.23312013709776	7.18155587283083	7.22175081057028
C	4.55993164426233	8.26400704226418	8.03870587411038
C	5.22803471051372	8.05209828492917	9.24612238337318
C	5.55951012249212	6.75348004594759	9.63772962158820
C	5.21443336956182	5.66856553579482	8.83001674645659
O	3.69530718724149	1.12414386612602	3.32594934535977
O	-1.12070681005448	2.57657415567784	4.66575406643414
H	5.37488545915267	2.03672233770284	3.45380759148658
H	7.47930174271706	4.80544185865183	4.59164103815415
H	7.73640392786570	3.23160000102197	8.59354300241572
H	5.68546662484879	1.01125459868526	5.49702278213588
H	7.08233538684024	6.35798590358883	5.68033512249278
H	8.88608302089844	7.67660782094498	8.66557567714731
H	7.48874850972533	11.66987293947662	7.80064210790638
H	6.60227211091768	8.42881446878220	5.08779177533439
H	2.74968966873315	4.62963510855813	0.29188703209843
H	4.86745503297695	4.63387097876241	-3.77697881674316
H	3.63281310382695	5.19355493363786	-1.68506396600608
H	3.18725173304712	0.81453083846111	0.49251278873776
H	5.21290446526760	-1.76765567290152	-2.26077608229113
H	5.67584639813130	0.17472451599508	-3.77873054840737
H	1.46610945927976	1.85491550290190	0.16617305099883
H	0.00211149309032	4.60823309114405	2.88877947165474
H	1.64881667685771	4.90323784833707	2.30340666117150
H	1.17249479976522	4.22410312376363	-1.45259290598443
H	0.12835627159470	2.80226804443751	-1.46581034344075
H	-0.67824130993967	6.09846659866646	1.20922076648964
H	0.86342295173688	5.97920882646347	0.34347455438122
H	-1.10881215094084	4.97728853179382	-0.94920717156219
H	-1.06243071739135	2.48688691002476	2.49904530696522
H	-0.55790949135656	1.57865550423606	1.05156829020219
H	-2.16032045935796	4.13400253620579	1.23848864347125
H	-1.94451509625223	1.98503301120591	-0.97280090539757
H	-4.37423941864270	2.19483769586307	-0.97991322025665
H	-4.28123343648063	3.49038594635309	0.36407826896537
H	3.74444627093596	2.20776950582807	1.72829208480709

H	3.69355016299953	-0.86175564523699	1.84331811923094
H	2.20035722135106	-1.21546622904124	0.91678101108474
H	3.18754858437968	-2.56361465658902	1.56783183249517
H	4.68060359690473	3.77945725697920	7.06817788291998
H	4.49884763074611	4.89493205395625	5.68522453061333
H	5.46374324675876	4.64952791885129	9.13842729425048
H	6.08915634500332	6.58325695362542	10.57680478411280
H	5.50675099870923	8.90003789098820	9.87435061024713
H	4.31472358963494	9.27811714950282	7.71811960608603
H	3.72678954304682	7.34741325325372	6.26680719852502
H	1.35582076506963	-0.12373003339232	3.13459853431143
H	-0.25220934395179	0.53216415815530	3.51519863605953
H	-1.27336535628177	0.05736270393920	5.62631979888379
H	-1.15999237406980	-0.91307725079600	7.91385177237598
H	1.01795474495166	-1.70567965432220	8.82701591632167
H	3.07611808309695	-1.54146454390135	7.42661939519583
H	2.94930349132411	-0.59798216253384	5.13551795386084
H	-1.57004838736896	3.52744717156335	6.90678383034124
H	0.09872888650036	3.23591243504669	7.51235668366392
H	-0.28242316710729	4.72569504523471	6.60539357323009

132

C2-H•4a$_{\mathbf{Enolate}}$ DZ=-4405.276221099813,TZ=-4409.948613956629,Free E. Corr.=0.94109940

N	0.88317097244091	3.30554361516308	1.72188804668671
C	1.56705416642110	1.59415896724401	4.16212943606349
H	1.38774430351196	2.75002850028835	2.45660998090712
C	3.70869843525725	0.65592268085122	-0.35130327984665
C	3.95631759483769	1.73421256190273	-1.25273894829631
C	4.61008829902430	1.46350218182568	-2.49248474229563
C	4.99551012656456	0.12339571599046	-2.79257240851440
C	4.75178915401270	-0.89359014805886	-1.91519574356039
C	4.09795671425507	-0.62956439656691	-0.67641738231186
N	4.89591665649188	2.42143640688224	-3.41281974250794
C	4.56687490933588	3.65973659521276	-3.14406725286250
C	3.92703941515111	4.04694382303822	-1.94171915076358
C	3.60913679643309	3.09955914841237	-0.99514908161603
C	2.93863742592500	3.55222840511961	0.29411016804863
C	1.48433020996399	3.05247338573622	0.36712927406475
C	0.91851546880932	4.75227377851882	2.11305371628500
C	0.31219692346495	5.58208973095430	0.96951896731692
C	-0.43980400790533	4.63461515941916	0.02469424142067
C	0.57910614952185	3.73299458150796	-0.68545386645603
C	-1.40371330102101	3.77889790310171	0.87740461829937
C	-0.53607368732040	2.81894938411918	1.71826747970923
C	-2.41648505735679	3.02037432937069	0.06575907448205
C	-3.73296793899468	3.20637325417027	0.15429028271092
O	3.90736485979364	-1.70276573415262	0.10729764339120
C	3.18019067317043	-1.53733457642911	1.30932472738037
N	3.72108667161044	3.19707615004972	1.46592330533693
C	4.47724690304088	4.10197620297462	2.08125786597645

C	4.57435671458983	5.57009525942109	2.01856641730306
O	4.04068743444250	6.44377159471548	1.37106683932100
C	5.45060777342324	4.01690730394560	3.09236880875662
C	5.50194699467324	5.49175316992957	3.21213721949612
O	5.98644769418794	6.32197396821474	3.96112210809783
N	5.92503084339291	2.91881122201532	3.71615028844202
C	6.49077860109775	2.96865094410606	4.99094310037759
C	7.23934369399066	4.06992735392888	5.39838089701687
C	7.57411803752940	4.23462319353069	6.74274844599965
C	7.30639624007245	3.22295471256820	7.66257042312528
C	6.64687835014681	2.07315983685297	7.22000806065353
C	6.21873295428244	1.94023009910225	5.90415016261266
C	8.07290748134306	5.55367238143882	7.26371226979212
O	8.67694442050072	5.64762338715571	8.31524607656005
C	6.26999426566879	1.03732414720117	8.24310768825885
F	5.72224861955364	-0.04729885759399	7.69355085286814
N	7.69445021684095	6.61504760754658	6.48640290890939
C	7.75540387767809	7.97037883750963	6.81022044683039
C	8.33624373894944	8.47092097688217	7.98199094832614
C	8.25028483529638	9.83566505323360	8.25168838453760
C	7.62719854511218	10.72567695495128	7.38302530436293
C	7.08312821191323	10.21383539306095	6.20495427254649
C	7.14655181561235	8.85932893466467	5.90985660403332
C	8.77292554877075	10.33297416257624	9.57151780877212
F	7.85144412969550	10.19940276034054	10.53592067770866
C	6.33037873353062	11.12787859563251	5.27739501134871
F	5.03041555408389	11.18459538549215	5.60097465360974
F	7.32450440506965	0.63692292691023	8.95746389403398
F	5.38099747283338	1.53002870578967	9.11545831769367
F	9.10208900773715	11.62462523044944	9.52781737213054
F	9.84946968626936	9.65530766612546	9.97277026101635
F	6.79240742563682	12.37812006653611	5.32253990064062
F	6.39199661189357	10.71834956788655	4.00974989640090
N	1.14759261685922	2.48140266282619	5.20179432950328
C	2.27884748356106	3.19866951877863	5.56713571742854
N	3.32865664273994	2.83281177794969	4.90094777777204
C	2.94274874804334	1.79369653459835	4.04386277330800
C	0.85720688593532	0.32132545376387	3.80989756168470
C	0.77930760955469	-0.61656315029563	5.00079587906415
C	-0.44807779433599	-1.00623494701062	5.54288708838011
C	-0.49838299180935	-1.84375733721006	6.66128838762649
C	0.68095481945568	-2.29675976438988	7.25089550350682
C	1.91403949344681	-1.90891153277404	6.71738059385137
C	1.96053482799487	-1.07486270232540	5.60264182174847
C	-0.18574155906552	2.62963332121947	5.61010460676982
C	-0.46920860995201	3.40475082876558	6.86606688523790
S	2.33054581041446	4.52895222846777	6.72456015579798
C	4.12460815367424	4.88009116573011	6.61082534196440
C	4.45740132597222	6.07318812836565	7.46322680683536
C	4.16450204362359	7.36789900988648	7.01626672939667

C	4.48430300288927	8.47608228000327	7.80160583714200
C	5.11345081188042	8.30071642196411	9.03528731425354
C	5.41185172074455	7.01286786247543	9.48556660008243
C	5.07472959485950	5.90386495592989	8.70841691140083
O	3.79495623612926	1.19040521079731	3.29395172436100
O	-1.06883910667029	2.12450766686628	4.95267492372136
H	5.33398826462206	2.08164293705013	3.53662595124560
H	7.50484284825133	4.82621697513480	4.66375238425170
H	7.57238520208537	3.35866755759260	8.71244665697772
H	5.62768883839313	1.07922029520224	5.58864649675231
H	7.10709655803697	6.41255758899675	5.67517436589915
H	8.82063806806901	7.79745755509831	8.68355901445397
H	7.57154129171876	11.79008536288277	7.61248817038374
H	6.69533761618845	8.46875954809438	4.99422393140758
H	2.91571369455592	4.65104870957688	0.26222147227805
H	4.80945231490259	4.42001889879348	-3.89324623797693
H	3.69603911959305	5.10045123370221	-1.76962526180880
H	3.22127802763920	0.82507374303507	0.60630986584971
H	5.04288156365395	-1.92231997372625	-2.13151499472216
H	5.49350794075181	-0.05563982829764	-3.74626477262984
H	1.46021817399342	1.96033393125419	0.25514455456230
H	0.34093494850791	4.82289828925474	3.04461607850171
H	1.95644169440548	5.02389550195791	2.33907397560337
H	1.19460084561348	4.33982902175078	-1.36422025916111
H	0.07640010911712	2.97527569376795	-1.30043497455174
H	-0.37117219465011	6.33369543857899	1.38516494696161
H	1.10187798145251	6.11710821023901	0.42172676634656
H	-1.01057316462436	5.20946885831648	-0.71588138209557
H	-0.86179901667114	2.74716545073192	2.76424393464784
H	-0.51504340779721	1.80660201772603	1.29062760645496
H	-1.94657707147769	4.45860860793128	1.55205525923604
H	-2.02253502013781	2.27288149555110	-0.63395757846384
H	-4.42983397857049	2.63681653189863	-0.46387569491914
H	-4.15478803558960	3.93683576928238	0.85100879571667
H	3.76135604648228	2.23683985401075	1.85523140702118
H	3.65852777107480	-0.80731583596905	1.98171680295127
H	2.15282573000900	-1.20053446828601	1.09076620991123
H	3.14437995575507	-2.52073031254137	1.78995835668296
H	4.65712790624007	3.98047118471734	6.94472196903659
H	4.36240931212273	5.05743736126411	5.55456679628524
H	5.30207691512672	4.89356060089456	9.06002956844428
H	5.91228750282730	6.87104568407226	10.44522105077464
H	5.38860905331786	9.16760517009771	9.63897696134240
H	4.26592295829414	9.48093406522174	7.43581628233739
H	3.69001142076650	7.50431323937603	6.04068786583478
H	1.47273082553296	-0.13978981519882	3.02172427319876
H	-0.14828097378762	0.48362064125199	3.40332370346314
H	-1.37148204759841	-0.63855085425524	5.08952108267594
H	-1.46478963430187	-2.13847910005496	7.07480394660334
H	0.64257364647567	-2.94795547287788	8.12588440661899

H	2.84280745385783	-2.25510212977234	7.17503742784754
H	2.92399564767105	-0.76543734787218	5.18690704325567
H	-1.50492326766803	3.19216551740911	7.15064515364454
H	0.21582554663050	3.13228980331876	7.67825124753039
H	-0.37014743769713	4.48309044190088	6.67225287931728

139

C2•3a DZ=-4596.820691445224,TZ=-4601.696882978957,Free E. Corr.=0.99154475

H	4.51968563457667	0.14228275317730	0.57493963334701
N	3.52120044869294	1.88537457831481	-0.91228883556109
C	3.95594420611138	-0.71514550527041	0.97018504472331
C	5.26017073075566	-3.97194687501425	1.58715364257065
C	4.43050301920549	-2.96270537603141	2.09047454904012
C	3.21967095256936	-3.32676736561505	2.69745774484425
C	2.85409096583252	-4.67027593221038	2.79925093397908
C	3.69347468939399	-5.66817592657270	2.29785071212614
C	4.89882662481121	-5.31633815060385	1.69231081100526
C	4.83802966128177	-1.51584552622148	1.94286179594469
N	3.39784645713289	-1.49903420905368	-0.13460120657008
C	2.03104668362800	-1.31669550584847	-0.13192870607877
N	1.59272543312986	-0.53604839507568	0.82315073280412
C	2.67911410920151	-0.14379382720579	1.56588457326616
C	4.24435875807119	-2.21954953477771	-0.99081902093462
O	5.40629603934051	-1.89540190755094	-1.06901367919228
S	0.94173835150861	-1.99485667348027	-1.30394521919983
C	-0.62695136049842	-1.34435988176488	-0.59567137433955
C	-1.80003237477759	-1.86678696248569	-1.37421967384692
C	-2.29069708866694	-1.16124761412233	-2.47911975587824
C	-3.39723966539996	-1.63688166999378	-3.18430053699102
C	-4.02929418472299	-2.81444054102659	-2.78210667266243
C	-3.54487661820357	-3.52279069063198	-1.67960501171289
C	-2.42919770086251	-3.05620694397955	-0.98448507056627
O	2.62154211523834	0.57230398778350	2.55217009865754
C	3.68041020097589	-3.39553705243974	-1.70708767753972
C	3.98458289357631	-3.57630353875243	-3.06020667468671
C	3.52201017291097	-4.71164559791750	-3.72127170178848
C	2.79209864222624	-5.67654171239291	-3.02203363286540
C	2.51838481681280	-5.50899666536172	-1.66287182235688
C	2.95305471870365	-4.36237876964719	-1.00279357645077
N	-0.40744684259662	0.97887391099350	2.40756952197883
C	-1.18149275976179	-0.16537700677223	2.60577919660498
C	-2.48910621519695	-0.22277401833844	2.12629408914748
C	-3.16813658068365	-1.43867428458244	2.07573782320863
C	-2.61484248822254	-2.57630419464828	2.66028263696279
C	-1.35472009081825	-2.47627878206092	3.25285207126462
C	-0.61986909828597	-1.29526495947886	3.21473775632082
C	-4.44461137927815	-1.58782275064504	1.29428910644616
N	-4.59731704299953	-0.64605264412146	0.31444370739277
C	-5.56032536518660	-0.61798854543754	-0.69483588799838
C	-6.59266936875792	-1.55677038887672	-0.81995533210547
C	-7.44449089077006	-1.48636170913182	-1.92096117554395

C	-7.31556858711166	-0.50259578906922	-2.89654941449613
C	-6.30127269506508	0.44099751735891	-2.73943345288128
C	-5.43693749268270	0.39899312202909	-1.65337443248159
C	-0.76230082185365	-3.71462274618583	3.86601084468108
F	-1.57973393687725	-4.25869235433128	4.76874685214863
C	-8.48593876193369	-2.56157055282989	-2.07600191606842
F	-9.47332938382648	-2.19171166533075	-2.89221392838364
C	-6.07558545933335	1.49533286719468	-3.78846762743461
F	-7.11757152937722	1.62175829592545	-4.60904015588094
C	-0.53406539010462	1.77440627302517	1.31776869407920
C	0.41762166582546	2.62833399440849	0.73319199903540
N	1.67830789218902	2.93178234276291	0.99150428003404
C	2.40095101732200	3.87106125613560	0.13716191016743
C	3.67354346407053	3.29296434109650	-0.51841984461872
C	2.44162403730895	1.69642723135384	-1.88860291940512
C	2.57641888809663	2.69221908246920	-3.06655766539544
C	3.98208844124260	3.30708151170669	-3.00229069949354
C	4.07557438482828	4.17284222466079	-1.73969124837697
C	5.00272300942125	2.15018948161783	-2.91833703173643
C	4.78022088858742	1.46514410228406	-1.53630450170771
C	6.42609135643267	2.58640004867061	-3.10332002066247
C	7.22924450390504	2.16233006484349	-4.08009567025641
C	-1.47851787907514	1.99390170006395	0.20364844443746
O	-2.52919785872091	1.53181081759381	-0.20932262131550
C	-0.53348195736408	3.04555510522232	-0.32356754117410
O	-0.51425327339628	3.81063051460325	-1.25857130676952
O	-5.21378671799817	-2.50880369314813	1.49421687172751
C	2.68552354953981	5.17058233571308	0.87127613806336
C	3.49585201828404	5.23074522208536	2.05082070800044
C	3.67400868967081	6.51407856695566	2.64720578472510
N	3.12023716519840	7.65190315173596	2.15111213008768
C	2.38135987325478	7.56196054197121	1.07368841635757
C	2.13280100827889	6.34029756252632	0.40168273925421
C	4.47138393395820	6.62346263110242	3.82491039911061
C	5.06435127356977	5.52583843483515	4.38003036577286
C	4.89192648101931	4.24151913664921	3.78642312995534
C	4.11737632975771	4.09580531198619	2.65229080596905
O	5.53286737770771	3.23526092332326	4.40506703677720
C	5.44943391476643	1.94065157284393	3.84476446111976
F	-0.53321730405414	-4.65450781366933	2.93942890361429
F	0.40157558296526	-3.47187978894366	4.47410749324650
F	-5.83991011178395	2.69179657711304	-3.24602274191471
F	-5.01078796870138	1.20428551750805	-4.54875230709528
F	-9.03413871911193	-2.89525469170071	-0.90590538059292
F	-7.95596041813778	-3.68105941334547	-2.58640294941652
H	0.53936847807223	0.93656792458976	2.79162443362239
H	-2.94826870431817	0.68336330112052	1.74221396289124
H	-3.15827803497816	-3.52229407983082	2.62840882768486
H	0.39354999157114	-1.25166246543523	3.61761904123410
H	-3.83278779117101	0.01853660958654	0.17662560242423

H	-6.71098453145499	-2.34208091181099	-0.07832572306655
H	-7.99027740172604	-0.46526672229555	-3.75131960361010
H	-4.63357726559320	1.13411103946422	-1.55244812590409
H	1.70066604561657	4.11478800669908	-0.67251773535352
H	1.93920321237763	8.48650767031825	0.68868534943479
H	1.49571359512583	6.33189494078688	-0.48518249940249
H	4.00242543102121	3.10652127609690	2.21519836741124
H	5.68048859419058	5.59677533985122	5.27738191101114
H	4.59065984101767	7.61558629423311	4.26215060415171
H	4.49113080336591	3.30805342729662	0.21502694861772
H	2.51252072689579	0.65536436085425	-2.24229907552653
H	1.46434332119172	1.78320638221378	-1.39248632547137
H	3.40289674238113	5.03892260506645	-1.83919885146721
H	5.09057904870951	4.57521511742020	-1.61247602881799
H	2.42398151185279	2.17988208463415	-4.02731768870571
H	1.81735669093933	3.48731226669251	-2.99401854102111
H	4.17896597065361	3.91529637537585	-3.89661293897029
H	4.78204386930731	0.37263760026386	-1.65160364824339
H	5.60145005356826	1.72024169313533	-0.84604015959220
H	4.76098893602318	1.43339224282281	-3.72015232276824
H	6.81756921257324	3.29604776867411	-2.36301729486392
H	8.26079053045316	2.51132211564559	-4.16327829729234
H	6.87655108082894	1.44753483061065	-4.82988247665048
H	2.15552595747650	2.43125063427194	1.73984431899205
H	4.40949562610536	1.58283872812107	3.80935983202470
H	5.86387644578766	1.92760230686272	2.82243770988156
H	6.04601935067450	1.28355039287845	4.48631050611558
H	-0.65296088404434	-1.67915571385070	0.44875256715202
H	-0.56820398660560	-0.25038298464969	-0.62512358487026
H	-2.04732426057596	-3.60682985466051	-0.11987227006828
H	-4.04192356000469	-4.43900540925204	-1.35596536390701
H	-4.91266617963333	-3.17292268000653	-3.31483412695688
H	-3.78382960881983	-1.06966096554634	-4.03268406725105
H	-1.81023494778805	-0.22546769197072	-2.77596462939875
H	2.73823230290489	-4.21913560275880	0.05949513259867
H	1.96197008139755	-6.27111577052246	-1.11527582199566
H	2.43862046924376	-6.56961356237879	-3.54025074275286
H	3.73695421381605	-4.84956637300317	-4.78173451758423
H	4.57188477668784	-2.82148058845133	-3.58626490873519
H	4.79050733322557	-0.98989013528289	2.90979994461054
H	5.87527097931396	-1.46609483231700	1.59009266626767
H	6.20023856868601	-3.69664225871065	1.10303035700746
H	5.56066119294991	-6.08820863029647	1.29564556703374
H	3.40536043942647	-6.71732653751740	2.38198899083909
H	1.91243064306459	-4.94009495140609	3.27626092509977
H	2.56013141542285	-2.55655159369874	3.10856680063866

139

TS1-3a DZ=-4596.798338082777, TZ=-4601.671353661248, Free E. Corr.=0.98678712

H	3.74070564293333	0.77157924553782	-0.22719132266227
N	4.00375002678702	1.78359128414265	-0.99777150656052

C	3.59182535035054	-0.53005512160918	0.41324621785245
C	5.64923661608187	-3.37001160544652	0.85180972391182
C	4.85161900979089	-2.40796368354752	1.47916945268994
C	3.93008747791438	-2.82830589293733	2.45161458377910
C	3.80651494547410	-4.17729046369072	2.78044057765423
C	4.60779036490117	-5.13070359112447	2.14456896050242
C	5.53200807097726	-4.72283877329736	1.18397913708490
C	4.90348224591642	-0.95326009053144	1.05563009047334
N	3.02050916269272	-1.45957450995365	-0.54801432708048
C	1.66485599237631	-1.48897754412086	-0.32526160911001
N	1.30015144660354	-0.79172537609601	0.71277197088970
C	2.45740158762905	-0.27175086037782	1.28257760827287
C	3.76587511799662	-2.13162647513652	-1.52287507294092
O	4.78534769035449	-1.64583029116858	-1.96064828981662
S	0.51531624626179	-2.25371524406609	-1.41509661800630
C	-0.97064298624209	-1.25338381017301	-0.99962598552391
C	-2.21621057504913	-1.98651514565462	-1.41306322372670
C	-2.91242158246342	-1.62080022349831	-2.56907258566012
C	-4.08118813220884	-2.29436173095673	-2.93441033912674
C	-4.57132898564151	-3.32564294434603	-2.13564127864640
C	-3.87589224373551	-3.70230550809896	-0.98118624824227
C	-2.69902080549407	-3.04515830623654	-0.62987123343655
O	2.45375555581656	0.36375419121845	2.35441296796674
C	3.28692655032175	-3.48461355103871	-1.91745349670228
C	3.41283565977600	-3.88291337218499	-3.25307960147227
C	3.01677053363016	-5.16312138136998	-3.62894760248575
C	2.52920110802844	-6.05281467888135	-2.66688921980239
C	2.43700882009508	-5.66586913319846	-1.32876685152961
C	2.80821649496827	-4.37741127325674	-0.95006614612040
N	-0.38055405944825	0.90505430615340	2.32091432005902
C	-1.24468996247815	-0.16326757661068	2.58333794326848
C	-2.58060934467882	-0.10484237752379	2.19386310655613
C	-3.37496107039021	-1.25122676779131	2.23266186794763
C	-2.89146561225753	-2.42186843231218	2.81274912105757
C	-1.57741434127146	-2.44189628268856	3.28545797894542
C	-0.74076697064117	-1.33823528952828	3.15756567838934
C	-4.69183566450469	-1.31027590751740	1.50977842452233
N	-4.76302883611155	-0.43493759120047	0.45977909163289
C	-5.69557093505094	-0.43002699365778	-0.57755179665131
C	-6.83646635290456	-1.24029593239780	-0.61432307872389
C	-7.63909568316004	-1.23082867454432	-1.75567415395796
C	-7.35173923582917	-0.43282547776333	-2.85786750484808
C	-6.22825702113591	0.39137172625566	-2.78931957840036
C	-5.41533257045807	0.41142528119664	-1.66557151016639
C	-1.03569388527978	-3.73786631721231	3.82448148294583
F	-1.84241991063558	-4.26437222690121	4.74832621028552
C	-8.81775798695947	-2.16659604562264	-1.78101397486575
F	-9.60841939297807	-1.95657589283707	-2.83361782418530
C	-5.80460716356603	1.20233321716828	-3.98288582496757
F	-6.79201227453200	1.36676244241991	-4.86231619673988

C	-0.44328125657927	1.62084173593135	1.17514918598566
C	0.56204204531615	2.36224169958983	0.53105663639386
N	1.84496157135819	2.59627156607381	0.78635084047404
C	2.59229149413338	3.60532015188028	0.05201310950024
C	3.99402078318799	3.14609496015773	-0.39580965960324
C	3.07440133405672	1.67571580655666	-2.15036594292312
C	3.33607317211591	2.82943508520692	-3.13943765722950
C	4.69058065517629	3.46275618879345	-2.79173338143363
C	4.57713346891432	4.15198293811333	-1.42655486895556
C	5.73687801656498	2.33060598315096	-2.71209899415587
C	5.39020664576432	1.49603749782972	-1.45650699300781
C	7.15499077640295	2.82393285736727	-2.67020701703310
C	8.07396487650461	2.55063860873681	-3.59633047349332
C	-1.38047823995645	1.83740935151439	0.05181449272907
O	-2.47797192702241	1.44461021802859	-0.29979197444985
C	-0.33696737752507	2.73795819156013	-0.57678829382604
O	-0.23268492815907	3.38990131712330	-1.59013457434106
O	-5.55261512669907	-2.12149188688258	1.79263430980319
C	2.66283086876059	4.90436767337651	0.84407543976935
C	3.26611827218083	4.98494826188387	2.14051676891943
C	3.23710314771405	6.25625611701161	2.78714571740223
N	2.67796177544132	7.36340664757016	2.23219105612539
C	2.12839843922987	7.25397474141250	1.04873737064009
C	2.09193835397040	6.04084605351743	0.31858790394138
C	3.81556945322021	6.38421037496466	4.08495733755078
C	4.38772778858440	5.31479760598872	4.71249851766233
C	4.41717230764229	4.04179850034673	4.07196717778536
C	3.86715344545506	3.88125923398697	2.81559561794066
O	5.00529702215999	3.05858476850238	4.77287553305525
C	5.12856470585373	1.78499009353913	4.17149946156295
F	-0.91561790747858	-4.65385605101176	2.85461740260151
F	0.16954406186723	-3.59411670747712	4.37780335154197
F	-5.36702931655134	2.41338788168838	-3.63368716150366
F	-4.79694978299416	0.60337746777996	-4.63485957120754
F	-9.56877297649254	-2.04495114445569	-0.68333889570271
F	-8.42264544038820	-3.44508341015120	-1.83285613115275
H	0.57975244793319	0.77268224306207	2.66373277939611
H	-2.97689896331711	0.83230746581284	1.80998745918736
H	-3.52201292678848	-3.31254104866687	2.84349580877645
H	0.30709462744337	-1.39125538679599	3.45700293819689
H	-3.93088204630610	0.12603839115525	0.26696570428227
H	-7.07449783976040	-1.88877019153804	0.22526145264183
H	-7.98386956865228	-0.44806739303695	-3.74512661022262
H	-4.52421562005532	1.04438692350285	-1.63628102819501
H	2.00291849955381	3.81869219114982	-0.85085155472422
H	1.67698096850519	8.15360850014347	0.61840061664658
H	1.60453236451509	6.01406853379980	-0.65835977799255
H	3.90726738591130	2.89941402603496	2.35126007479818
H	4.83258968951882	5.40041153600776	5.70475759334221
H	3.78099965353560	7.36719765414222	4.55636071241909

H	4.65671720757908	3.07753974128598	0.47617737117307
H	3.26458655366431	0.69365740166585	-2.60670534333661
H	2.04172153530547	1.66924022355581	-1.77358766409647
H	3.91897086048479	5.02831187276093	-1.51204841759502
H	5.55534150251493	4.52144008622333	-1.09011382169937
H	3.34400533881241	2.44609225570630	-4.16863354996655
H	2.53838775509322	3.58502220647065	-3.07534729710524
H	4.98233926734996	4.19149986105323	-3.55997309501020
H	5.45444246204016	0.41948960834861	-1.65318580996596
H	6.05990291757144	1.73885703725748	-0.61783186607037
H	5.62124672975030	1.70066041384506	-3.60849495166573
H	7.42993296911382	3.44539839617821	-1.80859674405670
H	9.09155358379986	2.93959627697292	-3.52084990373005
H	7.83451209331530	1.92607317991097	-4.46214095673164
H	2.24685130070574	2.14892631209764	1.61185903224775
H	4.14511728957982	1.35819437062774	3.91878215794442
H	5.73716861755050	1.84606332328810	3.25381534622702
H	5.63428238851691	1.14357099684524	4.90058973586382
H	-0.94454712778835	-1.08851829721496	0.08504781433424
H	-0.88233600608387	-0.29239119468604	-1.52159412372840
H	-2.14584150044326	-3.34598317473584	0.26438049165485
H	-4.25565425074829	-4.50902169382438	-0.35143638300438
H	-5.50107469869415	-3.83129855559656	-2.40335462862779
H	-4.62075888827638	-1.98990397887677	-3.83320814341910
H	-2.54527772795055	-0.79282171709010	-3.18096492736463
H	2.74960069131967	-4.07053797311617	0.09821692742829
H	2.07556706472076	-6.36854725363226	-0.57658618987580
H	2.22596885353560	-7.05849979109238	-2.96306615786588
H	3.09193540829040	-5.47271522089679	-4.67224491972526
H	3.80802854066085	-3.17845689926658	-3.98704344615466
H	5.04165175784167	-0.29742775885214	1.92952933549916
H	5.75596847375678	-0.79144080110305	0.38141751419100
H	6.35993983110323	-3.05615578330837	0.08302165561685
H	6.15984531241799	-5.46019598205903	0.68025626044419
H	4.50921406337515	-6.18741409447045	2.39884409083654
H	3.07858935078616	-4.48508416533753	3.53354243177704
H	3.30670747308831	-2.08643337889847	2.95802385111048

139

C2H•3a$_{\textbf{enolate}}$ DZ=-4596.8099710566,TZ=-4601.68360262449,FreeE.Cor.=0.99057522

H	3.45339389243968	1.22856373287251	-0.11316561736522
N	3.68366441558496	1.85622322583582	-0.90862726928397
C	3.59178665604225	-0.81130500249034	0.80599488228799
C	5.70947929498601	-3.61563525268327	1.23136720612103
C	4.81509933930671	-2.70984564350215	1.80860762007253
C	3.80233439941307	-3.20182603086088	2.64472091260508
C	3.69053039452736	-4.56665875302124	2.90121523298420
C	4.58758961495998	-5.46505632849654	2.31487522781051
C	5.59694704933795	-4.98652339522728	1.48111446594243
C	4.86453211736577	-1.22894606450930	1.46990138609727
N	3.06627869474547	-1.53792061522962	-0.28943547581668

C	1.74801455999187	-1.16917374138405	-0.41552007184787
N	1.40197013493046	-0.30114726209213	0.48617334667071
C	2.51366467011849	-0.09833759837609	1.32302669129263
C	3.83111068608303	-2.36764038536995	-1.14626602733805
O	4.91724801044219	-2.00202690080582	-1.52934473198301
S	0.72566815193206	-1.71403352348004	-1.74658098202096
C	-0.78438164685553	-0.74443902021981	-1.36310077170864
C	-2.00121795222851	-1.48867118069418	-1.84455963546967
C	-2.77315280792622	-1.00462467857366	-2.90414597222786
C	-3.90325009594820	-1.70644501980309	-3.33572926980550
C	-4.27729428906422	-2.88797472638500	-2.69966364418591
C	-3.51045292513923	-3.37741352476144	-1.63581841708959
C	-2.37527845011362	-2.68760529234710	-1.21926932578075
O	2.44506032097072	0.67471370524300	2.35661787673863
C	3.24477043735234	-3.69368337244862	-1.46489026681787
C	3.65090813291218	-4.34906341197257	-2.63374457212672
C	3.15657396796225	-5.61865190921516	-2.91812789717739
C	2.27780403010278	-6.24074643366482	-2.02581275665850
C	1.88996690951240	-5.59627527750216	-0.84912220998366
C	2.36715644939827	-4.31850476305890	-0.56838291623218
N	-0.34033265891874	0.95012616943168	2.36608490023103
C	-1.04977566363021	-0.24922290345007	2.48033374817014
C	-2.39383928213985	-0.31592485353564	2.11818047644741
C	-3.02841047861328	-1.54996799591574	1.99001826896385
C	-2.37496565284058	-2.72198720480351	2.37101073741654
C	-1.05730551768391	-2.63388833515884	2.82137847247230
C	-0.38001917804350	-1.41763704041256	2.86443550811423
C	-4.36176894175714	-1.66599493451281	1.30701022868774
N	-4.60298806903921	-0.64576644105802	0.42707940391855
C	-5.59744877427974	-0.58655993698377	-0.54872747897251
C	-6.62174315522322	-1.53122401092599	-0.68468438435179
C	-7.49647897952091	-1.43304897487675	-1.76650738231547
C	-7.39882335308114	-0.41539595055583	-2.70903134550345
C	-6.39487978209151	0.53768587920015	-2.53586309640821
C	-5.50954953071730	0.46877006871636	-1.47003953968231
C	-0.30269177435017	-3.89774241530963	3.12915438020598
F	-1.09215703304750	-4.84060874618947	3.64119128341606
C	-8.53572831342449	-2.51095254413348	-1.91465796470464
F	-9.40260677523044	-2.24817977022418	-2.89255980317815
C	-6.18949372430053	1.61982816254614	-3.55946293086240
F	-7.25198505125571	1.77772682244718	-4.34832942433152
C	-0.52333622732345	1.77503955782274	1.30809321392645
C	0.41639706445364	2.61136249293662	0.68233502614046
N	1.70888115705772	2.83047525177360	0.89378871911600
C	2.43840833552610	3.80853035022404	0.10253555670664
C	3.78555425236531	3.27857491650683	-0.42823671227774
C	2.65498552660201	1.67564032725637	-1.98576739120659
C	2.91509954670581	2.71864833544389	-3.08380598392330
C	4.32397195816847	3.29283585588846	-2.88350230445703
C	4.33529117226230	4.12901504038244	-1.59604509127133

C	5.31088415158903	2.10743658989042	-2.76067540056709
C	5.03181828276275	1.42243951140572	-1.40708754588859
C	6.75399371237460	2.51390777588108	-2.87089088971162
C	7.57841212374133	2.08748218268447	-3.82679725299169
C	-1.51966186076347	2.03688962036644	0.25079608328486
O	-2.60222849070963	1.60193751852349	-0.10438309206474
C	-0.55908547667515	3.05242976428576	-0.33264278843611
O	-0.53678858485948	3.79311370500897	-1.28983812336821
O	-5.09687779089277	-2.62000896897845	1.47840019237756
C	2.64587569333784	5.11066937768907	0.86145109984884
C	3.32190303812603	5.16399815406864	2.12250305519374
C	3.42342826297231	6.44462001648785	2.74260029940857
N	2.92278412193247	7.58225381920216	2.19291184176709
C	2.30882749346201	7.49727297555402	1.03955080546786
C	2.14114887991392	6.27826917078913	0.33746644455572
C	4.07707321941577	6.54905873622428	4.00611861571980
C	4.60389121167849	5.44936687973244	4.62090581383904
C	4.50857853048693	4.16840249959210	4.00263926347629
C	3.87527601167016	4.02765934664337	2.78304023262306
O	5.06841904429801	3.15783279285545	4.68646233461774
C	5.09805548162217	1.87636967302326	4.08685388943470
F	0.25051216498736	-4.41833097805347	2.02029765281972
F	0.69419645940850	-3.68995056342003	3.99327207308769
F	-5.92905195502443	2.79942064857756	-2.99148082961141
F	-5.14706766464197	1.34153937065909	-4.35586995843402
F	-9.23687456291473	-2.67999439159070	-0.79044870954774
F	-7.97221732800926	-3.69374289966810	-2.19406723562299
H	0.66202089075116	0.87293973092578	2.62715666928037
H	-2.91862708318994	0.60783823338390	1.88594598726478
H	-2.88219066761990	-3.68306977755666	2.27473949363002
H	0.67283868668921	-1.36813415417283	3.15104122847970
H	-3.85949857345113	0.04422802929231	0.29912800781021
H	-6.71078804624975	-2.34803182607966	0.02731252706999
H	-8.08664744880006	-0.36033050011645	-3.55202053677317
H	-4.71010338921425	1.20688177746079	-1.36206681854315
H	1.80218872146426	4.04431118756614	-0.76289843568886
H	1.90744227997693	8.42221271911197	0.61340248428948
H	1.60174347365689	6.27148160784134	-0.61200840950792
H	3.80215856234910	3.03548741285093	2.34430786543900
H	5.10763108948030	5.51639845470195	5.58611886648455
H	4.13978738717269	7.53855997295023	4.46066314122559
H	4.51354673988791	3.23472567712512	0.39121394066128
H	2.77434073664337	0.64444607381353	-2.34619363213249
H	1.66219200514368	1.75377852831598	-1.52563739182429
H	3.70669778967740	5.02074765390882	-1.73053384609219
H	5.34859469973583	4.48133752382528	-1.36306162561185
H	2.82920687699616	2.24028012634456	-4.06780500114786
H	2.16687186232497	3.52318661877776	-3.03794412350504
H	4.60507843295288	3.92021401095839	-3.73904828945430
H	5.02828238239728	0.32467500629893	-1.45441647847330

H	5.75540219466271	1.72854365243227	-0.63834632413947
H	5.08977483296877	1.40068082815311	-3.57533649213548
H	7.12668118253023	3.20449088776328	-2.10427360828889
H	8.61782256359204	2.41915064980593	-3.86947185423852
H	7.23720689080829	1.39061572432742	-4.59797840114696
H	2.15686740271832	2.21129749400895	1.60493545610969
H	4.08660499514238	1.49879351589179	3.87077555247126
H	5.67049453743982	1.90500977754865	3.14414081864893
H	5.60212073831575	1.21256596712246	4.79710874757401
H	-0.79826849523151	-0.62483419597157	-0.27102055723451
H	-0.70477665108178	0.24660844575505	-1.82998144791327
H	-1.76205507577978	-3.07616066172164	-0.40084251843795
H	-3.80217568785249	-4.29937615992145	-1.12933071315507
H	-5.17339921527385	-3.42342321439227	-3.01929519695166
H	-4.50116164819360	-1.31146915156228	-4.15917018642217
H	-2.49319381540589	-0.06731570712471	-3.39160583866318
H	2.07562258768762	-3.81465717435007	0.35517744292448
H	1.21462905610734	-6.08712191016070	-0.14682134896782
H	1.89643451525404	-7.23876098105884	-2.24879674242374
H	3.45750655669002	-6.12850121627997	-3.83430060288795
H	4.34659419243155	-3.85036550288668	-3.31069668609173
H	4.94921347623935	-0.62652845201396	2.38729362348321
H	5.75207236467250	-1.02325990236272	0.85224658914003
H	6.49012368940220	-3.24414482630107	0.56294957657171
H	6.29832889606913	-5.68121435947224	1.01514728349402
H	4.49511897520533	-6.53548208811264	2.50675312593838
H	2.89351704487939	-4.93097290240340	3.55125304945092
H	3.08678346353125	-2.50127189638113	3.08569818779161

169

IM2 DZ=-6232.229485473162, TZ=-6238.418863867380,Free E. Corr.=1.19927353

C	-2.83016151351988	1.28142215398498	1.21123963276245
C	-5.50050758803797	0.68209328086823	0.61318231824572
C	0.01198434855331	-4.31314025901691	0.94388725843908
C	-1.34931849700064	-4.44067305059949	0.55037930201761
C	-2.25530273344206	-5.14900964318414	1.38889206474276
C	-1.78497437296598	-5.64168651537428	2.64152650812217
C	-0.48239399178810	-5.47266103860985	3.02337647117550
C	0.43758036931315	-4.82120682339531	2.15279123324909
N	-3.54394332364694	-5.40326084295926	1.03792555110704
C	-3.96487693001955	-4.94418479930023	-0.11131114191919
C	-3.16807778943202	-4.17137458485317	-0.99521252880634
C	-1.85715887811078	-3.90711408567104	-0.67726603137586
C	-0.91440144769304	-3.09431995314390	-1.55480751580220
C	-1.43310221496754	-2.81393893906255	-2.97526195252487
N	-2.38560034642086	-1.64452431794434	-3.09922320590493
C	-1.68726625656248	-0.32040317829935	-3.26150910887127
C	-0.96233641709139	-0.28320363656390	-4.61363131685014
C	-0.88065548839828	-1.71521918120605	-5.14714465091458
C	-0.33026835679806	-2.60247356098656	-4.02011466250821
C	-2.31451406128764	-2.14416583253256	-5.53585689837592

C	-3.23404219071278	-1.88579516972441	-4.31280849639694
C	-2.39118443094560	-3.56083010103171	-6.04064415211312
C	-2.69054846950538	-3.88260292752770	-7.29863665917212
O	1.70655677572158	-4.73488584809153	2.60104373414931
C	2.69015247913887	-4.21295781031802	1.72925917755633
N	-0.52033070061759	-1.88200249422027	-0.85031292337170
C	0.73262392000927	-1.47606001556642	-0.78803940283000
C	2.02492455671641	-2.10149414380177	-1.13318172359514
O	2.36357271822826	-3.18276823744018	-1.56409478231501
C	1.37516291889568	-0.30753511520070	-0.32164235396693
C	2.69753045807655	-0.80481714271195	-0.75962285410050
O	3.84609608191333	-0.39744995643156	-0.81089293441974
N	0.87810347323601	0.84727237408923	0.16991321464999
C	1.68225229913700	1.92144478398109	0.56133270073489
C	2.98405217731086	1.66646727041245	0.98884139500009
C	3.88093300155120	2.70802165406479	1.20805588478138
C	3.44670343715205	4.02966290280443	1.13648751327443
C	2.11312050856542	4.27536564751397	0.80538282034668
C	1.22546107977799	3.24427790459137	0.50086242995048
C	5.32634040314752	2.41114865534704	1.48831917466462
O	6.03264497536387	3.16126552508912	2.13391055142029
C	1.66530028815375	5.71069789778744	0.76187974144380
F	1.61385248588721	6.23765020156254	1.99612153199487
N	5.74904687472695	1.24143018359607	0.91502178755696
C	6.99385661122173	0.62387873270020	1.03482091822925
C	8.06485903940745	1.15341463688685	1.76290621572026
C	9.26161945454719	0.43576732959856	1.83277178264701
C	9.42480817249700	-0.78968063739457	1.20227667787256
C	8.34614215200303	-1.30224805884881	0.47605798037219
C	7.14545914490679	-0.61646006553914	0.38723589853600
C	10.40280518026383	1.04827489018288	2.60090105345341
F	11.37202413941027	0.16678809087949	2.84783773794668
C	8.52224414441945	-2.62553135641954	-0.21888869442024
F	7.37915833393066	-3.09149723591372	-0.71990944177096
F	2.50932206814469	6.47287384041443	0.06048182624074
F	0.45540209541133	5.85832743130190	0.22802418996901
F	9.99626960535165	1.53856619841044	3.77439370961686
F	10.95582466316429	2.06431536486904	1.93081173099575
F	9.39221943570345	-2.53463950280930	-1.22961164012789
F	9.00213648423383	-3.55100345585535	0.61639840917650
O	-2.62013700001935	-0.42960868673612	-0.46883329069056
C	-2.58811900081271	0.78612793924594	-0.06223910585687
N	-2.75522437976955	2.69015751653907	1.11095086433161
C	-2.44701553467944	2.95524498823213	-0.22993228001688
N	-2.31653413567092	1.86647299219699	-0.92192143776365
C	-5.61006356773115	1.79137446807027	-0.33372507013408
C	-5.21022129970285	1.67262306085272	-1.67599082338338
C	-5.30633039580433	2.76083368009064	-2.53641933167680
C	-5.79378644833868	3.98388769631135	-2.06826823600478
C	-6.18325609648939	4.11752367541534	-0.73384531307810

C	-6.08287561448033	3.03120918985835	0.13148450326769
C	-2.73463098627517	0.53242534201594	2.50259735988491
C	-1.28793085236740	0.23191794025824	2.83895529742497
C	-0.44131158710747	1.24495380832911	3.31141709776054
C	0.89951581813323	0.98194976821726	3.58701605903703
C	1.41470048272482	-0.30235925168865	3.39394697457084
C	0.57857788881886	-1.31723765196609	2.92623732503207
C	-0.76369532930651	-1.05183539907458	2.64206766707577
S	-2.30582575465162	4.57653194845104	-0.90848322956975
C	-1.69522751262276	4.14025715779414	-2.57873611874184
C	-0.21098392695347	3.88016628102610	-2.62504610526876
C	0.69870406791829	4.94624556063626	-2.59555042289414
C	2.07207631245763	4.70988160574043	-2.62639951874271
C	2.55930651173524	3.40118817310924	-2.69462584563672
C	1.66265459392652	2.33449150974997	-2.73212013076728
C	0.28731887974385	2.57510841042846	-2.69252717555401
C	-3.48437390962740	3.51616729068763	1.99158758377454
C	-3.17224070850889	4.97113267083629	2.02279211560844
C	-4.24751499302609	5.86683043314602	2.07607195444130
C	-4.00360226455547	7.23356332050379	2.16880556973788
C	-2.68846974291518	7.70017004696770	2.24810517027433
C	-1.61881728452669	6.80452507691554	2.22748950501991
C	-1.85660876925399	5.43625880224402	2.10179810342801
C	-5.52869029756784	-0.64076484471394	0.32945911937087
S	-5.37751447428030	-1.83885456068598	1.63613888180658
C	-6.45176178519472	-3.05842152615470	0.91616990302377
C	-6.72011180845869	-2.87227668645501	-0.43221130350474
S	-6.06293297004170	-1.39432831756008	-1.17963377116461
C	-7.46450332590085	-3.79682772460654	-1.15673581000392
C	-7.92634910644642	-4.92802230868683	-0.48239628331218
C	-7.65241190968919	-5.11689978375315	0.87772032355642
C	-6.90583431683519	-4.18219201863167	1.59607700165768
O	-5.93578545642729	-1.28199444804126	2.86211657226310
O	-4.04237633701084	-2.42493451326938	1.69937872279426
O	-4.95433922775405	-1.77440570009883	-2.06180480592730
O	-7.15645534989420	-0.64482240403924	-1.78214401376933
O	-4.32818817059587	3.03762648017719	2.71539826077712
H	-2.99776820513356	-1.55059635818859	-2.26386564961491
H	-7.68032874867930	-3.63518102819227	-2.21438348856906
H	-8.51320252505897	-5.67134895659813	-1.02323610536276
H	-8.02697035947699	-6.00702827613998	1.38449105295643
H	-6.68161127511549	-4.32289600317694	2.65469821624989
H	-5.45428091349100	0.95811776116617	1.67364618878950
H	-4.76350618731982	0.74095686716516	-2.02984540690034
H	-4.98099109735966	2.66016540772296	-3.57332615913443
H	-5.86081291398533	4.83838493527904	-2.74396702843666
H	-6.55740563973655	5.07368365030838	-0.36446013261539
H	-6.37557425292387	3.13128467030311	1.17846236643674
H	-0.13458566524764	0.96745689978062	0.19927691209636
H	3.29193772577293	0.63740748897278	1.15055459132296

H	4.14626753687587	4.84625878601317	1.32270428383841
H	0.20543939721787	3.46885020903251	0.17778221829869
H	5.08815495082662	0.76205315197072	0.29927886604916
H	7.96153268123544	2.10920434350508	2.27063175792578
H	10.36386010720553	-1.33934883763903	1.27562726402248
H	6.30539128801701	-1.03211584386802	-0.17429101009522
H	-0.00828785465875	-3.70225289678452	-1.71288637925172
H	-4.99786005259098	-5.17506990436495	-0.39608465613420
H	-3.63542912024295	-3.80799232667382	-1.90979275321130
H	0.72564761073571	-3.82763923953888	0.28470017158501
H	-0.11089787967819	-5.84658681074717	3.97837019961666
H	-2.49887604399292	-6.16487623077437	3.27903241106853
H	-2.02558847189637	-3.68904204311668	-3.26780427714677
H	-2.46108295376793	0.44847707368257	-3.14761971242723
H	-1.01282028767068	-0.20952514191356	-2.41143229033729
H	0.54723456745378	-2.11353855477780	-3.56632949811325
H	-0.00162522232488	-3.58536487583116	-4.38402166510907
H	-1.49603761306133	0.35957454478323	-5.32780569187574
H	0.04557212160210	0.13202508291319	-4.47583548115442
H	-0.23153201965599	-1.76632131229535	-6.03035520341282
H	-3.85918771781010	-0.99415843009513	-4.43985977179453
H	-3.89343365847286	-2.73387235021516	-4.09213723182101
H	-2.64445541488235	-1.48128633473797	-6.34878559125362
H	-2.18060684260068	-4.36343010706299	-5.32317832341730
H	-2.72352225521262	-4.92335724182131	-7.62716711104833
H	-2.91327813769873	-3.11011610110255	-8.04084226240861
H	-1.32585672211199	-1.24187062378241	-0.55301498446308
H	2.72380479048135	-4.77844964919183	0.78504326157861
H	2.49640373023796	-3.15098298737232	1.50104435154149
H	3.64898799083288	-4.30042385784568	2.25028402722366
H	-2.27727429965956	3.28026104128620	-2.93199483581807
H	-1.95697920935162	5.02512958975068	-3.17472484203230
H	-0.42143242995439	1.74910273329290	-2.69039465354054
H	2.03293201918472	1.30664095539241	-2.77855118769102
H	3.63496175445103	3.21584887963077	-2.71523149990412
H	2.76722166118818	5.55059450388377	-2.59454435744282
H	0.32303077075720	5.97077345971115	-2.53547203543925
H	-1.02637549317023	4.72659472652302	2.07249817204751
H	-0.59503305790886	7.17058019683120	2.30121237743375
H	-2.49634552623575	8.77118826324814	2.33238365507274
H	-4.83752065081594	7.93652015161371	2.18961236702424
H	-5.26811675292196	5.48130173307708	2.03551764050746
H	-3.26405296457118	-0.41955765829787	2.39274609362194
H	-3.21092168534963	1.09310076295382	3.31696766356642
H	-0.84348757742873	2.25028301236771	3.47018352618208
H	1.54755773032778	1.78275648148001	3.95018432533469
H	2.46282962653835	-0.51382148829656	3.61509584998510
H	0.96899568677517	-2.32816109628141	2.80077170812098
H	-1.41875132888377	-1.84832223818903	2.27422818931485

169

TS2 DZ=-6232.228636980629, TZ=-6238.416493647162,Free E. Corr.=1.20051916

C	-3.04813298264733	1.23064981046744	1.25224659122274
C	-5.35860680789330	0.74343504333587	0.66918256541509
C	-0.04324914901416	-4.36219418673322	0.99740445037565
C	-1.39954855216563	-4.47143318106137	0.58221964633457
C	-2.33104961915125	-5.16016754129600	1.40902990639525
C	-1.89031319759340	-5.65062184131185	2.67309839661858
C	-0.59283945921974	-5.49554531044715	3.07751142575932
C	0.35229159673469	-4.86396972677195	2.21910444474889
N	-3.61699097822086	-5.39619581222757	1.03703303994110
C	-4.01342554712341	-4.93084683159076	-0.11820999617318
C	-3.19049951449781	-4.17296296605411	-0.99106642072536
C	-1.87856783505347	-3.93599398820958	-0.65554740787275
C	-0.90600329244377	-3.15418397515994	-1.52707359062462
C	-1.40360633034901	-2.86855337498939	-2.95277004690533
N	-2.35993898125896	-1.70373288739684	-3.08276525606519
C	-1.67046357105417	-0.37243394940271	-3.21718202268147
C	-0.91283346640252	-0.32053211099801	-4.55211432661298
C	-0.81790968379898	-1.74519280961757	-5.10290853971977
C	-0.28528896161275	-2.64551864000498	-3.97839360721493
C	-2.24158810235664	-2.17565156111102	-5.52455822842650
C	-3.18417060370143	-1.93822353257257	-4.31562355394756
C	-2.29957372832360	-3.58593589281931	-6.04945239519078
C	-2.55461292267887	-3.89194771312858	-7.32107655529914
O	1.61308067484010	-4.79001121244682	2.69079883137961
C	2.62658566583514	-4.31673411152344	1.82590163098333
N	-0.48519892202035	-1.94841968609075	-0.82820884676562
C	0.77097698374941	-1.54291019359146	-0.78576862137959
C	2.05747393800393	-2.19401024726083	-1.10158537226583
O	2.38563993614414	-3.29043783500844	-1.49718701044342
C	1.41600345317606	-0.36025840499823	-0.36934234479093
C	2.74314468084156	-0.89115529750058	-0.75967094517974
O	3.89663317021471	-0.50293909427768	-0.79598898916123
N	0.91693081523337	0.82184437792057	0.05514835865253
C	1.71297122932349	1.89014034177855	0.47161407455810
C	3.00644006834739	1.63639990291514	0.92536388175274
C	3.89374107193240	2.68012146806067	1.17280062414848
C	3.45642245331737	4.00079588685860	1.10623802403797
C	2.12748075773404	4.24351895910964	0.75488364597597
C	1.25127864567699	3.21247684084239	0.42254402457135
C	5.33715464952180	2.39318140014871	1.47259252972637
O	6.03023963990172	3.15493921169116	2.11888671260513
C	1.67320647628154	5.67674451801353	0.72031544085048
F	1.66256594562933	6.20771588780485	1.95388952304850
N	5.77816701365108	1.22364048911787	0.91311600455439
C	7.04017524642981	0.63994119001903	1.02574779508231
C	8.11024657707748	1.21493771653978	1.71891064727698
C	9.33123390385498	0.53747857405844	1.77066669576430
C	9.51878931653804	-0.69347256697279	1.15875051527921
C	8.43860867169518	-1.25413998153466	0.47044546523194

C	7.21534937872979	-0.60785226051524	0.39864690464961
C	10.46716883583191	1.20981788765021	2.49507108821523
F	11.50681691997579	0.39427856955137	2.66969166527454
C	8.63901037936027	-2.58445838392159	-0.20435978577532
F	7.50791993503583	-3.06973939388667	-0.71426446712244
F	2.49079257700629	6.43957305018164	-0.01140659110457
F	0.44586357841738	5.81784191353110	0.22567005362335
F	10.09093032309806	1.64470607756182	3.70043236684835
F	10.90840773647110	2.27795093687815	1.82276173205385
F	9.52163023551195	-2.49574130577536	-1.20421717460357
F	9.11807771904199	-3.49407382687076	0.64852887654313
O	-2.61489503218225	-0.48042564918919	-0.36719936183018
C	-2.62517105245548	0.72562877301589	0.00794932963710
N	-2.91069582554401	2.63747181387704	1.15083539112344
C	-2.46929590524903	2.88990186820243	-0.14808798163887
N	-2.25857968837055	1.79403435656405	-0.81993384821191
C	-5.53369682304048	1.85570589378229	-0.27522507933408
C	-5.11938703173218	1.76852119097861	-1.61578861556758
C	-5.27055402377755	2.85112732704908	-2.47533697173928
C	-5.82973104393844	4.04405897620834	-2.00907348229406
C	-6.24211665776120	4.14575816280200	-0.67955659941707
C	-6.08973586114058	3.06183215244454	0.18324386291907
C	-2.87590843298098	0.51550585018137	2.56036315467340
C	-1.40983755889624	0.24141533209639	2.82275940771985
C	-0.55845655094937	1.25726195422658	3.27982506810553
C	0.79668885238711	1.00689595328199	3.49413976231092
C	1.31939144112649	-0.26645057462319	3.25592575756738
C	0.47884845481977	-1.28259242615637	2.79885562410514
C	-0.87652876991105	-1.03045508645980	2.57390992966215
S	-2.32884290228139	4.49888168039386	-0.83589045103352
C	-1.77571066772315	4.06517566246948	-2.52669243332589
C	-0.28730359606457	3.85477759706936	-2.63509668336545
C	0.58807414449744	4.94930133387237	-2.62873368280174
C	1.96593223879229	4.75639748393030	-2.71331950325726
C	2.49053588613418	3.46443758267200	-2.81323422772608
C	1.62729479009574	2.36990510081599	-2.82715986438330
C	0.24785853014623	2.56691738425235	-2.73307302387455
C	-3.58442747104226	3.49738478930222	2.03941750810943
C	-3.20418655359323	4.93567010728091	2.07031925400861
C	-4.23860626501725	5.87825386940274	2.12342911420851
C	-3.93368954021108	7.23197848098510	2.22460734650305
C	-2.59926097268944	7.63791477302584	2.31362758175774
C	-1.57101703868108	6.69525954070517	2.29175561821913
C	-1.86853136934553	5.33962324720367	2.15543448873518
C	-5.44212009501698	-0.59390870554343	0.34645468853224
S	-5.38698952803331	-1.80217549489742	1.63071262667479
C	-6.46580934816240	-2.98934157229077	0.86485148742532
C	-6.68855122021073	-2.77109764334028	-0.48469851207006
S	-5.98846358190113	-1.28142180643309	-1.17068071563942
C	-7.42825545969949	-3.66487798713148	-1.25116203159446

C	-7.93760430306955	-4.79692798582984	-0.61295451540038
C	-7.70939347649261	-5.01833724711086	0.75101453812066
C	-6.96139645258224	-4.11651873709493	1.50912304442249
O	-5.97637429632488	-1.24384862517557	2.84583004863678
O	-4.08088040771928	-2.44756010817682	1.76578960200619
O	-4.90711750446276	-1.67569412972069	-2.08892006379486
O	-7.06490782529642	-0.49499170719563	-1.76464191651139
O	-4.43336947521916	3.05303339894077	2.77795329025006
H	-3.00164677950362	-1.62785707924558	-2.26795394871029
H	-7.60662227000949	-3.47835565138311	-2.31164763463822
H	-8.52483732177516	-5.51597209603402	-1.18537462247129
H	-8.11987768517280	-5.90898704788975	1.22828075073162
H	-6.76778349844670	-4.28370382789999	2.56990734279112
H	-5.47246677650119	0.99361153339744	1.72896197922246
H	-4.63521992880700	0.85734837391380	-1.97406457608425
H	-4.93822577024416	2.76852834924648	-3.51173939402054
H	-5.94100288048485	4.89529599589313	-2.68301061333212
H	-6.68036101268255	5.07447586390445	-0.31031559693520
H	-6.40713932575505	3.14034112259170	1.22460667263584
H	-0.08615133889548	0.98578389642588	-0.03834636717722
H	3.31689825936783	0.60825794570007	1.08452938930494
H	4.14911654228719	4.81760728105630	1.31557285374660
H	0.23406355486073	3.43758587055082	0.09307348161073
H	5.12760440767315	0.72543851636160	0.30229845081385
H	7.98998275412108	2.17721321584289	2.21029902373064
H	10.47803923420944	-1.20935216059882	1.21492762441446
H	6.37724110046104	-1.05890218937895	-0.13775497412305
H	-0.01331248794170	-3.78502365629654	-1.67288677521116
H	-5.04513471683640	-5.14478751241183	-0.41936110249831
H	-3.63856697751010	-3.80567483839838	-1.91412290756716
H	0.68850687953633	-3.89027069281907	0.34760159720556
H	-0.24407480928201	-5.86602945167874	4.04234810118793
H	-2.62288740944130	-6.15884671786831	3.30143407210189
H	-1.98842898150182	-3.74511179306034	-3.25710178684330
H	-2.45561731491818	0.38823566155000	-3.12330856028766
H	-1.01838325519095	-0.25713960521159	-2.34978570986872
H	0.58477134491814	-2.16051025563283	-3.50684751705791
H	0.04966895517348	-3.62386122211910	-4.34857066577730
H	-1.42882083366625	0.33227783898810	-5.27014828276540
H	0.09250585298886	0.09037049944845	-4.38669631546643
H	-0.15166512115830	-1.78174146102117	-5.97398417229523
H	-3.81478907307066	-1.05094114702766	-4.44210472266709
H	-3.83992462822355	-2.79439522817166	-4.11475592719013
H	-2.55940768537592	-1.50339786120762	-6.33457895222463
H	-2.11144691834182	-4.39717483480326	-5.33546746940806
H	-2.57334272794335	-4.92842121553182	-7.66394045210877
H	-2.75299612337815	-3.11033920193974	-8.06062675545502
H	-1.26218791909047	-1.32259863752728	-0.49737105062849
H	2.65849740197204	-4.90315960770422	0.89439165759605
H	2.47233675317833	-3.25445377814779	1.57169985381656

H	3.57331903029256	-4.42273967737364	2.36530282582007
H	-2.34528046451923	3.18357872644284	-2.84533143983669
H	-2.09326584155182	4.93550928059052	-3.11727107849905
H	-0.43512870659304	1.71982825902430	-2.70823476772819
H	2.02789727621974	1.35497950358903	-2.89641243671450
H	3.56984862395033	3.31401307081483	-2.87678044825819
H	2.63535708777170	5.61805615216356	-2.69854122717096
H	0.18307189891434	5.96081040015346	-2.54379940504325
H	-1.06868006205800	4.59505713447175	2.12076735001868
H	-0.53265624693955	7.01537203319919	2.37161255096937
H	-2.35929559516084	8.69847536406792	2.40675919132720
H	-4.73512340792339	7.97174093612122	2.24464981855552
H	-5.27522122271015	5.53883530457819	2.07569396764407
H	-3.39496973516802	-0.44576319248233	2.51487390833161
H	-3.31912134825100	1.09863255265331	3.37752922770854
H	-0.96739227555890	2.25163473274156	3.48294988649501
H	1.44842373217916	1.80858145271558	3.84873620853298
H	2.37788066509602	-0.46838455426637	3.43274184992137
H	0.87592751261908	-2.28530952521029	2.63400233267978
H	-1.53796811102171	-1.82650571747683	2.21689724307979

169

IM3 DZ=-6232.251404891817, TZ=-6238.432983907621,Free E.Corr.=1.20301448

C	-3.68054317197641	1.02616960789970	0.57140241586636
C	-5.09388569438936	0.85966493525460	-0.15520133199198
C	2.35581612180342	-2.73095033784563	-0.16179053067676
C	1.05984900509961	-2.72007714424498	0.43491990361848
C	0.95319733206088	-2.50579724717994	1.84174214662973
C	2.12611688717071	-2.17090371087396	2.58100810411822
C	3.35316938401827	-2.13497202452303	1.98053893160093
C	3.47982921351963	-2.46430939317310	0.59989525763384
N	-0.21309715156720	-2.62280991877055	2.53049426402342
C	-1.28786237856596	-2.94891309013555	1.86129705267873
C	-1.31264873158519	-3.10820953507890	0.45253585084255
C	-0.16388316288885	-2.94893925768866	-0.28530326221341
C	-0.20800207979622	-2.84218969347620	-1.81140788941187
C	-1.44812146448704	-3.44725977808858	-2.48549196370191
N	-2.71819890121557	-2.61537325112604	-2.43099604292954
C	-2.78720581923693	-1.54588917190123	-3.49664347300041
C	-2.91537677628157	-2.21691165569780	-4.87108570970185
C	-2.53585621111672	-3.69439073450170	-4.72886498382291
C	-1.20651807431988	-3.77587113490464	-3.96575901225489
C	-3.65873931766341	-4.38366915004223	-3.92276059310907
C	-3.88107875566137	-3.54687065112101	-2.63957175139849
C	-3.38830506494783	-5.83791047721079	-3.64182051784885
C	-4.10531278680202	-6.83479723551616	-4.15953469336786
O	4.74409167190808	-2.49541335940360	0.14117990628947
C	4.98539509323839	-2.98387352848914	-1.16286325797004
N	-0.06210343564996	-1.42287707548307	-2.14920389479899
C	1.14142288232565	-0.84912691748894	-2.28027633140015
C	2.42691752044415	-1.19775795641167	-2.91258659982564

O	2.83514460058878	-2.08651415478413	-3.61691092131126
C	1.64562400874015	0.31508057550166	-1.71213564654460
C	3.02539511211448	-0.01234061863988	-2.14894157093744
O	4.14910720199429	0.36214274399851	-1.89572377034463
N	1.04409121302712	1.20093690966479	-0.88427048632968
C	1.72987178893620	1.81928662508335	0.15677693798395
C	2.87801209371555	1.20897112511087	0.66072235918691
C	3.63235483667654	1.81649072867890	1.66025555437779
C	3.19113468937985	3.00206515277074	2.24569362697294
C	2.00599937924817	3.57392660291231	1.77444084453080
C	1.27423128115034	3.00908509692565	0.73496621935150
C	4.91780403400862	1.17750838687939	2.10239571516601
O	5.34858359724400	1.28996998286908	3.23286575247676
C	1.53662627788600	4.85632402336942	2.40416951372503
F	1.23536071990121	4.68576446987219	3.69933861367667
N	5.54081673100461	0.48064936327396	1.09950356269693
C	6.70514508372575	-0.28352260305646	1.18030676317582
C	7.32632972896906	-0.63598915879635	2.38177846136621
C	8.46619539170467	-1.44556129676410	2.34711997736734
C	9.00558865735250	-1.90977819841165	1.15609856469653
C	8.37394424615539	-1.54228087711616	-0.03616637714273
C	7.24555937041643	-0.74012915330687	-0.03459796401070
C	9.11482236657097	-1.79490301477749	3.66066539902395
F	10.06499163858421	-2.71959705796800	3.52179434324683
C	8.91260769503671	-2.08152554275452	-1.33374656600545
F	8.64696578103965	-3.38939953236853	-1.45973435634685
F	2.47782606342212	5.80283810374967	2.35121472082645
F	0.44679033640014	5.34505825547499	1.81113809039942
F	8.22329130129903	-2.26558892741154	4.53638927556023
F	9.68205185933823	-0.72582316559938	4.22811031714342
F	8.38064478703274	-1.47265870962716	-2.39293531580981
F	10.23740244346645	-1.95080817728404	-1.41292667298758
O	-2.21128471414468	-0.24812055072289	-0.86273887618774
C	-2.54173691078432	0.83463766655613	-0.41438525849654
N	-3.46682139166725	2.44963756697935	0.89940738447314
C	-2.54898364930775	2.95185902615946	0.00825749393801
N	-1.94473726781093	2.03572805000873	-0.71775186643106
C	-5.27478707665395	2.03349923351771	-1.11038223840489
C	-4.59387470884009	2.08873376286294	-2.33178538488626
C	-4.70582639114020	3.20850162538936	-3.15835795629263
C	-5.50193361636825	4.28703829039669	-2.77338272883218
C	-6.19713048790831	4.23345169229628	-1.56329113002816
C	-6.08542940694995	3.11354324129017	-0.74130509026876
C	-3.46868703667957	0.11944594585516	1.79862623029374
C	-2.32572836172600	0.58390130602220	2.67441410591336
C	-2.59990036895802	1.15656035048000	3.92335119277919
C	-1.56966501619989	1.61994442095695	4.74331061224843
C	-0.24339990904990	1.51842415698303	4.32311331673181
C	0.04415983802608	0.93944975655205	3.08543164778281
C	-0.98697530516214	0.47577721793538	2.26592284227010

S	-2.35865476446766	4.65907662846273	-0.25266194757628
C	-1.57840926652247	4.68517112686865	-1.91397232256731
C	-0.07458743673327	4.74240273185371	-1.90053198005797
C	0.59298488870636	5.77000956796012	-1.21849266076253
C	1.98393164934479	5.81508552562904	-1.19453057182130
C	2.72762882487301	4.83696325872674	-1.86303535770304
C	2.07129311256596	3.82425058507587	-2.55943157933934
C	0.67435584585443	3.77711124191311	-2.57738768286744
C	-4.21988030689114	3.08773778219817	1.89655591546763
C	-3.70447545190361	4.36039560704712	2.47030897296599
C	-4.62686529474844	5.38950114545835	2.69473834617476
C	-4.21117587799786	6.56430882663042	3.31327698041656
C	-2.88765957447061	6.69077671199008	3.74346241182935
C	-1.97631832942899	5.65325022062050	3.54280927279402
C	-2.37497072802889	4.48782409006788	2.88928351759634
C	-5.30263788428763	-0.48177661684381	-0.85323285374718
S	-5.49074554288624	-1.84690252908981	0.18277647570265
C	-6.67175965339464	-2.79036595946139	-0.75640008539666
C	-7.12407020325149	-2.15373373743547	-1.89213765834781
S	-6.45158875535803	-0.51932787342576	-2.15829835570357
C	-7.99581615271962	-2.77979576831766	-2.77671877887407
C	-8.41697756780404	-4.07216736958956	-2.46055031441555
C	-7.95776384504061	-4.71752020074080	-1.30452119290830
C	-7.06409761806210	-4.08618453468191	-0.43856683326794
O	-6.05424806895338	-1.50389137345080	1.49816833107258
O	-4.27444886977808	-2.69386120823244	0.25891369388163
O	-5.83210484389742	-0.55550010623901	-3.49974467805280
O	-7.53536919730639	0.46252755535111	-2.02249642866039
O	-5.23188869600271	2.56624929665023	2.30159759174077
H	-2.85210471158310	-2.14725544582519	-1.51503629878382
H	-8.33068801335875	-2.27421477196177	-3.68404559442848
H	-9.10696383499890	-4.59056657964464	-3.12765611521627
H	-8.29553698451562	-5.73071425523441	-1.08288580493315
H	-6.67981705769085	-4.58471436455399	0.45309658129088
H	-5.82150792104474	0.97545112404900	0.66180235257043
H	-3.97065619619011	1.24789613797183	-2.64449042036305
H	-4.16731491447533	3.23433647220069	-4.10750307049970
H	-5.58789615312972	5.16335482896326	-3.41813424222596
H	-6.83283846812929	5.06686883861912	-1.25931266067992
H	-6.63326702535853	3.07028176518618	0.20222401083357
H	0.05080812271869	1.42394315520542	-1.00767690671029
H	3.16696057822536	0.22947847032903	0.28690290282629
H	3.77052337117254	3.46957771558536	3.04396869454532
H	0.36316412473648	3.48616009852506	0.37191854965699
H	5.15182839814483	0.57050118796703	0.16120853847259
H	6.92375562943513	-0.28858144821994	3.33063725401818
H	9.89376119861489	-2.54213582998657	1.14921208166628
H	6.75660184220696	-0.46999790087564	-0.97326823210448
H	0.64198834992833	-3.38739213021482	-2.25006203362882
H	-2.22165999416857	-3.06868001316737	2.42103440775482

H	-2.27953842096304	-3.32517592658643	0.00456642920746
H	2.46731384869015	-3.01108593951048	-1.20795940199573
H	4.26205340230276	-1.90577187388520	2.54111875690950
H	2.00845902717449	-1.97034179366029	3.64686587778140
H	-1.69549764444389	-4.36539134540025	-1.93694560861996
H	-3.66568773258262	-0.94012395694165	-3.24531904210388
H	-1.88572010189899	-0.93753205048715	-3.41464170944652
H	-0.48846745235635	-3.05606283033237	-4.39131424583772
H	-0.75235847325524	-4.77388578725039	-4.03057715630226
H	-3.94492554933887	-2.12332658852395	-5.24567079765468
H	-2.24824740874020	-1.71607501929616	-5.58492938556168
H	-2.44387101629429	-4.17085058832371	-5.71314904253891
H	-4.76033988883017	-2.89941318019224	-2.73576612200104
H	-3.98510465127956	-4.15739655042262	-1.73370206906133
H	-4.58103181432215	-4.32283382826354	-4.51888816985679
H	-2.54883048512860	-6.07673625960638	-2.97746276411046
H	-3.87031843887664	-7.87829133564748	-3.94030199643108
H	-4.95369407751769	-6.63484332246211	-4.82106051185955
H	-0.72574484768999	-0.83882319081556	-1.62492819372212
H	4.50393411108915	-3.96328065003747	-1.31074490564211
H	4.62652053354497	-2.27983212007007	-1.92778395629015
H	6.07057277568407	-3.09963914190015	-1.25607772305277
H	-1.95979842769584	3.82322836596159	-2.47387926406019
H	-2.00723233415576	5.60211796339913	-2.34494573788895
H	0.16027064994577	2.97348116748602	-3.11105072981410
H	2.64722966524574	3.05474788700918	-3.07831195338049
H	3.81836039831129	4.86592482803975	-1.83743241895301
H	2.49196232578916	6.60971060575477	-0.64507079790945
H	0.01442910764387	6.52571356027505	-0.68103243708521
H	-1.66101908392390	3.67459461027152	2.72396268545450
H	-0.94937145537371	5.75111838556538	3.89268252429420
H	-2.56458048717093	7.60451484448451	4.24509172542906
H	-4.92041939179940	7.37773418216033	3.47177729557840
H	-5.66194679525153	5.25989018852253	2.37316477667533
H	-3.27091733299239	-0.89217952027275	1.41307163751297
H	-4.39779515066654	0.07976368526965	2.37785789241567
H	-3.63850867508439	1.24289964595615	4.25197805892948
H	-1.80674711409654	2.06600396962590	5.71085346105849
H	0.56661166162338	1.88309436658937	4.95705215916963
H	1.08221934698529	0.83744985033633	2.75953560393762
H	-0.73863894939215	-0.00334153540867	1.31251807536128

169

TS3 DZ=-6232.218969964530, TZ=-6238.393090605124,Free E. Corr.=1.19686861

C	-3.49982205814506	1.06855063522643	0.50477670103784
C	-4.74390831550884	0.70135764310594	-0.39071188182161
C	2.33868331267711	-2.66812678753832	-0.37759462853531
C	1.07251141093295	-2.52222255729019	0.26952559735940
C	1.04877896154177	-2.36384744031806	1.69023344475952
C	2.28026566054864	-2.19472204581543	2.39175005582060
C	3.47831602251679	-2.28063425838857	1.74350794954415

C	3.51294259631586	-2.56877281648976	0.34720585065697
N	-0.08768661047717	-2.40656312529769	2.43126161182960
C	-1.22181488097770	-2.57913278438954	1.79995478820557
C	-1.32332088668560	-2.63434044115282	0.39063148209902
C	-0.19980509555330	-2.57675187666833	-0.39794631026426
C	-0.34145877498502	-2.53714238566147	-1.92059249857829
C	-1.65927062811570	-3.12426950106382	-2.47387957957569
N	-2.94914722718027	-2.30421601123420	-2.54790797044422
C	-2.94787333915829	-1.43289978383324	-3.76537047822663
C	-3.02768583280945	-2.27180178840634	-5.05529734729948
C	-2.64209898091053	-3.71493435769816	-4.72000722358405
C	-1.36271365961420	-3.66552243374891	-3.89088282574394
C	-3.77393565135074	-4.31920985915811	-3.87075394992930
C	-4.03390593869471	-3.31784399510724	-2.71827850803689
C	-3.49953030574456	-5.71695449220392	-3.38698514558812
C	-4.24498996333471	-6.77326414265790	-3.71265335948898
O	4.74927246151620	-2.74303170095944	-0.15208484846246
C	4.89971073263406	-3.14703441025338	-1.49668435320503
N	-0.10527167573399	-1.18165528702832	-2.44431592329955
C	1.14601783033682	-0.66916901428291	-2.43941038956966
C	2.44091484392716	-1.04635756105537	-3.03027999896728
O	2.84011451016517	-1.88918886365721	-3.79352905664277
C	1.68013916663958	0.42019566715671	-1.75984152228894
C	3.06616481334683	0.03887947924406	-2.14630183686047
O	4.19181715958039	0.32993867508309	-1.81063020249307
N	1.09339223624504	1.31040988033081	-0.92795330857971
C	1.75253921762390	1.86040394354602	0.16798301585627
C	2.84909758780345	1.18424866868494	0.70092514266538
C	3.60147199054199	1.74304826926093	1.73086277943036
C	3.18818602502990	2.92949822949631	2.33453166058928
C	2.03376031183725	3.55284633130504	1.85114102913557
C	1.32012357325484	3.04931986535250	0.76838532894817
C	4.85801062999864	1.05221577507947	2.17771400724670
O	5.26568808783566	1.10764977282378	3.32098339470877
C	1.57718008408877	4.81988763163807	2.52090225349139
F	1.37843378287994	4.63759095389953	3.83263454085829
N	5.48670185522970	0.38524453739109	1.15799238027273
C	6.62517285112703	-0.41858683109473	1.22756905288263
C	7.24798306613859	-0.79196691260606	2.42162683426855
C	8.36229621373791	-1.63664851882814	2.37295495103599
C	8.87466718310402	-2.11282779849833	1.17533662574684
C	8.24091031645234	-1.72450770395798	-0.00982105458961
C	7.13603826165528	-0.89149333610839	0.00534125708922
C	9.01085234244450	-2.01095828536143	3.67946911514983
F	9.95701825052826	-2.93710992617560	3.52448758388280
C	8.76020843613994	-2.26636772891309	-1.31381047827578
F	8.51986916311739	-3.58028157808762	-1.42372991591420
F	2.48560962685341	5.79341236363349	2.40372632391238
F	0.43202548044469	5.28035744769901	2.01365360933895
F	8.11788691781917	-2.49308399227048	4.54784652036299

F	9.58270774542688	-0.95436814721701	4.26522491301508
F	8.19360621056395	-1.67691463348359	-2.36632703545366
F	10.08039816970724	-2.10940157108129	-1.42345770065712
O	-1.86452310475835	-0.02080183796527	-0.84836022342389
C	-2.27887709177106	1.01851690502494	-0.38401361479671
N	-3.49158568611448	2.50829103538943	0.82196440597368
C	-2.53989928824776	3.11131617167239	0.03308175291880
N	-1.78469327195164	2.27332234893656	-0.64025575789969
C	-4.88213437934725	1.76579403676034	-1.47960914863347
C	-4.07751677248686	1.78219525431593	-2.62260080388250
C	-4.18357387787788	2.81459078843875	-3.55697764602479
C	-5.08935043408784	3.85383608412759	-3.35042631122414
C	-5.89618540286387	3.84931951622270	-2.21003249212788
C	-5.79556727702009	2.81072491493255	-1.28767575457316
C	-3.32927100165501	0.19995717452053	1.75566827576571
C	-2.27233137167470	0.71560337422023	2.70333848921997
C	-2.64794684673388	1.23509430839579	3.94805651081838
C	-1.69000192703715	1.72727876798467	4.83633584068351
C	-0.33837557299142	1.69991774775730	4.49215632127747
C	0.04885276231569	1.17000933568778	3.25929062373637
C	-0.90986304426219	0.68653730832342	2.36908559011590
S	-2.48191817630055	4.83294792239128	-0.19534671438326
C	-1.62645859818947	4.93232797228461	-1.81590911776740
C	-0.12425160752795	4.97485206895626	-1.75241211218173
C	0.53424986382799	5.96633099409358	-1.01201458867673
C	1.92543124952438	6.00548585954535	-0.96558053962540
C	2.67679498322438	5.05693253380886	-1.66665464888153
C	2.02890750146537	4.07528910286999	-2.41434148398294
C	0.63289755083868	4.03470663666075	-2.45621523922696
C	-4.35339676884724	3.06400567433456	1.78443676765713
C	-3.88984345428349	4.28479334776890	2.49805113023802
C	-4.84864665255174	5.24197538958769	2.85175263098493
C	-4.46659799312442	6.36378555449523	3.58094780079966
C	-3.13664680277531	6.51119140627091	3.98467173218824
C	-2.18595961485147	5.54357369298183	3.65524141610433
C	-2.55550445435539	4.43142926393670	2.90183664474478
C	-4.79711980681034	-0.76619028775753	-0.95419157440025
S	-5.33720165035627	-1.89800245955798	0.39612563656565
C	-6.46294468337792	-2.93634858061643	-0.51025523787959
C	-6.94311512999634	-2.38375130017818	-1.67988778758355
S	-6.34010213606535	-0.73897678336253	-2.00139180842992
C	-7.80372562766088	-3.08153071772783	-2.51805479141677
C	-8.17593909649449	-4.36883699890504	-2.12557844576405
C	-7.68693179955337	-4.93142423875556	-0.94013816562371
C	-6.81266770705896	-4.22187618746068	-0.11492791986127
O	-6.09321669184942	-1.17291982586520	1.42107017856277
O	-4.24791052683965	-2.76703203217084	0.85495078490195
O	-6.03050737192553	-0.62857771115697	-3.42914069760729
O	-7.32334457656535	0.19091043209925	-1.44718570832372
O	-5.39128686451057	2.50238127736877	2.04181326117348

H	-3.60485747971462	-1.51689978512572	-1.65198075421456
H	-8.15411967345700	-2.64183113088865	-3.45324970845360
H	-8.84701636633310	-4.94913671812740	-2.76001313996630
H	-7.98169427822095	-5.94426114040483	-0.66285034025208
H	-6.40188095284833	-4.65841492225051	0.79677442668497
H	-5.60852039505751	0.82153893527693	0.27603180735819
H	-3.34522857701870	0.99118812645674	-2.78435360734857
H	-3.54731416218630	2.80628901270707	-4.44367473727719
H	-5.17039596194898	4.66391119285551	-4.07722248180395
H	-6.61149803371473	4.65569556803163	-2.04069182307426
H	-6.43625741481512	2.79853934396419	-0.40317758001261
H	0.11996166615271	1.58628683017396	-1.08349440820686
H	3.10445159078653	0.19907686229558	0.31579041825742
H	3.75947984884325	3.35254929924823	3.16293868472833
H	0.43907258414822	3.56955397683143	0.38971124846122
H	5.11025905396053	0.51365592606709	0.21856714136714
H	6.86673026956624	-0.43279090075588	3.37492830963500
H	9.74423442425924	-2.77041096431901	1.15749105070115
H	6.64060464747649	-0.60881238840399	-0.92622057703035
H	0.43377326322882	-3.19332755949216	-2.34782827045721
H	-2.13595648947634	-2.65049689761074	2.39919125489183
H	-2.31561414133879	-2.70035405490373	-0.04658722504800
H	2.37646491884956	-2.92104795676755	-1.43595079689577
H	4.42898709915177	-2.18385224635297	2.27215459213108
H	2.22606119688618	-2.02856824811093	3.46863181572098
H	-1.91276962673400	-3.96554755119236	-1.81585107466621
H	-3.81969874167928	-0.77600764849672	-3.68267130055930
H	-2.03925136893331	-0.82335196263527	-3.73405515403705
H	-0.62959794932185	-3.00627026134833	-4.38417473219897
H	-0.89433412132066	-4.65499226654538	-3.79008159837367
H	-4.04465681913450	-2.23840542807840	-5.47441634401738
H	-2.34245348976710	-1.85751325681609	-5.80751919077098
H	-2.49659400192178	-4.30783079561910	-5.63302755374253
H	-4.93537994530898	-2.75101689576819	-2.95731954424971
H	-4.17142634911429	-3.82394248964499	-1.75070571302443
H	-4.68376961236268	-4.35490624507250	-4.48945862319876
H	-2.64122588324560	-5.85953001790902	-2.71922449469674
H	-4.01343220788323	-7.77191065705117	-3.33646531037182
H	-5.11404277583031	-6.66915349279516	-4.36954786554836
H	-0.75159264989044	-0.54160893094207	-1.97352235874711
H	4.32052433946368	-4.06170772689928	-1.70083413886110
H	4.58536591386413	-2.35482382771564	-2.19145218071835
H	5.96568877172733	-3.35655257380194	-1.63878062026891
H	-1.99190739138202	4.10194960722805	-2.43168073878907
H	-2.03352938064889	5.87200250325798	-2.21827673181315
H	0.12345823035882	3.25694453862783	-3.03120403753175
H	2.60992940615281	3.32449544587880	-2.95459476224596
H	3.76703503929118	5.08212522183409	-1.62368293548974
H	2.42707202246409	6.77307531249202	-0.37388735914044
H	-0.05098753293648	6.69963414127730	-0.45121765175886

H	-1.80941830021102	3.67081667781981	2.65136330717796
H	-1.15190528543813	5.65226913175058	3.98263635155531
H	-2.83996590922687	7.38544831776193	4.56661184560893
H	-5.20640762777283	7.12138387077971	3.84256672843597
H	-5.88560551885098	5.09906355776778	2.54296129879144
H	-3.05985105803435	-0.80424007098411	1.39956930644591
H	-4.29489060607901	0.12423408534657	2.26860698463065
H	-3.70688032682478	1.25860245587669	4.21849728364469
H	-2.00303114123813	2.13462228597933	5.79926101211011
H	0.41481679182440	2.08228653042520	5.18290495833353
H	1.10655385618377	1.11737958380813	2.99104296642616
H	-0.58454843118836	0.24570934540889	1.42112274271144

169

C2・15aa DZ=-6232.26519075307,TZ=-6238.445956589882,FreeE. Cor.=1.19882866

C	-3.61874783215840	1.21417378258583	0.60636593123817
C	-4.96682577238831	0.97396924090839	-0.17671830686896
C	2.35423242953761	-2.81798637469029	-0.31750095893490
C	1.05330806645425	-2.73861847929394	0.26794714060262
C	0.94885575808488	-2.51848829499708	1.67517853944263
C	2.13558353418591	-2.27377057454808	2.42934181731740
C	3.36766373271894	-2.31248293323388	1.84098808908128
C	3.48523477695954	-2.62166425131688	0.45421012513389
N	-0.22868843898134	-2.54532432561587	2.35165622247347
C	-1.32255826131546	-2.76844090211523	1.66638196611911
C	-1.34776149288193	-2.91825861077605	0.25858247180922
C	-0.17555321817032	-2.88395735862316	-0.46415369300729
C	-0.21624469106600	-2.81877456163369	-1.99376694472716
C	-1.43476210266203	-3.49699097577322	-2.63598511495884
N	-2.70085733071367	-2.73703540432051	-2.55944496910043
C	-2.85801054034214	-1.77366685704572	-3.66483455933546
C	-2.95723142371344	-2.49144932280408	-5.03460421277337
C	-2.53590760784664	-3.94903852709095	-4.81966381732181
C	-1.18099041435102	-3.93613202430161	-4.09240482629200
C	-3.62295288608608	-4.61624194197210	-3.94187723564225
C	-3.78369749926156	-3.71804518269419	-2.66933664347217
C	-3.32313457504486	-6.04935937284614	-3.60588627067052
C	-4.09569862399958	-7.08154510890768	-3.94867683118831
O	4.75040915806839	-2.71172078161518	0.00435786948226
C	4.97185690006336	-3.09981355883611	-1.33523551763036
N	-0.14126126158301	-1.38570197429042	-2.33525202486345
C	1.05701608180493	-0.78788733837553	-2.35343720533922
C	2.36745209308776	-1.13328980783663	-2.93986634781760
O	2.78908154315828	-1.99305675961879	-3.67264340714793
C	1.56283839561623	0.34379099080796	-1.71598761733737
C	2.95245070200524	0.00410108402543	-2.10542649851193
O	4.07288590098136	0.35306974156968	-1.79837362062148
N	0.96781249829400	1.23369955368801	-0.88350732504581
C	1.64501826147525	1.80577981964005	0.18847331423198
C	2.75085473519043	1.13937161065954	0.71760071424371
C	3.51826362080438	1.71597733889357	1.72621000850866

C	3.11889702778520	2.91604526694465	2.31168631220354
C	1.96344338781017	3.53651639051206	1.82795690226330
C	1.23095329535419	3.01164332817920	0.76844200953735
C	4.78509419424137	1.03905336990660	2.16447046835329
O	5.20825875561925	1.11110980481325	3.30128674415350
C	1.53198894848882	4.82829669296598	2.46580653344135
F	1.26395122533266	4.66698059458591	3.76934746985061
N	5.40797081299311	0.36973876229826	1.14301709661855
C	6.57799728820626	-0.38778176607883	1.20235158436451
C	7.19952964854721	-0.77359190948151	2.39335711353072
C	8.34922202111553	-1.56815731840999	2.33528678200387
C	8.89904799762707	-1.98211782583805	1.13083212856524
C	8.26604788114573	-1.58247967121340	-0.05058807036790
C	7.12546521078746	-0.79868395623607	-0.02588196382894
C	8.99927846965323	-1.95513042277495	3.63745971006352
F	9.94152803945131	-2.88367695539130	3.47209711343909
C	8.82612647574289	-2.05915117055141	-1.36321634708759
F	8.62891570923415	-3.37491100992134	-1.52447818534541
F	2.48525695887553	5.76124275697864	2.38441611178979
F	0.43282282617184	5.33353472225056	1.90318085526745
F	8.10708731765773	-2.44018432387217	4.50467791657169
F	9.57837578688690	-0.90602149739319	4.22979561996473
F	8.26016649782987	-1.44925613139127	-2.40396841972713
F	10.14258302861412	-1.85773950749207	-1.44156122886036
O	-2.10696748163850	0.00982901263375	-0.83785687619988
C	-2.44909416370883	1.07220945146556	-0.36480285448626
N	-3.48139265046500	2.64245126762069	0.92832736314779
C	-2.55077853099052	3.18325595611653	0.06855455540131
N	-1.89590722533240	2.29817013908211	-0.64687524349477
C	-5.13595101737453	2.06122877767241	-1.23324699246830
C	-4.41821241375492	2.03585066769990	-2.43432046615497
C	-4.52404900817081	3.08975864495069	-3.34283601308957
C	-5.34686284102855	4.17933261039571	-3.05801799506108
C	-6.07826506067519	4.20392990811856	-1.86877137294833
C	-5.97562209635249	3.14876893846914	-0.96462933280641
C	-3.42574225037632	0.30616222415346	1.83042019516413
C	-2.32024658406867	0.77284482305826	2.74757244536999
C	-2.63091890178582	1.34748683992688	3.98586607124847
C	-1.62003114494392	1.79444241334321	4.83838907973026
C	-0.28226407262673	1.66850707884156	4.46269945721110
C	0.03836599557831	1.08439596656154	3.23551028925643
C	-0.97228071409613	0.64165110750860	2.38148656100988
S	-2.40589824416362	4.89516161046823	-0.17676278768069
C	-1.59367559697584	4.93861954984795	-1.82315597049168
C	-0.08965260613517	4.91437894111037	-1.79606389411882
C	0.62773039940675	5.89372050000680	-1.09490293987552
C	2.01956479116346	5.86761241715197	-1.07126562791603
C	2.71300022639486	4.86547222259790	-1.75762357317095
C	2.00676063773540	3.89812196642322	-2.47003031142352
C	0.60965100447498	3.92206885245736	-2.48781998254548

C	-4.27934135236720	3.24909464981920	1.91355827016695
C	-3.76472433931210	4.48244271449410	2.56462305037370
C	-4.68701201689439	5.48610476801445	2.88524966367426
C	-4.26005440699809	6.62184440192572	3.56595982007993
C	-2.92330246746951	6.73527803117619	3.95803436761020
C	-2.01047637621605	5.72194731337064	3.66185217107985
C	-2.42317125404309	4.59615781062744	2.95167666604696
C	-5.10062248266136	-0.43850661549636	-0.77693501200874
S	-5.52241184920261	-1.74178723126435	0.46667912892351
C	-6.49017871149794	-2.78775113845852	-0.59414288979821
C	-6.95114646846247	-2.19790528755819	-1.75844969187807
S	-6.54289123012647	-0.48043972400757	-1.94762330331640
C	-7.63450656996655	-2.92846474012632	-2.72311141036489
C	-7.85394023924014	-4.28325874547758	-2.46866744097554
C	-7.38713829063857	-4.88043939119728	-1.29181974998264
C	-6.68691326629192	-4.13968866292865	-0.33851614418711
O	-6.34918332683335	-1.12074291836932	1.49268892197516
O	-4.35113964517403	-2.51766125766361	0.85886752296682
O	-6.11885122560564	-0.26294774956345	-3.32325577787069
O	-7.59816511060391	0.33732138183995	-1.37000198350791
O	-5.31568198572640	2.71869482748939	2.23801221962933
H	-4.22069949214783	-0.82566391069774	-1.32298576703731
H	-7.96464125738055	-2.45791986001776	-3.65044574093203
H	-8.38186380807016	-4.88768752362057	-3.20723482860601
H	-7.55518172362810	-5.94481958041433	-1.12374814685280
H	-6.28533125180537	-4.60291565207158	0.56411897535743
H	-5.77403089145070	1.08529268693897	0.55994646471640
H	-3.77257303685886	1.18736558575603	-2.67592091365797
H	-3.95850914059477	3.05585101752708	-4.27540821649470
H	-5.42614727006933	5.00498214506345	-3.76716117484270
H	-6.73294048947239	5.04725669942924	-1.64410184273117
H	-6.54981489903939	3.16255596809873	-0.03557191121051
H	0.00095516772901	1.52187217874987	-1.05174275456927
H	3.00352429701285	0.14825493167285	0.34600214062641
H	3.70663275554269	3.35615850746337	3.11940273625175
H	0.35218536528502	3.53346122848040	0.38885696818287
H	5.01866828439548	0.48757043070789	0.20701497920034
H	6.78914097219542	-0.46393361197935	3.35184744478736
H	9.79603548279584	-2.60156839121250	1.10526385920032
H	6.63227208160743	-0.50619311289920	-0.95566041928754
H	0.67388345482901	-3.30879291545704	-2.41582638125681
H	-2.26816158151941	-2.80722409196008	2.21719192625300
H	-2.31061019651694	-3.00270703439778	-0.24263499662951
H	2.45848195029229	-3.08333983186010	-1.36790840772437
H	4.28393813780034	-2.14908827517157	2.41217235775146
H	2.02101252092619	-2.07656706678744	3.49624864179147
H	-1.59963686486723	-4.40244082558485	-2.03242699518157
H	-3.77127618649586	-1.19210125640218	-3.46877497182959
H	-2.01674153640431	-1.07397721609655	-3.65251619170339
H	-0.50497353135302	-3.22182420439309	-4.59323183777370

H	-0.68799727716940	-4.91935457794558	-4.11112444143274
H	-3.98437096311997	-2.45156440999891	-5.42914080002119
H	-2.29955196694437	-2.01013642552404	-5.77304046582035
H	-2.45783847203394	-4.48079633785084	-5.77847874532016
H	-4.73273649012876	-3.16512698703090	-2.72552418443200
H	-3.81056315179228	-4.33187887148381	-1.75352598919057
H	-4.56700329922760	-4.59641925526793	-4.50929674523369
H	-2.41064183815950	-6.24203374724836	-3.02701787163902
H	-3.83718728458938	-8.10598517175326	-3.67203300537991
H	-5.01598403657793	-6.93024204705572	-4.52160046178555
H	-0.85175886230377	-0.84911214511115	-1.82745621617823
H	4.47957775281502	-4.06071608922530	-1.55427829670818
H	4.60696999029665	-2.33624149098027	-2.03841686155075
H	6.05512521924782	-3.21670154538217	-1.44989112615217
H	-2.01218795837988	4.11798326522357	-2.41787448705334
H	-1.96899380239707	5.89177946219146	-2.22439470870966
H	0.05453520078639	3.15518242127242	-3.03417189704067
H	2.54311799672975	3.10887186206675	-3.00152245722209
H	3.80382483896553	4.83931725682853	-1.73302805977397
H	2.56725515001056	6.62583409617227	-0.50888698790329
H	0.08756225237097	6.66880267746363	-0.54524261470376
H	-1.70754191571873	3.80092127928959	2.72066210495114
H	-0.97222046081482	5.80584424249312	3.98188997398862
H	-2.59112699503968	7.61958047151209	4.50451259635601
H	-4.97008011357847	7.41592940176836	3.80046481940719
H	-5.73078281493203	5.36802149166636	2.58898407006789
H	-3.17951304136914	-0.69141863844454	1.44048601448579
H	-4.37362202483006	0.23544549467238	2.37976692487026
H	-3.67859216930447	1.44659549144309	4.28127766468787
H	-1.88061561520069	2.24409068799688	5.79812191467233
H	0.51169873336395	2.01739192434126	5.12503433686568
H	1.08389393085746	0.96378481679891	2.94229314593647
H	-0.70414401339046	0.15804336005226	1.43610842900409

169

TS2-B DZ=-6232.21956599110,TZ=-6238.407607789930,Free E. Corr.=1.19864178

C	-2.61756733726848	1.15697547216339	1.65633990842524
C	-4.90573467585897	0.37662466689090	1.16926796893027
C	1.13203516352218	-3.27432493655396	1.31635173554068
C	-0.23303564815886	-3.68041961890185	1.27986067012447
C	-0.89896227651275	-3.98097900419914	2.50215696519447
C	-0.20106408180034	-3.79901721776912	3.73219316586705
C	1.10142994888988	-3.38599486924510	3.74795887203142
C	1.78911920557109	-3.14541517350961	2.52392019699725
N	-2.17151049987279	-4.45765589662952	2.55586104289741
C	-2.80216151994048	-4.65874462103894	1.42871170042550
C	-2.24610378356082	-4.36408473266389	0.15791583724357
C	-0.98045115149788	-3.83494822424730	0.06488931838707
C	-0.37245551279402	-3.35333239960807	-1.24806396654446
C	-1.15680294985695	-3.73279085910823	-2.50778786190899
N	-2.47097807645235	-3.01279965384602	-2.68049193947955

C	-2.34882895745363	-1.64137029765299	-3.28347922423596
C	-1.84914030833007	-1.77317107441464	-4.73008951486931
C	-1.35221758208053	-3.20706443153335	-4.94119708987711
C	-0.36803534052186	-3.54088123303797	-3.81017397792871
C	-2.58222842503912	-4.14318821069592	-4.89065817405301
C	-3.34328593109234	-3.84644024835445	-3.57219664119722
C	-2.22187095464179	-5.59714401028385	-5.04300052103344
C	-2.55185352023546	-6.33440628396244	-6.10279741209400
O	3.08401857131251	-2.79817646042713	2.65409336579906
C	3.83356789786851	-2.53824913359084	1.48669020356482
N	-0.13706277090963	-1.91869177737319	-1.13386646778644
C	1.05228014093506	-1.38382547645731	-1.33487364800095
C	2.36258956034716	-1.91957500348376	-1.76759423151549
O	2.76548818139297	-3.00102138814823	-2.13170657264410
C	1.61586148629427	-0.12984056416287	-1.03740678788317
C	2.94491430903587	-0.54369955206820	-1.52416827322659
O	4.06129579218163	-0.06103053147513	-1.60308147855889
N	1.06428708151739	1.00586858476518	-0.55305626432551
C	1.83006796740442	2.00040256656326	0.04604494003942
C	3.10110855026233	1.67366746691882	0.52006000199489
C	3.97045575891933	2.64409767533056	0.99967276015851
C	3.54045701295487	3.96339971398782	1.13592179387504
C	2.23613881662492	4.26887268253178	0.74579037755694
C	1.37691535638911	3.31545347690736	0.19689209420781
C	5.35313972511423	2.22360181137500	1.40372916143830
O	5.97018575850994	2.76083402787400	2.30255196945776
C	1.73441674680967	5.67377778123747	0.92249660048043
F	1.19656927617722	5.85476353246300	2.13995709960186
N	5.81457742641883	1.16350107602822	0.66449102428827
C	6.86844297887662	0.30023119790195	0.96264019501839
C	7.84242984744035	0.55951735616334	1.93404838303045
C	8.81891518760194	-0.40471650008839	2.18877699917264
C	8.85504806961923	-1.61895365154974	1.51117030520393
C	7.88125655280789	-1.85595132742954	0.54036072540211
C	6.90222017636211	-0.91373598617807	0.25756402480904
C	9.87528321067943	-0.07873041382205	3.21180931296770
F	10.56874120319411	-1.15583396323063	3.58069795641529
C	7.87954868834203	-3.14641131793243	-0.23529336001899
F	6.63523263394043	-3.58368693672839	-0.45320692468432
F	2.70587379034061	6.57715712852821	0.79743918837758
F	0.78215673583555	5.98167859656510	0.03612784509557
F	9.34497801127024	0.45430956246758	4.31521767908974
F	10.75228740432591	0.81171937679112	2.73773046824824
F	8.45289472322978	-3.00413777003999	-1.43351116129314
F	8.53886414013725	-4.11277812323620	0.40352547773721
O	-1.93785489571269	-0.75065827476688	0.37494507978981
C	-2.13223967081653	0.48327977229764	0.51905983885604
N	-2.67075179618874	2.52832308629651	1.27507208762268
C	-2.26473178161068	2.58254030435776	-0.05205602398815
N	-1.92890301887822	1.41054245295078	-0.51338009816648

C	-4.95814113765176	-0.39827719130049	2.40991994388338
C	-4.41508287361728	-1.69116937578637	2.50121877457533
C	-4.45555747046988	-2.38145401378551	3.70875137418048
C	-5.04390737209932	-1.79361813619088	4.83286162477335
C	-5.57147064585483	-0.50397113852399	4.75351404492452
C	-5.51541798611402	0.19834488711225	3.55122053785481
C	-2.23991339256823	0.77622283981864	3.05777394558063
C	-0.77033447027402	1.06613604612169	3.28661685140755
C	-0.33838598784906	2.32829111506263	3.71031788749927
C	1.02399353301205	2.60415242541354	3.86158355193682
C	1.97076161154176	1.61358238568824	3.60163955294158
C	1.55059693838185	0.35011451460811	3.17415742402891
C	0.19170753837142	0.08213752409569	3.01052583716963
S	-2.26458386548441	4.04471123731767	-1.02347879546990
C	-2.05227471963641	3.30789044727766	-2.67956519042005
C	-0.66565201842111	2.81647526517651	-3.01293756273098
C	0.46151800341899	3.63016735512584	-2.86395954128826
C	1.72985006045970	3.15395836372628	-3.20219001934539
C	1.88472380573055	1.86393806686294	-3.71048830223106
C	0.76068674780510	1.05158888940672	-3.88307913332240
C	-0.50198739536124	1.52452506409944	-3.52886189869024
C	-3.45210952236675	3.45740448836888	1.98719563646506
C	-3.20585298818430	4.90855777212840	1.77058810432314
C	-4.32122075651814	5.75052461429172	1.68324495076620
C	-4.14024355738689	7.12290547915359	1.54582026768455
C	-2.84791579050694	7.65514008610396	1.53528287468580
C	-1.73822172805849	6.81806414664076	1.65406973758374
C	-1.91125836117192	5.43795519961971	1.75844384178990
C	-5.05502616032979	-0.15901113906115	-0.09280643952748
S	-5.66179149876354	-1.75092763794625	-0.51042492787582
C	-6.77809224810066	-1.19490204447276	-1.79472493216867
C	-6.63428921211303	0.12676722550502	-2.19515033328900
S	-5.21651207702805	0.92848202787207	-1.46985855610506
C	-7.45766232521893	0.68491243678390	-3.16349913399204
C	-8.45203324330543	-0.12715729871159	-3.71419170566743
C	-8.59838548433957	-1.45864388521974	-3.31100940899968
C	-7.76076138840160	-2.01175570237557	-2.33961887896783
O	-6.45366328041978	-2.36289283785889	0.54738518964658
O	-4.63611504063777	-2.60615247257492	-1.13163073122248
O	-4.14894288344986	0.76597259266513	-2.46019560225353
O	-5.49841565487611	2.28240862671151	-1.00134738004824
O	-4.28014046304159	3.06331649241441	2.77696202392045
H	-2.97222277907495	-2.88508851386000	-1.78469171743842
H	-7.33261090135476	1.72481852736791	-3.47034579066536
H	-9.12456129494159	0.28552349055341	-4.46723677443984
H	-9.38362463579120	-2.07295316412572	-3.75319275408839
H	-7.87822964443155	-3.04428029000785	-2.00591482530644
H	-5.07756725941798	1.45187887395277	1.25790786206875
H	-3.91223523097381	-2.12758939136879	1.63380115657027
H	-4.00312294933048	-3.37213360829895	3.77467463010556

H	-5.07566633706346	-2.33936034401122	5.77747127298140
H	-6.01701700810396	-0.03801831254282	5.63376400628603
H	-5.89316995249788	1.21975258424037	3.48682754577083
H	0.05490505488394	1.15455762098028	-0.68126588755526
H	3.40386360343513	0.63208210988186	0.53997075817848
H	4.21227188942690	4.72767086436703	1.52836806890794
H	0.37795155078189	3.60181453697911	-0.13861370838290
H	5.24386006167207	0.87782453704880	-0.13286600945377
H	7.82564766283048	1.49618160408835	2.48710285944102
H	9.61499108333128	-2.36642406957100	1.73715993172971
H	6.13167087019657	-1.11631094183185	-0.49251347457072
H	0.60207789895602	-3.85129032041030	-1.38399894551187
H	-3.82434204603071	-5.04844044878129	1.48249878482158
H	-2.86963199923184	-4.56318990052928	-0.71190244954051
H	1.67520920612219	-3.11236748676236	0.38960837959908
H	1.65037699393272	-3.25099939618407	4.68089194814565
H	-0.73932365969173	-4.01381427074477	4.65621868619554
H	-1.42704132263164	-4.79161921122902	-2.39760884894861
H	-3.34404646099634	-1.19179339830616	-3.19908810933617
H	-1.67543049733676	-1.06504464327557	-2.64256607671943
H	0.35501174019436	-2.71584528302900	-3.70344613404757
H	0.20770657176320	-4.45345344080032	-4.01416634110846
H	-2.65510283049591	-1.54347824855983	-5.44067247083360
H	-1.03339950487652	-1.06070344026286	-4.91092437319600
H	-0.86089978024841	-3.30537876141368	-5.91742558384639
H	-4.25983886841526	-3.26812877203239	-3.73686045510222
H	-3.60378626487651	-4.76070222086857	-3.02167904799406
H	-3.23912631370850	-3.86793028966728	-5.72825631559629
H	-1.64945906811627	-6.05956024961054	-4.22948022968646
H	-2.25870418565018	-7.38337759151452	-6.17942436179882
H	-3.12736399082737	-5.90785192310254	-6.92983191350563
H	-0.86245505386957	-1.36291573293688	-0.58792992709321
H	3.85005062532773	-3.41045336641863	0.81410535362030
H	3.41685066804064	-1.67080578791491	0.94715110916566
H	4.85468966874535	-2.30875288093849	1.81049424174181
H	-2.79518052147013	2.50612662341092	-2.77452671658853
H	-2.33740874849165	4.14049315533972	-3.34006635286157
H	-1.38770654626044	0.89579897044027	-3.64519701385154
H	0.87559330233623	0.03797279042342	-4.27630464062886
H	2.87781272120209	1.48921830504932	-3.96492363172179
H	2.60272022910801	3.79464368684563	-3.06117620901072
H	0.35013170095139	4.63795586171470	-2.45459178379389
H	-1.04800688636543	4.77314332819478	1.84768290302030
H	-0.73397819781353	7.23947644887685	1.66471144951635
H	-2.70489541436339	8.73294659807968	1.44119956830277
H	-5.00547681170180	7.78106252993739	1.45552531297387
H	-5.32186483449584	5.31600016189075	1.71189790274580
H	-2.41773390893587	-0.30114406192665	3.17362456262786
H	-2.86991896308684	1.30224113378830	3.78950449028270
H	-1.07801357890937	3.10258196782801	3.93620873883182

H 1.34431234398519 3.59556581755800 4.18821567139592
H 3.03481256614979 1.82635225775734 3.72394386482942
H 2.28654665593199 -0.43330687885359 2.97439308363098
H -0.13558727789699 -0.90375370617935 2.66612050067983
169
TS2-C DZ=-6232.210389519358, TZ=-6238.405361016456,FreeE.Cor.=1.19882982
C 3.49197298128337 -1.24683347959899 -2.18467170980759
H 0.27286474100195 3.12012957538895 -2.03634497350113
N 0.83483475147613 3.97995244660973 -2.05972309830734
C 4.61584454648047 -4.75775721723642 2.66573278765433
C 4.41456572646340 -4.09216188384723 1.44988590927015
C 3.96159368110747 -4.80018734416186 0.33442182433748
C 3.66841118274844 -6.15957083491226 0.44825555878043
C 3.83043312006259 -6.81184126502692 1.67098978099197
C 4.31092858528308 -6.11081058534148 2.77952170383122
C 4.82039790664043 -2.65293878246000 1.39524429005488
O 5.90263955008490 -2.30270049349473 1.79934498637191
N 3.89924263172515 -1.69923583444426 0.92119605583665
C 4.22749345110568 -0.35403696768631 0.75108759575098
N 3.17774860124318 0.41188419981983 0.67914858242821
C 2.07068858793449 -0.41525821558595 0.80386748134911
C 2.48105093177451 -1.75537660989264 0.85406493119343
S 5.88088042309411 0.23122365148694 0.65312679545297
C 5.53393870658177 2.00366744456709 0.36389402110176
C 6.71439822237168 2.64747367603901 -0.31126417879888
C 7.07574558483408 2.25527125038902 -1.60756111711514
C 8.12243393099360 2.88717374094154 -2.27418662594818
C 8.83124664472466 3.91666672055467 -1.64660359297811
C 8.48838732995432 4.30164565717525 -0.35075628231107
C 7.43575744816842 3.66753988416460 0.31461214333510
C 1.56606711955698 -2.88690082980672 1.16570364205312
C 1.56252915208859 -3.38944361423230 2.59638182379173
C 2.05209333243083 -2.61313122762001 3.65261560782665
C 2.03901876512524 -3.10507196628002 4.95887167313458
C 1.53012563852740 -4.37745943078029 5.22498724414594
C 1.02696411193385 -5.15169758777609 4.17753287764800
C 1.04560426320111 -4.65984331321372 2.87329978411463
O 0.88662425196792 0.04584228284867 0.85233506038390
O 3.75218217549315 1.43335429191249 -2.20732519725640
S 3.63396238672360 0.26199229673057 -3.08375584993394
S 4.91783797121618 -2.29718492408868 -2.36227567357984
C 5.83775028325489 -1.24615523175098 -3.45955977409706
C 5.22241950023414 -0.05797331642286 -3.81336406172908
C 7.11160402518669 -1.56560876584821 -3.91909679067146
C 7.74220819036426 -0.65562319251372 -4.76698546933465
C 7.11199788338073 0.53898722417325 -5.13855769260242
C 5.84119208167172 0.85651436936841 -4.66040784870377
C 2.33540108717472 -1.82117860371163 -1.72219016246028
C 0.94956556312595 -1.38230872030650 -1.87507777527259
C 0.55832678818351 -0.04315141199174 -2.04692305488888

C	-0.76767486104493	0.26196474086551	-2.33921389160425
C	-1.73115211724247	-0.74685427328136	-2.40431602097081
C	-1.36888060226564	-2.07122461891669	-2.15429507203752
C	-0.03784585936313	-2.38530263580462	-1.89378246150007
O	2.63777420473796	0.35567924742117	-4.14964677437774
O	4.49891368819387	-3.52409888277039	-3.03901876132277
O	5.70492871115090	-2.47278438758508	-1.14566706597810
C	1.55870021519678	4.07844047248310	-0.73999775584566
C	2.18806484157206	5.47672960736937	-0.65761393033397
C	2.23562975841374	6.08237915460141	-2.06731728392004
C	0.79833160740161	6.40476328609185	-2.49806057640941
C	-0.07340997922594	5.15063172838100	-2.30888724352622
C	0.58795394420659	3.76671119303969	0.40120562869466
N	-0.00035925620016	2.44358692309530	0.22345684591190
C	-1.31442172499001	2.24236971126235	0.28630387868201
C	-2.11415668779880	1.09417648230408	0.47565941568888
N	-1.76209480127540	-0.16792446781975	0.76122022944637
C	-2.65889075872537	-1.22161542600216	0.89944866457570
C	-2.31265424340221	-2.36599210341728	1.62665228877648
C	-3.25483655279385	-3.38322492740858	1.78682097598061
C	-4.55428325078260	-3.26383275533904	1.29775144105511
C	-4.89179624504969	-2.10996153267040	0.58590757118281
C	-3.93008330285137	-1.14078194629523	0.32549498793004
C	-2.83233924666014	-4.62631420240400	2.52391427992851
F	-2.12654528669605	-4.33471153641724	3.61878417881292
C	-6.31205529142396	-1.89768164999380	0.14198244618162
N	-6.64990733892430	-0.57285041692484	0.04113653002894
C	-7.85920166450004	-0.02219146666778	-0.38603932360875
C	-7.92632085381705	1.38282752538621	-0.44408175028493
C	-9.09312971274863	2.00502700812985	-0.85825820374365
C	-10.22035992075711	1.26517149269833	-1.22662190286431
C	-10.13824263093026	-0.11850276654336	-1.16781828563981
C	-8.97792745450931	-0.77768161644803	-0.75283352232533
C	-9.15776074897269	3.50521763786360	-0.96341256308135
F	-10.29157618892676	3.98349648699212	-0.44483394314941
C	-11.31833782754770	-0.96953678273296	-1.55550904486152
F	-11.69390687104979	-1.76995379011222	-0.55401575135778
C	1.24736810133086	3.83879408035392	1.77009103125200
C	0.45219055906587	4.09116574726451	2.93400212902453
C	1.11389229051842	4.04182141441865	4.19580422058268
N	2.43994764019731	3.77680157505297	4.33096192611034
C	3.13785444356347	3.55171222958214	3.24688487089482
C	2.58452446984865	3.56752312611103	1.94249550241819
C	-0.94322680237487	4.37550626674689	2.88921352419023
C	-1.64719046272522	4.58980117302608	4.05837476900947
C	-0.98336310087391	4.53902327367562	5.31852517449229
C	0.35571913773161	4.27656194675451	5.38020740833032
O	-2.96283121917313	4.85893021735184	4.11677637992301
C	-3.70600788486331	4.85889249626318	2.91627655339749
C	1.83310665292768	3.87996893155378	-3.17611659657106

C	2.82318214131980	5.06520783443950	-3.06581083489619
C	4.23366163402699	4.61219665266009	-2.75940133651249
C	5.14369877648782	5.30676605885270	-2.07469427707715
C	-2.48954361387463	3.09979323111097	0.08744396731069
C	-3.35618253846195	1.90377820018059	0.41019772666033
O	-4.55216505172337	1.72639207782716	0.55362826335032
O	-2.66136779808957	4.28084926260551	-0.14460933036153
F	-3.87438891189044	-5.36558639052983	2.90253681951910
F	-2.05402934832340	-5.40470466129644	1.76086101174771
O	-7.07415908561720	-2.82146286423464	-0.06547652840680
F	-8.14528275519158	4.10101938104012	-0.33480910443914
F	-9.12214201940064	3.90491960263333	-2.23901140012505
F	-12.37742062554533	-0.23763148067699	-1.89950195118028
F	-11.02899755771812	-1.76196320324970	-2.59211739791509
H	0.58227655376880	1.59298034553389	0.45589311600156
H	-0.73829908227763	-0.34232923805502	0.86470595617270
H	2.33167169517477	2.90837003274750	-3.08509136225876
H	1.25335059004715	3.90261413097217	-4.10621262875469
H	2.84701004899172	5.55075721331545	-4.05503157435968
H	2.83565819279233	7.00100468399463	-2.06529362965917
H	3.18812400756231	5.39710105422570	-0.21648854756541
H	1.58504041607656	6.12245872502147	0.00021164519567
H	2.32592729722819	3.28757872874184	-0.79629186265828
H	-0.65615518485204	4.89943290673396	-3.20264402086184
H	-0.77026285246858	5.21980594571150	-1.46484458470939
H	0.78434183930218	6.72515526276847	-3.54880422929352
H	0.39634699514272	7.22565187922113	-1.89057897563944
H	6.15714417908160	4.91959721130386	-1.94312731712974
H	4.51874361708423	3.63912112969529	-3.17605845033140
H	4.91953607082662	6.28037559886252	-1.62941943650602
H	-0.22218991642067	4.50837292931878	0.36336220794813
H	4.20426361211642	3.33600969398795	3.37275692426137
H	3.23555055384581	3.33860200565228	1.09936296351544
H	-1.45671635093403	4.44679032765796	1.93366726447197
H	0.88351598664005	4.23481772371271	6.33378263820050
H	-1.57601344048041	4.71645435646519	6.21694349963222
H	-3.31263882871187	5.59305800942624	2.19642354859372
H	-3.69852545742973	3.86179845717842	2.44684119688937
H	-4.73395795745814	5.12268351591233	3.18544027902193
H	-4.16315832576202	-0.29967418726495	-0.32101267083769
H	-5.95187167119019	0.11127888402925	0.33734513842320
H	-11.13671889208433	1.76102018974675	-1.54753576972110
H	-8.94275528197962	-1.86367870469306	-0.70948483364734
H	-5.30273197377800	-4.03581570326582	1.47683870101924
H	-1.32444096870828	-2.44820806318923	2.08615275330434
H	-7.05076867149858	1.97264328071558	-0.16158733910574
H	5.32682322393934	2.47992800420421	1.33057706066611
H	4.64379494832458	2.05682796868669	-0.27593478619103
H	6.51510363975548	1.45017244056483	-2.08666442044832
H	8.38678925412230	2.57855838145910	-3.28777851004427

H	9.65120440394795	4.41363649233327	-2.16774485623010
H	9.04079699967162	5.10072488034747	0.14675871214348
H	7.16297069723193	3.97616140853248	1.32646711235825
H	0.56587285922534	-2.48418958854983	0.92748383079926
H	3.87183689722479	-4.30791302713566	-0.63442001193894
H	0.65805153577453	-5.27063994880278	2.05384803636818
H	0.61999154085701	-6.14498935647792	4.37702812745483
H	1.52319414850692	-4.76320441069741	6.24590171982533
H	2.43129404156393	-2.49173742084635	5.77225356755544
H	2.45838179804836	-1.61876548748413	3.44865867930253
H	1.70236303000362	-3.74762309518898	0.49318921079431
H	4.99547355088939	-4.19963194223673	3.52356031990257
H	3.32145125344435	-6.71075857337031	-0.42705117066828
H	4.44820646627977	-6.62033883173648	3.73454766033372
H	3.59282032985058	-7.87357270463337	1.75738794584480
H	5.34676230708606	1.79132513709541	-4.93244720017750
H	7.62381763457175	1.23501396736077	-5.80439607424004
H	8.74062585300117	-0.87911022290835	-5.14461937678703
H	7.59369932006138	-2.49842224366917	-3.62093802904405
H	2.42875405661056	-2.88249438344764	-1.46975601632469
H	1.29129972620281	0.75952851231084	-1.94320910069559
H	-1.06788251727668	1.29823564986234	-2.50889690161237
H	-2.76983664313165	-0.49504058937443	-2.62825860480458
H	-2.12124981652815	-2.86155316518164	-2.17333373793404
H	0.25534806154353	-3.42461793128686	-1.72889377222317

169

TS2-D DZ=-6232.204593376208, TZ=-6238.398845316929,FreeE. Cor.=1.19765473

C	2.89547318716460	-1.20577085133393	-2.22957350795115
H	1.55079092933080	2.63707360074892	-1.87910910500461
N	1.74868447003846	3.63232959140085	-1.67660523261716
C	4.46896874776010	-4.83227254290744	1.89323997992884
C	4.67075553315516	-4.44305384088382	0.56610388945776
C	4.65154248947192	-5.39253331773562	-0.46129892021927
C	4.35907199444624	-6.72245696308584	-0.16165739997114
C	4.12525167926278	-7.10883048261718	1.16040499891649
C	4.19719364072640	-6.16753274463811	2.18850033393585
C	5.04340368361850	-3.02740634112977	0.25518299226378
O	6.13619446337024	-2.74065403379756	-0.16846638665191
N	4.11088398490128	-2.01642647522905	0.54961488584709
C	4.45423895739052	-0.66512569012587	0.58004714218882
N	3.45895093747938	0.09391152944535	0.94242122835090
C	2.36113115001648	-0.72680460413416	1.10352928606166
C	2.70631934846958	-2.06618533204521	0.78757061188767
S	6.06011963476663	-0.05873897111066	0.22092908322320
C	5.77260465427506	1.71423484537136	0.57214305767962
C	6.63193590807504	2.59412949575472	-0.30181308568302
C	6.67655607154687	2.41245595359976	-1.69173466452168
C	7.42128861607766	3.27224851359970	-2.49744225088269
C	8.13633498556217	4.32794512155912	-1.92619156219787
C	8.10172004237379	4.51365052125638	-0.54427724920180

C	7.35598204408643	3.65069634722748	0.26208233150385
C	1.88185493528458	-3.20129435222251	1.29762245833184
C	1.85148838067015	-3.39807953396466	2.80468260609161
C	2.50893569008013	-2.55711792592799	3.70755822300574
C	2.42349999724622	-2.78482666012495	5.08354689787277
C	1.67118292312735	-3.85096299749210	5.57590868838762
C	1.00271434088980	-4.69086774210518	4.68190088746027
C	1.09633089041998	-4.46510944072834	3.31000351038525
O	1.22887550003771	-0.28220306130561	1.44132524641675
O	3.72897238993933	1.30531097534396	-1.57375474260059
S	3.09754987554988	0.49032173889978	-2.61523868816623
S	3.69777006732476	-2.26833432244720	-3.38482781521427
C	4.59254930918487	-1.00617161500128	-4.26079895186141
C	4.26454810704765	0.30072748012912	-3.94272354666891
C	5.54349498322729	-1.30018302758762	-5.23155053941977
C	6.15973500422523	-0.23112019318792	-5.88177326383307
C	5.83107684224358	1.09096296605413	-5.55963437287326
C	4.88190228623156	1.37429820206007	-4.57762263156255
C	1.82892439433373	-1.57779444676368	-1.42242134938033
C	1.05660488249474	-2.82895544480490	-1.53487170435600
C	1.63801965356209	-4.09367514023610	-1.71494653215074
C	0.84142173203822	-5.23333121641155	-1.75554937557415
C	-0.54732381765486	-5.13108687289430	-1.62590438367745
C	-1.13692280111492	-3.87750295866989	-1.46729870903409
C	-0.33797914101786	-2.73531611923216	-1.41936395611805
O	1.82582616087592	1.06139039055179	-3.09886944641112
O	2.74430081280463	-2.88042905879472	-4.30601486245307
O	4.65737178456513	-3.16364664238974	-2.73631103170731
C	2.11802858169727	3.78243533764696	-0.22327733901736
C	2.63512167599559	5.22292609592695	-0.01654048007509
C	2.29662608755181	6.06983780246298	-1.25172481782879
C	0.80307227637521	5.90170487702930	-1.56840973686007
C	0.55843731238080	4.46345679643757	-2.05393099700689
C	0.92212534213189	3.45270710099354	0.67485688834174
N	0.41150485440217	2.11030769127611	0.41618873116098
C	-0.90805692942053	1.89699860286871	0.40179975236136
C	-1.71606926467378	0.75369189391002	0.55194169175558
N	-1.39345735513677	-0.50652619502853	0.89158702745156
C	-2.29516659271859	-1.56686660385100	0.92855438435876
C	-2.03127276091290	-2.70626395239995	1.69744627538632
C	-2.95707350915467	-3.75086621320263	1.70326590863954
C	-4.17071594458849	-3.66011501913526	1.02398054734381
C	-4.43147266332178	-2.50781703725554	0.27868387889140
C	-3.46883579056976	-1.51028821334347	0.17345764123642
C	-2.60599921304166	-5.01154826453262	2.44705723368229
F	-2.04685944869540	-4.75357038582712	3.63166834879109
C	-5.76737862265414	-2.34512215882793	-0.39102613570328
N	-6.13173099076487	-1.03470317699722	-0.56105574344584
C	-7.28355163426799	-0.53325631688647	-1.17084693700699
C	-7.41147688592441	0.86595092385116	-1.22713492649640

C	-8.52831194006501	1.43985549043673	-1.81701539633864
C	-9.54496646519208	0.65409832520409	-2.36364624021689
C	-9.40428154562498	-0.72600257493122	-2.30355663725855
C	-8.29274040222657	-1.33512926683030	-1.71751389466223
C	-8.62845671071233	2.93909046529816	-1.91423427158191
F	-9.89607934478840	3.35048203262579	-1.87234278031300
C	-10.48140653838031	-1.62480173105322	-2.85150145563178
F	-11.09205308376785	-2.30404929847925	-1.87587533768174
C	1.30226317075668	3.60036175651331	2.14064235426494
C	0.55585955609448	4.43733039105894	3.02568269203869
C	1.00168239667436	4.50361754875987	4.37995227083074
N	2.06680087491357	3.80288583508576	4.84702678121419
C	2.70948588953364	3.02223134000714	4.01262675689615
C	2.36688801565060	2.88700068649205	2.64582319918147
C	-0.58962352831237	5.18748298212535	2.62868751006875
C	-1.25323099592543	5.97723806586468	3.54572287194028
C	-0.79647331575982	6.05734512968116	4.89407795593670
C	0.29477591213831	5.33967026769927	5.29445657106671
O	-2.34633081918715	6.70755878331022	3.26407722602071
C	-2.84432943090783	6.67954150035579	1.94182101646495
C	2.91760691736441	4.03390256045423	-2.52689412978510
C	3.09803300240406	5.56603893450409	-2.46146781775264
C	4.56785829484265	5.90172901837283	-2.47562712801797
C	5.20517813600350	6.72260486557080	-1.64102242839558
C	-2.07112114506356	2.75789408420558	0.13622301888009
C	-2.95732631650967	1.54686602990064	0.35055634278125
O	-4.15410253606112	1.34465705010890	0.33392573298005
O	-2.23266628608567	3.93770078749198	-0.10194745851856
F	-3.67027320027663	-5.78295405325860	2.66392253305059
F	-1.72247773781922	-5.74770909169556	1.75698599276441
O	-6.45069967357258	-3.29819170527634	-0.71026237391948
F	-7.97408953632947	3.54768043108889	-0.92640042765766
F	-8.11351222639838	3.39328352351143	-3.06008480525107
F	-11.42180888760507	-0.94479767262627	-3.50728238231430
F	-9.98042389628342	-2.53053456638897	-3.69500517702842
H	0.96860397465663	1.30748610590780	0.77579646484720
H	-0.40185580880237	-0.66797950166717	1.16353704117831
H	3.79122235173998	3.50485663781345	-2.12649459921195
H	2.70763348503509	3.66759829368513	-3.53977501263652
H	2.65192374648473	6.00456766441363	-3.37024119589939
H	2.51841466715041	7.12677957787880	-1.05717726198104
H	3.71816548426072	5.20927848463572	0.16346347744744
H	2.15901610219869	5.66214594217808	0.87177755300084
H	2.90466726968419	3.03162851378135	-0.06476572495716
H	0.48011237563288	4.39468661025700	-3.14569059426542
H	-0.33939457724355	4.01088601526558	-1.61806141228359
H	0.49176851463107	6.60734865802714	-2.34927853444262
H	0.20294435987831	6.11655765718642	-0.67087829404303
H	6.27768252210119	6.89844265358206	-1.74642769852962
H	5.14241233635469	5.40449881148659	-3.26735886608314

H 4.69172108335845 7.24531395854851 -0.82960202547916
H 0.11753502141828 4.15706771627856 0.43996384717130
H 3.55787670667048 2.45485744125195 4.40907997035754
H 2.94084773745206 2.19840942934625 2.01895280130640
H -0.96930193867254 5.11683738122134 1.61154646442083
H 0.65515796135076 5.38061162416508 6.32315318312336
H -1.34747727556551 6.69700167737888 5.58490219473983
H -2.09711325371975 7.07165719882315 1.23268358409471
H -3.11710672992142 5.65720014198390 1.63749711722015
H -3.73230368015495 7.31968694416774 1.92868546042962
H -3.62088330434071 -0.67278738808809 -0.50137170060301
H -5.51863355914141 -0.32428710960272 -0.15950926200512
H -10.42347148072681 1.11066599373570 -2.81896230634394
H -8.21097205114482 -2.41863312074729 -1.67994030615206
H -4.91180622236341 -4.45793642295038 1.07375803639010
H -1.11119360758385 -2.77137640028051 2.28564991801787
H -6.62428053437981 1.49355344204530 -0.80164150802785
H 5.96650331184750 1.90566464101911 1.63580714109324
H 4.71066671372169 1.88840699564091 0.36541318218474
H 6.11801200324899 1.58495973937901 -2.13196444758689
H 7.44527623398656 3.11522926241001 -3.57826199215818
H 8.72090309384859 5.00044102650186 -2.55650044805002
H 8.65991634395725 5.33297488215707 -0.08754019097637
H 7.33407656967819 3.79851529851888 1.34433295597521
H 0.84827382805335 -2.97340898953014 0.98565833052024
H 4.84662052179946 -5.07275788756969 -1.48625534478942
H 0.57106490182899 -5.12307137295577 2.61200097433863
H 0.40127362523215 -5.52250360712904 5.05341517493722
H 1.60286978266366 -4.02564957239003 6.65091145573269
H 2.94850335216134 -2.12101985100760 5.77291531173879
H 3.10736705357312 -1.72031133151420 3.33996172165328
H 2.12283084259839 -4.15336335244208 0.80742068122935
H 4.51939644529125 -4.09288117482164 2.69399282367837
H 4.32031381611171 -7.46197841700398 -0.96310679627584
H 4.03610821795270 -6.46997313874000 3.22461764408888
H 3.89945452759007 -8.15119998771169 1.39136568533539
H 4.64398817729992 2.40447897434065 -4.30648311675427
H 6.32915045692565 1.91348672424360 -6.07442361708614
H 6.91155271793896 -0.42955393436447 -6.64646545003425
H 5.79784287962736 -2.33522389027370 -5.46613568360367
H 1.23711089819670 -0.74313982214448 -1.02505437897276
H 2.72196988984869 -4.19505053895372 -1.77612276592800
H 1.31042998044800 -6.21172178181476 -1.87411179137779
H -1.16659316689892 -6.02923379268991 -1.64430822052878
H -2.22105722162356 -3.78460374041694 -1.36659953628888
H -0.79590093839965 -1.75227081268603 -1.29509506414334
90
C2 DZ= -3013.472339012195 TZ= -3016.906994729501 FreeECorr= 0.62073253
C 0.29600487897103 0.23106756909925 -2.53816120132793
C -0.81675758546060 1.18590458433476 -2.49957008261684

C	-1.77517551139584	0.16535722128838	-2.57559186250843
C	-0.71425064982818	-0.87023215145390	-2.78836193738166
O	-0.66545374357561	-2.05203247776901	-3.00217241366769
N	-0.91674964598169	2.52305511619396	-2.26621688876475
C	0.05887288861445	3.19064861536405	-1.51852118007273
C	0.80549685541445	2.43916349843627	-0.60954567222510
C	1.88737050736451	2.98626553767189	0.06672999493549
C	2.15743110674433	4.35250982754362	-0.03821941316873
C	1.34910440183744	5.12190837935147	-0.87654800972501
C	0.32409666838759	4.55737815025401	-1.64265707501650
C	2.71460122875405	2.06052551505033	0.91386787581182
O	3.24907302693563	2.40149036716632	1.95032061337436
N	2.76140712080864	0.79279247316833	0.38786862710984
C	3.08162009568316	-0.40057703054440	1.03694320666031
C	2.69179831024271	-1.59375626048645	0.39977087280120
C	2.91566483832573	-2.81434098703520	1.01793340069047
C	3.52877908563381	-2.88860593449134	2.27181910879813
C	3.91803121142651	-1.70287215858649	2.88295712814314
C	3.70760508731283	-0.45752516393572	2.28536865717849
O	1.49742196003789	0.25119316463020	-2.33888397377217
C	-3.89170744647011	-1.00177047256492	-2.02490830939071
C	-3.12586781923679	-2.07666936926384	-1.27612057156003
C	-2.24803440837441	-1.80991691928385	-0.16744503196388
C	-1.58743049534094	-2.93585815037809	0.41707078063271
N	-1.78257136730939	-4.21850402112970	0.00353983452283
C	-2.60481732693811	-4.42513047161663	-0.99154782667454
C	-3.29099184622213	-3.38330231921604	-1.66577035585480
C	-1.95687241072794	-0.51950767971154	0.34867960941820
C	-1.03074490202071	-0.34246255783679	1.36240204569072
C	-0.38290855766284	-1.46360614286194	1.94526518777441
C	-0.67062060437637	-2.72312004843539	1.47909397071500
C	-5.06620747732981	-0.49403183908797	-1.16120227749291
N	-5.66349602120279	0.71637043059755	-1.73686278342963
C	-6.53054530776352	0.38571456006267	-2.87477934829581
C	-6.13816530516553	-1.57776634232342	-0.91165720790371
C	-7.75848753719060	-0.44306878714616	-2.41448447233334
C	-7.50637739222748	-0.88469430981522	-0.96801774505933
C	-6.46628533809662	1.36831878621451	-0.69563110811266
C	-7.48817310229714	0.37869918133351	-0.07147643015918
C	-7.26340670064095	0.00302895266651	1.37183697546751
C	-6.27573943565434	0.37522843566932	2.18845546919146
N	-3.08429341956592	0.16015106246998	-2.38387567776162
F	1.44034970367200	7.04586355224277	-2.22701435327297
F	0.67619818706443	7.28486528157258	-0.23574749106881
F	2.77601745608125	6.97229827551929	-0.54024836753495
C	1.56759783136871	6.61002258806896	-0.97023987944493
F	5.92745477646332	-1.45200352093639	4.07538155789685
F	4.12879942478942	-0.81619843685147	5.05382085859808
F	4.53720459523396	-2.91607222910464	4.80983272293685
C	4.62626338306590	-1.72774924105457	4.21252417893647

C	2.50356207102162	-4.09228581602094	0.33135006436082
F	1.55297534523730	-3.89047910029744	-0.58138705607877
F	2.03673852817020	-4.98771134252250	1.20905889703299
F	3.53377814561208	-4.67203586075366	-0.29440037165602
O	-0.77532544216051	0.93104493859494	1.73325548648762
C	0.10579743156866	1.16400432922157	2.81330124562105
H	-1.76984529743294	3.01173269126291	-2.52491590674785
H	0.51719596624733	1.40881741792029	-0.41616146710165
H	2.98510880425781	4.79914358291257	0.51387217514034
H	-0.25849857044819	5.17221624545923	-2.33138371529152
H	2.38557258707087	0.67662795715050	-0.55543484298520
H	2.18305271055873	-1.54145427567995	-0.56682384614644
H	3.68846937112255	-3.84988412990670	2.76036220993587
H	4.01278376104215	0.45998804465813	2.78526422285928
H	-4.30013524227829	-1.46518373251132	-2.93781193774069
H	-2.75115012398212	-5.46027400037613	-1.31649545278845
H	-3.92993096588539	-3.62793496873880	-2.51637305514335
H	-2.43370191767853	0.37587883887351	-0.04686713304858
H	0.34740325031753	-1.33781715935012	2.74488945572363
H	-0.18148372668033	-3.60106003010925	1.90398116027503
H	-4.63923438902004	-0.18189016401655	-0.19549734896281
H	-6.84106029846362	1.32758679505237	-3.34838457549437
H	-5.93590930416225	-0.16351280821832	-3.61948917070423
H	-6.09207602450027	-2.36111122282360	-1.68614920344437
H	-5.97225700304334	-2.06652424932170	0.05896104942594
H	-8.67958604266727	0.15692414656070	-2.46364049281177
H	-7.90116747460692	-1.32239818140495	-3.05951048355807
H	-8.29651038722575	-1.56985935880674	-0.62831859402199
H	-6.98922387107315	2.21597743752119	-1.16000386473209
H	-5.78701313694989	1.78333440964961	0.06103710950153
H	-8.49735712545122	0.82018259407147	-0.13023162631695
H	-8.02545022806946	-0.67939742241333	1.76964492840669
H	-6.24255765001530	0.01133044932885	3.21777912552035
H	-5.46928007705077	1.04629566614057	1.88129397057135
H	-3.57464111780319	1.04444209108793	-2.22586331523954
H	0.22338101592138	2.25080934239738	2.89090580002533
H	-0.30679448634291	0.76711607395271	3.75416000681264
H	1.09345964266791	0.70944366840633	2.63702682259121

30

2a DZ=-1635.379599037188 TZ=-1636.711000965312 Free ECorr=0.17883861

C	-3.48629094499795	0.69725377190271	-0.23604999067374
C	-4.17177276611219	1.83110483046866	0.19984619669026
C	-3.49446227663573	2.88719145514938	0.81919841352548
C	-2.11455816833467	2.83537772153965	1.01713262066625
C	-1.43781671229106	1.70473124234345	0.57240346511238
C	-2.11073618186750	0.65377134292383	-0.03683031749941
S	-1.08371342384024	-0.68388378837512	-0.60460611298877
C	0.39609875624315	-0.17715160212325	0.25898319174526
S	0.32958237704071	1.54227947486096	0.72965720976135
O	0.97755473451802	2.37625152553279	-0.27510433042968

O	0.70472485946364	1.70277688688499	2.12663628925634
O	-1.54412078922655	-1.96148420527378	-0.08160537496863
O	-0.89233616955741	-0.54368615798613	-2.03992228586229
C	1.34006370701255	-1.08974368775930	0.54112965483116
C	2.65543318362674	-0.89509671932414	1.14767074173699
C	3.33997622950393	0.33198940967660	1.10799959563753
C	4.58556765644380	0.45093122492873	1.71470710894962
C	5.16068765461999	-0.64594675115577	2.36115034324715
C	4.49838754391809	-1.87457781573879	2.38516302393677
C	3.25697744982283	-2.00215773165134	1.77005039033716
H	-4.00563706599390	-0.13325484614922	-0.71726661767245
H	-5.25119709996051	1.89232116049404	0.05717762861493
H	-4.05157412112466	3.76289760938792	1.15448614654857
H	-1.57873342519339	3.64975705319395	1.50817939895032
H	1.08195277038878	-2.12687103761789	0.28806591782860
H	2.92221980650189	1.18421721631504	0.57072723629962
H	5.11797965032259	1.40217155285783	1.67496013412503
H	6.13887000740559	-0.54469370404071	2.83445419624879
H	4.95511560000974	-2.73493495273667	2.87591700818065
H	2.73485715829368	-2.96124047852844	1.77388911786471

49

3a DZ=-1583.299203876115 TZ=-1584.752800881469 Free Ecor=0.34473774

C	0.95228341275440	4.90727913651349	-0.16613320390873
C	1.20148604999808	3.53883933784081	-0.31868385627373
C	0.33533627775196	2.74619990868828	-1.08025579731446
C	-0.78856321253649	3.32179832251352	-1.67134353562658
C	-1.05529092692668	4.68012543705421	-1.49068650705988
C	-0.18449055346733	5.47269586527346	-0.73950847101984
C	2.46067867720900	2.98281111012295	0.26641642085184
O	3.52625800991954	3.54294819090054	0.15516330259300
N	2.35175405819024	1.76714996866021	0.94200984357539
C	3.41154957694981	0.94158321761619	1.28955372012473
N	3.08487869052372	-0.11248713708205	1.97588822974106
C	1.70744225250158	-0.06722157773812	2.17870175120019
C	1.14234327824733	1.21303752269533	1.55513408221027
S	5.03927214408437	1.30180153228258	0.79637145871382
C	5.81457328187218	-0.31173765104560	1.18224452109686
C	5.49540762199732	-1.37229868958551	0.16222201077510
C	4.71785804161627	-2.48463390282883	0.50039443218581
C	4.43097165257020	-3.46317415261067	-0.45381040788303
C	4.91589244734528	-3.33776527053190	-1.75535366428577
C	5.69274977898546	-2.22873650960893	-2.10060147852330
C	5.97977052551870	-1.25446272165560	-1.14769836997129
O	1.06128985117811	-0.88495090216718	2.78185362099863
C	0.52484212875802	2.14115818733364	2.61926760273950
C	-0.88472469012295	1.73451399704319	2.96882509252852

C	-1.17119436179931	0.98490872185784	4.11352352249181
C	-2.48366070965234	0.60790638877299	4.40143329691987
C	-3.52210335434941	0.97523859213301	3.54481652140805
C	-3.24494474360337	1.72309260370246	2.39863860323089
C	-1.93340929701245	2.10004482444237	2.11441143580261
H	0.39301788606211	0.94883433414696	0.79665785092017
H	6.88624897644742	-0.06764664713269	1.17979747733573
H	5.51520627256182	-0.60501607119554	2.19430299067811
H	6.58474934360536	-0.38569294919864	-1.42080491894228
H	6.07959282737604	-2.12491412601119	-3.11597527074580
H	4.69351321958018	-4.10341402892745	-2.50079574589449
H	3.82425112161934	-4.32713035608923	-0.17641682880487
H	4.32912308159156	-2.57533523992947	1.51598251141776
H	0.55172896158242	1.68771719637874	-1.23850105694275
H	-1.45472954560386	2.70679842509031	-2.27796477883814
H	-1.94184234342402	5.12551917235754	-1.94504934806399
H	-0.38878195710521	6.53568497032562	-0.60436834632345
H	1.64739713406072	5.51812742474826	0.41285429504249
H	0.50863550484542	3.16307587333884	2.21358622175192
H	1.17072228587399	2.14704178070043	3.50995625418682
H	-1.71252597777078	2.69166967144275	1.21994713487068
H	-4.05399956095510	2.01824640916673	1.72825491209038
H	-4.54898899785264	0.68383966063459	3.77252218897919
H	-2.69714445509335	0.02457491339210	5.29884240277414
H	-0.35670168590276	0.69096623616862	4.77870787718699
